# Enantiospecific Synthesis of Planar Chiral Rhodium and Iridium Cyclopentadienyl Complexes: Enabling Streamlined and Computer-Guided Access to Highly Selective Catalysts for Asymmetric C–H Functionalizations

**DOI:** 10.1021/jacs.4c13279

**Published:** 2024-12-06

**Authors:** Young Sebastian Ye, Aragorn Laverny, Matthew D. Wodrich, Ruben Laplaza, Farzaneh Fadaei-Tirani, Rosario Scopelliti, Clemence Corminboeuf, Nicolai Cramer

**Affiliations:** † Laboratory of Asymmetric Catalysis and Synthesis, Institute of Chemical Sciences and Engineering, Ecole Polytechnique Fédérale de Lausanne (EPFL), Lausanne 1015, Switzerland; ‡ Laboratory for Computational Molecular Design, Institute of Chemical Sciences and Engineering, Ecole Polytechnique Fédérale de Lausanne (EPFL), Lausanne 1015, Switzerland; § X-Ray Diffraction and Surface Analytics Facility, Institute of Chemical Sciences and Engineering, Ecole Polytechnique Fédérale de Lausanne (EPFL), Lausanne 1015, Switzerland

## Abstract

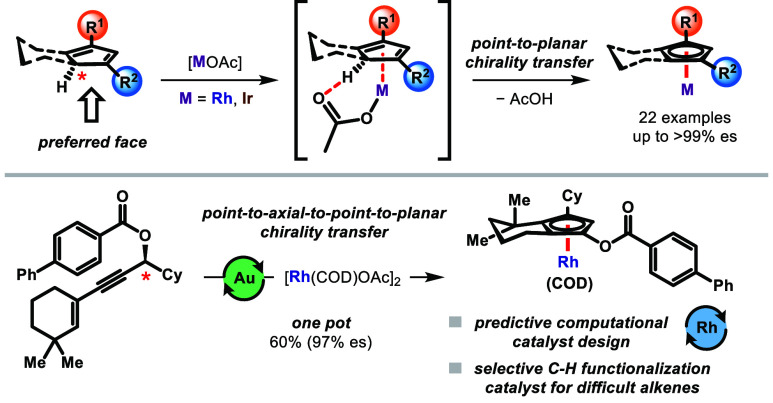

Chiral cyclopentadienyl (Cp^X^) metal complexes are frequently used in asymmetric catalysis by virtue of their high reactivity and selectivity. Planar-chiral-only rhodium and iridium cyclopentadienyl complexes are particularly promising due to unrestricted chemical space for Cp^X^ ligand design while retaining structural simplicity. However, they are currently still niche because of a lack of efficient synthetic strategies that avoid lengthy chiral auxiliary routes or chiral preparatory HPLC resolution of the complexes. To streamline access to such planar-chiral-only Cp^X^-metal complexes, we designed a straightforward, highly enantiospecific, point-to-planar chirality transfer complexation via facially selective concerted-metalation-deprotonation between metal-carboxylate precursor [M(olefin)_2_OAc]_2_ and a chiral cyclopentadiene. This entirely avoids the typical stereoablative complexation of an achiral cyclopentadienyl anion that detrimentally yields a racemate. Exploiting the described enantiospecific complexation protocol and a simple divergent synthetic route to suitable chiral cyclopentadienes, we generated a structurally diverse library of new planar chiral Cp-Rh(I), Cp-Ir(I), Cp-Rh(III), and Cp-Ir(III) complexes. Moreover, the enantiospecific complexation step can be concatenated with a preceding Au-catalyzed cyclization in an efficient one-pot process that likely involves an elaborate point-to-axial-to-point-to-planar chirality transfer. Guided by computational selectivity predictions, the structure of a Cp^X^-Rh complex in our library was tuned to optimize reactivity and selectivity in the asymmetric C–H functionalization of a benzamide with various challenging alkenes. With an optimized Cp^X^-Rh complex in hand, we showcased its excellent catalytic performance and high selectivity for refractory alkene substrates that reacted in poor selectivity with previous Cp^X^-Rh catalysts.

## Introduction

Chiral cyclopentadienyl (Cp^X^) ligands have been used as a stereochemical element in transition-metal-based asymmetric catalysis to great success, delivering high performance with respect to both reactivity and selectivity.^1^ For example, Cp^X^ complexes of Group 9 metals (Co, Rh, and Ir) have enabled challenging enantioselective C–H functionalization reactions to be carried out.^2^ Other attractive characteristics of Cp^X^-metal complexes include low molecular weight, simplicity, rigidity, and a largely unexplored chemical space. Despite this, fundamental problems exist in their preparation on a general level.

**Scheme 1:**
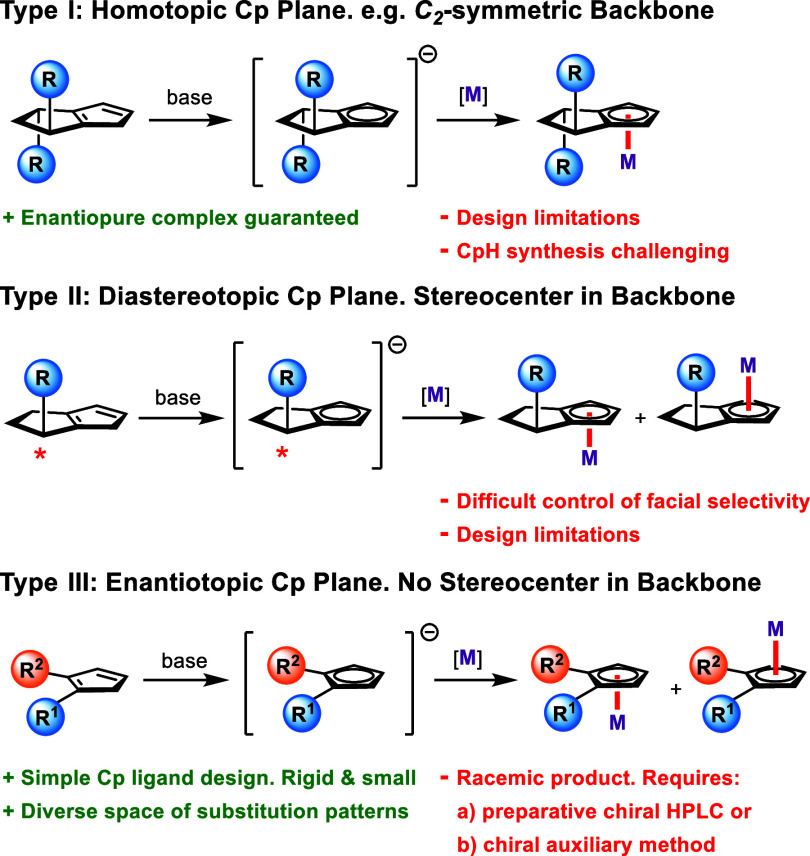
Overview of Three Different Cp-metal Complexation Outcomes Depending on Cp Anion Geometry

Traditionally, Cp^X^-metal complexes are formed by deprotonation of a cyclopentadiene (CpH) and subsequent complexation (i.e., transmetalation) of a metal precursor.^3,4^ The geometry of the intermediate Cp anion falls into one of three possible categories: (I) homotopic, (II) diastereotopic, and (III) enantiotopic (Scheme 1). By far the most common geometry type seen in literature is Type I, in which a corresponding CpH typically contains a *C*_2_-symmetric (chiral) backbone.^5−11^ The metal complex formed from a Type I anion is always enantiomerically pure, as complexation to either face of the Cp anion is identical. This design avoids any need for chiral separation postcomplexation. However, Type I CpHs with different chiral backbones do not share a common synthetic intermediate, and therefore, traversal of chemical space can be tedious. Additionally, Cp^X^-metal complexes with *C*_2_-symmetric backbones are not structurally optimal as only one half of the backbone (facing the metal) significantly affects the relevant chiral environment. A non-*C*_2_-symmetric cyclopentadiene with one or more stereogenic centers in its backbone will generate a Type II Cp anion.^12^ Complexation in this case leads to two possible diastereomers. Good control of facial selectivity in the complexation of Type II anions is rare and restricted to very specific examples. For instance, Loginov describes one diastereoselective complexation of RhCl_3_ with an indene containing an α-pinene-derived backbone.^13^ Wang reports a diastereoselective complexation of [Rh(COD)Cl]_2_ with a planar chiral indene containing a [2.2]benzoindenophane backbone.^14^ However, other examples of Type II anion complexation in literature are not selective,^15^ indicating the highly restrictive nature of such CpH backbone design. The Type III Cp anion is prochiral and, upon addition of a binding metal, delivers a complex that is planar chiral without other sources of chirality (planar-chiral-only). Assuming that the typical transmetalation process takes place, there is no facial selectivity in their complexation, and the resulting product is a racemate. Nevertheless, CpHs that lead to Type III anions have a large design space as they do not need to be chiral themselves and do not require a structurally elaborate backbone.

To our knowledge, all literature reports of synthesized planar-chiral-only Cp^X^-metal complexes (Type III) describe the need for some variation of chiral separation of the complexes themselves.^16,17^ There exist enterprising methods to, at the very least, avoid the use of chiral preparatory HPLC. Perekalin has previously used a chiral resolution strategy that involved addition of a chiral amine or amino acid to a racemic Cp^X^-Rh(III) complex, followed by diastereomeric separation and finally removal of the auxiliary.^18,19^ A clear drawback to this approach is the number of extra operational steps. More recently, Wang^20^ reported a unique protocol using a tailored [Rh(chiral diene)OAc]_2_ complex. Direct complexation with achiral CpH led to a diastereomeric mixture that was separable by flash column chromatography. While an improvement over existing methods, this strategy still required effort in synthesizing the chiral diene auxiliary, and the facial selectivity of the complexation ranged from good to very poor, depending on the substitution pattern of the CpH.

**Scheme 2:**
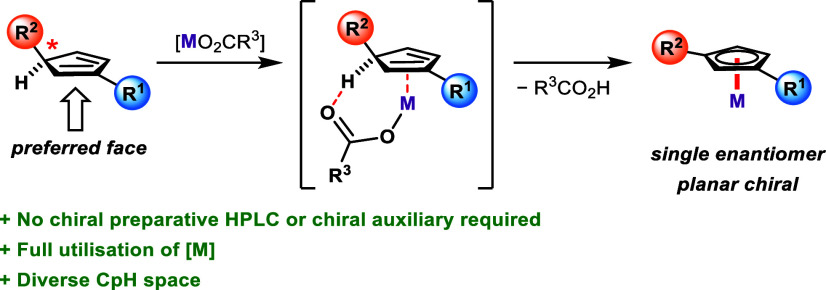
Enantiospecific Complexation of CpHs Containing a Core Tertiary Carbon Stereocenter via Point-To-Planar Chirality Transfer

In general, as illustrated with the various caveats of the Type I–III Cp anions, the synthesis of enantiopure planar chiral Cp^X^-metal complexes remains challenging. This makes the development of a globally applicable facially selective complexation strategy a high priority endeavor. *However, this could only be made possible by escaping the paradigm of deprotonation and complexation occurring in separate steps.* Carboxylate ligands in metal complexes can facilitate C–H activation of appropriate substrates via a concerted metalation-deprotonation (CMD) mechanism. This has not only been long exploited in C–H functionalization reactions^21^ but also more recently to prepare derivative complexes under mild conditions.^22,23^ One such complexation protocol, which uses either [Rh(COD)OAc]_2_ or [Ir(COD)OAc]_2_, circumvents the need for any additional base. We reasoned, using the same or similar conditions, that an appropriate CpH with a core tertiary carbon stereocenter could undergo metal complexation preferentially on the same face as its tertiary hydrogen through a CMD pathway (Scheme 2). The process would in effect be a full point-to-planar chirality transfer and, unlike the examples in Scheme 1, not involve formal deprotonation of a CpH.

We herein report a very successful proof of this concept, establishing a general method for the enantiospecific complexation of chiral CpHs with [M(olefin)_2_OAc]_2_ precursors. We also demonstrate that several of the new Cp^X^-Rh complexes prepared by this approach set new performance benchmarks in asymmetric C–H functionalization reactions.

## Results and Discussion

**Scheme 3:**
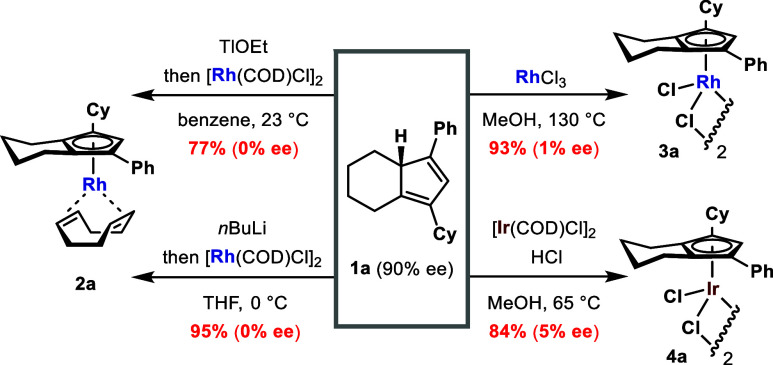
Application of Typical Complexation Protocols on Enantioenriched CpH 1a

Using compound **1a**, an enantioenriched CpH that importantly contained a single tertiary carbon stereocenter in its core, we first applied typical complexation methods to establish a baseline for chirality transfer, or to confirm its absence (Scheme 3). The reaction between **1a** and [Rh(COD)Cl]_2_ in the presence of thallium ethoxide^5^ delivered Rh(I) complex **2a** in 77% yield as a fully racemic mixture. When *n*BuLi^11^ was used as the base, **2a** was formed, again with full stereochemical ablation. Clearly, in both of these cases, complexation occurs from the generated achiral Cp anion with no facial selectivity. The microwave-assisted reaction of **1a** with RhCl_3_ afforded [Cp^X^RhCl_2_]_2_**3a** in 93% yield.^24^ Chiral SFC analysis of the trimethyl phosphite derivative of **3a** confirmed that a virtually racemic product was formed. Finally, a complexation attempt using [Ir(COD)Cl]_2_ and aqueous HCl gave the analogous Ir(III) complex **4a** in 84% yield.^25^ Again, trimethylphosphite derivatization and chiral SFC analysis were carried out to indicate this complexation proceeded with only negligible enantiospecificity. *Therefore, none of these traditional methods allow any significant degree of chirality transfer of CpH to a planar chiral Cp*^*X*^*-metal complex.*

We then screened various reaction conditions for the complexation of **1a** and different rhodium and iridium precursors (Table 1). A 1:1 toluene/methanol mixture was optimal in balancing between dissolution of reactants and deaggregation of the [M]_2_ dimer. After 3 h of stirring at ambient temperature, the desired chiral Cp^X^-Rh(I) complex **2a** was cleanly formed and isolated in 83% yield. Pleasingly, the enantiomeric excess of **2a** was 90%, corresponding to >99% enantiospecificity (es) and a full transfer of point chirality of **1a** to planar chirality of **2a**. Using alternative complexes [Rh(COD)OMe]_2_ and [Rh(COD)OH]_2_ also yielded **2a** in full enantiospecificity, albeit with somewhat lower yields (**entries 2** and **3**). No complexation was observed at all when using [Rh(COD)Cl]_2_ (**entry 4**), providing evidence that CMD facilitated by an anionic oxygen-containing ligand was indeed occurring with the other precursors. [Rh(COD)Cl]_2_ could instead be converted to the [Rh(COD)OAc]_2_ in situ with KOAc (**entry 5**), and these conditions delivered **2a** but in both lower yield and enantioselectivity compared to **entry 1**. This outcome can be explained by a possible competing pathway involving coordination of the metal to the “wrong” face of CpH and then CMD with an external acetate. The reaction of **1a** with iridium analogue [Ir(COD)OAc]_2_ proceeded more slowly than that for rhodium (**entry 6**), requiring elevated temperature to deliver **5a** in 73% yield (**entry 7**) but also with complete enantiospecificity. Complexation of **1a** with [Ir(C_2_H_4_)_2_OAc]_2_ generated in situ from [Ir(COE)_2_Cl]_2_, KOAc, and ethylene gas (**entry 8**) delivered the bis–ethylene complex **5b** in 86% yield, again with full chirality transfer.^26^

**Table 1:**
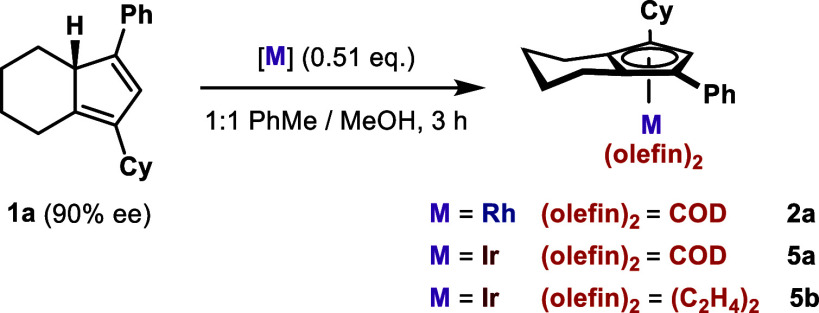
Optimization of the Enantiospecific Complexation

entry	[M]	*T* (°C)	% yield	% es
1	[Rh(COD)OAc]_2_	23	83	>99
2	[Rh(COD)OMe]_2_	23	63	>99
3	[Rh(COD)OH]_2_	23	70	>99
4	[Rh(COD)Cl]_2_	23	0	n.d
5^a^	[Rh(COD)Cl]_2_	23	59	79
6	[Ir(COD)OAc]_2_	23	<5	n.d
7	[Ir(COD)OAc]_2_	70	73	>99
8^b^	[Ir(COE)_2_Cl]_2_	60	86	>99

a Isolated yields shown. Enantiospecificity calculated as ee of complex ÷ ee of CpH. with 1.2 eq. KOAc.

b With 2.0 eq. KOAc under ethylene atmosphere for 16 h. n.d. = not determined.

**Scheme 4:**
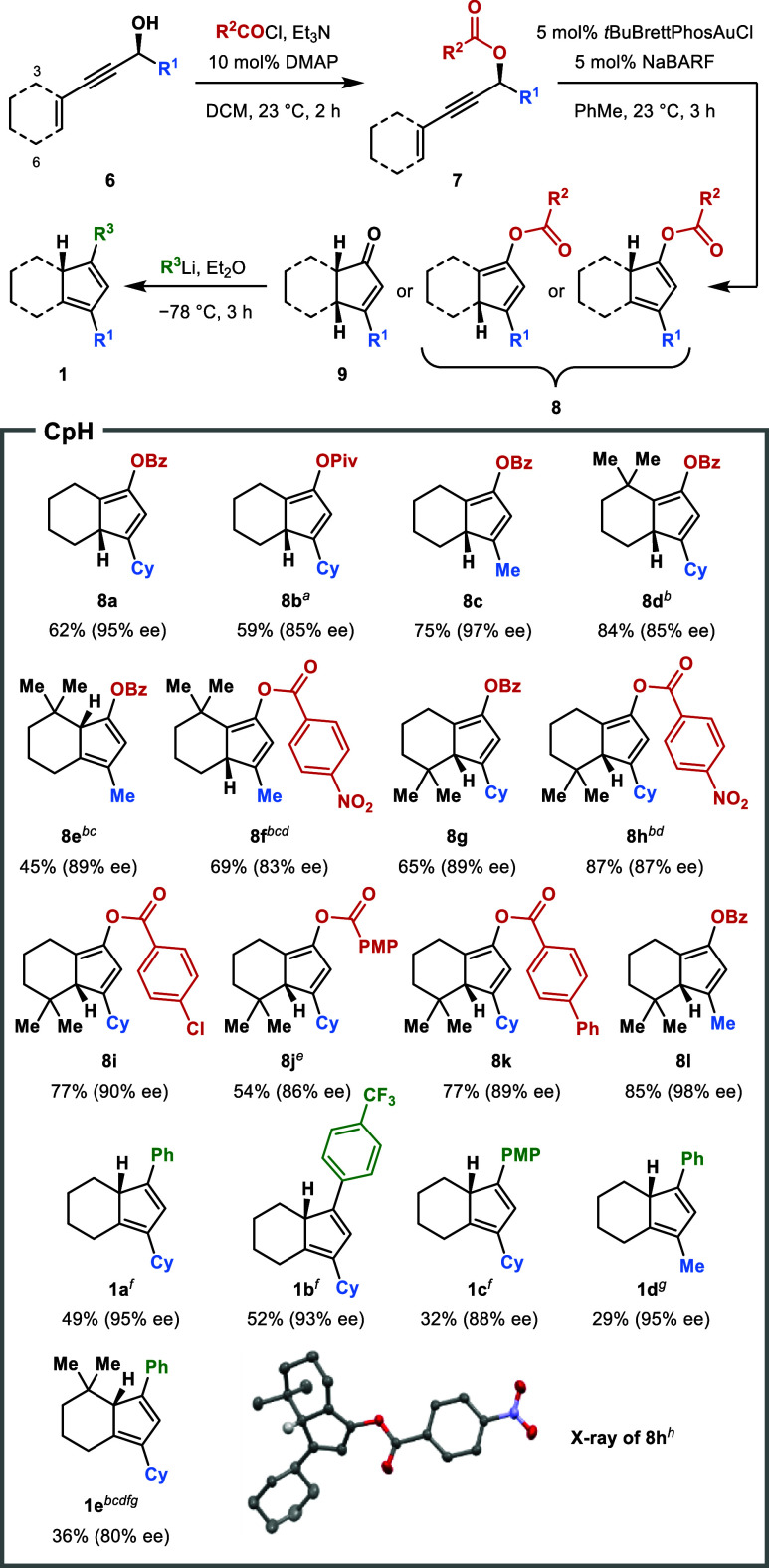
Enantioselective Synthesis of Chiral CpHs Isolated yields over multiple steps from **6** are shown. Alkynylation and acylation were performed in one pot. AgSbF_6_ instead of NaBARF. PPh_3_AuCl instead of *t*BuBrettPhosAuCl. 10 mol % of additive. Acylation reaction time of 18 h and cyclization time of 6 h. Wet DCM as the solvent. During workup of ArLi addition, 1.5 equiv of 2-NsCl was added to induce dehydration of the direct addition intermediate. 50% probability thermal ellipsoids. Hydrogen atoms omitted for clarity, except for on the stereocenter.

Encouraged by these initial results, we then sought to build a library of chiral CpHs containing a single core stereogenic tertiary carbon and demonstrate that the enantiospecific complexation method is applicable to all of these compounds. A moderately sized collection of these CpHs was obtained (several on gram scale) via a short divergent sequence, as described in Scheme 4, starting from a variety of chiral propargylic alcohols (**6**). Compounds **6** all contained a cyclohexenyl group, and some contained a 3,3-dimethyl or 6,6-dimethyl substitution pattern on the cyclohexene ring. **6** was prepared either by the Sonogashira coupling of a cyclohexenyl triflate with an appropriate alkynol, or by Carreira alkynylation of aldehydes.^27^ Esterification of **6** with a variety of acyl chlorides yielded **7**, from which a gold-catalyzed cyclization protocol was carried out according to Zhang.^28^ Depending on the substrate and the conditions of the cyclization reaction, either cyclopentadienyl ester **8** (one of two isomers) or cyclopentenone **9** was obtained as the major product, generally with a high degree of chirality transfer. Replacing *t*BuBrettPhosAuCl with PPh_3_AuCl and replacing NaBARF with AgSbF_6_ led to a higher reaction rate, which was necessary for full conversion in some cases. When wet solvent was used, **9** would be preferentially formed over **8**, presumably by some hydrolytic process taking place. Arylation of **9** subsequently yielded a group of aryl-substituted chiral CpHs, including the first example **1a**. Acyloxy-substituted CpHs such as **8** are previously *completely unexplored candidates* for metal complexation. Overall, a chemical space for **8** and **1** was traversed in three dimensions: (a) presence of a *gem*-dimethyl group on the cyclohexane, (b) the alkyl substituent on the Cp, and (c) the acyloxy/aryl group.

**Scheme 5:**
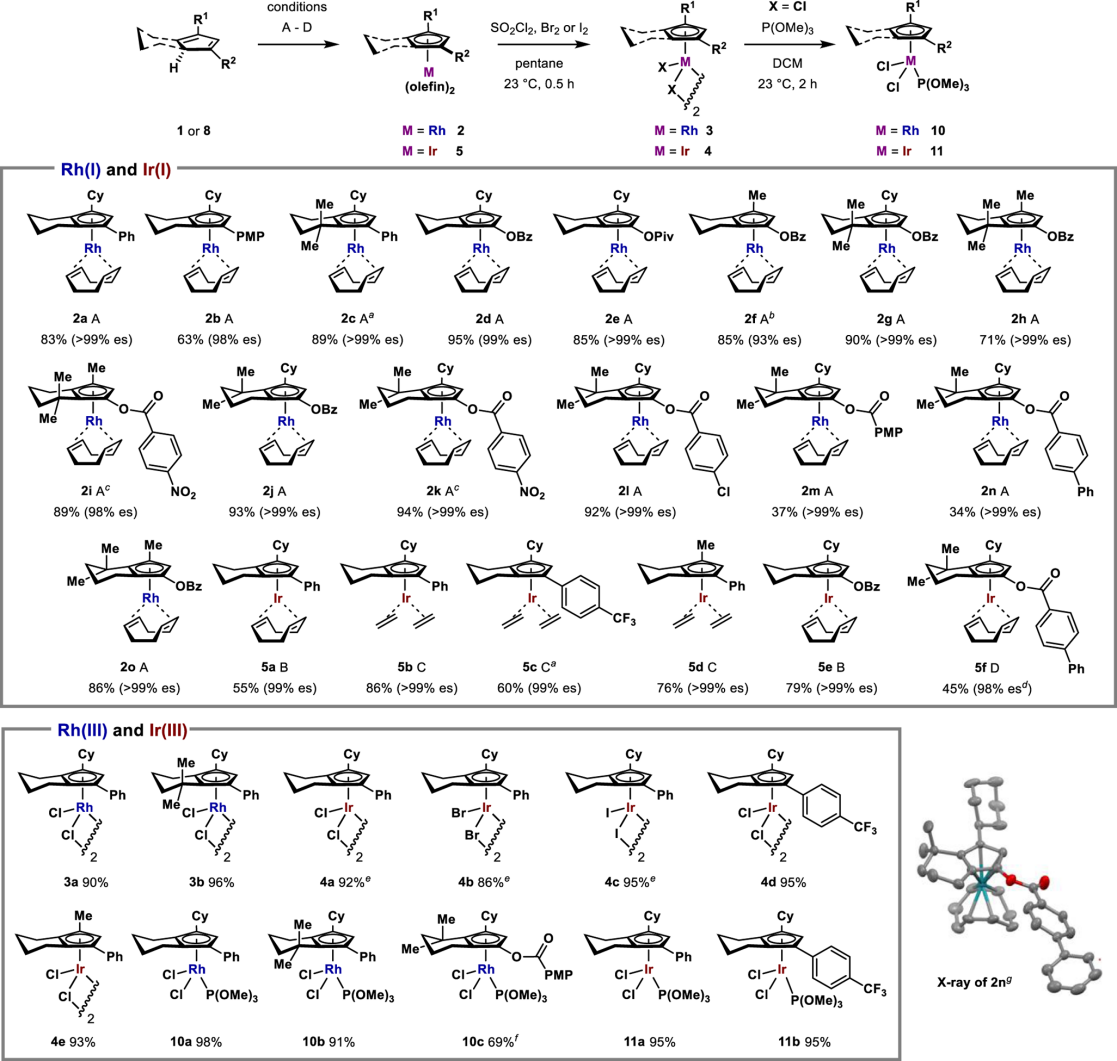
Planar Chiral Cp^X^-Rh(I) and Cp^X^-Ir(I) Complexes Formed by Enantiospecific Complexation of Chiral CpHs, and Subsequent Cp^X^-Rh(III) and Cp^X^-Ir(III) Complexes Conditions A: [Rh(COD)OAc]_2_ (0.51 equiv) in 1:1 PhMe/MeOH at 23 °C for 3 h. Conditions B: [Ir(COD)OAc]_2_ (0.51 equiv) in 1:1 PhMe/MeOH at 70 °C for 3 h. Conditions C: [Ir(COE)_2_Cl]_2_ (0.60 equiv), KOAc (2.0 equiv) in 1:1 PhMe/MeOH under C_2_H_4_ atmosphere at 60 °C for 16 h. Conditions D: Used crude CpH, [Ir(COD)OAc]_2_ (0.51 equiv) in 1:1 PhMe/MeOH at 40 °C for 16 h. Isolated yields of the last step shown, unless stated otherwise. Enantiospecificity calculated as ee of complex ÷ ee of CpH, determined from chiral HPLC analysis unless indicated otherwise. ee of complex was determined by chiral HPLC or SFC analysis of the [Cp^X^MCl_2_(P(OMe)_3_)] derivative. Reaction temperature of 0 °C and time of 30 min. Reaction time: 16 h. Isolated yield over two steps, enantiospecificity determined from ee of crude CpH. Synthesized from **5b**. Isolated yield over two steps from **2m**. 50% probability thermal ellipsoids. Hydrogen atoms omitted for clarity.

We found that all of the synthesized CpHs (**8** and **1**) were amenable to enantiospecific complexation, delivering a range of unique planar-chiral-only Cp-Rh(I) and Cp-Ir(I) complexes in generally high yield and high enantiopurity (Scheme 5). There was virtually complete point-to-planar transfer of chirality over nearly the entire chemical space of CpHs. The presence of the acyloxy group on the Cp ring was well tolerated despite initial concerns with electrophilicity. All the Rh(I) and Ir(I) complexes generated were stable under ambient conditions and could be purified by typical chromatographic methods without precaution to moisture or air.

We also demonstrated that various Rh(I) and Ir(I) complexes (**2** and **5**) could be readily oxidized into the catalytically active Rh(III) and Ir(III) halide complexes (**3** and **4**) by treatment with sulfuryl chloride, bromine, or iodine.^29−31^ Several of the M(III) chlorides, in particular, were also converted into the [Cp^X^MCl_2_(P(OMe)_3_)] derivatives (**10** and **11**).^32^ M(III) complexes with an aryl substituent on the Cp ring were all completely stable under ambient conditions. The Rh(III) chloride derivative of **2m**, containing an acyloxy group, proved to be a transient species (likely susceptible to hydrolysis), but immediate conversion to the trimethylphosphite derivative yielded stable complex **10c**.

**Scheme 6:**
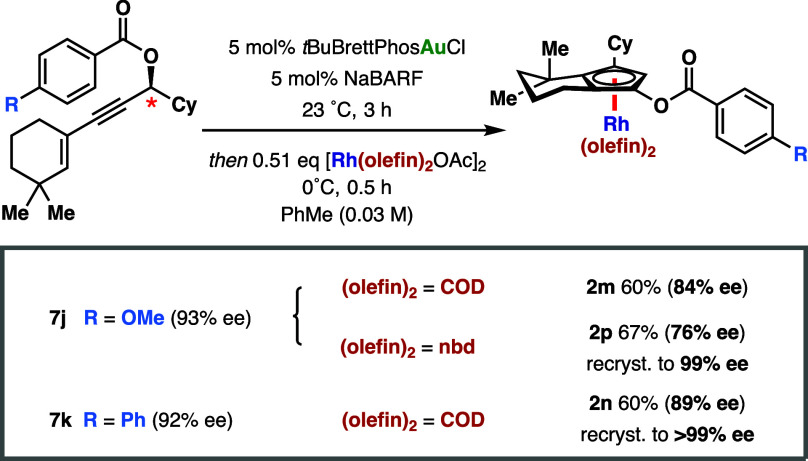
Point-to-Axial-to-Point-to-Planar Chirality Transfer in a One-Pot Synthesis of Cp^X^ -Rh(I) Complexes 2m, 2n, and 2p

**2m** and **2n**, bearing 4-methoxybenzoyloxy and 4-phenylbenzoyloxy groups, respectively, became important complexes in later stages of the study as they proved to be highly competent in asymmetric C–H functionalization. However, their corresponding yields following the method in Scheme 5 were disappointingly low due to the difficulty in isolating the sensitive electron-rich CpHs **8j** and **8k**. Logically, we then sought to develop a protocol that combined both the preceding gold-catalyzed cyclization and enantiospecific complexation steps in a single pot. This proved to be highly successful, allowing complexes **2m** and **2n** to be furnished in much improved yields and with almost no decrease in enantiopurity compared to the stepwise approach (Scheme 6). This method constitutes, according to accepted mechanisms,^28^ an incredible *point-to-axial-to-point-to-planar chirality transfer in a single pot process*. In a scaled-up synthesis of **2n**, the desired complex was obtained in 60% yield over two steps in 89% ee and was then recrystallized to full enantiopurity. Because **2m** was not crystalline, its norbornadiene analogue (**2p**) was prepared and could then be recrystallized to afford the enantioenriched complex.

**Scheme 7:**
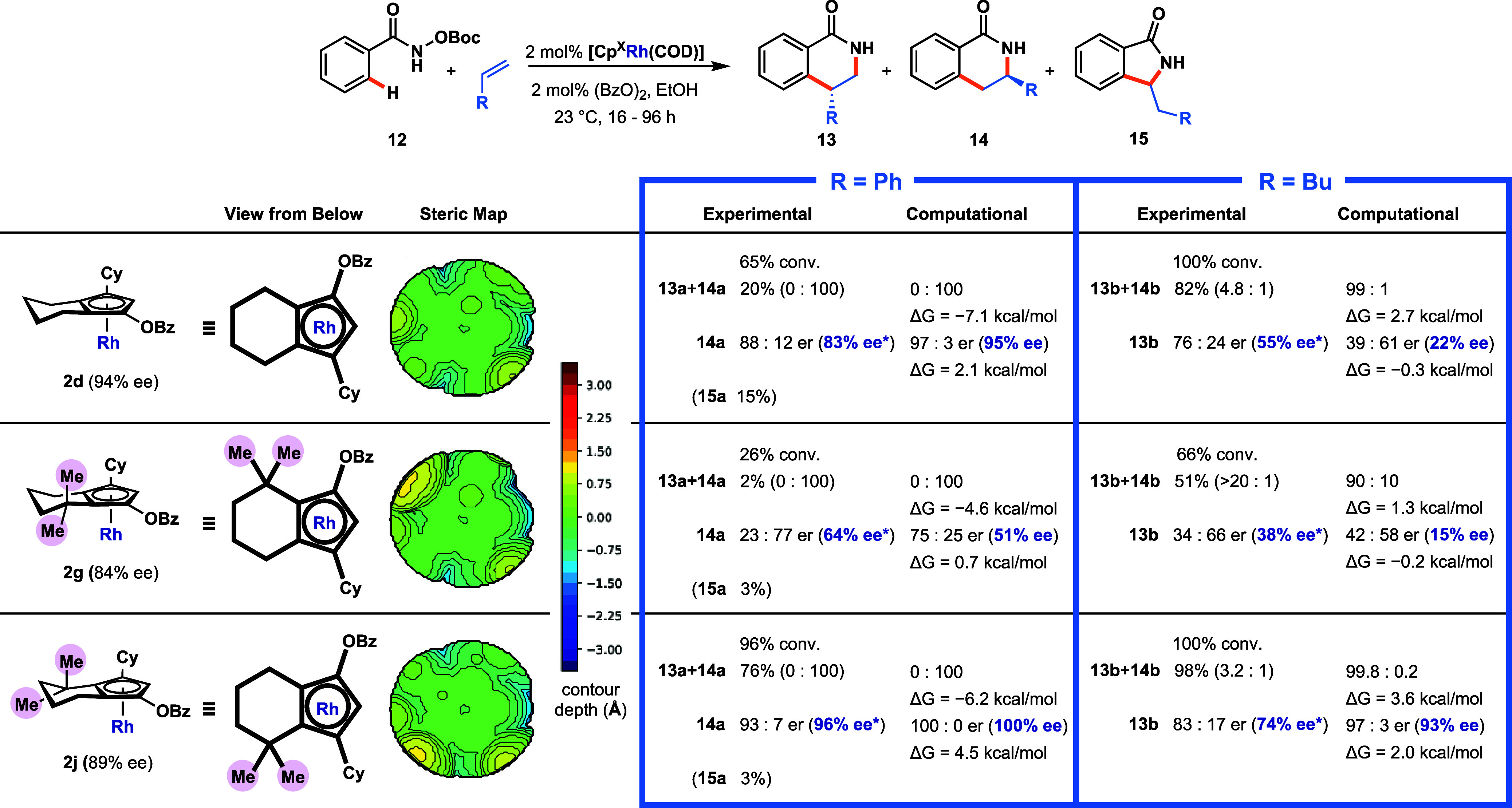
Steric Trend of Rh Complexes 2d, 2g, and 2j in the C–H Functionalization of Benzamide 12 with Styrene and 1-hexene, with Steric Maps and Computational Predictions Conversion determined by isolation of **12**. Isolated yields are shown. Adjusted enantiomeric excess ee* was calculated as ee of major product ÷ ee of complex, determined from chiral HPLC analysis. Steric maps generated using SambVca 2.1 (bondi radii scaled by 1.17, sphere radius 3.5 Å, mesh spacing 0.1 Å).^47^ Computed free energies were determined at the PBE0-D3(BJ)/def2-TZVP//PBE0-D3(BJ)/def2-SVP level (see Supporting Information Section 6 for more detail).

Our next major endeavor was to show the catalytic potential of this new library of Cp^X^-metal complexes. We evaluated their performance in the C–H functionalization of benzamide derivative **12** with various alkenes as this is a well-accepted benchmark transformation (Scheme 7).^5^ There exist numerous examples in literature in which reaction with cyclic alkenes (e.g., norbornene) and electronically activated alkenes (e.g., styrene) give high enantioselectivity,.^5,13,14,18,20,33−35^ However, simple unbiased terminal alkenes are far more challenging substrates, and regio- and enantioselectivity remain unsolved for their Rh(III) catalysis. We previously reported the C–H functionalization of *N*-chlorobenzamide with 1-hexene or 1-octene using a Cp^X^-Co catalyst, giving the 3-substituted dihydroisoquinolones exclusively with high enantioselectivity.^35^ In contrast, poor enantioselectivity has always been observed for the analogous Rh-catalyzed reaction in which the 4-alkyl-substituted product is primarily formed.^13,18−20,25^ We first attempted the C–H functionalization of **12** with styrene (**a**, R = Ph) and 1-hexene (**b**, R = Bu) using our first generation [Cp^X^Rh(COD)] complex **2d** under standard conditions that formed the catalytically active Rh(III) species in situ (Scheme 7).^5^ The reaction with styrene led to the formation of only the 3-substituted regioisomer **14a** along with side product isoindolinone **15a**. For 1-hexene, only the regioisomers **13b/14b** were produced, in strong favor of the 4-substituted **13b**. While typically enantioselectivity is judged from the ee of the product(s), to make fair comparisons, we used an adjusted enantiomeric excess of the product (ee*) that accounted for the less than full enantiopurity of the catalyst used. Using complex **2d,** across both reactions, good to moderate enantioselectivity was already observed (83% for styrene and 55% for 1-hexene). We then proposed to further improve catalyst performance by modifying **2d** to incorporate a *gem*-dimethyl group on the cyclohexyl backbone (analogous to a *tert*-butyl group) at one of two positions (**2g** and **2j**).

Prior to experimental work on these derivative complexes, computational predictions aimed at identifying the lowest energy transition states for the regio-/enantiodetermining step were undertaken for the C–H functionalization of **12** with styrene or 1-hexene, leading to dihydroisoquinolones (**13** and **14**) using complexes **2d**, **2g**, and **2j**. To accomplish this, we employed a computational workflow based on previous work that capably reproduced experimental enantioselectivites of *C*_2_-symmetric Cp derivatives in the rhodium-catalyzed C–H bond functionalization of hydroxamic acid derivatives.^36,37^ Beginning from eight preconstructed TS structural templates associated with formation of possible regio- and enantiomers (two each, leading to the 3R, 3S, 4R, and 4S products, see Supporting Information Figure S1) we used the SCINE Molassembler library^38,39^ to generate 3200 total conformers (400 for each of the possible pathways) with full stereoisomer control for each catalyst/substrate combination. Generated conformers that lead to chemically reasonable structures (i.e., those without overlapping chemical moieties) when projected back to three-dimensional coordinates from their graphs^38,39^ were then subjected to a series of constrained optimizations, at the PM7^40^ and PBE0^41,42^-D3(BJ)^43,44^/def2-SVP^45^ levels, followed by full TS optimizations at the PBE0-D3(BJ)/def2-SVP level and single point energies at the PBE0-D3(BJ)/def2-TZVP level (see Supporting Information Section 6 for full details). The optimized conformer ensembles associated with each pathway (i.e., 3DR, 3DS, 4DR, 4DS, etc.) were then analyzed using marc,^46^ a conformer analysis program that uses k-means clustering, in order to identify the ten most representative conformers for each of the aforementioned pathways. Those representative conformers having free energies within 4.0 kcal/mol from lowest energy species of the respective pathway were retained and used to determine effective Δ*G* values through Boltzmann weighting at 296 K. These Δ*G* values were subsequently used to determine regio-/enantioselectivity ratios from the theoretical kinetic constants:  as *rr*_4/3_ = 100 ×  and *k*_*S/R*_ =  as *er*_*S/R*_ = 100 × , respectively.

Across the three complexes, regioselectivity in the reaction with styrene was always predicted to be overwhelmingly in favor of the 3-substituted product **14a** (Δ*G*_eff,4/3_^*TS*^ > 4.50 kcal/mol for all ligands). For 1-hexene, the 4-substituted regioisomer **13b** was predicted to be the major isomer (Δ*G*_eff,4/3_^*TS*^ = 1.26 to 3.64 kcal/mol). Furthermore, the computational results (see the Supporting Information for a comprehensive comparison of experimental and computational selectivity data) suggested that, for both styrene and 1-hexene, a steric trend exists where placement of the *gem*-dimethyl group adjacent to the Cy group (**2j**) had a strongly positive effect on enantioselectivity for the major isomer compared to **2d**, while a negative effect is associated with placing the *gem*-dimethyl group adjacent to the OBz group (**2g**). With this insight in hand, analogues **2g** and **2j** were synthesized and tested. The relevant steric maps of **2d**, **2g**, and **2j** were also obtained from their respective X-ray structures. For complex **2g**, the steric bulk of the *gem*-dimethyl group was directly opposite that of the Cy group, which seems to largely nullify the steric bias. For complex **2j**, the *gem*-dimethyl group proximal to the Cy group appears to enhance the overall steric bias. From this primitive analysis, **2j** was expected to induce the highest enantioselectivity of the three. As predicted from the computations, in the reaction with styrene, the steric modification of complex **2d** did not affect its already excellent regioselectivity. Meanwhile, for 1-hexene, the observed regioselectivities for **2d**, **2g**, and **2j** were relatively consistent with predictions. At the same time, the steric environment of **2j** was predicted to induce superior enantioselectivity relative to **2d** and **2g**. Supported by prior rationale, complex **2j** indeed outperformed **2d** with 96% enantioselectivity in the reaction with styrene and 74% for 1-hexene. Catalytic reactivity was also improved over **2d** with 76% yield for styrene and 96% yield for 1-hexene.

**Scheme 8:**
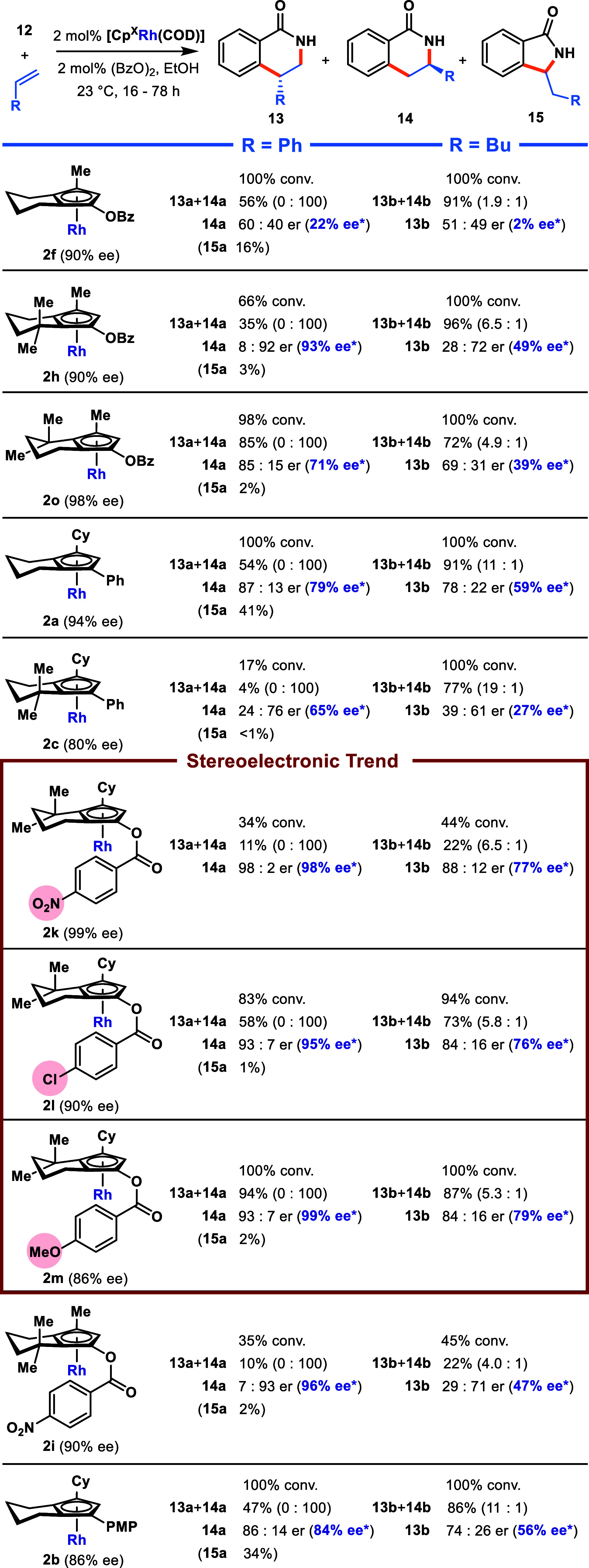
Screening of Other Rh Complexes in the C–H Functionalization of Benzamide 12 with Styrene and 1-Hexene Conversion determined by isolation of **12**. Isolated yields shown. Adjusted enantiomeric excess ee* calculated as ee of major product ÷ ee of complex, determined from chiral HPLC analysis.

Other [Cp^X^Rh(COD)] complexes in our library were then screened in the same C–H functionalization reactions of **12** with styrene and 1-hexene (Scheme 8). When examining the effect of *gem*-dimethyl group steric modification in the series **2f**/**2h**/**2o** and **2a**/**2c**, trends similar to those of **2d**/**2g**/**2j** for enantioselectivity were observed. However, none outperformed existing **2j**. Thus, we next investigated modifying the electron density of the benzoyl group of **2j** to determine stereoelectronic effects on the outcome of the reaction for the same two alkene substrates. A series of analogues **2k** (para-nitro), **2l** (*para*-chloro) and **2m** (*para*-methoxy) were prepared and subjected to the same catalytic screening. Interestingly, for both the reactions with styrene and 1-hexene, conversion and yield significantly increased as the electron density of the benzoyl group increased, with only slight differences in regio- and enantioselectivity. This is consistent with our assumption that introducing the distal para-substituent does not influence the steric environment already elucidated in **2j**. To justify the observed stereoelectronic trend with reactivity, we reason that for more electron-dense Cp ligands the Cp–Rh bond is less labile, and therefore, the active Cp-Rh(III) species is more long-lived and effectively leads to increased catalytic turnover.

**Scheme 9:**
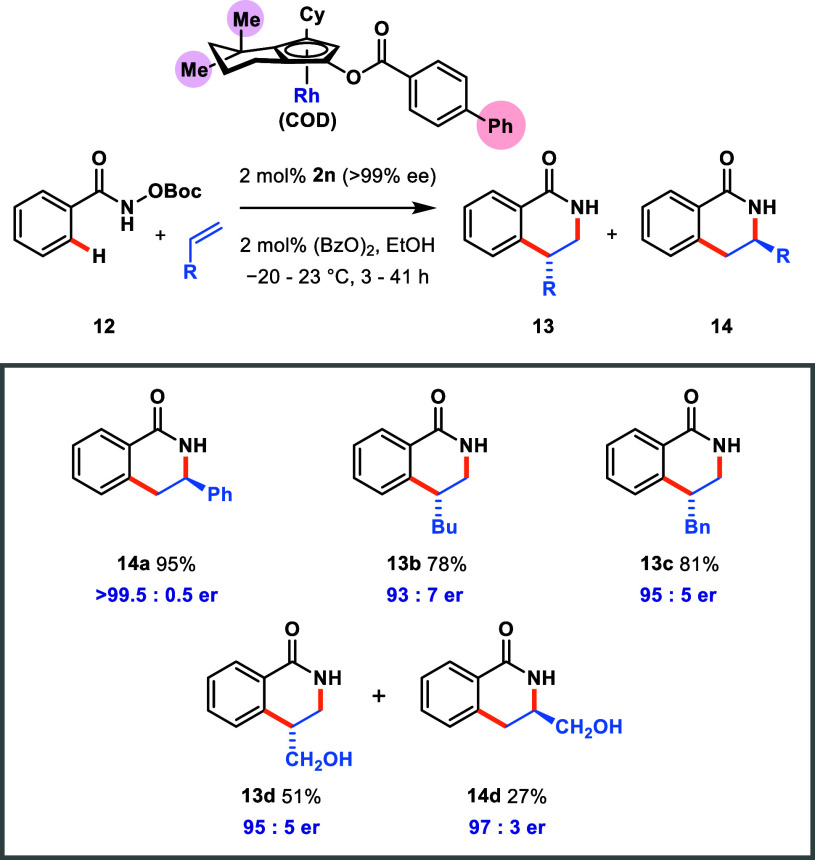
High Selectivity in the C–H Functionalization of Benzamide 12 with Various Alkenes Using Optimized Rh Catalyst **2n** Conversion determined by isolation of **12**. Isolated yields shown. Enantiomeric ratios were determined from chiral HPLC analysis.

Complex **2n** was then designed to combine ideal steric (*gem*-dimethyl substitution next to Cy), electronic (electron-rich para-phenyl substituent), and physical (easily crystallized to full enantiopurity) properties (Scheme 9). The methodology described in Scheme 4 and Scheme 6 allowed for the rapid synthesis of enantiopure **2n**. When subjected to the same C–H functionalization benchmarks as before, the fast catalytic turnover of **2n** could be exploited with a lower reaction temperature. In the reaction between **12** and styrene, using **2n** under optimized conditions, **14a** was formed in 95% yield with virtually full enantioselectivity (>99.5:0.5 er). Using the same reaction conditions with 1-hexene as the substrate, the major regioisomer **13b** was formed in 78% yield and 93:7 er. To date, this is by a margin the highest enantioselectivity seen for any existing Cp^X^-Rh complex using terminal unbiased alkenes. Finally, two other highly challenging substrates—allylbenzene (**c**, R = Bn) and allyl alcohol (**d**, R = CH_2_OH)—for which little to no selectivity was previously observed,^18^ were tested. Again, the high catalytic performance and selectivity of **2n** was demonstrated for both substrates, delivering their respective dihydroisoquinolones in good yield and with 95:5 er of both major regioisomers.

## Conclusions

In summary, we have reported a novel complexation strategy for efficient access to planar-chiral-only cyclopentadienyl Rh(I) and Ir(I) complexes, avoiding tedious chiral chromatographic separation of the complexes or resolution with chiral auxiliaries. A structurally diverse Cp ligand library (including a unique acyloxy substitution pattern) was assembled, characterized, and evaluated in benchmark transformations. The most salient achievements on different axes are

- A first-of-its-kind, straightforward method for the fully enantiospecific complexation of Rh and Ir precursors with chiral cyclopentadienes. This features a point-to-planar chirality transfer via facially selective concerted-metalation-deprotonation between a metal-carboxylate [M(olefin)_2_OAc]_2_ and chiral cyclopentadiene, circumventing the typical stereoablative complexation of an achiral cyclopentadienyl anion.
- An unprecedented point-to-axial-to-point-to-planar chirality transfer demonstrated by a highly efficient one-pot synthesis of enantiopure Cp^X^-Rh complexes from a chiral propargylic ester precursor, telescoping an Au-catalyzed cyclization and subsequent Rh(I) complexation.
- The application of sophisticated computational selectivity predictions, delivering ligand design blueprints that could subsequently be tuned in practice for their synthesizability and stability.
- Optimized planar chiral Cp^X^-Rh complexes that display best-in-class enantioselectivities for benchmark asymmetric C–H functionalizations of aryl hydroxamates with terminal aliphatic alkenes.

These accomplishments can ultimately be used to access further novel families of planar chiral group 9 metal complexes and to address yet unsolved challenges in enantioselective catalysis.

## Supporting Information Available

Experimental procedures, characterization data, computational details, spectroscopic data, crystallographic data, and steric maps (PDF)
Cartesian coordinates and energy values of optimized transition state conformers (ZIP)

## References

[ref1] Mas-Roselló J.; Herraiz A.; Audic B.; Laverny A.; Cramer N. Chiral cyclopentadienyl ligands: design, syntheses and applications in asymmetric catalysis. Angew. Chem., Int. Ed. 2021, 60, 13198–13244. 10.1002/anie.202008166. 32672405

[ref2] Newton C.; Kossler D.; Cramer N. Asymmetric catalysis powered by chiral cyclopentadienyl ligands. J. Am. Chem. Soc. 2016, 138, 3935–3941. 10.1021/jacs.5b12964. 26863546

[ref3] Scholz H. J.; Werner H.; Basische metalle L. V. I. Synthese von Li_2_[(C_5_Me_4_)_2_CH_2_] und ringverbrückter rhodium-zweikernkomplexe mit dem (C_5_Me_4_)_2_CH_2_-dianion als brückenliganden. J. Organomet. Chem. 1986, 303, C8–C12. 10.1002/ange.19830950426.

[ref4] Enders M.; Kohl G.; Pritzkow H. Rhodium-Carbonyl Complexes with a Quinolyl Functionalized Cp-Ligand: Synthesis and Photochemical Activation. J. Organomet. Chem. 2004, 689, 3024–3030. 10.1016/j.jorganchem.2004.06.046.

[ref5] Ye B.; Cramer N. Chiral cyclopentadienyl ligands as stereocontrolling element in asymmetric C-H functionalization. Science 2012, 338, 504–506. 10.1126/science.1226938. 23112328

[ref6] Ye B.; Cramer N. A Tunable Class of Chiral Cp Ligands for Enantioselective Rhodium(III)-Catalyzed C–H Allylations of Benzamides. J. Am. Chem. Soc. 2013, 135, 636–639. 10.1021/ja311956k. 23286204

[ref7] Wang S.; Park S. H.; Cramer N. A Readily Accessible Class of Chiral Cp Ligands and Their Application in Ru^II^-Catalyzed Enantioselective Syntheses of Dihydrobenzoindoles. Angew. Chem., Int. Ed. 2018, 57, 5459–5462. 10.1002/anie.201802244. 29528173

[ref8] Li G.; Yan X.; Jiang J.; Liang H.; Zhou C.; Wang J. Chiral Bicyclo[2.2.2]octane-Fused CpRh Complexes: Synthesis and Potential Use in Asymmetric C–H Activation. Angew. Chem., Int. Ed. 2020, 59, 22436–22440. 10.1002/anie.202010489. 32840946

[ref9] Liang H.; Vasamsetty L.; Li T.; Jiang J.; Pang X.; Wang J. A New Class of *C*_2_-Symmetric Chiral Cyclopentadienyl Ligand Derived from Ferrocene Scaffold: Design, Synthesis and Application. Chem.—Eur. J. 2020, 26, 14546–14550. 10.1002/chem.202001814. 32470226

[ref10] Zheng J.; Cui W.-J.; Zheng C.; You S.-L. Synthesis and Application of Chiral Spiro Cp Ligands in Rhodium-Catalyzed Asymmetric Oxidative Coupling of Biaryl Compounds with Alkenes. J. Am. Chem. Soc. 2016, 138, 5242–5245. 10.1021/jacs.6b02302. 27070297

[ref11] Pan C.; Yin S.; Wang S.; Gu Q.; You S. Oxygen-Linked Cyclopentadienyl Rhodium(III) Complexes-Catalyzed Asymmetric C–H Arylation of Benzo[*h*]Quinolines with 1-Diazonaphthoquinones. Angew. Chem., Int. Ed. 2021, 60, 15510–15516. 10.1002/anie.202103638. 33856719

[ref12] Jia Z.-J.; Merten C.; Gontla R.; Daniliuc C.; Antonchick A.; Waldmann H. General enantioselective C-H activation with efficiently tunable cyclopentadienyl ligands. Angew. Chem., Int. Ed. 2017, 56, 2429–2434. 10.1002/anie.201611981. 28124831

[ref13] Kharitonov V. B.; Podyacheva E.; Chusov D.; Nelyubina Y. V.; Muratov D. V.; Loginov D. A. Planar Chiral Rhodium Complex Based on the Tetrahydrofluorenyl Core for Enantioselective Catalysis. Org. Lett. 2023, 25, 8906–8911. 10.1021/acs.orglett.3c03726. 38051945

[ref14] Guo W.; Jiang J.; Wang J. [2.2]Benzoindenophane-Based Chiral Indenyl Ligands: Design, Synthesis, and Applications in Asymmetric C–H Activation. Angew. Chem., Int. Ed. 2024, 63, e202400279 10.1002/anie.202400279. 38781117

[ref15] Yan X.; Jiang J.; Wang J. A class of readily tunable planar-chiral cyclopentadienyl rhodium(III) catalysts for asymmetric C–H activation. Angew. Chem., Int. Ed. 2022, 61, e202201522 10.1002/anie.202201522. 35302699

[ref16] Farr C.; Kazerouni A.; Park B.; Poff C.; Won J.; Sharp K.; Baik M.-H.; Blakey S. Designing a planar chiral rhodium indenyl catalyst for region- and enantioselective allylic C–H amidation. J. Am. Chem. Soc. 2020, 32, 13996–14004. 10.1021/jacs.0c07305. 32667782

[ref17] Gross P.; Im H.; Laws D. III; Park B.; Baik M.-H.; Blakey S. B. Enantioselective Aziridination of Unactivated Terminal Alkenes Using a Planar Chiral Rh(III) Indenyl Catalyst. J. Am. Chem. Soc. 2024, 146, 1447–1454. 10.1021/jacs.3c10637. 38170978 PMC10797617

[ref18] Trifonova E.; Ankudinov N.; Mikhaylov A.; Chusov D.; Nelyubina Y.; Perekalin D. A planar-chiral rhodium(III) catalyst with a sterically demanding cyclopentadienyl ligand and its application in the enantioselective synthesis of dihydroisoquinolones. Angew. Chem., Int. Ed. 2018, 57, 7714–7718. 10.1002/anie.201801703. 29624840

[ref19] Kolos A.; Nelyubina Y.; Sundararaju B.; Perekalin D. Synthesis of overloaded cyclopentadienyl rhodium(III) complexes via cyclotetramerization of *tert-*butylacetylene. Organometallics 2021, 40, 3712–3719. 10.1021/acs.organomet.1c00403.

[ref20] Zhang C.; Jiang J.; Huang X.; Wang J. Planar-Chiral Cyclopentadienyl Rhodium Catalysts: Design Concept, Chiral Resolution Strategy, and Applications. ACS Catal. 2023, 13, 10468–10473. 10.1021/acscatal.3c02865.

[ref21] Gandon V.; Hoarau C. Concerted vs Nonconcerted Metalation–Deprotonation in Orthogonal Direct C–H Arylation of Heterocycles with Halides: A Computational Study. J. Org. Chem. 2021, 86, 1769–1778. 10.1021/acs.joc.0c02604. 33406843

[ref22] Audic B.; Wodrich M. D.; Cramer N. Mild complexation protocol for chiral Cp^X^ Rh and Ir complexes suitable for *in situ* catalysis. Chem. Sci. 2019, 10, 781–787. 10.1039/c8sc04385j. 30774871 PMC6346397

[ref23] Pototskiy R. A.; Kolos A. V.; Nelyubina Y. V.; Perekalin D. S. Rhodium Catalysts with a Chiral Cyclopentadienyl Ligand Derived from Natural R-Myrtenal. Eur. J. Org. Chem. 2020, 2020, 6019–6025. 10.1002/ejoc.202001029.

[ref24] Brauns M.; Cramer N. Efficient kinetic resolution of sulfur-stereogenic sulfoximes exploiting Cp^X^Rh^III^-catalyzed C-H functionalization. Angew. Chem., Int. Ed. 2019, 58, 8902–8906. 10.1002/anie.201904543. 31045299

[ref25] Amouri H.; Gruselle M.; Jaouen G. bis[Dichloro(η-pentamethylcyclopentadienyl)rhodium(III) and – iridium(III)] from bis[Chloro(1, 5-cyclooctadiene)rhodium(I) and – iridium(I)] Oxidation, and Formation of 1, 5-Cyclooctadiene(η-pentamethylcyclopentadienyl)rhodium(I). Synth. React. Inorg. Met.Org. Chem. 1994, 24, 395–400. 10.1016/S0022-328X(98)00939-5.

[ref26] Wozniak Ł.; Cramer N. Atropo-enantioselective oxidation-enabled iridium(III)-catalyzed C–H arylations with boronic esters. Angew. Chem., Int. Ed. 2021, 60, 18532–18536. 10.1002/anie.202106403. PMC8457206 34153163

[ref27] Frantz D.; Fässler R.; Carreira E. Facile enantioselective synthesis of propargylic alcohols by direct addition of terminal alkynes to aldehydes. J. Am. Chem. Soc. 2000, 122, 1806–1807. 10.1021/ja993838z.

[ref28] Zhao K.; Hsu Y.-C.; Yang Z.; Liu R.-S.; Zhang L. Gold-catalyzed synthesis of chiral cyclopentadienyl esters via chirality transfer. Org. Lett. 2020, 22, 6500–6504. 10.1021/acs.orglett.0c02293. 32806155 PMC8623356

[ref29] Fast C. D.; Schley N. D. Light-Promoted Transfer of an Iridium Hydride in Alkyl Ether Cleavage. Organometallics 2021, 40, 3291–3297. 10.1021/acs.organomet.1c00391.

[ref30] Trifonova E. A.; Ankudinov N. M.; Kozlov M. V.; Sharipov M. Y.; Nelyubina Y. V.; Perekalin D. S. Rhodium(III) Complex with a Bulky Cyclopentadienyl Ligand as a Catalyst for Regioselective Synthesis of Dihydroisoquinolones through C–H Activation of Arylhydroxamic Acids. Chem.—Eur. J. 2018, 24, 16570–16575. 10.1002/chem.201804050. 30209829

[ref31] Lin W.; Li W.; Lu D.; Su F.; Wen T.-B.; Zhang H.-J. Dual Effects of Cyclopentadienyl Ligands on Rh(III)-Catalyzed Dehydrogenative Arylation of Electron-Rich Alkenes. ACS Catal. 2018, 8, 8070–8076. 10.1021/acscatal.8b01753.

[ref32] Tellers D. M.; Yung C. M.; Arndtsen B. A.; Adamson D. R.; Bergman R. G. Electronic and Medium Effects on the Rate of Arene C–H Bond Activation by Cationic Ir(III) Complexes. J. Am. Chem. Soc. 2002, 124, 1400–1410. 10.1021/ja011809u. 11841308

[ref33] Hyster T. K.; Knörr L.; Ward T. R.; Rovis T. Biotinylated Rh(III) Complexes in Engineered Streptavidin for Accelerated Asymmetric C–H Activation. Science 2012, 338, 500–503. 10.1126/science.1226132. 23112327 PMC3820005

[ref34] Cui W.-J.; Wu Z.-J.; Gu Q.; You S.-L. Divergent Synthesis of Tunable Cyclopentadienyl Ligands and Their Application in Rh-Catalyzed Enantioselective Synthesis of Isoindolinone. J. Am. Chem. Soc. 2020, 142, 7379–7385. 10.1021/jacs.0c02813. 32259425

[ref35] Ozols K.; Jang Y.-S.; Cramer N. Chiral Cyclopentadienyl Cobalt(III) Complexes Enable Highly Enantioselective 3d-Metal-Catalyzed C–H Functionalizations. J. Am. Chem. Soc. 2019, 141, 5675–5680. 10.1021/jacs.9b02569. 30901216

[ref36] Laplaza R.; Sobez J.-G.; Wodrich M. D.; Reiher M.; Corminboeuf C. The (Not So) Simple Prediction of Enantioselectivity – A Pipeline for High-Fidelity Computations. Chem. Sci. 2022, 13, 6858–6864. 10.1039/D2SC01714H. 35774159 PMC9200111

[ref37] Wodrich M. D.; Laplaza R.; Cramer N.; Reiher M.; Corminboeuf C. Toward in silico Catalyst Optimization. Chimia 2023, 77, 139–143. 10.2533/chimia.2023.139. 38047817

[ref38] Sobez J.-G.; Reiher M. MOLASSEMBLER: Molecular Graph Construction, Modification, and Conformer Generation for Inorganic and Organic Molecules. J. Chem. Inf. Model. 2020, 60, 3884–3900. 10.1021/acs.jcim.0c00503. 32610018 PMC12344703

[ref39] Bensberg M.; Grimmel S.; Sobez J.-G.; Steiner M.; Unsleber J. P.; Rieher M. qcscine/Molassembler: Release 3.0.0, 2024, https://zenodo.org/records/13372940 (accessed September 22, 2024).

[ref40] Stewart J. J. P. Optimization of Parameters for Semiempirical Methods VI: More Modifications to the NDDO Approximations and Re-optimization of Parameters. J. Mol. Model. 2013, 19, 1–32. 10.1007/s00894-012-1667-x. 23187683 PMC3536963

[ref41] Perdew J. P.; Burke K.; Ernzerhof M. Generalized Gradient Approximation Made Simple. Phys. Rev. Lett. 1996, 77, 3865–3868. 10.1103/PhysRevLett.77.3865. 10062328

[ref42] Adamo C.; Barone V. Toward Reliable Density Functional Methods without Adjustable Parameters: The PBE0Model. J. Chem. Phys. 1999, 110, 6158–6170. 10.1063/1.478522.

[ref43] Grimme S.; Antony J.; Ehrlich S.; Krieg H. A consistent and accurate *ab initio* parametrization of density functional dispersion correction (DFT-D) for the 94 elements H-Pu. J. Chem. Phys. 2010, 132, 154104 10.1063/1.3382344. 20423165

[ref44] Grimme S.; Ehrlich S.; Goerigk L. Effect of the Damping Function in Dispersion Corrected Density Functional Theory. J. Comput. Chem. 2011, 32, 1456–1465. 10.1002/jcc.21759. 21370243

[ref45] Weigend F.; Ahlrichs R. Balanced Basis Sets of Split Valence, Triple Zeta Valence and Quadruple Zeta Valence Quality for H to Rn: Design and Assessment of Accuracy. Phys. Chem. Chem. Phys. 2005, 7, 3297–3305. 10.1039/b508541a. 16240044

[ref46] Laplaza R.; Wodrich M. D.; Corminboeuf C. Overcoming the Pitfalls of Computing Reaction Selectivity from Ensembles of Transition States. J. Phys. Chem. Lett. 2024, 15, 7363–7370. 10.1021/acs.jpclett.4c01657. 38990895 PMC11284845

[ref47] Falivene L.; Cao Z.; Petta A.; Serra L.; Poater A.; Oliva R.; Scarano V.; Cavallo L. Towards the Online Computer-Aided Design of Catalytic Pockets. Nat. Chem. 2019, 11, 872–879. 10.1038/s41557-019-0319-5. 31477851

