# Supplementary material for: Enantiospecific Synthesis of Planar Chiral Rhodium and Iridium Cyclopentadienyl Complexes: Enabling Streamlined and Computer-Guided Access to Highly Selective Catalysts for Asymmetric C–H Functionalizations
Source: J Am Chem Soc. 2024 Dec 6;146(50):34786–95. doi: 10.1021/jacs.4c13279 (PMC11664503; doi:10.1021/jacs.4c13279)
Supplement: Supplementary file 1 — ja4c13279_si_001.pdf [file ja4c13279_si_001.pdf]

Supporting Information

Enantiospecific Synthesis of Planar Chiral Rhodium and Iridium Cyclopentadienyl Complexes: Enabling Streamlined and Computer-Guided Access to Highly Selective Catalysts for Asymmetric C-H Functionalizations

*Young Sebastian Ye,[a]‡ Aragorn Laverny,[a]‡ Matthew D. Wodrich,[b] Ruben Laplaza,[b] Farzaneh Fadaei-Tirani,[c] Rosario Scopelliti,[c] Clemence Corminboeuf,[b] and Nicolai Cramer[a]**

[a] *Laboratory of Asymmetric Catalysis and Synthesis, Institute of Chemical Sciences and Engineering, Ecole Polytechnique Fédérale de Lausanne (EPFL), 1015 Lausanne, Switzerland.*

[b] *Laboratory for Computational Molecular Design, Institute of Chemical Sciences and Engineering, Ecole Polytechnique Fédérale de Lausanne (EPFL), 1015 Lausanne, Switzerland.*

[c] *X-Ray Diffraction and Surface Analytics Facility, Institute of Chemical Sciences and Engineering, Ecole Polytechnique Fédérale de Lausanne (EPFL), 1015 Lausanne, Switzerland.*

** Email: nicolai.cramer@epfl.ch*

Homepage: https://lcsa.epfl.ch/

Table of Contents

1. General Considerations 3

2. Synthesis and Characterisation of Chiral Cyclopentadienes 5

3. Synthesis and Characterization of Rh(I) and Ir(I) Cp Complexes 71

4. Synthesis and Characterization of Rh(III) and Ir(III) Cp Complexes 108

5. Rh-Catalyzed Asymmetric C-H Functionalization Reactions 125

6. Computational Details 141

7. NMR Spectra 147

8. X-ray Crystallography Data 247

9. Steric Maps 270

10. References 280

1. General Considerations

All reactions were carried out under an atmosphere of nitrogen in flame-dried glassware with magnetic stirring inside a glove box or using Schlenk technique, unless otherwise indicated. Dichloromethane, *n*-pentane and diethyl ether were purified by an Innovative Technology Solvent Delivery System degassed *via* freeze-pump-thaw technique and stored over 4 Å molecular sieves in a nitrogen-filled glove box. Chemicals were used as obtained from the suppliers unless otherwise indicated. Flash chromatography was performed with Silicycle silica gel 60 (40-63 µm grade). Analytical thin-layer chromatography was performed with commercial glass plates coated with 0.25 mm silica gel (E. Merck, Kieselgel 60 F_{254}). Compounds were either visualized under UV-light at 254 nm or by dipping the plates in an aqueous potassium permanganate solution followed by heating. Proton nuclear magnetic resonance (^{1}H NMR) data was acquired on a Bruker AVANCE400 (400 MHz), Bruker DRX-400 (400 MHz) or Bruker AVANCEIII-400 (400 MHz) spectrometer at 298 K unless otherwise noted. Chemical shifts (δ) are reported in parts per million (ppm) relative to incompletely deuterated $CDCl_3$ (s, 7.26 ppm), CD_2Cl_2 (s, 5.32 ppm), or C_6D_6 (s, 7.16 ppm). Splitting patterns are designated as s, singlet; d, doublet; t, triplet; q, quartet; hept, heptet; m, multiplet, br, broad. Proton-decoupled Carbon-13 nuclear magnetic resonance (^{13}C{^{1}H} NMR) data were acquired on a Bruker *AVANCE400* (101 MHz), Bruker *DRX-400* (101 MHz) or Bruker *AVANCEIII-400* (101 MHz). The assignment of primary (CH_3), secondary (CH_2), tertiary (CH), and quaternary carbon nuclei (C_{quat}) was made using DEPT-135 spectra. Chemical shifts are reported in ppm relative to $CDCl_3$ (77.16 ppm), CD_2Cl_2 (53.84 ppm) or C_6D_6 (128.06 ppm). Infrared (IR) data were recorded on an Alpha-P Bruker FT-IR Spectrometer. Absorbance frequencies are reported in reciprocal centimeters (cm^{-1}). Melting points were measured on a Büchi B-540 and are uncorrected. HRMS measurements were performed by an Agilent LC-MS TOF or Orbitrap. High resolution mass values are given in *m/z*. Optical rotations were measured on a Polartronic M polarimeter using a 10.0 cm cell with a Na 589 nm filter. Enantiomeric excesses were measured on an Agilent HPLC system, a Shimadzu HPLC system or an Agilent SFC Investigator system using CHIRALPAK® IA-IG chiral stationary phase columns. Enantiomeric excess was also measured on an Agilent 7890A GC Chromatograph using a CHIRALDEX® G-TA chiral stationary phase column. X-ray analysis was performed by Dr. F. Tirani and Dr. R. Scopelliti at the EPF Lausanne. Elemental analyses were performed by Roxane Moinat at the elemental analysis

platform of ISIC at the EPF Lausanne. Buried volumes and steric maps were generated with SambVca 2.1 (bond radii scaled by 1.17, sphere radius 3.5, mesh spacing 0.10, H atoms included).[1]

2. Synthesis and Characterization of Chiral Cyclopentadienes

Note: cyclohex-1-en-1-yl trifluoromethanesulfonate and (*S*)-3-butyn-2-ol were purchased from chemical vendors.

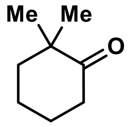

P1

2,2-dimethylcyclohexanone (**P1**) was prepared *via* a procedure by Baran[2]. The following characterization data were consistent with literature.[3]

^{1}H NMR (400 MHz, $CDCl_3$) δ 2.41 – 2.37 (m, 2H), 1.86 – 1.79 (m, 2H), 1.76 – 1.70 (m, 2H), 1.68 – 1.63 (m, 2H), 1.11 (s, 6H).

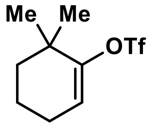

P2

6,6-dimethylcyclohex-1-en-1-yl trifluoromethanesulfonate (**P2**) was prepared *via* a procedure by Nair[4] with **P1**. The following characterization data were consistent with literature.[5]

^{1}H NMR (400 MHz, $CDCl_3$) δ 5.66 (t, *J* = 4.1 Hz, 1H), 2.20 – 2.13 (m, 2H), 1.64 (d, *J* = 2.9 Hz, 4H), 1.14 (s, 6H).

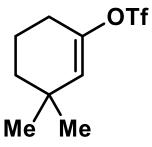

P3

3,3-dimethylcyclohex-1-en-1-yl trifluoromethanesulfonate (**P3**) was prepared *via* a procedure by Ishihara[6]. The following characterization data were consistent with literature.[6]

^{1}H NMR (400 MHz, $CDCl_3$) δ 5.51 (s, 1H), 2.27 (td, *J* = 6.3, 1.6 Hz, 2H), 1.84 – 1.74 (m, 2H), 1.46 – 1.40 (m, 2H), 1.06 (s, 6H).

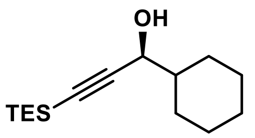

P4

(S)-1-cyclohexyl-3-(triethylsilyl)prop-2-yn-1-ol (**P4**) was prepared *via* a procedure by Carreira[7] using (−)-*N*-methylephedrine instead of (+)-*N*-methylephedrine. The following characterization data were consistent with literature.[7]

^{1}H NMR (400 MHz, $CDCl_3$) δ 4.16 (d, J = 5.9 Hz, 1H), 1.85 (d, J = 12.0 Hz, 2H), 1.81 – 1.73 (m, 2H), 1.73 – 1.64 (m, 2H), 1.60 – 1.51 (m, 1H), 1.31 – 1.04 (m, 5H), 0.99 (t, J = 7.9 Hz, 9H), 0.60 (q, J = 7.9 Hz, 6H).

Opt. Rot. $[\alpha]_D^{20}$ +4.90 (c = 0.68 in $CHCl_3$, 93% ee as determined in compound **P5**). Literature for opposite enantiomer: $[\alpha]_D^{27}$ −5.4 (c = 1.1 in $CHCl_3$, 96% ee).

Synthesis of (*S*)-1-cyclohexylprop-2-yn-1-ol (P5) adapted from a procedure by Molinski[8]

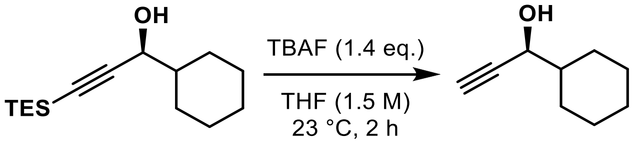

A solution of **P4** (2.18 g, 8.64 mmol) in dry THF (5.76 mL) was treated dropwise over 5 min with TBAF (12.2 mL, 1 M in THF, 12.2 mmol). The reaction mixture was stirred at 23 °C for 2 h, then cooled to 0 °C and quenched by dropwise addition of NH_4Cl solution (10 mL, sat. aq.). The mixture was extracted with Et_2O (3 × 5 mL), and the combined organic layers dried with Na_2SO_4, filtered, and concentrated *in vacuo*. The resulting crude residue was subjected to column chromatography (silica gel, 10:1 pentane / EtOAc) to afford **P5** as a pale yellow solid (0.959 g, 80% yield, 93% ee). The following characterization data were consistent with literature.[9]

^{1}H NMR (400 MHz, $CDCl_3$) δ 4.16 (td, J = 5.9, 2.2 Hz, 1H), 2.46 (d, J = 2.2 Hz, 1H), 1.90 – 1.82 (m, 2H), 1.82 – 1.75 (m, 2H), 1.72 (s, 2H), 1.61 – 1.54 (m, 1H), 1.34 – 1.01 (m, 5H).

GC CHIRALDEX® G-TA, inlet 250 °C, FID detector 250 °C, oven initial 80°C (2 min), rate 1 °C / min, oven final 130 °C (5 min), t_R major 27.54 min; t_R minor 27.97 min.

Opt. Rot. $[\alpha]_D^{20}$ −10.67 (c = 0.25 in Et_2O, 93% ee). Literature for opposite enantiomer: $[\alpha]_D^{25}$

+10 (c = 0.500 in Et_2O, >99% ee).

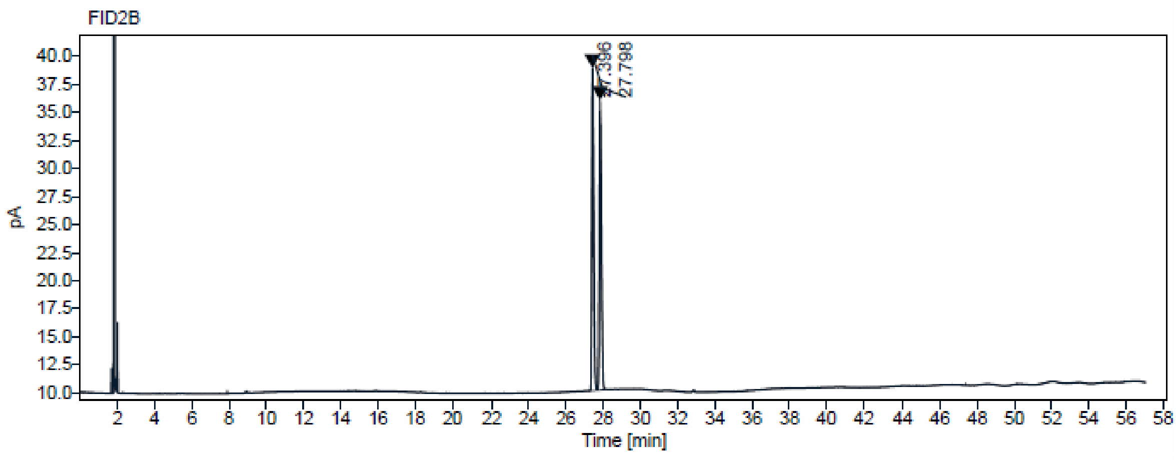

Signal: FID2B

RT [min]	Type	Width [min]	Area	Height	Area%	Name
27.396	BV	0.3822	189.8253	28.7410	49.9656	
27.798	VV	0.3817	190.0870	25.8218	50.0344	
		Sum	379.9123			

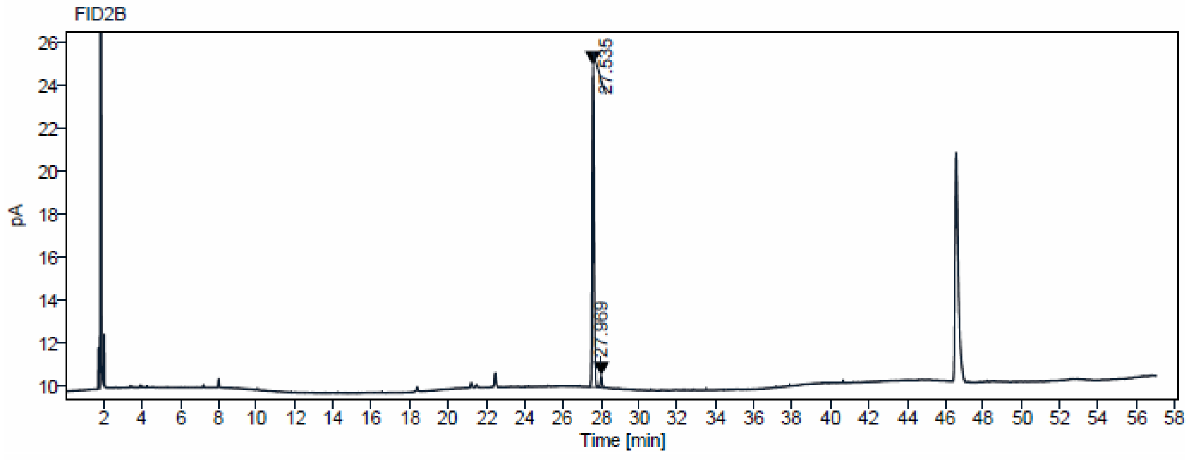

Signal: FID2B

RT [min]	Type	Width [min]	Area	Height	Area%	Name
27.535	BB	0.3797	95.9378	14.9533	96.3222	
27.969	BB	0.2869	3.6631	0.5468	3.6778	
		Sum	99.6009			

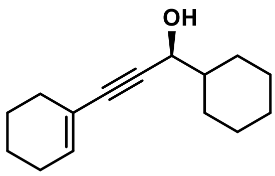

6a

(*S*)-3-(cyclohex-1-en-1-yl)-1-cyclohexylprop-2-yn-1-ol (**6a**) was prepared *via* a procedure by Carreira[10] using (−)-*N*-methylephedrine instead of (+)-*N*-methylephedrine. The following characterization data were consistent with literature.[11]

^{1}H NMR (400 MHz, $CDCl_3$) δ 6.10 (tt, *J* = 3.8, 1.7 Hz, 1H), 4.25 (s, 1H), 2.14 – 2.05 (m, 4H), 1.90 – 1.81 (m, 2H), 1.81 – 1.73 (m, 2H), 1.71 – 1.54 (m, 7H), 1.29 – 1.07 (m, 5H).

^{13}C NMR (101 MHz, $CDCl_3$) δ 135.2, 120.4, 87.7, 86.6, 67.8, 44.5, 29.4, 28.8, 28.3, 26.6, 26.08, 26.06, 25.7, 22.4, 21.6.

IR (ATR) 3352, 2921, 2851, 1448, 1435, 1379, 1335, 1269, 1204, 1081, 1018, 983, 892, 841, 799.

HRMS (ESI / QTOF) m/z: $[M + Ag]^+$ calculated for $[C_{15}H_{22}AgO]^+$: 325.0716; found: 325.0709.

Rf 0.35 (silica gel, 10:1 pentane / EtOAc, UV / CAM).

HPLC CHIRALPAK® IC, 99:1 hexane / IPA, rate 1 mL / min, 25 min, 254 nm, t_R major 17.94 min; t_R minor 20.19 min.

Opt. Rot. $[\alpha]_D^{21}$ +10.00 (c = 1.0 in $CHCl_3$, 97% ee). Literature for opposite enantiomer: $[\alpha]_D^{21}$ −6.6 (c = 1.00 in $CHCl_3$, 85% ee).

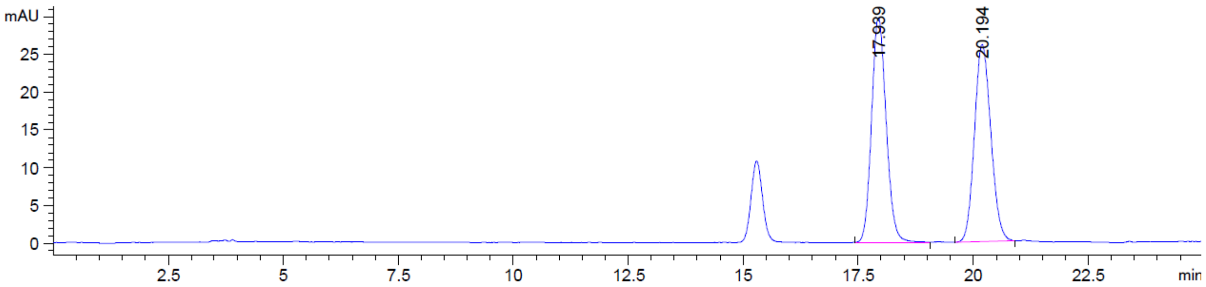

Peak #	RetTime [min]	Type	Width [min]	Area [mAU*s]	Height [mAU]	Area %
1	17.939	BB	0.3376	656.52905	29.73965	50.3490
2	20.194	BB	0.3872	647.42682	26.11732	49.6510

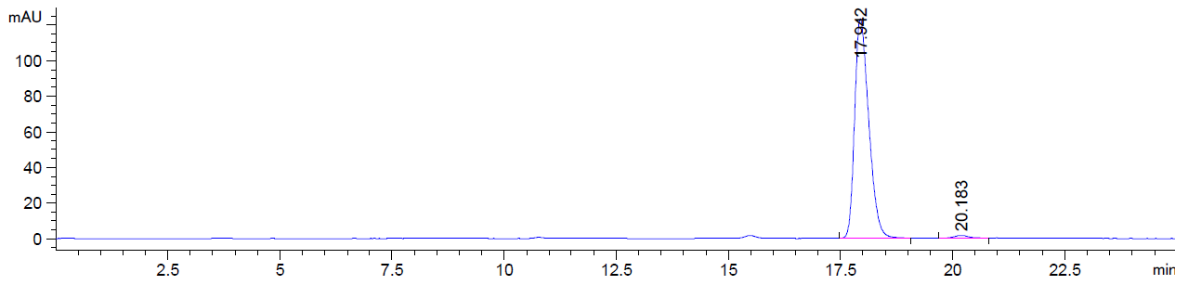

Peak #	RetTime [min]	Type	Width [min]	Area [mAU*s]	Height [mAU]	Area %
1	17.942	BB	0.3372	2697.43799	123.33196	98.7237
2	20.183	BB	0.3121	34.87358	1.52887	1.2763

General procedure A for the Sonogashira coupling of cyclohexenyl triflates with propargylic alcohols

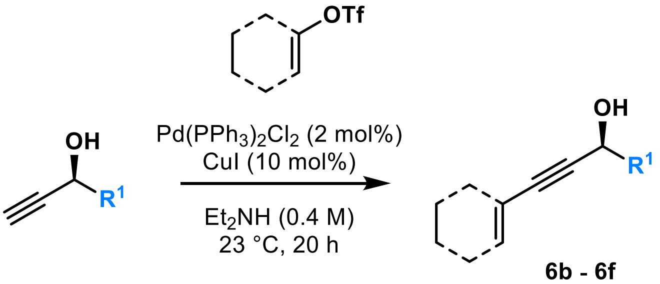

6b - 6f

A solution of alkynol (1 eq.) in Et_2NH (1.3 M relative to alkynol) and a suspension of cyclohexenyl triflate (1.0 - 1.4 eq), $Pd(PPh_3)_2Cl_2$ (2 mol%) and CuI (10 mol%) in Et_2NH (0.64 M relative to alkynol) were prepared separately and both deoxygenated under N_2 bubbling for 10 min. Then, the triflate suspension was treated with the alkynol solution and stirred at 23 °C for 20 h. The reaction mixture was quenched at 0 °C with NH_4Cl solution (equivolume to Et_2NH, sat. aq.) and extracted with EtOAc (3 × equivolume to Et_2NH). The combined organic layers were dried (Na_2SO_4), filtered and concentrated *in vacuo*. The crude residue was subjected to column chromatography (silica gel) to afford adduct **6**.

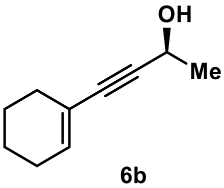

6b

Propargylic alcohol **6b** was prepared via general procedure **A** with (*S*)-but-3-yn-2-ol (0.25 g, 3.6 mmol), cyclohex-1-en-1-yl trifluoromethanesulfonate (0.82 g, 3.6 mmol), $Pd(PPh_3)_2Cl_2$ (50 mg, 71 µmol), CuI (68 mg, 0.36 mmol) and 10:1 pentane / EtOAc eluent, to give a brown oil

(0.50 g, 93% yield, 96% ee). (see page **16** for characterization data).

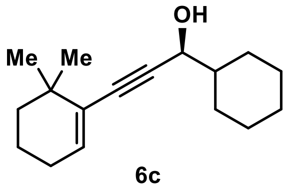

6c

Propargylic alcohol **6c** was prepared via general procedure **A** with **P5** (500 mg, 3.62 mmol), **P2** (1.31 g, 5.07 mmol), $Pd(PPh_3)_2Cl_2$ (50.7 mg, 72.4 µmol), CuI (68.9 mg, 0.362 mmol) and 10:1 pentane / EtOAc eluent, to give a brown oil (780 mg, 88% yield, 92% ee).

^{1}H NMR (400 MHz, $CDCl_3$) δ 6.03 (t, J = 4.1 Hz, 1H), 4.29 (t, J = 5.8 Hz, 1H), 2.08 – 2.01 (m, 2H), 1.86 (d, J = 11.3 Hz, 2H), 1.78 (d, J = 12.3 Hz, 2H), 1.68 (d, J = 5.9 Hz, 2H), 1.64 – 1.56 (m, 3H), 1.52 – 1.47 (m, 2H), 1.30 – 1.07 (m, 11H).

^{13}C NMR (101 MHz, $CDCl_3$) δ 134.7, 129.9, 88.1, 85.7, 77.2, 76.8, 67.9, 44.6, 37.7, 33.8, 28.9, 28.8, 28.2, 26.6, 26.3, 26.1, 26.1, 18.9.

IR (ATR) 3356, 2924, 2852, 1451, 1383, 1360, 1336, 1189, 1081, 1017, 981, 892, 814.

HRMS (ESI/QTOF) m/z: $[M - OH]^+$ calculated for $[C_{17}H_{25}]^+$: 229.1951; found: 229.1947.

R*f* 0.32 (silica gel, 5:1 pentane / EtOAc, UV / PMA).

HPLC CHIRALPAK® IC, 99:1 hexane / IPA, rate 1 mL / min, 25 min, 230 nm, t_R major 12.69 min; t_R minor 13.51 min.

Opt. Rot. $[\alpha]_D^{20}$ +11.44 (c = 0.51 in $CHCl_3$, 92% ee).

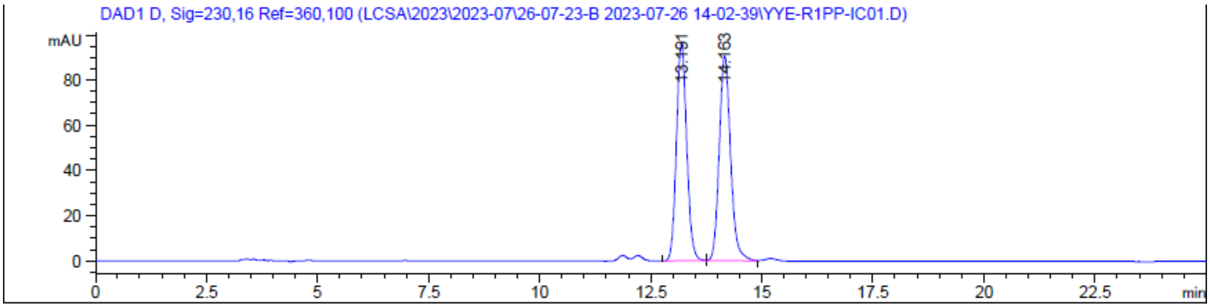

Signal 3: DAD1 D, Sig=230,16 Ref=360,100

Peak #	RetTime [min]	Type	Width [min]	Area [mAU*s]	Height [mAU]	Area %
1	13.191	BB	0.2492	1545.21265	96.30853	49.0655
2	14.163	BB	0.2749	1604.07385	90.47083	50.9345

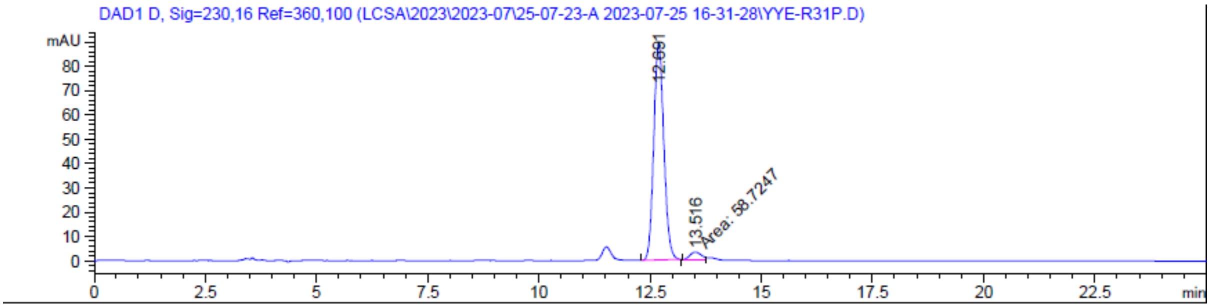

Signal 3: DAD1 D, Sig=230,16 Ref=360,100

Peak #	RetTime [min]	Type	Width [min]	Area [mAU*s]	Height [mAU]	Area %
1	12.691	BB	0.2441	1408.93774	89.27266	95.9988
2	13.516	MM T	0.3078	58.72466	3.17958	4.0012

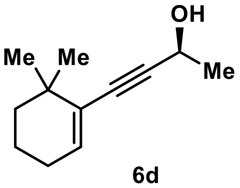

6d

Propargylic alcohol **6d** was prepared via general procedure **A** with (*S*)-but-3-yn-2-ol (0.50 g, 7.1 mmol), **P2** (1.8 g, 7.1 mmol), $Pd(PPh_3)_2Cl_2$ (0.10 g, 0.14 mmol), CuI (0.14 g, 0.71 mmol) and 10:1 pentane / EtOAc eluent, to give a yellow oil (1.1 g, 89% yield, 98% ee).

^{1}H NMR (400 MHz, $CDCl_3$) δ 6.02 (t, *J* = 4.1 Hz, 1H), 4.73 – 4.60 (m, 1H), 2.08 – 2.00 (m, 2H), 1.80 – 1.74 (m, 1H), 1.66 – 1.55 (m, 2H), 1.51 – 1.46 (m, 5H), 1.09 (s, 6H).

^{13}C NMR (101 MHz, $CDCl_3$) δ 134.8, 129.7, 90.0, 84.0, 59.1, 37.7, 33.7, 28.7, 26.3, 24.8, 18.9.

IR (ATR) 3345, 2963, 2931, 2867, 2832, 1456, 1429, 1384, 1361, 1332, 1188, 1095, 1076, 1026, 998, 933, 910.

HRMS (Sicrit plasma/LTQ-Orbitrap) m/z: $[M - H]^+$ calculated for $[C_{12}H_{17}O]^+$: 177.1274; found: 177.1270.

Rf 0.57 (silica gel, 10:1 pentane / EtOAc, UV / PMA).

HPLC CHIRALPAK® IB, 98:2 hexane / IPA, rate 1 mL / min, 15 min, 254 nm, t_R minor 4.50 min; t_R major 4.92 min.

Opt. Rot. $[\alpha]_D^{20}$ −13.64 (c = 0.33 in $CHCl_3$, 98% ee).

Chromatogram

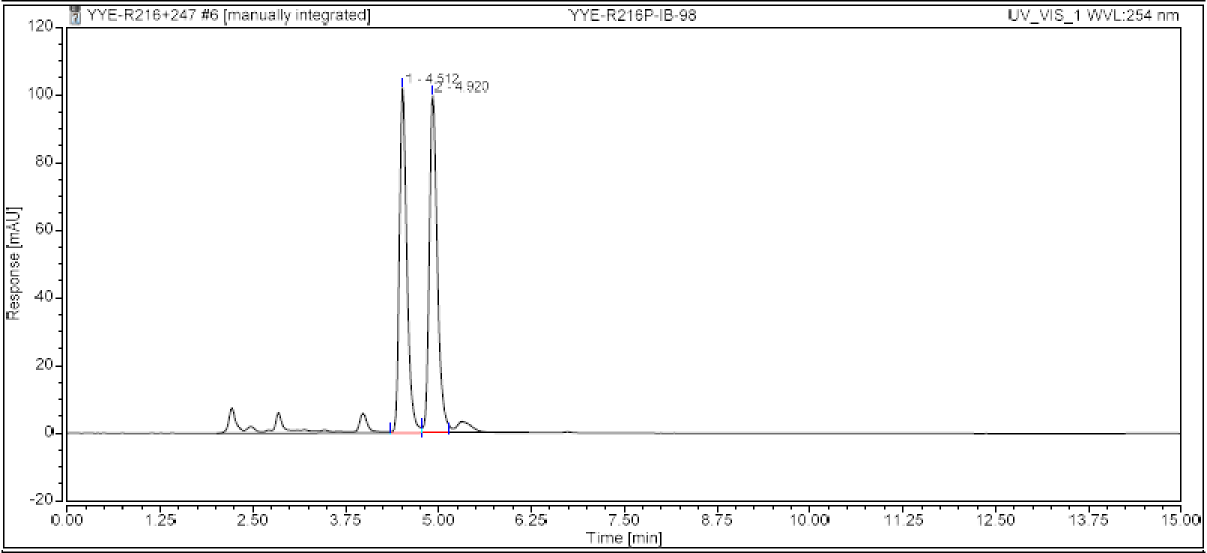

Integration Results

No.	Peak Name	Retention Time<br>min	Area<br>mAU*min	Height<br>mAU	Relative Area<br>%	Relative Height<br>%	Amount<br>n.a.
1		4.512	11.936	101.903	48.96	50.58	n.a.
2		4.920	12.441	99.573	51.04	49.42	n.a.
**Total:**			**24.377**	**201.476**	**100.00**	**100.00**	

Chromatogram

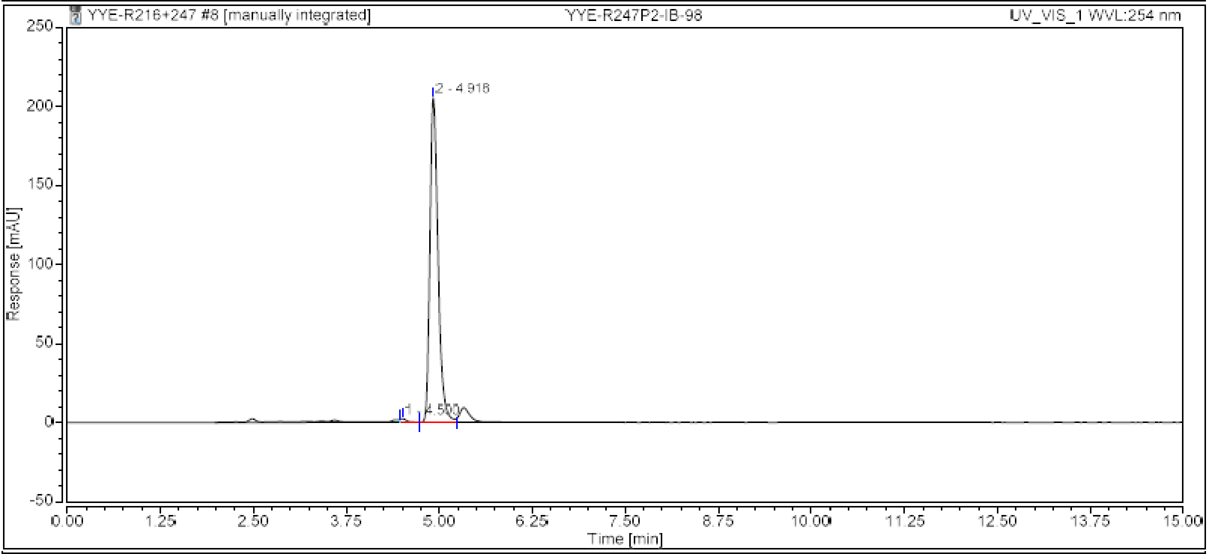

Integration Results

No.	Peak Name	Retention Time<br>min	Area<br>mAU*min	Height<br>mAU	Relative Area<br>%	Relative Height<br>%	Amount<br>n.a.
1		4.500	0.231	2.285	0.88	1.10	n.a.
2		4.918	26.103	205.601	99.12	98.90	n.a.
**Total:**			**26.335**	**207.886**	**100.00**	**100.00**	

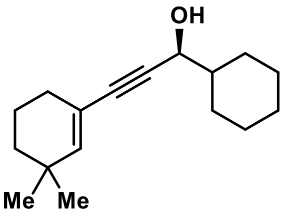

6e

Propargylic alcohol **6e** was prepared via general procedure **A** with **P5** (207 mg, 1.50 mmol), **P3** (504 mg, 1.95 mmol), $Pd(PPh_3)_2Cl_2$ (21.0 mg, 30.0 µmol), CuI (28.5 mg, 0.150 mmol) and 10:1

to 5:1 pentane / EtOAc gradient eluent, to give a brown oil (352 mg, 95% yield, 93% ee).

^{1}H NMR (400 MHz, $CDCl_3$) δ 5.83 (s, 1H), 4.25 (d, *J* = 6.0 Hz, 1H), 2.06 (td, *J* = 6.2, 1.9 Hz, 2H), 1.87 (d, *J* = 10.6 Hz, 2H), 1.77 (d, *J* = 12.6 Hz, 2H), 1.67 – 1.60 (m, 3H), 1.59 – 1.48 (m, 2H), 1.45 – 1.37 (m, 2H), 1.29 – 1.03 (m, 5H), 0.99 (s, 6H).

^{13}C NMR (101 MHz, $CDCl_3$) δ 144.9, 118.2, 87.6, 86.7, 67.8, 44.5, 36.4, 32.5, 29.6, 29.5, 28.7, 28.4, 26.5, 26.1, 19.5.

IR (ATR) 3344, 2924, 2852, 1450, 1382, 1359, 1264, 1204, 1153, 1082, 1018, 992, 937, 892, 872.

HRMS (Sicrit plasma/LTQ-Orbitrap) m/z: $[M + H]^+$ calculated for $[C_{17}H_{27}O]^+$: 247.2056; found: 247.2055.

Rf 0.50 (silica gel, 10:1 pentane / EtOAc, UV / PMA).

HPLC CHIRALPAK® IC, 99:1 hexane / IPA, rate 1 mL / min, 15 min, 230 nm, t_R major 10.77 min; t_R minor 12.73 min.

Opt. Rot. $[\alpha]_D^{20}$ +9.83 (c = 0.39 in $CHCl_3$, 93% ee).

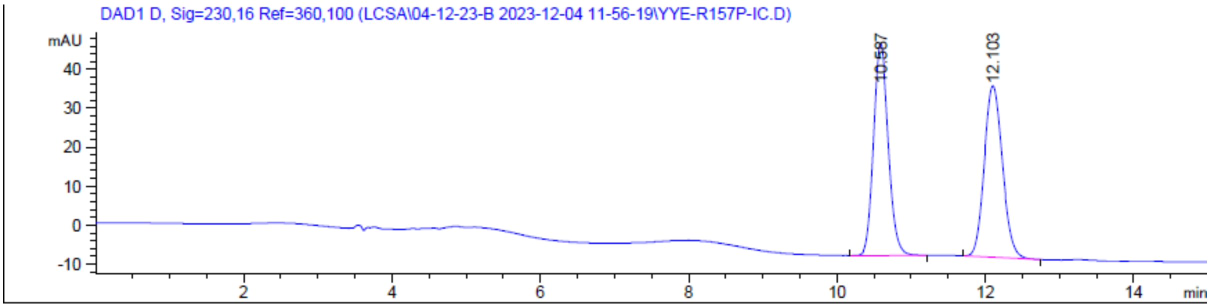

Signal 3: DAD1 D, Sig=230,16 Ref=360,100

Peak #	RetTime [min]	Type	Width [min]	Area [mAU*s]	Height [mAU]	Area %
1	10.587	BB	0.2143	748.91949	54.49914	49.9994
2	12.103	BB	0.2665	748.93732	44.01826	50.0006

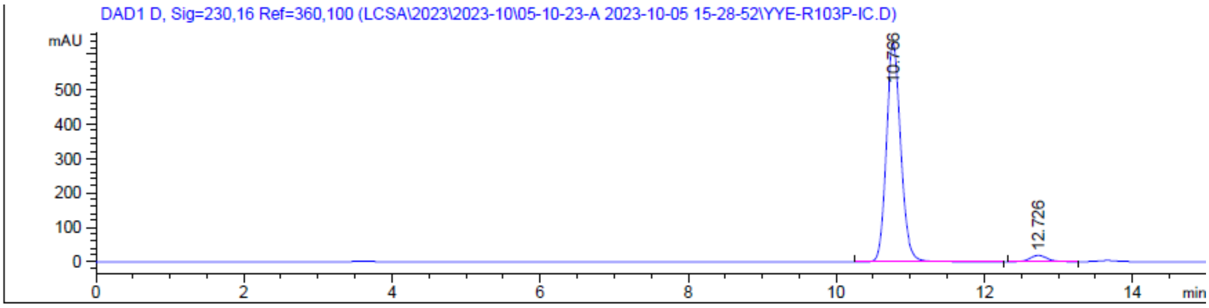

Signal 3: DAD1 D, Sig=230,16 Ref=360,100

Peak #	RetTime [min]	Type	Width [min]	Area [mAU*s]	Height [mAU]	Area %
1	10.766	BB	0.2089	8512.20215	633.03351	96.6881
2	12.726	BB	0.2485	291.57257	18.23574	3.3119

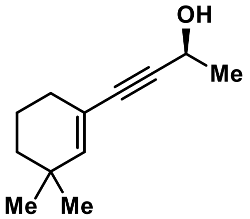

6f

Propargylic alcohol **6f** was prepared via general procedure **A** with (*S*)-but-3-yn-2-ol (25.3 mg, 0.362 mmol), **P3** (121 mg, 0.470 mmol), $Pd(PPh_3)_2Cl_2$ (5.1 mg, 7.2 µmol), CuI (6.9 mg, 36 µmol) and 10:1 to 5:1 pentane / EtOAc gradient eluent, to give a yellow oil (60.5 mg, 94% yield, >99% ee).

^{1}H NMR (400 MHz, $CDCl_3$) δ 5.84 (s, 1H), 4.63 (q, *J* = 6.6 Hz, 1H), 2.05 (td, *J* = 6.2, 1.9 Hz, 2H), 1.79 (s, 1H), 1.67 – 1.59 (m, 2H), 1.46 (d, *J* = 6.6 Hz, 3H), 1.42 – 1.37 (m, 2H), 0.98 (s, 6H).

^{13}C NMR (101 MHz, $CDCl_3$) δ 145.2, 118.0, 88.5, 85.9, 59.0, 36.3, 32.5, 29.5, 29.4, 24.7, 19.5.

IR (ATR) 3348, 2954, 2933, 2864, 1466, 1452, 1382, 1360, 1327, 1307, 1278, 1265, 1156, 1097, 1077, 1060, 1026, 989, 956, 938, 904, 873.

HRMS (Sicrit plasma/LTQ-Orbitrap) m/z $[M + H]^+$ calculated for $[C_{12}H_{19}O]^+$: 179.1430; found: 179.1430.

R*f* 0.35 (silica gel, 10:1 pentane / EtOAc, UV / PMA).

HPLC CHIRALPAK® IG, 99:1 hexane / IPA, rate 1 mL / min, 25 min, 230 nm, t_R minor 15.02 min; t_R major 15.57 min.

Opt. Rot. $[\alpha]_D^{20}$ −25.88 (c = 0.38 in $CHCl_3$, >99% ee).

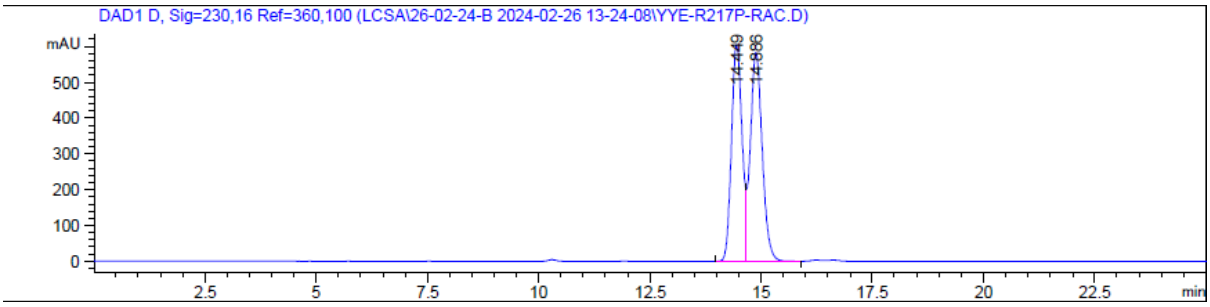

Signal 3: DAD1 D, Sig=230,16 Ref=360,100

Peak #	RetTime [min]	Type	Width [min]	Area [mAU*s]	Height [mAU]	Area %
1	14.449	VV	0.2575	1.00466e4	605.72565	48.0435
2	14.886	VB	0.2807	1.08648e4	584.81610	51.9565

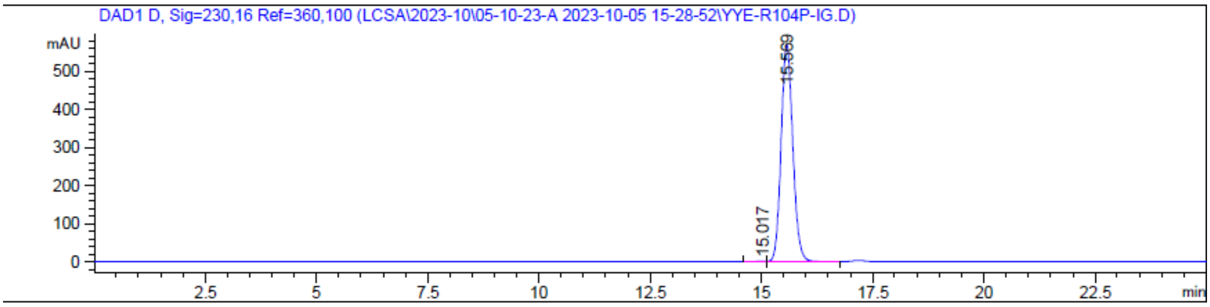

Signal 3: DAD1 D, Sig=230,16 Ref=360,100

Peak #	RetTime [min]	Type	Width [min]	Area [mAU*s]	Height [mAU]	Area %
1	15.017	BV	0.2154	22.83617	1.63045	0.2189
2	15.569	VV	0.2835	1.04113e4	568.95642	99.7811

Alternative preparation of (*S*)-4-(cyclohex-1-en-1-yl)but-3-yn-2-ol (6b) adapted from a kinetic resolution procedure by Guo[12]

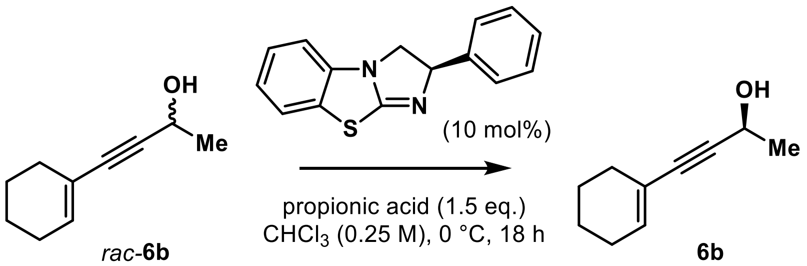

A solution of *rac*-**6b** (0.45 g, 3.0 mmol) in chloroform (12 mL, 0.25 M) was treated with (*R*)-2-phenyl-2,3-dihydrobenzo[d]imidazo[2,1-b]thiazole (76 mg, 0.30 mmol). The resulting solution was cooled to 0 °C (EtOH bath, cryostat) and treated with propionic anhydride (0.58 mL, 4.5 mmol) dropwise. After stirring at 0 °C for 18 h, the reaction was quenched with methanol (15 mL) and diluted with DCM (20 mL). The mixture was successively washed with HCl solution

(20 mL, 1 M), $NaHCO_3$ solution (20 mL, sat. aq.) and brine (20 mL), then the organic layer was dried ($MgSO_4$), filtered and concentrated *in vacuo*. The crude residue was purified by column chromatography (silica gel, 10:1 pentane / EtOAc) to afford **6b** as a colorless oil (0.15 g, 1.0 mmol, 34% yield over 50% maximum theoretical yield, >99% ee). The following characterization data were consistent with literature[13].

^{1}H NMR (400 MHz, $CDCl_3$) δ 6.10 (s, 1H), 4.64 (d, *J* = 6.9 Hz, 1H), 2.14 – 2.03 (m, 4H), 1.76 (bs, 1H), 1.66 – 1.56 (m, 4H), 1.46 (d, *J* = 6.5 Hz, 3H).

^{13}C NMR (101 MHz, $CDCl_3$) δ 135.4, 120.2, 88.4, 86.0, 59.0, 29.3, 25.8, 24.7, 22.4, 21.6.

IR (ATR) 3320, 2979, 2928, 2858, 2839, 1436, 1368, 1269, 1207, 1098, 1076, 1027, 916, 842.

HRMS (ESI + APCI) m/z: $[M + H]^+$ calculated for $[C_{10}H_{15}O]^+$: 151.1117; found: 151.1111.

Rf 0.24 (silica gel, 10:1 pentane / EtOAc, UV / CAM).

HPLC CHIRALPAK® IB, 99:1 hexane / IPA, rate 1 mL / min, 30 min, 230 nm, t_R major 8.04 min; t_R minor 9.33 min.

Opt. Rot. $[\alpha]_D^{20}$ −25.80 (c = 1.0 in $CHCl_3$, >99% ee). Literature: $[\alpha]_D^{21}$ −28.3 (c = 0.454 in $CHCl_3$, 95% ee).

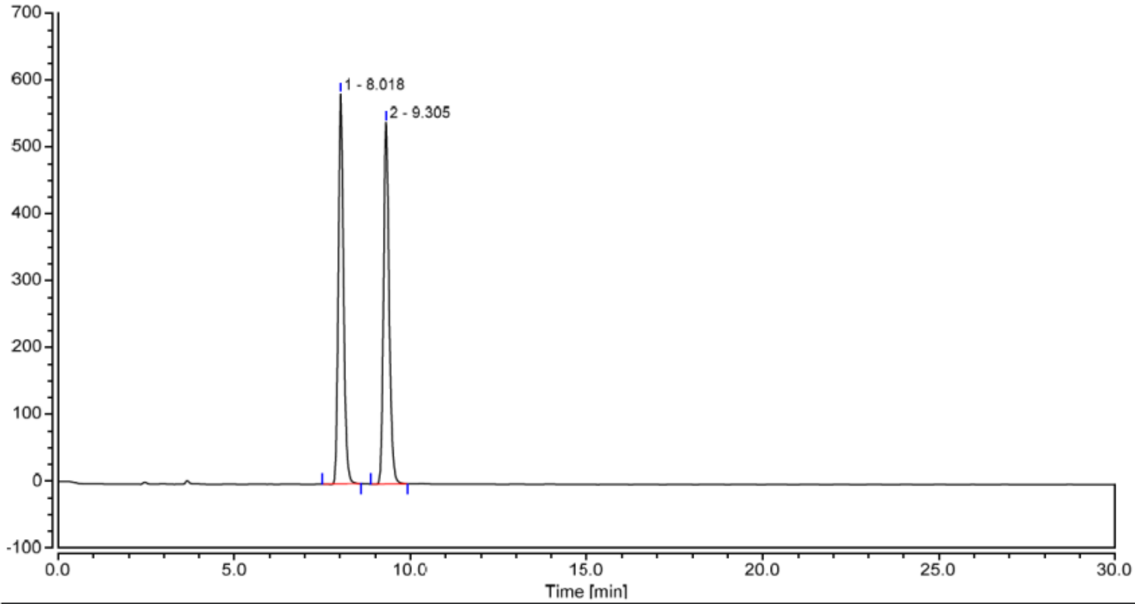

Integration Results							
No.	Peak Name	Retention Time min	Area mAU*min	Height mAU	Relative Area %	Relative Height %	Amount n.a.
1		8.018	99.496	583.100	49.89	51.87	n.a.
2		9.305	99.929	541.109	50.11	48.13	n.a.
Total:			199.426	1124.209	100.00	100.00	

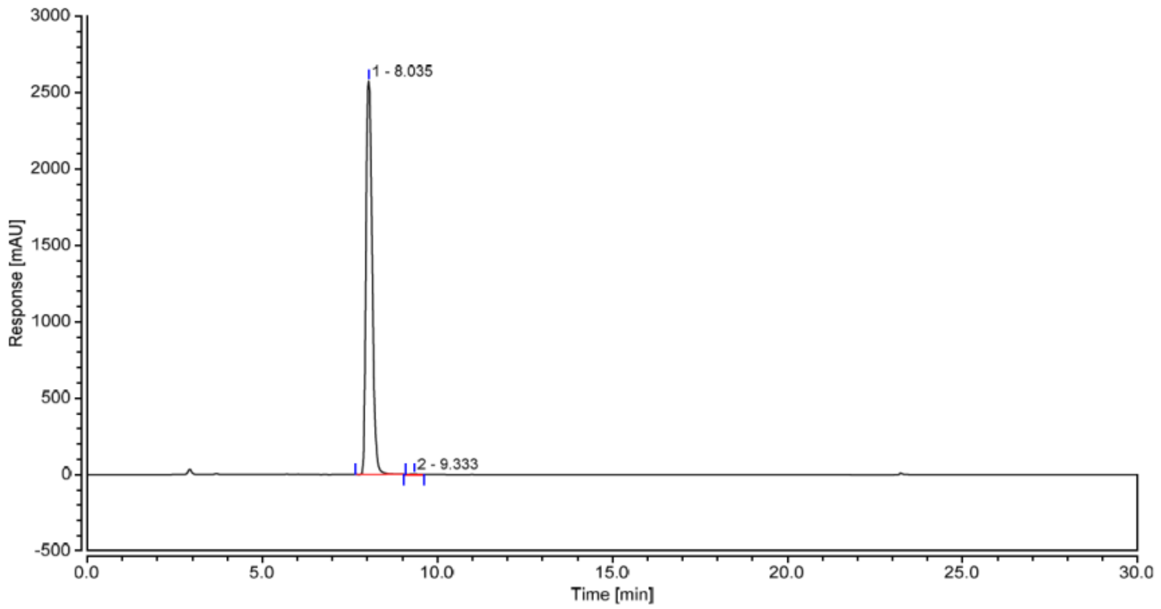

Integration Results							
No.	Peak Name	Retention Time min	Area mAU*min	Height mAU	Relative Area %	Relative Height %	Amount n.a.
1		8.035	527.043	2577.230	99.93	99.92	n.a.
2		9.333	0.376	2.090	0.07	0.08	n.a.
**Total:**			**527.419**	**2579.321**	**100.00**	**100.00**	

General procedure B for the acylation of propargylic alcohols

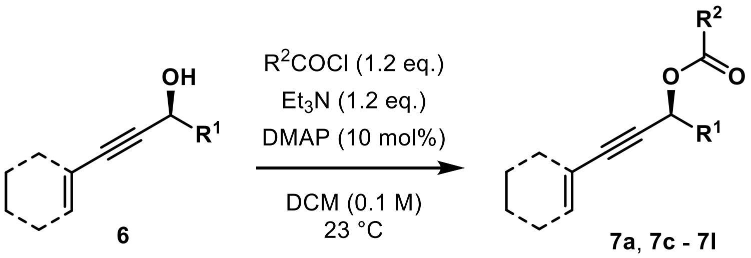

A solution of propargylic alcohol **6** (1.0 eq.), Et_3N (1.2 - 1.5 eq.) and DMAP (10 mol%) in DCM (0.1 M) was treated with acyl chloride (1.1 - 1.5 eq.) and stirred at 23 °C, until TLC indicated the reaction was complete. The reaction mixture was then diluted with pentane (equivolume to DCM) and filtered through celite, washing the solids with pentane. The filtrate was concentrated *in vacuo* and subjected to column chromatography (silica gel) to afford propargylic ester **7**.

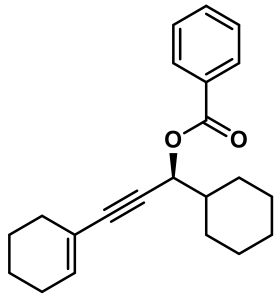

7a

Propargylic ester **7a** was prepared via general procedure **B** with **6a** (7.0 g, 32 mmol, 97% ee), Et_3N (5.0 mL, 38 mmol), DMAP (0.39 g, 3.2 mmol), benzoyl chloride (4.5 mL, 38 mmol), duration 2 h and 10:1 to 3:1 pentane / DCM gradient eluent, to give a colorless oil (9.0 g, 88% yield, 97% ee). The following characterization data were consistent with literature[14].

^{1}H NMR (400 MHz, $CDCl_3$) δ 8.09 – 8.05 (m, 2H), 7.59 – 7.52 (m, 1H), 7.48 – 7.40 (m, 2H), 6.16 – 6.10 (m, 1H), 5.59 (d, J = 5.9 Hz, 1H), 2.16 – 2.03 (m, 4H), 1.98 – 1.74 (m, 6H), 1.72 – 1.66 (m, 1H), 1.64 – 1.56 (m, 3H), 1.32 – 1.14 (m, 5H).

^{13}C NMR (101 MHz, $CDCl_3$) δ 165.8, 135.9, 133.1, 130.5, 129.9, 128.5, 120.2, 88.0, 83.0, 69.6, 42.5, 29.3, 28.8, 28.5, 26.4, 26.0, 26.0, 25.8, 22.4, 21.6.

IR (ATR) 2928, 2854, 1721, 1450, 1345, 1315, 1262, 1175, 1106, 1068, 1025, 969, 951, 710.

HRMS (ESI/QTOF) m/z: $[M + Na]^+$ calculated for $[C_{22}H_{26}NaO_2]^+$: 345.1825; found: 345.1829.

R*f* 0.28 (silica gel, 5:1 pentane / DCM, UV / PMA).

HPLC CHIRALPAK® IA, 99:5:0.5 hexane / IPA, rate 1 mL / min, 25 min, 254 nm, t_R major 5.81 min; t_R minor 6.58 min.

Opt. Rot. $[\alpha]_D^{21}$ −15.69 (c = 1.0 in $CHCl_3$, 97% ee).

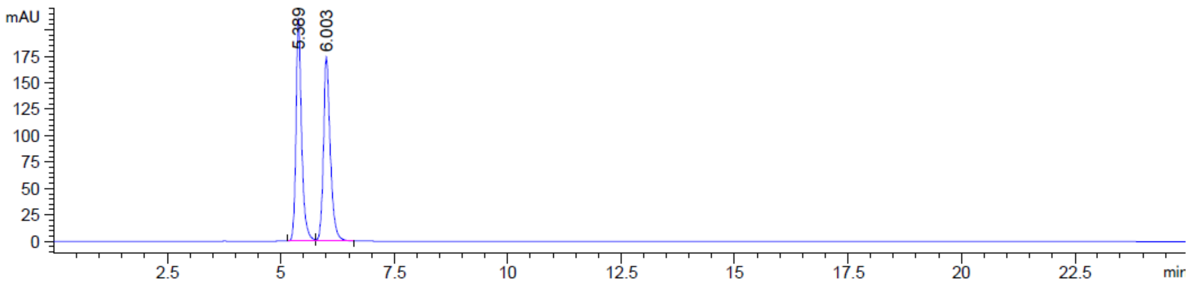

Peak #	RetTime [min]	Type	Width [min]	Area [mAU*s]	Height [mAU]	Area %
1	5.389	BV	0.1270	1819.06445	210.18454	49.9705
2	6.003	VB	0.1538	1821.21558	174.46893	50.0295

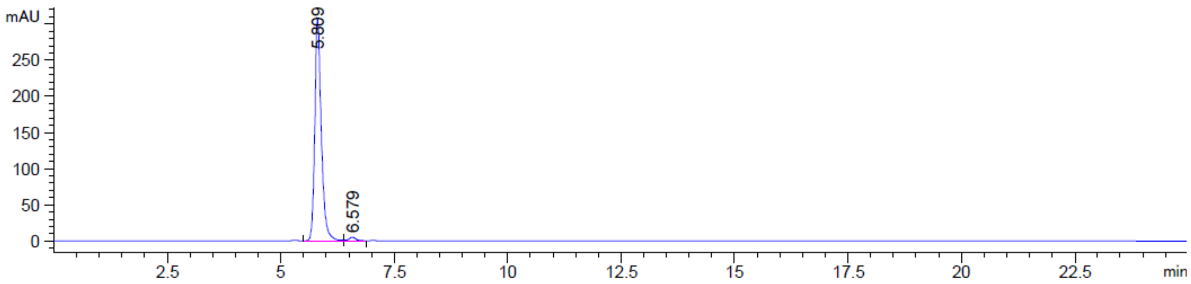

Peak #	RetTime [min]	Type	Width [min]	Area [mAU*s]	Height [mAU]	Area %
1	5.809	BV	0.1484	3070.70459	307.72705	98.3451
2	6.579	VB	0.1610	51.67184	4.74697	1.6549

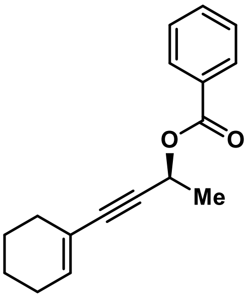

7c

Propargylic ester **7c** was prepared via general procedure **B** with **6b** (0.50 g, 3.3 mmol, >99% ee), Et_3N (0.55 mL, 4.0 mmol), DMAP (40 mg, 0.33 mmol), benzoyl chloride (0.46 mL, 4.0 mmol), duration 2 h and 10:1 to 3:1 pentane / DCM gradient eluent, to give a colorless oil (0.82 g, 97% yield, 98% ee). The following characterization data were consistent with literature[14].

^{1}H NMR (400 MHz, $CDCl_3$) δ 8.13 – 8.03 (m, 2H), 7.61 – 7.51 (m, 1H), 7.50 – 7.40 (m, 2H), 6.20 – 6.08 (m, 1H), 5.83 (q, J = 6.6 Hz, 1H), 2.15 – 2.04 (m, 4H), 1.66 – 1.52 (m, 7H).

^{13}C NMR (101 MHz, $CDCl_3$) δ 165.7, 136.1, 133.2, 130.3, 129.9, 128.5, 120.1, 86.7, 84.9, 61.7,

29.2, 25.8, 22.4, 21.9, 21.6.

IR (ATR) 2932, 1718, 1450, 1337, 1314, 1262, 1094, 1068, 1046, 1023, 1002, 918, 709, 687.

HRMS (ESI/QTOF) m/z: [M + Ag]$^+$ calculated for $[C_{17}H_{18}AgO_2]^+$: 361.0352; found: 361.0353.

Rf 0.15 (silica gel, 5:1 pentane / DCM, UV / PMA).

HPLC CHIRALPAK® IG, 99:1 hexane / IPA, rate 1 mL / min, 25 min, 254 nm, t_R minor 6.46 min; t_R major 7.13 min.

Opt. Rot. $[\alpha]_D^{21}$ −7.67 (c = 1.0 in $CHCl_3$, 98% ee).

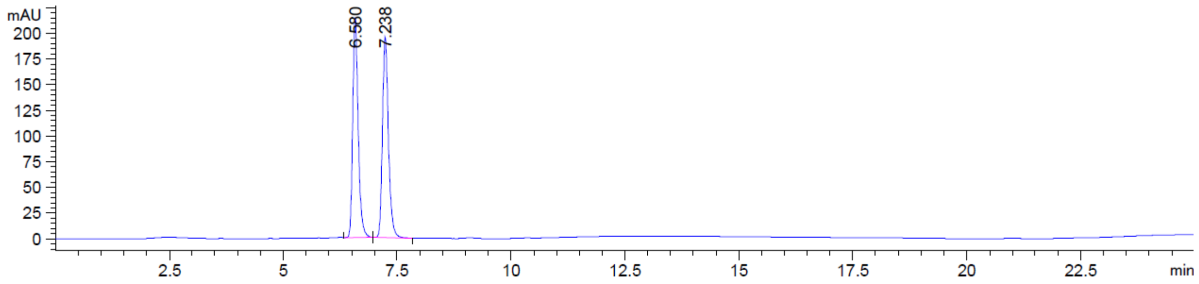

Peak #	RetTime [min]	Type	Width [min]	Area [mAU*s]	Height [mAU]	Area %
1	6.580	BB	0.1268	1783.27930	214.91382	49.9945
2	7.238	BB	0.1385	1783.67407	195.32744	50.0055

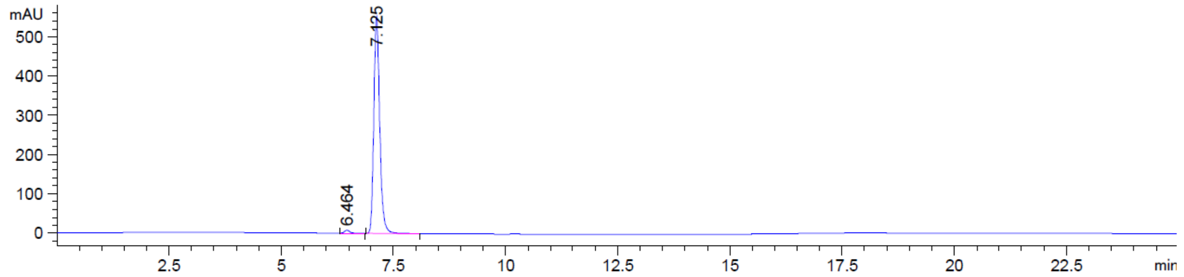

Peak #	RetTime [min]	Type	Width [min]	Area [mAU*s]	Height [mAU]	Area %
1	6.464	BB	0.1244	59.40905	7.34371	1.1672
2	7.125	BB	0.1380	5030.38574	553.49536	98.8328

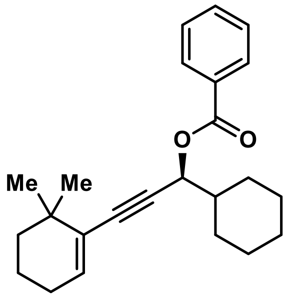

7d

Propargylic ester **7d** was prepared via general procedure **B** with **6c** (267 mg, 1.08 mmol, 92% ee), Et_3N (181 µL, 1.30 mmol), DMAP (13.2 mg, 0.108 mmol), benzoyl chloride (151 µL, 1.30 mmol), duration 2 h and 50:1 pentane / EtOAc eluent, to give a colorless oil (339 mg, 89% yield, 90% ee).

^{1}H NMR (400 MHz, $CDCl_3$) δ 8.13 – 8.01 (m, 2H), 7.60 – 7.51 (m, 1H), 7.44 (t, *J* = 7.6 Hz, 2H), 6.06 (t, *J* = 4.1 Hz, 1H), 5.62 (d, *J* = 5.6 Hz, 1H), 2.06 – 2.00 (m, 2H), 1.97 (d, *J* = 6.9 Hz, 1H), 1.91 – 1.75 (m, 4H), 1.70 (d, *J* = 11.5 Hz, 1H), 1.63 – 1.56 (m, 2H), 1.51 – 1.45 (m, 2H), 1.32 – 1.16 (m, 5H), 1.11 (s, 3H), 1.10 (s, 3H).

^{13}C NMR (101 MHz, $CDCl_3$) δ 165.8, 135.2, 133.1, 130.5, 129.9, 129.7, 128.5, 86.2, 84.6, 77.5, 77.2, 76.8, 69.7, 42.5, 37.7, 33.8, 28.9, 28.8, 28.8, 28.5, 26.5, 26.3, 26.0, 26.0, 18.9.

IR (ATR) 2928, 2854, 1722, 1451, 1315, 1263, 1176, 1107, 1096, 1068, 1026, 968, 954, 711.

HRMS (ESI/QTOF) m/z: $[M + Na]^+$ calculated for $[C_{24}H_{30}NaO_2]^+$: 373.2138; found: 373.2128.

R*f* 0.53 (silica gel, 3:1 pentane / DCM, UV).

HPLC CHIRALPAK® IC, 99.5:0.5 hexane / IPA, rate 0.7 mL / min, 25 min, 210 nm, t_R major 9.43 min; t_R major 10.83 min.

Opt. Rot. $[\alpha]_D^{20}$ −11.46 (c = 1.12 in $CHCl_3$, 90% ee).

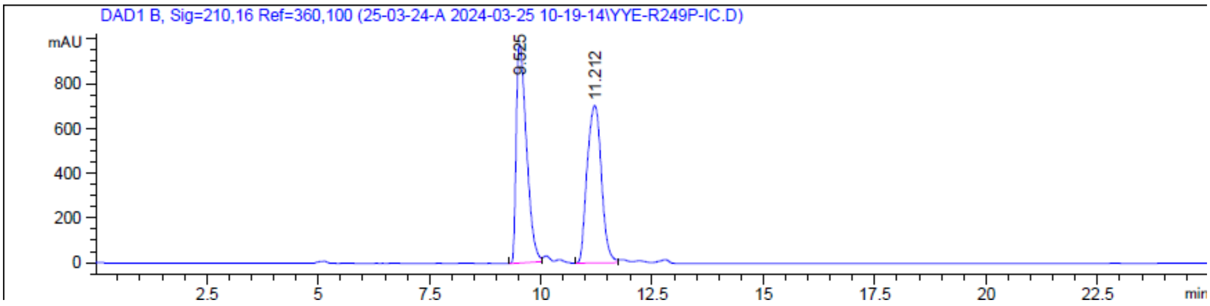

Signal 2: DAD1 B, Sig=210,16 Ref=360,100

Peak #	RetTime [min]	Type	Width [min]	Area [mAU*s]	Height [mAU]	Area %
1	9.525	BV	0.2515	1.56927e4	976.18390	49.9672
2	11.212	BV	0.3664	1.57132e4	703.98169	50.0328

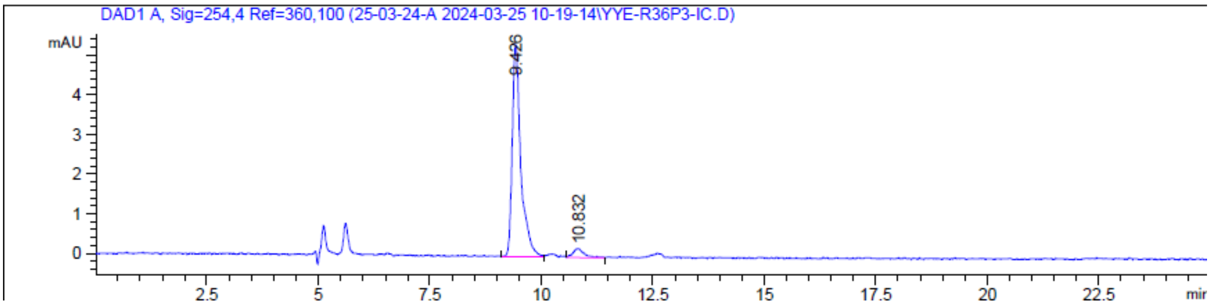

Signal 1: DAD1 A, Sig=254,4 Ref=360,100

Peak #	RetTime [min]	Type	Width [min]	Area [mAU*s]	Height [mAU]	Area %
1	9.426	BV	0.1988	71.85233	5.34662	94.9284
2	10.832	BB	0.2208	3.83873	2.20809e-1	5.0716

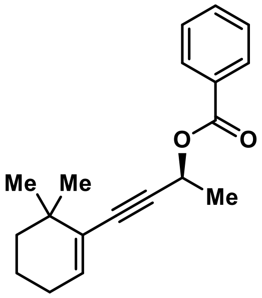

7e

Propargylic ester **7e** was prepared via general procedure **B** with **6d** (0.45 g, 2.5 mmol, 98% ee), Et_3N (0.40 mL, 3.0 mmol), DMAP (31 mg, 0.25 mmol), benzoyl chloride (0.32 mL, 2.8 mmol), duration 2 h and 3:1 pentane / DCM eluent, to give a colorless oil (0.64 g, 89% yield, 98% ee).

^{1}H NMR (400 MHz, $CDCl_3$) δ 8.14 – 8.01 (m, 2H), 7.62 – 7.49 (m, 1H), 7.50 – 7.36 (m, 2H), 6.06 (t, J = 4.1 Hz, 1H), 5.84 (q, J = 6.6 Hz, 1H), 2.11 – 1.96 (m, 2H), 1.69 – 1.54 (m, 6H), 1.52 – 1.45 (m, 1H), 1.10 (d, J = 4.7 Hz, 6H).

^{13}C NMR (101 MHz, $CDCl_3$) δ 165.7, 135.4, 133.1, 130.4, 129.9, 129.6, 128.5, 86.6, 84.9, 61.8, 37.7, 33.8, 28.7, 26.3, 22.0, 18.9.

IR (ATR) 2961, 2935, 1722, 1451, 1341, 1314, 1266, 1196, 1176, 1096, 1069, 1025, 711.

HRMS (Sicrit plasma/LTQ-Orbitrap) m/z: $[M + H]^+$ calculated for $[C_{19}H_{23}O_2]^+$: 283.1693; found: 283.1688.

Rf 0.47 (silica gel, 3:1 pentane / DCM, UV / PMA).

HPLC CHIRALPAK® IG, 99.5:0.5 hexane / IPA, rate 1 mL / min, 15 min, 254 nm, t_R minor 4.72 min; t_R major 5.10 min.

Opt. Rot. $[\alpha]_D^{20}$ −25.00 (c = 1.0 in $CHCl_3$, 98% ee).

Chromatogram

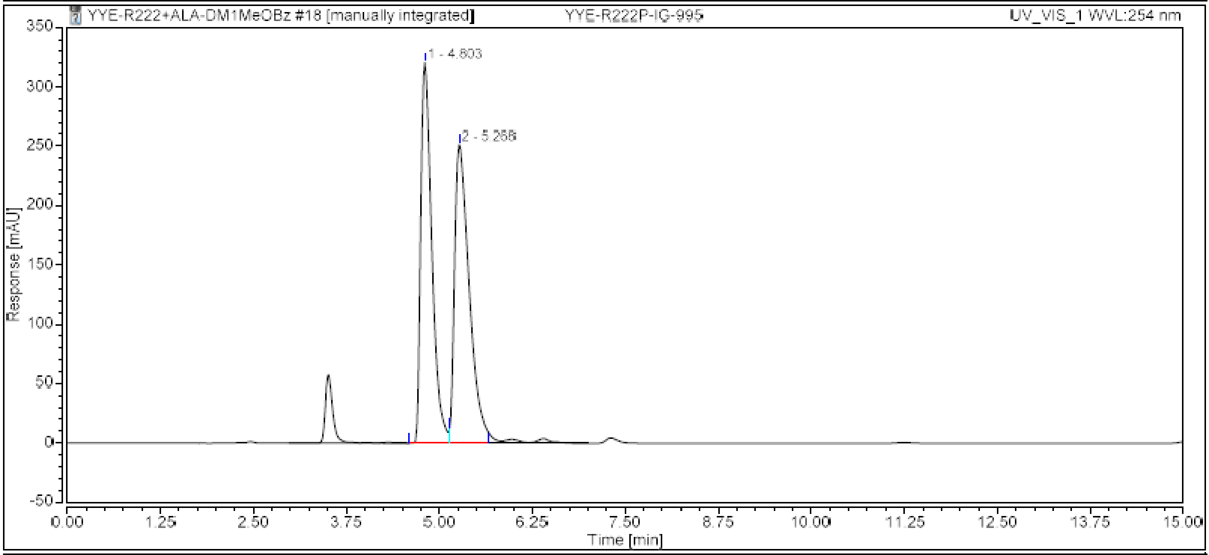

Integration Results

No.	Peak Name	Retention Time min	Area mAU*min	Height mAU	Relative Area %	Relative Height %	Amount n.a.
1		4.803	55.517	320.534	49.08	56.06	n.a.
2		5.268	57.599	251.200	50.92	43.94	n.a.
**Total:**			**113.116**	**571.735**	**100.00**	**100.00**	

Chromatogram

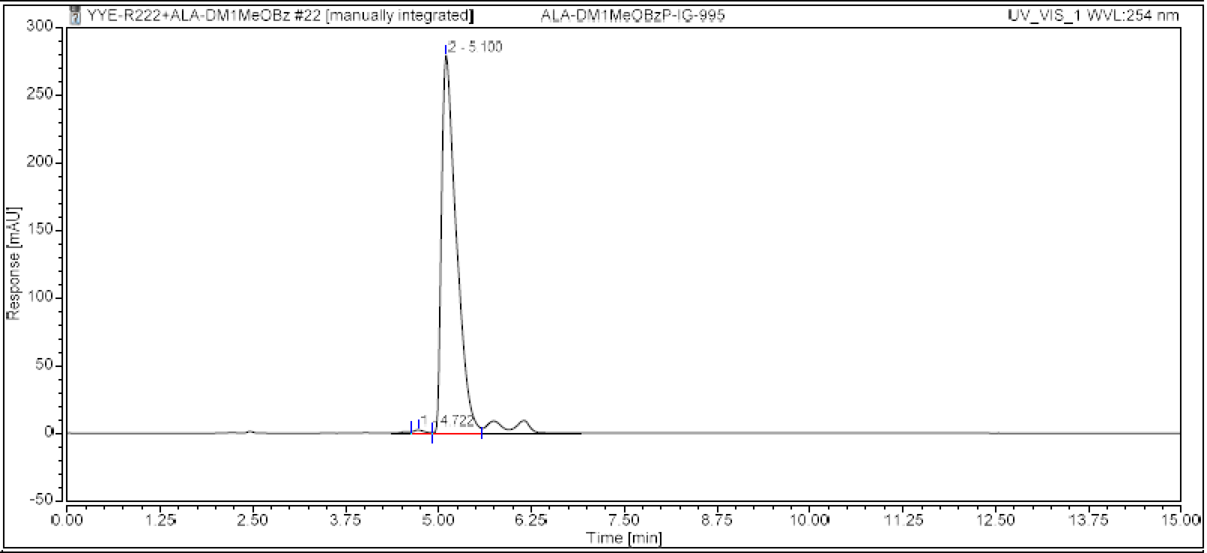

Integration Results

No.	Peak Name	Retention Time min	Area mAU*min	Height mAU	Relative Area %	Relative Height %	Amount n.a.
1		4.722	0.384	2.383	0.59	0.85	n.a.
2		5.100	64.502	279.065	99.41	99.15	n.a.
**Total:**			**64.886**	**281.447**	**100.00**	**100.00**	

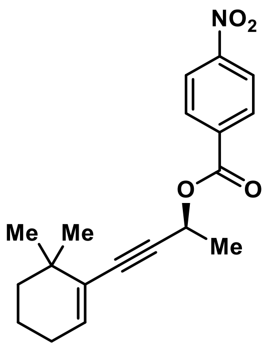

7f

Propargylic ester **7f** was prepared via general procedure **B** with **6d** (100 mg, 0.561 mmol, 98% ee), Et_3N (94 µL, 0.67 mmol), DMAP (6.9 mg, 0.56 mmol), 4-nitrobenzoyl chloride (125 mg, 0.673 mmol), duration 2 h and 50:1 to 20:1 pentane / EtOAc gradient eluent, to give a colorless solid (182 mg, 99% yield, 96% ee).

^{1}H NMR (400 MHz, $CDCl_3$) δ 8.34 – 8.17 (m, 4H), 6.08 (t, *J* = 4.1 Hz, 1H), 5.85 (q, *J* = 6.6 Hz, 1H), 2.04 (td, *J* = 6.1, 4.0 Hz, 2H), 1.67 (d, *J* = 6.6 Hz, 3H), 1.64 – 1.57 (m, 2H), 1.51 – 1.46 (m, 2H), 1.09 (d, *J* = 5.3 Hz, 6H).

^{13}C NMR (101 MHz, $CDCl_3$) δ 163.8, 150.7, 135.9, 135.8, 131.0, 129.4, 123.7, 85.8, 85.6, 63.1, 37.6, 33.8, 28.7, 26.3, 21.9, 18.9.

IR (ATR) 2866, 1726, 1608, 1528, 1458, 1410, 1341, 1316, 1265, 1196, 1115, 1098, 1076, 1037, 1014, 874, 848, 815, 784, 719, 504.

HRMS Ion peak not observed in ESI+, APCI+, ESI+Ag+ or GCMS.

m.p. 58.6 – 59.5 °C (racemate).

R*f* 0.58 (silica gel, 50:1 pentane / EtOAc, UV).

HPLC CHIRALPAK® IF, 99:5:0.5 hexane / IPA, rate 1 mL / min, 25 min, 210 nm, t_R major 10.99 min; t_R minor 12.16 min.

Opt. Rot. $[\alpha]_D^{20}$ +1.75 (c = 0.38 in $CHCl_3$, 96% ee).

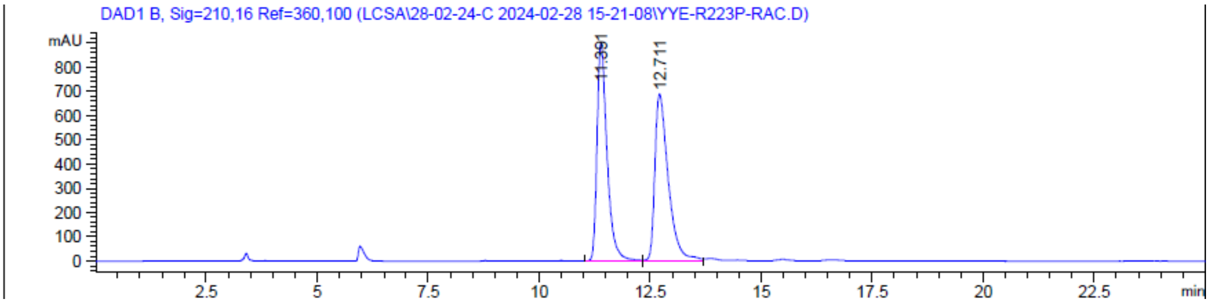

Signal 2: DAD1 B, Sig=210,16 Ref=360,100

Peak #	RetTime [min]	Type	Width [min]	Area [mAU*s]	Height [mAU]	Area %
1	11.391	BV	0.2381	1.41995e4	899.86554	49.4707
2	12.711	VV	0.3156	1.45033e4	689.05536	50.5293

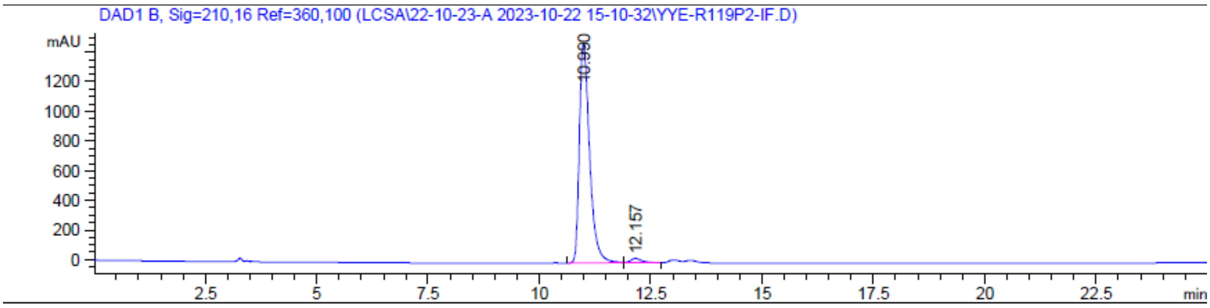

Signal 2: DAD1 B, Sig=210,16 Ref=360,100

Peak #	RetTime [min]	Type	Width [min]	Area [mAU*s]	Height [mAU]	Area %
1	10.990	BV	0.2450	2.36825e4	1477.86414	97.8292
2	12.157	VB	0.2579	525.51459	29.77932	2.1708
Totals :				2.42081e4	1507.64346	

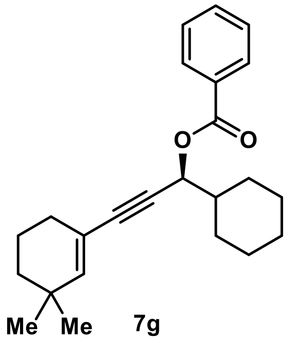

7g

Propargylic ester **7g** was prepared via general procedure **B** with **6e** (50.0 mg, 0.200 mmol, 93% ee), Et_3N (34 µL, 0.244 mmol), DMAP (2.5 mg, 20 µmol), benzoyl chloride (28 µL, 0.244 mmol), duration 2 h and 50:1 pentane / EtOAc eluent, to give a colorless oil (67.4 mg, 95% yield, 92% ee).

^{1}H NMR (400 MHz, $CDCl_3$) δ 8.11 – 8.04 (m, 2H), 7.59 – 7.52 (m, 1H), 7.48 – 7.41 (m, 2H), 5.86 (s, 1H), 5.58 (d, *J* = 6.0 Hz, 1H), 2.06 (td, *J* = 6.3, 1.9 Hz, 2H), 1.97 – 1.75 (m, 5H), 1.73 – 1.66 (m, 1H), 1.66 – 1.58 (m, 2H), 1.42 – 1.36 (m, 2H), 1.30 – 1.16 (m, 5H), 0.97 (s, 6H).

^{13}C NMR (101 MHz, $CDCl_3$) δ 165.8, 145.4, 133.1, 130.4, 129.9, 128.5, 118.0, 87.9, 83.1, 69.5,

42.5, 36.3, 32.5, 29.5, 29.4, 28.8, 28.5, 26.4, 26.0, 26.0, 19.5.

IR (ATR) 2929, 2854, 1721, 1451, 1315, 1262, 1176, 1106, 1096, 1068, 1026, 993, 967, 938, 873, 803, 711.

HRMS (ESI/QTOF) m/z: $[M + K]^+$ calculated for $[C_{24}H_{30}KO_2]^+$: 389.1877; found: 389.1870.

Rf 0.50 (silica gel, 50:1 pentane / EtOAc, UV).

HPLC CHIRALPAK® IA, 99.5:0.5 hexane / IPA, rate 0.5 mL / min, 25 min, 254 nm, t_R major 9.93 min; t_R minor 11.52 min.

Opt. Rot. $[\alpha]_D^{20}$ −17.82 (c = 0.29 in $CHCl_3$, 92% ee).

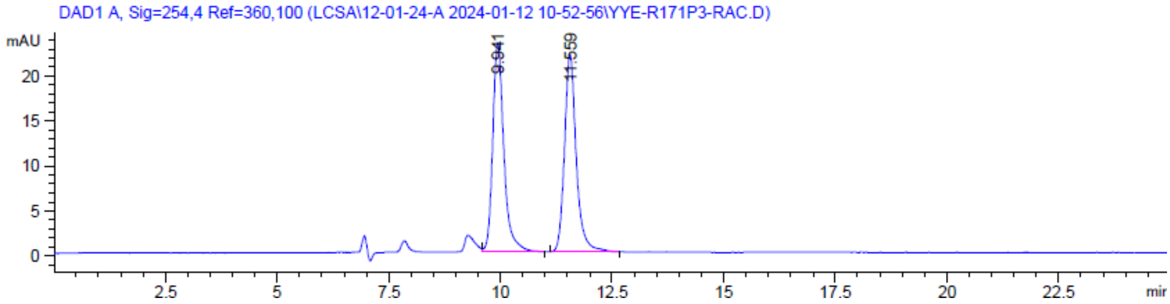

Signal 1: DAD1 A, Sig=254,4 Ref=360,100

Peak #	RetTime [min]	Type	Width [min]	Area [mAU*s]	Height [mAU]	Area %
1	9.941	VB	0.2623	402.31995	23.20190	50.1198
2	11.559	BB	0.2728	400.39639	21.94781	49.8802

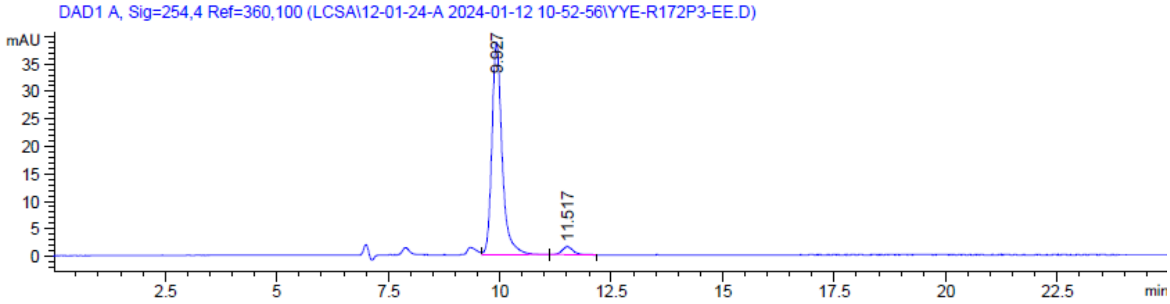

Signal 1: DAD1 A, Sig=254,4 Ref=360,100

Peak #	RetTime [min]	Type	Width [min]	Area [mAU*s]	Height [mAU]	Area %
1	9.927	VB	0.2395	615.19958	38.68152	96.0974
2	11.517	BB	0.2418	24.98360	1.47393	3.9026

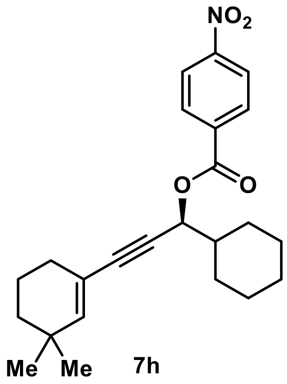

7h

Propargylic ester **7h** was prepared via general procedure **B** with **6e** (19.8 mg, 80.4 µmol, 93% ee), Et_3N (15 µL, 0.10 mmol), DMAP (1.0 mg, 8.0 µmol), 4-nitrobenzoyl chloride (19.3 mg, 0.104 mmol), duration 2 h and 50:1 pentane / EtOAc eluent, to give a colorless solid (31.7 mg, 99% yield, 93% ee).

^{1}H NMR (400 MHz, $CDCl_3$) δ 8.32 – 8.19 (m, 4H), 5.88 (s, 1H), 5.60 (d, J = 5.8 Hz, 1H), 2.06 (td, J = 6.2, 1.9 Hz, 2H), 1.94 (d, J = 10.8 Hz, 1H), 1.90 – 1.76 (m, 4H), 1.71 (d, J = 11.6 Hz, 1H), 1.65 – 1.58 (m, 2H), 1.43 – 1.37 (m, 2H), 1.30 – 1.15 (m, 5H), 0.98 (s, 6H).

^{13}C NMR (101 MHz, $CDCl_3$) δ 163.9, 150.7, 145.9, 135.8, 131.1, 123.7, 117.9, 88.6, 82.3, 70.7, 42.4, 36.3, 32.5, 29.5, 29.3, 28.8, 28.5, 26.3, 25.9, 25.9, 19.5.

IR (ATR) 2930, 2855, 1728, 1607, 1530, 1451, 1344, 1318, 1268, 1112, 1100, 1015, 964, 942, 890, 873, 847, 719.

HRMS Ion peak not observed in ESI+.

m.p. 69.2 – 70.3 °C (racemate).

R*f* 0.56 (silica gel, 50:1 pentane / EtOAc, UV).

HPLC CHIRALPAK® IC, 99.5:0.5 hexane / IPA, rate 1 mL / min, 25 min, 254 nm, t_R major 14.67 min; t_R minor 16.32 min.

Opt. Rot. $[\alpha]_D^{20}$ +2.33 (c = 0.50 in $CHCl_3$, 93% ee).

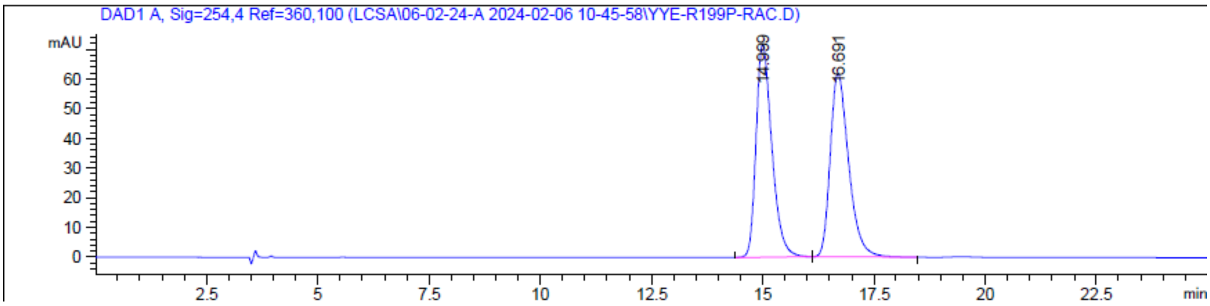

Signal 1: DAD1 A, Sig=254,4 Ref=360,100

Peak #	RetTime [min]	Type	Width [min]	Area [mAU*s]	Height [mAU]	Area %
1	14.999	BB	0.3790	1773.58838	71.60828	49.9186
2	16.691	BB	0.4423	1779.36951	61.72145	50.0814

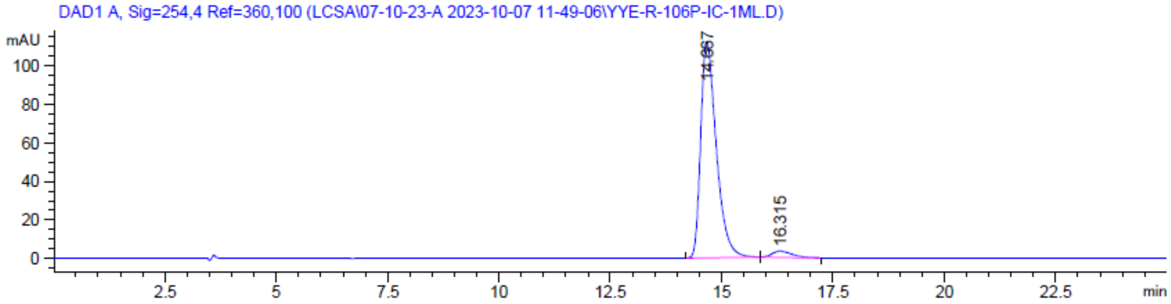

Signal 1: DAD1 A, Sig=254,4 Ref=360,100

Peak #	RetTime [min]	Type	Width [min]	Area [mAU*s]	Height [mAU]	Area %
1	14.667	BB	0.3701	2734.69873	112.28848	96.4349
2	16.315	BB	0.4183	101.09825	3.25537	3.5651

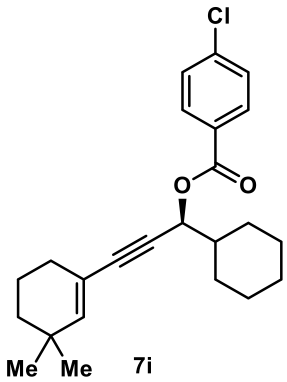

7i

Propargylic ester **7i** was prepared via general procedure **B** with **6e** (20.5 mg, 83.2 µmol, 93% ee), Et_3N (15 µL, 0.10 mmol), DMAP (1.0 mg, 8.0 µmol), 4-chlorobenzoyl chloride (14 µL, 0.11 mmol), duration 2 h and 50:1 pentane / EtOAc eluent, to give a colorless oil (30.9 mg, 96% yield, 93% ee).

^{1}H NMR (400 MHz, $CDCl_3$) δ 8.00 (d, J = 8.6 Hz, 2H), 7.41 (d, J = 8.6 Hz, 2H), 5.86 (s, 1H), 5.57 (d, J = 5.9 Hz, 1H), 2.06 (td, J = 6.2, 1.9 Hz, 2H), 1.93 (d, J = 11.0 Hz, 1H), 1.89 – 1.73 (m, 4H), 1.69 (d, J = 11.3 Hz, 1H), 1.65 – 1.57 (m, 2H), 1.42 – 1.36 (m, 2H), 1.31 – 1.12 (m, 5H), 0.97 (s, 6H).

^{13}C NMR (101 MHz, $CDCl_3$) δ 164.9, 145.6, 139.6, 131.3, 128.9, 128.8, 118.0, 88.1, 82.8, 69.9, 42.4, 36.3, 32.5, 29.5, 29.4, 28.8, 28.5, 26.4, 26.0, 25.9, 19.5.

IR (ATR) 2930, 2854, 1724, 1595, 1489, 1451, 1401, 1264, 1171, 1111, 1093, 1015, 966, 943, 849, 758.

HRMS (ESI/QTOF) m/z: $[M + Na]^+$ calculated for $[C_{24}H_{29}ClNaO_2]^+$: 407.1748; found: 407.1745.

Rf 0.50 (silica gel, 50:1 pentane / EtOAc, UV).

HPLC CHIRALPAK® IA, 99.5:0.5 hexane / IPA, rate 1 mL / min, 25 min, 210 nm, t_R major 4.97 min; t_R minor 5.58 min.

Opt. Rot. $[\alpha]_D^{20}$ +2.78 (c = 0.42 in $CHCl_3$, 93% ee).

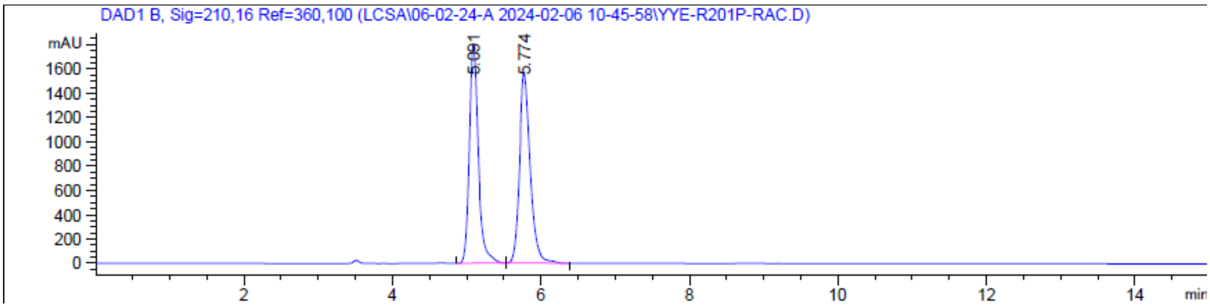

Signal 2: DAD1 B, Sig=210,16 Ref=360,100

Peak #	RetTime [min]	Type	Width [min]	Area [mAU*s]	Height [mAU]	Area %
1	5.091	BB	0.1259	1.51095e4	1799.80066	49.0127
2	5.774	BV	0.1492	1.57182e4	1564.00439	50.9873

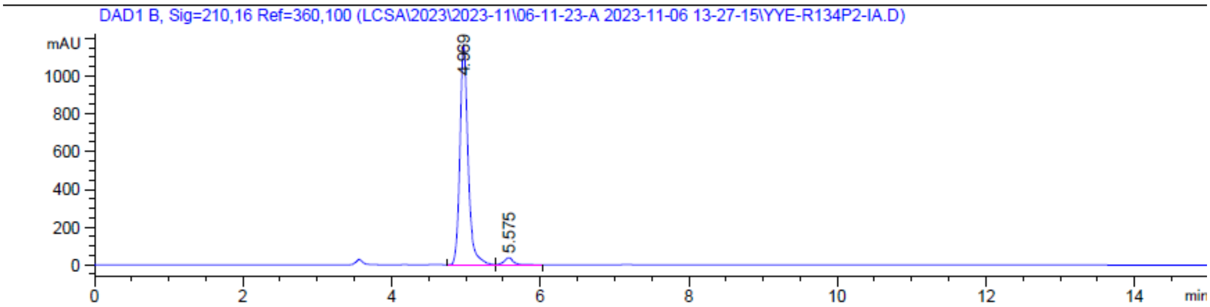

Signal 2: DAD1 B, Sig=210,16 Ref=360,100

Peak #	RetTime [min]	Type	Width [min]	Area [mAU*s]	Height [mAU]	Area %
1	4.969	VV	0.1162	8775.48340	1160.46033	96.3474
2	5.575	VB	0.1307	332.68057	37.77397	3.6526

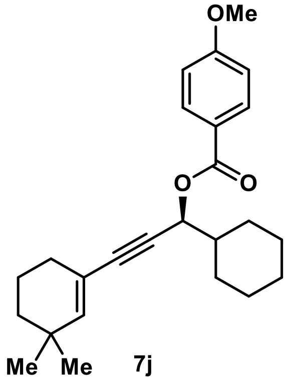

7j

Propargylic ester **7j** was prepared via general procedure **B** with **6e** (50.0 mg, 0.202 mmol, 93% ee), Et_3N (42 µL, 0.30 mmol), DMAP (2.5 mg, 20 µmol), 4-methoxybenzoyl chloride (41 µL, 0.30 mmol), duration 18 h and 50:1 pentane / EtOAc eluent, to give a colorless oil (76.8 mg, 99% yield, 93% ee).

^{1}H NMR (400 MHz, $CDCl_3$) δ 8.02 (d, J = 8.9 Hz, 2H), 6.92 (d, J = 8.9 Hz, 2H), 5.85 (s, 1H), 5.56 (d, J = 6.0 Hz, 1H), 3.86 (s, 3H), 2.06 (td, J = 6.3, 1.9 Hz, 2H), 1.93 (d, J = 10.3 Hz, 1H), 1.87 (d, J = 13.4 Hz, 1H), 1.83 – 1.72 (m, 3H), 1.69 (d, J = 11.3 Hz, 1H), 1.65 – 1.59 (m, 2H), 1.41 – 1.36 (m, 2H), 1.30 – 1.12 (m, 5H), 0.97 (s, 6H).

^{13}C NMR (101 MHz, $CDCl_3$) δ 165.5, 163.5, 145.3, 132.0, 122.8, 118.1, 113.7, 87.7, 83.3, 69.2, 55.6, 42.5, 36.4, 32.5, 29.5, 29.4, 28.8, 28.6, 26.4, 26.0, 26.0, 19.5.

IR (ATR) 2930, 2854, 1716, 1606, 1581, 1510, 1451, 1316, 1255, 1167, 1097, 1031, 1008, 968, 874, 846, 769, 697.

HRMS (ESI/QTOF) m/z: $[M + Na]^+$ calculated for $[C_{25}H_{32}NaO_3]^+$: 403.2244; found: 403.2243.

R*f* 0.56 (silica gel, 20:1 pentane / EtOAc, UV).

HPLC CHIRALPAK® IC, 99:1 hexane / IPA, rate 1 mL / min, 15 min, 230 nm, t_R major 9.06 min; t_R minor 11.83 min.

Opt. Rot. $[\alpha]_D^{20}$ +10.48 (c = 1.05 in $CHCl_3$, 93% ee).

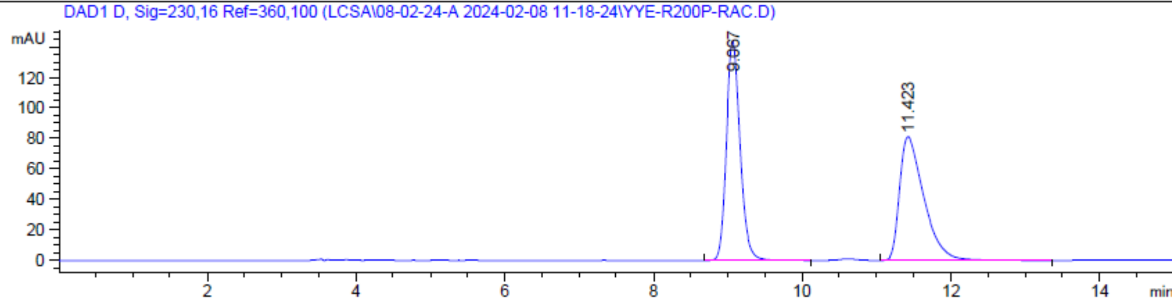

Signal 3: DAD1 D, Sig=230,16 Ref=360,100

Peak #	RetTime [min]	Type	Width [min]	Area [mAU*s]	Height [mAU]	Area %
1	9.067	BB	0.1971	1838.66565	143.89531	50.0053
2	11.423	BB	0.3445	1838.27734	81.07939	49.9947

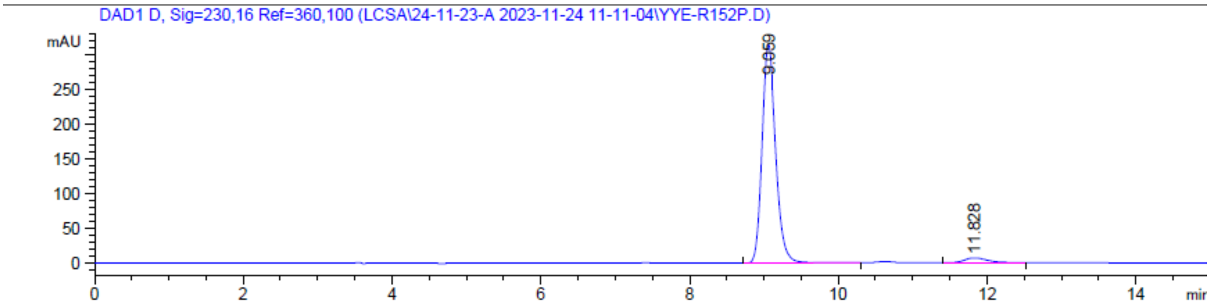

Signal 3: DAD1 D, Sig=230,16 Ref=360,100

Peak #	RetTime [min]	Type	Width [min]	Area [mAU*s]	Height [mAU]	Area %
1	9.059	BB	0.1935	3971.62817	314.23273	96.3426
2	11.828	BB	0.3240	150.77100	7.09227	3.6574

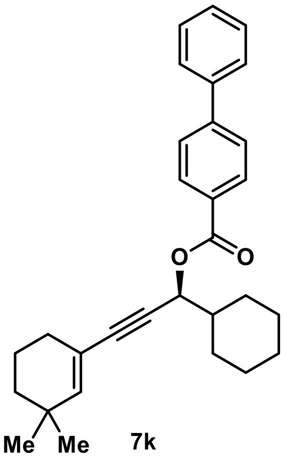

7k

Propargylic ester **7k** was prepared via general procedure **B** with **6e** (50.0 mg, 0.202 mmol, 93% ee), Et_3N (37 µL, 0.26 mmol), DMAP (2.5 mg, 20 µmol), biphenyl-4-carbonyl chloride (57.2 mg, 0.264 mmol), duration 2 h and 50:1 pentane / EtOAc eluent, to give a colorless oil (85.5 mg, 99% yield, 92% ee).

^{1}H NMR (400 MHz, $CDCl_3$) δ 8.07 (d, J = 8.4 Hz, 2H), 7.59 (d, J = 8.4 Hz, 2H), 7.55 (d, J = 7.0 Hz, 2H), 7.40 (t, J = 7.4 Hz, 2H), 7.33 (t, J = 7.3 Hz, 1H), 5.80 (s, 1H), 5.54 (d, J = 5.9 Hz, 1H), 2.00 (td, J = 6.2, 1.9 Hz, 2H), 1.92 – 1.69 (m, 5H), 1.63 (d, J = 10.9 Hz, 1H), 1.60 – 1.51 (m, 2H), 1.35 – 1.29 (m, 2H), 1.27 – 1.11 (m, 5H), 0.91 (s, 6H).

^{13}C NMR (101 MHz, $CDCl_3$) δ 165.7, 145.9, 145.4, 140.2, 130.5, 129.2, 129.1, 128.3, 127.4,

127.2, 118.1, 87.9, 83.1, 69.6, 42.5, 36.4, 32.5, 29.6, 29.4, 28.9, 28.6, 26.4, 26.0, 26.0, 19.5.

IR (ATR) 2929, 2854, 1720, 1609, 1450, 1405, 1342, 1314, 1263, 1178, 1098, 1008, 967, 857, 748, 698.

HRMS (ESI/QTOF) m/z: [M + Na]$^+$ calculated for $[C_{30}H_{34}NaO_2]^+$: 449.2451; found: 449.2443.

Rf 0.46 (silica gel, 50:1 pentane / EtOAc, UV).

HPLC CHIRALPAK® IF, 99.5:0.5 hexane / IPA, rate 1 mL / min, 25 min, 230 nm, t_R major 10.53 min; t_R minor 11.33 min.

Opt. Rot. $[\alpha]_D^{20}$ +33.97 (c = 0.52 in $CHCl_3$, 92% ee).

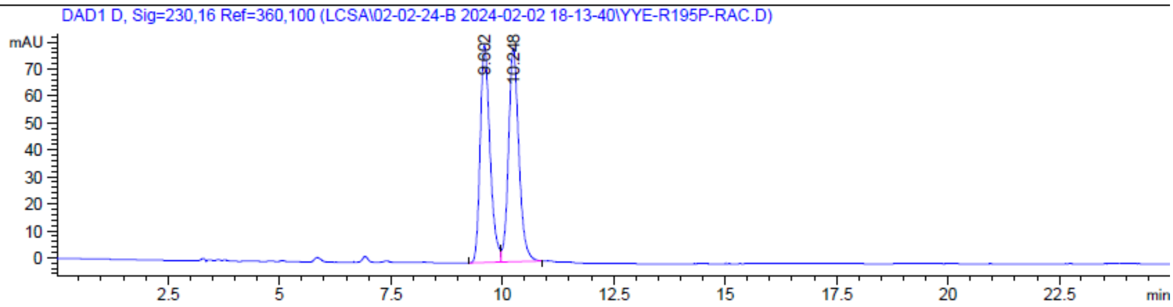

Signal 3: DAD1 D, Sig=230,16 Ref=360,100

Peak #	RetTime [min]	Type	Width [min]	Area [mAU*s]	Height [mAU]	Area %
1	9.602	BV	0.2327	1238.86340	80.89399	49.2133
2	10.248	VB	0.2462	1278.47351	79.27363	50.7867

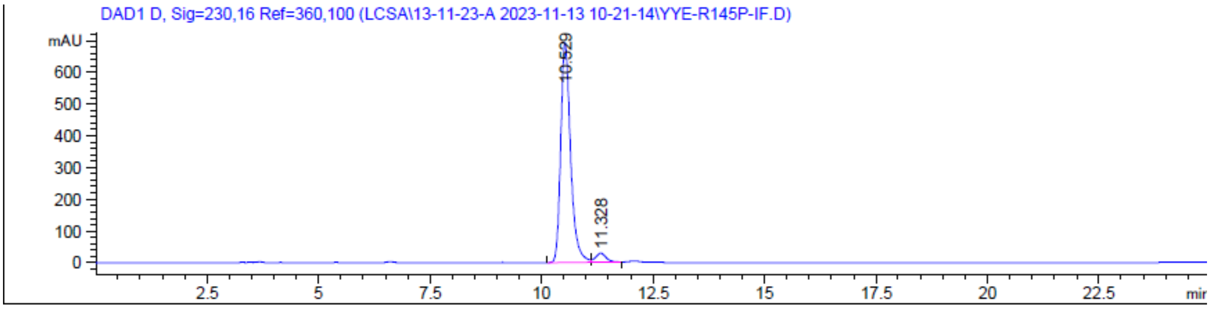

Signal 3: DAD1 D, Sig=230,16 Ref=360,100

Peak #	RetTime [min]	Type	Width [min]	Area [mAU*s]	Height [mAU]	Area %
1	10.529	BV	0.2335	1.06398e4	691.49084	95.7597
2	11.328	VB	0.2490	471.13901	28.47817	4.2403

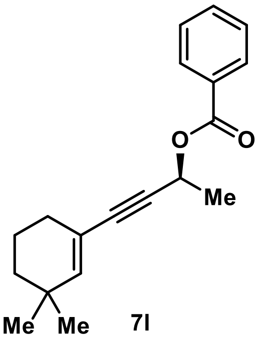

7l

Propargylic ester **7l** was prepared via general procedure **B** with **6f** (53.5 mg, 0.300 mmol, >99% ee), Et_3N (54 µL, 0.39 mmol), DMAP (3.7 mg, 30 µmol), benzoyl chloride (45 µL, 0.39 mmol), duration 2 h and 50:1 pentane / EtOAc eluent, to give a colorless oil (76.5 mg, 90% yield, >99% ee).

^{1}H NMR (400 MHz, $CDCl_3$) δ 8.12 – 8.02 (m, 2H), 7.56 (t, *J* = 7.4 Hz, 1H), 7.44 (t, *J* = 7.7 Hz, 2H), 5.89 (s, 1H), 5.83 (q, *J* = 6.6 Hz, 1H), 2.06 (td, *J* = 6.2, 1.9 Hz, 2H), 1.66 – 1.59 (m, 5H), 1.42 – 1.37 (m, 2H), 0.98 (s, 6H).

^{13}C NMR (101 MHz, $CDCl_3$) δ 165.7, 145.8, 133.2, 130.3, 129.9, 128.4, 117.9, 86.6, 85.0, 61.7, 36.3, 32.5, 29.5, 29.3, 21.9, 19.5.

IR (ATR) 2954, 2935, 2864, 1721, 1451, 1338, 1315, 1265, 1096, 1081, 1069, 1025, 711.

HRMS (ESI/QTOF) m/z: $[M + Na]^+$ calculated for $[C_{19}H_{22}NaO_2]^+$: 305.1512; found: 305.1516.

R*f* 0.61 (silica gel, 50:1 pentane / EtOAc, UV).

HPLC CHIRALPAK® IC, 99.5:0.5 hexane / IPA, rate 0.7 mL / min, 25 min, 254 nm, t_R minor 9.69 min; t_R major 10.77 min.

Opt. Rot. $[\alpha]_D^{20}$ −20.74 (c = 0.45 in $CHCl_3$, >99% ee).

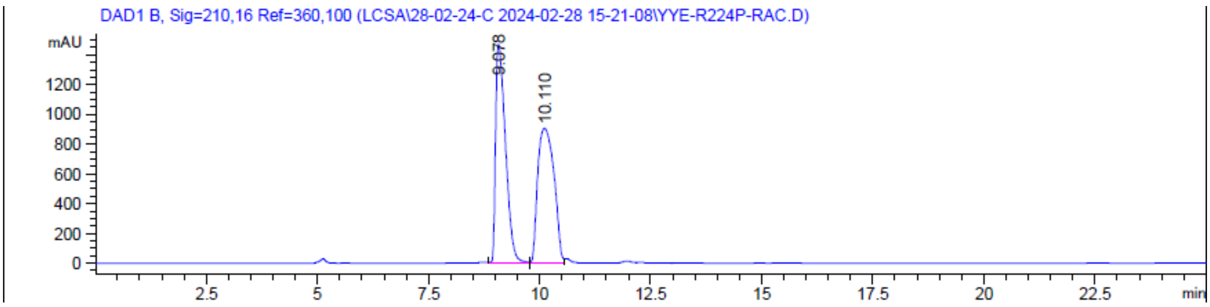

Signal 2: DAD1 B, Sig=210,16 Ref=360,100

Peak #	RetTime [min]	Type	Width [min]	Area [mAU*s]	Height [mAU]	Area %
1	9.078	VV	0.2430	2.27816e4	1468.25427	49.6030
2	10.110	VV	0.4483	2.31463e4	905.31232	50.3970

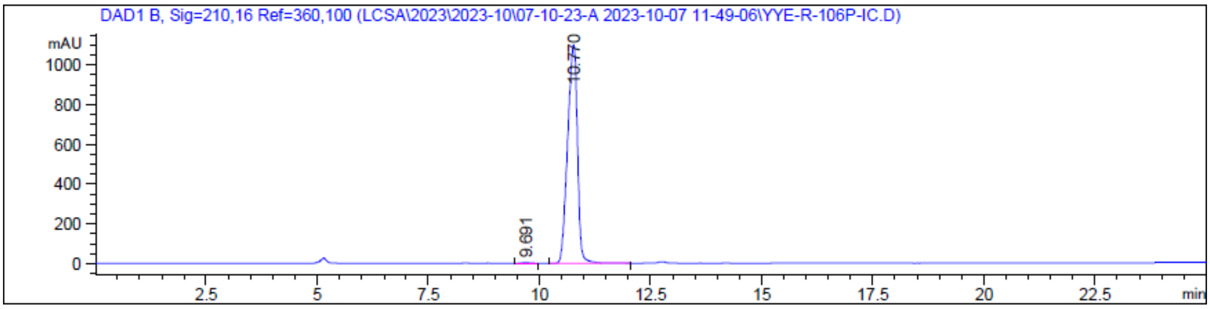

Signal 2: DAD1 B, Sig=210,16 Ref=360,100

Peak #	RetTime [min]	Type	Width [min]	Area [mAU*s]	Height [mAU]	Area %
1	9.691	BB	0.1761	44.75250	3.83702	0.2633
2	10.770	BB	0.2494	1.69542e4	1102.42285	99.7367

Synthesis of (*S*)-3-(cyclohex-1-en-1-yl)-1-cyclohexylprop-2-yn-1-yl pivalate (7b) via racemate synthesis and chiral separation

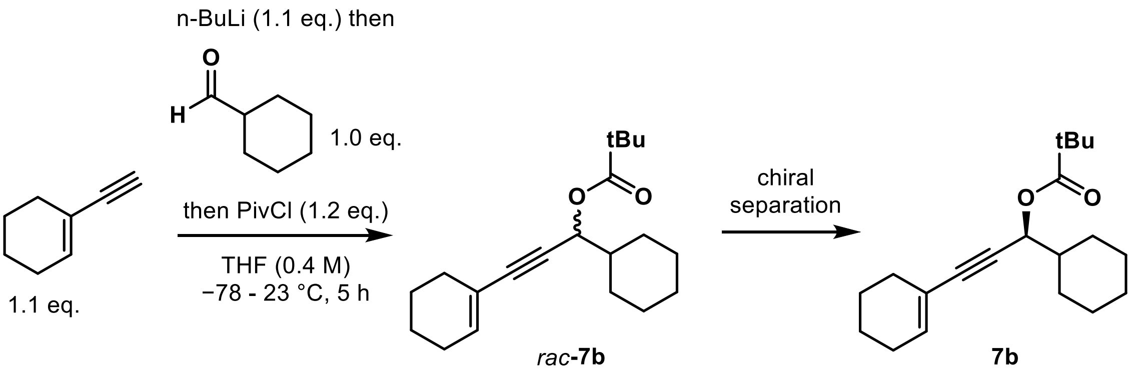

A solution of 1-ethynylcyclohex-1-ene (0.12 mL, 0.98 mmol) in dry THF (2.5 mL) at −78 °C was treated dropwise with *n*BuLi (0.50 mL, 1.9 M in hexane), then stirred for 1 h before the dropwise addition of cyclohexanecarbaldehyde (0.11 mL, 0.89 mmol). After stirring for an additional 30 min, the reaction mixture was slowly warmed to 23 °C over a further 1.5 h, before being treated with pivaloyl chloride (0.13 mL, 1.1 mol). After another 2 h of stirring, the reaction mixture was quenched by addition of NH_4Cl solution (2.5 mL, sat. aq.). The separated aqueous layer was extracted with Et_2O (3 × 5 mL), and the combined organic layers were washed with brine, dried ($MgSO_4$), filtered and concentrated *in vacuo*. The resulting crude residue was subjected to column chromatography (silica gel, 20:1 pentane / EtOAc) to

afford *rac*-**7b** as a pale yellow oil (210 mg, 84% yield). The enantiopure ester **7b** was obtained by separation on preparative HPLC (CHIRALPAK® IA, 99:1 hexane / IPA, rate 18 mL / min, 254 nm).

^{1}H NMR (400 MHz, $CDCl_3$) δ 6.15 – 6.00 (m, 1H), 5.29 (d, *J* = 6.0 Hz, 1H), 2.15 – 2.02 (m, 4H), 1.89 – 1.72 (m, 4H), 1.71 – 1.52 (m, 7H), 1.21 (s, 9H), 1.16 – 0.99 (m, 4H).

^{13}C NMR (101 MHz, $CDCl_3$) δ 177.6, 135.4, 120.3, 87.4, 83.3, 68.7, 42.3, 39.0, 29.3, 28.7, 28.5, 27.3, 26.5, 26.0, 25.7, 22.4, 21.6.

IR (ATR) 2926, 2854, 1732, 1478, 1449, 1362, 1345, 1278, 1148, 1137, 1079, 1030, 953, 918.

HRMS (ESI/QTOF) m/z: $[M + Na]^+$ calculated for $[C_{20}H_{30}NaO_2]^+$: 325.2138; found: 325.2136.

Rf 0.61 (silica gel, pentane / EtOAc 20:1, UV / CAM).

HPLC CHIRALPAK® IA, 99:1 hexane / IPA, rate 1 mL / min, 25 min, 254 nm, t_R major 4.19 min; t_R minor 5.05 min.

Opt. Rot. $[\alpha]_D^{21}$ −71.00 (c = 1.0 in $CHCl_3$, >99% ee).

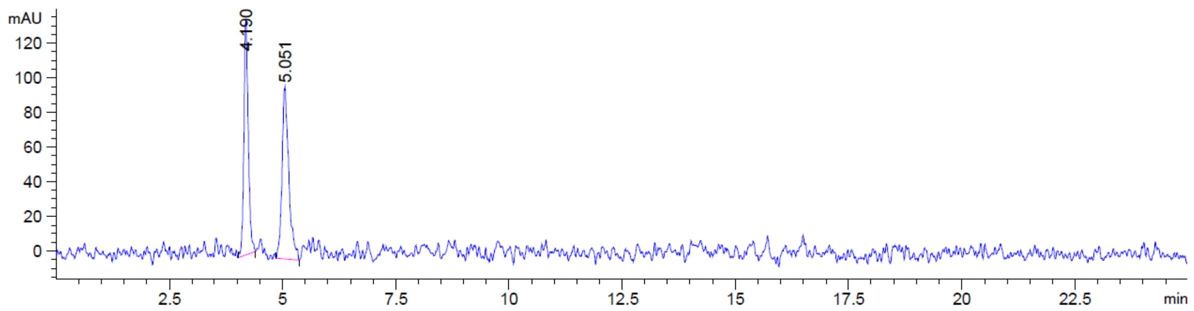

Peak #	RetTime [min]	Type	Width [min]	Area [mAU*s]	Height [mAU]	Area %
1	4.190	BB	0.1032	908.13794	133.83830	46.7970
2	5.051	BB	0.1453	1032.45264	99.33092	53.2030

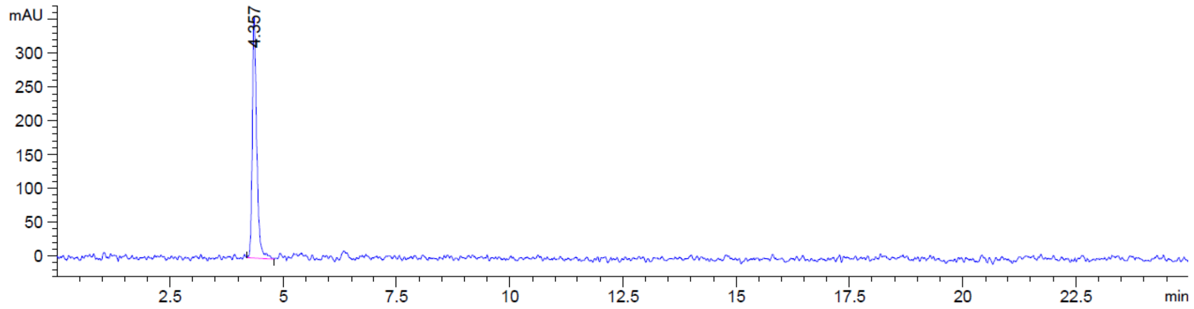

Peak #	RetTime [min]	Type	Width [min]	Area [mAU*s]	Height [mAU]	Area %
1	4.357	BB	0.1036	2420.86108	354.83142	100.0000

Synthesis of tBuBrettPhosAuCl according to procedures from Che[15] and Echavarren.[16]

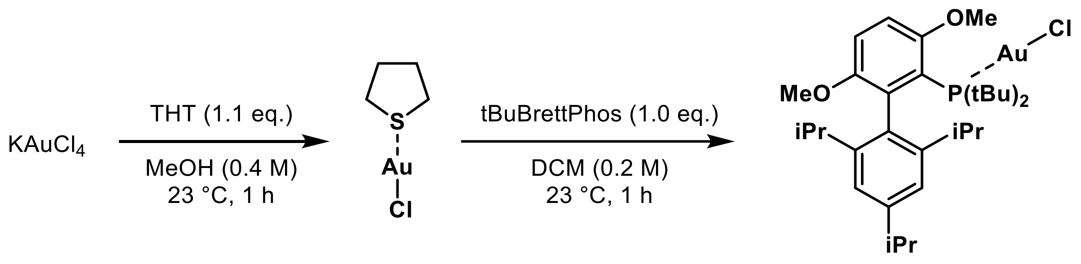

In the dark, a solution of potassium tetrachloroaurate (0.65 g, 1.7 mmol) in dry MeOH (4.0 mL) under N_2 was treated dropwise with tetrahydrothiophene (0.17 mL, 1.9 mmol), and then vigorously stirred at 23 °C for 1 h. The precipitate formed was then collected by filtration with a sintered glass funnel, and then washed with ice-cold methanol (3 × 3.0 mL) and dried on high vacuum to afford (THT)AuCl as a colorless solid. This material was immediately used without further purification.

In the dark, a solution of tBuBrettPhos (0.33 g, 0.68 mmol) in dry degassed DCM (3.0 mL) under N_2 was treated with (THT)AuCl (0.20 g, 0.68 mmol) and stirred for 23 °C for 1 h. The reaction mixture was then concentrated under reduced pressure and the crude residue purified by column chromatography (silica gel, DCM) to afford tBuBrettPhosAuCl as colorless crystals (0.44 g, 91% yield). The following characterization data were consistent with literature[16].

^{1}H NMR: (400 MHz, $CDCl_3$) δ 7.04 (s, 2H), 7.00 (d, *J* = 8.9 Hz, 1H), 6.93 (dd, *J* = 9.0, 3.3 Hz, 1H), 3.83 (s, 3H), 3.52 (s, 3H), 3.00 (hept, *J* = 7.0 Hz, 1H), 2.34 (hept, *J* = 6.9 Hz, 2H), 1.53 (s, 3H), 1.44 (s, 9H), 1.40 (s, 9H), 1.38 (s, 3H), 1.28 (d, *J* = 6.8 Hz, 6H), 0.86 (d, *J* = 6.6 Hz, 6H).

^{13}C NMR: (101 MHz, $CDCl_3$) δ 154.6, 153.4 (d, *J* = 11.6 Hz), 149.9, 146.2, 139.8 (d, *J* = 15.5 Hz), 130.9 (d, *J* = 7.4 Hz), 122.1, 119.5 (d, *J* = 37.4 Hz), 113.8, 109.1 (d, *J* = 5.3 Hz), 54.7, 54.2, 40.5 (d, *J* = 25.4 Hz), 34.1, 32.5 (d, *J* = 7.9 Hz), 31.0, 25.4, 24.5, 24.3.

^{31}P NMR: (162 MHz, CDCl3) δ 69.82.

IR (ATR) 2957, 2866, 1579, 1456, 1420, 1381, 1363, 1258, 1233, 1169, 1085, 1043, 1012, 731, 719.

HRMS (ESI + APCI) m/z: $[M - Cl]^+$ calculated for $[C_{31}H_{49}AuO_2P]^+$: 681.3130; found: 681.3115.

m.p. 283.2 °C (decomposition).

Rf 0.56 (silica gel, 2:1 pentane / EtOAc, UV / CAM).

Elemental Analysis calculated for AuCl•$C_{31}H_{49}O_2P$: C, 51.92; H, 6.89; N, 0.00. found: C, 51.81; H, 6.87; N, 0.04.

General procedure C for the gold-catalyzed synthesis of cyclopentadienyl esters according to a procedure by Zhang[14]

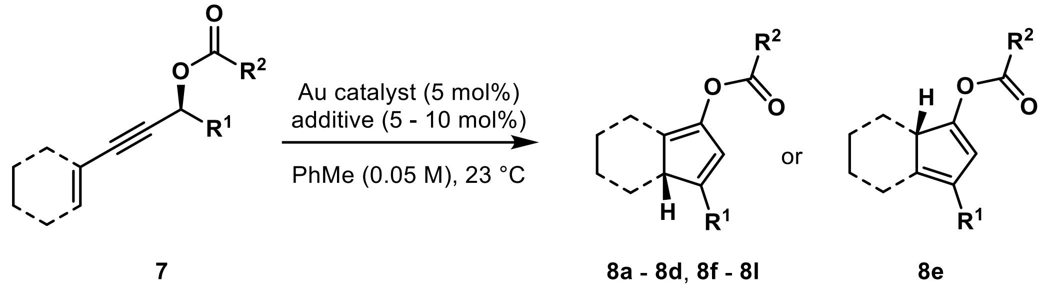

A dry mixture of gold catalyst (5 mol%) and additive (5 – 10 mol%) was dissolved in **dry** PhMe (0.1 M relative to propargylic ester) and stirred under N_2 for 5 min at 23 °C. The reaction mixture was then treated, in one portion, with a solution of the propargylic ester **7** in **dry** PhMe (0.1 M relative to propargylic ester), and further stirred until TLC indicated the reaction was complete. The suspension was concentrated *in vacuo*, and the crude residue subjected to column chromatography (silica gel) to afford **8**.

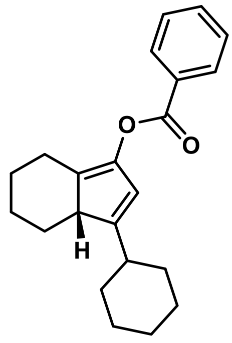

8a

Cyclopentadienyl ester **8a** was prepared via general procedure **C** with **7a** (0.10 g, 0.31 mmol, 97% ee), tBuBrettPhosAuCl (11 mg, 16 µmol, 5 mol%), NaBARF (14 mg, 16 µmol, 5 mol%), duration 3 h and 25:1 to 10:1 pentane / EtOAc gradient eluent, to give a colorless oil (71 mg, 71% yield, 95% ee). The following characterization data were consistent with literature[14].

^{1}H NMR (400 MHz, C_6D_6) δ 8.27 – 8.17 (m, 2H), 7.14 – 7.09 (m, 1H), 7.07 – 7.01 (m, 2H), 6.26 (d, *J* = 1.6 Hz, 1H), 2.83 – 2.72 (m, 1H), 2.55 (dd, *J* = 12.5, 5.9 Hz, 1H), 2.20 – 2.04 (m, 2H), 2.03 – 1.91 (m, 1H), 1.89 – 1.81 (m, 1H), 1.80 – 1.53 (m, 6H), 1.33 – 1.02 (m, 7H), 0.86 (qd, *J* = 12.5, 3.4 Hz, 1H).

^{13}C NMR (101 MHz, C_6D_6) δ 164.4, 156.0, 142.6, 133.2, 130.6, 130.4, 129.1, 128.7, 121.8, 49.3, 37.9, 35.2, 31.9, 31.5, 28.2, 27.0, 26.7, 26.7, 25.9, 24.6.

IR (ATR) 2922, 2849, 1733, 1448, 1294, 1246, 1203, 1175, 1165, 1141, 1091, 1065, 1024, 862.

HRMS (ESI/QTOF) m/z: $[M + H]^+$ calculated for $[C_{22}H_{27}O_2]^+$: 323.2006; found: 323.1998.

Rf 0.42 (silica gel, 20:1 pentane / EtOAc, UV / CAM).

HPLC CHIRALPAK® IA, 99:1 hexane / IPA, rate 1 mL / min, 25 min, 254 nm, t_R minor 5.84 min; t_R major 8.15 min.

Opt. Rot. $[\alpha]_D^{25}$ −38.67 (c = 1.00 in $CHCl_3$, 95% ee).

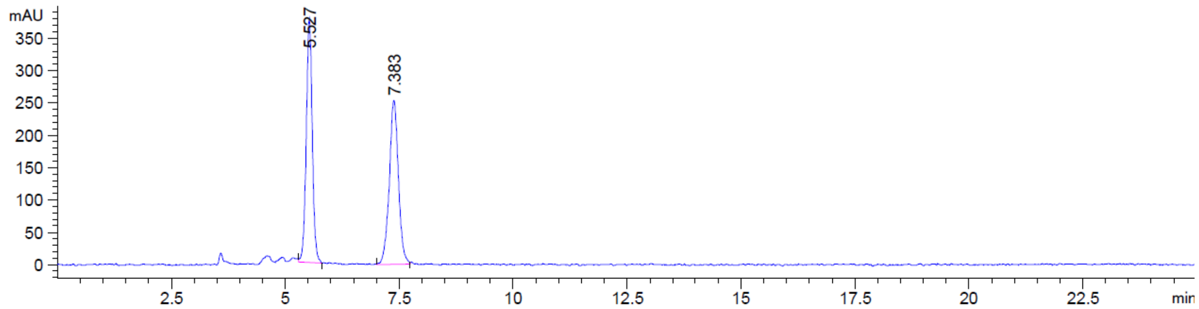

Peak #	RetTime [min]	Type	Width [min]	Area [mAU*s]	Height [mAU]	Area %
1	5.527	VB	0.1411	3459.05420	376.90262	49.3942
2	7.383	VV	0.2072	3543.89917	253.29207	50.6058

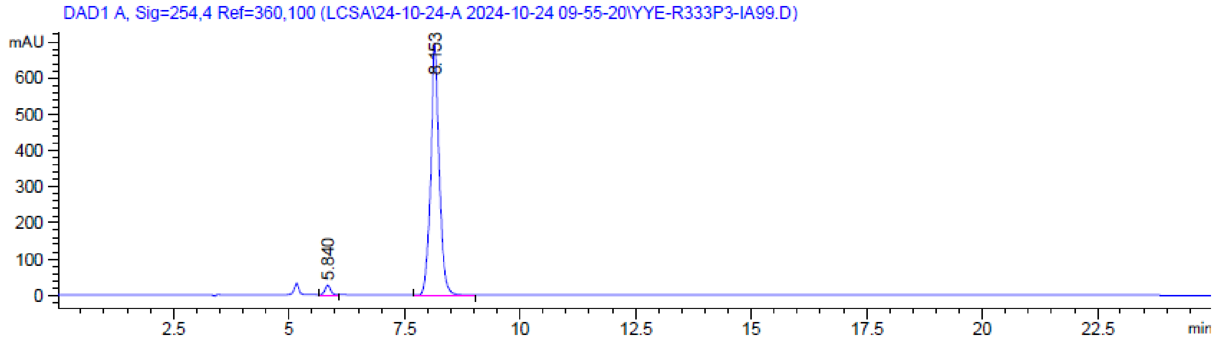

Peak #	RetTime [min]	Type	Width [min]	Area [mAU*s]	Height [mAU]	Area %
1	5.840	BV	0.1202	218.44521	27.05527	2.4022
2	8.153	BB	0.1838	8875.21875	691.28412	97.5978

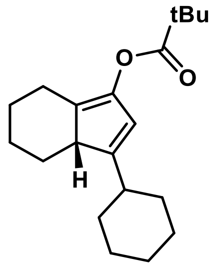

8b

Cyclopentadienyl ester **8b** was prepared via general procedure **C** with **7b** (50 mg, 0.17 mmol, >99% ee), tBuBrettPhosAuCl (11 mg, 16 µmol, 5 mol%), NaBARF (5.9 mg, 8.3 µmol, 5 mol%), duration 3 h and 100:1 pentane / EtOAc eluent, to give a colorless oil (35 mg, 70% yield, 85% ee).

^{1}H NMR (400 MHz, $CDCl_3$) δ 5.90 (s, 1H), 2.63 (dd, J = 12.5, 5.9 Hz, 1H), 2.58 – 2.51 (m, 1H), 2.32 – 2.24 (m, 1H), 2.23 – 2.14 (m, 1H), 1.99 (td, J = 13.3, 5.2 Hz, 1H), 1.86 – 1.64 (m, 7H), 1.43 – 1.34 (m, 1H), 1.32 – 1.27 (m, 12H), 1.19 – 1.09 (m, 3H), 0.88 – 0.79 (m, 1H).

^{13}C NMR (101 MHz, $CDCl_3$) δ 176.9, 156.4, 141.8, 128.8, 120.7, 49.1, 39.2, 37.7, 35.1, 31.7, 31.4, 27.9, 27.4, 26.8, 26.5, 25.7, 24.1.

IR (ATR) 2923, 2850, 1748, 1659, 1577, 1479, 1446, 1395, 1321, 1272, 1123, 1029, 886, 839.

HRMS (ESI + APCI) m/z: $[M + H]^+$ calculated for $[C_{20}H_{31}O_2]^+$: 303.2319; found: 303.2327.

Rf 0.22 (silica gel, 50:1 pentane / EtOAc, UV / CAM).

HPLC CHIRALPAK® IG, 99.5:0.5 hexane / IPA, rate 0.5 mL / min, 25 min, 254 nm, t_R minor 9.72 min; t_R major 11.76 min.

Opt. Rot. $[\alpha]_D^{25}$ −28.33 (c = 1.00 in $CHCl_3$, 85% ee).

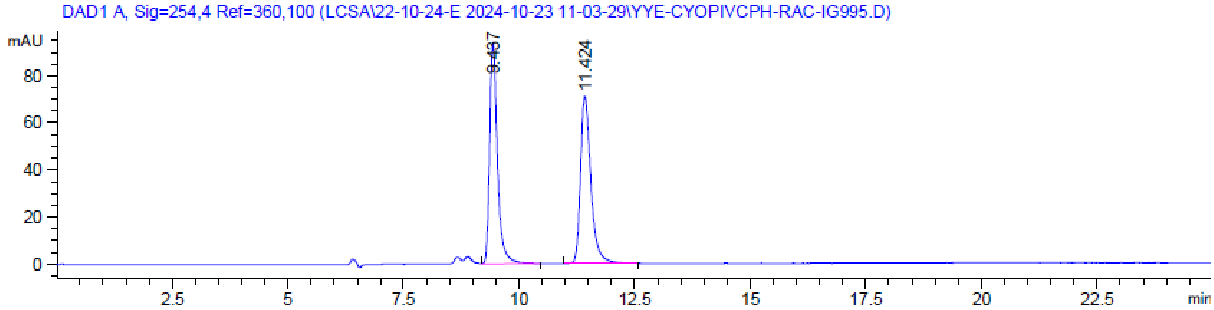

Peak #	RetTime [min]	Type	Width [min]	Area [mAU*s]	Height [mAU]	Area %
1	9.437	VB	0.1752	1090.92468	94.13297	49.6411
2	11.424	BB	0.2355	1106.70142	71.14319	50.3589

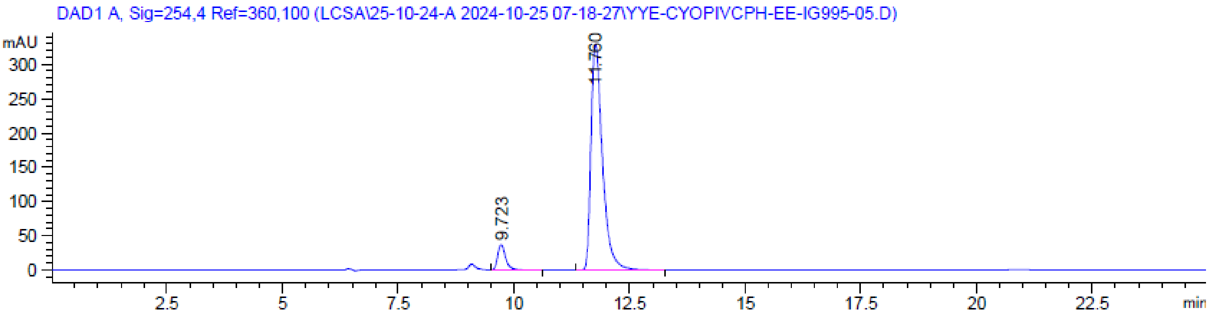

Peak #	RetTime [min]	Type	Width [min]	Area [mAU*s]	Height [mAU]	Area %
1	9.723	VB	0.1797	444.48447	37.11469	7.2641
2	11.760	BB	0.2609	5674.39697	329.50629	92.7359

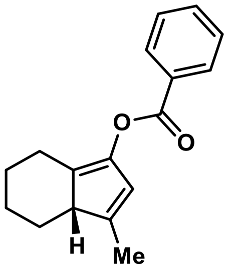

8c

Cyclopentadienyl ester **8c** was prepared via general procedure **C** with **7c** (36.2 mg, 0.143 mmol, 98% ee), tBuBrettPhosAuCl (5.1 mg, 7.1 µmol, 5 mol%), NaBARF (6.3 mg, 7.1 µmol, 5 mol%), duration 3 h and 20:1 pentane / EtOAc eluent, to give a colorless oil (28.1 mg, 77% yield, 97% ee). The following characterization data were consistent with literature[14].

^{1}H NMR (400 MHz, $CDCl_3$) δ 8.15 (d, *J* = 7.3 Hz, 2H), 7.60 (t, *J* = 7.4 Hz, 1H), 7.48 (t, *J* = 7.7 Hz, 2H), 6.07 (s, 1H), 2.68 (dd, *J* = 13.8, 4.8 Hz, 1H), 2.54 (dd, *J* = 12.5, 6.0 Hz, 1H), 2.36 – 2.31 (m, 1H), 2.04 (td, *J* = 13.2, 5.1 Hz, 1H), 1.97 (s, 3H), 1.92 – 1.82 (m, 2H), 1.48 – 1.36 (m, 1H), 1.26 – 1.17 (m, 1H), 0.89 (qd, *J* = 12.6, 3.2 Hz, 1H).

^{13}C NMR (101 MHz, $CDCl_3$) δ 164.9, 146.4, 141.7, 133.4, 130.2, 129.9, 129.8, 128.6, 128.6, 123.4, 51.5, 31.3, 28.1, 25.7, 24.2, 14.5.

IR (ATR) 2930, 2855, 1722, 1601, 1451, 1315, 1265, 1176, 1111, 1067, 1025, 990, 710.

HRMS (ESI/QTOF) m/z: $[M + Na]^+$ calculated for $[C_{17}H_{18}NaO_2]^+$: 277.1199; found: 277.1198.

Rf 0.54 (silica gel, 50:1 pentane / EtOAc, UV / CAM).

HPLC CHIRALPAK® IC, 99.5:0.5 hexane / IPA, rate 1 mL / min, 25 min, 254 nm, t_R minor 10.05 min; t_R major 10.99 min.

Opt. Rot. $[\alpha]_D^{20}$ +26.76 (c = 0.71 in $CHCl_3$, 96% ee).

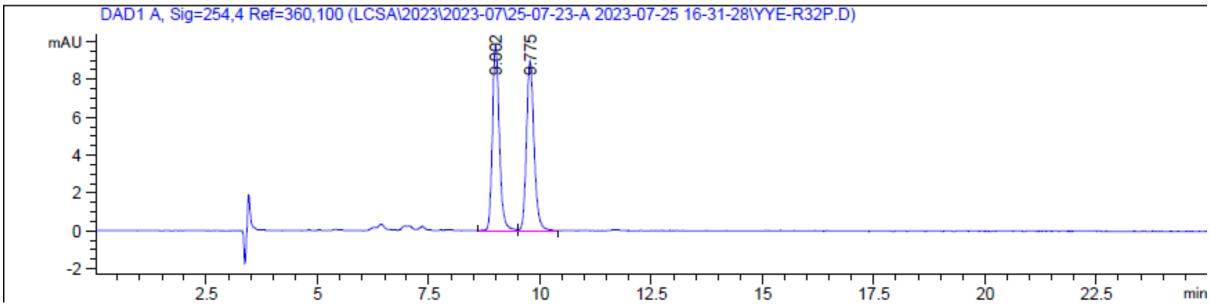

Signal 1: DAD1 A, Sig=254,4 Ref=360,100

Peak #	RetTime [min]	Type	Width [min]	Area [mAU*s]	Height [mAU]	Area %
1	9.002	BV	0.1669	106.71564	9.81366	49.9719
2	9.775	VB	0.1833	106.83575	8.94911	50.0281

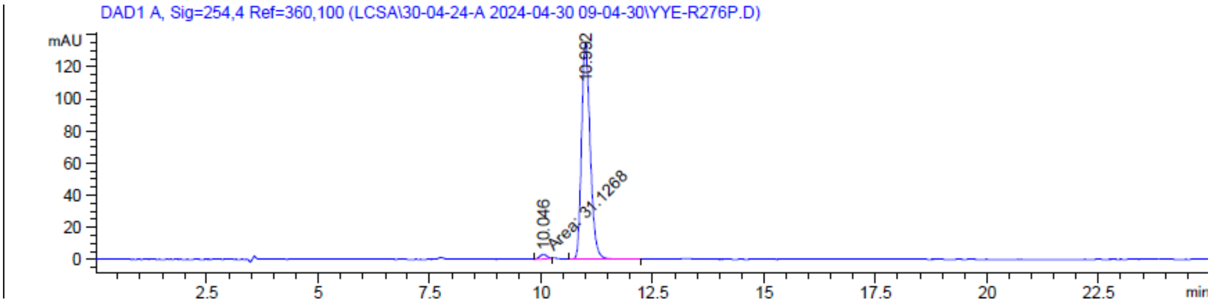

Signal 1: DAD1 A, Sig=254,4 Ref=360,100

Peak #	RetTime [min]	Type	Width [min]	Area [mAU*s]	Height [mAU]	Area %
1	10.046	MM T	0.1819	31.12683	2.85171	1.6776
2	10.992	BB	0.2079	1824.26465	134.81290	98.3224

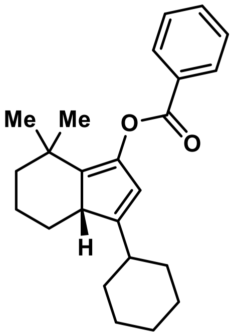

8d

Cyclopentadienyl ester **8d** was prepared via general procedure **C** with **7d** (50.0 mg, 0.143

mmol, 90% ee), tBuBrettPhosAuCl (5.1 mg, 7.1 µmol, 5 mol%), $AgSbF_6$ (2.5 mg, 7.1 µmol, 5 mol%), duration 3 h and 50:1 pentane / EtOAc eluent, to give a yellow oil (46.9 mg, 94% yield, 85% ee).

^{1}H NMR (400 MHz, $CDCl_3$) δ 8.15 – 8.11 (m, 2H), 7.60 (tt, *J* = 7.4, 1.9 Hz, 1H), 7.48 (t, *J* = 7.6 Hz, 2H), 5.94 (d, *J* = 0.8 Hz, 1H), 2.85 (dd, *J* = 13.8, 5.5 Hz, 1H), 2.31 – 2.16 (m, 2H), 1.88 (d, *J* = 9.6 Hz, 2H), 1.79 – 1.65 (m, 5H), 1.54 (d, *J* = 14.5 Hz, 1H), 1.30 (d, *J* = 4.4 Hz, 7H), 1.21 – 1.11 (m, 5H), 0.94 – 0.82 (m, 1H).

^{13}C NMR (101 MHz, $CDCl_3$) δ 165.1, 156.0, 141.1, 135.4, 133.4, 130.2, 130.0, 128.6, 121.2, 47.8, 43.3, 37.6, 34.9, 34.7, 31.5, 31.3, 29.3, 28.7, 26.8, 26.5, 22.6.

IR (ATR) 2922, 2851, 1735, 1642, 1450, 1362, 1342, 1311, 1288, 1260, 1191, 1175, 1144, 1126, 1108, 1082, 1065, 1026, 863, 706.

HRMS (ESI/QTOF) m/z: $[M + Na]^+$ calculated for $[C_{24}H_{30}NaO_2]^+$: 373.2138; found: 373.2128.

Rf 0.33 (silica gel, 100:1 pentane / EtOAc, UV).

HPLC CHIRALPAK® IG, 99.5:0.5 hexane / IPA, rate 1 mL / min, 25 min, 254 nm, t_R minor 9.86 min; t_R major 10.56 min.

Opt. Rot. $[\alpha]_D^{20}$ −6.33 (c = 1.00 in $CHCl_3$, 85% ee).

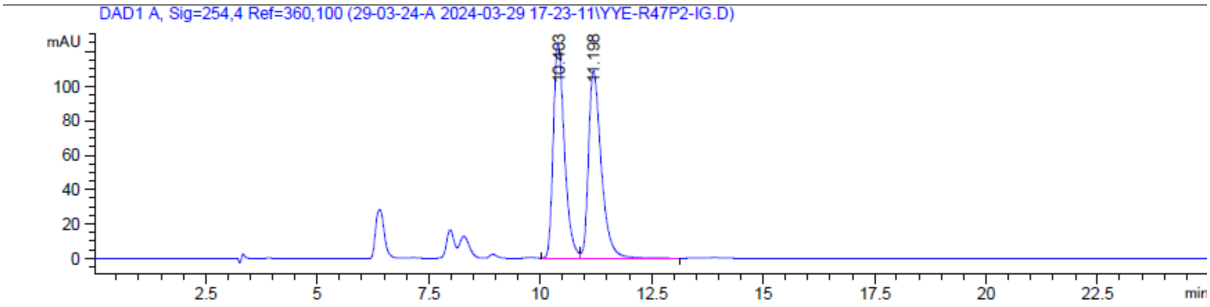

Signal 1: DAD1 A, Sig=254,4 Ref=360,100

Peak #	RetTime [min]	Type	Width [min]	Area [mAU*s]	Height [mAU]	Area %
1	10.403	BV	0.2669	2162.07739	124.29549	49.9475
2	11.198	VB	0.3004	2166.62524	108.75792	50.0525

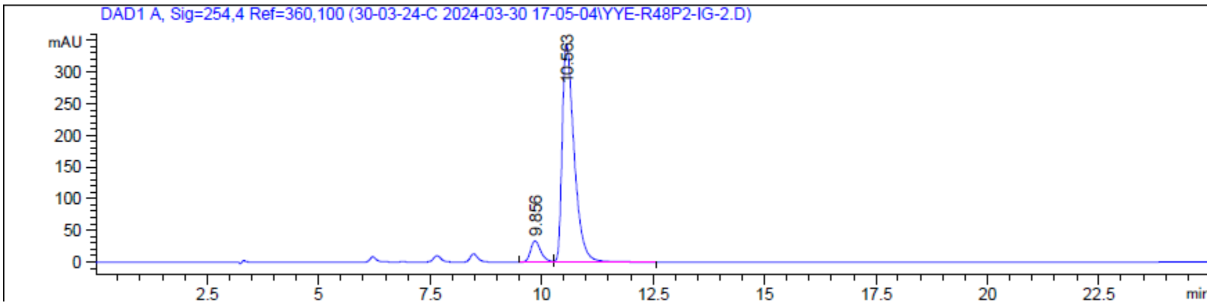

Signal 1: DAD1 A, Sig=254,4 Ref=360,100

Peak #	RetTime [min]	Type	Width [min]	Area [mAU*s]	Height [mAU]	Area %
1	9.856	BV	0.2442	534.56171	33.48947	7.7000
2	10.563	VB	0.2836	6407.81934	343.51398	92.3000

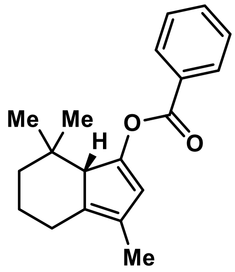

8e

Cyclopentadienyl ester **8e** was prepared via general procedure **C** with **7e** (0.10 g, 0.35 mmol, 98% ee), PPh_3AuCl (8.8 mg, 18 µmol, 5 mol%), $AgSbF_6$ (5.9 mg, 8.3 µmol, 5 mol%), duration 3 h and 25:1 pentane / EtOAc eluent, to give a colorless oil (51 mg, 51% yield, 89% ee).

^{1}H NMR (400 MHz, CD_2Cl_2) δ 8.10 (d, *J* = 7.0 Hz, 2H), 7.62 (t, *J* = 7.4 Hz, 1H), 7.50 (t, *J* = 7.6 Hz, 2H), 6.15 (s, 1H), 2.80 (s, 1H), 2.64 (d, *J* = 13.3 Hz, 1H), 2.00 (t, *J* = 12.6 Hz, 1H), 1.87 (s, 3H), 1.74 – 1.66 (m, 1H), 1.53 (s, 1H), 1.47 – 1.36 (m, 3H), 1.31 – 1.18 (m, 4H), 0.64 (s, 3H).

^{13}C NMR (101 MHz, CD_2Cl_2) δ 155.6, 133.6, 133.1, 130.4, 130.1, 129.3, 129.0, 119.8, 59.6, 41.3, 37.4, 31.1, 25.2, 23.9, 19.6, 12.6.

IR (ATR) 2929, 1735, 1452, 1259, 1177, 1136, 1081, 1070, 1057, 1024, 707.

HRMS (APCI/QTOF) m/z: $[M + H]^+$ calculated for $[C_{19}H_{23}O_2]^+$: 283.1693; found: 283.1683.

Rf 0.49 (silica gel, 50:1 pentane / EtOAc, UV).

HPLC CHIRALPAK® IF, 99:1 hexane / IPA, rate 1 mL / min, 25 min, 280 nm, t_R major 17.33 min; t_R minor 18.53 min.

Opt. Rot. $[\alpha]_D^{20}$ +18.40 (c = 0.48 in $CHCl_3$, 85% ee).

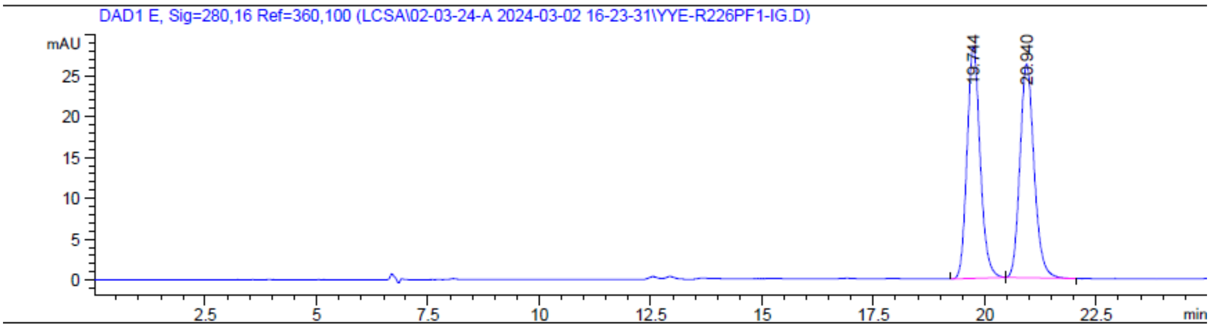

Signal 5: DAD1 E, Sig=280,16 Ref=360,100

Peak #	RetTime [min]	Type	Width [min]	Area [mAU*s]	Height [mAU]	Area %
1	19.744	BB	0.3134	577.57043	28.37958	49.9848
2	20.940	BB	0.3402	577.92206	26.11673	50.0152

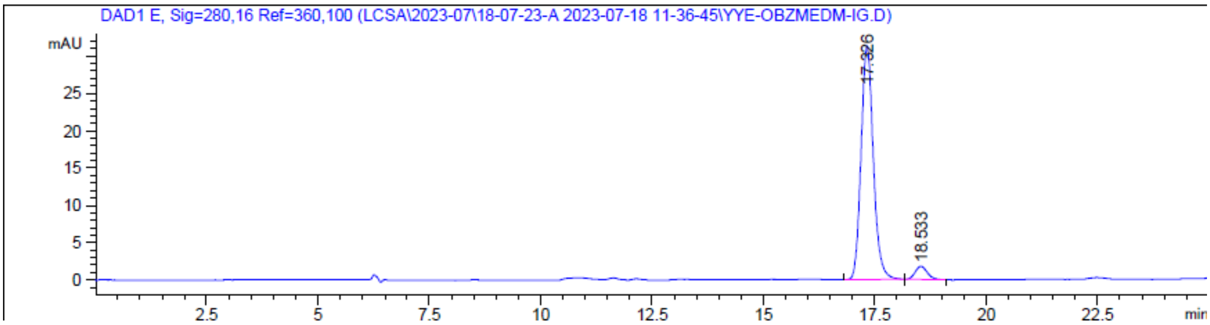

Signal 5: DAD1 E, Sig=280,16 Ref=360,100

Peak #	RetTime [min]	Type	Width [min]	Area [mAU*s]	Height [mAU]	Area %
1	17.326	BB	0.2926	597.00458	31.28607	94.6551
2	18.533	BB	0.2925	33.71143	1.76742	5.3449

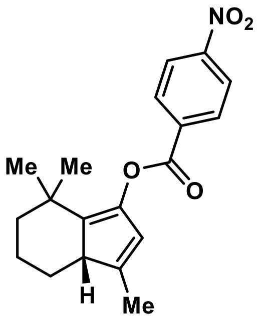

8f

Cyclopentadienyl ester **8f** was prepared via general procedure **C** with **7f** (65.4 mg, 0.200 mmol, 96% ee), PPh_3AuCl (4.9 mg, 10 µmol, 5 mol%), $AgSbF_6$ (6.9 mg, 20 µmol, 10 mol%), duration 3 h and 50:1 pentane / EtOAc eluent, to give a yellow oil (45.6 mg, 70% yield, 83% ee).

^{1}H NMR (400 MHz, CD_2Cl_2) δ 8.35 – 8.25 (m, 4H), 5.94 (s, 1H), 2.71 (dd, *J* = 12.9, 5.5 Hz, 1H),

2.35 – 2.25 (m, 1H), 1.96 (s, 3H), 1.77 – 1.65 (m, 2H), 1.57 (d, J = 12.9 Hz, 1H), 1.31 – 1.26 (m, 4H), 1.15 (s, 3H), 0.88 – 0.75 (m, 1H).

^{13}C NMR (101 MHz, CD_2Cl_2) δ 163.5, 151.2, 146.7, 140.9, 136.6, 135.7, 131.5, 124.1, 123.5, 50.6, 43.5, 35.0, 31.5, 29.2, 28.6, 22.7, 14.4.

IR (ATR) 2928, 1737, 1529, 1348, 1320, 1290, 1264, 1233, 1123, 1105, 1080, 101, 848, 717.

HRMS (APCI/QTOF) m/z: $[M + H]^+$ calculated for $[C_{19}H_{22}NO_4]^+$: 328.1543; found: 328.1525.

Rf 0.30 (silica gel, 50:1 pentane / EtOAc, UV).

HPLC CHIRALPAK® IF, 99:1 hexane / IPA, rate 1 mL / min, 25 min, 254 nm, t_R minor 11.01 min; t_R major 12.61 min.

Opt. Rot. $[\alpha]_D^{20}$ −28.95 (c = 0.38 in $CHCl_3$, 83% ee).

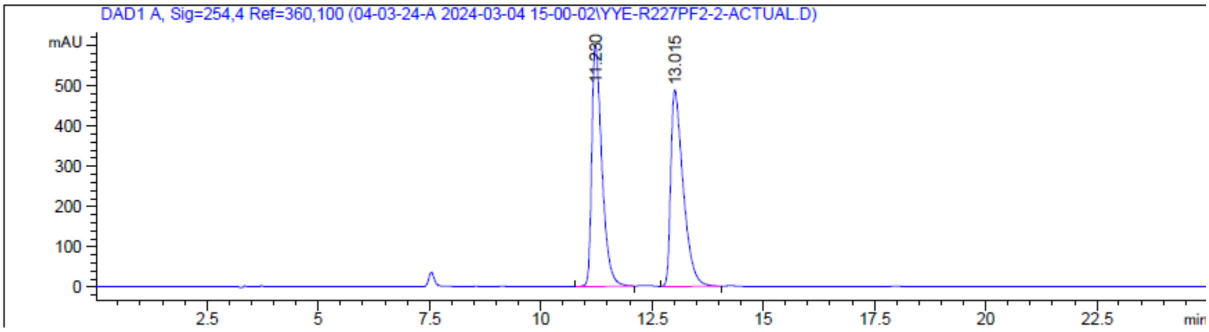

Signal 1: DAD1 A, Sig=254,4 Ref=360,100

Peak #	RetTime [min]	Type	Width [min]	Area [mAU*s]	Height [mAU]	Area %
1	11.230	BB	0.2391	9523.70996	600.34113	50.1211
2	13.015	BB	0.2903	9477.69727	488.68799	49.8789

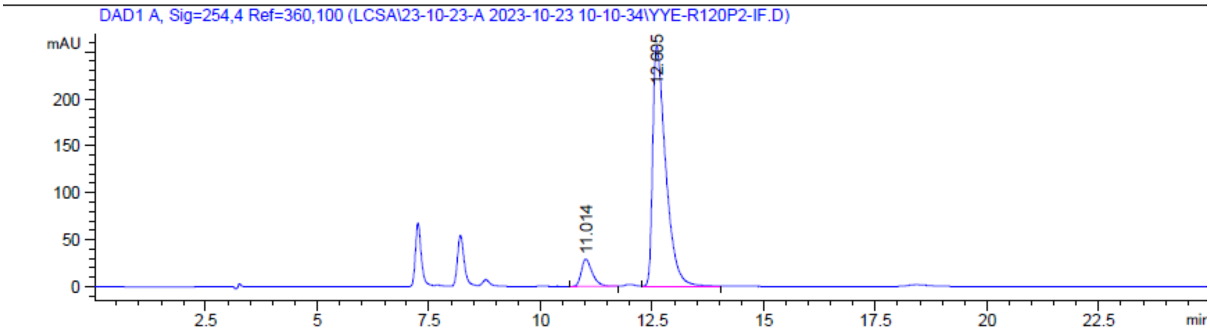

Signal 1: DAD1 A, Sig=254,4 Ref=360,100

Peak #	RetTime [min]	Type	Width [min]	Area [mAU*s]	Height [mAU]	Area %
1	11.014	BB	0.2544	492.28342	29.24591	8.6990
2	12.605	VB	0.2997	5166.81543	255.77617	91.3010

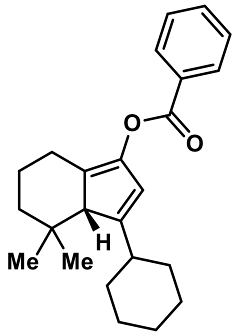

8g

Cyclopentadienyl ester **8g** was prepared via general procedure **C** with **7g** (35.4 mg, 0.101 mmol, 92% ee), tBuBrettPhosAuCl (3.6 mg, 5.0 µmol, 5 mol%), NaBARF (4.4 mg, 5.0 µmol, 5 mol%), duration 3 h and 50:1 pentane / EtOAc eluent, to give a colorless oil (23.9 mg, 68% yield, 89% ee).

^{1}H NMR (400 MHz, $CDCl_3$) δ 8.15 (d, *J* = 7.1 Hz, 2H), 7.60 (t, *J* = 7.4 Hz, 1H), 7.48 (t, *J* = 7.6 Hz, 2H), 6.04 (s, 1H), 2.70 – 2.56 (m, 2H), 2.30 – 2.22 (m, 1H), 2.04 – 1.88 (m, 3H), 1.82 – 1.61 (m, 4H), 1.47 – 1.24 (m, 10H), 1.05 (qd, *J* = 12.9, 3.5 Hz, 1H), 0.63 (s, 3H).

^{13}C NMR (101 MHz, $CDCl_3$) δ 165.1, 156.0, 141.1, 135.4, 133.4, 130.2, 130.0, 128.6, 121.2, 47.8, 43.3, 37.6, 34.9, 34.7, 31.5, 31.3, 29.3, 28.7, 26.8, 26.5, 22.6.

IR (ATR) 2927, 2851, 1737, 1450, 1278, 1262, 1238, 1228, 1177, 1127, 1089, 1070, 1057, 1025, 707.

HRMS (ESI/QTOF) m/z: $[M + Na]^+$ calculated for $[C_{24}H_{30}NaO_2]^+$: 373.2138; found: 373.2145.

Rf 0.49 (silica gel, 50:1 pentane / EtOAc, UV).

HPLC CHIRALPAK® IA, 99:1 hexane / IPA, rate 0.5 mL / min, 25 min, 280 nm, t_R minor 9.73 min; t_R major 10.70 min.

Opt. Rot. $[\alpha]_D^{20}$ −31.20 (c = 0.78 in $CHCl_3$, 89% ee).

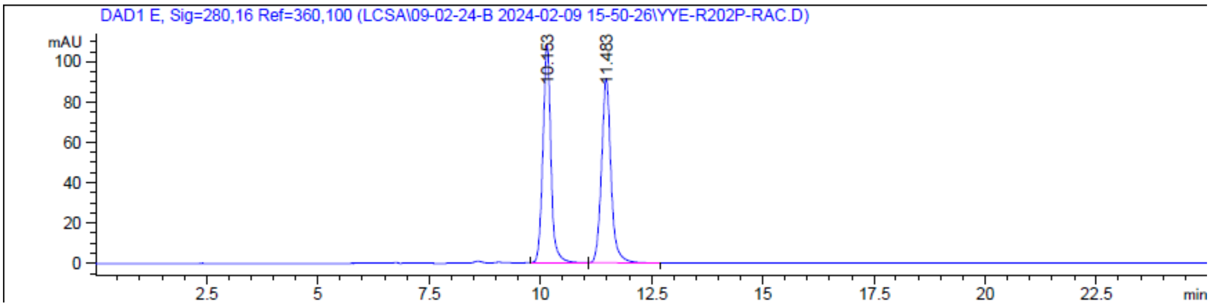

Signal 3: DAD1 E, Sig=280,16 Ref=360,100

Peak #	RetTime [min]	Type	Width [min]	Area [mAU*s]	Height [mAU]	Area %
1	10.153	BB	0.1898	1349.72021	107.99656	49.9032
2	11.483	BB	0.2211	1354.95679	91.31844	50.0968

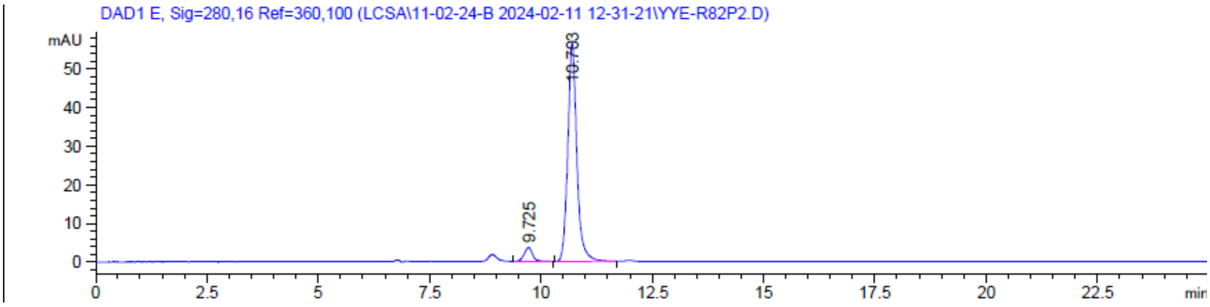

Signal 3: DAD1 E, Sig=280,16 Ref=360,100

Peak #	RetTime [min]	Type	Width [min]	Area [mAU*s]	Height [mAU]	Area %
1	9.725	BB	0.2014	48.03143	3.60522	5.6512
2	10.703	BB	0.2111	801.90222	56.67329	94.3488

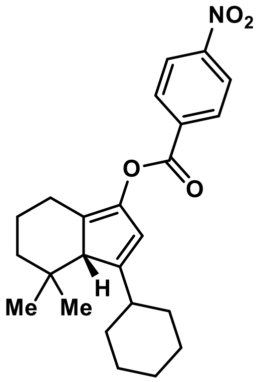

8h

Cyclopentadienyl ester **8h** was prepared via general procedure **C** with **7h** (22.9 mg, 0.0579 mmol, 93% ee), tBuBrettPhosAuCl (2.1 mg, 2.9 µmol, 5 mol%), $AgSbF_6$ (2.0 mg, 5.8 µmol, 10 mol%), duration 3 h and 50:1 pentane / EtOAc eluent, to give a yellow solid (20.2 mg, 88% yield, 87% ee). Yellow crystals (99% ee) suitable for X-ray analysis were obtained by cooling a saturated solution of **8h** (87% ee) in pentane to −20 °C.

^{1}H NMR (400 MHz, $CDCl_3$) δ 8.35 – 8.30 (m, 4H), 6.04 (s, 1H), 2.68 (s, 1H), 2.64 – 2.55 (m, 1H), 2.32 – 2.21 (m, 1H), 2.05 – 1.88 (m, 3H), 1.84 – 1.64 (m, 4H), 1.49 – 1.26 (m, 10H), 1.05 (qd, *J* = 12.8, 3.3 Hz, 1H), 0.62 (s, 3H).

^{13}C NMR (101 MHz, $CDCl_3$) δ 162.9, 156.1, 150.9, 142.2, 135.3, 131.3, 128.0, 123.8, 122.0, 57.4, 42.2, 40.0, 37.8, 36.1, 31.3, 31.2, 26.9, 26.7, 26.5, 24.0, 22.3, 19.3.

IR (ATR) 2927, 2851, 1741, 1529, 1449, 1348, 1319, 1280, 1263, 1238, 1228, 1128, 1090, 1015, 871, 847, 716.

HRMS (Sicrit plasma/LTQ-Orbitrap) m/z: $[M + H]^+$ calculated for $[C_{24}H_{30}NO_4]^+$: 396.2169; found: 396.2168.

m.p. 151.6-154.9 (99% ee).

Rf 0.42 (silica gel, 50:1 pentane / EtOAc, UV).

HPLC CHIRALPAK® IA, 98:2 hexane / IPA, rate 1 mL / min, 25 min, 254 nm, t_R minor 4.95 min; t_R major 11.35 min.

Opt. Rot. $[\alpha]_D^{20}$ −37.00 (c = 0.50 in $CHCl_3$, 99% ee).

Chromatogram

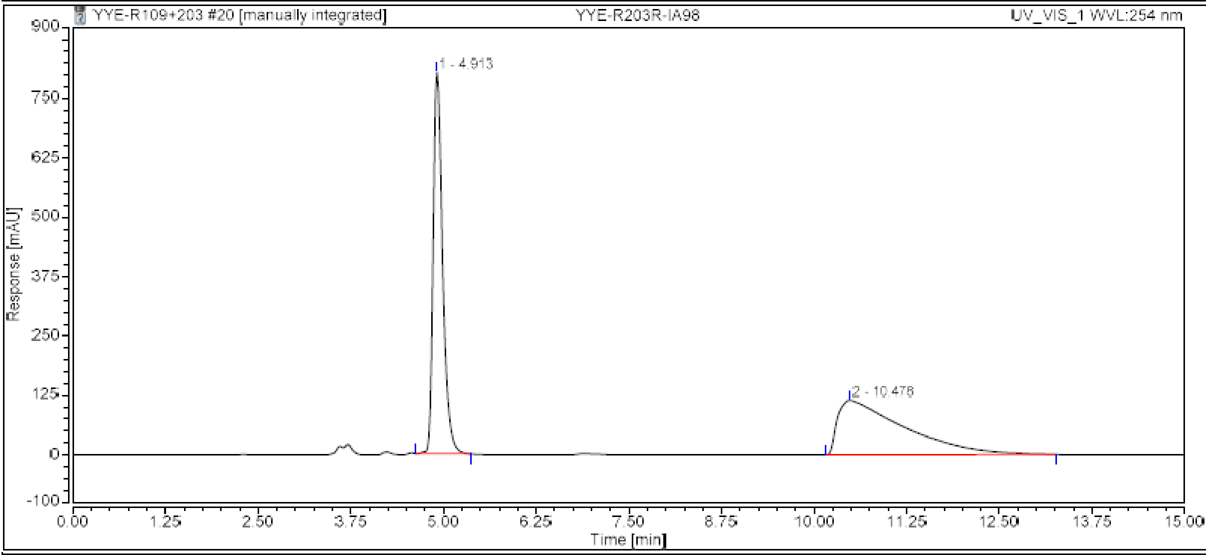

Integration Results

No.	Peak Name	Retention Time min	Area mAU*min	Height mAU	Relative Area %	Relative Height %	Amount n.a.
1		4.913	118.773	802.222	50.09	87.60	n.a.
2		10.478	118.345	113.591	49.91	12.40	n.a.
**Total:**			**237.118**	**915.813**	**100.00**	**100.00**	

Chromatogram

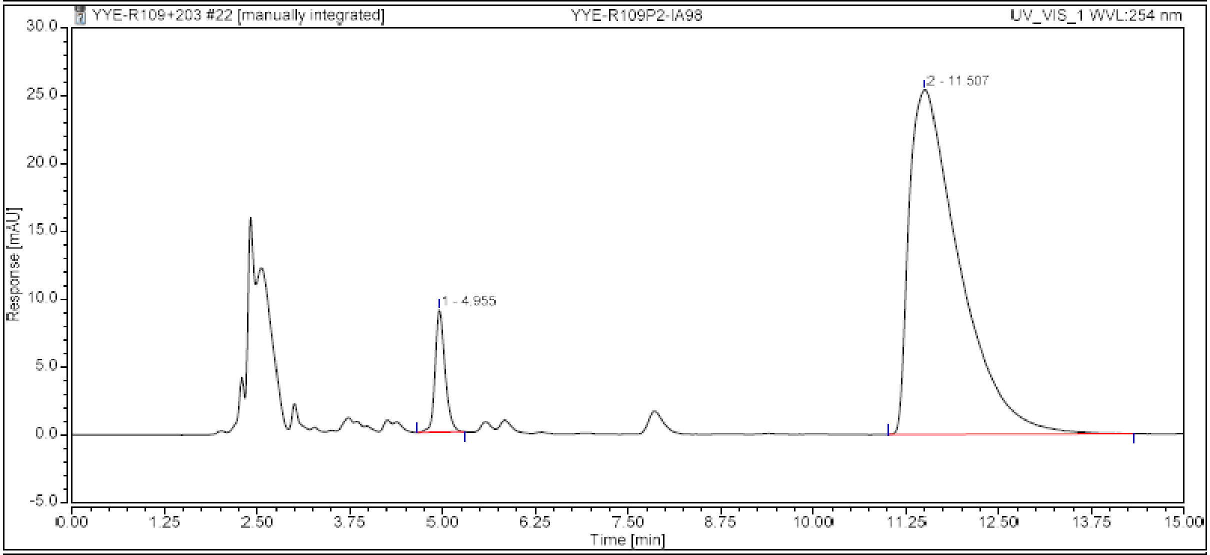

Integration Results

No.	Peak Name	Retention Time min	Area mAU*min	Height mAU	Relative Area %	Relative Height %	Amount n.a.
1		4.955	1.413	9.029	6.66	26.23	n.a.
2		11.507	19.800	25.392	93.34	73.77	n.a.
**Total:**			**21.212**	**34.421**	**100.00**	**100.00**	

After recrystallization:

Chromatogram

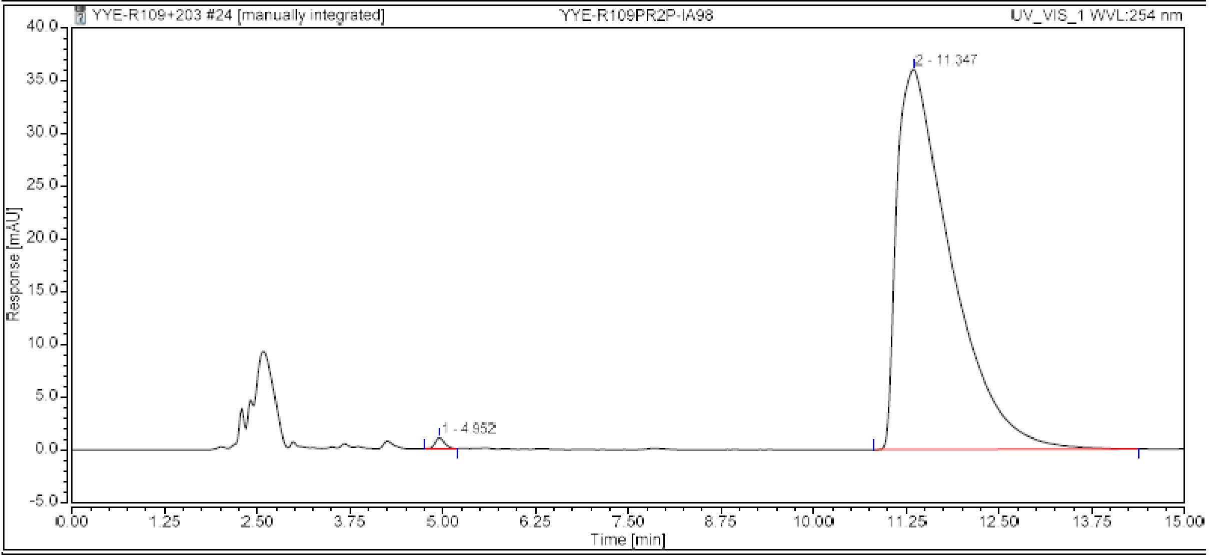

Integration Results

No.	Peak Name	Retention Time min	Area mAU*min	Height mAU	Relative Area %	Relative Height %	Amount n.a.
1		4.952	0.147	1.015	0.48	2.74	n.a.
2		11.347	30.370	36.025	99.52	97.26	n.a.
**Total:**			**30.518**	**37.040**	**100.00**	**100.00**	

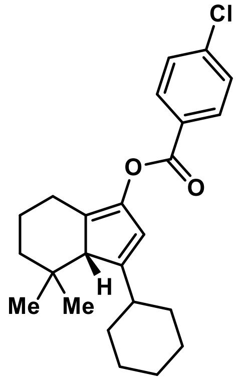

8i

Cyclopentadienyl ester **8i** was prepared via general procedure **C** with **7i** (23.2 mg, 60.3 µmol, 93% ee), tBuBrettPhosAuCl (2.2 mg, 3.0 µmol, 5 mol%), NaBARF (2.7 mg, 3.0 µmol, 5 mol%), duration 3 h and 50:1 pentane / EtOAc eluent, to give a colorless oil (18.5 mg, 80% yield, 90% ee).

^{1}H NMR (400 MHz, $CDCl_3$) δ 8.07 (d, J = 8.6 Hz, 2H), 7.45 (d, J = 8.6 Hz, 2H), 6.02 (s, 1H), 2.66 (s, 1H), 2.60 (d, J = 13.8 Hz, 1H), 2.26 (s, 1H), 2.05 – 1.88 (m, 3H), 1.86 – 1.59 (m, 4H), 1.48 – 1.18 (m, 10H), 1.05 (q, J = 12.6 Hz, 1H), 0.62 (s, 3H).

^{13}C NMR (101 MHz, $CDCl_3$) δ 164.0, 155.7, 142.4, 139.9, 131.6, 129.0, 128.4, 127.6, 122.4, 57.3, 42.3, 40.0, 37.7, 36.1, 31.3, 31.2, 26.9, 26.7, 26.6, 23.9, 22.4, 19.3.

IR (ATR) 2927, 2852, 1738, 1666, 1594, 1488, 1449, 1401, 1366, 1341, 1280, 1261, 1238, 1228, 1201, 1171, 1127, 1091, 1058, 1015, 848, 755, 682.

HRMS (ESI/QTOF) m/z: $[M + Na]^+$ calculated for $[C_{24}H_{29}ClNaO_2]^+$: 407.1748; found: 407.1745.

R*f* 0.43 (silica gel, 50:1 pentane / EtOAc, UV).

HPLC CHIRALPAK® IA, 99.5:0.5 hexane / IPA, rate 1 mL / min, 25 min, 230 nm, t_R minor 6.03 min; t_R major 7.05 min.

Opt. Rot. $[\alpha]_D^{20}$ −31.78 (c = 0.43 in $CHCl_3$, 90% ee).

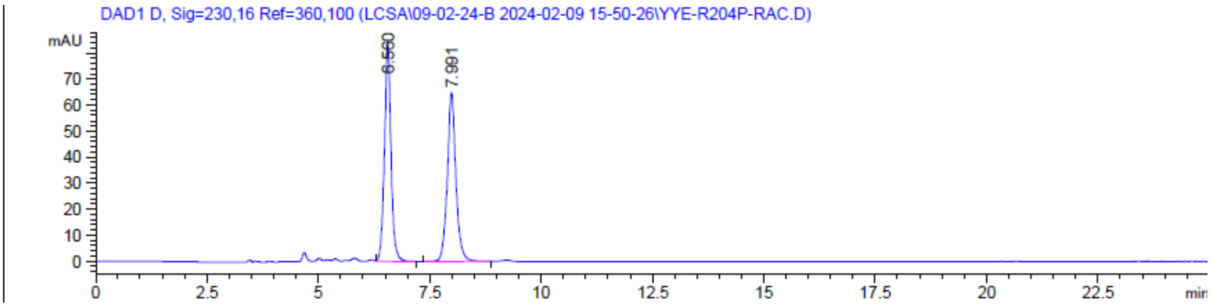

Signal 3: DAD1 D, Sig=230,16 Ref=360,100

Peak #	RetTime [min]	Type	Width [min]	Area [mAU*s]	Height [mAU]	Area %
1	6.560	VB	0.1520	864.59753	84.02641	49.8497
2	7.991	BB	0.1941	869.81183	64.99652	50.1503

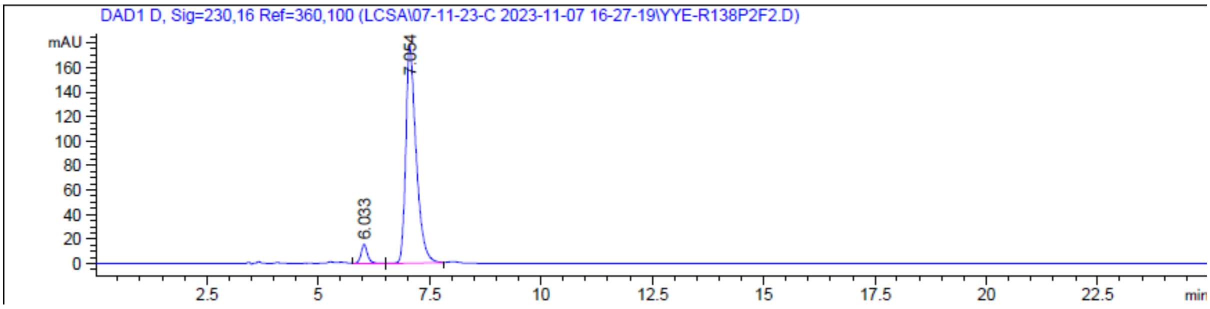

Signal 3: DAD1 D, Sig=230,16 Ref=360,100

Peak #	RetTime [min]	Type	Width [min]	Area [mAU*s]	Height [mAU]	Area %
1	6.033	BB	0.1491	157.90778	15.73812	5.2226
2	7.054	BB	0.2358	2865.62378	178.03340	94.7774

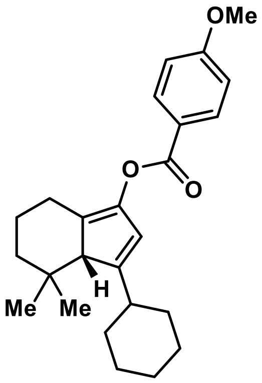

8j

Cyclopentadienyl ester **8j** was prepared via general procedure **C** with **7j** (19.7 mg, 0.0518 mmol, 93% ee), tBuBrettPhosAuCl (1.9 mg, 2.6 µmol, 5 mol%), NaBARF (2.3 mg, 2.6 µmol, 5 mol%), duration 6 h and 50:1 pentane / EtOAc eluent, to give a colorless oil (10.9 mg, 55% yield, 86% ee).

^{1}H NMR (400 MHz, $CDCl_3$) δ 8.09 (d, J = 8.9 Hz, 2H), 6.95 (d, J = 8.9 Hz, 2H), 6.03 (s, 1H), 3.88 (s, 3H), 2.68 – 2.55 (m, 2H), 2.30 – 2.18 (m, 1H), 2.03 – 1.88 (m, 3H), 1.80 – 1.63 (m, 4H), 1.45 – 1.24 (m, 10H), 1.10 – 0.99 (m, 1H), 0.62 (s, 3H).

^{13}C NMR (101 MHz, $CDCl_3$) δ 164.6, 163.8, 155.4, 142.7, 132.3, 132.3, 127.2, 122.8, 122.3, 113.9, 113.9, 57.2, 55.6, 42.3, 40.0, 37.7, 36.1, 31.3, 31.3, 26.9, 26.7, 26.6, 23.9, 22.4, 19.4.

IR (ATR) 2929, 2853, 1716, 1605, 1512, 1451, 1257, 1168, 1099, 1029, 848.

HRMS (ESI/QTOF) m/z: [M + Na]$^+$ calculated for [$C_{25}H_{32}NaO_3$]$^+$: 403.2244; found: 403.2233.

Rf 0.35 (silica gel, 50:1 pentane / EtOAc, UV).

HPLC CHIRALPAK® IA, 99:1 hexane / IPA, rate 1 mL / min, 25 min, 254 nm, t_R minor 8.62 min; t_R major 12.00 min.

Opt. Rot. $[\alpha]_D^{20}$ −28.99 (c = 0.23 in $CHCl_3$, 86% ee).

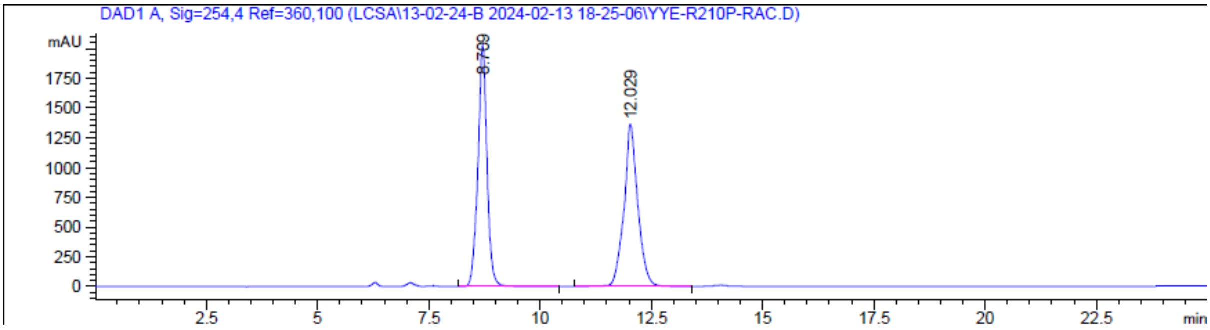

Signal 1: DAD1 A, Sig=254,4 Ref=360,100

Peak #	RetTime [min]	Type	Width [min]	Area [mAU*s]	Height [mAU]	Area %
1	8.709	BB	0.2083	2.89141e4	2028.05896	49.5377
2	12.029	BB	0.3038	2.94538e4	1364.58105	50.4623

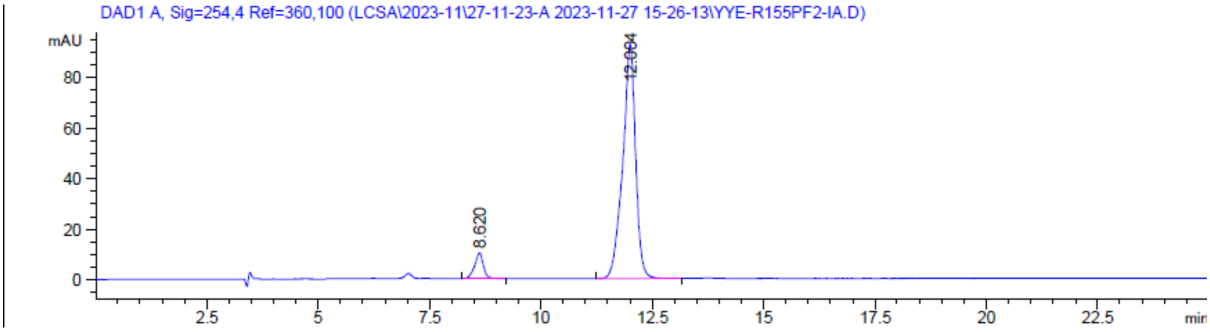

Signal 1: DAD1 A, Sig=254,4 Ref=360,100

Peak #	RetTime [min]	Type	Width [min]	Area [mAU*s]	Height [mAU]	Area %
1	8.620	BB	0.1993	138.94606	10.17811	6.9591
2	12.004	BB	0.2879	1857.65295	92.66076	93.0409

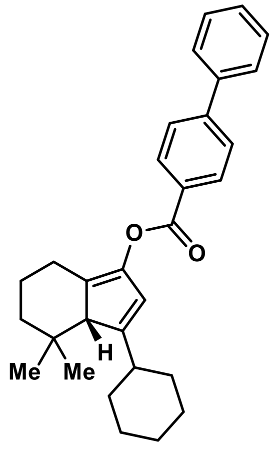

8k

Cyclopentadienyl ester **8k** was prepared via general procedure **C** with **7k** (78.9 mg, 0.185 mmol, 92% ee), tBuBrettPhosAuCl (6.6 mg, 9.2 µmol, 5 mol%), NaBARF (8.2 mg, 9.2 µmol, 5 mol%), duration 3 h and 50:1 pentane / EtOAc eluent, to give a colorless oil (61.7 mg, 78% yield, 89% ee).

^{1}H NMR (400 MHz, $CDCl_3$) δ 8.19 (d, *J* = 8.3 Hz, 2H), 7.74 (d, *J* = 8.4 Hz, 2H), 7.67 (d, *J* = 7.0 Hz, 2H), 7.49 (t, *J* = 7.5 Hz, 2H), 7.41 (t, *J* = 7.3 Hz, 1H), 6.05 (s, 1H), 2.69 (s, 1H), 2.67 – 2.61 (m, 1H), 2.30 (t, *J* = 10.1 Hz, 1H), 2.08 – 1.91 (m, 3H), 1.87 – 1.61 (m, 4H), 1.53 – 1.22 (m, 10H), 1.05 (qd, *J* = 12.9, 3.4 Hz, 1H), 0.63 (s, 3H).

^{13}C NMR (101 MHz, $CDCl_3$) δ 164.8, 155.9, 146.3, 142.9, 140.3, 130.8, 129.4, 129.0, 128.7, 127.8, 127.6, 127.5, 122.9, 57.5, 42.5, 40.3, 37.9, 36.4, 31.6, 31.3, 27.2, 27.0, 26.8, 24.1, 22.7, 19.3.

IR (ATR) 2926, 2851, 1733, 1608, 1449, 1278, 1262, 1238, 1228, 1189, 1179, 1127, 1085, 1008, 856, 745, 697.

HRMS (ESI/QTOF) m/z: $[M + Na]^+$ calculated for $[C_{30}H_{34}NaO_2]^+$: 449.2451; found: 449.2448.

R*f* 0.64 (silica gel, 50:1 pentane / EtOAc, UV).

HPLC CHIRALPAK® IA, 99.5:0.5 hexane / IPA, rate 1 mL / min, 15 min, 254 nm, t_R minor 10.43 min; t_R major 11.10 min.

Opt. Rot. $[\alpha]_D^{20}$ −18.44 (c = 0.94 in $CHCl_3$, 89% ee).

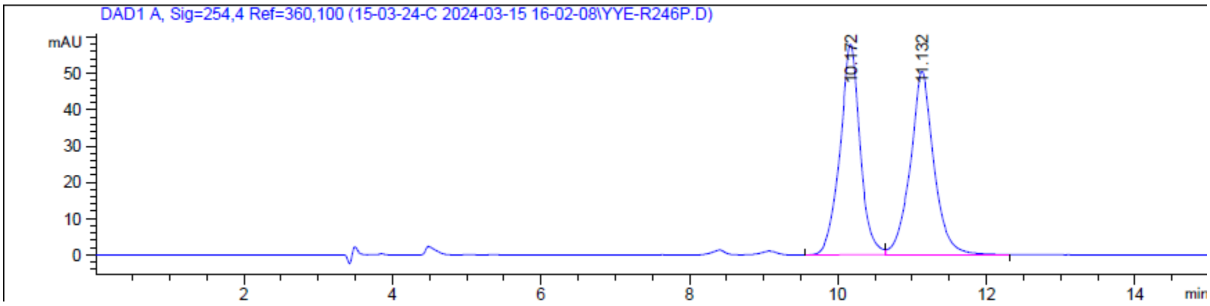

Signal 1: DAD1 A, Sig=254,4 Ref=360,100

Peak #	RetTime [min]	Type	Width [min]	Area [mAU*s]	Height [mAU]	Area %
1	10.172	BV	0.2741	1095.42578	58.05466	49.5614
2	11.132	VB	0.3151	1114.81348	50.58259	50.4386

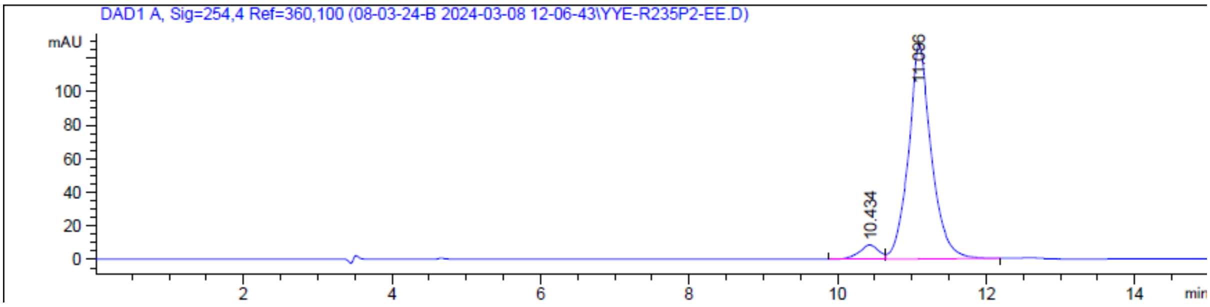

Signal 1: DAD1 A, Sig=254,4 Ref=360,100

Peak #	RetTime [min]	Type	Width [min]	Area [mAU*s]	Height [mAU]	Area %
1	10.434	BV	0.2696	155.01663	8.46847	5.3566
2	11.096	VB	0.3036	2738.92896	128.03534	94.6434

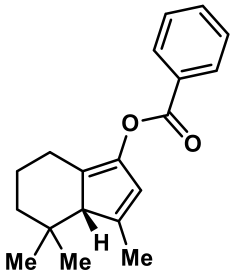

8l

Cyclopentadienyl ester **8l** was prepared via general procedure **C** with **7l** (28.2 mg, 0.100 mmol, >99% ee), tBuBrettPhosAuCl (3.6 mg, 5.0 µmol, 5 mol%), NaBARF (4.4 mg, 5.0 µmol, 5 mol%), duration 3 h and 50:1 pentane / EtOAc eluent, to give a yellow oil (26.6 mg, 94% yield, 98% ee).

^{1}H NMR (400 MHz, $CDCl_3$) δ 8.19 – 8.08 (m, 2H), 7.64 – 7.55 (m, 1H), 7.48 (t, *J* = 7.6 Hz, 2H),

6.07 (s, 1H), 2.63 (dd, J = 15.6, 2.9 Hz, 1H), 2.44 (s, 1H), 2.06 (s, 3H), 2.03 – 1.92 (m, 1H), 1.69 – 1.61 (m, 1H), 1.48 – 1.37 (m, 3H), 1.28 (s, 3H), 0.64 (s, 3H).

^{13}C NMR (101 MHz, $CDCl_3$) δ 164.9, 144.8, 142.4, 133.4, 130.2, 129.9, 128.6, 127.6, 126.0, 60.5, 41.9, 37.3, 31.2, 23.8, 22.7, 19.4, 18.3.

IR (ATR) 2934, 2865, 2840, 1736, 1663, 1601, 1452, 1365, 1340, 1314, 1264, 1236, 1176, 1128, 1087, 1071, 1056, 1024, 851, 707.

HRMS (Sicrit plasma/LTQ-Orbitrap) m/z: $[M + H]^+$ calculated for $[C_{19}H_{23}O_2]^+$: 283.1693; found: 283.1693.

Rf 0.49 (silica gel, 50:1 pentane / EtOAc, UV).

HPLC CHIRALPAK® IC, 99.5:0.5 hexane / IPA, rate 1 mL / min, 15 min, 254 nm, t_R minor 5.19 min; t_R major 5.74 min.

Opt. Rot. $[\alpha]_D^{20}$ +6.31 (c = 0.37 in $CHCl_3$, 98% ee).

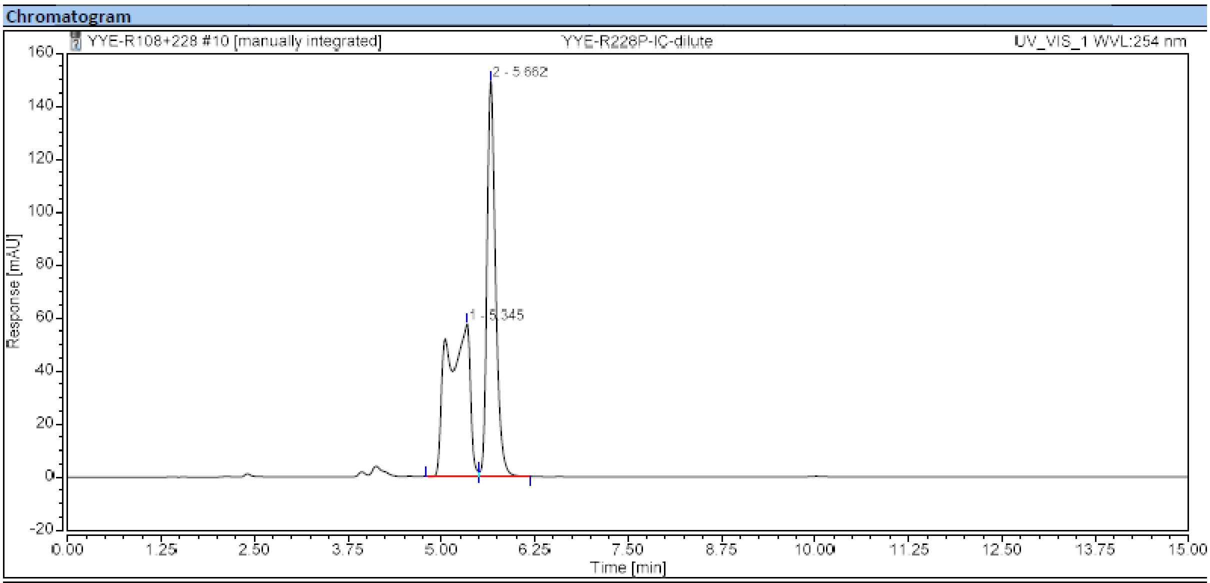

Integration Results

No.	Peak Name	Retention Time min	Area mAU*min	Height mAU	Relative Area %	Relative Height %	Amount n.a.
1		5.345	19.965	57.498	49.90	27.82	n.a.
2		5.662	20.048	149.186	50.10	72.18	n.a.
**Total:**			**40.013**	**206.684**	**100.00**	**100.00**	

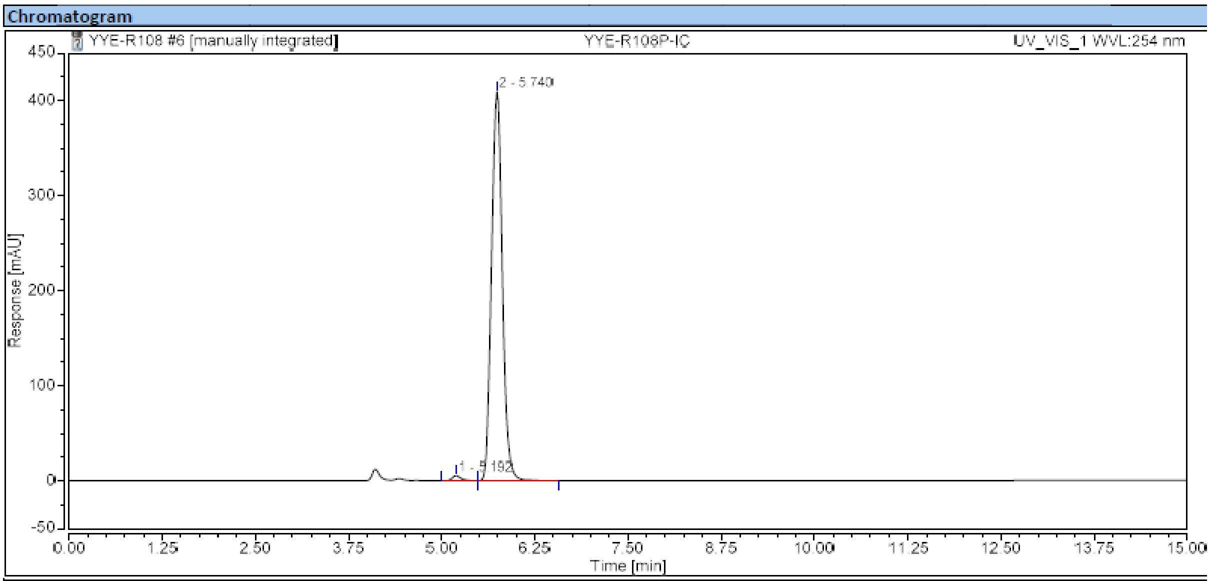

Integration Results

No.	Peak Name	Retention Time min	Area mAU*min	Height mAU	Relative Area %	Relative Height %	Amount n.a.
1		5.192	0.675	5.151	0.97	1.24	n.a.
2		5.740	68.644	409.144	99.03	98.76	n.a.
**Total:**			**69.319**	**414.295**	**100.00**	**100.00**	

General procedure D for the gold-catalyzed synthesis of cyclopentenones according to a procedure by Zhang[14]

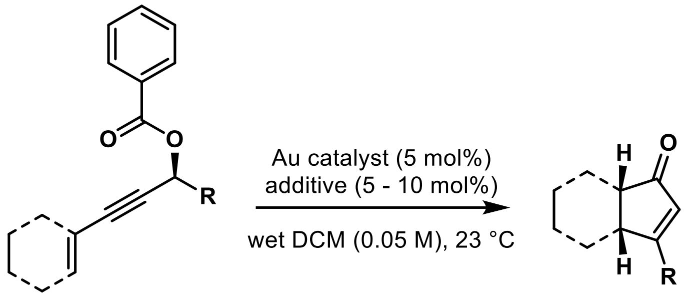

7a, **7c**, **7d** **9**

A dry mixture of gold catalyst (4 - 5 mol%) and additive (4 - 10 mol%) was dissolved in **dry** DCM (0.1 M relative to propargylic ester) and stirred under N_2 for 5 min at 23 °C. The reaction mixture was then treated, in one portion, with a solution of the propargylic ester **7** in **wet** DCM (0.1 M relative to propargylic ester), and further stirred until TLC indicated the reaction was complete. The suspension was concentrated *in vacuo*, and the crude residue subjected to column chromatography (silica gel) to afford cyclopentenone **9**.

Note: the wet DCM was prepared by vigorously shaking equivolume water and DCM in a separatory funnel (c.a. 1400 ppm of water in DCM).

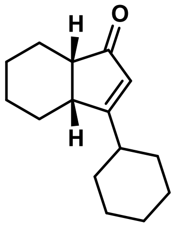

9a

Cyclopentenone **9a** was prepared via general procedure **D** with **7a** (8.4 g, 26 mmol, 96% ee), tBuBrettPhosAuCl (0.75 g, 1.0 mmol, 4 mol%), NaBARF (0.92 g, 1.0 mmol, 4 mol%), duration 3 h and 25:1 to 10:1 pentane / EtOAc gradient eluent, to give a colorless oil (4.9 g, 87% yield, 95% ee). The observed ee of the product ranged from 88-95%.

^{1}H NMR (400 MHz, $CDCl_3$) δ 5.84 (t, *J* = 1.2 Hz, 1H), 2.98 – 2.88 (m, 1H), 2.46 – 2.38 (m, 1H), 2.29 – 2.19 (m, 1H), 2.05 – 1.90 (m, 3H), 1.88 – 1.77 (m, 3H), 1.76 – 1.64 (m, 2H), 1.57 – 1.48 (m, 2H), 1.38 – 1.18 (m, 6H), 1.15 – 1.01 (m, 2H).

^{13}C NMR (101 MHz, $CDCl_3$) δ 211.6, 190.0, 125.6, 46.7, 41.8, 39.6, 32.0, 31.1, 28.6, 26.6, 26.2, 26.0, 22.7, 22.1, 21.7.

IR (ATR) 2923, 2851, 1696, 1600, 1447, 1318, 1268, 1226, 1182, 1169, 1128, 1113, 879, 856.

HRMS (ESI + APCI) m/z: $[M + H]^+$ calculated for $[C_{15}H_{23}O]^+$: 219.1743; found: 219.1738.

Rf 0.61 (silica gel, 10:1 pentane / EtOAc, UV / CAM).

HPLC CHIRALPAK® IC, 90:10 hexane / IPA, rate 1 mL / min, 25 min, 230 nm, t_R minor 18.25 min; t_R major 21.33 min.

Opt. Rot. $[\alpha]_D^{20}$ −8.17 (c = 1.0 in $CHCl_3$, 95% ee).

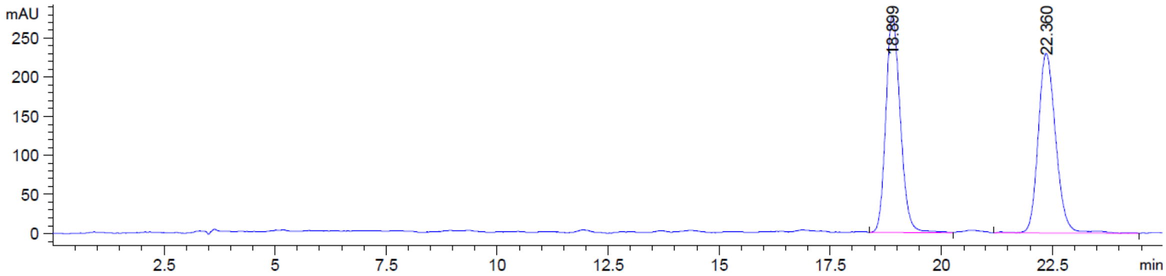

Peak #	RetTime [min]	Type	Width [min]	Area [mAU*s]	Height [mAU]	Area %
1	18.899	VV	0.3522	6277.17285	277.14319	49.7452
2	22.360	BB	0.4257	6341.48291	229.95959	50.2548

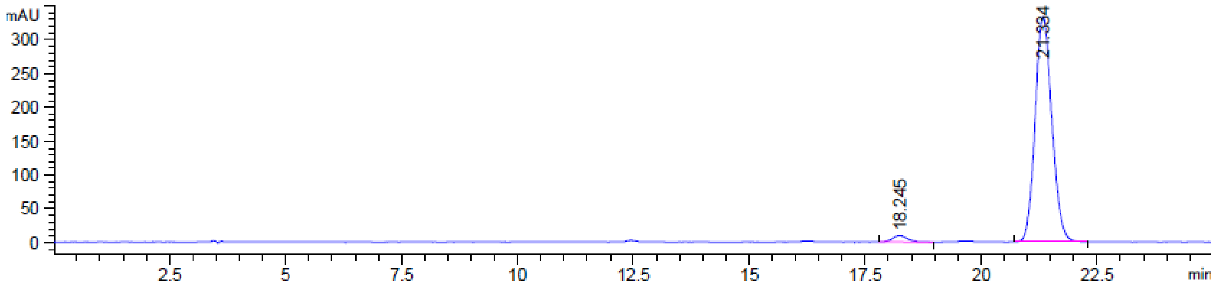

Peak #	RetTime [min]	Type	Width [min]	Area [mAU*s]	Height [mAU]	Area %
1	18.245	BB	0.3429	222.79558	10.11728	2.5895
2	21.334	BB	0.3893	8380.89355	333.34485	97.4105

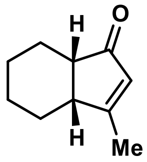

9b

Cyclopentenone **9b** was prepared via general procedure **D** with **7c** (0.83 g, 3.3 mmol, 98% ee), tBuBrettPhosAuCl (0.12 g, 0.16 mmol, 5 mol%), NaBARF (0.14 g, 0.16 mmol, 5 mol%), duration 3 h and 50:1 DCM / EtOAc eluent, to give a colorless oil (0.26 g, 54% yield, 96% ee). The following characterization data were consistent with literature[17].

^{1}H NMR (400 MHz, $CDCl_3$) δ 5.88 (t, J = 1.4 Hz, 1H), 2.80 – 2.72 (m, 1H), 2.46 (q, J = 6.3 Hz, 1H), 2.08 (s, 3H), 2.05 – 1.97 (m, 1H), 1.96 – 1.87 (m, 1H), 1.70 – 1.61 (m, 1H), 1.58 – 1.47 (m, 2H), 1.39 – 1.22 (m, 2H), 1.17 – 1.07 (m, 1H).

^{13}C NMR (101 MHz, $CDCl_3$) δ 211.4, 181.4, 128.7, 46.9 44.2, 27.8, 22.7, 21.7, 21.5, 17.6.

IR (ATR) 2932, 2857, 1689, 1611, 1583, 1449, 1434, 1376, 1314, 1235, 1173, 1111, 923, 909, 877, 780.

HRMS (ESI + APCI) m/z: $[M + H]^+$ calculated for $[C_{10}H_{15}O]^+$: 151.1117; found: 151.1122.

Rf 0.35 (silica gel, 10:1 pentane / EtOAc, UV / CAM).

HPLC CHIRALPAK® IC, 80:20 hexane / IPA, rate 1 mL / min, 25 min, 210 nm, t_R minor 13.90 min; t_R major 14.91 min.

Opt. Rot. $[\alpha]_D^{21}$ 60.17 (c = 1.0 in $CHCl_3$, 96% ee).

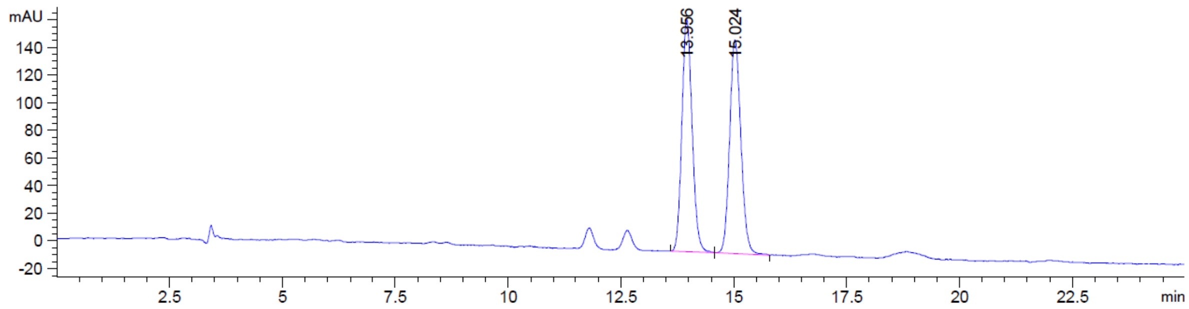

Peak #	RetTime [min]	Type	Width [min]	Area [mAU*s]	Height [mAU]	Area %
1	13.956	BB	0.2442	2650.78003	167.89461	49.8248
2	15.024	BB	0.2677	2669.42212	154.43750	50.1752

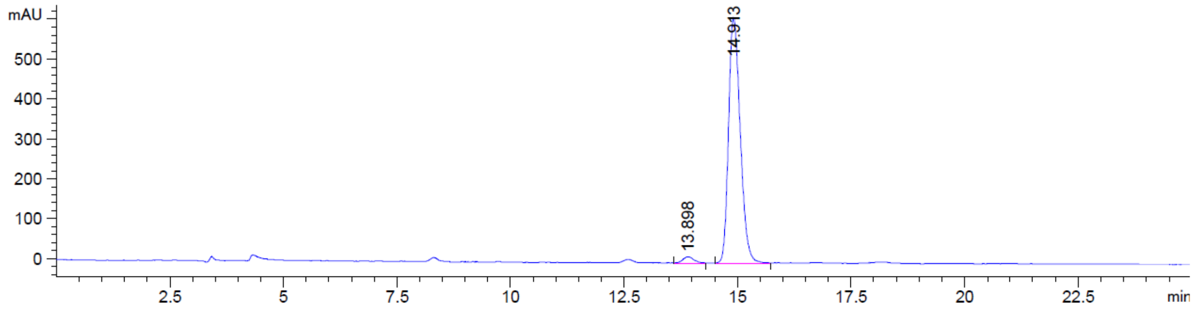

Peak #	RetTime [min]	Type	Width [min]	Area [mAU*s]	Height [mAU]	Area %
1	13.898	BV	0.2112	257.42294	15.38709	2.2972
2	14.913	BV	0.2777	1.09484e4	615.07684	97.7028

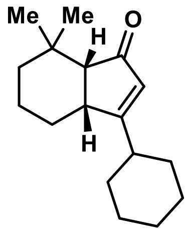

9c

Cyclopentenone **9c** was prepared via general procedure **D** with **7d** (50.2 mg, 0.143 mmol, 90% ee), PPh_3AuCl (3.5 mg, 7.1 µmol, 5 mol%), $AgSbF_6$ (4.9 mg, 0.143 mmol, 10 mol%), duration 3 h and 20:1 to 10:1 pentane / EtOAc gradient eluent. This gave a 6.7 : 1 mixture of **9c** and isomer **9c'** as a colorless oil (24.2 mg, 60% yield of **9c**, 84% ee of **9c**).

Note: The ratio of isomers ranged from 3.7 : 1 to 6.7 : 1 across different batches.

^{1}H NMR (400 MHz, $CDCl_3$) δ 5.69 (s, 1H), 2.91 – 2.82 (m, 1H), 2.20 – 2.14 (m, 1H), 2.10 (d, *J* = 6.2 Hz, 1H), 1.95 – 1.83 (m, 2H), 1.78 – 1.70 (m, 3H), 1.65 (d, *J* = 11.6 Hz, 1H), 1.52 – 1.45 (m,

2H), 1.31 – 1.12 (m, 8H), 1.03 (d, J = 2.4 Hz, 6H).

^{13}C NMR (101 MHz, $CDCl_3$) δ 210.1, 188.4, 126.7, 56.5, 41.3, 39.5, 37.1, 32.9, 31.9, 31.5, 31.1, 28.3, 26.6, 26.2, 26.2, 26.1, 19.7.

IR (ATR) 2926, 2853, 1698, 1607, 1449, 1385, 1268.

HRMS (ESI/QTOF) m/z: $[M + H]^+$ calculated for $[C_{17}H_{27}O]^+$: 247.2056; found: 247.2058.

Rf 0.30 (silica gel, 50:1 pentane / EtOAc, UV / CAM).

HPLC CHIRALPAK® IG, 90:10 hexane / IPA, rate 1 mL / min, 25 min, 210 nm, t_R major 7.41 min; t_R minor 9.27 min.

Opt. Rot. $[\alpha]_D^{20}$ +10.17 (c = 1.00 in $CHCl_3$, 84% ee).

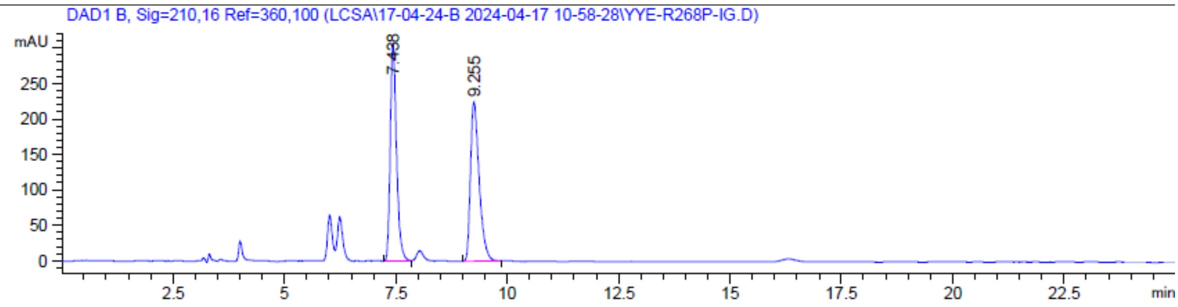

Signal 2: DAD1 B, Sig=210,16 Ref=360,100

Peak #	RetTime [min]	Type	Width [min]	Area [mAU*s]	Height [mAU]	Area %
1	7.438	BV	0.1488	3013.90161	306.21219	49.6262
2	9.255	BB	0.2089	3059.31006	224.63719	50.3738

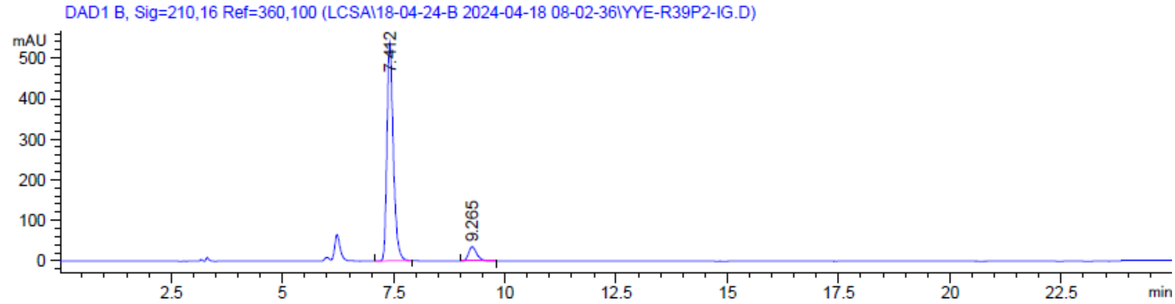

Signal 2: DAD1 B, Sig=210,16 Ref=360,100

Peak #	RetTime [min]	Type	Width [min]	Area [mAU*s]	Height [mAU]	Area %
1	7.412	BB	0.1499	5358.69482	539.62549	91.8790
2	9.265	BB	0.1975	473.64581	36.00647	8.1210

Synthesis of dihydroindanone isomer 9c’

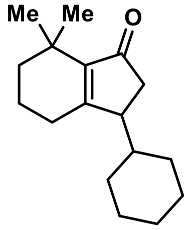

9c'

A suspension of cyclopentadienyl ester *rac*-**8d** (39.1 mg, 0.111 mmol) and NaOH (26.7 mg, 0.669 mmol, 6.0 eq.) in EtOH (1.12 mL, 0.1 M) under N_2 was stirred at 60 °C for 1 h. The reaction mixture was neutralized with HCl solution (1 M) and extracted with pentane (3 × 5 mL). The combined organic layers were dried (Na_2SO_4), filtered and concentrated *in vacuo*. No presence of desired product *rac*-**9c** was observed by NMR analysis of the crude mixture. The crude residue was subjected to column chromatography (silica gel, 20:1 pentane / EtOAc) to afford *rac*-**9c'** as a colorless oil (22.2 mg, 81%, racemic).

^{1}H NMR (400 MHz, $CDCl_3$) δ 2.68 – 2.56 (m, 1H), 2.32 – 2.07 (m, 4H), 1.79 – 1.59 (m, 7H), 1.51 – 1.36 (m, 2H), 1.26 – 1.13 (m, 9H), 1.13 – 1.05 (m, 2H), 0.86 – 0.78 (m, 1H).

^{13}C NMR (101 MHz, $CDCl_3$) δ 208.3, 174.4, 145.7, 46.5, 39.6, 38.5, 37.5, 32.2, 31.4, 27.7, 27.5, 26.9, 26.6, 26.4, 26.3, 25.5, 19.2.

IR (ATR) 2921, 2851, 1692, 1633, 1449, 1419, 1383, 1358, 1320, 1267, 1215, 990, 888.

HRMS (ESI/QTOF) m/z: $[M + H]^+$ calculated for $[C_{17}H_{27}O]^+$: 247.2056; found: 247.2061.

R*f* 0.35 (silica gel, 50:1 pentane / EtOAc, UV / CAM).

HPLC CHIRALPAK® IG, 90:10 hexane / IPA, rate 1 mL / min, 25 min, 210 nm, t_R 6.00 min; t_R 6.22 min.

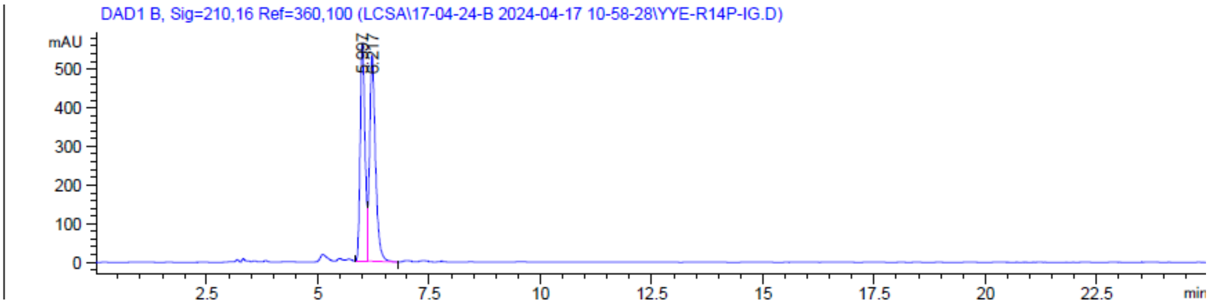

```
Signal 2: DAD1 B, Sig=210,16 Ref=360,100

Peak RetTime Type  Width     Area      Height      Area
  #   [min]        [min]   [mAU*s]     [mAU]        %
----|-------|----|-------|----------|----------|--------
   1    5.997 BV    0.1158 4238.65039  563.07629  46.4115
   2    6.217 VB    0.1371 4894.11133  533.36377  53.5885
```

General procedure E for the synthesis of aryl substituted cyclopentadienes

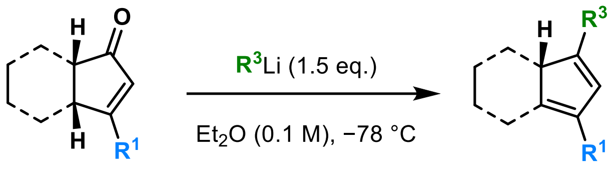

A solution of aryllithium (1.5 eq.) in dry Et_2O (0.2 M relative to cyclopentenone) under N_2 at −78 °C was treated dropwise with a solution of cyclopentenone (1.0 eq.) in dry Et_2O (0.2 M), and stirred for a further 3 h at −78 °C. When specified, 2-nosyl chloride (1.5 eq.) was then added to induce dehydration of the addition intermediate and warmed to 23 °C over 10 min. The reaction mixture was quenched with NH_4Cl solution (equivolume to Et_2O, sat. aq.) at −78 °C then warmed to 23 °C. The separated aqueous layer was extracted with Et_2O (3 × equivolume to reaction mixture Et_2O) and the combined organic layers were washed with brine (equivolume to reaction mixture Et_2O), dried ($MgSO_4$), filtered and concentrated *in vacuo*. The resulting crude product was purified by column chromatography (silica gel) to afford cyclopentadiene **1.**

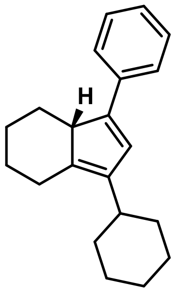

1a

Cyclopentadiene **1a** was prepared via general procedure **E** with **9a** (0.16 g, 0.70 mmol, 95% ee), phenyllithium (0.60 mL, 1.8 M in hexane, 1.1 mmol) and pentane eluent to give a white solid (0.13 g, 64% yield, 95% ee). Colorless crystals suitable for X-ray analysis were obtained by slow evaporation of a solution of *rac*-**1a** in pentane.

¹H NMR (400 MHz, $CDCl_3$) δ 7.43 – 7.38 (m, 2H), 7.34 – 7.27 (m, 2H), 7.17 – 7.11 (m, 1H), 6.79 (s, 1H), 3.08 (dd, *J* = 12.6, 5.7 Hz, 1H), 2.81 – 2.74 (m, 1H), 2.51 – 2.33 (m, 2H), 2.15 (td, *J* = 13.3, 5.0 Hz, 1H), 2.09 – 1.93 (m, 1H), 1.86 – 1.61 (m, 6H), 1.56 – 1.07 (m, 7H), 0.80 (qd, *J* = 12.9, 3.4 Hz, 1H).

¹³C NMR (101 MHz, $CDCl_3$) δ 148.1, 143.5, 140.8, 136.0, 128.6, 127.6, 125.9, 125.5, 52.1, 36.3, 34.0, 33.5, 33.0, 29.9, 26.8, 26.5, 26.4, 26.2.

IR (ATR) 2922, 2849, 1597, 1490, 1444, 860, 754, 693.

HRMS (ESI + APCI) m/z: $[M + H]^+$ calculated for $[C_{21}H_{27}]^+$: 279.2107; found: 279.2108.

m.p. 99.8 – 102.3 °C.

Rf 0.61 (silica gel, 50:1 pentane / EtOAc, UV / CAM).

HPLC CHIRALPAK® IG, 99.5:0.5 hexane / IPA, rate 1 mL / min, 25 min, 320 nm, t_R minor 4.36 min; t_R major 5.22 min.

Opt. Rot. $[\alpha]_D^{20}$ −12.33 (c = 1.00 in $CHCl_3$, 95% ee).

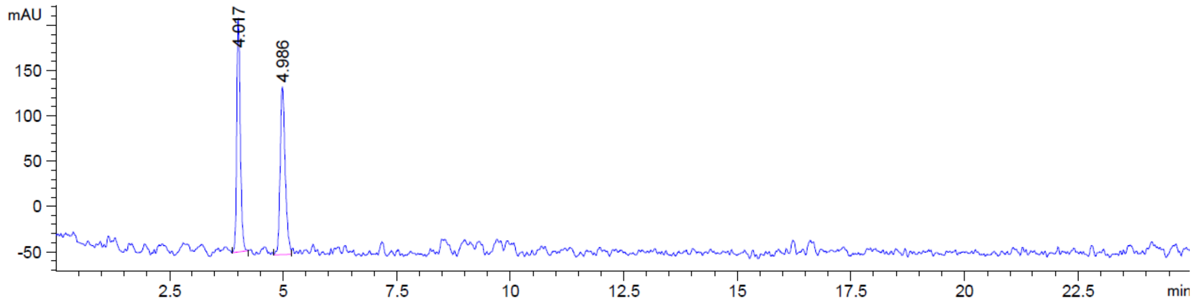

Peak #	RetTime [min]	Type	Width [min]	Area [mAU*s]	Height [mAU]	Area %
1	4.017	BB	0.0836	1409.23413	258.57825	49.5199
2	4.986	BV	0.1163	1436.55774	185.67166	50.4801

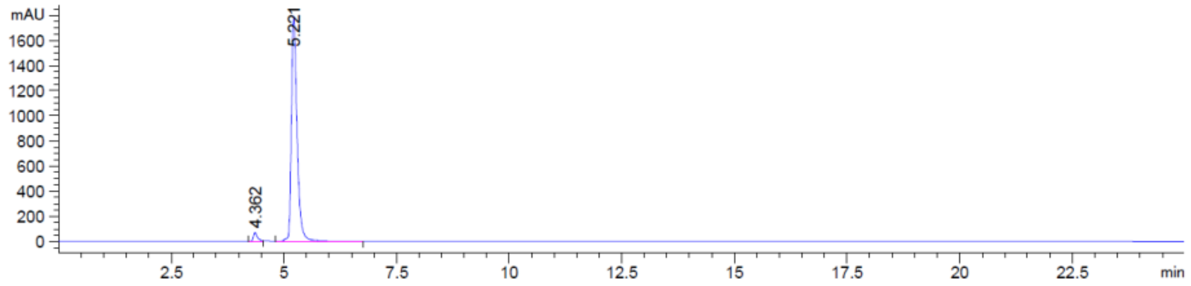

Peak #	RetTime [min]	Type	Width [min]	Area [mAU*s]	Height [mAU]	Area %
1	4.362	BV	0.0939	445.69498	70.34380	2.7367
2	5.221	VB	0.1352	1.58402e4	1791.56494	97.2633

Preparation of (4-(trifluoromethyl)phenyl)lithium and (4-methoxyphenyl)lithium

A solution of 1-bromo-4-(trifluoromethyl)benzene (1.0 equiv.) or 1-bromo-4-methoxybenzene (1.0 equiv.) in dry Et_2O (0.2 M) under N_2 at -78 °C was treated dropwise with *n*BuLi (2.5 M, 1.0 eq.) or *t*BuLi (1.6M, 1.0 eq.) respectively. The reaction mixture was left to stir at -78 °C for 1 h, then warmed to 0 °C for 30 min. The presumed aryllithium solutions were cooled to -78 °C again and were immediately used to prepare **1b** or **1c**.

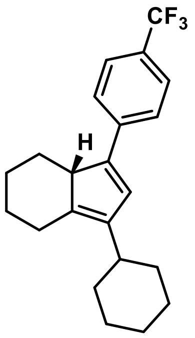

1b

Cyclopentadiene **1b** was prepared via general procedure **E** with **9a** (0.20 g, 0.92 mmol, 96% ee), 4-(trifluoromethyl)phenyllithium (1.4 mmol, 1.5 eq.) and 50:1 pentane / EtOAc eluent to give a colorless oil (0.22 g, 68% yield, 93% ee).

^{1}H NMR (400 MHz, C_6D_6) δ 7.45 (d, *J* = 8.3 Hz, 2H), 7.16 – 7.14 (m, 2H), 6.83 (s, 1H), 2.84 – 2.68 (m, 2H), 2.55 – 2.43 (m, 1H), 2.17 – 2.10 (m, 1H), 2.03 (td, *J* = 13.3, 5.0 Hz, 1H), 1.82 – 1.68 (m, 5H), 1.56 (dt, *J* = 13.5, 3.7 Hz, 1H), 1.48 – 1.37 (m, 3H), 1.35 – 1.19 (m, 4H), 1.13 – 0.97 (m, 1H), 0.72 (qd, *J* = 12.8, 3.3 Hz, 1H).

^{13}C NMR (101 MHz, C_6D_6) δ 147.1, 145.2, 141.2, 139.5, 130.3, 125.9 – 125.7 (m, C_{Ar}, CF_3), 52.2, 36.6, 33.7, 33.6, 33.3, 29.9, 27.0, 26.6, 26.5, 26.1.

^{19}F NMR (376 MHz, C_6D_6) δ −61.78.

IR (ATR) 2924, 2851, 1612, 1446, 1322, 1188, 1162, 1120, 1068, 1014, 839, 827.

HRMS (ESI + APCI) m/z: $[M + H]^+$ calculated for $[C_{22}H_{26}F_3]^+$: 347.1981; found: 347.1962.

Rf 0.65 (silica gel, pentane, UV / CAM).

HPLC CHIRALPAK® IB, 99.5:0.5 hexane / IPA, rate 0.2 mL / min, 30 min, 254 nm, t_R major 11.74 min; t_R minor 13.68 min.

Opt. Rot. $[\alpha]_D^{20}$ +9.00 (c = 1.00 in $CHCl_3$, 93% ee).

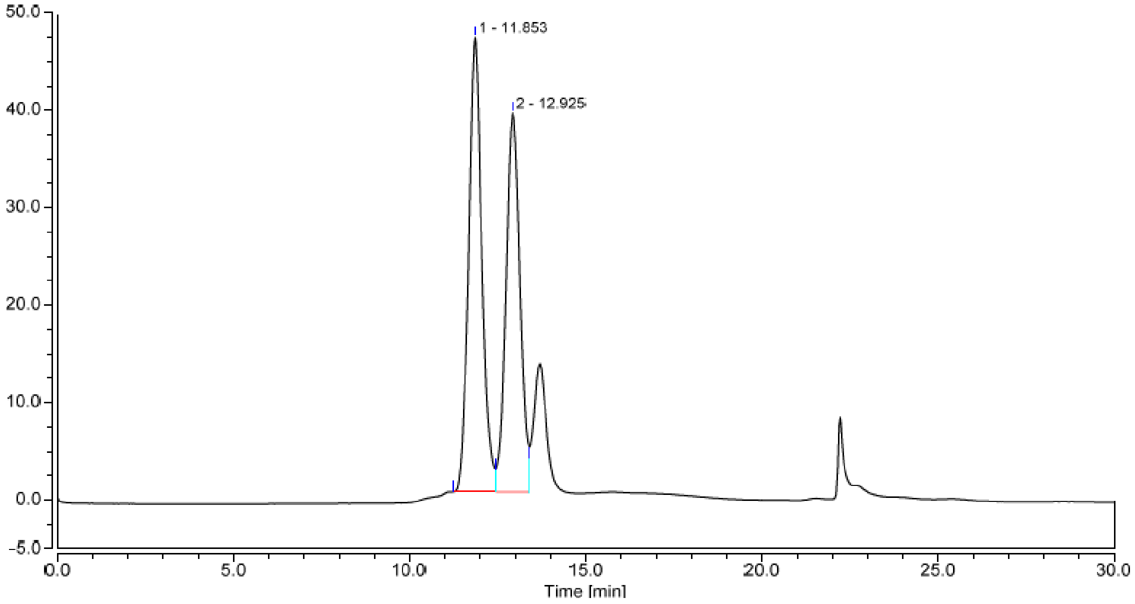

Integration Results							
No.	Peak Name	Retention Time min	Area mAU*min	Height mAU	Relative Area %	Relative Height %	Amount n.a.
1		11.853	20.223	46.627	52.97	54.52	n.a.
2		12.925	17.953	38.893	47.03	45.48	n.a.
Total:			38.176	85.521	100.00	100.00	

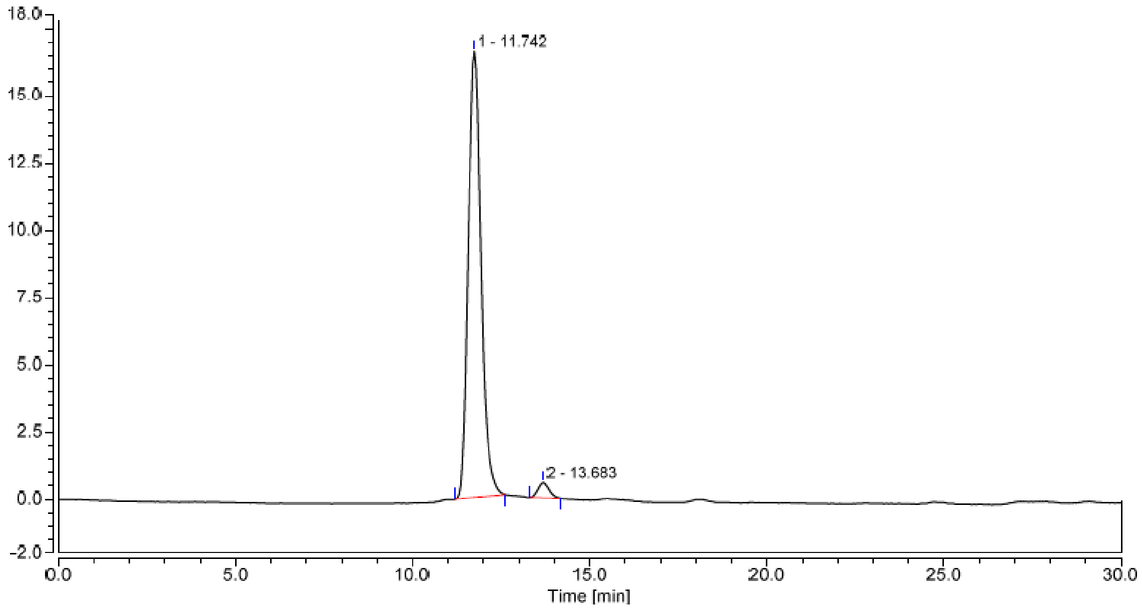

Integration Results							
No.	Peak Name	Retention Time min	Area mAU*min	Height mAU	Relative Area %	Relative Height %	Amount n.a.
1		11.742	6.969	16.590	97.11	96.72	n.a.
2		13.683	0.207	0.563	2.89	3.28	n.a.
**Total:**			**7.176**	**17.153**	**100.00**	**100.00**	

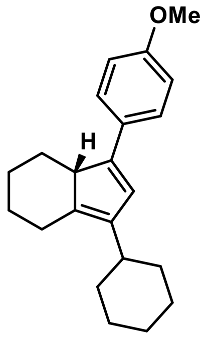

1c

Cyclopentadiene **1c** was prepared via general procedure **E** with **9a** (0.40 g, 1.8 mmol, 88% ee), 4-methoxyphenyllithium (2.8 mmol, 1.5 eq.) and 3 : 1 pentane / DCM eluent to give a white solid (0.24 g, 42% yield, 88% ee).

^{1}**H NMR** (400 MHz, C_6D_6) δ 7.35 (d, *J* = 8.8 Hz, 2H), 6.87 (d, *J* = 8.8 Hz, 2H), 6.79 (s, 1H), 3.37 (s, 3H), 3.01 – 2.91 (m, 1H), 2.82 – 2.73 (m, 1H), 2.61 – 2.48 (m, 1H), 2.37 – 2.27 (m, 1H), 2.16 – 2.05 (m, 1H), 1.88 – 1.74 (m, 5H), 1.73 – 1.66 (m, 1H), 1.65 – 1.59 (m, 1H), 1.55 – 1.43 (m, 2H), 1.40 – 1.28 (m, 3H), 1.27 – 1.09 (m, 2H), 0.98 – 0.80 (m, 1H).

^{13}**C NMR** (101 MHz, C_6D_6) δ 158.8, 148.6, 142.2, 141.0, 129.6, 127.1, 125.8, 114.4, 54.8, 52.5, 36.8, 34.1, 33.7, 33.4, 30.2, 27.1, 26.7, 26.6, 26.4.

IR (ATR) 2921, 2848, 1606, 1575, 1548, 1504, 1444, 1418, 1339, 1301, 1280, 1245, 1175, 1147, 1105, 1036, 998, 971, 889, 833, 823.

HRMS (ESI + APCI) m/z: $[M+H]^+$ calculated for $[C_{22}H_{29}O]^+$: 309.2213; found: 309.2199.

m.p. 102.6 – 105.1 °C.

Rf 0.21 (silica gel, 10:1 pentane / DCM, UV / CAM).

HPLC CHIRALPAK® IA, 99:1 hexane / IPA, rate 1 mL / min, 25 min, 320 nm, t_R minor 5.23 min; t_R major 5.71 min.

Opt. Rot. $[\alpha]_D^{21}$ +0.86 (c = 1.00 in $CHCl_3$, 88% ee).

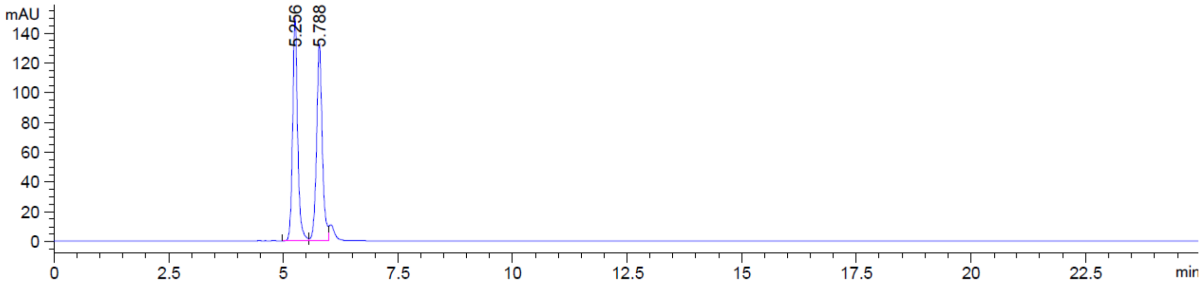

Peak #	RetTime [min]	Type	Width [min]	Area [mAU*s]	Height [mAU]	Area %
1	5.256	BV	0.1102	1141.56982	150.93532	50.0487
2	5.788	VV	0.1266	1139.34607	132.14056	49.9513

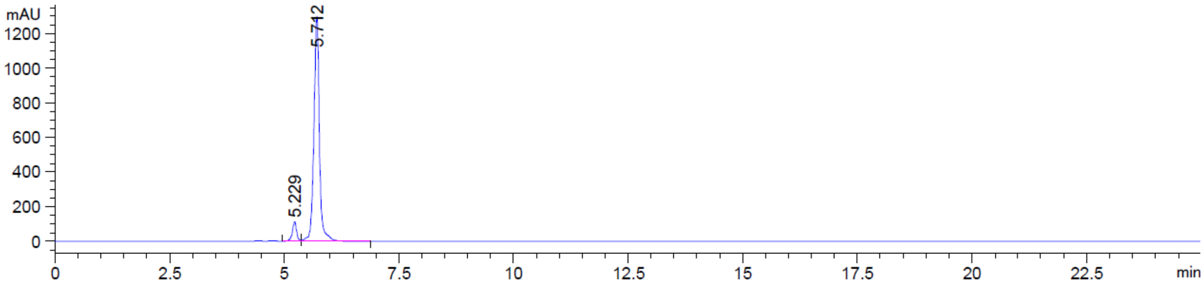

Peak #	RetTime [min]	Type	Width [min]	Area [mAU*s]	Height [mAU]	Area %
1	5.229	BV	0.0981	731.82568	112.20737	6.3968
2	5.712	VB	0.1220	1.07086e4	1301.30090	93.6032

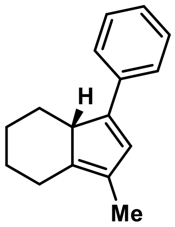

1d

Cyclopentadiene **1d** was prepared via general procedure **E** with **9b** (88 mg, 0.59 mmol, 95% ee), phenyllithium (0.49 mL, 1.8 M in hexane, 0.88 mmol) and 50 : 1 DCM/EtOAc eluent to give a white solid (69 mg, 56% yield, 95% ee).

^{1}H NMR (400 MHz, $CDCl_3$) δ 7.42 – 7.38 (m, 2H), 7.31 (t, *J* = 7.8 Hz, 2H), 7.15 (t, *J* = 7.3 Hz, 1H), 6.64 (s, 1H), 3.08 (dd, *J* = 12.6, 5.9 Hz, 1H), 2.77 – 2.68 (m, 1H), 2.42 – 2.32 (m, 1H), 2.22 – 2.11 (m, 1H), 2.02 – 1.94 (m, 1H), 1.92 (t, *J* = 1.6 Hz, 3H), 1.83 – 1.76 (m, 1H), 1.53 – 1.43 (m, 1H), 1.23 – 1.14 (m, 1H), 0.81 (qd, *J* = 12.9, 3.4 Hz, 1H).

^{13}C NMR (101 MHz, $CDCl_3$) δ 148.1, 144.9, 135.9, 131.1, 130.3, 128.6, 126.1, 125.6, 52.1, 33.6, 29.3, 26.4, 26.0, 12.3.

IR (ATR) 2928, 2852, 1597, 1491, 1443, 860, 756, 692.

HRMS (ESI + APCI) m/z: $[M + H]^+$ calculated for $[C_{16}H_{19}]^+$: 211.1481; found: 211.1470.

m.p. 46.2 – 47.9 °C.

R*f* 0.35 (silica gel, 10:1 pentane / EtOAc, UV / CAM).

HPLC CHIRALPAK® IG, 99.5:0.5 hexane / IPA, rate 1 mL / min, 25 min, 320 nm, t_R minor 4.19 min; t_R major 5.51 min.

Opt. Rot. $[\alpha]_D^{20}$ +74.17 (c = 1.00 in $CHCl_3$, 95% ee).

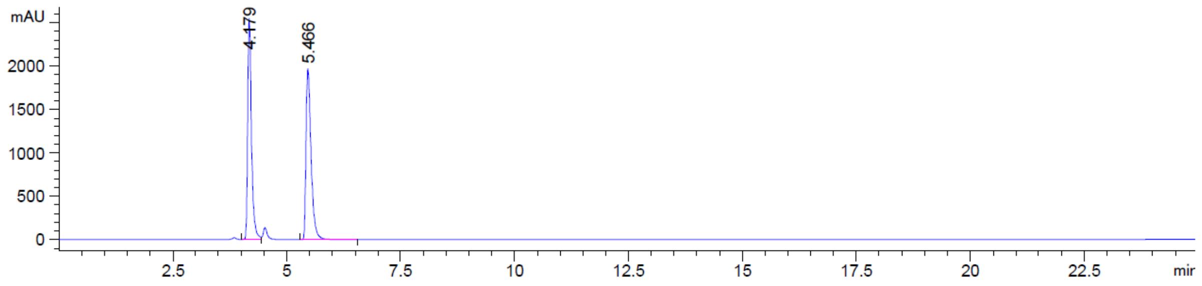

Peak #	RetTime [min]	Type	Width [min]	Area [mAU*s]	Height [mAU]	Area %
1	4.179	VV	0.0873	1.47147e4	2552.43188	49.2173
2	5.466	BB	0.1179	1.51826e4	1971.37231	50.7827

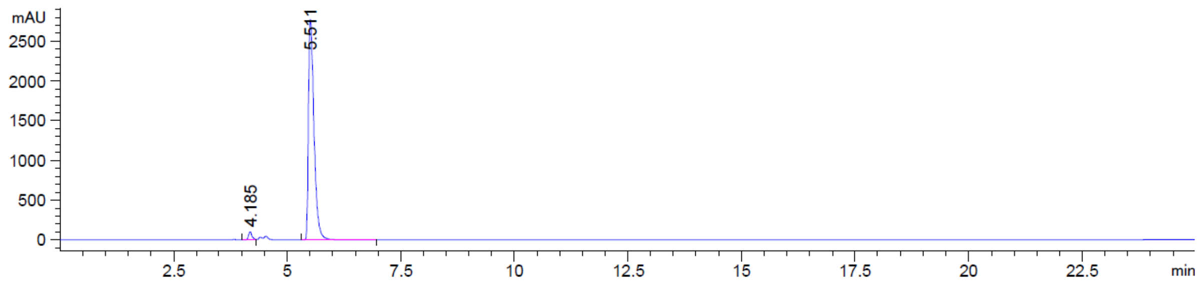

Peak #	RetTime [min]	Type	Width [min]	Area [mAU*s]	Height [mAU]	Area %
1	4.185	BV	0.0814	551.52533	101.51582	2.2748
2	5.511	BB	0.1315	2.36931e4	2779.24487	97.7252

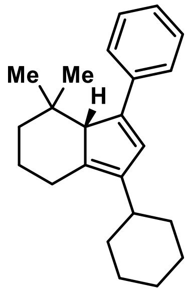

1e

Cyclopentadiene **1e** was prepared via general procedure **E** with a 3.7 : 1 mixture of **9c** and **9c'** (21.3 mg, 0.674 mmol of **9c**, 84% ee), phenyllithium (53 µL, 1.8 M in hexane, 0.10 mmol), 2-nitrobenzenesulfonyl chloride (22.4 mg, 0.10 mmol) and pentane eluent to give a colorless oil (14.1 mg, 68% yield based on **9c**, 80% ee).

Note: Subjecting a pure sample of isomer *rac*-**9c'** to the same reaction conditions leads to full conversion and no formation of desired arylation product.

^{1}H NMR (400 MHz, $CDCl_3$) δ 7.34 – 7.27 (m, 4H), 7.21 – 7.15 (m, 1H), 6.46 (s, 1H), 3.12 (d, *J* = 0.9 Hz, 1H), 2.79 – 2.67 (m, 1H), 2.45 (ddt, *J* = 11.5, 7.1, 3.6 Hz, 1H), 2.11 (td, *J* = 13.0, 5.4 Hz, 1H), 1.83 – 1.62 (m, 6H), 1.58 – 1.52 (m, 1H), 1.44 – 1.23 (m, 7H), 0.91 (s, 3H), 0.42 (s, 3H).

^{13}C NMR (101 MHz, $CDCl_3$) δ 149.1, 141.8, 140.1, 139.8, 131.4, 128.1, 127.6, 126.0, 61.5, 42.1, 38.5, 36.5, 33.7, 32.7, 31.8, 26.8, 26.8, 26.4, 26.0, 23.7, 18.9.

IR (ATR) 2920, 2849, 1598, 1488, 1445, 1383, 1338, 1254, 1221, 1071, 1034.

HRMS (ESI) m/z: $[M+H]^+$ calculated for $[C_{23}H_{31}]^+$: 307.2420; found: 307.2422.

Rf 0.70 (silica gel, pentane, UV / PMA).

HPLC CHIRALPAK® IG, 99.7:0.3 hexane / IPA, rate 1 mL / min, 15 min, 280 nm, t_R minor 3.69

min; t_R major 3.99 min.

Opt. Rot. $[\alpha]_D^{21}$ −34.06 (c = 0.23 in $CHCl_3$, 80% ee).

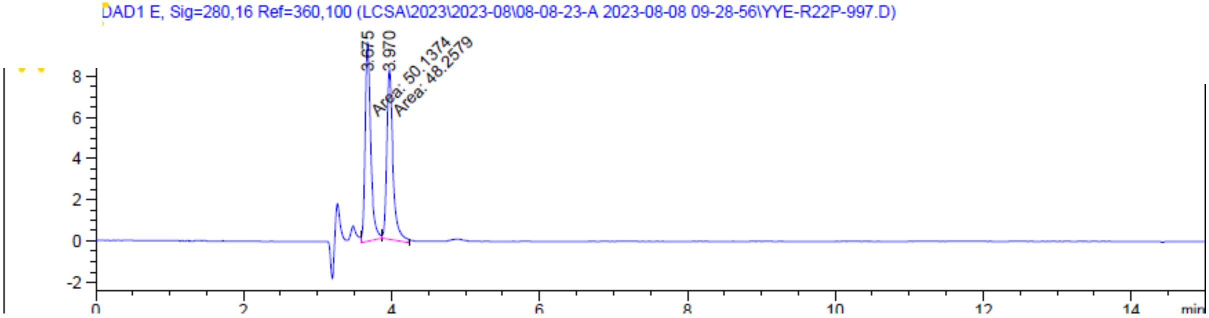

Signal 5: DAD1 E, Sig=280,16 Ref=360,100

Peak #	RetTime [min]	Type	Width [min]	Area [mAU*s]	Height [mAU]	Area %
1	3.675	MM T	0.0859	50.13737	9.72979	50.9551
2	3.970	MM T	0.0974	48.25787	8.25513	49.0449

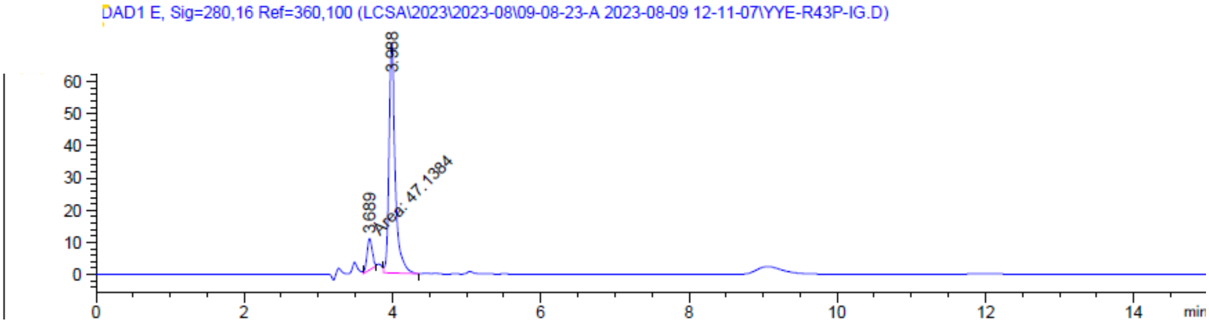

Signal 5: DAD1 E, Sig=280,16 Ref=360,100

Peak #	RetTime [min]	Type	Width [min]	Area [mAU*s]	Height [mAU]	Area %
1	3.689	MM T	0.0805	47.13842	9.76102	9.9485
2	3.988	VB	0.0894	426.68384	71.70310	90.0515

3. Synthesis and Characterization of Rh(I) and Ir(I) Cp Complexes

General procedure F for the synthesis of [Cp^{X}Rh(COD)] and [Cp^{X}Ir(COD)] complexes

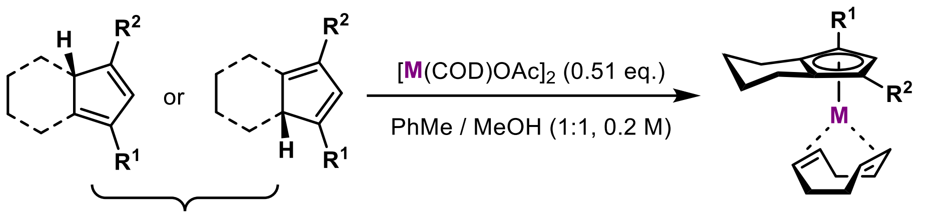

A dry mixture of cyclopentadiene **1** or **8** (1.0 eq.) and $[M(COD)OAc]_2$ (M = Rh or Ir, 0.51 eq.) under N_2 was dissolved in dry degassed PhMe / MeOH (1:1, 0.2 M), and stirred in a closed vessel at the specified temperature and for the specified duration. The reaction mixture was concentrated *in vacuo* and the crude residue subjected to either column chromatography or preparatory TLC (silica gel) to afford the [Cp^{X}M(COD)] complex **2** or **5**.

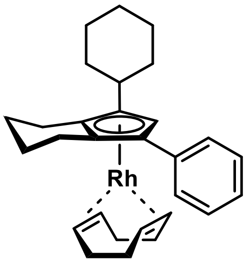

2a

Rh(I) complex **2a** was prepared via general procedure **F** with **1a** (15 mg, 53 µmol, 95% ee), $[Rh(COD)OAc]_2$ (15 mg, 27 µmol), temperature 23 °C, duration 3 h and purifying by column chromatography (50:1 pentane / EtOAc) to give an orange solid (22 mg, 83% yield, 95% ee).

^{1}H NMR (400 MHz, C_6D_6) δ 7.44 (dd, *J* = 8.3, 1.3 Hz, 2H), 7.21 (dd, *J* = 8.4, 7.1 Hz, 2H), 7.10 – 7.03 (m, 1H), 5.27 (s, 1H), 3.47 – 3.37 (m, 2H), 3.31 – 3.21 (m, 2H), 2.76 (ddd, *J* = 14.9, 8.8, 5.5 Hz, 1H), 2.48 (dt, *J* = 15.6, 5.4 Hz, 1H), 2.34 – 2.12 (m, 7H), 2.09 – 1.91 (m, 6H), 1.90 – 1.82 (m, 1H), 1.78 – 1.63 (m, 3H), 1.61 – 1.46 (m, 3H), 1.43 – 1.28 (m, 3H), 1.27 – 1.16 (m, 2H).

^{13}C NMR (101 MHz, C_6D_6) δ 136.5, 128.5, 126.0, 109.3 (d, *J* = 4.2 Hz), 101.6 (d, *J* = 3.6 Hz), 100.9 (d, *J* = 3.5 Hz), 99.5 (d, *J* = 4.1 Hz), 80.7 (d, *J* = 4.1 Hz), 70.9 (d, *J* = 14.1 Hz), 70.1 (d, *J* = 14.2 Hz), 36.0, 35.7, 33.6, 33.2, 32.8, 27.4, 27.3, 26.8, 24.8, 24.2, 23.4, 22.7.

IR (ATR) 2920, 2848, 2820, 1600, 1445, 1321, 1259, 1070, 1016, 997, 864, 796, 695, 661, 496.

HRMS (ESI + APCI) m/z: $[M]^+$ calculated for $[C_{29}H_{37}Rh]^+$: 488.1945; found: 488.1918.

m.p. 80.2 – 81.9 °C.

Rf 0.43(silica gel, 5:1 pentane / DCM, UV / CAM).

HPLC 2 × CHIRALPAK® IA in series, 99.5:0.5 hexane / IPA, rate 0.5 mL / min, 22 min, 254 nm, t_R major 16.60 min; t_R minor 17.52 min.

Opt. Rot. $[\alpha]_D^{21}$ −68.83 (c = 1.0 in $CHCl_3$, 95% ee).

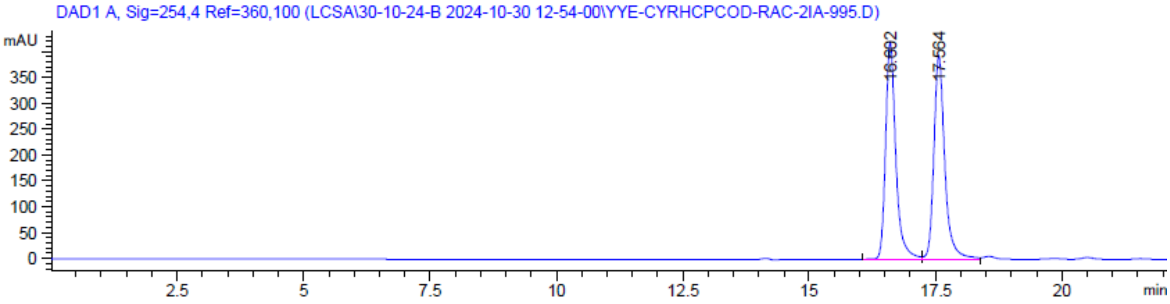

Peak #	RetTime [min]	Type	Width [min]	Area [mAU*s]	Height [mAU]	Area %
1	16.602	BV	0.2104	5850.37500	420.38245	49.9439
2	17.564	VV	0.2245	5863.51270	392.04727	50.0561

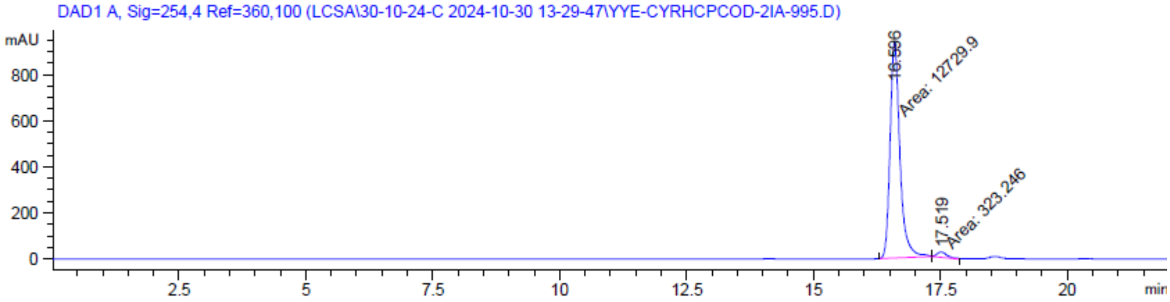

Peak #	RetTime [min]	Type	Width [min]	Area [mAU*s]	Height [mAU]	Area %
1	16.596	MM T	0.2251	1.27299e4	942.39063	97.5236
2	17.519	MM T	0.2273	323.24628	23.69810	2.4764

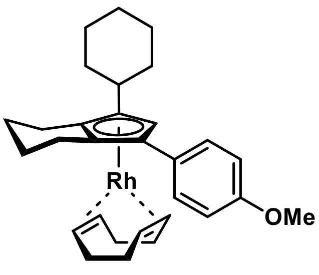

2b

Rh(I) complex **2b** was prepared via general procedure **F** with **1c** (50 mg, 0.16 mmol, 88% ee), $[Rh(COD)OAc]_2$ (45 mg, 83 µmol), temperature 23 °C, duration 3 h and purifying by column

chromatography (pentane to 10:1 pentane / DCM gradient) to give an orange oil (53 mg, 63% yield, 86% ee).

^{1}H NMR (400 MHz, C_6D_6) δ 7.38 (d, *J* = 8.8 Hz, 2H), 6.84 (d, *J* = 8.8 Hz, 2H), 5.30 (s, 1H), 3.47 – 3.39 (m, 2H), 3.34 (s, 3H), 3.31 – 3.24 (m, 2H), 2.81 (ddd, *J* = 14.9, 8.8, 5.5 Hz, 1H), 2.52 (dt, *J* = 15.5, 5.4 Hz, 1H), 2.37 – 2.26 (m, 4H), 2.25 – 2.18 (m, 2H), 2.18 – 2.10 (m, 1H), 2.09 – 1.96 (m, 6H), 1.91 – 1.83 (m, 1H), 1.80 – 1.67 (m, 3H), 1.63 – 1.55 (m, 2H), 1.55 – 1.48 (m, 1H), 1.44 – 1.34 (m, 2H), 1.34 – 1.28 (m, 1H), 1.28 – 1.18 (m, 2H)

^{13}C NMR (101 MHz, C_6D_6) δ 158.4, 128.9, 128.7, 114.1, 108.8 (d, *J* = 4.3 Hz), 101.2 (d, *J* = 3.7 Hz), 100.4 (d, *J* = 3.5 Hz), 99.7 (d, *J* = 4.1 Hz), 80.5 (d, *J* = 4.1 Hz), 70.8 (d, *J* = 14.1 Hz), 70.0 (d, *J* = 14.1 Hz), 54.8, 36.0, 35.8, 33.5, 33.3, 32.9, 27.4, 27.3, 26.8, 24.8, 24.2, 23.5, 22.7.

IR (ATR) 2921, 2849, 2822, 1611, 1521, 1445, 1322, 1299, 1287, 1272, 1244, 1176, 1037, 864, 830, 795.

HRMS (ESI + APCI) m/z: $[M + H]^+$ calculated for $[C_{30}H_{40}ORh]^+$: 519.2129; found: 519.2114.

R*f* 0.11 (silica gel, 50:1 pentane / DCM, UV / CAM).

HPLC CHIRALPAK® IA, 99:1 hexane / IPA, rate 1 mL / min, 15 min, 254 nm, t_R major 2.80 min; t_R minor 3.15 min.

Opt. Rot. $[\alpha]_D^{21}$ −49.67 (c = 1.0 in $CHCl_3$, 86% ee).

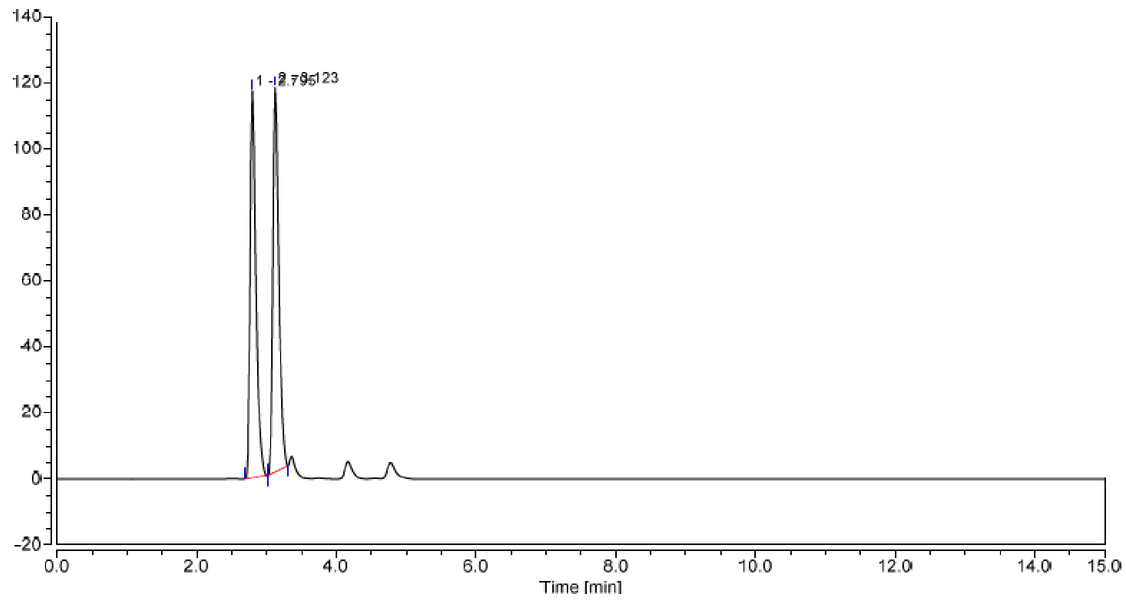

Integration Results							
No.	Peak Name	Retention Time<br>min	Area<br>mAU*min	Height<br>mAU	Relative Area<br>%	Relative Height<br>%	Amount<br>n.a.
1		2.795	11.180	117.364	49.67	50.18	n.a.
2		3.123	11.331	116.536	50.33	49.82	n.a.
**Total:**			**22.511**	**233.900**	**100.00**	**100.00**	

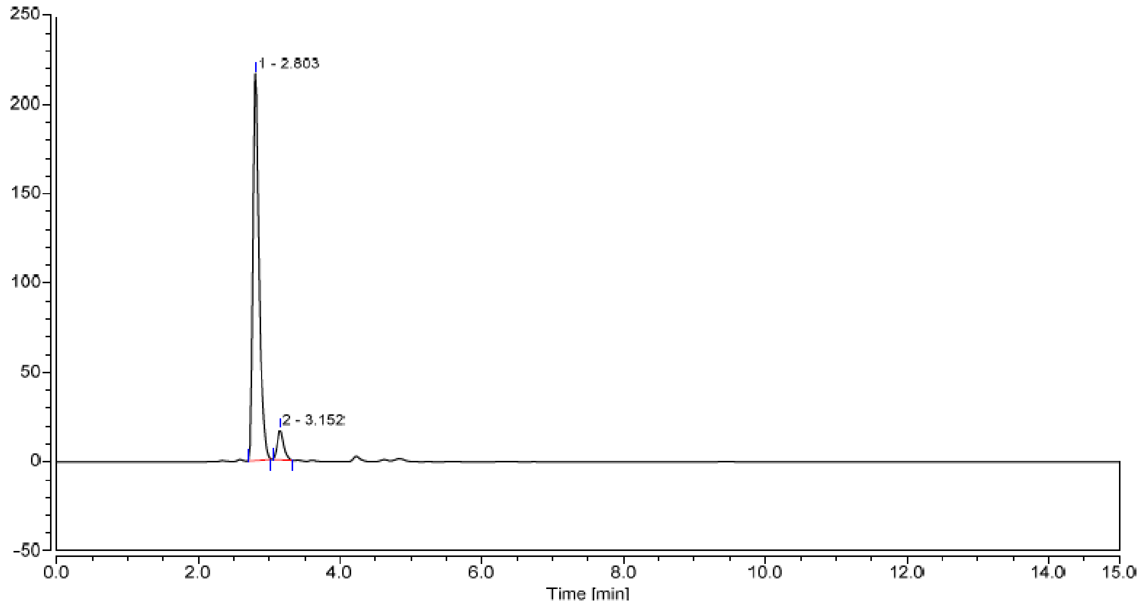

Integration Results							
No.	Peak Name	Retention Time<br>min	Area<br>mAU*min	Height<br>mAU	Relative Area<br>%	Relative Height<br>%	Amount<br>n.a.
1		2.803	21.433	216.554	92.58	92.79	n.a.
2		3.152	1.717	16.828	7.42	7.21	n.a.
**Total:**			**23.150**	**233.382**	**100.00**	**100.00**	

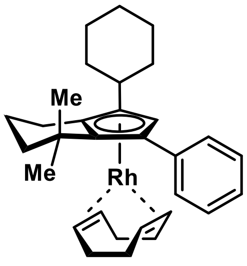

2c

Rh(I) complex **2c** was prepared via general procedure **F** with **1e** (17 mg, 55 µmol, 80% ee), $[Rh(COD)OAc]_2$ (15 mg, 28 µmol), temperature 23 °C, duration 3 h and purifying by column chromatography (50:1 pentane / EtOAc) to give a yellow oil (26 mg, 89% yield, 81% ee as determined after conversion to compound **10b**).

^{1}H NMR (400 MHz, C_6D_6) δ 7.59 – 7.53 (m, 2H), 7.20 – 7.13 (m, 2H), 7.11 – 7.04 (m, 1H), 4.89 (s, 1H), 3.75 (td, *J* = 7.7, 2.8 Hz, 2H), 3.38 (td, *J* = 7.7, 3.2 Hz, 2H), 2.50 – 2.30 (m, 5H), 2.21 – 2.06 (m, 4H), 2.06 – 1.96 (m, 3H), 1.93 – 1.84 (m, 1H), 1.77 (s, 3H), 1.75 – 1.65 (m, 3H), 1.65 – 1.56 (m, 2H), 1.54 – 1.44 (m, 2H), 1.42 – 1.27 (m, 3H), 1.27 – 1.16 (m, 2H), 1.05 (s, 3H).

^{13}C NMR (101 MHz, C_6D_6) δ 138.5, 131.4, 128.4 (seen in DEPT-135), 126.8, 113.8 (d, *J* = 3.8 Hz), 109.1 (d, *J* = 4.2 Hz), 103.1 (d, *J* = 4.1 Hz), 97.9 (d, *J* = 3.8 Hz), 84.8 (d, *J* = 4.1 Hz), 70.8 (d, *J* = 14.3 Hz), 66.9 (d, *J* = 14.0 Hz), 42.2, 36.2, 35.9, 33.9, 33.9, 32.6, 32.4, 29.7, 27.4, 27.3, 26.8, 22.4, 20.2.

IR (ATR) 2984, 2920, 2848, 2822, 1600, 1472, 1445, 1359, 1322, 864, 700.

HRMS (ESI) m/z: $[M]^+$ calculated for $[C_{31}H_{41}Rh]^+$: 516.2258; found: 516.2257.

R*f* 0.65 (silica gel, pentane, UV / CAM).

Opt. Rot. $[\alpha]_D^{20}$ −81.56 (c = 0.47 in $CHCl_3$, 81% ee).

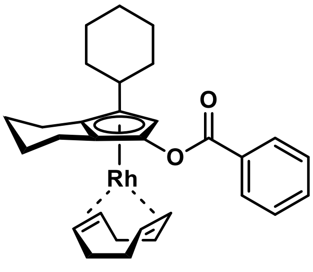

2d

Rh(I) complex **2d** was prepared via general procedure **F** with **8a** (17 mg, 53 µmol, 95% ee), $[Rh(COD)OAc]_2$ (17 mg, 27 µmol), temperature 23 °C, duration 3 h and purifying by column

chromatography (100:1 pentane / EtOAc) to give a yellow solid (27 mg, 95% yield, 94% ee). Yellow crystals suitable for X-ray analysis were obtained by slow evaporation of a solution of *rac*-**2d** in pentane.

^{1}H NMR (400 MHz, C_6D_6) δ 8.19 – 8.09 (m, 2H), 7.12 – 7.06 (m, 1H), 7.05 – 6.96 (m, 2H), 5.81 (s, 1H), 3.69 – 3.59 (m, 2H), 3.56 – 3.42 (m, 2H), 2.79 – 2.62 (m, 1H), 2.50 – 2.26 (m, 5H), 2.20 – 2.08 (m, 4H), 2.07 – 1.96 (m, 3H), 1.95 – 1.89 (m, 1H), 1.86 – 1.72 (m, 4H), 1.69 – 1.59 (m, 2H), 1.57 – 1.45 (m, 2H), 1.43 – 1.02 (m, 5H).

^{13}C NMR (101 MHz, C_6D_6) δ 164.0, 133.3, 130.4, 130.1, 128.7, 119.1 (d, J = 4.5 Hz), 103.9 (d, *J* = 4.5 Hz), 97.1 (d, *J* = 3.9 Hz), 95.4 (d, *J* = 3.5 Hz), 75.3 (d, *J* = 3.7 Hz), 70.7 (d, *J* = 14.0 Hz), 70.6 (d, *J* = 13.9 Hz), 35.6, 35.6, 33.5, 32.7, 32.2, 27.2, 27.2, 26.6, 23.7, 23.5, 21.5, 21.4.

IR (ATR) 2925, 2851, 2824, 1738, 1600, 1449, 1393, 1342, 1323, 1312, 1261, 1176, 1134, 1036, 706.

HRMS (ESI + APCI) m/z: $[M]^+$ calculated for $[C_{30}H_{37}O_2Rh]^+$: 532.1843; found: 532.1842.

m.p. 129.8 – 130.7 °C.

R*f* 0.49 (silica gel, 20:1 pentane / EtOAc, UV / CAM).

Elemental Analysis calculated for $C_{22}H_{25}O_2Rh{\bullet}C_8H_{12}$: C, 67.66; H, 7.00; N, 0.00. found: C, 67.77; H, 7.11; N, 0.03.

HPLC CHIRALPAK® IA, 99.5:0.5 hexane / IPA, rate 1 mL / min, 25 min, 254 nm, t_R major 5.37 min; t_R minor 6.59 min.

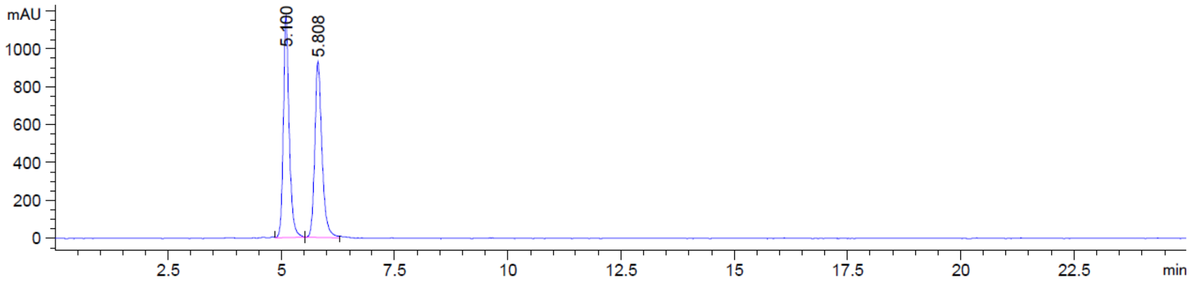

Peak #	RetTime [min]	Type	Width [min]	Area [mAU*s]	Height [mAU]	Area %
1	5.100	VB	0.1302	1.04294e4	1167.37341	50.5299
2	5.808	BV	0.1606	1.02106e4	926.42371	49.4701

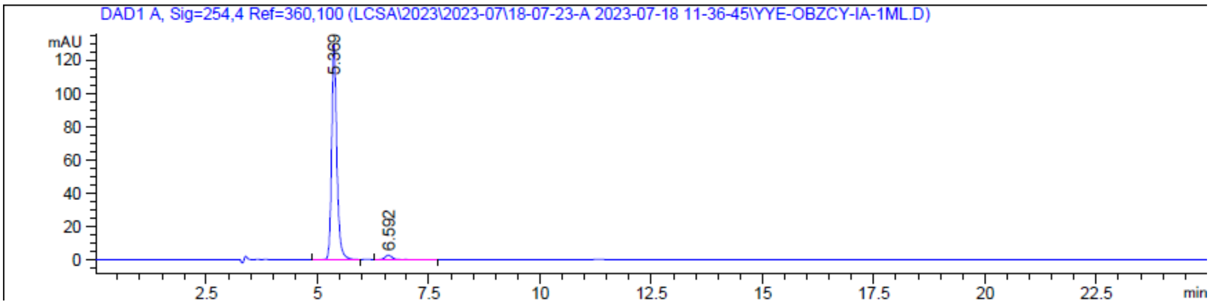

Signal 1: DAD1 A, Sig=254,4 Ref=360,100

Peak #	RetTime [min]	Type	Width [min]	Area [mAU*s]	Height [mAU]	Area %
1	5.369	BV	0.1237	1061.52942	129.40984	96.8739
2	6.592	VB	0.1843	34.25500	2.76870	3.1261

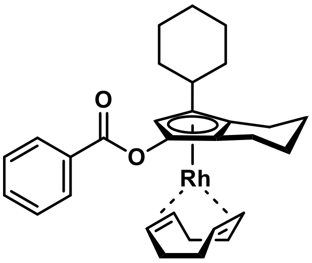

ent-**2d**

Following general procedure **F**, *ent*-**2d** (97% ee) was also synthesized from *ent*-**8a** (98% ee, originally obtained after chiral separation of *rac*-**8a**).

HPLC CHIRALPAK® IA, 99.5:0.5 hexane / IPA, rate 1 mL / min, 25 min, 254 nm, t_R minor 5.11 min; t_R major 5.83 min.

Opt. Rot. $[\alpha]_D^{20}$ −22.73 (c = 0.1 in $CHCl_3$, 97% ee).

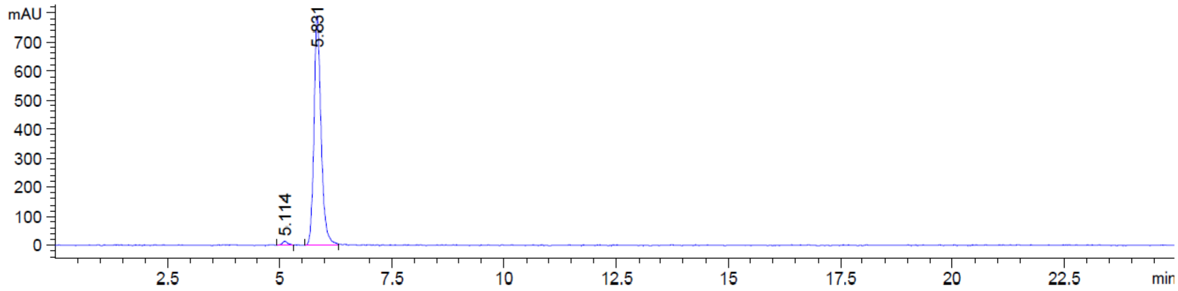

Peak #	RetTime [min]	Type	Width [min]	Area [mAU*s]	Height [mAU]	Area %
1	5.114	VV	0.1355	142.15863	15.14735	1.6214
2	5.831	BV	0.1634	8625.42773	789.65686	98.3786

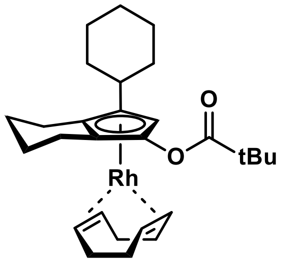

2e

Rh(I) complex **2e** was prepared via general procedure **F** with **8b** (20 mg, 66 µmol, 85% ee), $[Rh(COD)OAc]_2$ (18 mg, 33 µmol), temperature 23 °C, duration 3 h and purifying by column chromatography (100:1 pentane / EtOAc) to give a yellow oil (29 mg, 85% yield, 85% ee).

^{1}H NMR (400 MHz, C_6D_6) δ 5.78 (d, *J* = 0.7 Hz, 1H), 3.59 – 3.50 (m, 2H), 3.45 – 3.39 (m, 2H), 2.67 (ddd, *J* = 15.4, 7.7, 5.7 Hz, 1H), 2.46 – 2.24 (m, 5H), 2.19 – 2.07 (m, 4H), 2.06 – 1.94 (m, 3H), 1.93 – 1.69 (m, 5H), 1.68 – 1.47 (m, 4H), 1.41 – 1.17 (m, 3H), 1.14 (s, 9H), 1.12 – 1.00 (m, 2H).

^{13}C NMR (101 MHz, C_6D_6) δ 175.4, 118.9 (d, *J* = 4.5 Hz), 103.6 (d, *J* = 4.5 Hz), 97.0 (d, *J* = 3.9 Hz), 95.0 (d, *J* = 3.4 Hz), 75.1 (d, *J* = 3.8 Hz), 70.7 (d, *J* = 13.6 Hz), 70.6 (d, *J* = 13.7 Hz), 39.4, 35.7, 35.5, 33.6, 32.7, 32.1, 30.9, 27.3, 27.2, 27.1, 26.6, 23.7, 23.5, 21.5, 21.2.

IR (ATR) 2922, 2850, 2822, 1750, 1478, 1448, 1391, 1362, 1342, 1323, 1275, 1167, 1116, 886, 864, 813.

HRMS (ESI + APCI) m/z: $[M + H]^+$ calculated for $[C_{28}H_{42}O_2Rh]^+$: 513.2234; found: 513.2233.

m.p. 110.4 – 111.9 °C.

R*f* 0.33 (silica gel, 50:1 pentane / EtOAc, UV / CAM).

HPLC CHIRALPAK® IA, 99.99:0.01 hexane / IPA, rate 1 mL / min, 25 min, 254 nm, t_R minor 4.90 min; t_R major 5.65 min.

Opt. Rot. $[\alpha]_D^{20}$ +40.83 (c = 1.0 in $CHCl_3$, 85% ee).

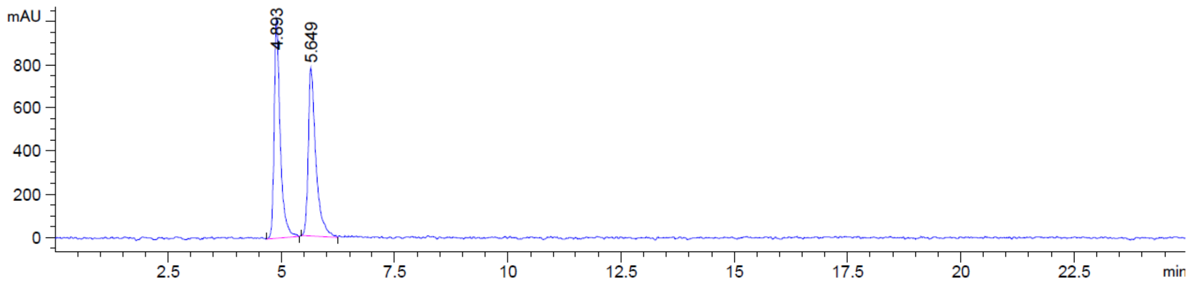

Peak #	RetTime [min]	Type	Width [min]	Area [mAU*s]	Height [mAU]	Area %
1	4.893	BB	0.1334	9371.50781	1018.06842	50.6864
2	5.649	BB	0.1725	9117.68457	779.44031	49.3136

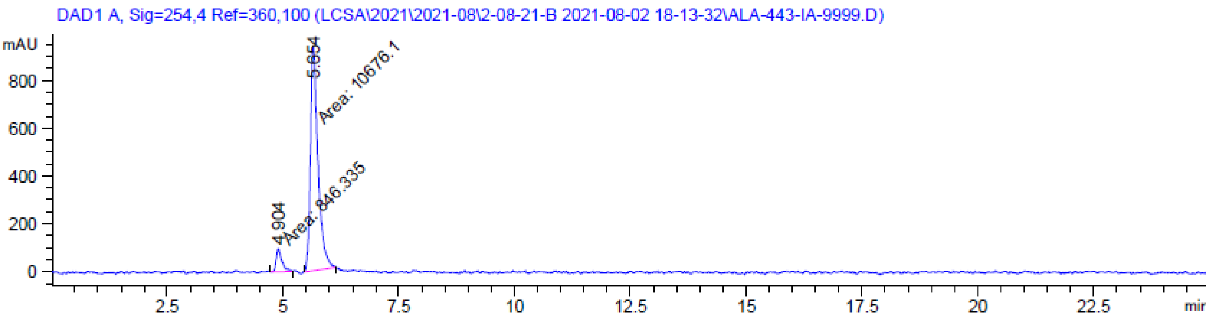

Peak #	RetTime [min]	Type	Width [min]	Area [mAU*s]	Height [mAU]	Area %
1	4.904	MM T	0.1475	846.33466	95.62830	7.3451
2	5.654	MM T	0.1904	1.06761e4	934.61841	92.6549

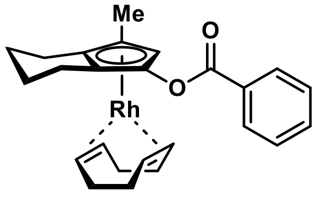

2f

Rh(I) complex **2f** was prepared via general procedure **F** with **8c** (4.0 mg, 16 µmol, 97% ee), $[Rh(COD)OAc]_2$ (4.3 mg, 8.0 µmol), temperature 0 °C, duration 30 min and purifying by column chromatography (50:1 pentane / EtOAc) to give a yellow oil (6.2 mg, 85% yield, 90% ee). Yellow crystals suitable for X-ray analysis were obtained by cooling a saturated solution of *rac*-**2f** in pentane to −20 °C.

^{1}H NMR (400 MHz, C_6D_6) δ 8.16 – 8.09 (m, 2H), 7.11 – 7.05 (m, 1H), 7.00 (t, *J* = 7.3 Hz, 2H), 5.84 (s, 1H), 3.56 – 3.46 (m, 2H), 3.46 – 3.37 (m, 2H), 2.51 – 2.41 (m, 1H), 2.41 – 2.28 (m, 5H), 2.15 – 1.99 (m, 6H), 1.87 – 1.72 (m, 2H), 1.59 – 1.47 (m, 2H), 1.41 (s, 3H).

^{13}C NMR (101 MHz, C_6D_6) δ 164.1, 133.3, 130.3, 130.2, 128.7, 119.4 (d, *J* = 4.7 Hz), 99.4 (d, *J* =

3.8 Hz), 96.6 (d, J = 3.2 Hz), 90.2 (d, J = 4.5 Hz), 77.9 (d, J = 3.9 Hz), 71.8, 71.6, 71.1, 71.0, 33.3, 33.1, 23.6, 23.4, 21.2 (d, J = 5.2 Hz), 10.1.

IR (ATR) 2981, 2925, 2870, 2823, 1739, 1450, 1393, 1372, 1323, 1312, 1261, 1170, 1131, 1083, 1062, 1026, 864, 705.

HRMS (nanochip-ESI/LTQ-Orbitrap) m/z: $[M + H]^+$ calculated for $[C_{25}H_{30}O_2Rh]^+$: 465.1295; found: 465.1277.

m.p. 129.3 – 130.4 °C (racemate).

Rf 0.46 (silica gel, 50:1 pentane / EtOAc, UV / CAM).

HPLC CHIRALPAK® IA, 99.5:0.5 hexane / IPA, rate 1 mL / min, 25 min, 254 nm, t_R major 6.06 min; t_R minor 7.13 min.

Opt. Rot. $[\alpha]_D^{20}$ +3.03 (c = 0.11 in $CHCl_3$, 70% ee).

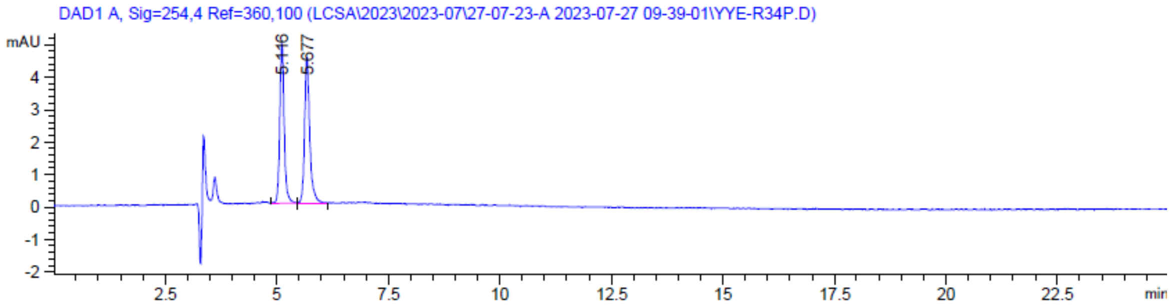

Signal 1: DAD1 A, Sig=254,4 Ref=360,100

Peak #	RetTime [min]	Type	Width [min]	Area [mAU*s]	Height [mAU]	Area %
1	5.116	BB	0.1067	33.89038	4.89706	48.6706
2	5.677	BB	0.1186	35.74176	4.50478	51.3294

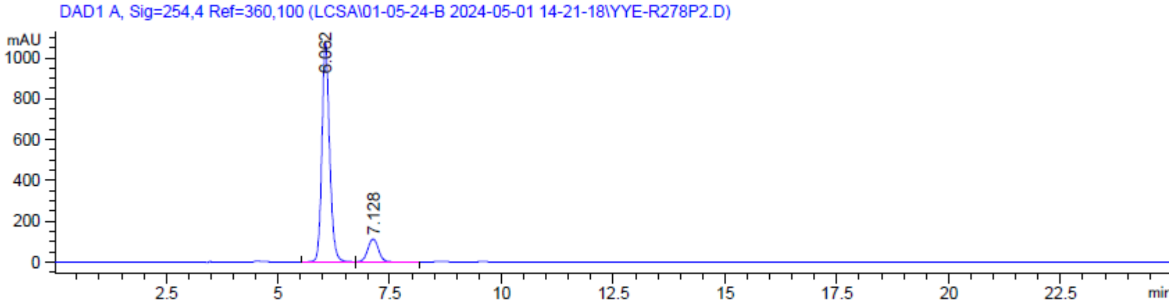

Signal 1: DAD1 A, Sig=254,4 Ref=360,100

Peak #	RetTime [min]	Type	Width [min]	Area [mAU*s]	Height [mAU]	Area %
1	6.062	VV	0.1821	1.29313e4	1076.78943	87.2944
2	7.128	VB	0.2639	1882.13635	112.08739	12.7056

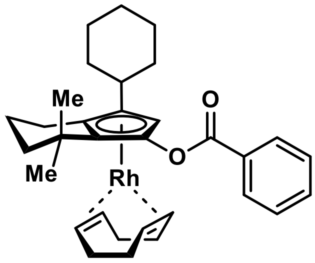

2g

Rh(I) complex **2g** was prepared via general procedure **F** with **8d** (20.2 mg, 57.6 µmol, 85% ee), $[Rh(COD)OAc]_2$ (15.8 mg, 29.4 µmol), temperature 23 °C, duration 3 h and purifying by column chromatography (50:1 pentane / EtOAc) to give a yellow solid (29.2 mg, 90% yield, 85% ee). Yellow crystals suitable for X-ray analysis were obtained by cooling a saturated solution of **2g** (85% ee) in pentane to −20 °C.

^{1}H NMR (400 MHz, C_6D_6) δ 8.23 – 8.14 (m, 2H), 7.07 (t, *J* = 7.3 Hz, 1H), 7.01 (t, *J* = 7.3 Hz, 2H), 5.78 (s, 1H), 3.87 – 3.69 (m, 2H), 3.52 – 3.38 (m, 2H), 2.47 – 2.30 (m, 5H), 2.14 – 1.99 (m, 5H), 1.98 – 1.80 (m, 6H), 1.71 – 1.57 (m, 5H), 1.54 – 1.31 (m, 8H), 1.19 – 1.09 (m, 2H).

^{13}C NMR (101 MHz, C_6D_6) δ 164.1, 133.3, 130.5, 130.0, 128.9, 119.3 (d, *J* = 4.3 Hz), 105.6 (d, *J* = 4.5 Hz), 104.0 (d, *J* = 3.8 Hz), 93.3 (d, *J* = 4.2 Hz), 77.5 (d, *J* = 3.6 Hz), 69.9, 69.8, 69.1, 68.9, 40.7, 36.1, 35.6, 35.3, 33.2, 33.1, 32.9, 31.5, 28.6, 27.4, 27.2, 26.6, 21.0, 20.6.

IR (ATR) 2924, 2851, 2824, 1737, 1449, 1311, 1260, 1174, 1144, 1082, 1065, 1026, 704.

HRMS (ESI/QTOF) m/z: $[M]^+$ calculated for $[C_{32}H_{41}O_2Rh]^+$: 560.2156; found: 560.2158.

m.p. 121.6 – 129.1 °C.

Rf 0.50 (silica gel, 50:1 pentane / EtOAc, UV / CAM).

HPLC CHIRALPAK® IA, 99.5:0.5 hexane / IPA, rate 0.5 mL / min, 25 min, 220 nm, t_R major 8.71 min; t_R minor 10.06 min.

Opt. Rot. $[\alpha]_D^{20}$ +109.90 (c = 0.32 in $CHCl_3$, 85% ee).

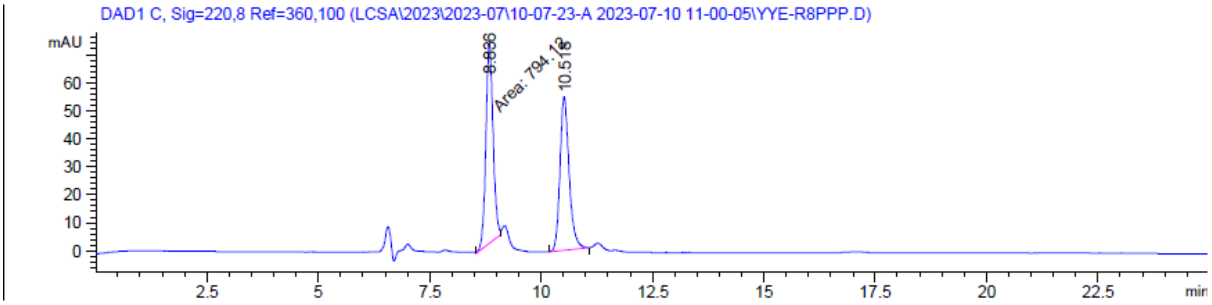

Signal 3: DAD1 C, Sig=220,8 Ref=360,100

Peak #	RetTime [min]	Type	Width [min]	Area [mAU*s]	Height [mAU]	Area %
1	8.836	MM T	0.2407	794.12012	71.24763	50.5555
2	10.518	BB	0.2158	776.67017	54.63570	49.4445

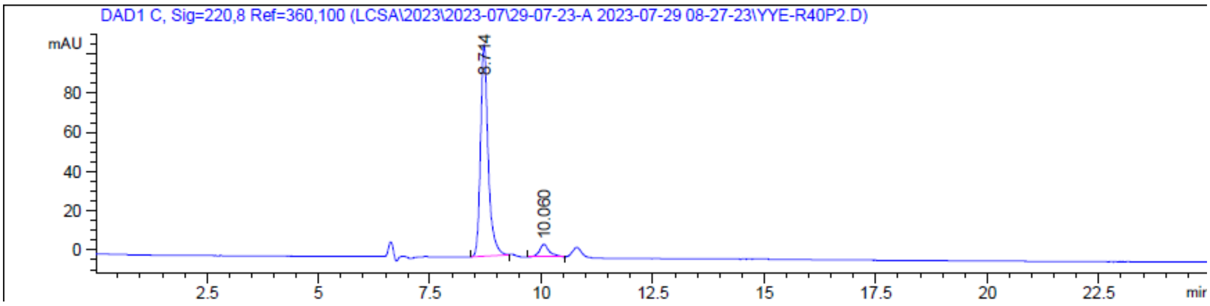

Signal 3: DAD1 C, Sig=220,8 Ref=360,100

Peak #	RetTime [min]	Type	Width [min]	Area [mAU*s]	Height [mAU]	Area %
1	8.714	BB	0.1763	1280.92151	108.06052	92.7101
2	10.060	BB	0.2268	100.72001	6.42754	7.2899

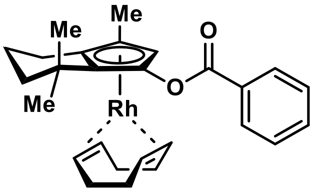

2h

Rh(I) complex **2h** was prepared via general procedure **F** with **8e** (12.9 mg, 46.0 µmol, 89% ee), $[Rh(COD)OAc]_2$ (12.6 mg, 23.5 µmol), temperature 23 °C, duration 3 h and purifying by column chromatography (50:1 pentane / EtOAc) to give a yellow solid (16.0 mg, 71% yield, 90% ee). Yellow crystals suitable for X-ray analysis were obtained by cooling a saturated solution of **2h** (90% ee) in pentane to −20 °C.

^{1}H NMR (400 MHz, C_6D_6) δ 8.16 (d, J = 6.9 Hz, 2H), 7.07 (t, J = 7.4 Hz, 1H), 7.00 (t, J = 7.3 Hz, 2H), 5.83 (s, 1H), 3.77 – 3.67 (m, 2H), 3.49 – 3.39 (m, 2H), 2.44 – 2.29 (m, 4H), 2.15 – 1.98 (m, 4H), 1.96 – 1.86 (m, 1H), 1.82 (s, 3H), 1.69 (s, 5H), 1.61 – 1.50 (m, 2H), 1.45 – 1.36 (m, 1H), 1.32 (s, 3H).

^{13}C NMR (101 MHz, C_6D_6) δ 164.2, 133.3, 130.4, 130.0, 128.8, 118.7 (d, J = 4.4 Hz), 104.6 (d, J = 4.0 Hz), 95.4 (d, J = 4.2 Hz), 93.3 (d, J = 4.2 Hz), 80.7 (d, J = 3.7 Hz), 71.0, 70.8, 69.7, 69.5, 40.5, 34.9, 33.2, 33.0, 31.6, 28.6, 20.9, 20.4, 11.1.

IR (ATR) 2958, 2925, 2869, 2823, 1737, 1451, 1428, 1389, 1371, 1312, 1260, 1174, 1136, 1085,

1065, 1026, 865, 704.

HRMS (ESI/QTOF) m/z: [M]$^+$ calculated for [$C_{27}H_{33}O_2Rh$]$^+$: 492.1530; found: 492.1539.

m.p. 139.9 – 147.0 °C.

Rf 0.46 (silica gel, 50:1 pentane / EtOAc, UV / CAM).

HPLC CHIRALPAK® IA, 99.5:0.5 hexane / IPA, rate 0.5 mL / min, 25 min, 254 nm, t_R major 9.76 min; t_R minor 12.65 min.

Opt. Rot. $[\alpha]_D^{20}$ +145.75 (c = 1.02 in $CHCl_3$, 90% ee).

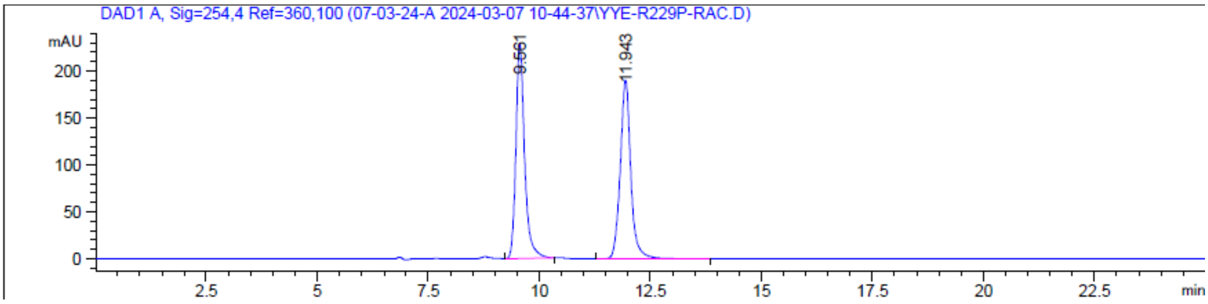

Signal 1: DAD1 A, Sig=254,4 Ref=360,100

Peak #	RetTime [min]	Type	Width [min]	Area [mAU*s]	Height [mAU]	Area %
1	9.561	BB	0.2045	3111.01880	228.95270	49.6892
2	11.943	BB	0.2447	3149.93384	190.68462	50.3108

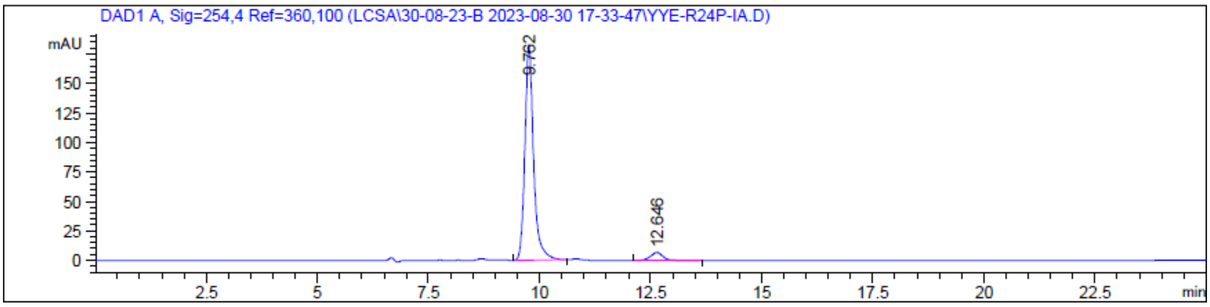

Signal 1: DAD1 A, Sig=254,4 Ref=360,100

Peak #	RetTime [min]	Type	Width [min]	Area [mAU*s]	Height [mAU]	Area %
1	9.762	BB	0.2028	2484.29810	182.51727	94.8875
2	12.646	BB	0.2950	133.85188	6.81854	5.1125

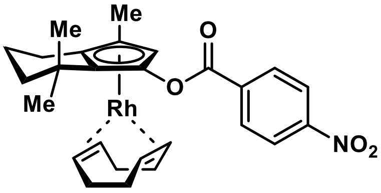

2i

Rh(I) complex **2i** was prepared via general procedure **F** with **8f** (11.5 mg, 35.1 µmol, 83% ee), $[Rh(COD)OAc]_2$ (9.7 mg, 18 µmol), temperature 23 °C, duration 16 h and purifying by column chromatography (50:1 pentane / EtOAc) to give a dark purple oil (16.8 mg, 89% yield, 81% ee). Purple crystals (racemic) suitable for X-ray analysis were obtained by cooling a saturated solution of **2i** (81% ee) in pentane to −20 °C. The resulting mother liquor was separated and concentrated to give enantioenriched **2i** as a dark purple oil (96% ee).

^{1}H NMR (400 MHz, C_6D_6) δ 7.75 (d, *J* = 8.8 Hz, 2H), 7.55 (d, *J* = 8.8 Hz, 2H), 5.82 (s, 1H), 3.77 – 3.67 (m, 2H), 3.44 – 3.36 (m, 2H), 2.46 – 2.29 (m, 4H), 2.17 – 1.98 (m, 4H), 1.96 – 1.88 (m, 1H), 1.79 (s, 3H), 1.71 – 1.62 (m, 5H), 1.61 – 1.48 (m, 2H), 1.45 – 1.38 (m, 1H), 1.29 (s, 3H).

^{13}C NMR (101 MHz, C_6D_6) δ 162.4, 150.8, 134.7, 130.5, 123.7, 118.5 (d, *J* = 4.5 Hz), 104.6 (d, *J* = 3.9 Hz), 95.6 (d, *J* = 4.1 Hz), 93.6 (d, *J* = 4.4 Hz), 80.2 (d, *J* = 3.6 Hz), 71.2, 71.0, 69.9, 69.7, 40.4, 34.9, 33.2, 32.9, 31.6, 28.5, 20.8, 20.4, 11.1.

IR (ATR) 2962, 2926, 2867, 2825, 1740, 1529, 1451, 1370, 1347, 1319, 1262, 1137, 1091, 1015, 869, 844, 715.

HRMS (ESI/QTOF) m/z: $[M]^+$ calculated for $[C_{27}H_{32}NO_4Rh]^+$: 537.1381; found: 537.1385.

m.p. 153.5 – 154.1 °C (racemate).

R*f* 0.46 (silica gel, 50:1 pentane / EtOAc, UV / CAM).

HPLC CHIRALPAK® IB, 99.5:0.5 hexane / IPA, rate 1 mL / min, 15 min, 254 nm, t_R minor 5.29 min; t_R major 5.84 min.

Opt. Rot. $[\alpha]_D^{20}$ −60.38 (c = 0.78 in $CHCl_3$, 81% ee).

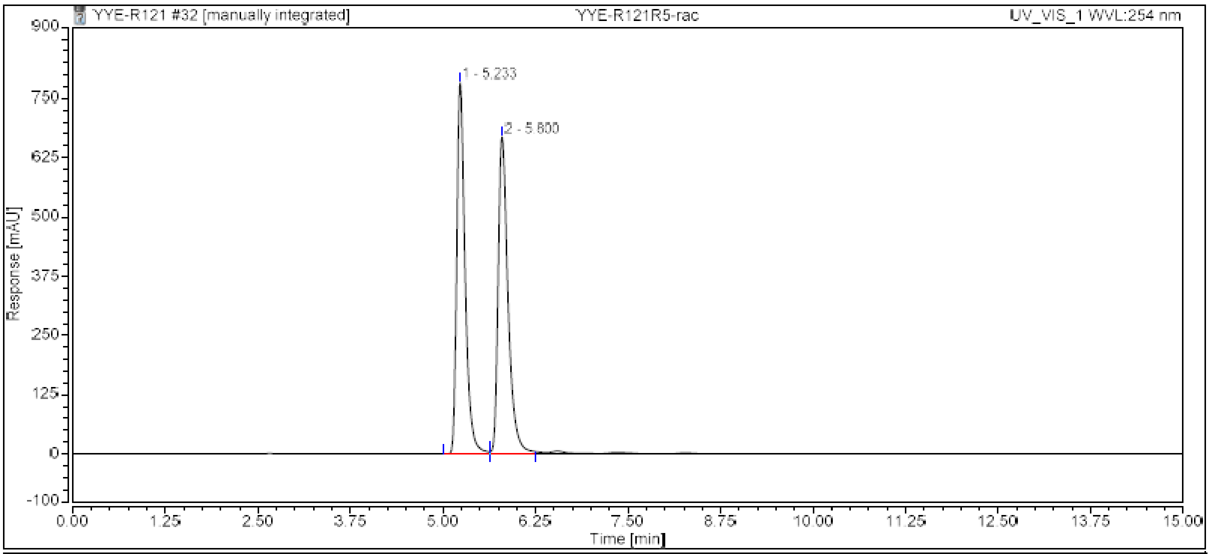

Integration Results

No.	Peak Name	Retention Time min	Area mAU*min	Height mAU	Relative Area %	Relative Height %	Amount n.a.
1		5.233	102.710	783.295	49.69	53.96	n.a.
2		5.800	103.989	668.290	50.31	46.04	n.a.
**Total:**			**206.699**	**1451.585**	**100.00**	**100.00**	

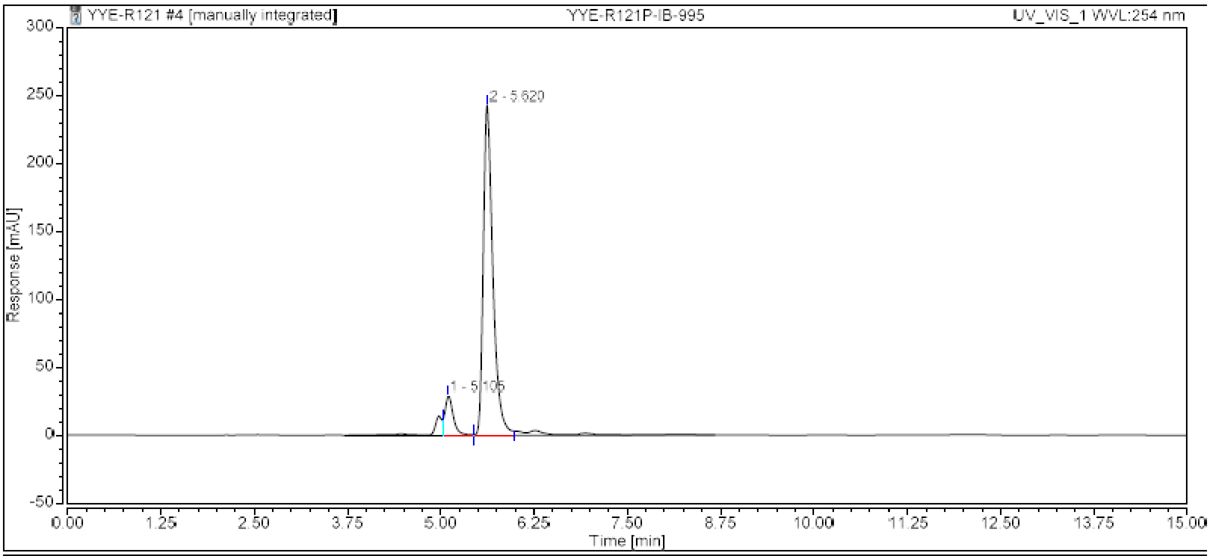

Integration Results

No.	Peak Name	Retention Time min	Area mAU*min	Height mAU	Relative Area %	Relative Height %	Amount n.a.
1		5.105	3.796	29.177	9.36	10.73	n.a.
2		5.620	36.776	242.637	90.64	89.27	n.a.
**Total:**			**40.572**	**271.813**	**100.00**	**100.00**	

After recrystallization and separation of racemate:

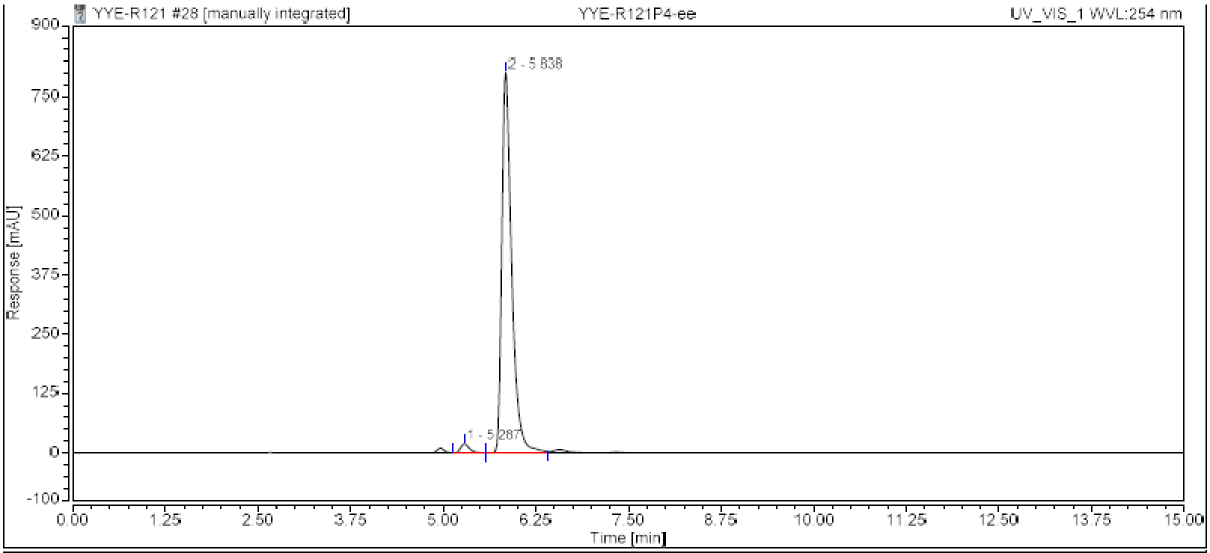

Integration Results

No.	Peak Name	Retention Time min	Area mAU*min	Height mAU	Relative Area %	Relative Height %	Amount n.a.
1		5.287	2.673	19.084	2.10	2.32	n.a.
2		5.838	124.756	801.737	97.90	97.68	n.a.
**Total:**			**127.430**	**820.821**	**100.00**	**100.00**	

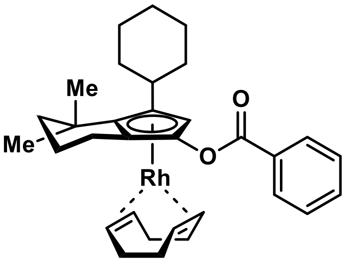

2j

Rh(I) complex **2j** was prepared via general procedure **F** with **8g** (8.8 mg, 25 µmol, 89% ee), $[Rh(COD)OAc]_2$ (6.9 mg, 13 µmol), temperature 23 °C, duration 3 h and purifying by preparatory TLC (50:1 pentane / EtOAc) to give a yellow oil (13.1 mg, 93% yield, 89% ee). Yellow crystals suitable for X-ray analysis were obtained by cooling a saturated solution of *rac*-**2j** in pentane to −20 °C.

^{1}H NMR (400 MHz, C_6D_6) δ 8.23 – 8.14 (m, 2H), 7.07 (t, *J* = 7.3 Hz, 1H), 7.01 (t, *J* = 7.3 Hz, 2H), 5.78 (s, 1H), 3.87 – 3.69 (m, 2H), 3.52 – 3.38 (m, 2H), 2.47 – 2.30 (m, 5H), 2.14 – 1.99 (m, 5H), 1.98 – 1.80 (m, 6H), 1.71 – 1.57 (m, 5H), 1.54 – 1.31 (m, 8H), 1.19 – 1.09 (m, 2H).

^{13}C NMR (101 MHz, C_6D_6) δ 163.4, 133.3, 130.6, 130.0, 128.8, 120.7 (d, *J* = 4.3 Hz), 106.5 (d, *J* = 4.3 Hz), 103.9 (d, *J* = 4.5 Hz), 91.0 (d, *J* = 3.7 Hz), 76.9 (d, *J* = 3.4 Hz), 69.4, 69.2, 68.1, 67.9, 42.0, 38.5, 37.6, 34.7, 34.3, 32.9, 32.6, 31.6, 29.7, 27.6, 27.4, 26.3, 20.0, 19.7.

IR (ATR) 2928, 2852, 2824, 1739, 1449, 1261, 1176, 1123, 1103, 1064, 1026, 704.

HRMS (ESI/QTOF) m/z: $[M]^+$ calculated for $[C_{32}H_{41}O_2Rh]^+$: 560.2156; found: 560.2157.

Rf 0.78 (silica gel, 50:1 pentane / EtOAc, UV / CAM).

HPLC CHIRALPAK® IF, 99.5:0.5 hexane / IPA, rate 0.5 mL / min, 25 min, 254 nm, t_R major 11.79 min; t_R minor 13.68 min.

Opt. Rot. $[\alpha]_D^{20}$ −22.60 (c = 0.73 in $CHCl_3$, 89% ee).

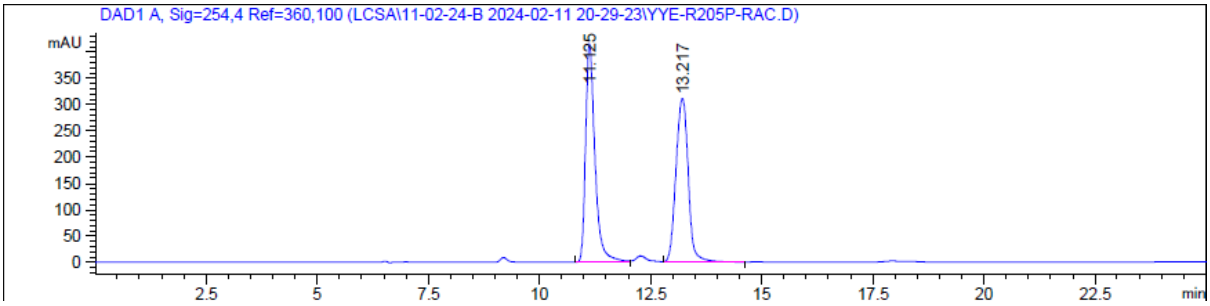

Signal 1: DAD1 A, Sig=254,4 Ref=360,100

Peak #	RetTime [min]	Type	Width [min]	Area [mAU*s]	Height [mAU]	Area %
1	11.125	BV	0.2216	6092.48975	414.32294	50.3962
2	13.217	VB	0.3066	5996.70117	311.55005	49.6038

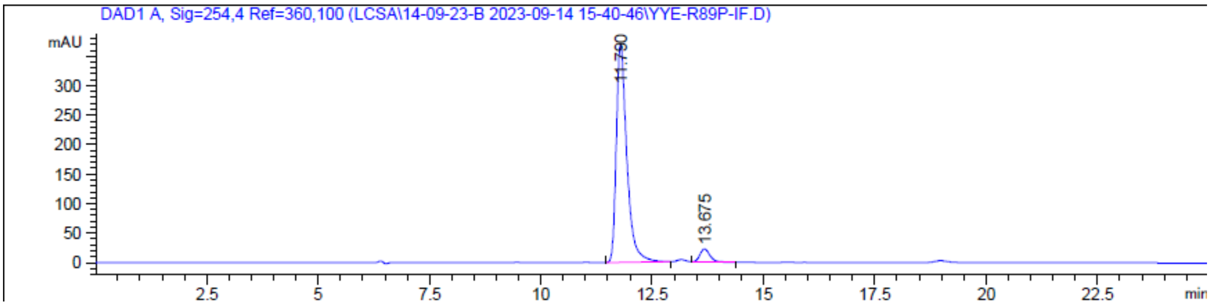

Signal 1: DAD1 A, Sig=254,4 Ref=360,100

Peak #	RetTime [min]	Type	Width [min]	Area [mAU*s]	Height [mAU]	Area %
1	11.790	BB	0.2451	6053.88770	369.57404	94.4990
2	13.675	VB	0.2421	352.41232	22.33358	5.5010

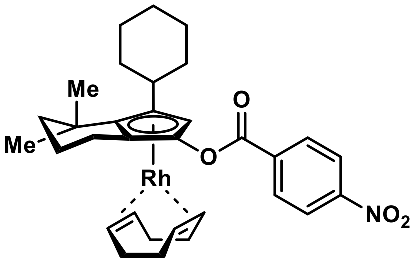

2k

Rh(I) complex **2k** was prepared via general procedure **F** with **8h** (5.0 mg, 13 µmol, 99% ee), $[Rh(COD)OAc]_2$ (3.5 mg, 13 µmol), temperature 23 °C, duration 16 h and purifying by column chromatography (50:1 pentane / EtOAc) to give a dark purple oil (7.2 mg, 94% yield, 99% ee). Yellow crystals suitable for X-ray analysis were obtained by cooling a saturated solution of *rac*-**2k** in EtOAc to −20 °C.

^{1}H NMR (400 MHz, C_6D_6) δ 7.78 (d, *J* = 8.8 Hz, 2H), 7.64 (d, *J* = 8.8 Hz, 2H), 5.93 (s, 1H), 3.87 – 3.78 (m, 2H), 3.76 – 3.64 (m, 2H), 2.49 – 2.21 (m, 6H), 2.21 – 2.10 (m, 2H), 2.05 (d, *J* = 8.1 Hz, 1H), 1.98 – 1.83 (m, 3H), 1.73 – 1.55 (m, 9H), 1.48 (d, *J* = 12.3 Hz, 1H), 1.44 – 1.37 (m, 1H), 1.29 (s, 3H), 1.25 – 1.04 (m, 5H).

^{13}C NMR (101 MHz, C_6D_6) δ 161.5, 150.7, 134.9, 130.5, 123.7, 120.4 (d, *J* = 4.5 Hz), 106.9 (d, *J* = 4.4 Hz), 104.3 (d, *J* = 4.3 Hz), 90.6 (d, *J* = 3.6 Hz), 76.7 (d, *J* = 3.3 Hz), 69.6, 69.4, 68.3, 68.2, 41.9, 38.5, 37.6, 34.7, 34.2, 32.9, 32.6, 31.6, 29.6, 27.5, 27.4, 26.3, 20.0, 19.6.

IR (ATR) 2926, 2852, 1739, 1529, 1448, 1346, 1319, 1259, 1238, 1215, 1144, 1123, 1098, 1071, 1014, 866, 844, 797, 755, 713.

HRMS (ESI/QTOF) m/z: $[M]^+$ calculated for $[C_{32}H_{40}NO_4Rh]^+$: 605.2007; found: 605.2009.

m.p. 213.5 – 214.4 °C (racemate).

R*f* 0.50 (silica gel, 50:1 pentane / EtOAc, UV / CAM).

HPLC CHIRALPAK® IA, 95:5 hexane / IPA, rate 1 mL / min, 15 min, 254 nm, t_R major 3.08 min; t_R minor 3.54 min.

Opt. Rot. $[\alpha]_D^{20}$ −235.80 (c = 0.25 in $CHCl_3$, 99% ee).

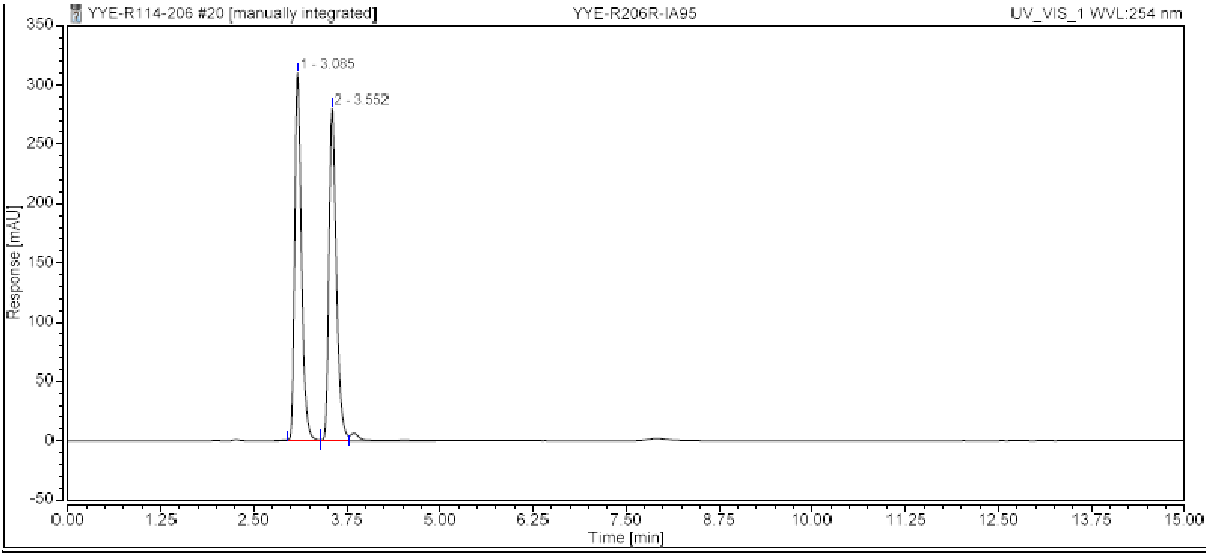

Integration Results

No.	Peak Name	Retention Time min	Area mAU*min	Height mAU	Relative Area %	Relative Height %	Amount n.a.
1		3.085	32.532	310.057	50.17	52.55	n.a.
2		3.552	32.315	279.978	49.83	47.45	n.a.
**Total:**			**64.847**	**590.035**	**100.00**	**100.00**	

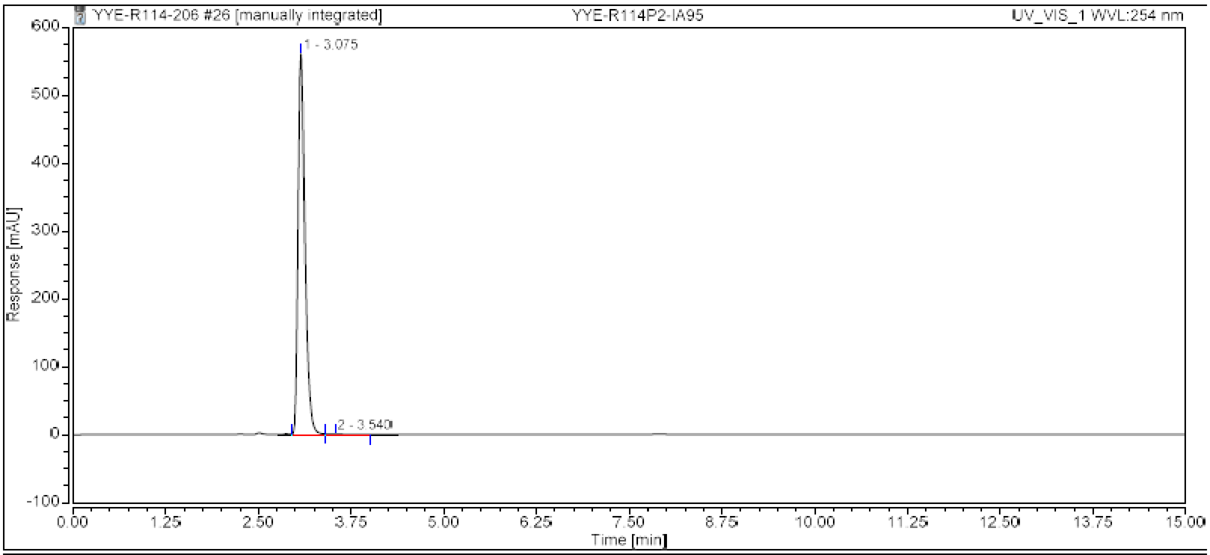

Integration Results

No.	Peak Name	Retention Time min	Area mAU*min	Height mAU	Relative Area %	Relative Height %	Amount n.a.
1		3.075	58.212	561.020	99.66	99.83	n.a.
2		3.540	0.199	0.960	0.34	0.17	n.a.
**Total:**			**58.410**	**561.980**	**100.00**	**100.00**	

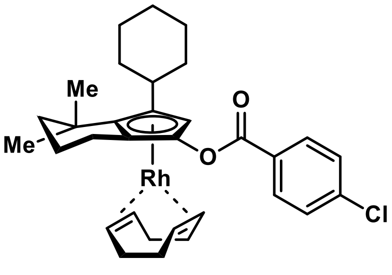

2l

Rh(I) complex **2l** was prepared via general procedure **F** with **8l** (6.0 mg, 16 μmol, 90% ee),

$[Rh(COD)OAc]_2$ (4.5 mg, 8.3 µmol), temperature 23 °C, duration 3 h and purifying by column chromatography (50:1 pentane / EtOAc) to give a yellow oil (9.0 mg, 92% yield, 90% ee). Yellow crystals suitable for X-ray analysis were obtained by cooling a saturated solution of *rac*-**2l** in pentane to −20 °C.

^{1}H NMR (400 MHz, C_6D_6) δ 7.89 (d, *J* = 8.6 Hz, 2H), 6.98 (d, *J* = 8.6 Hz, 2H), 5.91 (s, 1H), 3.85 – 3.77 (m, 2H), 3.77 – 3.70 (m, 2H), 2.49 – 2.38 (m, 2H), 2.37 – 2.22 (m, 4H), 2.21 – 2.11 (m, 2H), 2.04 (d, *J* = 8.3 Hz, 1H), 1.96 – 1.83 (m, 3H), 1.73 – 1.55 (m, 9H), 1.49 (d, *J* = 12.9 Hz, 1H), 1.43 – 1.37 (m, 1H), 1.28 (s, 3H), 1.25 – 1.06 (m, 5H).

^{13}C NMR (101 MHz, C_6D_6) δ 162.4, 139.8, 131.3, 129.1, 128.9, 120.6 (d, *J* = 4.5 Hz), 106.6 (d, *J* = 4.3 Hz), 104.0 (d, *J* = 4.5 Hz), 90.8 (d, *J* = 3.7 Hz), 76.8 (d, *J* = 3.3 Hz), 69.4, 69.3, 68.1, 68.0, 41.9, 38.5, 37.6, 34.7, 34.3, 32.9, 32.6, 31.6, 29.6, 27.5, 27.4, 26.3, 20.0, 19.6.

IR (ATR) 2928, 2852, 2824, 1739, 1593, 1488, 1449, 1260, 1238, 1171, 1124, 1092, 1071, 1015, 846, 752.

HRMS (ESI/QTOF) m/z: $[M]^+$ calculated for $[C_{32}H_{40}ClO_2Rh]^+$: 594.1766; found: 594.1778.

m.p. 160.0 – 161.0 °C (racemate).

Rf 0.41 (silica gel, 50:1 pentane / EtOAc, UV / CAM).

HPLC CHIRALPAK® IF, 99.5:0.5 hexane / IPA, rate 1 mL / min, 25 min, 254 nm, t_R major 5.89 min; t_R minor 6.86 min.

Opt. Rot. $[\alpha]_D^{20}$ −31.16 (c = 0.69 in $CHCl_3$, 90% ee).

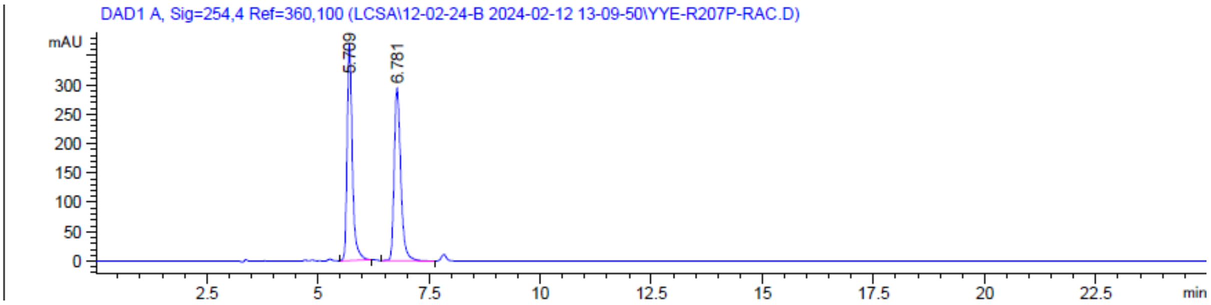

Signal 1: DAD1 A, Sig=254,4 Ref=360,100

Peak #	RetTime [min]	Type	Width [min]	Area [mAU*s]	Height [mAU]	Area %
1	5.709	BB	0.1226	2994.38428	369.37857	49.7967
2	6.781	BB	0.1560	3018.83643	293.57486	50.2033

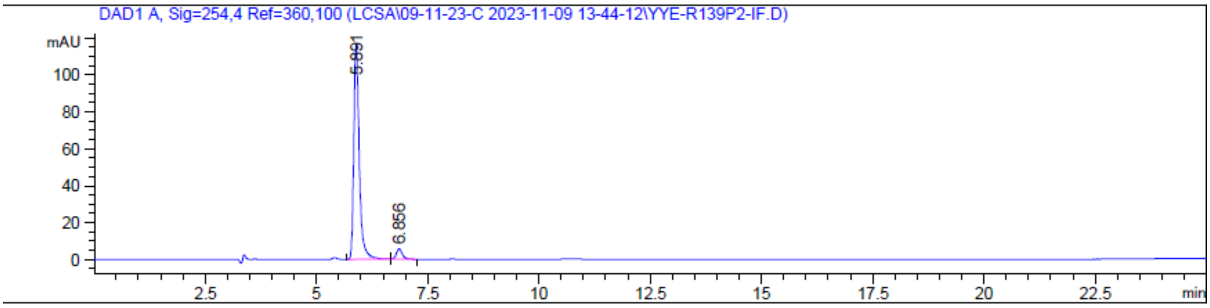

Signal 1: DAD1 A, Sig=254,4 Ref=360,100

Peak #	RetTime [min]	Type	Width [min]	Area [mAU*s]	Height [mAU]	Area %
1	5.891	BB	0.1271	990.87982	116.59811	95.0226
2	6.856	BB	0.1400	51.90296	5.60863	4.9774

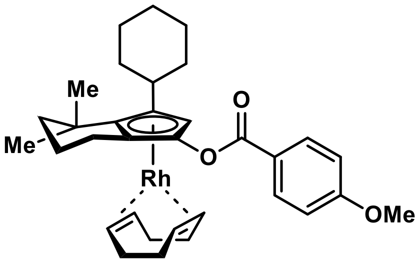

2m

Rh(I) complex **2m** was prepared via general procedure **F** with **8j** (4.9 mg, 13 µmol, 86% ee), $[Rh(COD)OAc]_2$ (3.5 mg, 6.6 µmol), temperature 23 °C, duration 3 h and purifying by column chromatography (50:1 pentane / EtOAc) to give a yellow oil (2.8 mg, 37% yield, 86% ee). Yellow crystals suitable for X-ray analysis were obtained by cooling a saturated solution of *rac*-**2m** in pentane to −20 °C.

^{1}H NMR (400 MHz, C_6D_6) δ 8.20 (d, *J* = 8.9 Hz, 2H), 6.62 (d, *J* = 8.9 Hz, 2H), 5.94 (s, 1H), 3.89 – 3.76 (m, 4H), 3.14 (s, 3H), 2.54 – 2.43 (m, 2H), 2.41 – 2.23 (m, 4H), 2.23 – 2.14 (m, 2H), 2.06 (d, *J* = 7.8 Hz, 1H), 2.03 – 1.94 (m, 1H), 1.94 – 1.84 (m, 2H), 1.76 – 1.55 (m, 9H), 1.52 (d, *J* = 11.6 Hz, 1H), 1.45 – 1.35 (m, 1H), 1.29 (s, 3H), 1.27 – 1.02 (m, 5H).

^{13}C NMR (101 MHz, C_6D_6) δ 164.0, 163.2, 132.2, 123.0, 120.9 (d, *J* = 4.6 Hz), 114.2, 106.4 (d, *J* = 4.3 Hz), 103.8 (d, *J* = 4.5 Hz), 91.1 (d, *J* = 3.6 Hz), 77.0 (d, *J* = 3.4 Hz), 69.3, 69.2, 68.0, 67.9, 54.9, 42.0, 38.5, 37.7, 34.7, 34.4, 32.9, 32.6, 31.6, 29.7, 27.6, 27.5, 26.4, 20.1, 19.7.

IR (ATR) 2926, 2851, 2823, 1732, 1606, 1510, 1449, 1315, 1254, 1166, 1144, 1123, 1098, 1068, 1030, 862, 845, 803, 760, 691.

HRMS (ESI/QTOF) m/z: $[M]^+$ calculated for $[C_{33}H_{43}O_3Rh]^+$: 590.2262; found: 590.2262.

m.p. 144.6 – 145.5 °C (racemate).

Rf 0.41 (silica gel, 50:1 pentane / EtOAc, UV / CAM).

HPLC CHIRALPAK® IC, 99.5:0.5 hexane / IPA, rate 1 mL / min, 25 min, 230 nm, t_R major 7.36 min; t_R minor 8.60 min.

Opt. Rot. $[\alpha]_D^{20}$ −53.86 (c = 0.69 in $CHCl_3$, 86% ee).

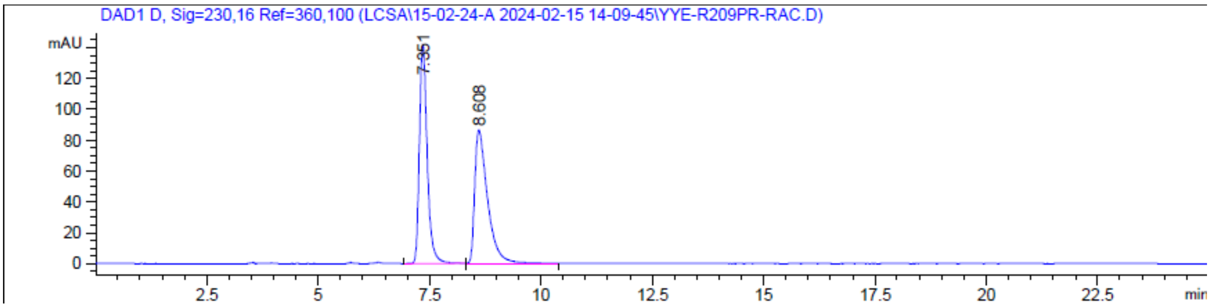

Signal 3: DAD1 D, Sig=230,16 Ref=360,100

Peak #	RetTime [min]	Type	Width [min]	Area [mAU*s]	Height [mAU]	Area %
1	7.351	BV	0.1848	1737.82275	141.97096	50.0533
2	8.608	VB	0.2975	1734.12402	86.63348	49.9467

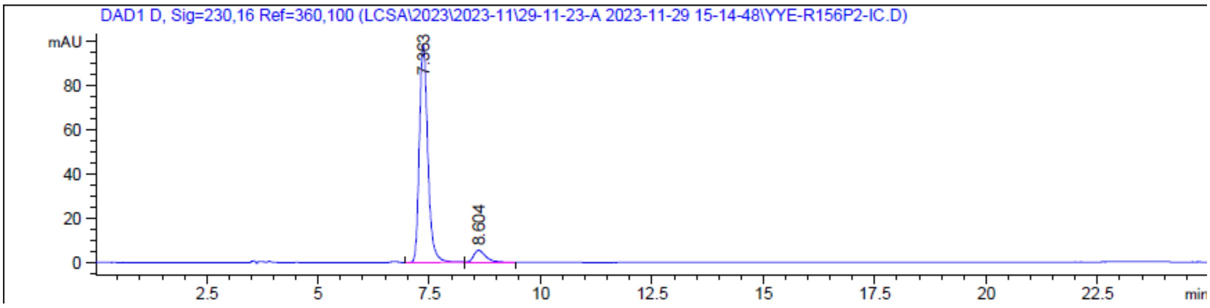

Signal 3: DAD1 D, Sig=230,16 Ref=360,100

Peak #	RetTime [min]	Type	Width [min]	Area [mAU*s]	Height [mAU]	Area %
1	7.363	BB	0.2040	1302.52759	98.67425	92.9341
2	8.604	BB	0.2786	99.03345	5.38430	7.0659

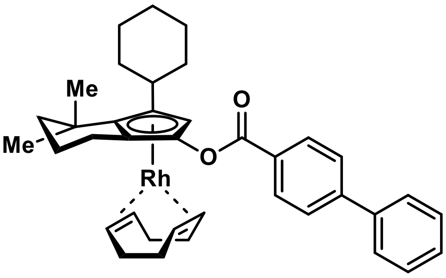

2n

Rh(I) complex **2n** was prepared via general procedure **F** with **8k** (18.7 mg, 43.8 µmol, 90% ee),

$[Rh(COD)OAc]_2$ (12.0 mg, 22.4 µmol), temperature 23 °C, duration 3 h and purifying by preparatory TLC (50:1 pentane / EtOAc) to give a yellow oil (9.5 mg, 34% yield, 90% ee). Orange crystals (>99% ee) suitable for X-ray analysis were obtained by cooling a saturated solution of **2n** (90% ee) in pentane to −20 °C.

^{1}H NMR (400 MHz, C_6D_6) δ 8.27 (d, J = 8.3 Hz, 2H), 7.40 – 7.33 (m, 4H), 7.21 – 7.17 (m, 2H), 7.14 – 7.10 (m, 1H), 5.98 (s, 1H), 3.90 – 3.78 (m, 4H), 2.55 – 2.24 (m, 6H), 2.24 – 2.14 (m, 2H), 2.11 – 1.97 (m, 2H), 1.95 – 1.85 (m, 2H), 1.75 – 1.57 (m, 9H), 1.52 (d, J = 11.9 Hz, 1H), 1.45 – 1.38 (m, 1H), 1.30 (s, 3H), 1.26 – 1.05 (m, 5H).

^{13}C NMR (101 MHz, C_6D_6) δ 163.3, 146.3, 140.3, 130.7, 129.3, 129.2, 127.6, 120.8 (d, J = 4.5 Hz), 106.6 (d, J = 4.2 Hz), 104.0 (d, J = 4.3 Hz), 91.0 (d, J = 3.8 Hz), 77.0 (d, J = 3.4 Hz), 69.4, 69.3, 68.1, 68.0, 42.0, 38.5, 37.7, 34.7, 34.3, 32.9, 32.6, 31.6, 29.7, 27.6, 27.5, 26.4, 20.1, 19.7.

IR (ATR) 2927, 2852, 2824, 1736, 1608, 1449, 1262, 1187, 1178, 1124, 1100, 1071, 1008, 858, 744, 697.

HRMS (ESI/QTOF) m/z: $[M]^+$ calculated for $[C_{38}H_{45}O_2Rh]^+$: 636.2469; found: 636.2485.

m.p. 179.3 – 181.4 °C.

Rf 0.36 (silica gel, 50:1 pentane / EtOAc, UV / CAM).

HPLC CHIRALPAK® IF, 99.5:0.5 hexane / IPA, rate 1 mL / min, 25 min, 254 nm, t_R major 9.78 min; t_R minor 11.86 min.

Opt. Rot. $[\alpha]_D^{20}$ −68.75 (c = 0.72 in $CHCl_3$, 90% ee).

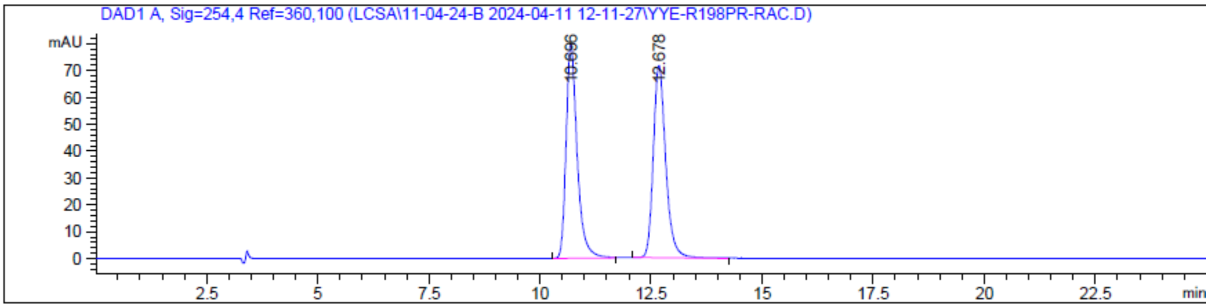

Signal 1: DAD1 A, Sig=254,4 Ref=360,100

Peak #	RetTime [min]	Type	Width [min]	Area [mAU*s]	Height [mAU]	Area %
1	10.696	BV	0.2622	1379.61768	79.59528	49.8333
2	12.678	BB	0.2946	1388.84949	71.50230	50.1667

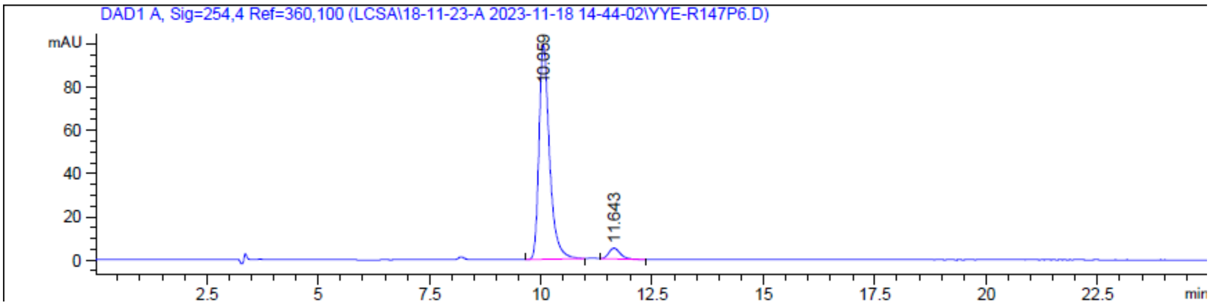

Signal 1: DAD1 A, Sig=254,4 Ref=360,100

Peak #	RetTime [min]	Type	Width [min]	Area [mAU*s]	Height [mAU]	Area %
1	10.059	BB	0.2473	1634.16113	99.64430	94.8552
2	11.643	BB	0.2682	88.63354	5.01363	5.1448

After recrystallization:

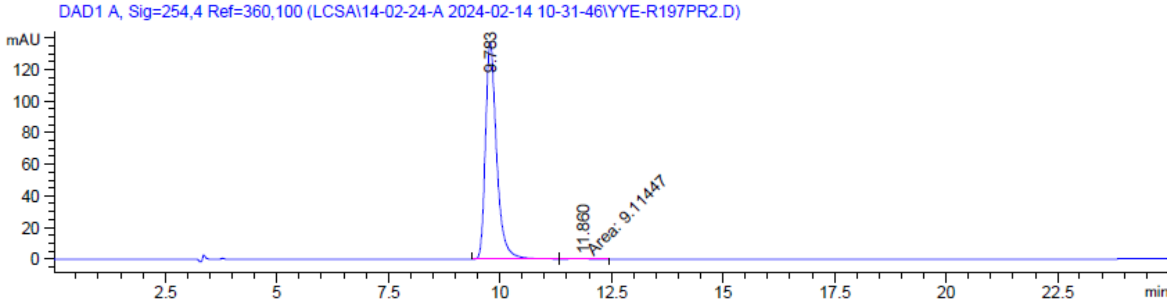

Signal 1: DAD1 A, Sig=254,4 Ref=360,100

Peak #	RetTime [min]	Type	Width [min]	Area [mAU*s]	Height [mAU]	Area %
1	9.783	BB	0.2630	2392.86108	137.54237	99.6205
2	11.860	MM T	0.5055	9.11447	3.00539e-1	0.3795

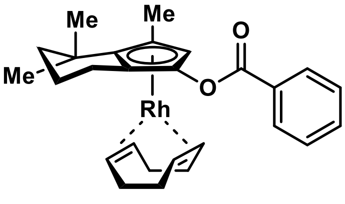

2o

Rh(I) complex **2o** was prepared via general procedure **F** with **8l** (13.3 mg, 47.1 µmol, 98% ee), $[Rh(COD)OAc]_2$ (12.9 mg, 24.0 µmol), temperature 23 °C, duration 3 h and purifying by column chromatography (50:1 pentane / EtOAc) to give a yellow oil (20.0 mg, 86% yield, 98% ee).

^{1}H NMR (400 MHz, C_6D_6) δ 8.20 – 8.11 (m, 2H), 7.13 – 6.97 (m, 3H), 5.82 (s, 1H), 3.61 – 3.52 (m, 2H), 3.52 – 3.42 (m, 2H), 2.47 – 2.30 (m, 4H), 2.30 – 2.19 (m, 1H), 2.11 – 2.01 (m, 4H), 1.90 (dt, *J* = 15.8, 6.5 Hz, 1H), 1.77 – 1.50 (m, 6H), 1.47 (s, 3H), 1.42 – 1.34 (m, 1H), 1.16 (s, 3H).

^{13}C NMR (101 MHz, C_6D_6) δ 163.6, 133.3, 130.4, 130.1, 128.7, 120.5 (d, *J* = 4.3 Hz), 108.8 (d, *J* = 4.5 Hz), 94.0 (d, *J* = 3.4 Hz), 87.3 (d, *J* = 4.6 Hz), 79.9 (d, *J* = 3.4 Hz), 70.2, 70.03, 69.95, 69.8, 41.5, 34.4, 33.2, 33.0, 32.0, 28.8, 20.9, 20.2, 12.6.

IR (ATR) 2927, 2871, 2823, 1739, 1450, 1313, 1261, 1176, 1135, 1109, 1078, 1063, 1026, 7056.

HRMS (ESI/QTOF) m/z: $[M]^+$ calculated for $[C_{27}H_{33}O_2Rh]^+$: 492.1530; found: 492.1526.

Rf 0.50 (silica gel, 50:1 pentane / EtOAc, UV / CAM).

HPLC CHIRALPAK® IF, 99.5:0.5 hexane / IPA, rate 0.5 mL / min, 25 min, 254 nm, t_R major 13.75 min; t_R minor 16.25 min.

Opt. Rot. $[\alpha]_D^{20}$ −11.50 (c = 1.00 in $CHCl_3$, 98% ee).

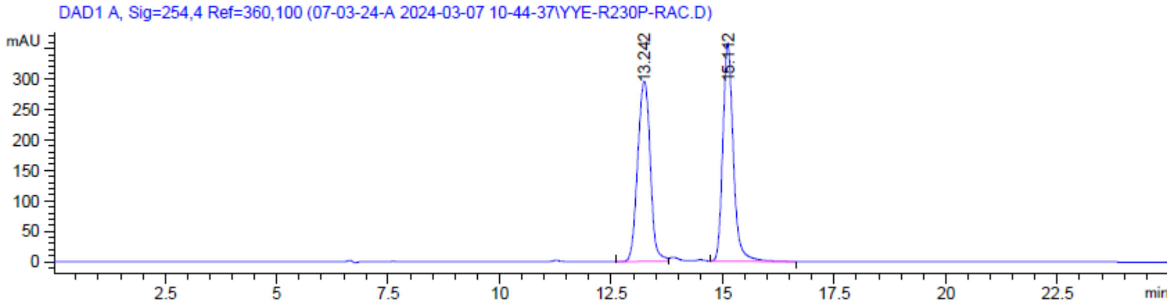

Signal 1: DAD1 A, Sig=254,4 Ref=360,100

Peak #	RetTime [min]	Type	Width [min]	Area [mAU*s]	Height [mAU]	Area %
1	13.242	BV	0.3147	5804.68311	296.17136	49.8809
2	15.112	BB	0.2500	5832.39307	358.21777	50.1191

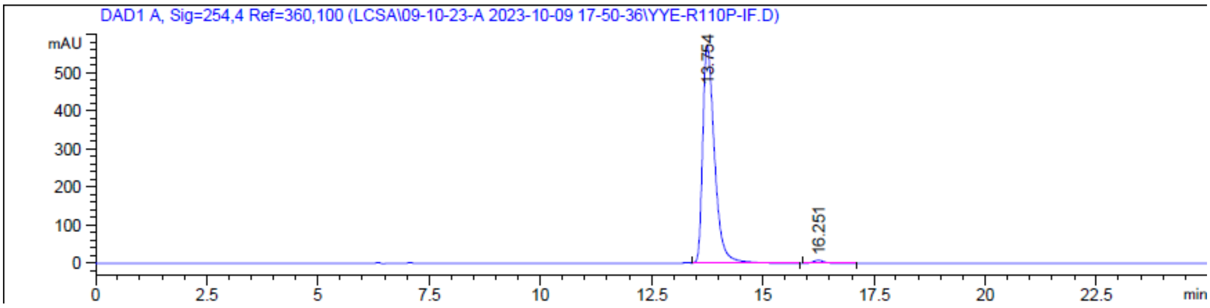

Signal 1: DAD1 A, Sig=254,4 Ref=360,100

Peak #	RetTime [min]	Type	Width [min]	Area [mAU*s]	Height [mAU]	Area %
1	13.754	VB	0.2809	1.05675e4	573.76147	98.8493
2	16.251	BB	0.2460	123.02017	7.63401	1.1507

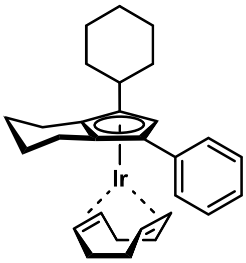

5a

Ir(I) complex **5a** was prepared via general procedure **F** with **1a** (17 mg, 57 µmol, 95% ee), $[Ir(COD)OAc]_2$ (21 mg, 29 µmol), temperature 70 °C, duration 3 h and purifying by column chromatography (pentane) to give a yellow oil (18 mg, 55% yield, 94% ee).

^{1}H NMR (400 MHz, $CDCl_3$) δ 7.36 – 7.31 (m, 2H), 7.32 – 7.23 (m, 2H), 7.19 – 7.12 (m, 1H), 5.17 (s, 1H), 3.13 – 3.03 (m, 2H), 2.99 – 2.89 (m, 2H), 2.89 – 2.76 (m, 1H), 2.71 – 2.61 (m, 1H), 2.55 – 2.44 (m, 1H), 2.37 – 2.25 (m, 1H), 2.11 – 1.91 (m, 7H), 1.86 – 1.66 (m, 8H), 1.65 – 1.56 (m, 2H), 1.42 – 1.13 (m, 6H).

^{13}C NMR (101 MHz, $CDCl_3$) δ 135.4, 128.2, 128.0, 126.0, 104.0, 97.9, 97.1, 95.4, 75.3, 53.6, 53.0, 35.4, 35.3, 34.4, 33.7, 33.4, 27.2, 27.1, 26.5, 24.0, 23.6, 22.9, 22.1.

IR (ATR) 2922, 2849, 2823, 1601, 1507, 1445, 1317, 1235, 1150, 1002, 905, 761, 695.

HRMS (ESI + APCI) m/z: $[M+H]^+$ calculated for $[C_{29}H_{38}Ir]^+$: 579.2597; found: 579.2578.

Rf 0.51 (silica gel, pentane, UV / CAM).

HPLC 2 × CHIRALPAK® IA in series, 99.5:0.5 hexane / IPA, rate 0.5 mL / min, 22 min, 254 nm, t_R major 16.62 min; t_R minor 17.45 min.

Opt. Rot. $[\alpha]_D^{20}$ −61.71 (c = 0.7 in $CHCl_3$, 94% ee).

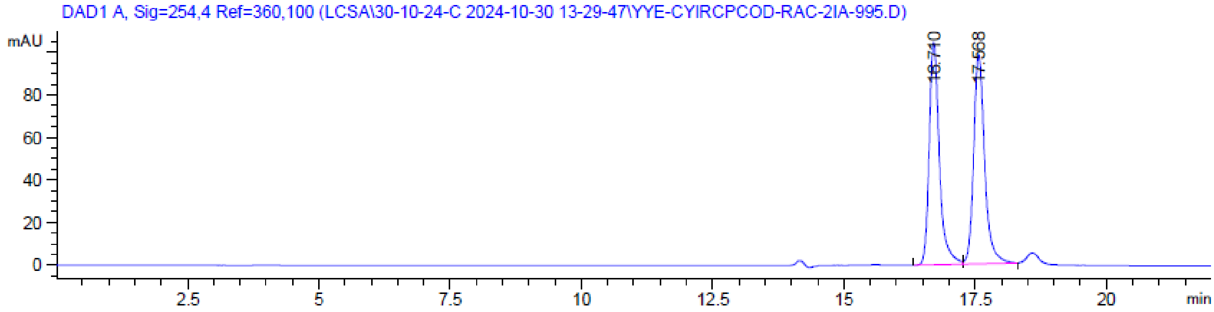

Peak #	RetTime [min]	Type	Width [min]	Area [mAU*s]	Height [mAU]	Area %
1	16.710	BV	0.2023	1397.15076	104.28061	49.5572
2	17.568	VB	0.2173	1422.11768	97.98722	50.4428

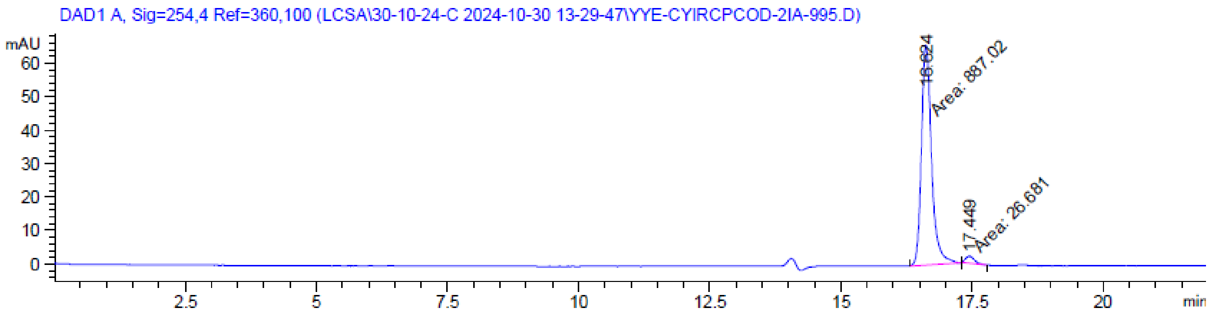

Peak #	RetTime [min]	Type	Width [min]	Area [mAU*s]	Height [mAU]	Area %
1	16.624	MM T	0.2248	887.01984	65.76748	97.0799
2	17.449	MM T	0.2061	26.68097	2.15752	2.9201

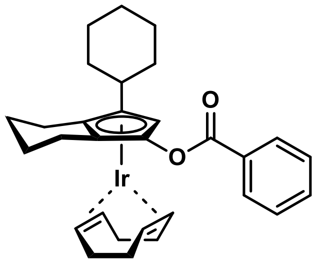

5e

Ir(I) complex **5e** was prepared via general procedure **F** with **8a** (20 mg, 62 µmol, 95% ee), $[Ir(COD)OAc]_2$ (23 mg, 32 µmol), temperature 70 °C, duration 3 h and purifying by column chromatography (100:1 pentane / EtOAc) to give a yellow solid (31 mg, 79% yield, 95% ee).

^{1}H NMR (400 MHz, C_6D_6) δ 8.22 – 8.07 (m, 2H), 7.13 – 7.06 (m, 1H), 7.04 – 6.97 (m, 2H), 5.72 (s, 1H), 3.56 – 3.47 (m, 2H), 3.45 – 3.37 (m, 2H), 2.68 – 2.54 (m, 1H), 2.45 – 2.36 (m, 1H), 2.35 – 2.17 (m, 5H), 2.16 – 1.95 (m, 6H), 1.91 – 1.80 (m, 2H), 1.78 – 1.54 (m, 5H), 1.51 – 1.38 (m, 2H), 1.29 – 1.16 (m, 3H), 1.15 – 1.05 (m, 2H).

^{13}C NMR (101 MHz, C_6D_6) δ 164.3, 133.4, 130.2, 130.0, 128.8, 115.1, 100.1, 94.0, 91.7, 70.3, 53.8, 53.8, 35.4, 35.2, 35.0, 34.3, 32.3, 27.2, 27.1, 26.6, 23.3, 23.1, 21.2, 21.1.

IR (ATR) 2924, 2850, 2824, 1740, 1449, 1398, 1314, 1261, 1176, 1135, 1083, 1062, 1026, 706.

HRMS (ESI + APCI) m/z: $[M]^+$ calculated for $[C_{30}H_{37}O_2Ir]^+$: 622.2417; found: 622.2442.

m.p. 111.1 – 112.5 °C.

Rf 0.57 (silica gel, 20:1 pentane / EtOAc, UV / CAM).

HPLC CHIRALPAK® IA, 99:1 hexane / IPA, rate 1 mL / min, 25 min, 254 nm, t_R major 4.75 min; t_R minor 5.64 min.

Opt. Rot. $[\alpha]_D^{20}$ +3.67 (c = 0.5 in $CHCl_3$, 95% ee).

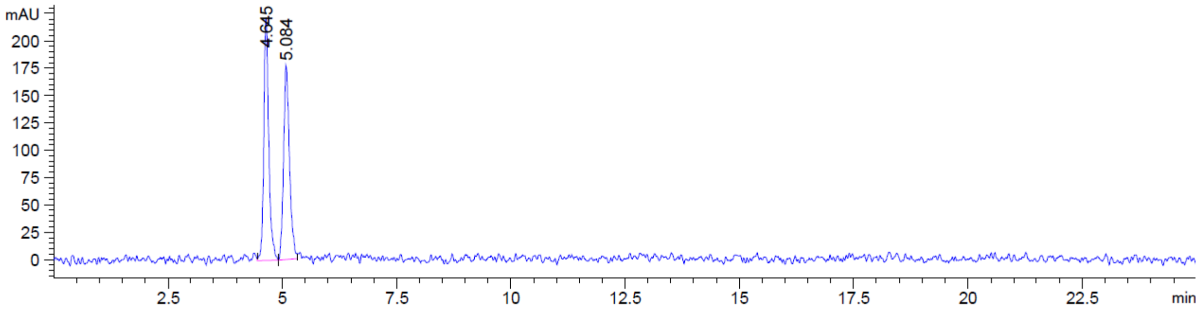

Peak #	RetTime [min]	Type	Width [min]	Area [mAU*s]	Height [mAU]	Area %
1	4.645	VB	0.1144	1684.08936	222.21608	51.3741
2	5.084	BV	0.1286	1594.00037	177.71445	48.6259

DAD1 A, Sig=254,4 Ref=360,100 (LCSA\24-10-24-B 2024-10-24 22-16-29\YYE-CYOBZIRCOD-EE-IA99-DIL.D)

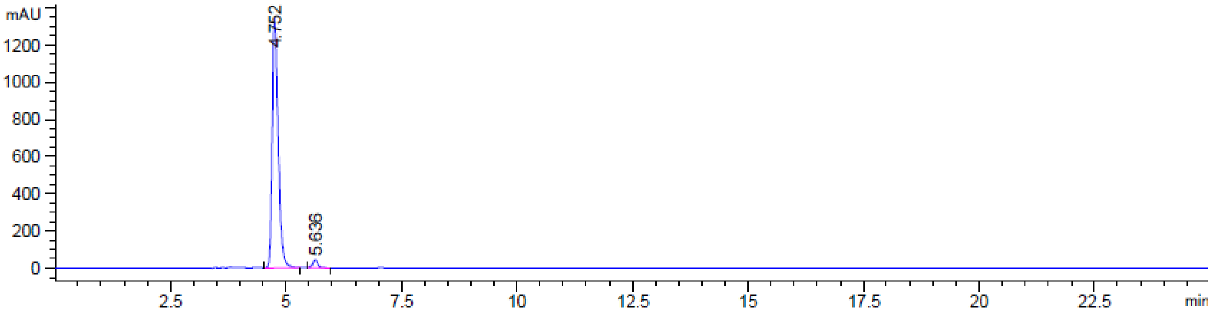

Peak #	RetTime [min]	Type	Width [min]	Area [mAU*s]	Height [mAU]	Area %
1	4.752	BB	0.1402	1.22761e4	1349.17603	97.4384
2	5.636	BV	0.1143	322.73050	42.62093	2.5616

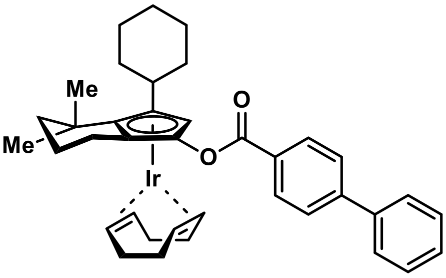

5f

Ir(I) complex **5f** was prepared according to a variation of general procedure **F**. The cyclization step was carried out on **7k** (11.1 mg, 26.0 μmol, 92% ee) as per usual. The reaction mixture was then filtered through celite (washing with pentane) and concentrated *in vacuo*. The crude cyclopentadienyl ester **8k** and $[Ir(COD)OAc]_2$ (9.5 mg, 13.3 μmol) under N_2 were dissolved in dry degassed PhMe / MeOH (130 μL). The reaction mixture was stirred in a closed vessel at 40 °C for 16 h, then filtered through a short column of silica (washing with 50:1 pentane/EtOAc). Crystallization from slow evaporation in EtOH gave **5f** as a yellow solid (8.5

mg, 45%, 87% ee). No enantioenrichment was observed in this step. Yellow crystals (racemic) suitable for X-ray analysis were obtained by cooling a saturated solution of **5f** (87% ee) in pentane to −20 °C. The resulting mother liquor was separated and concentrated to give enantioenriched **5f** as a yellow oil (99% ee).

^{1}H NMR (400 MHz, C_6D_6) δ 8.24 (d, *J* = 8.4 Hz, 2H), 7.38 – 7.34 (m, 4H), 7.21 – 7.17 (m, 2H), 7.15 – 7.11 (m, 1H), 5.97 (s, 1H), 3.73 – 3.61 (m, 4H), 2.49 – 2.20 (m, 8H), 2.14 – 2.02 (m, 2H), 1.87 – 1.78 (m, 2H), 1.71 – 1.63 (m, 3H), 1.55 (s, 7H), 1.37 – 1.32 (m, 1H), 1.30 (s, 3H), 1.22 – 1.02 (m, 5H).

^{13}C NMR (101 MHz, C_6D_6) δ 163.4, 146.4, 140.2, 130.7, 129.2, 128.9, 127.6, 118.1, 100.3, 98.9, 87.8, 73.5, 52.8, 51.3, 41.5, 39.0, 37.2, 36.1, 34.7, 32.8, 32.7, 28.9, 27.6, 26.3, 19.4, 19.2.

IR (ATR) 2925, 2850, 1737, 1608, 1449, 1262, 1240, 1177, 1124, 1099, 1071, 1008, 855, 744, 697.

HRMS (ESI/QTOF) m/z: [M + H]$^{+}$ calculated for $[C_{38}H_{46}IrO_2]^{+}$: 727.3122; found: 727.3122.

m.p. 162.1 – 162.8 °C.

R*f* 0.59 (silica gel, 50:1 pentane / EtOAc, UV / CAM).

HPLC CHIRALPAK® IF, 99.5:0.5 hexane / IPA, rate 1 mL / min, 25 min, 254 nm, t_R major 9.18 min; t_R minor 11.41 min.

Opt. Rot. $[\alpha]_D^{20}$ −39.29 (c = 0.56 in $CHCl_3$, 87% ee).

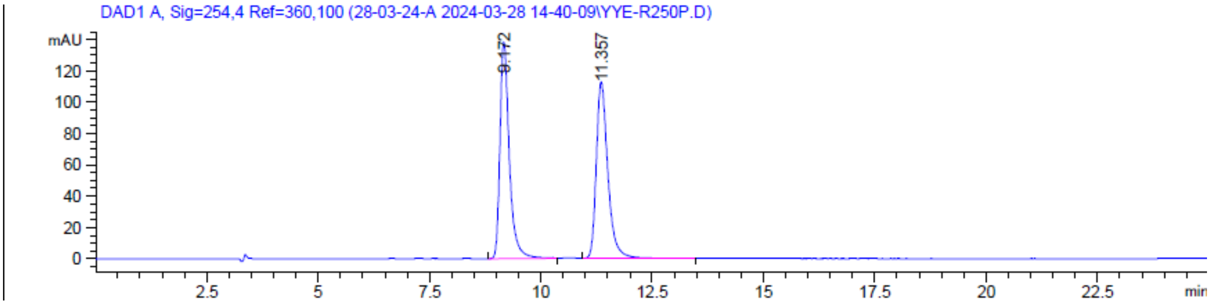

Signal 1: DAD1 A, Sig=254,4 Ref=360,100

Peak #	RetTime [min]	Type	Width [min]	Area [mAU*s]	Height [mAU]	Area %
1	9.172	BB	0.2195	2005.27075	138.03529	49.6940
2	11.357	BB	0.2720	2029.96948	112.75600	50.3060

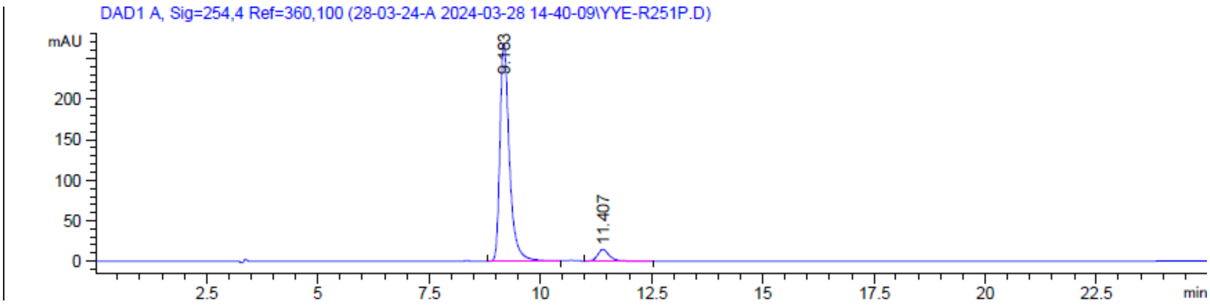

Signal 1: DAD1 A, Sig=254,4 Ref=360,100

Peak #	RetTime [min]	Type	Width [min]	Area [mAU*s]	Height [mAU]	Area %
1	9.183	BV	0.2235	3988.48486	268.20386	93.5965
2	11.407	VB	0.2842	272.87411	14.45921	6.4035

After recrystallization:

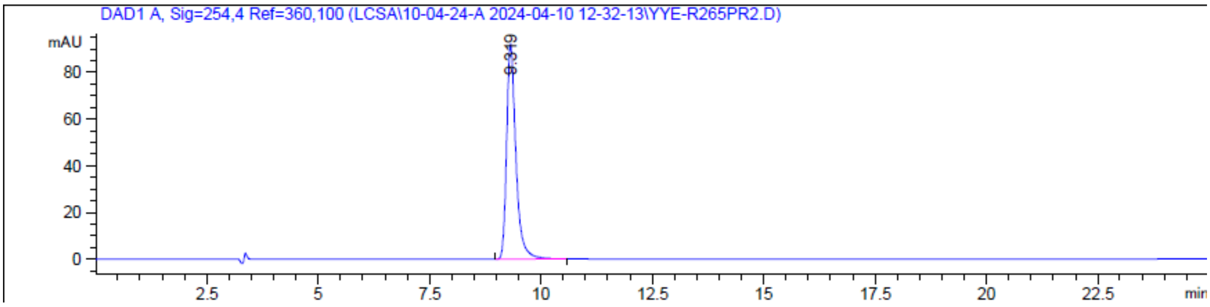

Signal 1: DAD1 A, Sig=254,4 Ref=360,100

Peak #	RetTime [min]	Type	Width [min]	Area [mAU*s]	Height [mAU]	Area %
1	9.319	BB	0.2174	1333.34448	91.82967	100.0000

General procedure G for the synthesis of [Cp^{X}Ir(C_2H_4)$_2$] complexes

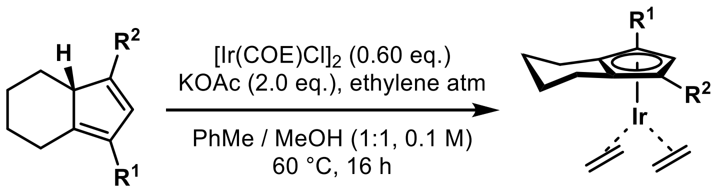

1a, **1b**, **1d** **5b**, **5c**, **5d**

A dry mixture of cyclopentadiene (1.0 eq.) and [Ir(COE)$_2$Cl]$_2$ (0.60 eq.) under N_2 was dissolved in dry degassed toluene (0.2 M) and sparged with ethylene gas for 2 min, then a solution of KOAc (2.0 eq.) in dry degassed MeOH (0.2 M relative to cyclopentadiene) was added. The reaction mixture was further sparged with ethylene gas for 2 min then heated to 60 °C in a closed vessel under ethylene for 16 h. The reaction mixture was concentrated *in vacuo* and the crude residue subjected to column chromatography (silica gel) to afford the [Cp^{X}Ir(C_2H_4)$_2$]

complex.

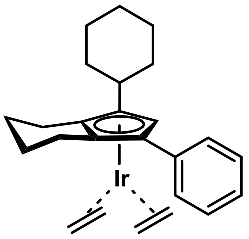

5b

Ir(I) complex **5b** was prepared via general procedure **G** with **1a** (0.15 g, 0.54 mmol, 89% ee), $[Ir(COE)_2Cl]_2$ (0.29 g, 0.32 mmol) and 10:1 pentane / PhMe eluent to give a colorless solid (0.24 g, 86% yield, 89% ee). Colorless crystals suitable for X-ray analysis were obtained by slow evaporation of *rac*-**5b** in hexane.

^{1}H NMR (400 MHz, C_6D_6) δ 7.42 – 7.30 (m, 2H), 7.21 – 7.17 (m, 2H), 7.11 – 7.02 (m, 1H), 5.13 (s, 1H), 2.73 (ddd, J = 15.6, 8.6, 5.5 Hz, 1H), 2.43 (dt, J = 15.7, 5.6 Hz, 1H), 2.27 – 2.14 (m, 3H), 2.12 – 2.05 (m, 1H), 2.04 – 1.88 (m, 5H), 1.81 – 1.60 (m, 3H), 1.58 – 1.48 (m, 1H), 1.47 – 1.38 (m, 2H), 1.36 – 1.06 (m, 10H).

^{13}C NMR (101 MHz, C_6D_6) δ 134.6, 128.7, 128.5, 126.8, 104.6, 97.7, 97.2, 96.2, 76.5, 35.3, 35.3, 33.4, 27.3, 27.1, 26.7, 25.9, 25.1, 24.2, 23.4, 22.6, 21.6.

IR (ATR) 3031, 2965, 2925, 2850, 2278, 1602, 1446, 1329, 1164, 1148, 811, 762, 696, 516.

HRMS (ESI + APCI) m/z: $[M - C_2H_4 + H]^+$ calculated for $[C_{23}H_{30}Ir]^+$: 499.1971; found: 499.1971.

m.p. 80.0 – 81.9 °C.

R*f* 0.45 (silica gel, 5:1 pentane / PhMe, UV / CAM).

HPLC CHIRALPAK® IB, 99.5:0.5 hexane / IPA, rate 0.5 mL / min, 30 min, 254 nm, t_R major 8.21 min; t_R minor 8.82 min.

Opt. Rot. $[\alpha]_D^{20}$ −70.17 (c = 1.0 in $CHCl_3$, 89% ee).

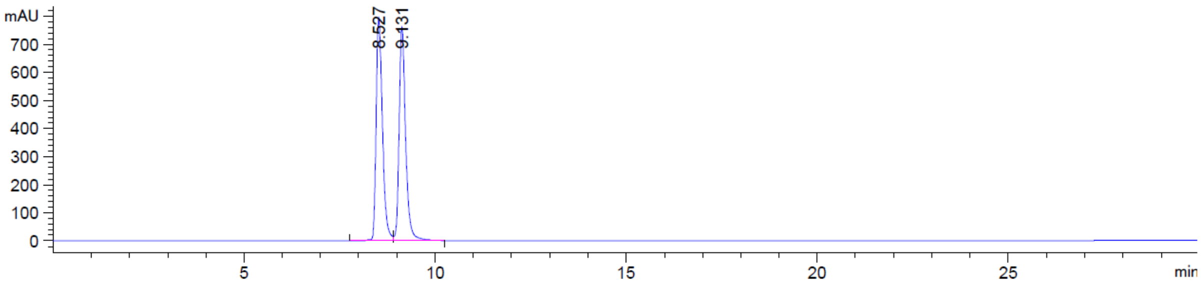

Peak #	RetTime [min]	Type	Width [min]	Area [mAU*s]	Height [mAU]	Area %
1	8.527	BV	0.1725	9027.82910	794.79089	50.0225
2	9.131	VV	0.1783	9019.71191	760.71368	49.9775

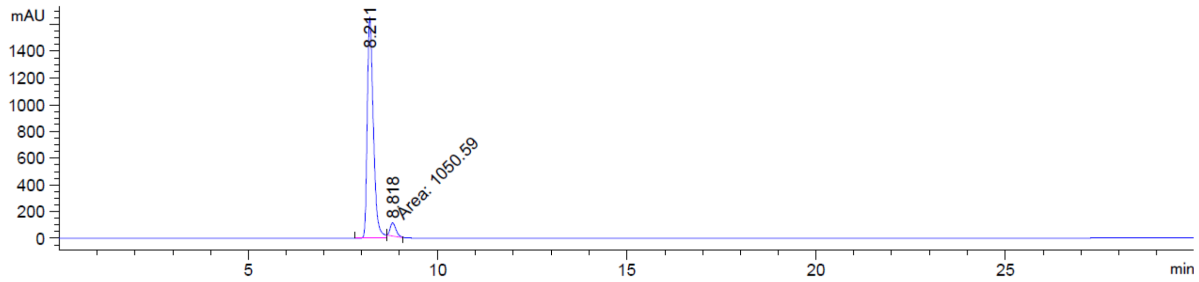

Peak #	RetTime [min]	Type	Width [min]	Area [mAU*s]	Height [mAU]	Area %
1	8.211	VV	0.1670	1.82777e4	1653.13086	94.5645
2	8.818	MM	0.1775	1050.58569	98.66489	5.4355

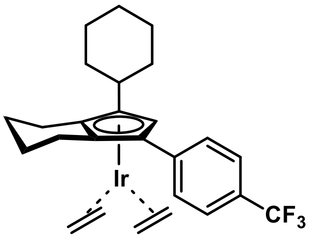

5c

Ir(I) complex **5c** was prepared via general procedure **G** with **1b** (0.20 g, 0.58 mmol, 89% ee), $[Ir(COE)_2Cl]_2$ (0.31 g, 0.35 mmol) and 10:1 pentane / DCM eluent to give a yellow oil (0.21 g, 60% yield, 88% ee as determined after conversion to derivative compound **11b**).

^{1}H NMR (400 MHz, C_6D_6) δ 7.38 (d, *J* = 8.2 Hz, 2H), 7.18 – 7.16 (m, 2H), 4.97 (s, 1H), 2.67 – 2.50 (m, 1H), 2.35 – 2.25 (m, 1H), 2.23 – 2.09 (m, 3H), 2.07 – 1.83 (m, 6H), 1.79 – 1.59 (m, 3H), 1.52 – 1.45 (m, 1H), 1.42 – 1.34 (m, 2H), 1.33 – 1.14 (m, 10H).

^{13}C NMR (101 MHz, C_6D_6) δ 138.8, 128.4 (seen in DEPT-135), 128.4 (seen in DEPT 135), 125.6 (q, J = 3.7 Hz), 105.5, 98.6, 97.6, 93.9, 76.5, 35.3, 35.2, 33.5, 27.3, 27.1, 26.7, 26.2, 25.4, 24.0, 23.2, 22.4, 21.6.

^{19}**F NMR** (376 MHz, C_6D_6) δ −62.00.

IR (ATR) 3034, 2967, 2851, 1616, 1447, 1322, 1164, 1123, 1106, 1067, 1016, 843.

HRMS (ESI + APCI) m/z: $[M - C_2H_4 + H]^+$ calculated for $[C_{24}H_{29}F_3Ir]^+$: 567.1845; found: 567.1844.

R*f* 0.26 (silica gel, pentane, UV / PMA).

Opt. Rot. $[\alpha]_D^{23}$ −64.17 (c = 0.8 in $CHCl_3$, 88% ee).

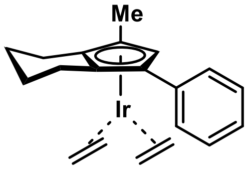

5d

Ir(I) complex **5d** was prepared via general procedure **G** with **1d** (45 mg, 0.21 mmol, 95% ee), $[Ir(COE)_2Cl]_2$ (0.12 g, 0.13 mmol) and 5:1 pentane / PhMe eluent to give a colorless solid (74 mg, 76% yield, 95% ee).

^{1}**H NMR** (400 MHz, C_6D_6) δ 7.25 – 7.21 (m, 2H), 7.19 – 7.17 (m, 1H), 7.16 – 7.13 (m, 1H), 7.08 – 7.03 (m, 1H), 5.26 (s, 1H), 2.50 (ddd, *J* = 15.6, 8.2, 5.5 Hz, 1H), 2.32 (dt, *J* = 15.7, 5.6 Hz, 1H), 2.19 (dt, *J* = 15.8, 6.1 Hz, 1H), 2.09 – 1.94 (m, 3H), 1.88 – 1.80 (m, 2H), 1.66 – 1.54 (m, 2H), 1.46 (s, 3H), 1.44 – 1.35 (m, 1H), 1.33 – 1.25 (m, 1H), 1.24 – 1.17 (m, 4H).

^{13}**C NMR** (101 MHz, C_6D_6) δ 134.4, 128.8, 127.9, 126.7, 100.8, 98.1, 96.0, 91.0, 78.1, 27.1, 25.9, 23.6, 23.4, 22.6, 21.7, 9.0.

IR (ATR) 3028, 2963, 2921, 2860, 1601, 1505, 1443, 1162, 1147, 1029, 990, 814.

HRMS (ESI + APCI) m/z: $[M - C_2H_4 + H]^+$ calculated for $[C_{18}H_{22}Ir]^+$: 431.1345; found: 431.1353.

m.p. 57.0 – 58.9 °C.

R*f* 0.15 (silica gel, 5:1 pentane / PhMe, UV / CAM).

HPLC CHIRALPAK® IB, 99.9:0.1 hexane / IPA, rate 0.5 mL / min, 25 min, 254 nm, t_R major 12.56 min; t_R minor 15.09 min.

Opt. Rot. $[\alpha]_D^{20}$ −1.00 (c = 0.5 in $CHCl_3$, 95% ee).

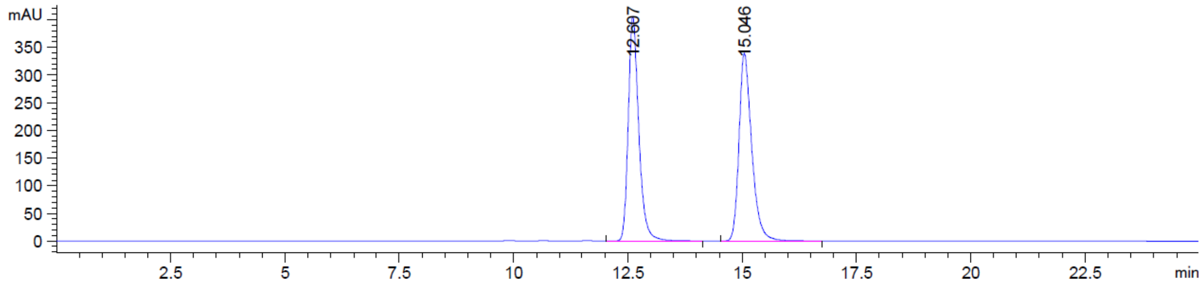

Peak #	RetTime [min]	Type	Width [min]	Area [mAU*s]	Height [mAU]	Area %
1	12.607	BB	0.2435	6514.61475	405.41690	49.7874
2	15.046	BB	0.2899	6570.24951	339.34918	50.2126

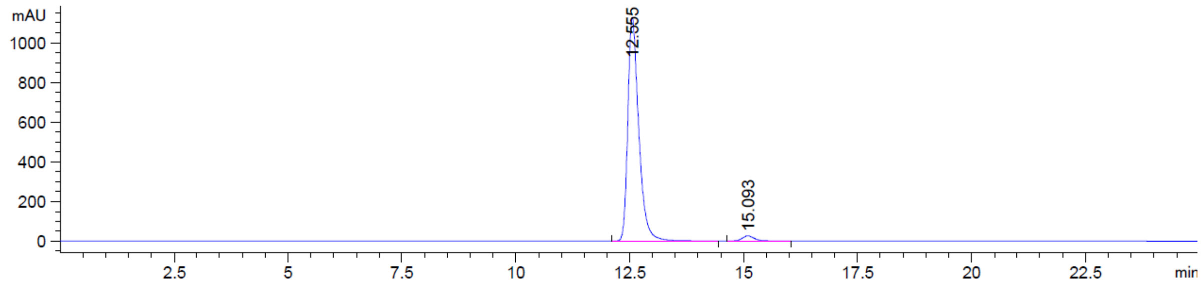

Peak #	RetTime [min]	Type	Width [min]	Area [mAU*s]	Height [mAU]	Area %
1	12.555	BB	0.2525	1.87917e4	1127.39148	97.4857
2	15.093	BB	0.2805	484.65884	26.11821	2.5143

General procedure H for the one-pot cyclization and Rh complexation of propargylic esters

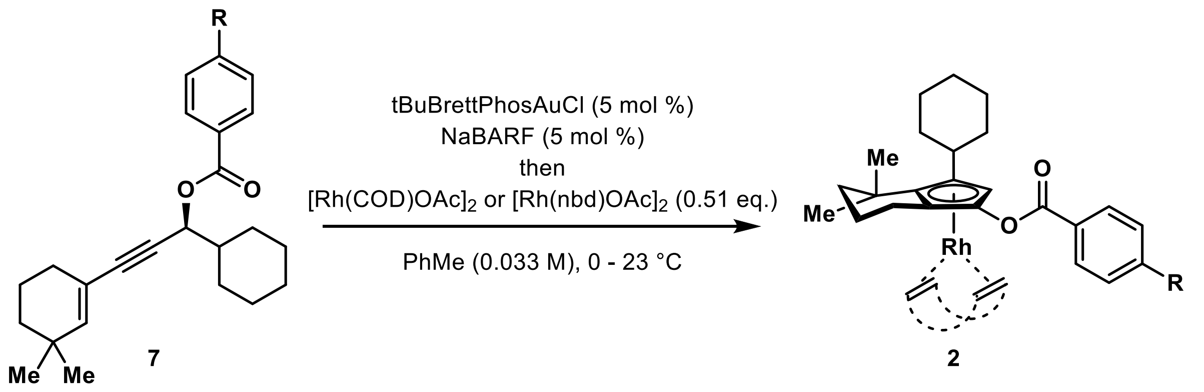

7

2

A dry mixture of gold catalyst (5 mol%) and additive (5 mol%) was suspended in dry degassed PhMe (0.1 M relative to propargylic ester) and stirred under N_2 for 5 min at 23 °C. The reaction mixture was then treated, in one portion, with a solution of the propargylic ester **7** in dry degassed PhMe (0.1 M relative to propargylic ester) and stirred for a further 3 h. The mixture was cooled to 0 °C, then a solution of $[Rh(COD)OAc]_2$ or $[Rh(nbd)OAc]_2$ (0.51 eq.) in dry degassed PhMe (0.1 M relative to propargylic ester) was added. The reaction mixture was

stirred at 0 °C for a further 30 min, then filtered through a short column of celite on silica gel, washing with 50:1 pentane / EtOAc to collect only the yellow or orange band. The crude residue was further purified to afford the pure Rh complex **2**.

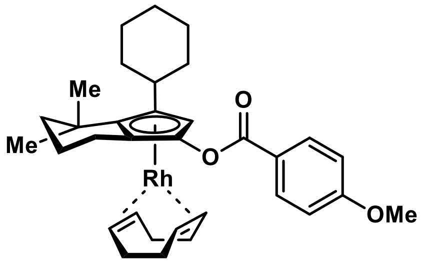

2m

Rh(I) complex **2m** was prepared via general procedure **H** with **7j** (23.1 mg, 60.7 µmol, 86% ee), $[Rh(COD)OAc]_2$ (16.5 mg, 30.7 µmol) and purifying by preparatory TLC (silica gel, 50:1 pentane / EtOAc) to give a yellow oil (21.6 mg, 60% yield, 84% ee). (see page **91** for characterization data)

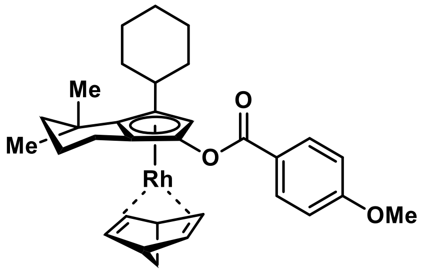

2p

Rh(I) complex **2p** was prepared via general procedure **H** with **7j** (11.7 mg, 30.1 µmol, 86% ee), $[Rh(nbd)OAc]_2$ (7.97 mg, 15.7 µmol) and purifying by column chromatography (silica gel, 50:1 pentane / EtOAc) to give a yellow oil (11.8 mg, 67% yield, 76% ee). Yellow crystals (racemic) suitable for X-ray analysis were obtained by cooling a saturated solution of **2p** (76% ee) in pentane to −20 °C. The resulting mother liquor was separated and concentrated to give enantioenriched **2p** as a yellow oil (99% ee).

^{1}H NMR (400 MHz, C_6D_6) δ 8.18 (d, *J* = 8.8 Hz, 2H), 6.63 (d, *J* = 8.8 Hz, 2H), 5.91 (s, 1H), 3.37 (s, 2H), 3.17 – 3.10 (m, 7H), 2.43 (dt, *J* = 15.4, 5.8 Hz, 1H), 2.34 – 2.25 (m, 1H), 2.25 – 2.16 (m, 1H), 2.03 (d, *J* = 8.0 Hz, 1H), 1.80 – 1.54 (m, 9H), 1.49 – 1.41 (m, 2H), 1.30 (s, 3H), 1.26 – 1.06 (m, 5H), 1.03 (s, 2H).

^{13}C NMR (101 MHz, C_6D_6) δ 163.9, 163.5, 132.3, 123.0, 120.4 (d, *J* = 5.3 Hz), 114.1, 105.6 (d, *J* = 5.0 Hz), 102.5 (d, *J* = 5.0 Hz), 90.5 (d, *J* = 4.2 Hz), 74.9 (d, *J* = 4.0 Hz), 56.9 (d, *J* = 6.8 Hz), 54.9,

47.1 (d, J = 2.6 Hz), 42.5, 37.8, 37.3, 36.4, 35.0, 33.6, 33.5, 32.3, 32.2, 32.1, 29.7, 27.8, 27.5, 26.4, 22.0, 20.4.

IR (ATR) 2927, 2850, 1732, 1606, 1511, 1454, 1315, 1298, 1255, 1167, 1145, 1124, 1097, 1069, 1031, 845, 767.

HRMS (ESI/QTOF) m/z: $[M + H]^+$ calculated for $[C_{32}H_{40}O_3Rh]^+$: 575.2027; found: 575.2035.

m.p. 176.2 – 176.7 °C (racemate).

Rf 0.27 (silica gel, 50:1 pentane / EtOAc, UV / CAM).

HPLC CHIRALPAK® IF, 99.5:0.5 hexane / IPA, rate 1 mL / min, 25 min, 254 nm, t_R major 13.64 min; t_R minor 17.42 min.

Opt. Rot. $[\alpha]_D^{20}$ −78.83 (c = 0.37 in $CHCl_3$, 99% ee).

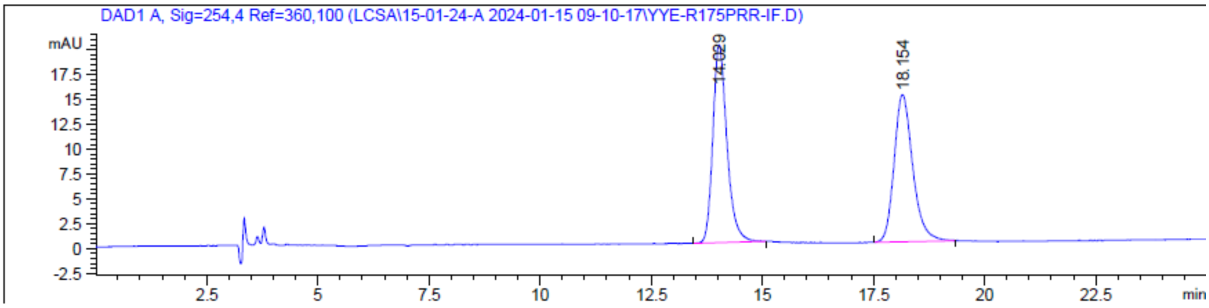

Signal 1: DAD1 A, Sig=254,4 Ref=360,100

Peak #	RetTime [min]	Type	Width [min]	Area [mAU*s]	Height [mAU]	Area %
1	14.029	BB	0.3371	438.33514	19.89132	50.3035
2	18.154	BB	0.4368	433.04501	14.82632	49.6965

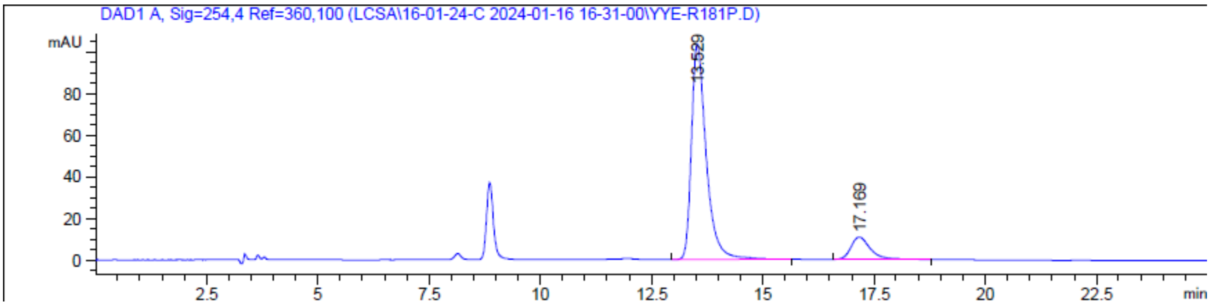

Signal 1: DAD1 A, Sig=254,4 Ref=360,100

Peak #	RetTime [min]	Type	Width [min]	Area [mAU*s]	Height [mAU]	Area %
1	13.529	BB	0.3481	2393.58154	103.37167	87.9753
2	17.169	BB	0.4484	327.16180	10.76917	12.0247

After recrystallization and separation of racemate:

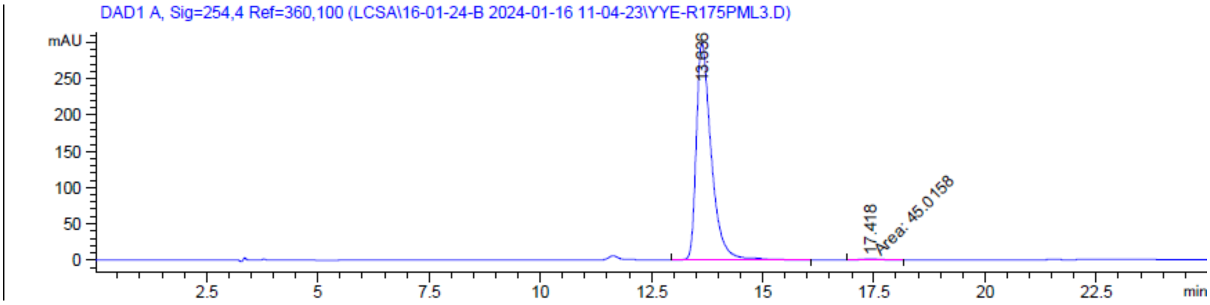

Signal 1: DAD1 A, Sig=254,4 Ref=360,100

Peak #	RetTime [min]	Type	Width [min]	Area [mAU*s]	Height [mAU]	Area %
1	13.636	BB	0.3535	7019.62109	299.43149	99.3628
2	17.418	MM T	0.4922	45.01580	1.52445	0.6372

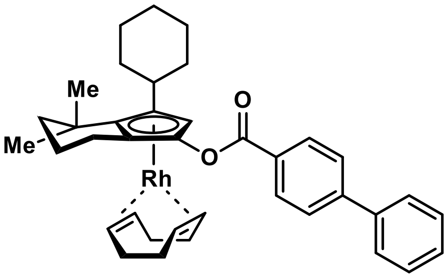

2n

Rh(I) complex **2n** was prepared via general procedure **H** with **7k** (213 mg, 0.500 mmol, 92% ee) and $[Rh(COD)OAc]_2$ (134 mg, 0.255 mmol). After celite/silica filtration and concentration *in vacuo*, slow evaporation of the crude complex (275 mg) in pentane / EtOH gave **2n** as an orange solid (**192 mg, 60% yield, 89% ee**). No enantioenrichment was observed in this step. Further recrystallization by cooling a saturated solution of **2n** (89% ee) in pentane to −20 °C yielded enantioenriched **2n** as orange needles (**143 mg, 45% yield, >99% ee**). (see page **93** for characterization data)

4. Synthesis of Characterization of Rh(III) and Ir(III) Complexes

General procedure I for the synthesis of $[Cp^XRhX_2]_2$ and $[Cp^XIrX_2]_2$ complexes according to procedures by Schley, Perekalin and Zhang[18-20]

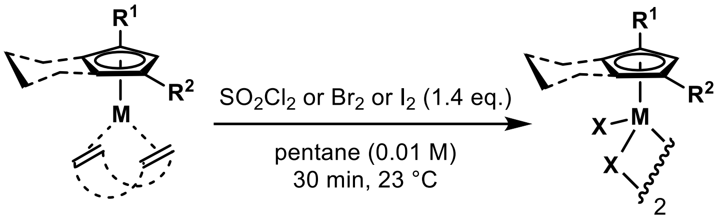

2 or **5** **3** or **4**

Without precaution to air or moisture, a solution of M(I) complex **2** or **5** in pentane (0.01 M) was treated with SO_2Cl_2 or Br_2, or Ir_2 (1.4 eq.) and stirred for 30 min at 23 °C. In the case of using SO_2Cl_2, the reaction mixture was concentrated under a stream of N_2. The precipitate was collected by filtration, washing with Et_2O or hexane and dried to afford the M(III) dimer complex **3** or **4**.

Note: Yields are calculated with respect to a monomeric unit of the product.

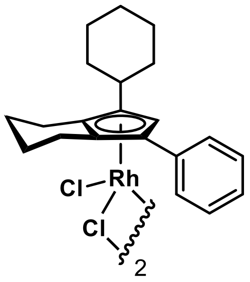

3a

Rh(III) complex **3a** was prepared via general procedure **I** with **2a** (20 mg, 40 µmol, 95% ee) and SO_2Cl_2 (4.6 µL, 57 µmol) to give an orange solid (17 mg, 90% yield, 95% ee based on **2a**).

^{1}H NMR (400 MHz, CD_2Cl_2) δ 7.75 (dt, *J* = 7.1, 1.4 Hz, 2H), 7.56 – 7.45 (m, 1H), 7.40 (dd, *J* = 8.2, 6.7 Hz, 2H), 5.50 (d, *J* = 1.5 Hz, 1H), 2.75 – 2.54 (m, 2H), 2.44 – 2.15 (m, 4H), 1.96 – 1.89 (m, 1H), 1.88 – 1.69 (m, 5H), 1.54 – 1.47 (m, 2H), 1.47 – 1.06 (m, 5H).

^{13}C NMR (101 MHz, CD_2Cl_2) δ 130.0, 129.9, 129.7, 129.2, 103.2, 101.3, 99.0, 87.9 (d, *J* = 9.7 Hz), 80.4 (d, J = 7.3 Hz), 34.2, 32.6, 30.7, 26.8, 26.4, 26.2, 22.7, 21.2, 21.1, 20.8.

IR (ATR) 2928, 2851, 1447, 1427, 1350, 1264, 1028, 980, 890, 733, 697.

HRMS (ESI/QTOF) m/z: [M - Cl]$^+$ calculated for $[C_{21}H_{25}ClRh]^+$: 415.0694; found: 415.0689.

m.p. 276.8 °C (decomposition).

Opt. Rot. $[\alpha]_D^{20}$ −39.28 (c = 0.4 in $CHCl_3$, 95% ee).

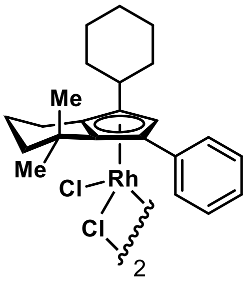

3b

Rh(III) complex **3b** was prepared via general procedure **I** with **2c** (3.8 mg, 7.4 µmol) and SO_2Cl_2 solution (41 µL, 0.25 M in dry PhMe, 10 µmol) to give an red solid (3.4 mg, 96% yield, 81% ee determined after conversion to compound **10b**). Red crystals suitable for X-ray analysis were obtained by slow evaporation of **3b** (81% ee) in DCM.

^{1}H NMR (400 MHz, CD_2Cl_2) δ 7.84 (d, *J* = 6.4 Hz, 2H), 7.46 – 7.35 (m, 3H), 5.20 (s, 1H), 3.00 (ddd, *J* = 17.8, 10.7, 7.5 Hz, 1H), 2.59 (td, *J* = 13.2, 3.4 Hz, 1H), 2.33 (d, *J* = 12.8 Hz, 1H), 2.20 (t, *J* = 11.7 Hz, 1H), 2.05 – 1.94 (m, 3H), 1.87 – 1.68 (m, 4H), 1.44 – 1.21 (m, 9H), 1.04 (s, 3H).

^{13}C NMR (101 MHz, CD_2Cl_2) δ 131.2, 131.1, 129.3, 128.3, 114.2 (d, *J* = 7.6 Hz), 102.7 (d, *J* = 10.2 Hz), 97.0 (d, *J* = 7.3 Hz), 91.4, 82.8 (d, *J* = 9.0 Hz), 37.6, 34.4, 33.9, 32.5, 30.6, 28.8, 27.1, 26.8, 26.3, 26.1, 19.8, 17.8.

IR (ATR) 2923, 2852, 1461, 1446, 1030, 759, 701, 665, 586.

HRMS (nanochip-ESI/LTQ-Orbitrap) m/z: [M − Cl]$^+$ calculated for $[C_{23}H_{29}ClRh]^+$: 443.1007; found: 443.099.

m.p. 221.5 °C (decomposition).

Opt. Rot. $[\alpha]_D^{20}$ +93.82 (c = 0.38 in $CHCl_3$, 81% ee).

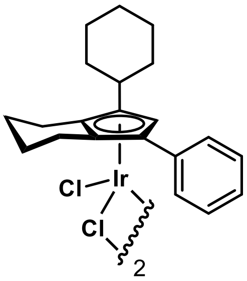

4a

Ir(III) complex **4a** was prepared via general procedure **I** with **5b** (20 mg, 44 µmol, 87% ee) and SO_2Cl_2 (5.0 µL, 60 µmol) to give an orange solid (19 mg, 92% yield, 87% ee determined after conversion to compound **11a**). Orange crystals suitable for X-ray analysis were obtained by slow evaporation of *rac*-**4a** in DCM.

^{1}H NMR (400 MHz, CD_2Cl_2) δ 7.71 – 7.61 (m, 2H), 7.49 – 7.42 (m, 1H), 7.41 – 7.34 (m, 2H), 5.85 (s, 1H), 2.61 – 2.51 (m, 1H), 2.50 – 2.39 (m, 1H), 2.30 – 2.20 (m, 2H), 2.19 – 1.92 (m, 5H), 1.85 – 1.69 (m, 3H), 1.67 – 1.52 (m, 2H), 1.46 – 1.31 (m, 2H), 1.30 – 1.11 (m, 3H).

^{13}C NMR (101 MHz, CD_2Cl_2) δ 131.2, 129.6, 129.3, 95.1, 94.8, 91.1, 82.5, 73.7, 34.5, 32.5, 30.8, 27.0, 26.4, 26.3, 22.6, 21.6, 21.2.

IR (ATR) 2927, 2850, 1448, 1429, 1266, 1029, 890, 765, 733, 696, 642, 567.

HRMS (ESI + APCI) m/z: [M - Cl]$^+$ calculated for $[C_{21}H_{25}ClIr]^+$: 505.1268; found: 505.1271.

m.p. 245.4 °C (decomposition).

Opt. Rot. $[\alpha]_D^{20}$ −19.93 (c = 1.0 in $CHCl_3$, 87% ee).

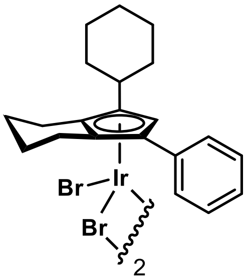

4b

Ir(III) complex **4b** was prepared via general procedure **I** with **5b** (10 mg, 19 µmol, 87% ee) and Br_2 (1.4 µL, 27 µmol) to give a red solid (10 mg, 86% yield, 87% ee based on **11a**).

^{1}H NMR (400 MHz, CD_2Cl_2) δ 7.63 – 7.58 (m, 2H), 7.45 – 7.39 (m, 1H), 7.39 – 7.32 (m, 2H), 5.79 (s, 1H), 2.80 – 2.65 (m, 1H), 2.55 – 2.43 (m, 1H), 2.41 – 2.28 (m, 1H), 2.28 – 2.01 (m, 5H), 2.00

– 1.92 (m, 1H), 1.85 – 1.68 (m, 3H), 1.67 – 1.56 (m, 2H), 1.46 – 1.31 (m, 2H), 1.29 – 1.12 (m, 3H).

^{13}C NMR (101 MHz, CD_2Cl_2) δ 131.3, 129.6, 129.2, 129.0, 97.4, 94.6, 92.3, 80.8 (seen in HMBC), 75.1, 34.4, 32.8, 30.8, 27.0, 26.4, 26.4, 22.8, 21.5, 21.1.

IR (ATR) 2926, 2850, 1447, 1428, 1414, 1265, 1029, 890, 764, 734, 697, 566.

HRMS (ESI + APCI) m/z: [M - Br]$^+$ calculated for $[C_{21}H_{25}BrIr]^+$: 549.0763; found: 549.0784.

m.p. 291.4 °C (decomposition).

Opt. Rot. $[\alpha]_D^{23}$ −21.67 (c = 1.00 in $CHCl_3$, 87% ee).

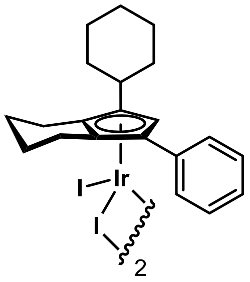

4c

Ir(III) complex **4c** was prepared via general procedure **I** with **5b** (0.10 g, 19 mmol, 87% ee) and I_2 (68 mg, 0.27 mmol) to give a dark red solid (0.13 g, 95% yield, 87% ee based on **11a**).

^{1}H NMR (400 MHz, CD_2Cl_2) δ 7.51 – 7.44 (m, 2H), 7.35 – 7.28 (m, 3H), 5.70 (s, 1H), 3.12 – 2.99 (m, 1H), 2.76 (dt, *J* = 16.9, 5.8 Hz, 1H), 2.50 – 2.27 (m, 4H), 2.23 – 2.12 (m, 1H), 2.04 (d, *J* = 12.5 Hz, 1H), 1.94 (d, *J* = 12.3 Hz, 1H), 1.83 – 1.58 (m, 5H), 1.40 – 1.27 (m, 2H), 1.25 – 1.10 (m, 3H).

^{13}C NMR (101 MHz, CD_2Cl_2) δ 130.3, 129.5, 129.0, 128.9, 99.4, 95.7, 94.5, 83.4, 75.7, 34.3, 33.6, 31.1, 26.8, 26.2, 26.2, 23.3, 21.9, 21.4, 21.0.

IR (ATR) 2924, 2850, 1446, 1427, 1260, 1091, 1026, 905, 802, 762, 728, 693, 644, 564.

HRMS (ESI + APCI) m/z: [M - I]$^+$ calculated for $[C_{21}H_{25}IIr]^+$: 597.0625; found: 597.0637.

m.p. 337.6 °C (decomposition).

Opt. Rot. $[\alpha]_D^{23}$ −21.82 (c = 1.00 in $CHCl_3$, 87% ee).

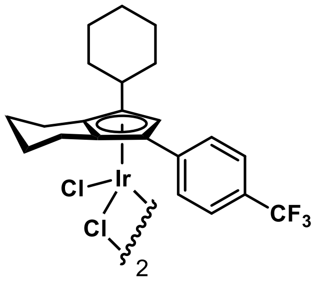

4d

Ir(III) complex **4d** was prepared via general procedure **I** with **5c** (55 mg, 93 µmol) and SO_2Cl_2 (11 µL, 0.13 mmol) to give an orange solid (53 mg, 95% yield, 88% ee as determined after conversion to compound **11b**).

^{1}H NMR (400 MHz, CD_2Cl_2) δ 7.79 (d, *J* = 8.1 Hz, 2H), 7.62 (d, *J* = 8.4 Hz, 2H), 5.84 (s, 1H), 2.71 – 2.55 (m, 1H), 2.43 – 2.31 (m, 1H), 2.28 – 1.92 (m, 7H), 1.85 – 1.69 (m, 3H), 1.68 – 1.54 (m, 2H), 1.46 – 1.30 (m, 2H), 1.29 – 1.13 (m, 3H).

^{13}C NMR (101 MHz, CD_2Cl_2) δ 135.7, 130.5 (q, *J* = 32.5 Hz), 130.1, 126.0 (q, *J* = 3.5 Hz), 96.2, 94.7, 91.2, 78.9, 74.6, 34.3, 32.6, 30.7, 27.0, 26.4, 26.3, 22.4, 21.4, 21.2, 21.1.

^{19}F NMR (376 MHz, CD_2Cl_2) δ −63.12.

IR (ATR) 2930, 2853, 1619, 1450, 1323, 1166, 1125, 1110, 1066, 1017, 845, 756.

HRMS (ESI + APCI) m/z: $[M - Cl]^+$ calculated for $[C_{22}H_{24}ClF_3Ir]^+$: 573.1142; found: 573.1150.

m.p. 281.4 °C (decomposition).

Opt. Rot. $[\alpha]_D^{20}$ −2.7 (c = 0.81 in $CHCl_3$, 88% ee).

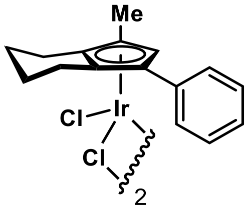

4e

Ir(III) complex **4e** was prepared via general procedure **I** with **5d** (70 mg, 0.15 mmol, 95% ee) and SO_2Cl_2 (18 µL, 0.20 mmol) to give an orange solid (67 mg, 93% yield, 95% ee based on **5d**).

^{1}H NMR (400 MHz, CD_2Cl_2) δ 7.62 – 7.54 (m, 2H), 7.48 – 7.39 (m, 1H), 7.39 – 7.30 (m, 2H), 5.90 (s, 1H), 2.61 – 2.51 (m, 1H), 2.17 – 2.03 (m, 2H), 2.02 – 1.88 (m, 2H), 1.87 – 1.78 (m, 4H), 1.74 – 1.62 (m, 1H), 1.52 – 1.42 (m, 1H).

^{13}C NMR (101 MHz, CD_2Cl_2) δ 131.1, 129.5, 129.2, 129.1, 95.0, 90.1, 87.6, 81.7, 76.1, 21.8, 21.6, 21.5, 21.0, 11.2.

IR (ATR) 2939, 2869, 1451, 1428, 1409, 1375, 1334, 1240, 1071, 1028, 905, 762, 696, 664.

HRMS (ESI + APCI) m/z: $[M - Cl]^+$ calculated for $[C_{16}H_{17}ClIr]^+$: 437.0642; found: 437.0626.

m.p. 244.5 °C (decomposition).

Opt. Rot. $[\alpha]_D^{20}$ +8.86 (c = 0.60 in $CHCl_3$, 95% ee).

General procedure J for the synthesis of [Cp^{X}MCl$_2$(P(OMe)$_3$)] complexes according to a procedure by Bergman[21]

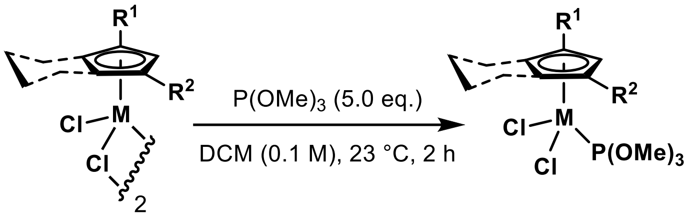

3 or **4** **10** or **11**

Without precaution to air moisture, a solution of $[Cp^XMCl_2]_2$ **3** or **4** in DCM (0.1 M) was treated with $P(OMe)_3$ (5.0 eq.). After stirring at 23 °C for 2 h, the reaction mixture was concentrated *in vacuo* and the crude residue was filtered through a short column of silica, washing with 1:1 pentane / EtOAc, and the filtrate concentrated *in vacuo* to afford the $[Cp^XMCl_2(P(OMe)_3)]$ complex **10** or **11**.

Note: Yields are calculated with respect to a monomeric unit of the starting material.

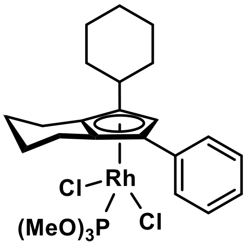

10a

Rh(III) complex **10a** was prepared via general procedure **J** with **3a** (10 mg, 11 µmol, 95% ee) and $P(OMe)_3$ (6.9 µmol, 55 µmol) to give an orange solid (13 mg, 98% yield, 95% ee based on **3a**). Orange crystals suitable for X-ray analysis were obtained by slow evaporation of *rac*-**10a** in benzene.

^{1}H NMR (400 MHz, CD_2Cl_2) δ 7.67 – 7.56 (m, 2H), 7.45 – 7.26 (m, 3H), 5.47 (d, J = 2.3 Hz, 1H), 3.47 (d, *J* = 11.2 Hz, 9H), 2.96 – 2.79 (m, 1H), 2.67 – 2.54 (m, 1H), 2.19 – 1.91 (m, 6H), 1.89 – 1.62 (m, 5H), 1.45 – 1.28 (m, 3H), 1.24 – 1.04 (m, 3H).

^{13}C NMR (101 MHz, CD_2Cl_2) δ 132.1, 129.1, 128.9, 128.8, 118.9 (dd, *J* = 10.5, 3.4 Hz), 112.0 (dd, *J* = 17.0, 3.3 Hz), 106.1 (dd, *J* = 7.5, 4.5 Hz), 87.1 (dd, *J* = 8.5, 2.5 Hz), 79.0 (dd, *J* = 7.5, 2.5 Hz), 54.2 (d, *J* = 6.4 Hz), 33.8, 33.7, 30.1, 29.5, 27.0, 26.5, 26.4, 22.3, 21.9 (d, *J* = 3.3 Hz), 21.7 (d, *J* = 4.8 Hz), 21.3.

^{31}P (NMR) (162 MHz, CD_2Cl_2) δ 117.37 (d, *J* = 217.0 Hz).

IR (ATR) 2926, 2850, 1450, 1183, 1058, 1028, 799, 755.

HRMS (ESI + APCI) m/z: $[M + Na]^+$ calculated for $[C_{24}H_{34}Cl_2RhNaO_3P]^+$: 597.0570; found: 597.0575.

m.p. 172.2 °C (racemate, decomposition).

R*f* 0.68 (silica gel, 1:1 pentane / EtOAc, UV).

Opt. Rot. $[\alpha]_D^{25}$ −44.05 (c = 0.14 in $CHCl_3$, 95% ee).

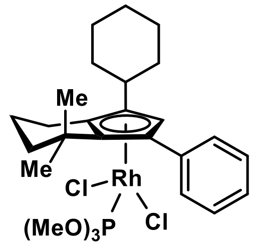

10b

Rh(III) complex **10b** was prepared via general procedure **J** with **3b** (3.3 mg, 6.9 µmol) and $P(OMe)_3$ (4.1 µmol, 35 µmol) to give a red solid (3.8 mg, 91% yield, 81% ee). Red crystals suitable for X-ray analysis were obtained by slow evaporation of **10b** (81% ee) in EtOAc.

^{1}H NMR (400 MHz, C_6D_6) δ 7.95 – 7.85 (m, 2H), 7.09 – 7.02 (m, 3H), 4.95 (d, *J* = 2.6 Hz, 1H), 3.56 (d, *J* = 10.9 Hz, 9H), 3.07 – 2.95 (m, 1H), 2.22 – 2.11 (m, 2H), 2.03 – 1.95 (m, 1H), 1.91 (d, *J* = 11.5 Hz, 1H), 1.82 – 1.71 (m, 6H), 1.69 – 1.59 (m, 2H), 1.58 – 1.47 (m, 1H), 1.38 – 1.29 (m, 2H), 1.22 – 1.04 (m, 4H), 0.82 (s, 3H).

^{13}C NMR (101 MHz, C_6D_6) δ 134.2, 131.9, 128.6, 127.6, 126.8 (dd, *J* = 16.5, 3.1 Hz), 113.3 (dd, *J* = 12.7, 3.5 Hz), 100.6 (dd, *J* = 8.1, 3.3 Hz), 92.8 (dd, *J* = 8.3, 3.4 Hz), 83.5 (dd, *J* = 7.7, 2.8 Hz),

54.9 (d, J = 7.5 Hz), 53.6 – 52.2 (m), 39.5, 35.1 (d, J = 3.8 Hz), 34.9, 33.4, 30.7, 30.4 (d, J = 9.6 Hz), 27.6 (d, J = 2.1 Hz), 27.1, 26.6 (d, J = 13.3 Hz), 21.3 (d, J = 4.2 Hz), 18.3.

^{31}P (NMR) (162 MHz, C_6D_6) δ 113.43 (d, J = 217.8 Hz).

IR (ATR) 2925, 2850, 1448, 1260, 1181, 1022, 797, 748, 702, 663, 532, 401.

HRMS (ESI/QTOF) m/z: $[M - Cl]^+$ calculated for $[C_{26}H_{38}ClO_3PRh]^+$: 567.1302; found: 567.1315.

m.p. 155.4 °C (decomposition).

Rf 0.68 (silica gel, 1:1 pentane / EtOAc, UV).

HPLC CHIRALPAK® IA, 90:10 hexane / IPA, rate 1 mL / min, 25 min, 254 nm, t_R major 11.32 min; t_R minor 12.88 min.

Opt. Rot. $[\alpha]_D^{22}$ −99.27 (c = 0.32 in $CHCl_3$, 81% ee).

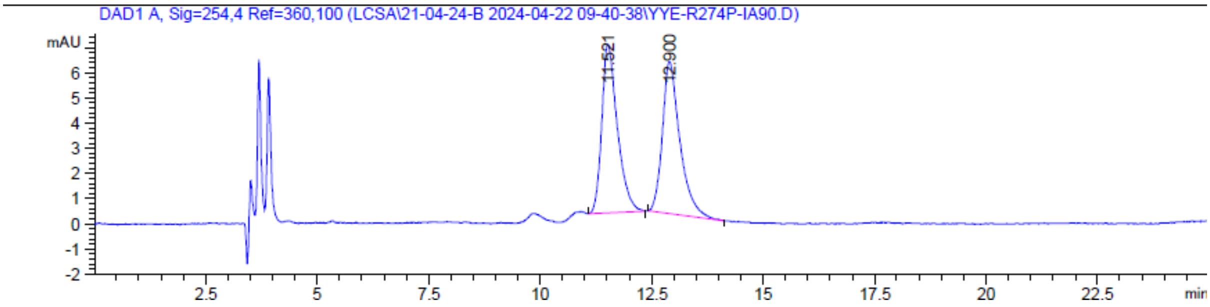

Signal 1: DAD1 A, Sig=254,4 Ref=360,100

Peak #	RetTime [min]	Type	Width [min]	Area [mAU*s]	Height [mAU]	Area %
1	11.521	BB	0.3531	162.50858	6.69758	49.5847
2	12.900	BB	0.3928	165.23056	6.04638	50.4153

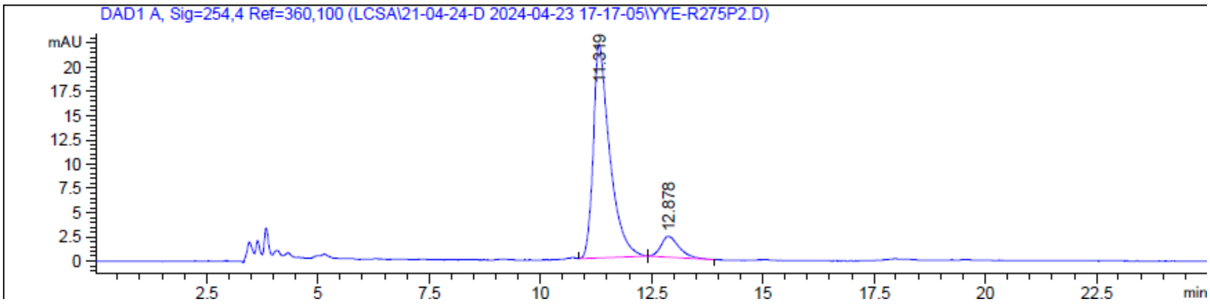

Signal 1: DAD1 A, Sig=254,4 Ref=360,100

Peak #	RetTime [min]	Type	Width [min]	Area [mAU*s]	Height [mAU]	Area %
1	11.319	BB	0.3657	564.96844	21.99079	90.3732
2	12.878	BB	0.3844	60.18209	2.15093	9.6268

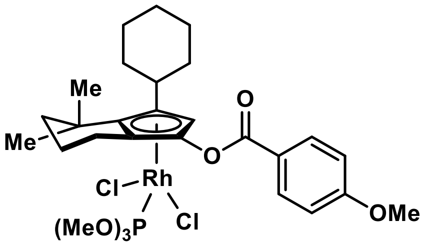

10c

Rh(III) complex **10c** was prepared according to a variation of general procedures **I** and **J.** A suspension of **2m** (7.1 mg, 12 µmol, 86% ee) and $NaHCO_3$ (5.0 mg, 60 µmol, 5.0 eq.) in dry Et_2O (1.2 mL, 0.01 M) under N_2 was treated with SO_2Cl_2 solution (67 µL, 0.25 M in dry Et_2O, 17 µmol, 1.4 eq.) and stirred for 5 min at 23 °C. The reaction mixture was concentrated under a stream of N_2, then the residue was directly treated with $P(OMe)_3$ solution (1.2 mL, 0.05 M in Et_2O, 60 µmol, 5.0 eq.) and stirred for a further 10 min at 23 °C. The reaction mixture was concentrated under a stream of N_2 and filtered through a short column of silica (2:1 to 1:1 pentane / EtOAc gradient) collecting only the red band, then concentrated to afford **10c** as a red oil (5.6 mg, 69% yield, 85% ee). Red crystals (racemic) suitable for X-ray analysis were obtained by cooling a saturated solution of **10c** (85% ee) in pentane to −20 °C. The resulting mother liquor was separated and concentrated to give enantioenriched **10c** as a red oil (99% ee).

^{1}H NMR (400 MHz, C_6D_6) δ 8.06 (d, *J* = 8.8 Hz, 2H), 6.59 (d, *J* = 8.7 Hz, 2H), 5.99 (s, 1H), 3.69 (d, *J* = 11.0 Hz, 9H), 3.16 (s, 3H), 3.04 (dtd, *J* = 17.1, 7.7, 3.2 Hz, 1H), 2.87 (d, *J* = 12.4 Hz, 1H), 2.71 (t, *J* = 11.5 Hz, 1H), 2.34 – 2.25 (m, 1H), 2.11 (d, *J* = 13.1 Hz, 1H), 2.01 – 1.88 (m, 4H), 1.75 (d, *J* = 11.4 Hz, 2H), 1.61 (t, *J* = 13.7 Hz, 2H), 1.51 – 1.33 (m, 2H), 1.29 – 1.18 (m, 2H), 1.15 – 0.94 (m, 6H).

^{13}C NMR (101 MHz, C_6D_6) δ 164.6, 163.1, 132.7, 121.4, 114.4, 112.8 – 112.4 (m), 111.5 (dd, *J* = 7.3, 5.3 Hz), 109.9 (d, *J* = 8.3 Hz), 107.2 (d, *J* = 3.7 Hz), 106.9 (d, *J* = 3.7 Hz), 69.9 (dd, *J* = 7.3, 2.6 Hz), 55.0 (d, *J* = 6.5 Hz), 40.1, 36.4, 36.2, 33.8 (d, *J* = 5.4 Hz), 30.9, 30.4, 30.2, 29.5 (d, *J* = 3.3 Hz), 27.0, 26.7, 26.3, 19.5, 17.6.

^{31}P (NMR) (162 MHz, C_6D_6) δ 112.25 (d, J = 210.9 Hz).

IR (ATR) 2925, 2851, 1741, 1605, 1512, 1454, 1252, 1168, 1097, 1058, 1023, 797. 759.

HRMS (ESI/QTOF) m/z: $[M-Cl]^+$ calculated for $[C_{28}H_{40}ClO_6PRh]^+$: 641.1301; found: 641.1322.

m.p. 156.2 – 157.2 °C (racemate).

Rf 0.25 (silica gel, 2:1 pentane / EtOAc, UV).

HPLC CHIRALPAK® IA, 80:20 hexane / IPA, rate 1 mL / min, 25 min, 254 nm, t_R minor 8.17 min; t_R major 12.30 min.

Opt. Rot. $[\alpha]_D^{20}$ −65.20 (c = 0.41 in $CHCl_3$, 99% ee).

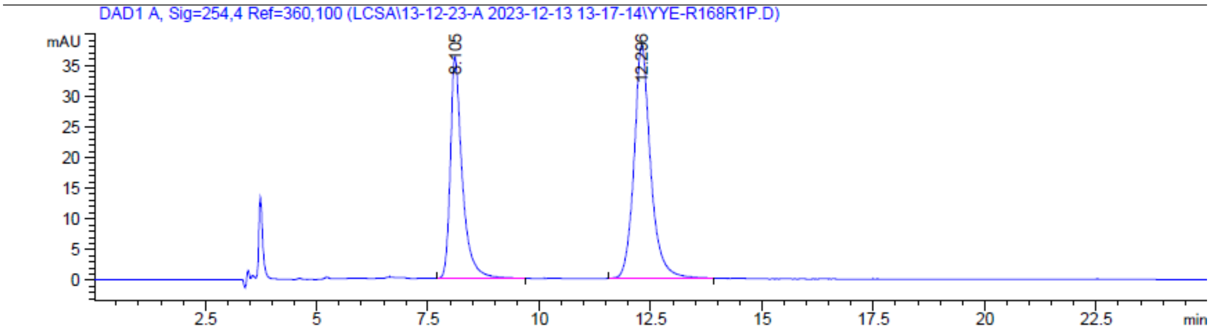

Signal 1: DAD1 A, Sig=254,4 Ref=360,100

Peak #	RetTime [min]	Type	Width [min]	Area [mAU*s]	Height [mAU]	Area %
1	8.105	BB	0.2708	670.01990	36.04794	40.5702
2	12.296	BB	0.3818	981.48737	37.93857	59.4298

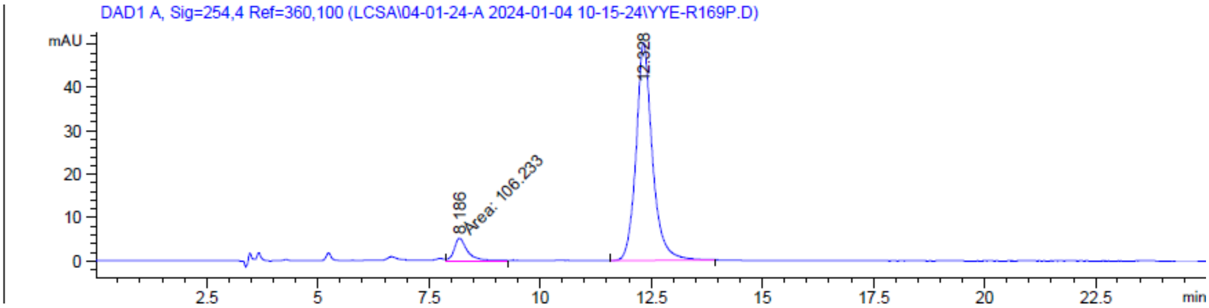

Signal 1: DAD1 A, Sig=254,4 Ref=360,100

Peak #	RetTime [min]	Type	Width [min]	Area [mAU*s]	Height [mAU]	Area %
1	8.186	MM T	0.3400	106.23318	5.20689	7.6599
2	12.328	BB	0.3743	1280.64148	50.09261	92.3401

After recrystallization and separation of racemate:

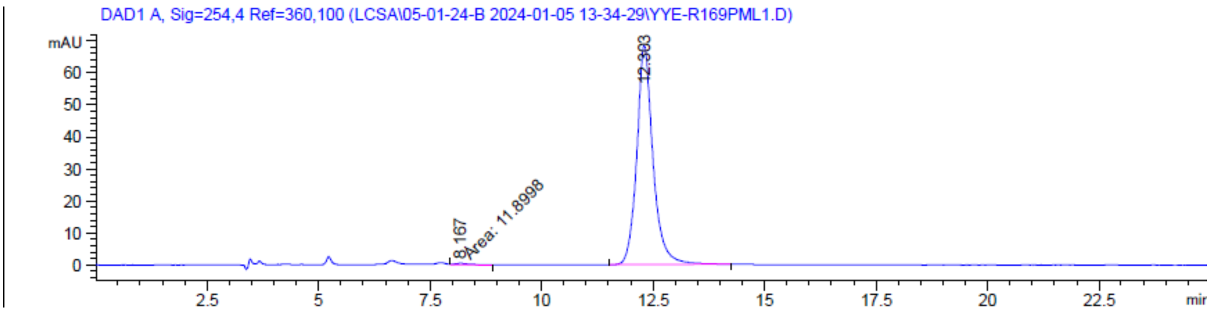

Signal 1: DAD1 A, Sig=254,4 Ref=360,100

Peak #	RetTime [min]	Type	Width [min]	Area [mAU*s]	Height [mAU]	Area %
1	8.167	MM T	0.3673	11.89981	4.57920e-1	0.6814
2	12.303	BB	0.3698	1734.54004	68.41701	99.3186

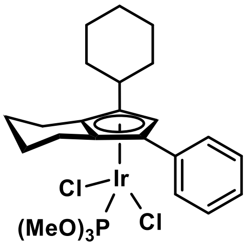

11a

Ir(III) complex **11a** was prepared via general procedure **J** with **4a** (20 mg, 19 µmol) and $P(OMe)_3$ (11 µL, 91 µmol) to give an orange solid (23 mg, 95% yield, 87% ee). Orange crystals suitable for X-ray analysis were obtained by slow evaporation of *rac*-**11a** in benzene.

^{1}H NMR (400 MHz, C_6D_6) δ 7.52 – 7.44 (m, 2H), 7.08 – 6.97 (m, 3H), 5.36 (s, 1H), 3.38 (d, *J* = 11.5 Hz, 9H), 3.04 – 2.88 (m, 1H), 2.78 – 2.66 (m, 1H), 2.35 – 2.20 (m, 2H), 1.92 – 1.82 (m, 1H), 1.81 – 1.55 (m, 7H), 1.52 – 1.41 (m, 1H), 1.40 – 1.34 (m, 1H), 1.34 – 1.22 (m, 1H), 1.22 – 1.12 (m, 1H), 1.11 – 0.98 (m, 3H).

^{13}C NMR (101 MHz, C_6D_6) δ 133.8, 128.8, 128.6, 115.9 (d, *J* = 10.5 Hz), 108.4 (d, *J* = 14.9 Hz), 95.6, 78.6, 73.3, 53.3 (d, *J* = 5.8 Hz), 34.1, 33.2, 30.1, 27.2, 26.7, 22.0, 21.1, 21.0, 21.9, 20.9.

^{31}P (NMR) (162 MHz, C_6D_6) δ 79.48.

IR (ATR) 2928, 2850, 1449, 1183, 1058, 1028, 813, 801, 761, 699.

HRMS (ESI + APCI) m/z: $[M + Na]^+$ calculated for $[C_{24}H_{34}Cl_2IrNaO_3P]^+$: 687.1144; found: 687.1146.

m.p. 194.5 – 196.5 °C.

Rf 0.98 (silica gel, 3:1 Et_2O / DCM, UV).

SFC CHIRALPAK® IJ, 10% MeOH in CO_2, rate 2 mL / min, 5 min, 240 nm, t_R minor 1.97 min; t_R major 2.31 min.

Opt. Rot. $[\alpha]_D^{20}$ −38.33 (c = 0.3 in $CHCl_3$, 87% ee).

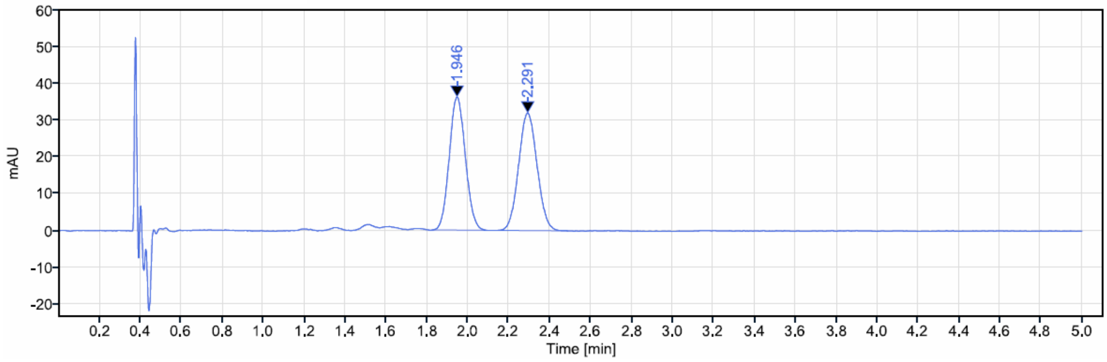

RT [min]	Type	Width [min]	Area	Height	Area%
1.946	MM m	0.29	198.83	36.29	49.62
2.291	MM m	0.35	201.89	32.12	50.38
		**Sum**	**400.73**		

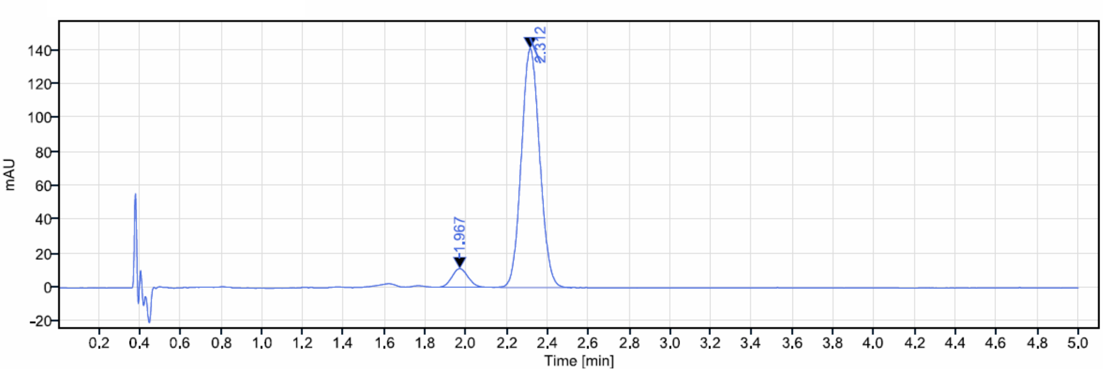

RT [min]	Type	Width [min]	Area	Height	Area%
1.967	MM m	0.26	60.91	11.02	6.46
2.312	MM m	0.38	881.86	140.60	93.54
		**Sum**	**942.77**		

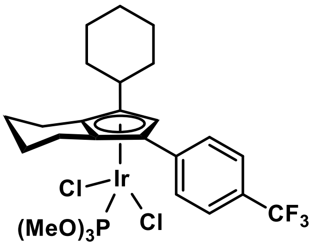

11b

Ir(III) complex **11b** was prepared via general procedure **J** with **4d** (19 mg, 15 µmol) and $P(OMe)_3$ (9.5 µL, 76 µmol) to give an orange solid (21 mg, 95% yield, 88% ee).

^{1}H NMR (400 MHz, CD_2Cl_2) δ 7.67 (d, J = 8.2 Hz, 2H), 7.62 – 7.56 (m, 2H), 5.52 (s, 1H), 3.47 (d, J = 11.5 Hz, 9H), 2.76 – 2.61 (m, 1H), 2.55 – 2.43 (m, 1H), 2.13 – 1.93 (m, 5H), 1.91 – 1.84 (m,

1H), 1.83 – 1.74 (m, 3H), 1.74 – 1.66 (m, 1H), 1.54 – 1.48 (m, 1H), 1.44 – 1.30 (m, 2H), 1.30 – 1.01 (m, 4H).

^{13}C NMR (101 MHz, CD_2Cl_2) δ 137.7, 129.7 (q, *J* = 32.6 Hz), 129.2, 125.7 (q, *J* = 3.5 Hz), 123.2, 116.3 (d, *J* = 10.2 Hz), 109.0 (d, *J* = 14.6 Hz), 96.9 (d, *J* = 2.0 Hz), 77.0, 74.4, 53.7 (d, *J* = 6.4 Hz), 34.0, 33.3, 30.0, 27.2, 26.6, 26.6, 22.0, 21.1 (d, *J* = 3.5 Hz), 21.1, 20.9 (d, *J* = 2.7 Hz).

^{19}F (NMR) (376 MHz, CD_2Cl_2) δ −63.04.

^{31}P (NMR) (162 MHz, CD_2Cl_2) δ 78.24.

IR (ATR) 2927, 2851, 1617, 1449, 1418, 1323, 1268, 1166, 1123, 1110, 1063, 1026, 847.

HRMS (ESI/QTOF) m/z: [M + Na]$^+$ calculated for $[C_{25}H_{33}Cl_2F_3IrNaO_3P]^+$: 755.1018; found: 755.1015.

m.p. 223.6 – 225.6 °C.

Rf 0.99 (silica gel, 3:1 Et_2O / DCM, UV).

SFC CHIRALPAK® IJ, 3% MeOH in CO_2, rate 2 mL / min, 10 min, 240 nm, t_R minor 5.30 min; t_R major 6.07 min.

Opt. Rot. $[\alpha]_D^{20}$ −30.46 (c = 0.29 in $CHCl_3$, 88% ee).

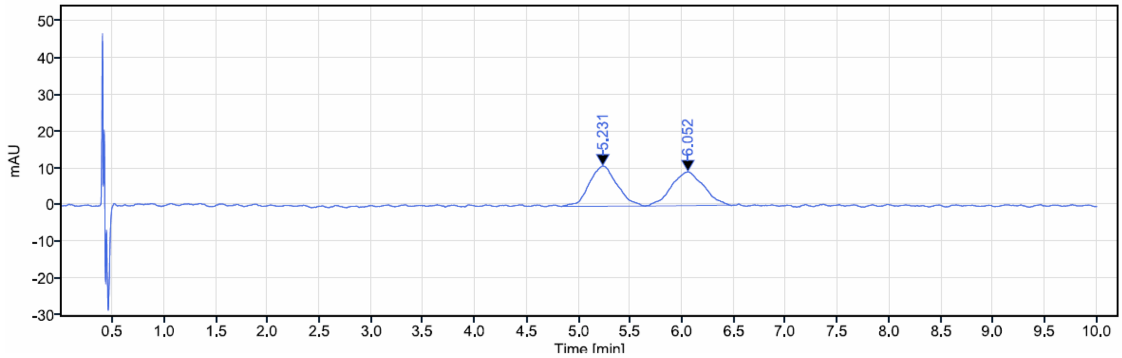

RT [min]	Type	Width [min]	Area	Height	Area%
5.231	MM m	0.82	202.63	10.92	50.48
6.052	MM m	0.86	198.81	9.21	49.52
		**Sum**	**401.44**		

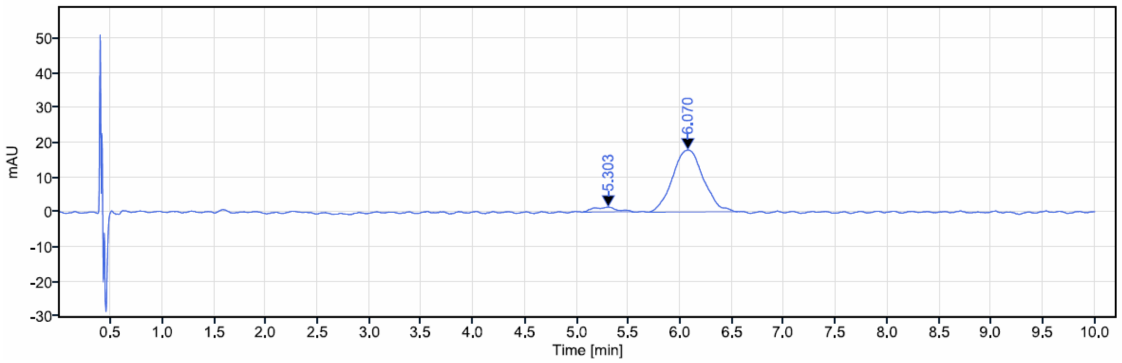

RT [min]	Type	Width [min]	Area	Height	Area%
5.303	MM m	0.49	22.64	1.44	5.76
6.070	MM m	0.85	370.31	17.87	94.24
		**Sum**	**392.95**		

Non-enantiospecific complexation of cyclopentadiene 1a with TlOEt and $[Rh(COD)Cl]_2$[22]

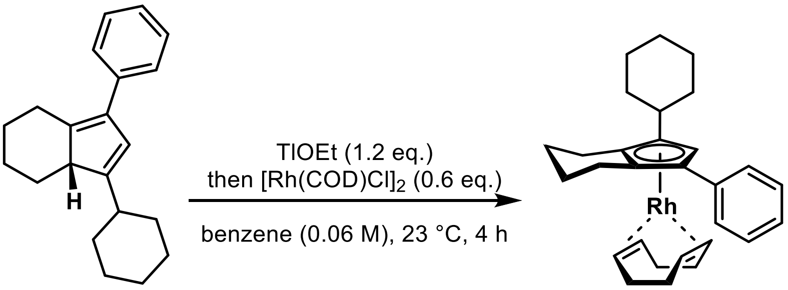

1a **2a**

In a N_2-filled glovebox, a solution of thallium ethoxide (16 mg, 65 µmol, 1.2 eq.) in dry degassed benzene (0.3 mL) was treated with a solution of **1a** (15 mg, 54 µmol, 1.0 eq.) in dry degassed benzene (0.2 mL). The mixture was stirred in the dark at 23 °C for 2 h, before being diluted with dry degassed benzene (0.4 mL) and treated with $[Rh(COD)Cl]_2$ (16 mg, 32 µmol, 0.60 eq.). After a further 4 h of stirring, the reaction mixture was filtered through neutral alumina on celite, and the filtrate concentrated *in vacuo* to afford Rh(I) complex **2a** as a yellow solid (20 mg, 77% yield, 0% ee). (see page **71** for full characterization data)

HPLC CHIRALPAK® IA, 99.5:0.5 hexane / IPA, rate 0.5 mL / min, 25 min, 230 nm, t_R 8.04 min; t_R 8.52 min.

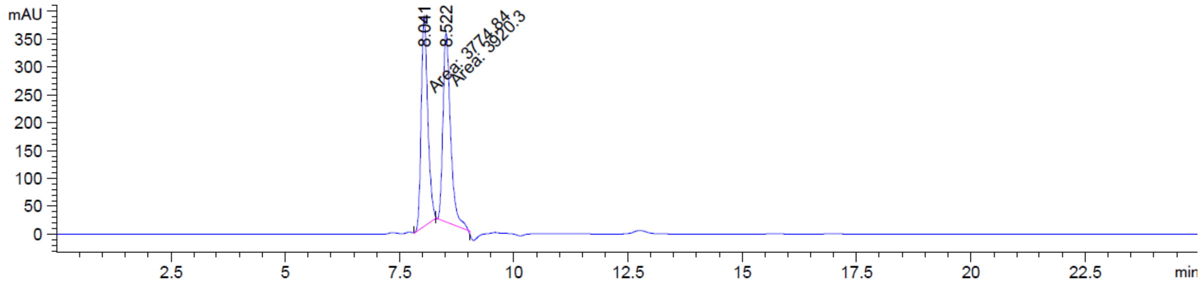

Peak #	RetTime [min]	Type	Width [min]	Area [mAU*s]	Height [mAU]	Area %
1	8.041	MM	0.1664	3774.84448	378.15509	49.0549
2	8.522	MM	0.1929	3920.30249	338.76675	50.9451

Non-enantiospecific complexation of cyclopentadiene 1a with nBuLi and $[Rh(COD)Cl]_2$[23]

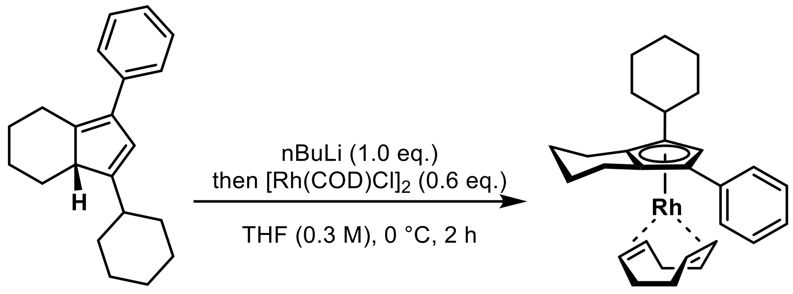

1a **2a**

In a N_2-filled glovebox, a solution of **1a** (40.0 mg, 0.144 mmol, 1.0 eq.) in dry THF (0.5 mL) was treated with nBuLi (57.5 µL, 2.50 M in hexanes, 0.144 mmol, 1.0 eq.) at 0 °C and stirred for 15 min. The mixture was then treated with $[Rh(COD)Cl]_2$ (42.5 mg, 86.2 µmol, 0.6 eq.) and stirred at 0 °C for 2 h. The reaction mixture was concentrated *in vacuo*, then the crude residue was suspended in pentane (2 mL) and filtered through celite on alumina, washing with 10 : 1 pentane/EtOAc. The filtrate was concentrated *in vacuo* to afford Rh(I) complex **2a** as a yellow solid (66.7 mg, 95% yield, 0% ee). (see page **71** for full characterization data)

HPLC CHIRALPAK® IA, 99.5:0.5 hexane / IPA, rate 0.5 mL / min, 25 min, 230 nm, t_R 7.66 min; t_R 8.17 min.

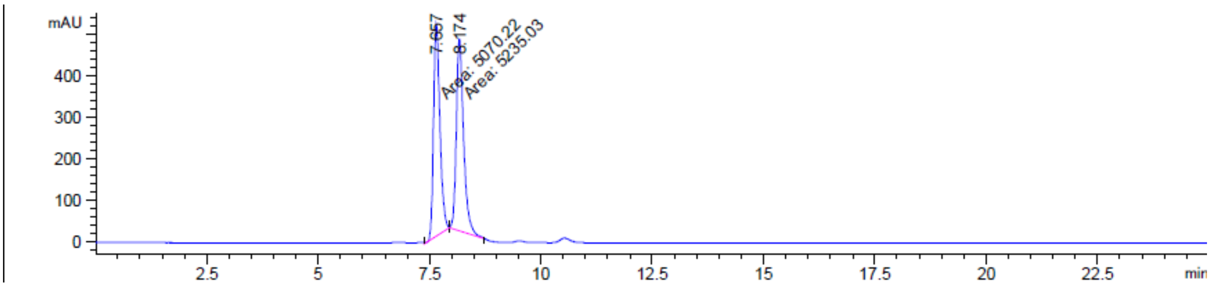

Peak #	RetTime [min]	Type	Width [min]	Area [mAU*s]	Height [mAU]	Area %
1	7.657	MM T	0.2100	5070.22021	510.62344	49.2004
2	8.174	MM T	0.1886	5235.02979	462.64459	50.7996
Totals :				1.03053e4	973.26804	

Non-enantiospecific complexation of cyclopentadiene 1a with $RhCl_3$[24]

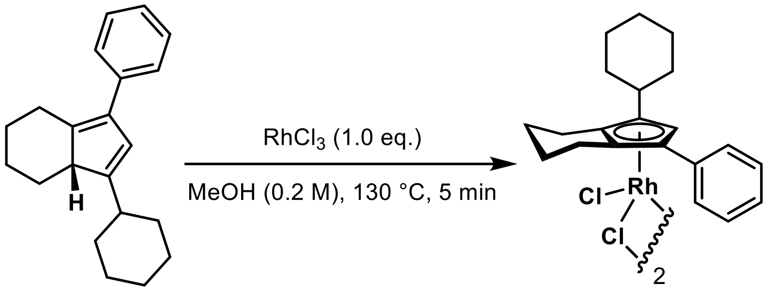

1a (1.1 eq.) **3a**

A suspension of **1a** (17.5 mg, 62.7 µmol, 1.1 eq., 98% ee), $RhCl_3$ (15 mg, 57 µmol, 1.0 eq.) in degassed MeOH (0.25 mL, 0.2 M relative to $RhCl_3$) under N_2 was stirred in a microwave reactor at 130 °C for 5 min. The reaction mixture was cooled to 0 °C and the precipitate collected by filtration, washing with ice-cold MeOH to afford Rh(III) complex **3a** as an orange solid (24 mg, 53 µmol, 93% yield, 1% ee as determined after conversion to compound **10a**). (see page **108** for full characterization data)

After conversion of **3a** to **10a**:

SFC CHIRALPAK® IJ, 10% MeOH in CO_2, rate 2 mL / min, 5 min, 240 nm, t_R major 1.79 min; t_R minor 2.18 min.

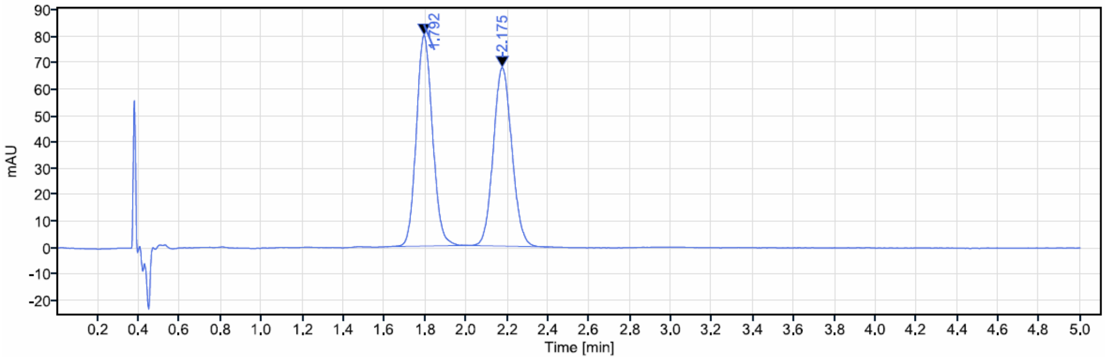

RT [min]	Type	Width [min]	Area	Height	Area%
1.792	MM m	0.38	438.47	79.65	50.72
2.175	MM m	0.41	425.96	67.50	49.28
		**Sum**	**864.42**		

Non-enantiospecific complexation of cyclopentadiene 1a with $[Ir(COD)Cl]_2$ and HCl[25]

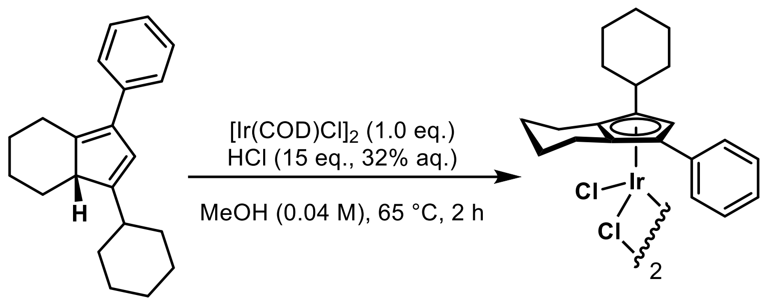

A suspension of **1a** (16 mg, 56 µmol, 2.5 eq., 90% ee), $[Ir(COD)Cl]_2$ (15.0 mg, 22.4 µmol, 1.0 eq.) and degassed HCl solution (32 µL, 32% aq., 0.33 mmol, 15 eq.) in degassed MeOH (0.6 mL, 0.04 M relative to $[Ir(COD)Cl]_2$) under N_2 was stirred in a closed vessel at 65 °C for 2 h. The reaction mixture was cooled to 0 °C and the precipitate collected by filtration, washing with ice-cold MeOH to afford Ir(III) complex **4a** as a red-orange solid (20 mg, 19 µmol, 84% yield, 5% ee as determined after conversion to compound **11a**). (see page **110** for full characterization data)

After conversion of **4a** to **11a**:

SFC CHIRALPAK® IJ, 10% MeOH in CO_2, rate 2 mL / min, 5 min, 240 nm, t_R minor 1.97 min; t_R major 2.32 min.

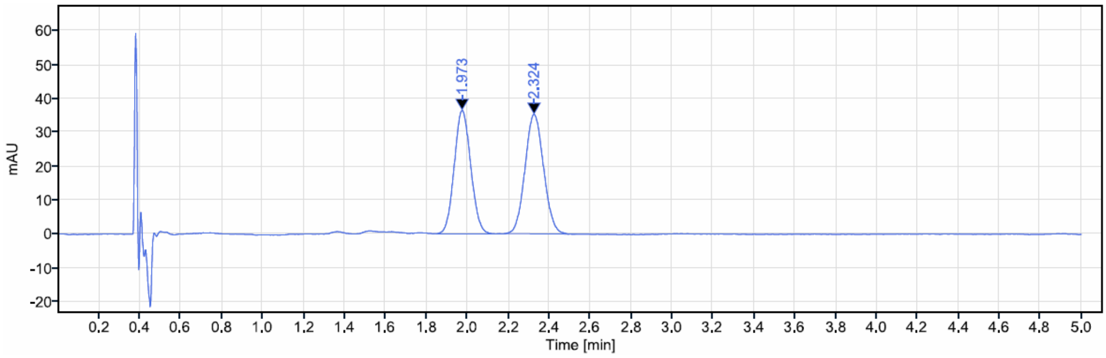

RT [min]	Type	Width [min]	Area	Height	Area%
1.973	MM m	0.31	202.64	36.34	47.67
2.324	MM m	0.37	222.47	35.24	52.33
		**Sum**	**425.12**		

5. Rh-Catalyzed Asymmetric C-H Functionalization Reactions

Note: *N*-((tert-butoxycarbonyl)oxy)benzamide was synthesized according to a procedure by Cramer.[22]

General procedure K for the C-H functionalization reaction of *N*-((tert-butoxycarbonyl)oxy)benzamide (12) with alkenes according to a procedure by Cramer[22]

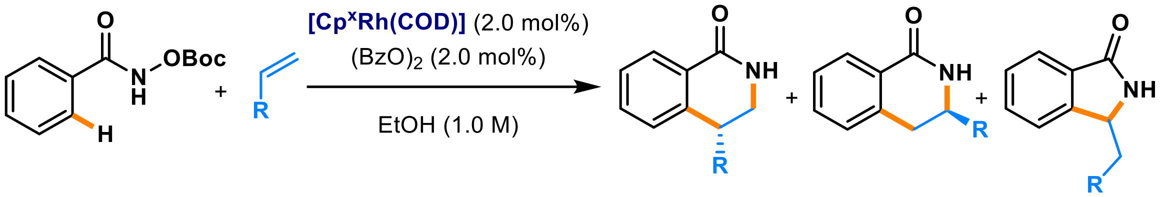

12 (1.0 eq.) (2.0 eq.) **13** **14** **15**

Without precaution to air or moisture, a suspension of *N*-((tert-butoxycarbonyl)oxy)benzamide (23.7 mg, 0.100 mmol, 1.0 eq.), alkene (0.20 mmol, 2.0 eq.) and [Cp^{X}Rh(COD)] (2.0 µmol, 2.0 mol%) in EtOH (0.10 mL, 1.0 M) was treated with benzoyl peroxide (0.50 mg, 2 µmol, 2.0 mol%) and then stirred at the specified temperature and duration. The reaction mixture was then concentrated *in vacuo* and the crude residue subjected to column chromatography (silica gel) to afford the 3,4-dihydroisoquinolone/isoindolin-1-one products.

Table S1. Benchmarks for C-H functionalization reaction of **12** with various alkenes.

Entry	R	[Rh]	[Rh] er	time / temp	conv	13+14 yield	13+14 rr	13 er (ee*)	14 er (ee*)	15 yield	15 er (ee*)
1	Ph	**2a**	97 : 3	16 h, 23 °C	100%	54%	0 : 100	-	87 :13 (79%)	41%	59 : 41 (19%)
2	Ph	**2b**	93 : 7	16 h, 23 °C	100%	47%	0 : 100	-	86 : 14 (84%)	34%	55 : 45 (12%)
3	Ph	**2c**	90 : 10	16 h, 23 °C	17%	4%	0 : 100	-	24 : 76 (65%)	<1%	n.d.
4	Ph	**2d**	97 : 3	16 h, 23 °C	65%	20%	0 : 100	-	88 : 12 (83%)	15%	40 : 60 (21%)
5	Ph	**2f**	95 : 5	19 h, 23 °C	100%	56%	0 : 100	-	60 : 40 (22%)	16%	59 : 41 (20%)

Entry	R	[Rh]	[Rh] er	time / temp	conv	13+14 yield	13+14 rr	13 er (ee*)	14 er (ee*)	15 yield	15 er (ee*)
6	Ph	**2g**	92 : 8	16 h, 23 °C	26%	2%	0 : 100	-	23 : 77 (64%)	3%	22 : 78 (67%)
7	Ph	**2h**	95.2 : 4.8	44 h, 23 °C	66%	35%	0 : 100	-	8.1 : 91.9 (93%)	3%	50 : 50 (0%)
8	Ph	**2i**	95 : 5	68 h, 23 °C	35%	10%	0 : 100	-	6.6 : 93.4 (96%)	2%	n.d.
9	Ph	**2j**	94.5 : 5.5	96 h, 23 °C	96%	76%	0 : 100	-	92.7 : 7.3 (96%)	3%	n.d.
10	Ph	**2k**	99.5 : 0.5	78 h, 23 °C	34%	11%	0 : 100	-	98.4 : 1.6 (98%)	0%	-
11	Ph	**2l**	94.8 : 8.2	67 h, 23 °C	83%	58%	0 : 100	-	92.5 : 7.5 (95%)	1%	n.d.
12	Ph	**2m**	93.0 : 7.0	17 h, 23 °C	100%	94%	0 : 100	-	92.5 : 7.5 (99%)	2%	n.d.
13	Ph	**2n**	99.5 : 0.5	41 h, 0 °C	91%	82%	0 : 100	-	98.8 : 1.2 (99%)	1%	n.d.
14	Ph	**2n**	99.8 : 0.2	41 h, −20 °C	100%	95%	0 : 100	-	99.8 : 0.2 (>99%)	4%	n.d.
15	Ph	**2o**	98.8 : 1.2	43 h, 23 °C	98%	85%	0 : 100	-	85 : 15 (71%)	2%	n.d.
16	Ph	**2p**	99.4 : 0.6	42 h, 23 °C	100%	69%	0 : 100	-	97.5 : 2.5 (96%)	1%	n.d.
17	Bu	**2a**	97 : 3	16 h, 23 °C	100%	91%	11 : 1	78 : 22 (59%)	n.d.	0%	-
18	Bu	**2b**	93 : 7	16 h, 23 °C	100%	86%	11 : 1	74 : 26 (56%)	59 : 41 (21%)	0%	-
19	Bu	**2c**	90 : 10	16 h, 23 °C	100%	77%	19 : 1	39 : 61 (27%)	44 : 56 (15%)	0%	-
20	Bu	**2d**	97 : 3	16 h, 23 °C	100%	82%	4.8 : 1	76 : 24 (55%)	74 : 26 (51%)	0%	-

Entry	R	[Rh]	[Rh] er	time / temp	conv	13+14 yield	13+14 rr	13 er (ee*)	14 er (ee*)	15 yield	15 er (ee*)
21	Bu	**2f**	95 : 5	16 h, 23 °C	100%	91%	1.9 : 1	51 : 49 (2%)	54 : 46 (9%)	0%	-
22	Bu	**2g**	92 : 8	16 h, 23 °C	66%	51%	> 20 : 1	34 : 66 (38%)	68 : 33 (42%)	0%	-
23	Bu	**2h**	95 : 5	16 h, 23 °C	100%	96%	6.5 : 1	28 : 72 (49%)	32 : 68 (40%)	0%	-
24	Bu	**2i**	95 : 5	67 h, 23 °C	45%	22%	4.0 : 1	29 : 71 (47%)	24 : 76 (58%)	0%	-
25	Bu	**2j**	94.5 : 5.5	72 h, 23 °C	100%	98%	3.2 : 1	83 : 17 (74%)	89 : 11 (88%)	0%	-
26	Bu	**2k**	99.5 : 0.5	76 h, 23 °C	44%	22%	6.5 : 1	88 : 12 (77%)	93 : 7 (87%)	0%	-
27	Bu	**2l**	94.8 : 5.2	67 h, 23 °C	94%	73%	5.8 : 1	84 : 16 (76%)	89 : 11 (87%)	0%	-
28	Bu	**2m**	93.0 : 7.0	17 h, 23 °C	100%	87%	5.3 : 1	84 : 16 (79%)	88 : 12 (88%)	0%	-
29	Bu	**2n**	98.9 : 1.1	17 h, 23 °C	100%	97%	5.3 : 1	88 : 12 (78%)	97 : 3 (96%)	0%	-
30	Bu	**2n**	99.6 : 0.4	2 h, 0 °C	100%	> 99%	5.3 : 1	91.6 : 8.4 (84%)	97 : 3 (95%)	0%	-
31	Bu	**2n**	99.7 : 0.3	41 h, −20 °C	100%	88%	7.5 : 1	93.3 : 6.7 (87%)	97 : 3 (95%)	0%	-
32	Bu	**2o**	98.8 : 1.2	43 h, 23 °C	100%	72%	4.9 : 1	69 : 31 (39%)	63 : 37 (27%)	0%	-
33	Bu	**2p**	99.4 : 0.6	16 h, 23 °C	100%	86%	5.3 : 1	87 : 13 (75%)	93 : 7 (87%)	0%	-
34	Bu	**10c**[a]	99.3 : 0.7	54 h, 23 °C	88%	14%	> 20 : 1	80 : 20 (61%)	94 : 6 (89%)	0%	-
35	Bn	**2n**	99.7 : 0.3	41 h, −20 °C	100%	97%	5.1 : 1	95.2 : 4.8 (91%)	94.3 : 5.8 (89%)	0%	-

Entry	R	[Rh]	[Rh] er	time / temp	conv	13+14 yield	13+14 rr	13 er (ee*)	14 er (ee*)	15 yield	15 er (ee*)
36	CH_2OH	**2n**	99.8 : 0.2	3 h, 23 °C	91%	78%	1.9 : 1	95.0 : 5.0 (90%)	97.0 : 3.0 (94%)	0%	-

ee* = ee of product / ee of catalyst.

[a] used 0.06 eq NaOBz instead of $(BzO)_2$.

Reaction entry 14:

General procedure **K** was carried out using styrene (23 µL, 0.20 mmol), Rh(I) complex **2n** (1.3 mg, 2.0 µmol, >99% ee), temperature −20 °C (stored in freezer with occasional agitation) and duration 41 h. A 24 : 1 ratio of **14a** and **15a** was determined by NMR analysis of the crude mixture. Chromatographic separation (2 : 1 to 1 : 2 pentane / EtOAc gradient) afforded **14a** (first fractions) as a colorless solid (21.2 mg, 95% yield, >99% ee) and **15a** (second fractions) as a colorless solid (0.9 mg, 4% yield, ee n.d.).

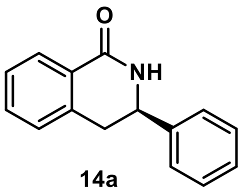

14a

The following characterization data were consistent with literature.[26]

^{1}H NMR (400 MHz, $CDCl_3$) δ 8.13 (d, *J* = 1.6 Hz, 1H), 7.47 (td, *J* = 7.4, 1.5 Hz, 1H), 7.43 – 7.32 (m, 6H), 7.19 (d, *J* = 7.7 Hz, 1H), 6.01 (s, 1H), 4.87 (dd, *J* = 11.2, 4.8 Hz, 1H), 3.27 – 3.07 (m, 2H).

HPLC CHIRALPAK® ID, 80:20 hexane / IPA, rate 1 mL / min, 15 min, 254 nm, t_R major 9.33 min; t_R minor 10.13 min.

Opt. Rot. $[\alpha]_D^{20}$ +198.17 (c = 1.00 in $CHCl_3$, >99% ee). Literature for opposite enantiomer: $[\alpha]_D^{20}$ −161 (c = 1.0 in $CHCl_3$, 99.2% ee).

Chromatogram

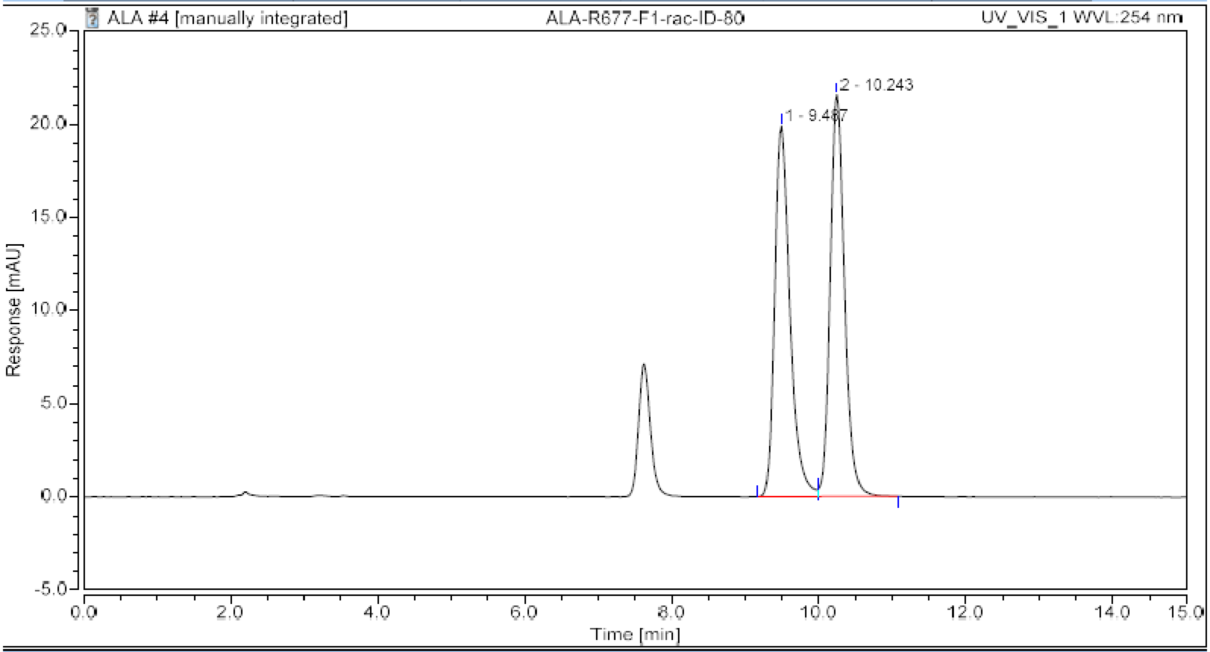

Integration Results

No.	Peak Name	Retention Time min	Area mAU*min	Height mAU	Relative Area %	Relative Height %	Amount n.a.
1		9.487	4.828	19.904	49.49	47.98	n.a.
2		10.243	4.928	21.579	50.51	52.02	n.a.
**Total:**			**9.755**	**41.483**	**100.00**	**100.00**	

For reaction entry **14**:

Chromatogram

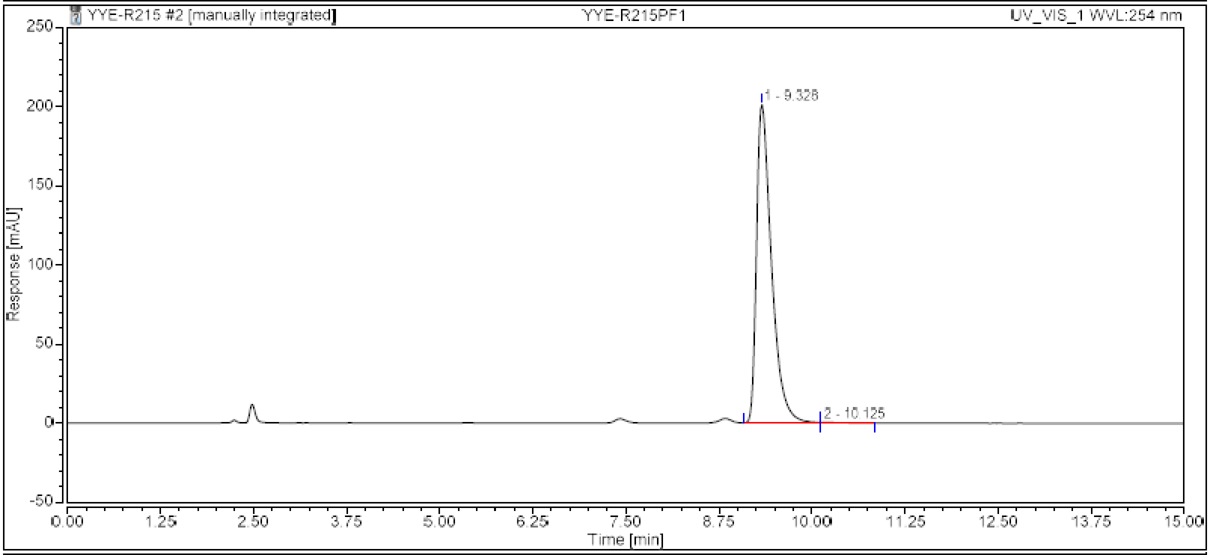

Integration Results

No.	Peak Name	Retention Time min	Area mAU*min	Height mAU	Relative Area %	Relative Height %	Amount n.a.
1		9.328	47.308	201.428	99.79	99.77	n.a.
2		10.125	0.100	0.459	0.21	0.23	n.a.
**Total:**			**47.408**	**201.888**	**100.00**	**100.00**	

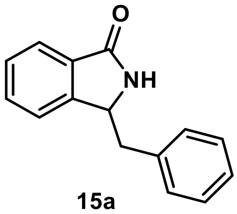

15a

The following characterization data were consistent with literature.[27]

^{1}H NMR (400 MHz, $CDCl_3$) δ 7.88 – 7.81 (m, 1H), 7.60 – 7.53 (m, 1H), 7.52 – 7.46 (m, 1H), 7.38 – 7.32 (m, 3H), 7.32 – 7.29 (m, 1H), 7.27 – 7.23 (m, 2H), 6.32 (s, 1H), 4.80 (dd, *J* = 9.3, 4.9 Hz, 1H), 3.26 (dd, *J* = 13.6, 5.0 Hz, 1H), 2.77 (dd, *J* = 13.6, 9.4 Hz, 1H).

HPLC CHIRALPAK® ID, 80:20 hexane / IPA, rate 1 mL / min, 15 min, 254 nm, t_R major 5.46 min; t_R minor 6.15 min.

Chromatogram

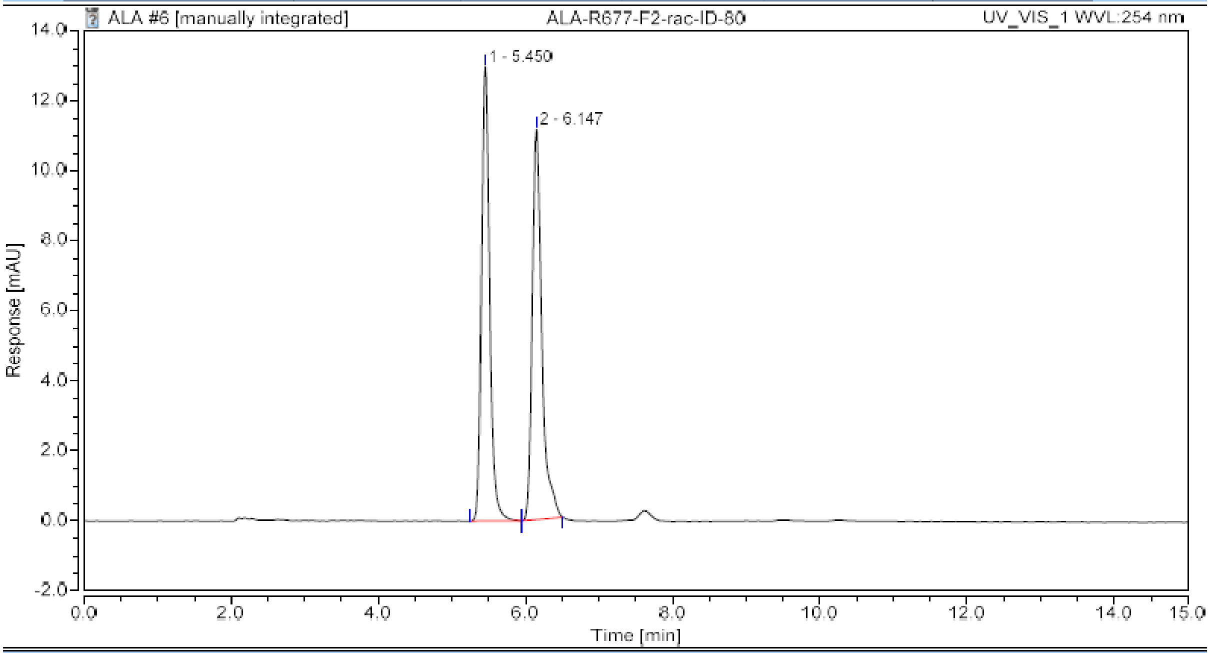

Integration Results

No.	Peak Name	Retention Time min	Area mAU*min	Height mAU	Relative Area %	Relative Height %	Amount n.a.
1		5.450	1.656	13.000	49.41	53.80	n.a.
2		6.147	1.696	11.162	50.59	46.20	n.a.
**Total:**			**3.352**	**24.162**	**100.00**	**100.00**	

For reaction entry **1**:

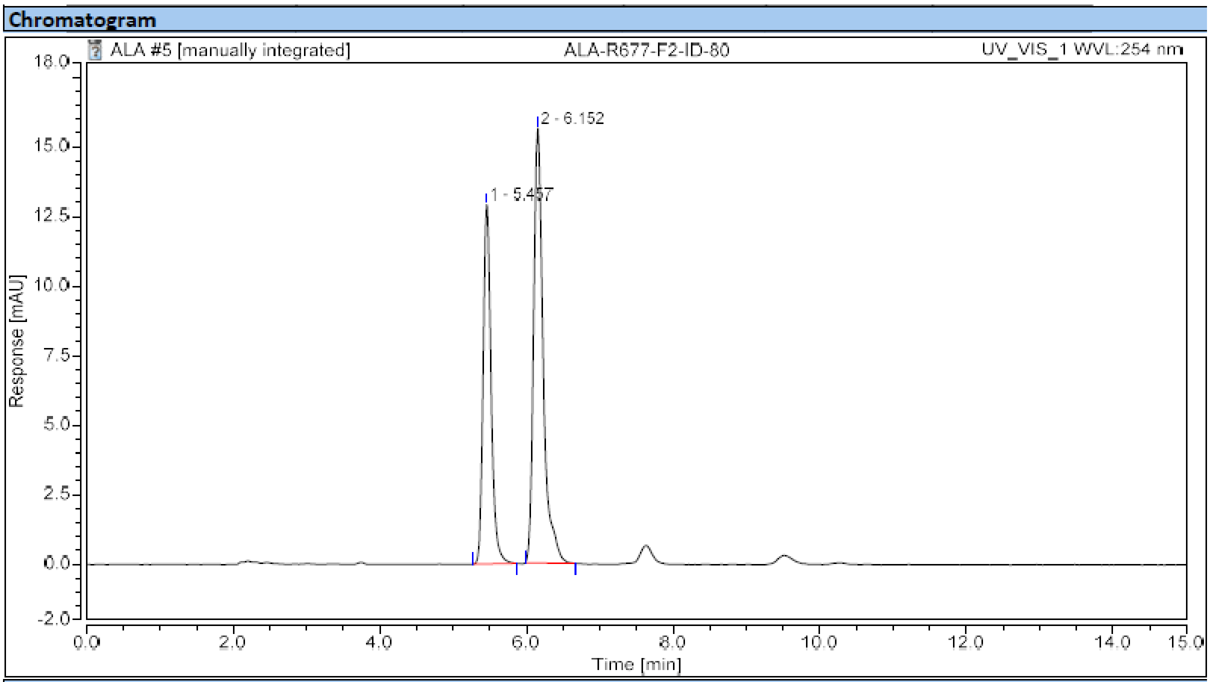

Integration Results

No.	Peak Name	Retention Time min	Area mAU*min	Height mAU	Relative Area %	Relative Height %	Amount n.a.
1		5.457	1.638	12.897	40.70	45.25	n.a.
2		6.152	2.386	15.608	59.30	54.75	n.a.
Total:			**4.024**	**28.505**	**100.00**	**100.00**	

Reaction entry 31:

General procedure **K** was carried out using 1-hexene (25 µL, 0.20 mmol), Rh(I) complex **2n** (1.3 mg, 2.0 µmol, >99% ee), temperature −20 °C (stored in freezer with occasional agitation) and duration 41 h. A 7.5 : 1 ratio of **13b** and **14b** was determined by NMR analysis of the crude mixture. Chromatographic separation (2 : 1 to 1 : 2 pentane / EtOAc gradient) afforded **14b** (first fractions) as a colorless solid (2.1 mg, 10% yield, 93% ee) and **13b** (second fractions) as a colorless oil (15.9 mg, 78% yield, 87% ee).

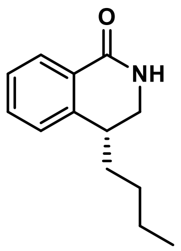

13b

Absolute configuration of **13b** was determined after reduction to amine **16b**. The following characterization data were consistent with literature.[28]

^{1}H NMR (400 MHz, $CDCl_3$) δ 8.06 (dd, *J* = 7.7, 1.5 Hz, 1H), 7.46 (td, *J* = 7.5, 1.5 Hz, 1H), 7.35 (td,

J = 7.6, 1.3 Hz, 1H), 7.21 (d, J = 6.4 Hz, 1H), 6.09 (s, 1H), 3.71 (ddd, J = 12.4, 4.4, 1.4 Hz, 1H), 3.38 (ddd, J = 12.4, 4.7, 3.3 Hz, 1H), 2.87 – 2.79 (m, 1H), 1.72 – 1.65 (m, 2H), 1.40 – 1.26 (m, 4H), 0.89 (t, J = 7.1 Hz, 3H).

HPLC CHIRALPAK® IC, 90:10 hexane / IPA, rate 1 mL / min, 45 min, 254 nm, t_R major 32.56 min; t_R minor 39.68 min.

Opt. Rot. $[\alpha]_D^{20}$ −129.17 (c = 1.20 in $CHCl_3$, 87% ee).

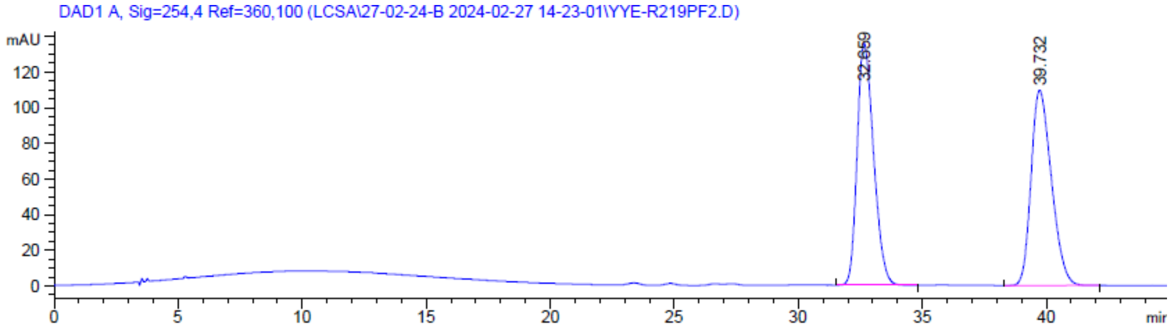

Signal 1: DAD1 A, Sig=254,4 Ref=360,100

Peak #	RetTime [min]	Type	Width [min]	Area [mAU*s]	Height [mAU]	Area %
1	32.659	BB	0.7120	6260.36328	135.76497	49.9885
2	39.732	BB	0.8924	6263.25342	109.65198	50.0115

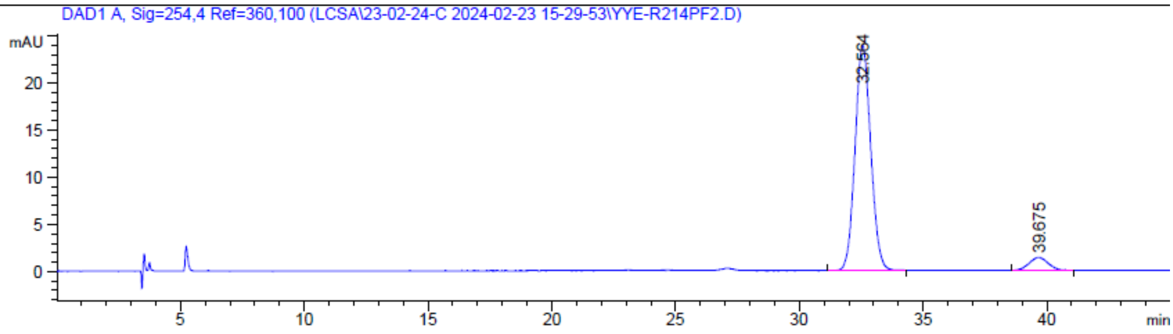

Signal 1: DAD1 A, Sig=254,4 Ref=360,100

Peak #	RetTime [min]	Type	Width [min]	Area [mAU*s]	Height [mAU]	Area %
1	32.564	BB	0.6708	1051.86633	23.83612	93.3155
2	39.675	BB	0.6580	75.34873	1.38661	6.6845

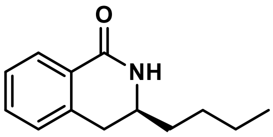

14b

Colorless crystals suitable for X-ray analysis were obtained by cooling a saturated solution of **14b** (93% ee) in pentane / DCM to −20 °C. The following characterization data were consistent

with literature.[26]

^{1}H NMR (400 MHz, $CDCl_3$) δ 8.05 (d, *J* = 6.1 Hz, 1H), 7.44 (t, *J* = 6.6 Hz, 1H), 7.34 (t, *J* = 7.5 Hz, 1H), 7.20 (d, *J* = 7.5 Hz, 1H), 5.88 (s, 1H), 3.76 – 3.62 (m, 1H), 2.97 (dd, *J* = 15.5, 4.4 Hz, 1H), 2.81 (dd, *J* = 15.6, 10.4 Hz, 1H), 1.62 – 1.59 (m, 2H), 1.43 – 1.32 (m, 4H), 0.93 (t, J = 7.1 Hz, 3H).

HPLC CHIRALPAK® IB, 90:10 hexane / IPA, rate 0.5 mL / min, 30 min, 254 nm, t_R major 13.91 min; t_R minor 15.02 min.

Opt. Rot. $[\alpha]_D^{20}$ +55.95 (c = 0.14 in $CHCl_3$, 93% ee). Literature for opposite enantiomer: $[\alpha]_D^{20}$ −33.5 (c = 1.00 in $CHCl_3$, 91% ee).

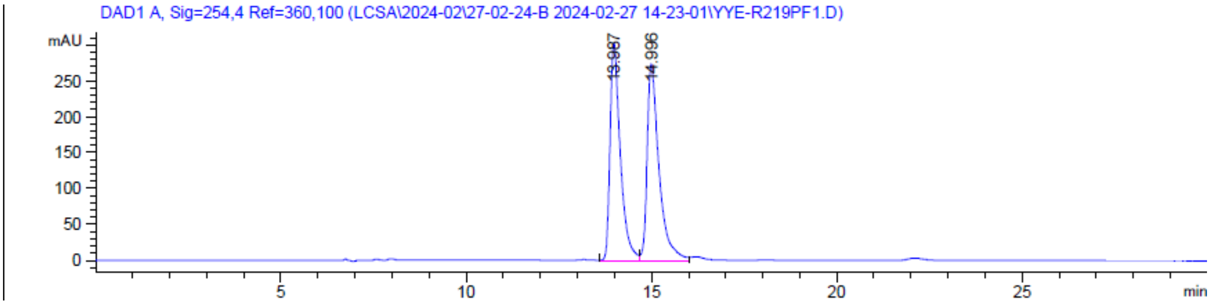

Signal 1: DAD1 A, Sig=254,4 Ref=360,100

Peak #	RetTime [min]	Type	Width [min]	Area [mAU*s]	Height [mAU]	Area %
1	13.987	BV	0.2855	5793.80518	302.40100	48.9151
2	14.996	VV	0.3272	6050.81738	272.34119	51.0849

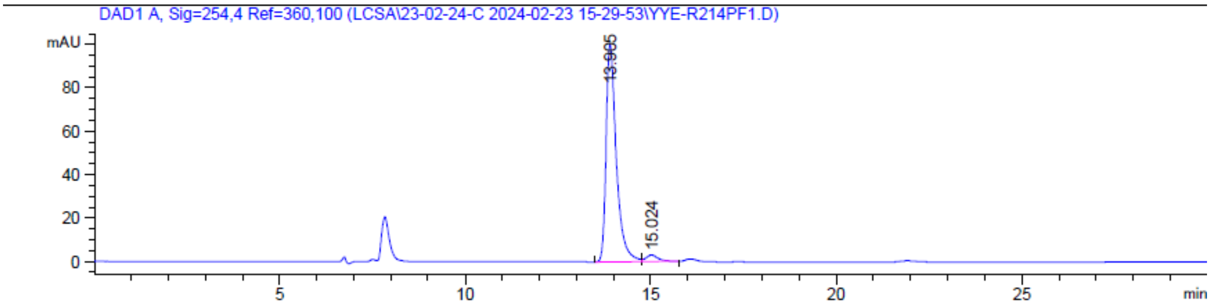

Signal 1: DAD1 A, Sig=254,4 Ref=360,100

Peak #	RetTime [min]	Type	Width [min]	Area [mAU*s]	Height [mAU]	Area %
1	13.905	BV	0.2854	1918.61121	100.21139	96.5160
2	15.024	VB	0.3386	69.25737	3.03066	3.4840

Reaction entry 35:

General procedure **K** was carried out using allylbenzene (27 µL, 0.20 mmol), Rh(I) complex **2n** (1.3 mg, 2.0 µmol, >99% ee), temperature −20 °C (stored in freezer with occasional agitation)

and duration 41 h. A 5.3 : 1 ratio of **13c** and **14c** was determined by NMR analysis of the crude mixture. Chromatographic separation (2 : 1 to 1 : 2 pentane / EtOAc gradient) afforded **14c** (first fractions) as a colorless oil (3.8 mg, 16% yield, 89% ee) and **13c** (second fractions) as a colorless oil (19.2 mg, 81% yield, 90% ee).

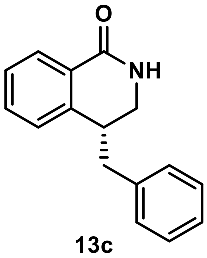

13c

Absolute configuration of **13c** was determined after reduction to amine **16c**. The following characterization data were consistent with literature.[29]

^{1}H NMR (400 MHz, $CDCl_3$) δ 8.11 (dd, *J* = 7.5, 1.6 Hz, 1H), 7.43 (td, *J* = 7.4, 1.7 Hz, 1H), 7.38 (td, *J* = 7.5, 1.5 Hz, 1H), 7.34 – 7.28 (m, 2H), 7.26 – 7.22 (m, 1H), 7.17 – 7.12 (m, 2H), 7.10 (d, *J* = 5.9 Hz, 1H), 6.05 (s, 1H), 3.60 (ddd, *J* = 12.4, 4.3, 1.2 Hz, 1H), 3.33 – 3.25 (m, 1H), 3.15 – 3.07 (m, 1H), 3.04 – 2.87 (m, 2H).

HPLC CHIRALPAK® IA, 80:20 hexane / IPA, rate 1 mL / min, 15 min, 254 nm, t_R major 4.46 min; t_R minor 5.25 min.

Opt. Rot. $[\alpha]_D^{20}$ −167.91 (c = 1.34 in $CHCl_3$, 90% ee).

Chromatogram

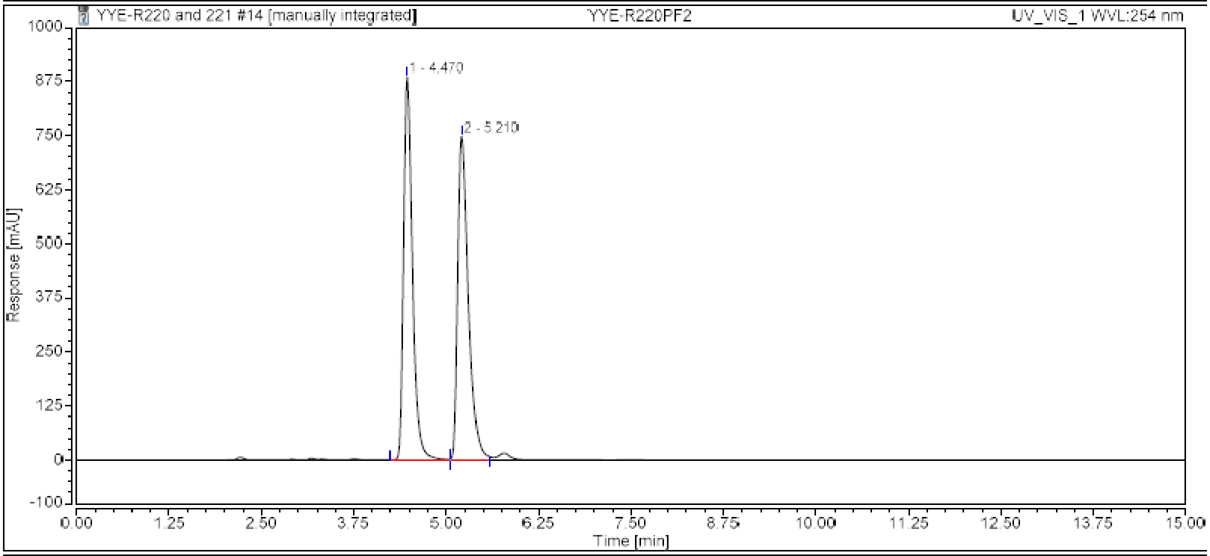

Integration Results

No.	Peak Name	Retention Time min	Area mAU*min	Height mAU	Relative Area %	Relative Height %	Amount n.a.
1		4.470	123.610	885.537	49.79	54.19	n.a.
2		5.210	124.644	748.555	50.21	45.81	n.a.
**Total:**			**248.254**	**1634.092**	**100.00**	**100.00**	

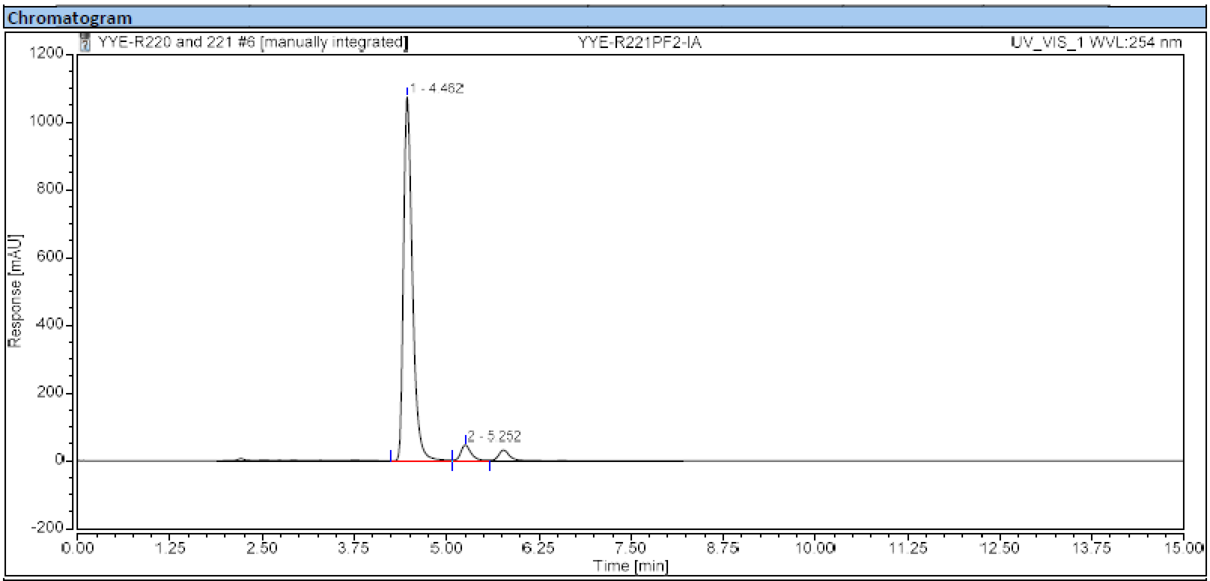

Integration Results

No.	Peak Name	Retention Time min	Area mAU*min	Height mAU	Relative Area %	Relative Height %	Amount n.a.
1		4.462	150.399	1074.876	95.15	95.83	n.a.
2		5.252	7.671	46.775	4.85	4.17	n.a.
**Total:**			**158.070**	**1121.651**	**100.00**	**100.00**	

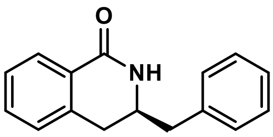

14c

The following characterization data were consistent with literature.[30]

^{1}H NMR (400 MHz, $CDCl_3$) δ 8.06 (dd, *J* = 7.6, 1.6 Hz, 1H), 7.46 (td, *J* = 7.5, 1.5 Hz, 1H), 7.39 – 7.32 (m, 3H), 7.32 – 7.26 (m, 1H), 5.74 (s, 1H), 4.01 – 3.89 (m, 1H), 3.06 – 2.86 (m, 3H), 2.81 (dd, *J* = 13.6, 8.8 Hz, 1H).

HPLC CHIRALPAK® ID, 80:20 hexane / IPA, rate 1 mL / min, 15 min, 254 nm, t_R major 6.90 min; t_R minor 7.44 min.

Opt. Rot. $[\alpha]_D^{20}$ +43.06 (c = 0.12 in $CHCl_3$, 89% ee). Literature: $[\alpha]_D^{20}$ +118.9 (c = 0.14 in $CHCl_3$, 99% ee).

Chromatogram

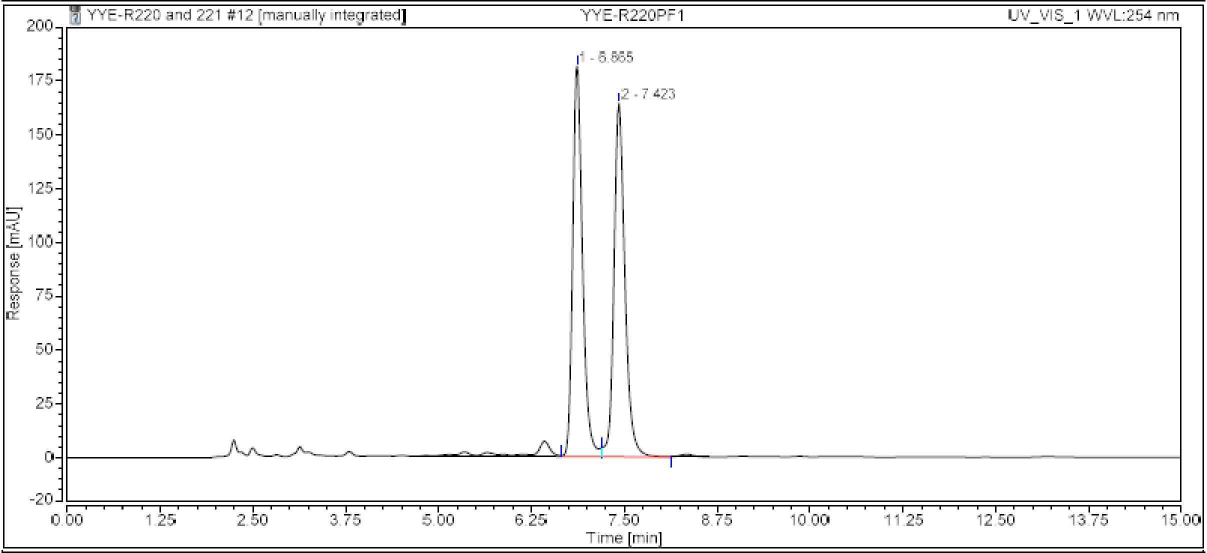

Integration Results

No.	Peak Name	Retention Time min	Area mAU*min	Height mAU	Relative Area %	Relative Height %	Amount n.a.
1		6.865	28.063	181.346	49.76	52.46	n.a.
2		7.423	28.339	164.354	50.24	47.54	n.a.
**Total:**			**56.403**	**345.700**	**100.00**	**100.00**	

Chromatogram

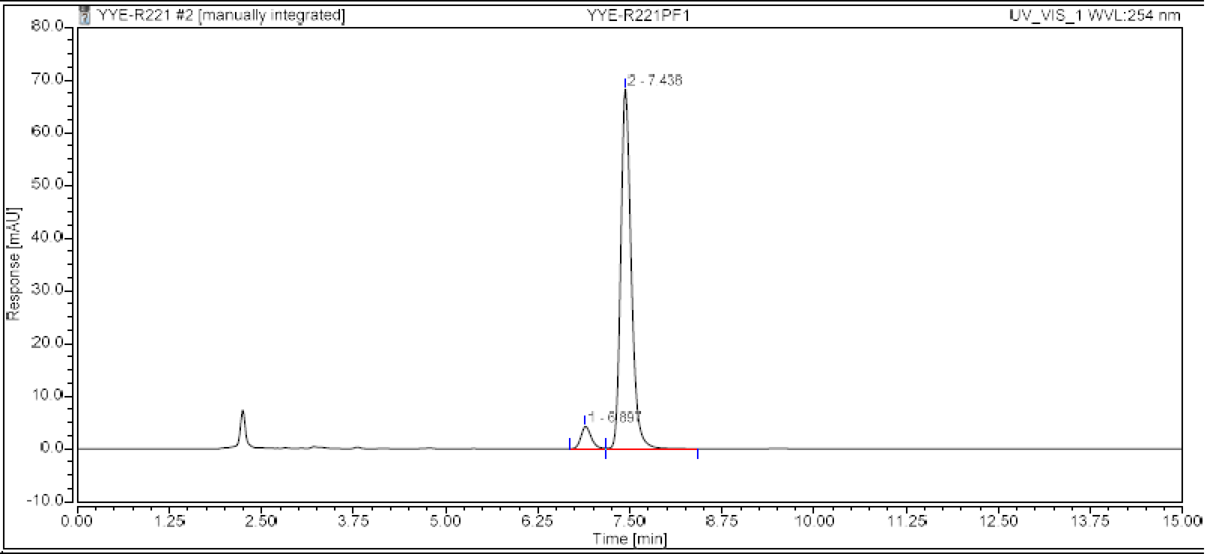

Integration Results

No.	Peak Name	Retention Time min	Area mAU*min	Height mAU	Relative Area %	Relative Height %	Amount n.a.
1		6.897	0.690	4.271	5.75	5.89	n.a.
2		7.438	11.320	68.286	94.25	94.11	n.a.
**Total:**			**12.011**	**72.558**	**100.00**	**100.00**	

Reaction entry 36:

General procedure **K** was carried out using allylbenzene (27 µL, 0.20 mmol), Rh(I) complex **2n** (1.3 mg, 2.0 µmol, >99% ee), temperature 23 °C and duration 3 h. A 1 : 5.9 : 3.4 ratio of unreacted **12**, **13d** and **14d** was determined by NMR analysis of the crude mixture. Chromatographic separation (20 : 1 EtOAc / MeOH) afforded unreacted **12** (first fractions) as a colorless solid (1.8 mg, 8%), **14d** (second fractions) as a colorless oil (4.8 mg, 27% yield, 94%

ee) and **13d** (third fractions) as a colorless solid (9.1 mg, 51% yield, 90% ee).

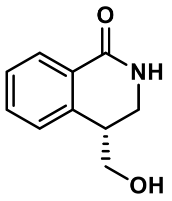

13d

Colorless crystals suitable for X-ray analysis were obtained by cooling a saturated solution of **13d** (90% ee) in pentane / DCM to −20 °C. The following characterization data were consistent with literature.[29]

^{1}H NMR (400 MHz, $CDCl_3$) δ 8.06 (d, *J* = 7.7 Hz, 1H), 7.48 (t, *J* = 7.5 Hz, 1H), 7.38 (t, *J* = 7.6 Hz, 1H), 7.28 (s, 1H), 6.60 (s, 1H), 3.81 – 3.65 (m, 4H), 3.06 (s, 1H), 2.63 (s, 1H).

SFC CHIRALPAK® IA, 10% MeOH in CO_2, rate 1 mL / min, 5 min, 230 nm, t_R minor 2.47 min; t_R major 2.66 min.

Opt. Rot. $[\alpha]_D^{20}$ −130.86 (c = 0.54 in EtOH, 90% ee).

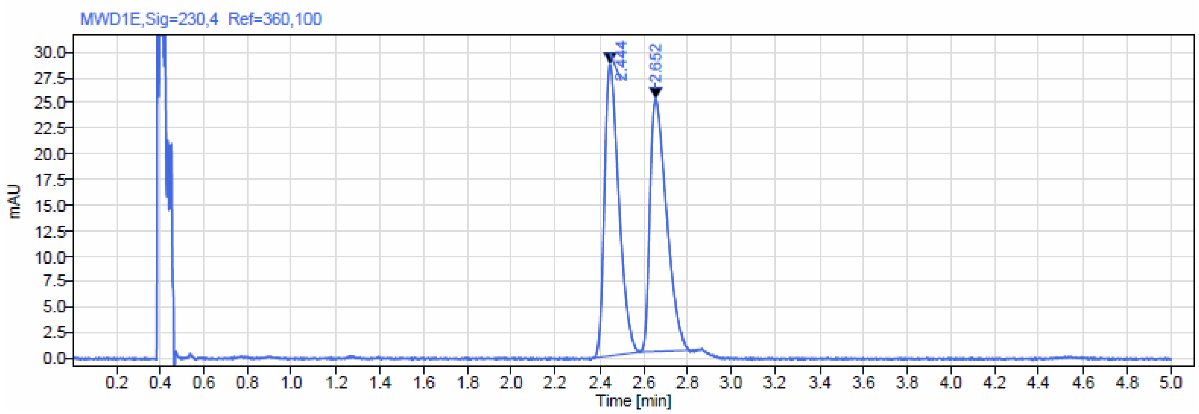

Signal: MWD1E,Sig=230,4 Ref=360,100

RT [min]	Type	Width [min]	Area	Height	Area%	Name
2.444	MM m	0.22	130.37	28.49	49.90	
2.652	MM m	0.22	130.89	24.64	50.10	
		**Sum**	**261.26**			

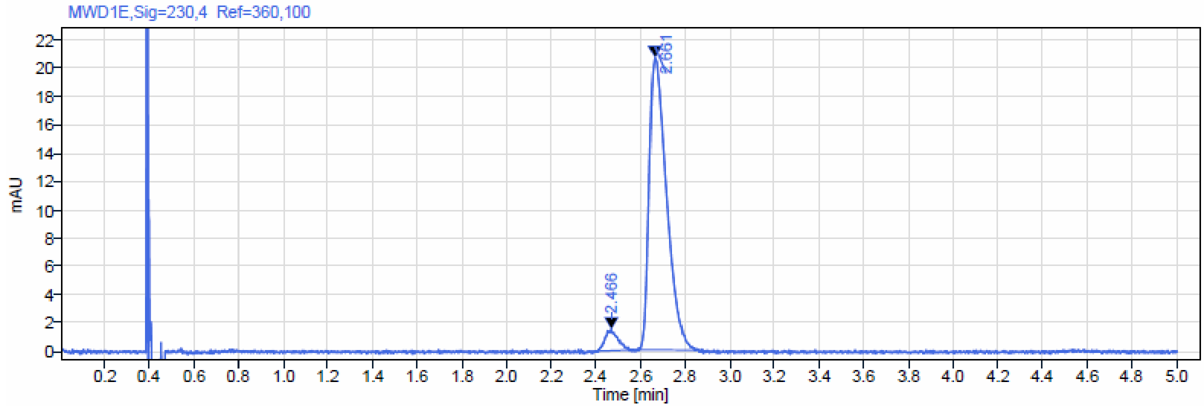

Signal: MWD1E,Sig=230,4 Ref=360,100

RT [min]	Type	Width [min]	Area	Height	Area%	Name
2.466	MM m	0.19	5.91	1.48	5.05	
2.661	MM m	0.27	111.27	20.63	94.95	
		**Sum**	**117.19**			

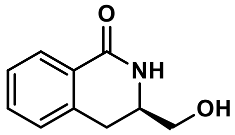

14d

The following characterization data were consistent with literature.[31]

^{1}H NMR (400 MHz, $CDCl_3$) δ 8.04 (d, J = 7.7 Hz, 1H), 7.50 – 7.31 (m, 3H), 7.21 (d, J = 7.5 Hz, 1H), 3.97 – 3.80 (m, 2H), 3.77 – 3.65 (m, 1H), 3.38 (s, 1H), 2.90 (d, J = 7.6 Hz, 2H).

SFC CHIRALPAK® IA, 10% MeOH in CO_2, rate 1 mL / min, 5 min, 230 nm, t_R major 2.62 min; t_R minor 2.85 min.

Opt. Rot. $[\alpha]_D^{20}$ +12.50 (c = 0.36 in EtOH, 94% ee). Literature: $[\alpha]_D^{20}$ +14 (c = 1.1 in EtOH, >99% ee).

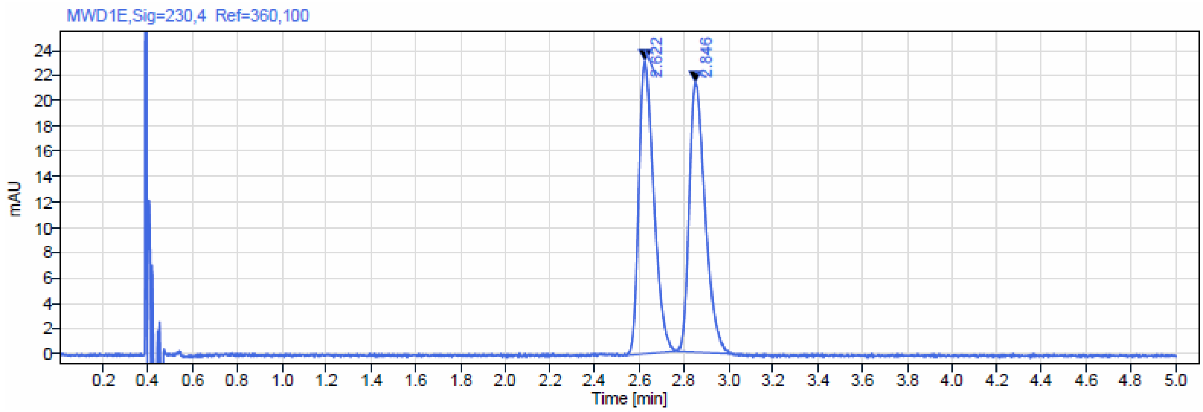

Signal:	MWD1E,Sig=230,4 Ref=360,100					
**RT [min]**	**Type**	**Width [min]**	**Area**	**Height**	**Area%**	**Name**
2.622	MM m	0.22	106.58	23.10	50.09	
2.846	MM m	0.27	106.21	21.24	49.91	
		**Sum**	**212.79**			

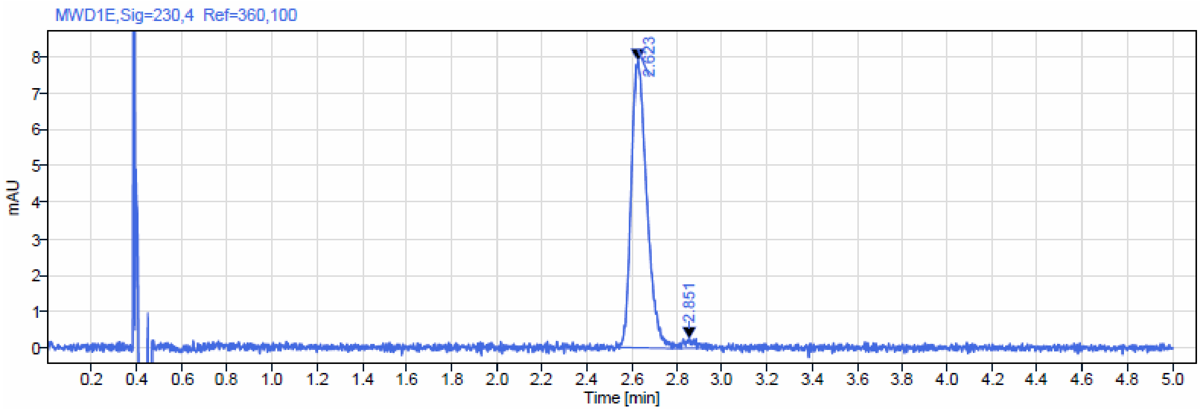

Signal:	MWD1E,Sig=230,4 Ref=360,100					
**RT [min]**	**Type**	**Width [min]**	**Area**	**Height**	**Area%**	**Name**
2.623	MM m	0.24	37.36	7.85	97.03	
2.851	MM m	0.19	1.14	0.25	2.97	
		**Sum**	**38.50**			

General procedure L for synthesis of tetrahydroisoquinolines 16

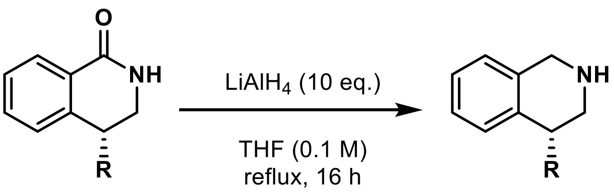

13b or **13c** **16b** or **16c**

A solution of dihydroisoquinolone **13** in dry THF (0.1 M) was treated with $LiAlH_4$ (10 eq.) at 0°C, then heated to reflux with stirring for 16 h. The reaction mixture was quenched by slow

addition of $Na_2SO_4.10H_2O$ at 0°C until evolution of H_2 bubbles ceased. The mixture was filtered through celite, washing with Et_2O (3 × equivolume to THF) and concentrated *in vacuo*. The resulting crude residue was subjected to column chromatography (silica gel, 100:1 EtOAc / NH_3 sat. MeOH) to afford **16**.

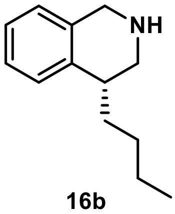

16b

Tetrahydroisoquinoline **16b** was prepared via general procedure **L** with **13b** (11.1 mg, 54.6 µmol, 83% ee) and $LiAlH_4$ (20.7 mg, 0.546 mmol) to give a clear oil (8.5 mg, 82% yield, 83% ee based on **13b**). The following characterization data were consistent with literature.[32]

^{1}H NMR (400 MHz, $CDCl_3$) δ 7.20 – 7.08 (m, 3H), 6.99 (d, *J* = 6.9 Hz, 1H), 3.99 (s, 2H), 3.13 (dd, *J* = 12.9, 4.8 Hz, 1H), 3.01 (dd, *J* = 13.0, 4.5 Hz, 1H), 2.73 – 2.62 (m, 1H), 1.88 (s, 1H), 1.74 – 1.59 (m, 2H), 1.45 – 1.29 (m, 4H), 0.93 (t, *J* = 7.1 Hz, 3H).

Opt. Rot. $[\alpha]_D^{25}$ +15.49 (c = 0.85 in MeOH, 83% ee). Literature: $[\alpha]_D^{25}$ +22.2 (c = 1.1 in MeOH, >99% ee).

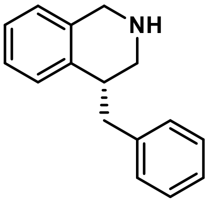

16c

Tetrahydroisoquinoline **16c** was prepared via general procedure **L** with **13c** (23.7 mg, 0.100 mmol, 89% ee) and $LiAlH_4$ (38.0 mg, 1.00 mmol) to give a clear oil (13.9 mg, 62% yield, 89% ee based on **13c**). The following characterization data were consistent with literature.[33]

^{1}H NMR (400 MHz, $CDCl_3$) δ 7.32 (t, *J* = 7.1 Hz, 2H), 7.26 – 7.13 (m, 6H), 7.06 – 7.02 (m, 1H), 4.02 (s, 2H), 3.10 (dd, *J* = 13.2, 4.3 Hz, 1H), 3.04 – 2.94 (m, 3H), 2.89 (dd, *J* = 13.2, 9.4 Hz, 1H), 2.02 (s, 1H).

Opt. Rot. $[\alpha]_D^{25}$ −27.22 (c = 1.39 in $CHCl_3$, 89% ee). Literature: $[\alpha]_D^{25}$ −32.86 (c = 1.3 in $CHCl_3$, >99% ee).

6. Computational Details

To probe regio- and enantioselectivity, we employed a variation of our previous computational workflow that successfully reproduced experimental enantioselectivities of C_2-symmetric Cp derivatives in the rhodium-catalyzed C-H functionalization of hydroxamic acid derivatives.[34,35] We began from a set of eight preconstructed "parent" transition state templates representing different binding motifs of the alkene with the catalyst (see **Figure S1** for examples with 1-hexene) that lead to the different enantiomers of 3- and 4-substituted products. To generate sets of conformers that consider the specific steric interactions present between the substrate and the ligand, we used the SCINE Molassembler library.[36,37] Molassembler is a graph-based software containing algorithms for constructing molecules possessing all elements of the periodic table that encodes structural information as graphs. Here, Molassembler is used to interpret the three-dimensional coordinates of the "parent" TS conformers into a reference graph, upon which the chemical modifications associated with the ligand and substrate of interest are conducted. Sets of 400 different conformers were then generated and projected back to three-dimensional coordinates with full stereoisomer control with the stereocenter configurations being extracted from the "parent" TS templates. During this process, only those conformers possessing chemically reasonable structures (i.e., those without geometrically overlapping chemical moieties) were retained. To aid in sampling, during this process the alkane tail of 1-hexene was kept frozen in a zig-zag conformation to minimize the sampling of rotation within this moiety (no constraints were used for styrene).

From the 3200 total conformers generated for each ligand/substrate combination, the chemically reasonable structures were retained for geometric optimization. Keeping the main bond distances associated with the transition state frozen, we performed a series of constrained optimizations, first at the semiempirical PM7[38] level, followed by dispersion-corrected DFT at the PBE0[39,40]-D3(BJ)[41,42]/def2-SVP[43] level. Following these constrained optimizations, the transition states of all structures were fully optimized at the PBE0-D3(BJ)/def2-SVP level (as confirmed by analysis of the vibrational frequencies) followed by single point computations at the PBE0-D3(BJ)/def2-TZVP[43] level in implicit ethanol solvent using the SMD solvation model.[44] Final reported free energies (available in the SI .dat file) include electronic energies from the PBE0-D3(BJ)/def2-TZVP single point computations and

free energy corrections from the PBE0-D3(BJ)/def2-SVP computations. To determine regio- and enantioselectivities, we individually fed the conformer ensembles associated with each pathway (i.e., 3DR, 3DS, 3UR, 3US, 4DR, 4DS, 4UR, 4US) into *marc* (a conformer analysis program),[45] which performed k-means clustering and identified the ten most representative conformers for each of the aforementioned pathways. Those conformers found to be within < 4.0 kcal/mol from the lowest energy species in their respective pathway were retained and used to determine effective ΔG values via Boltzmann weighting at 296K. These ΔG values were subsequently used to determine regio-/enantioselectivity ratios from the theoretical kinetic constants:

$$k_{4/3} = \exp\left(\frac{-\Delta G^{TS}_{eff,4/3}}{kT}\right) \text{ as } rr_{4/3} = 100 \times \frac{k_{4/3}}{k_4 + k_3}$$

and

$$k_{S/R} = \exp\left(\frac{-\Delta G^{TS}_{eff,S/R}}{kT}\right) \text{ as } er_{S/R} = 100 \times \frac{k_{S/R}}{k_S + k_R}\text{, respectively.}$$

Figure S1. Eight binding motifs between a 1-hexene substrate and the catalyst and their ultimate products. These orientations are associated with the eight parent TS structures used to generate the initial conformer library.

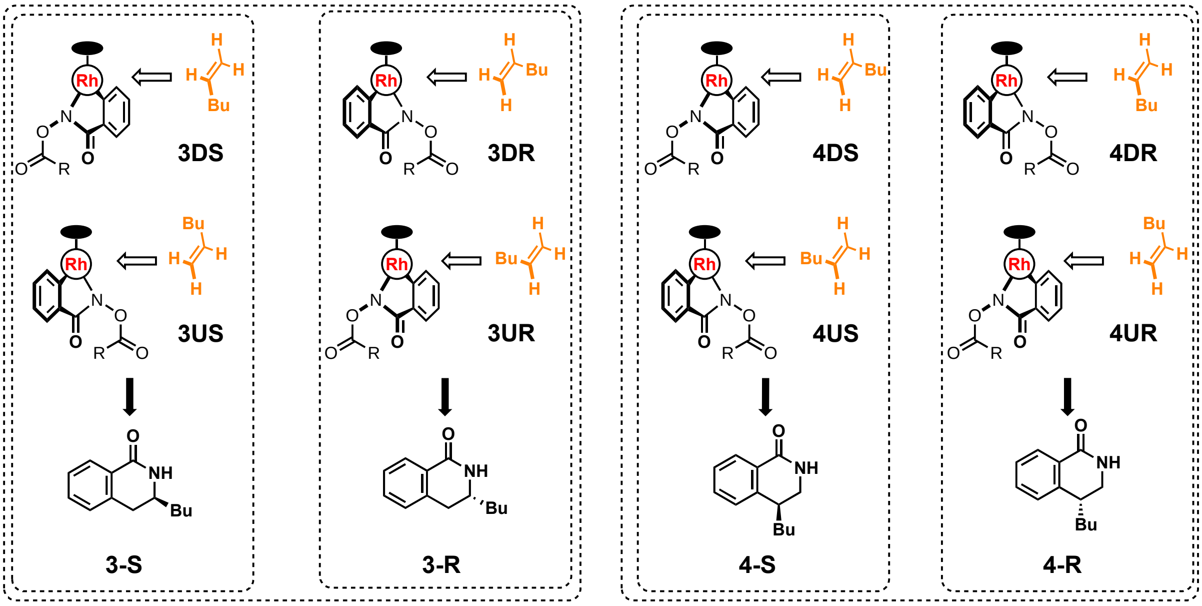

A comparison of the results shows good agreement between computed effective and

experimental ΔG values (**Table S2** and **Figure S2**), as indicated by Pearson correlation coefficient (r) of 0.89 and an R^2 of 0.79. If one considers that predictions of "nonselective" catalysts (points falling within the red box in **Figure S2**) are less important than predictions of selective species (points appearing outside the red box), than only a single example was found to have a qualitatively incorrect selectivity prediction (i.e., falling in the wrong quadrant). Quantitative analysis (**Table S2**) indicates that our computational protocol contains no systematic errors, with a mean unsigned deviation (MUD) of 0.25 kcal/mol, and the overall accuracy of our predictions, as determined by the mean signed deviation), is 1.02 kcal/mol.

Figure S2. Comparison of computed and experimentally determined selectivities. The experimental regioselectitives of Rh1-Rh3 with the styrene substrate showed 100:0 favoring the 3-regioisomer. Computations showed very large effective ΔG values, consistent with experimental. For the sake of this comparison, the experimental ΔG values have been set equivalent to the computed values.

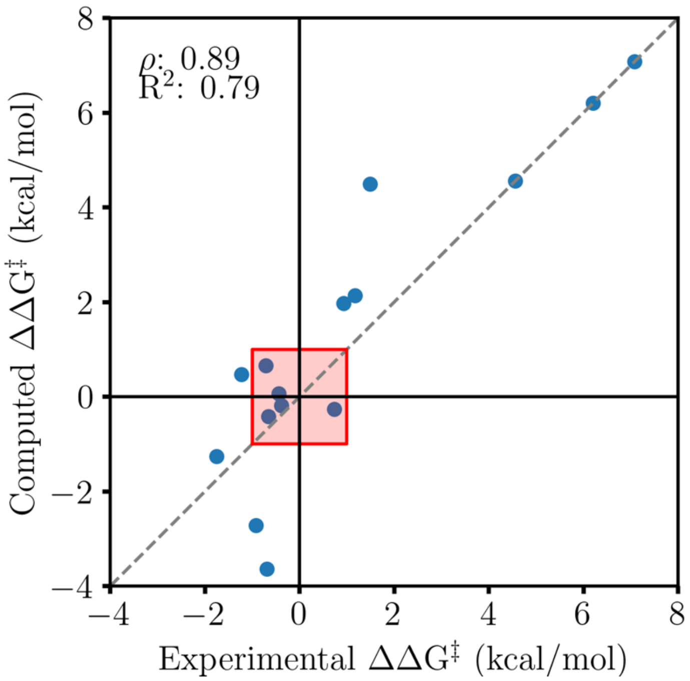

Table S2. Experimentally determined and computed effective ΔG values (kcal/mol). Rh1 = **2d**, Rh2 = **2g**, Rh3 = **2j**.

	Experimental ΔG	Computed ΔG	Signed Deviation (SD)	Absolute Deviation (AD)
Rh1-Styrene-Regio (3:4)	7.08	7.08	0.00	0.00
Rh1-Styrene-Major (R:S)	1.17	2.14	0.97	0.97

Rh2-Styrene-Regio (3:4)	4.56	4.56	0.00	0.00
Rh2-Styrene-Major (R:S)	-0.71	0.66	1.37	1.37
Rh3-Styrene-Regio (3:4)	6.20	6.20	0.00	0.00
Rh3-Styrene-Major (R:S)	1.49	4.49	3.00	3.00
Rh1-Hexene-Regio (3:4)	-0.92	-2.72	-1.80	1.80
Rh1-Hexene-Major (R:S)	0.73	-0.26	-0.99	0.99
Rh1-Hexene-Minor (R:S)	-0.66	-0.42	0.24	0.24
Rh2-Hexene-Regio (3:4)	-1.75	-1.26	0.49	0.49
Rh2-Hexene-Major (R:S)	-0.39	-0.18	0.21	0.21
Rh2-Hexene-Minor (R:S)	-0.44	0.07	0.51	0.51
Rh3-Hexene-Regio (3:4)	-0.69	-3.64	-2.95	2.95
Rh3-Hexene-Major (R:S)	0.93	1.97	1.04	1.04
Rh3-Hexene-Minor (R:S)	-1.23	0.47	1.70	1.70
			MSD = 0.25	MAD = 1.02

Table S3. Computed Boltzmann weighted relative free energies for the lowest energy conformers for each of the eight possible transition state orientations and effective free energies (obtained by Boltzmann weighting) associated with enantioselectivity and regioselectivity using styrene as a substrate.

Conformer	Effective ΔG for Enantioselectivity (kcal/mol)	Effective ΔG for Regioselectivity (kcal/mol)
**Rh1-Styrene ([Rh] = 2d and R = Ph)**		
Rh1-Styrene-3DR	0.00	0.00
Rh1-Styrene-3UR		
Rh1-Stryene-3DS	2.14	
Rh1-Styrene-3US		
Rh1-Styrene-4DR	0.00	7.08
Rh1-Styrene-4UR		
Rh1-Styrene-4DS	1.47	
Rh1-Styrene-4US		
**Rh2-Styrene ([Rh] = 2g and R = Ph)**		
Rh2-Styrene-3DR	0.00	0.00
Rh2-Styrene-3UR		
Rh2-Stryene-3DS	0.66	
Rh2-Styrene-3US		
Rh2-Styrene-4DR	0.00	4.56
Rh2-Styrene-4UR		
Rh2-Styrene-4DS	0.38	
Rh2-Styrene-4US		
**Rh3-Styrene ([Rh] = 2j and R = Ph)**		

Rh3-Styrene-3DR	0.00	0.00
Rh3-Styrene-3UR		
Rh3-Stryene-3DS	4.49	
Rh3-Styrene-3US		
Rh3-Styrene-4DR	0.00	6.20
Rh3-Styrene-4UR		
Rh3-Styrene-4DS	0.67	
Rh3-Styrene-4US		

Table S4. Computed Boltzmann weighted relative free energies for the conformers for each of the eight possible transition state orientations and effective free energies (obtained by Boltzmann weighting) associated with enantioselectivity and regioselectivity using 1-hexene as a substrate.

Conformer	Effective ΔG for Enantioselectivity (kcal/mol)	Effective ΔG for Regioselectivity (kcal/mol)
**Rh1-Hexene ([Rh] = 2d and R = Bu)**		
Rh1-Hexene-3DS	0.00	2.72
Rh1-Hexene-3US		
Rh1-Hexene-3DR	0.66	
Rh1-Hexene-3UR		
Rh1-Hexene-4DS	0.00	0.00
Rh1-Hexene-4US		
Rh1-Hexene-4DR	0.26	
Rh1-Hexene-4UR		
**Rh1-Hexene ([Rh] = 2g and R = Bu)**		
Rh2-Hexene-3DS	0.07	1.26
Rh2-Hexene-3US		
Rh2-Hexene-3DR	0.00	
Rh2-Hexene-3UR		
Rh2-Hexene-4DS	0.00	0.00
Rh2-Hexene-4US		
Rh2-Hexene-4DR	0.18	
Rh2-Hexene-4UR		
**Rh3-Hexene ([Rh] = 2j and R = Bu)**		
Rh3-Hexene-3DS	0.47	3.64
Rh3-Hexene-3US		

Rh3-Hexene-3DR	0.00	
Rh3-Hexene-3UR		
Rh3-Hexene-4DS	1.97	0.00
Rh3-Hexene-4US		
Rh3-Hexene-4DR	0.00	
Rh3-Hexene-4UR		

Table S5. Computed regiomeric and enantiomeric ratios based on effective ΔG values.

	rr (4:3)	er(4) – (R:S)	er(3) – (R:S)
Rh1-Styrene	0:100	92.4:7.6	97.4:2.6
Rh2-Styrene	0:100	65.8:34.2	75.4:24.6
Rh3-Styrene	0:100	75.7:24.3	100:0
Rh1-Hexene	99.0:1.0	39.2:60.8	32.9:67.1
Rh2-Hexene	89.5:10.5	42.4:57.6	47.0:53.0
Rh3-Hexene	99.8:0.2	96.6:3.4	68.9:31.1

7. NMR Spectra

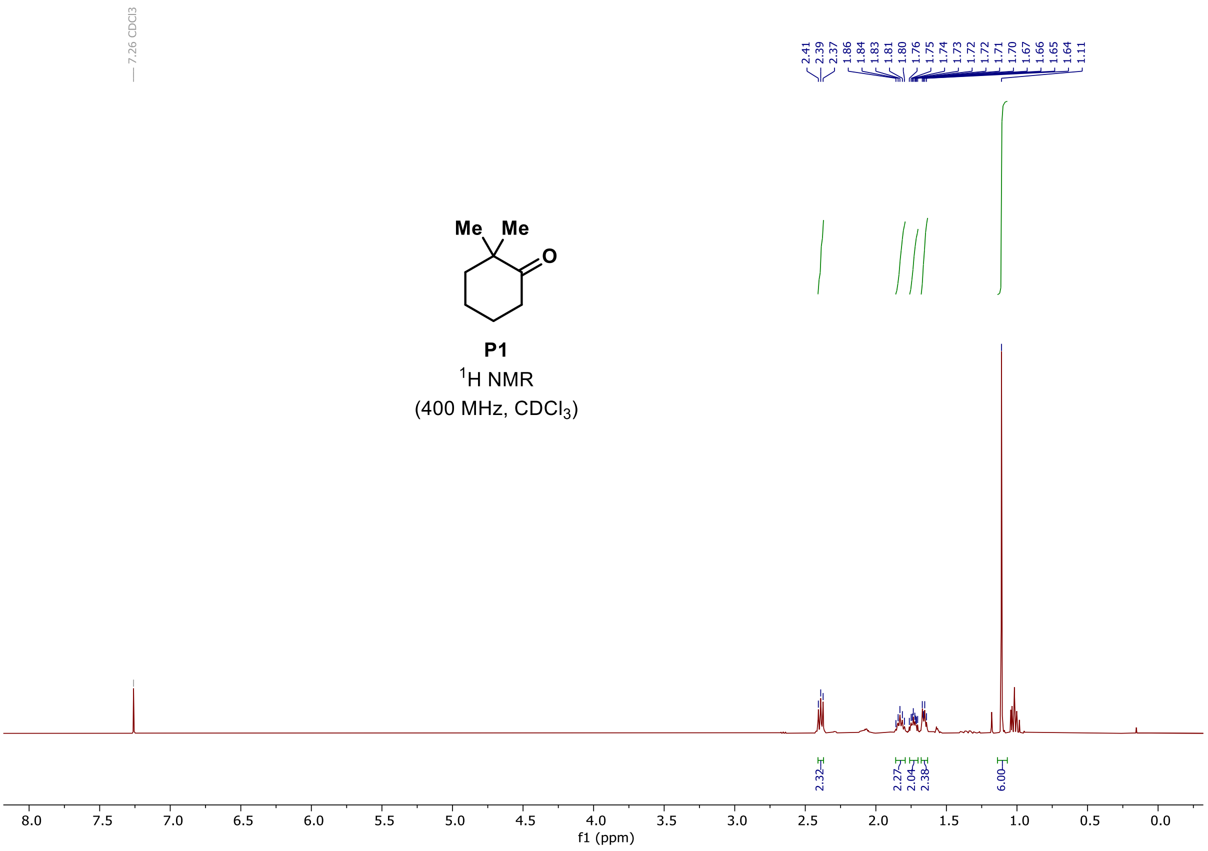

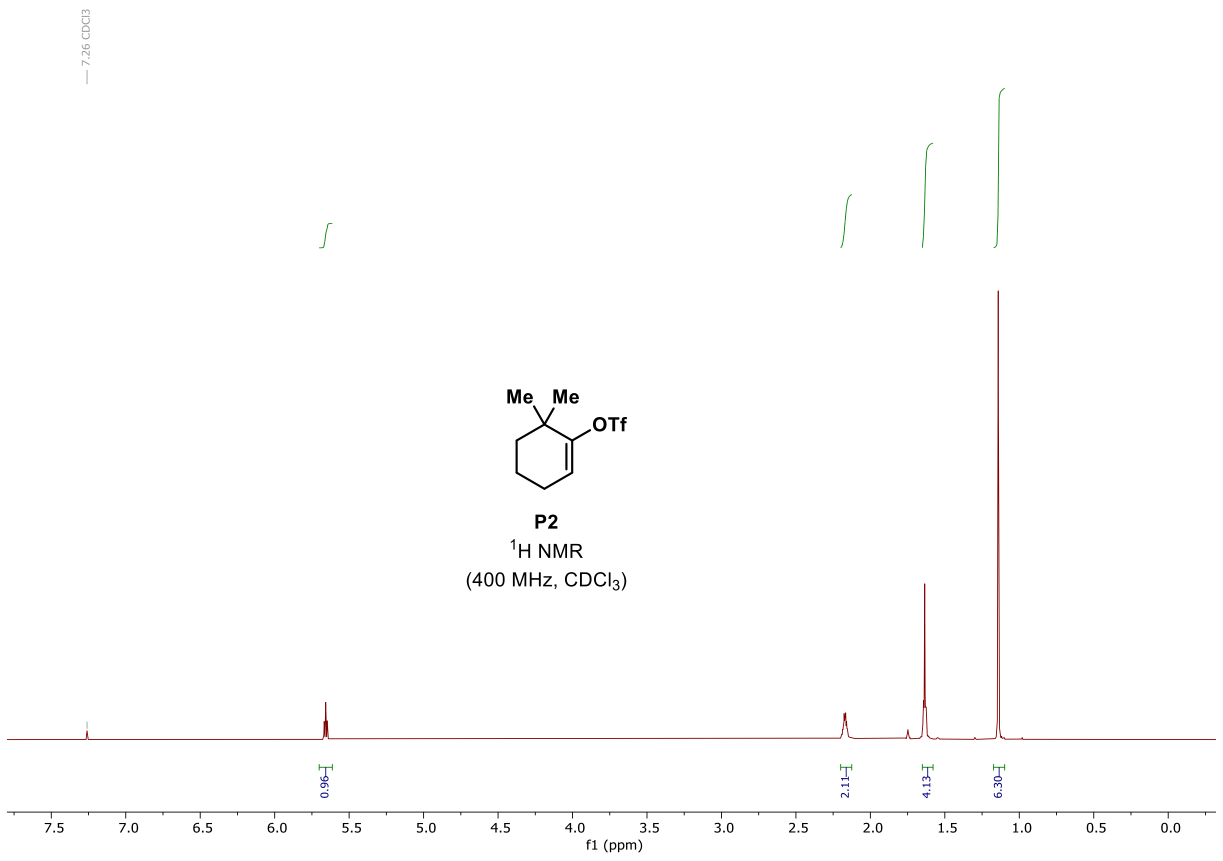

7.26 CDCl3
Me
Me
OTf
P2
1H NMR
(400 MHz, CDCl3)
0.96
2.11
4.13
6.30
7.5
7.0
6.5
6.0
5.5
5.0
4.5
4.0
3.5
3.0
2.5
2.0
1.5
1.0
0.5
0.0
f1 (ppm)

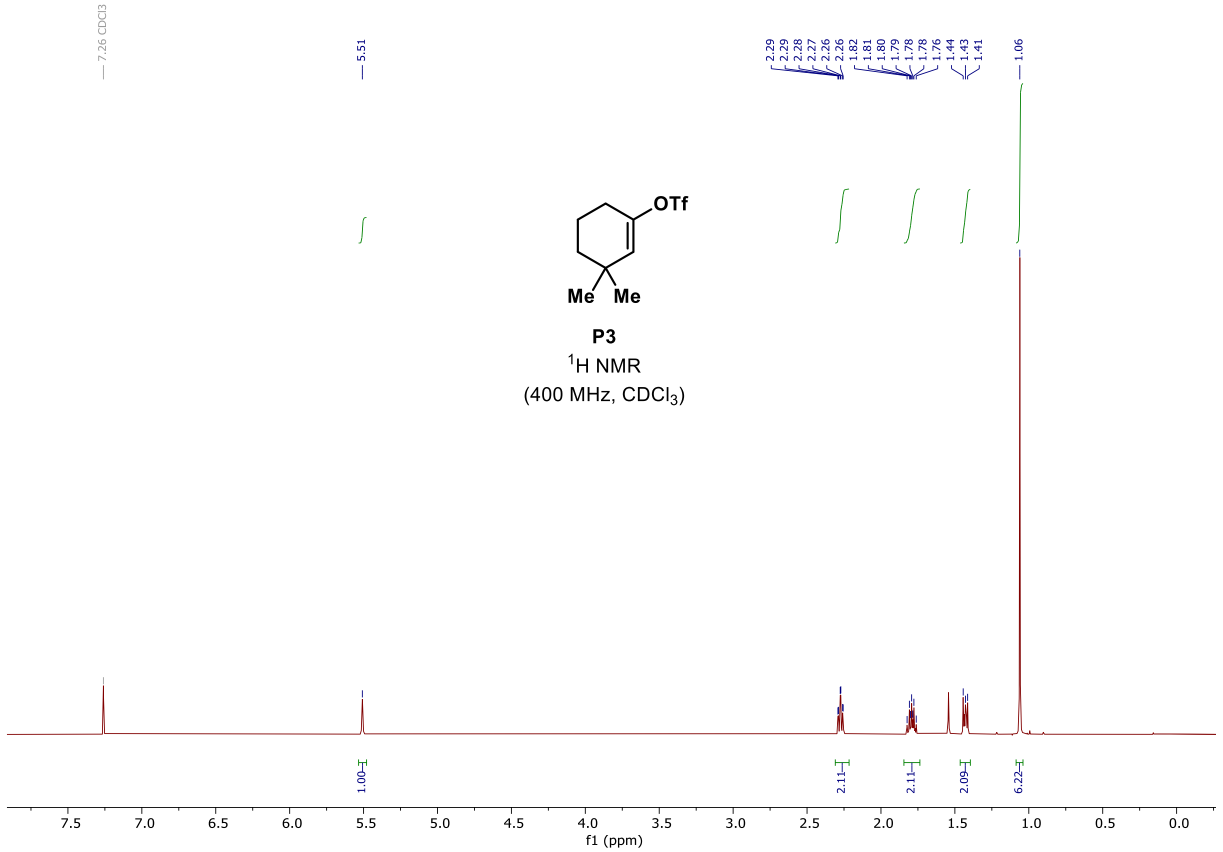

7.26 CDCl3
5.51
2.29
2.29
2.28
2.27
2.26
2.26
1.82
1.81
1.80
1.79
1.78
1.78
1.76
1.44
1.43
1.41
1.06
OTf
Me
Me
P3
1H NMR
(400 MHz, CDCl3)
1.00
2.11
2.11
2.09
6.22
7.5
7.0
6.5
6.0
5.5
5.0
4.5
4.0
3.5
3.0
2.5
2.0
1.5
1.0
0.5
0.0
f1 (ppm)

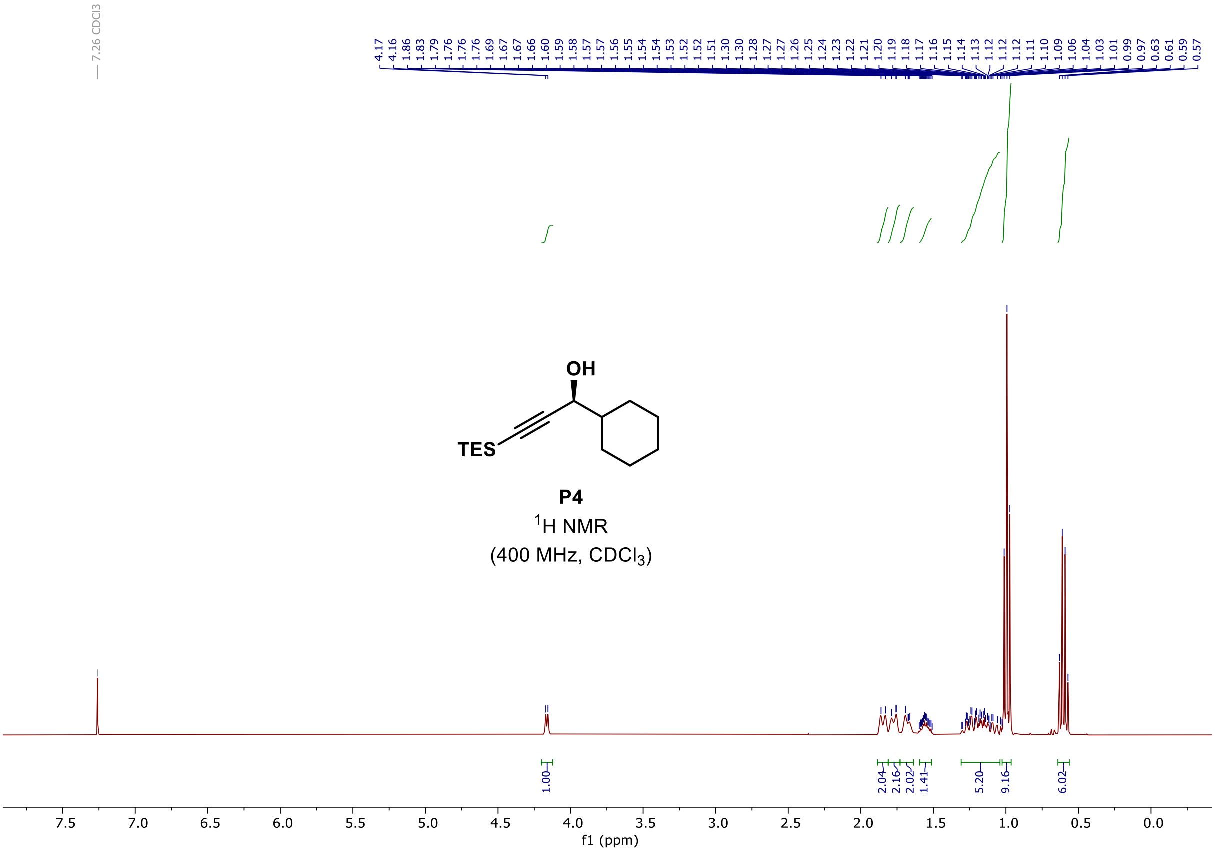

P4

^{1}H NMR

(400 MHz, $CDCl_3$)

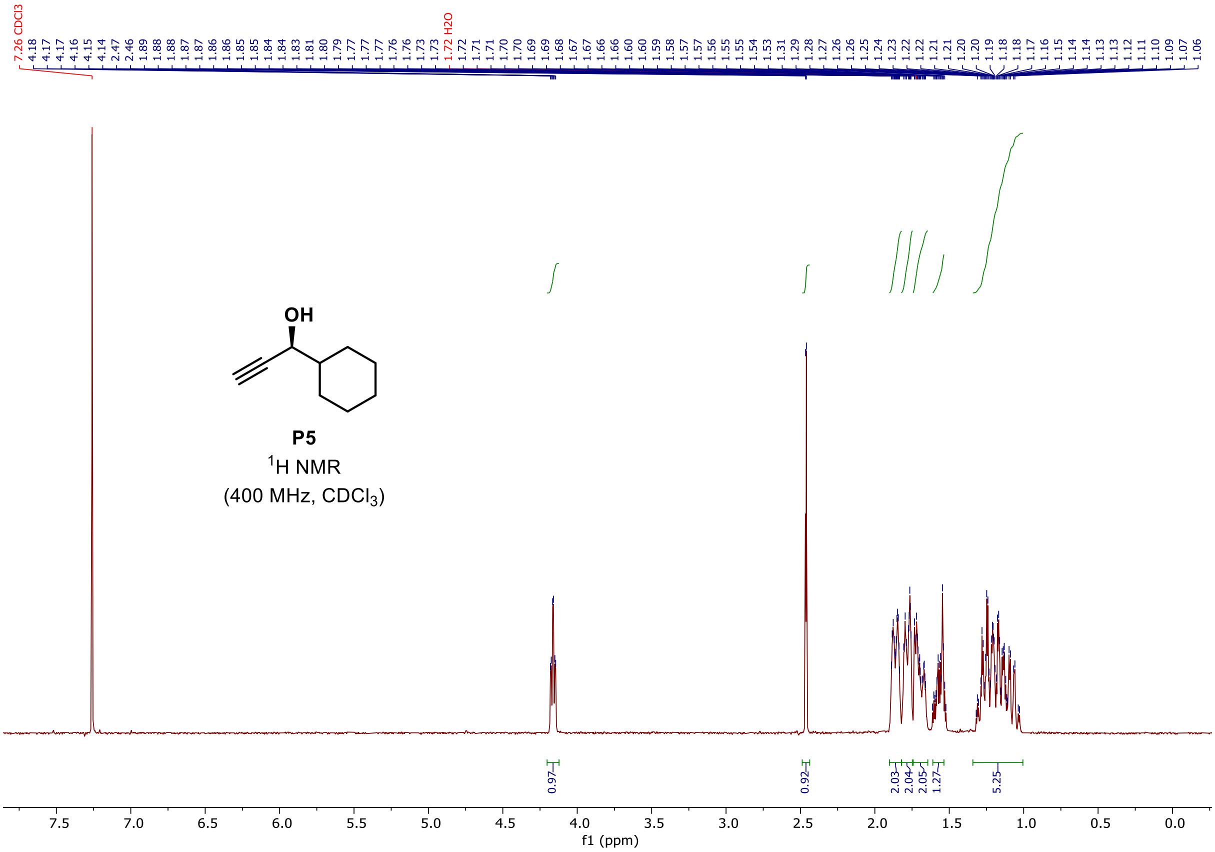
OH
P5
1H NMR
(400 MHz, CDCl3)
f1 (ppm)

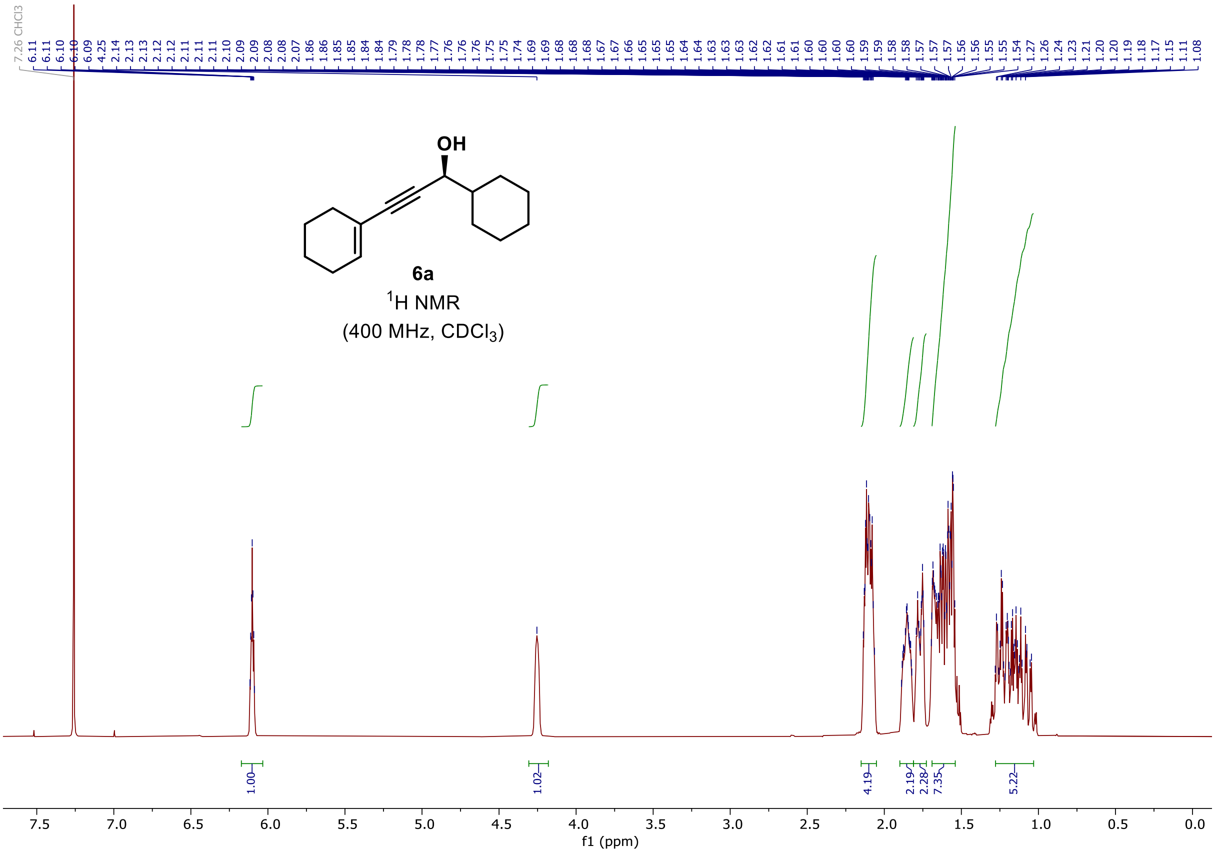
OH
6a
1H NMR
(400 MHz, CDCl3)
f1 (ppm)

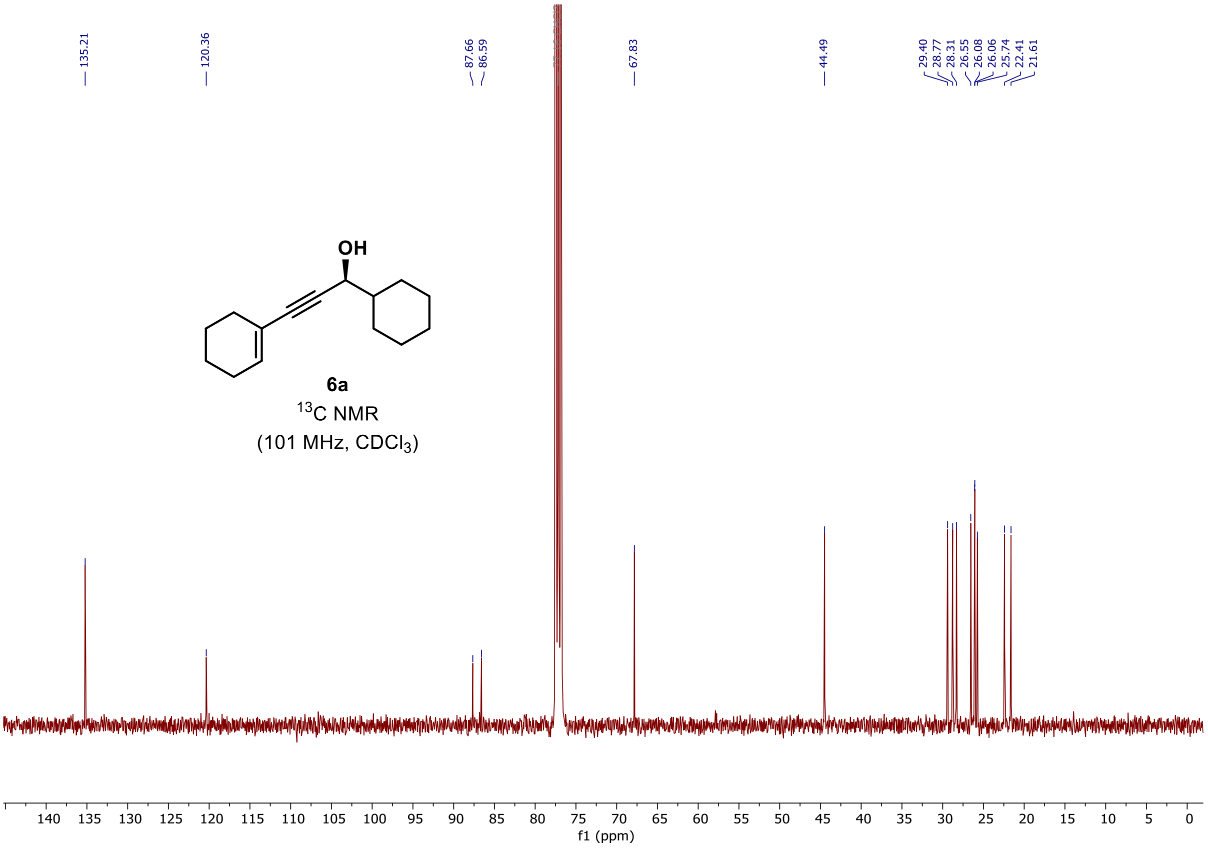
OH
6a
13C NMR
(101 MHz, CDCl3)
f1 (ppm)

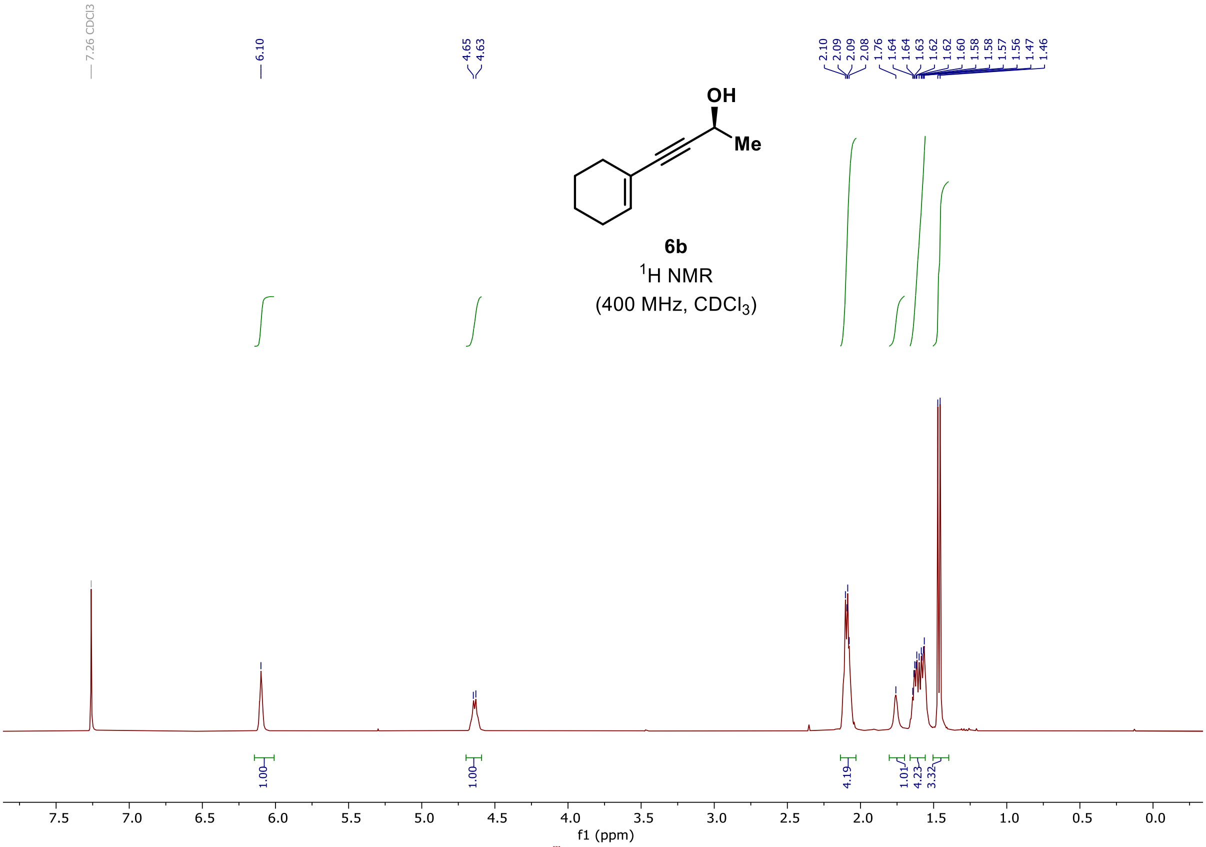
7.26 CDCl3
6.10
4.65
4.63
2.10 2.09 2.09 2.08 1.76 1.64 1.64 1.63 1.62 1.62 1.60 1.58 1.58 1.57 1.56 1.47 1.46
OH
Me
6b
^{1}H NMR
(400 MHz, $CDCl_3$)
1.00
1.00
4.19
1.01
4.23
3.32
f1 (ppm)

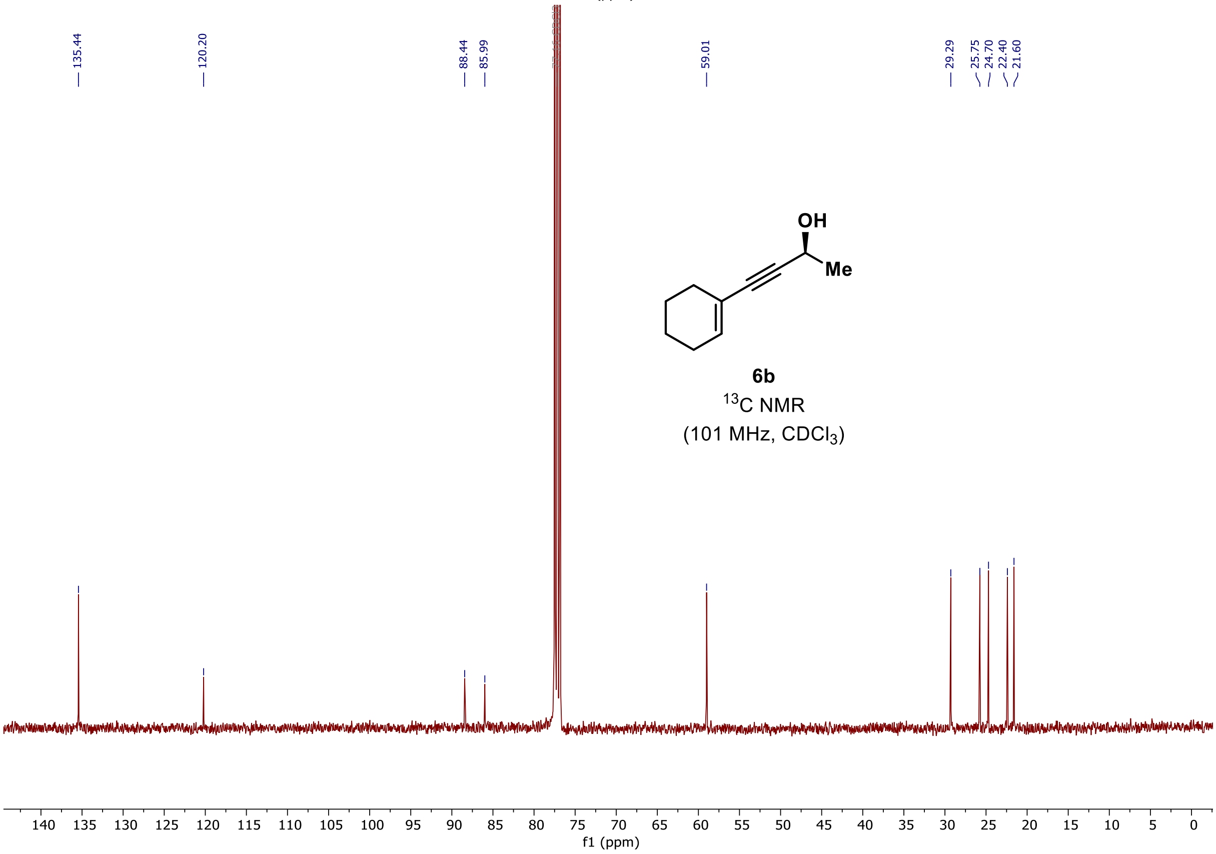
135.44
120.20
88.44
85.99
59.01
29.29
25.75
24.70
22.40
21.60
OH
Me
6b
^{13}C NMR
(101 MHz, $CDCl_3$)
f1 (ppm)

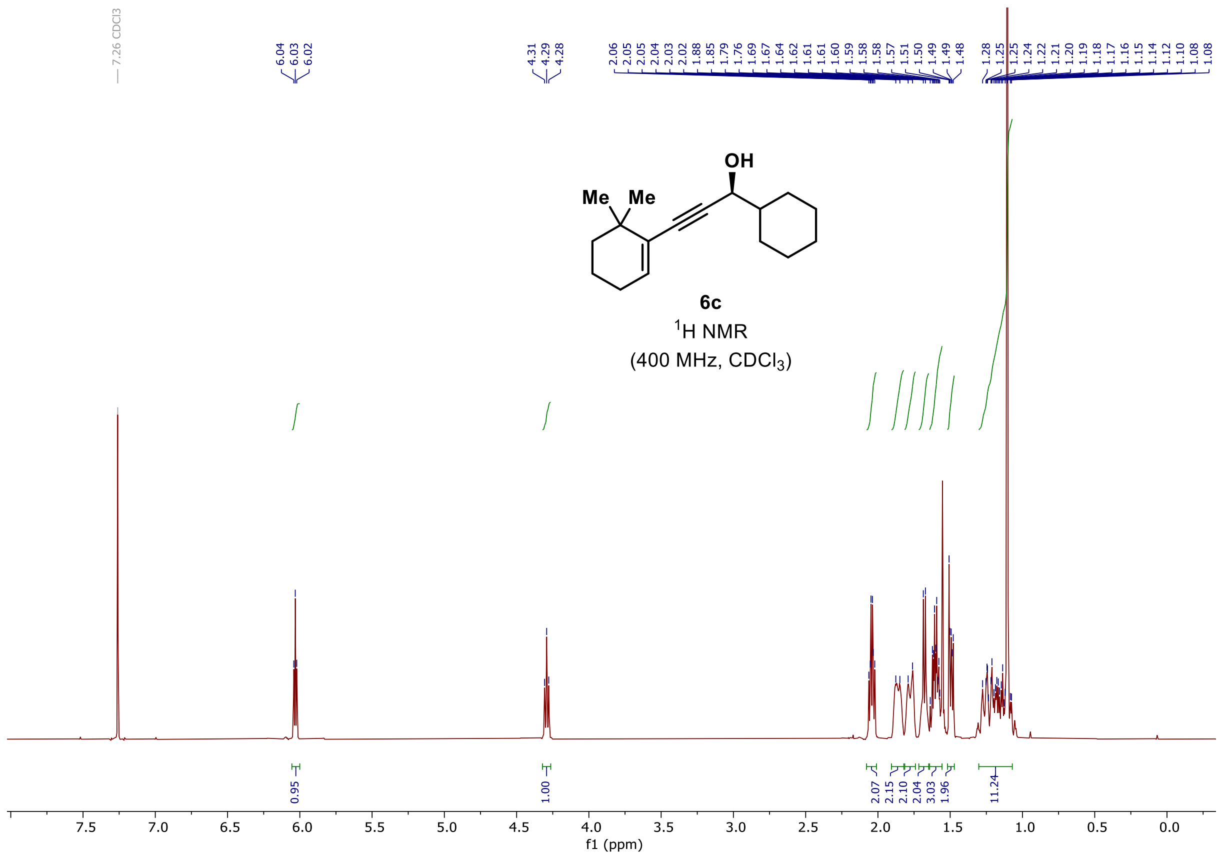
OH
Me Me
6c
1H NMR
(400 MHz, CDCl3)
f1 (ppm)

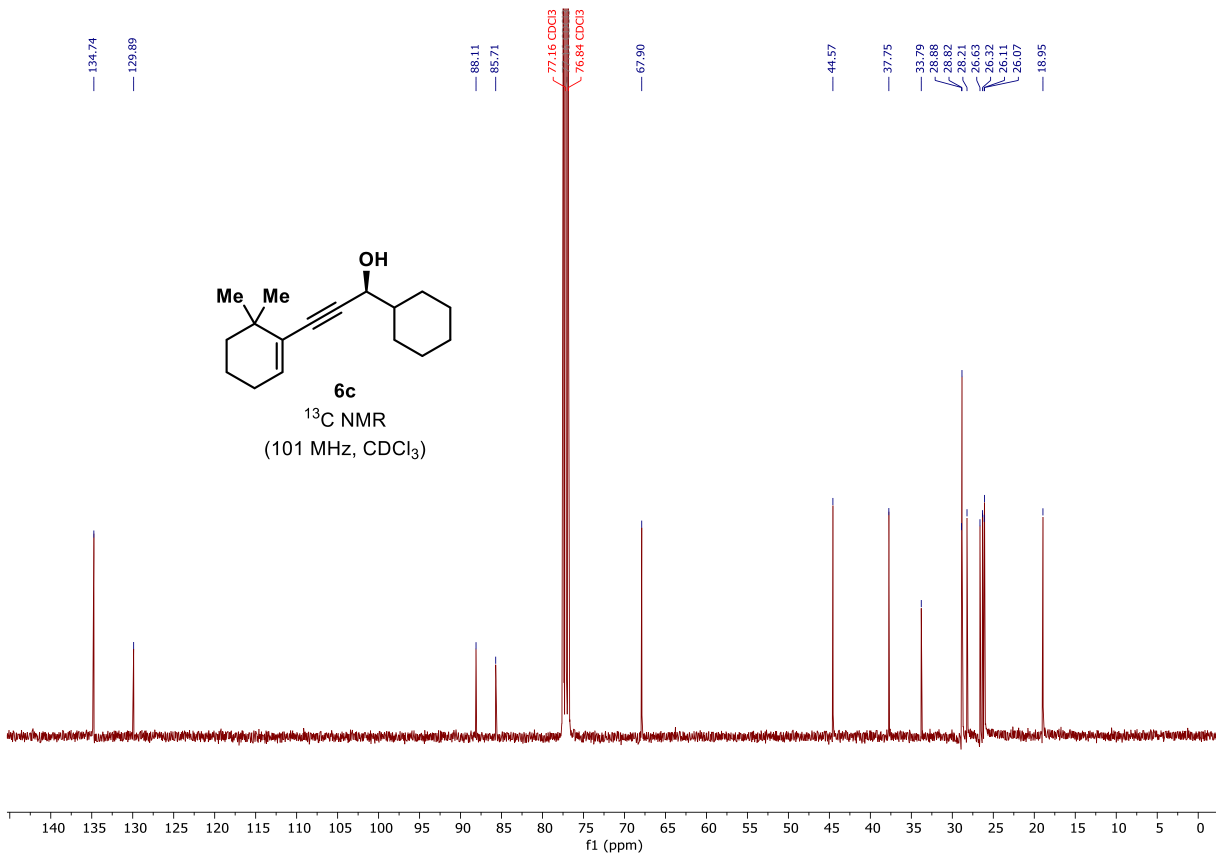
OH
Me Me
6c
13C NMR
(101 MHz, CDCl3)
f1 (ppm)

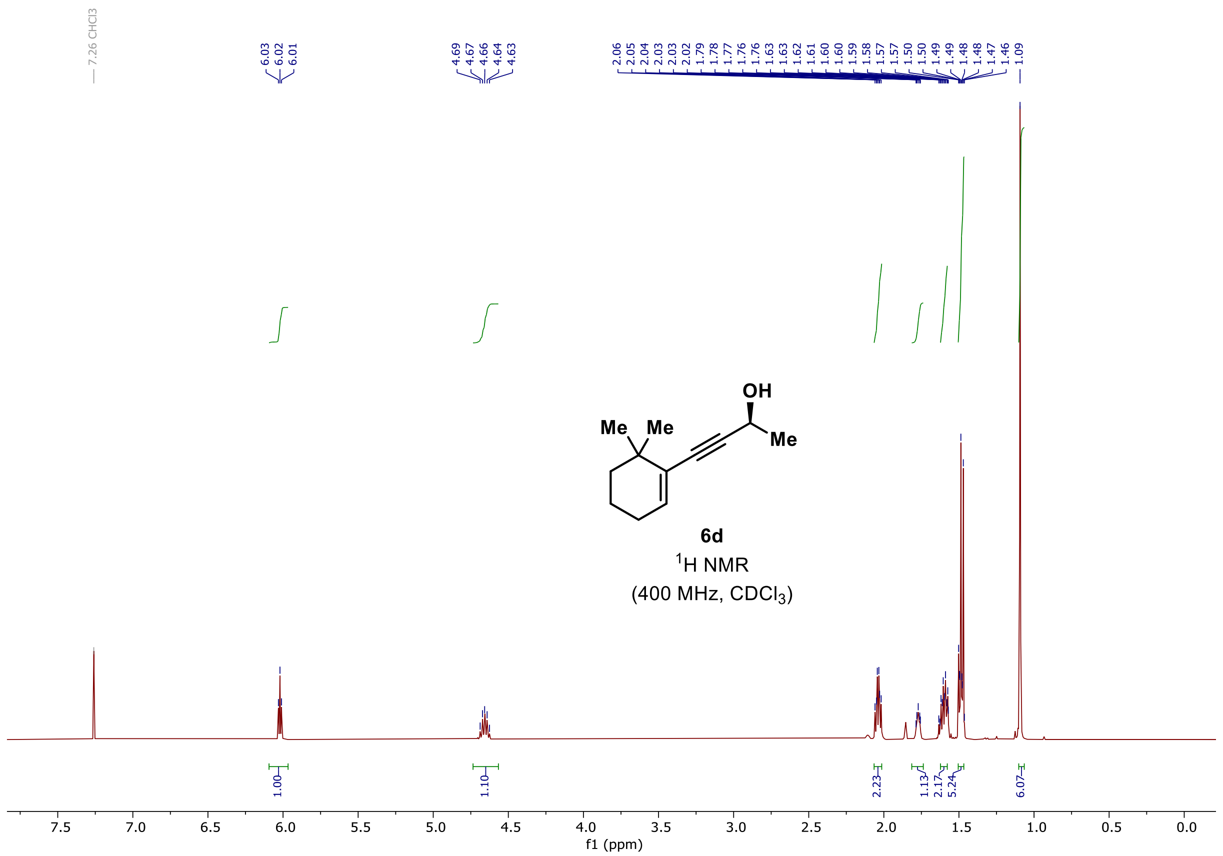
6d
^{1}H NMR
(400 MHz, CDCl$_3$)
f1 (ppm)

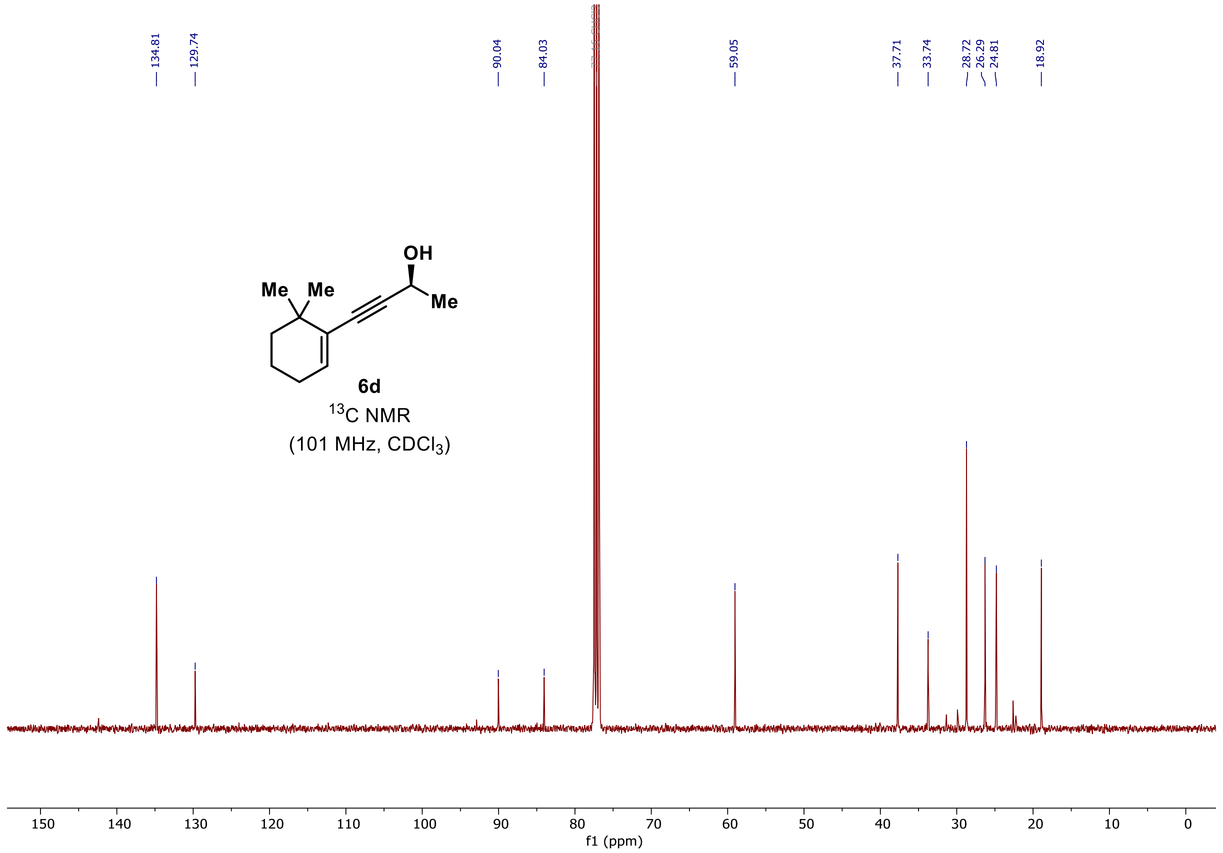
6d
^{13}C NMR
(101 MHz, CDCl$_3$)
f1 (ppm)

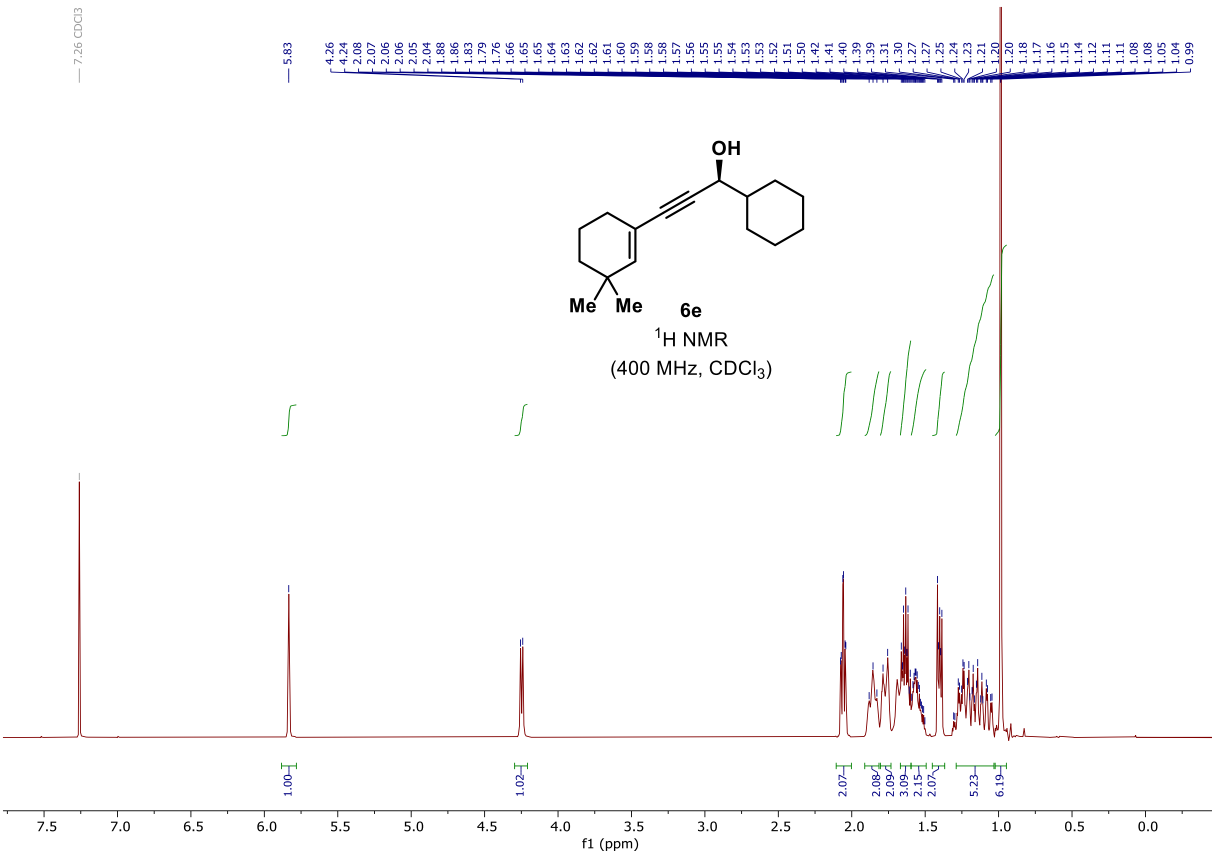

6e
^{1}H NMR
(400 MHz, $CDCl_3$)

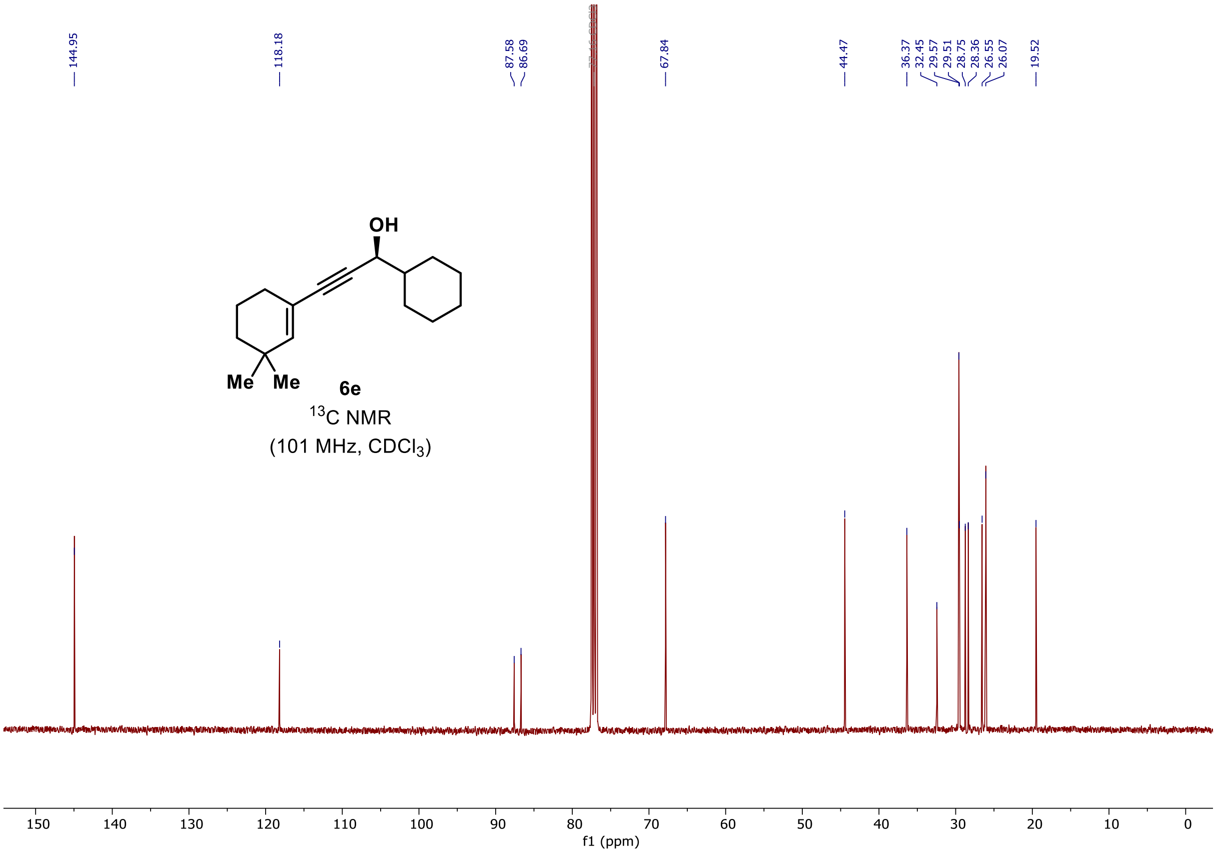

6e
^{13}C NMR
(101 MHz, $CDCl_3$)

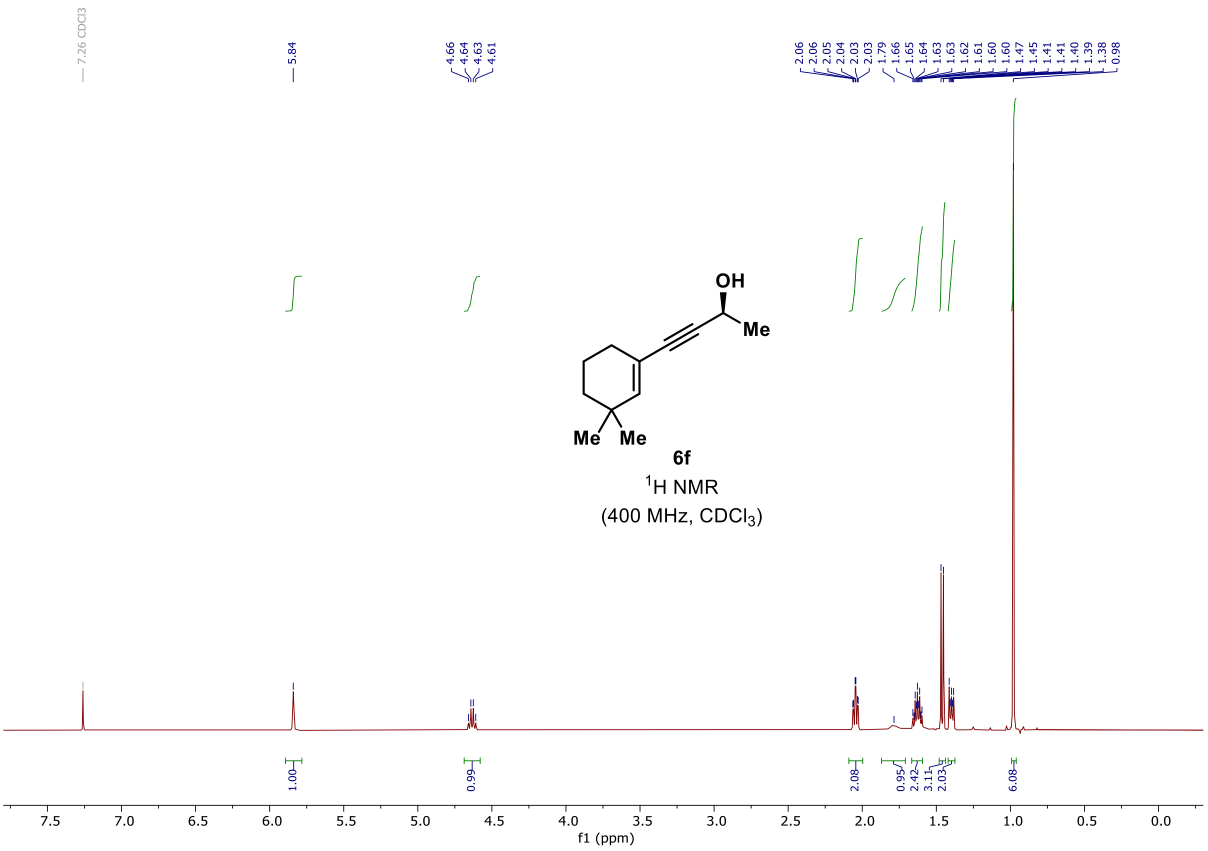

7.26 CDCl3
5.84
4.66 4.64 4.63 4.61
2.06 2.06 2.05 2.04 2.03 2.03 1.79 1.66 1.65 1.64 1.63 1.63 1.62 1.61 1.60 1.60 1.47 1.45 1.41 1.41 1.40 1.39 1.38 0.98
OH
Me
Me Me
6f
^{1}H NMR
(400 MHz, $CDCl_3$)
1.00
0.99
2.08
0.95
2.42
3.11
2.03
6.08
7.5 7.0 6.5 6.0 5.5 5.0 4.5 4.0 3.5 3.0 2.5 2.0 1.5 1.0 0.5 0.0
f1 (ppm)

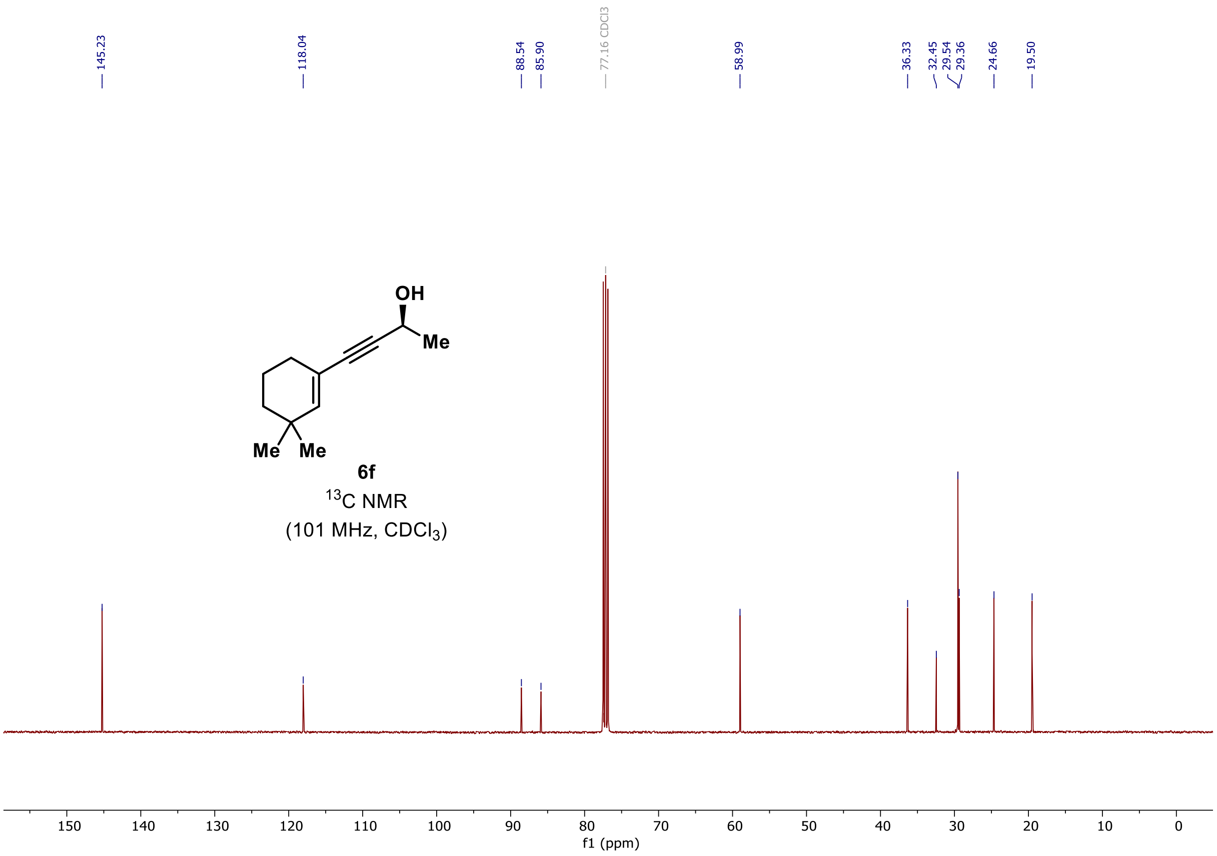

145.23
118.04
88.54
85.90
77.16 CDCl3
58.99
36.33
32.45
29.54
29.36
24.66
19.50
OH
Me
Me Me
6f
^{13}C NMR
(101 MHz, $CDCl_3$)
150 140 130 120 110 100 90 80 70 60 50 40 30 20 10 0
f1 (ppm)

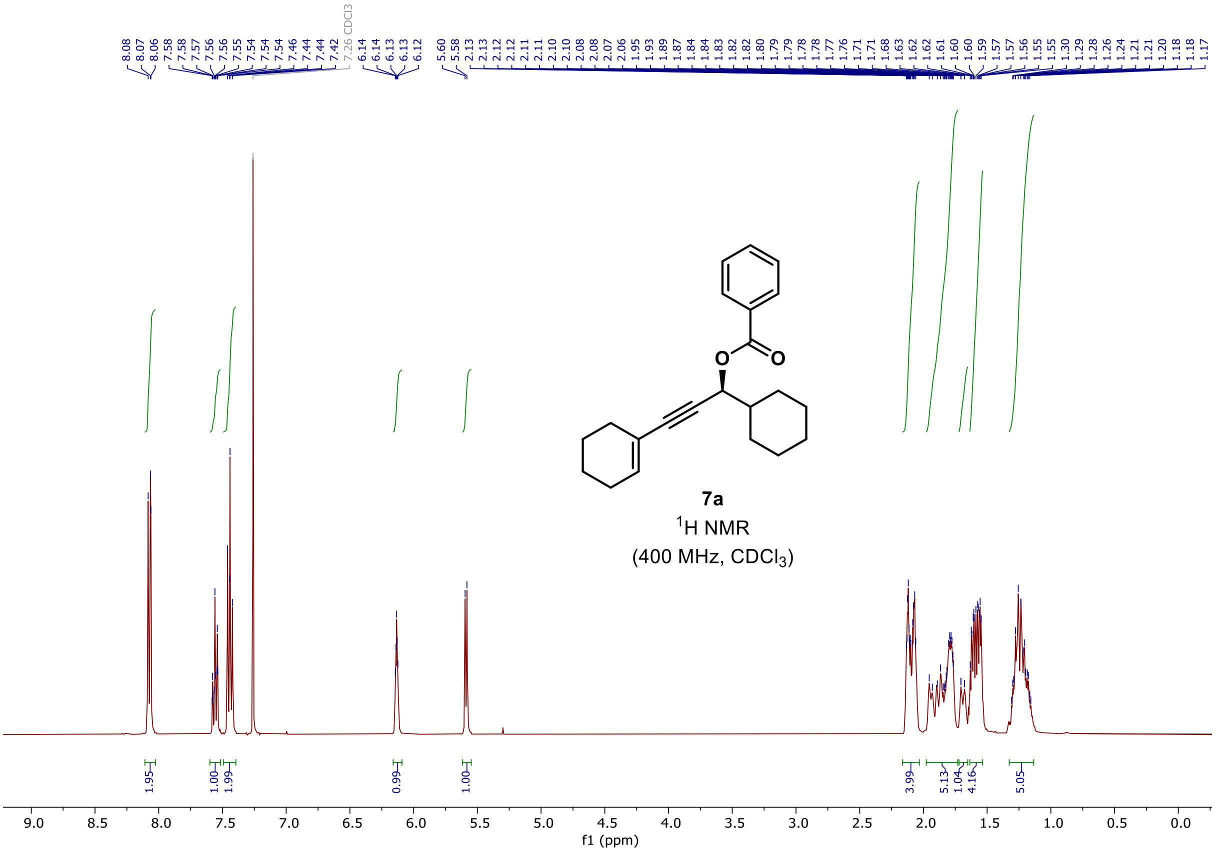
7a
1H NMR
(400 MHz, CDCl3)
f1 (ppm)

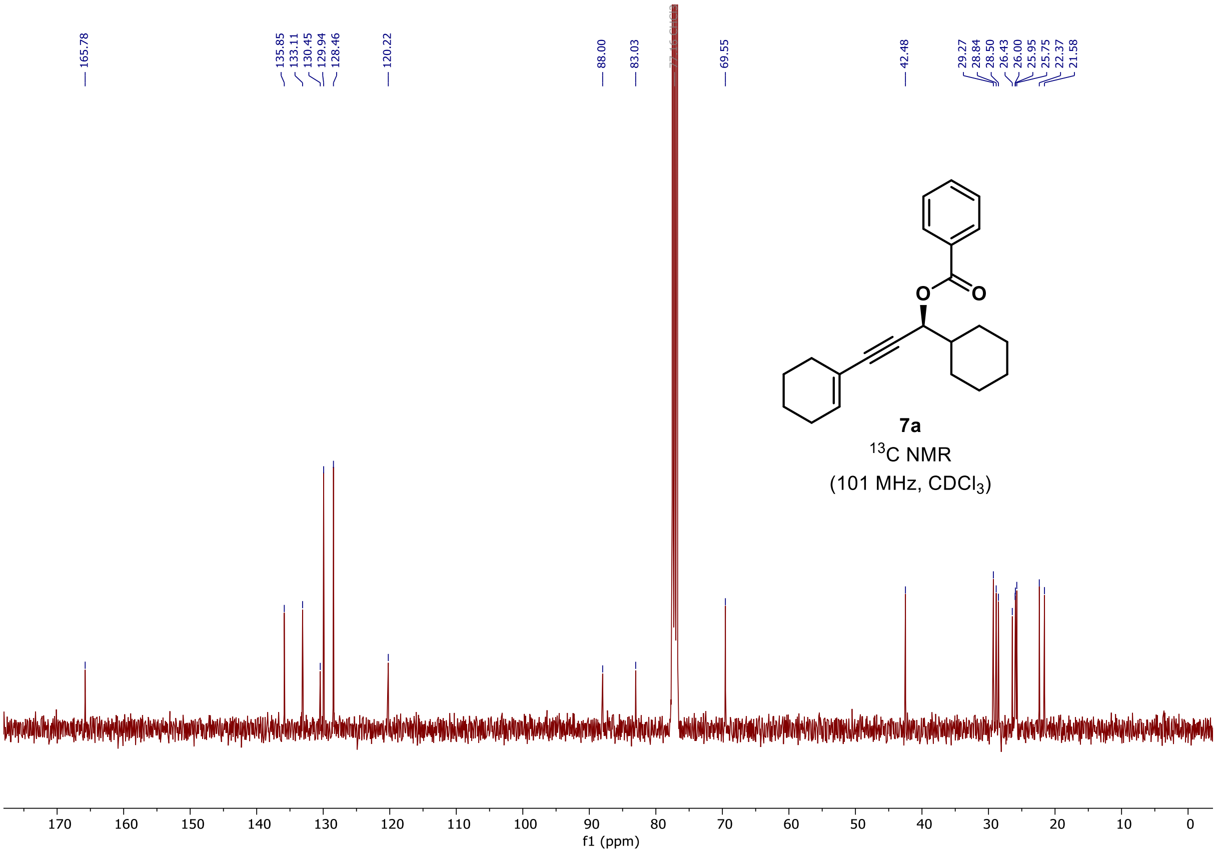
7a
13C NMR
(101 MHz, CDCl3)
f1 (ppm)

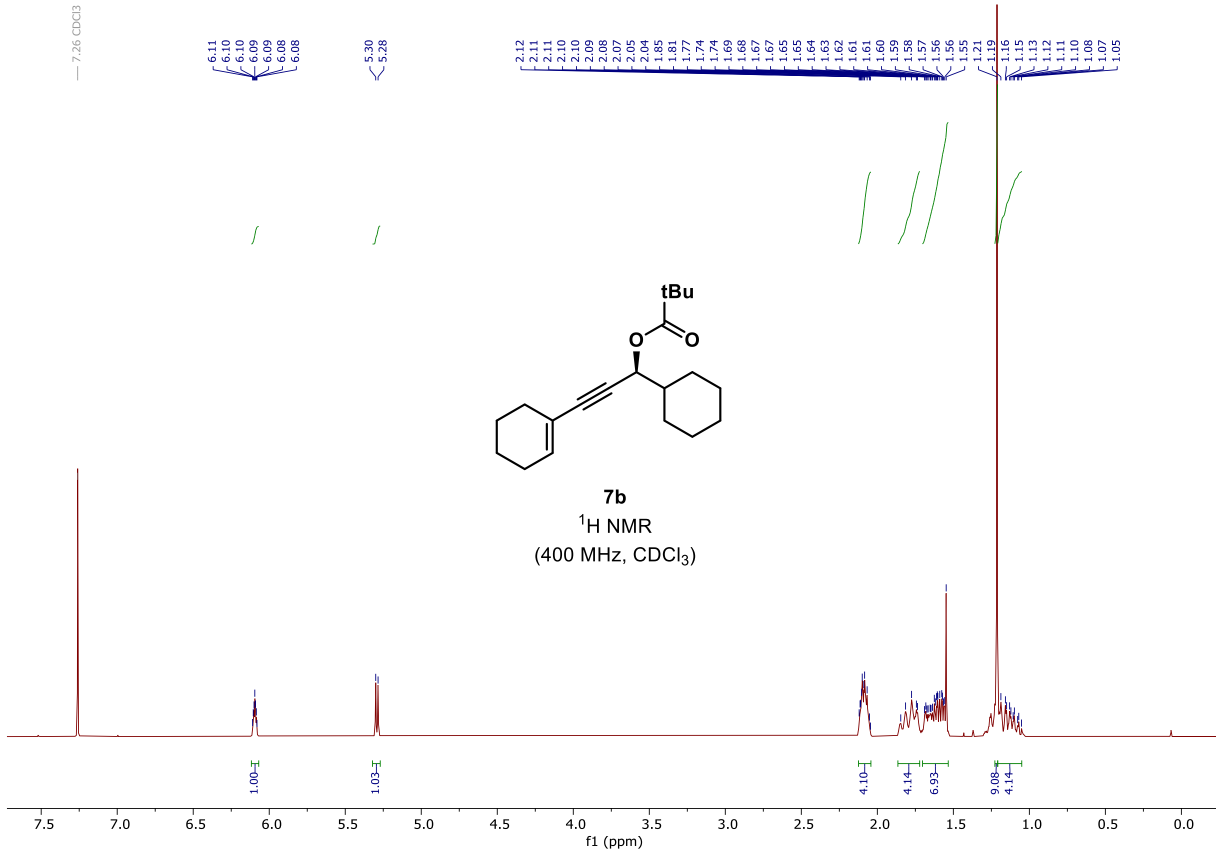
7b
^{1}H NMR
(400 MHz, $CDCl_3$)
tBu
O
O
f1 (ppm)

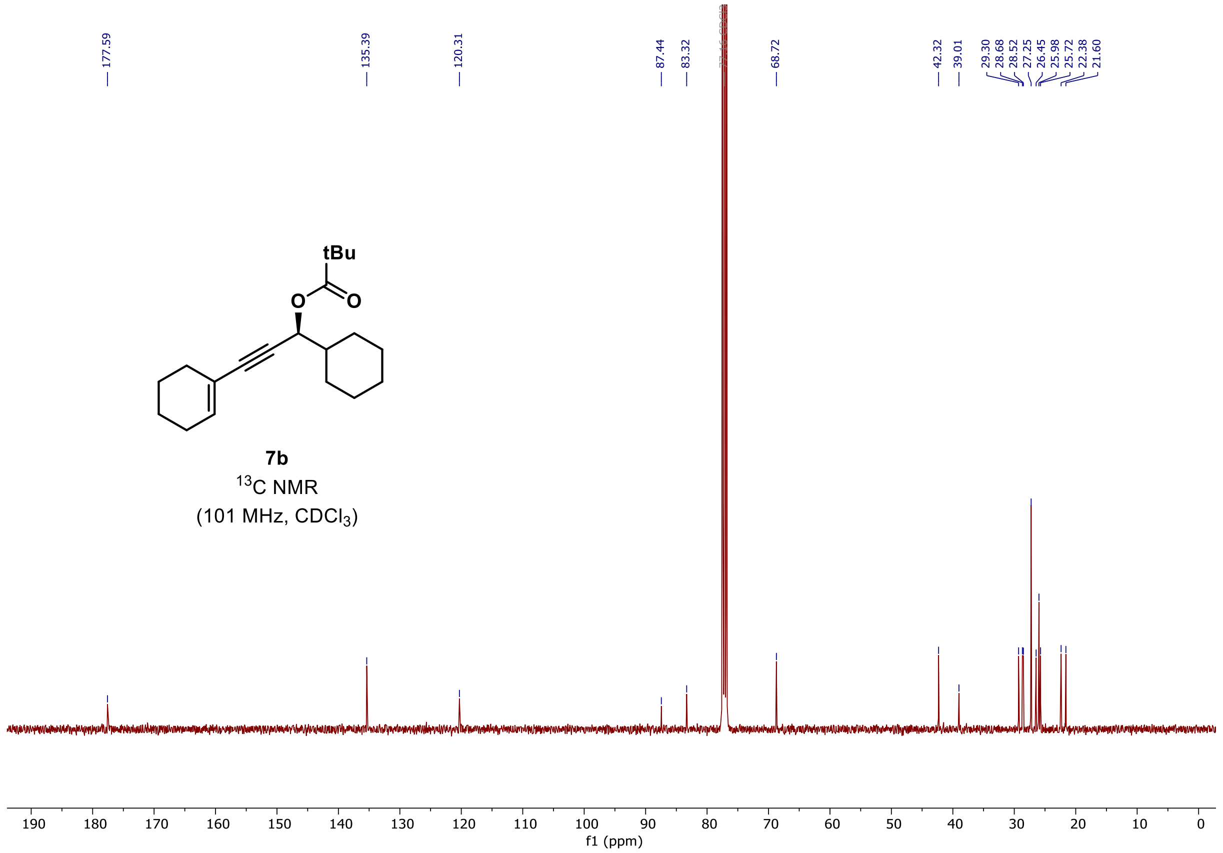
7b
^{13}C NMR
(101 MHz, $CDCl_3$)
tBu
O
O
f1 (ppm)

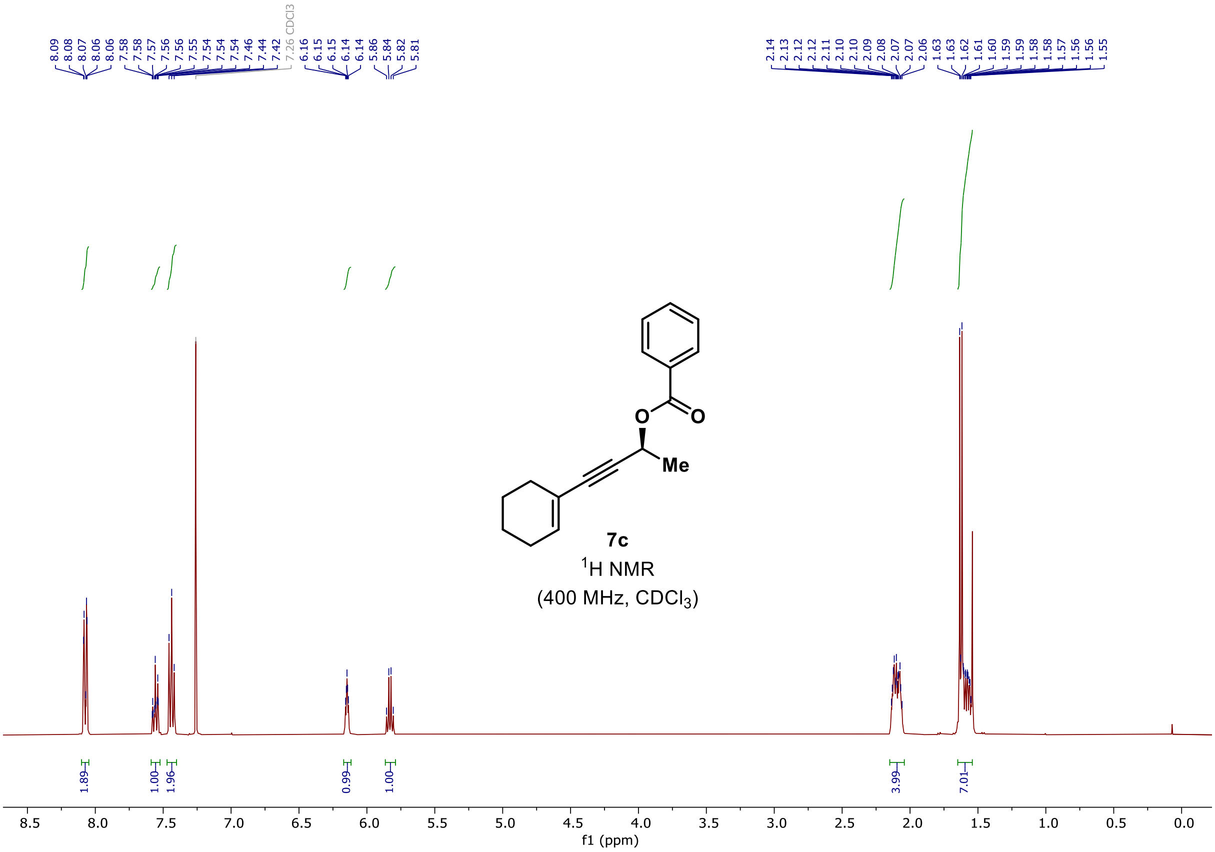
7c
^{1}H NMR
(400 MHz, CDCl$_3$)
f1 (ppm)

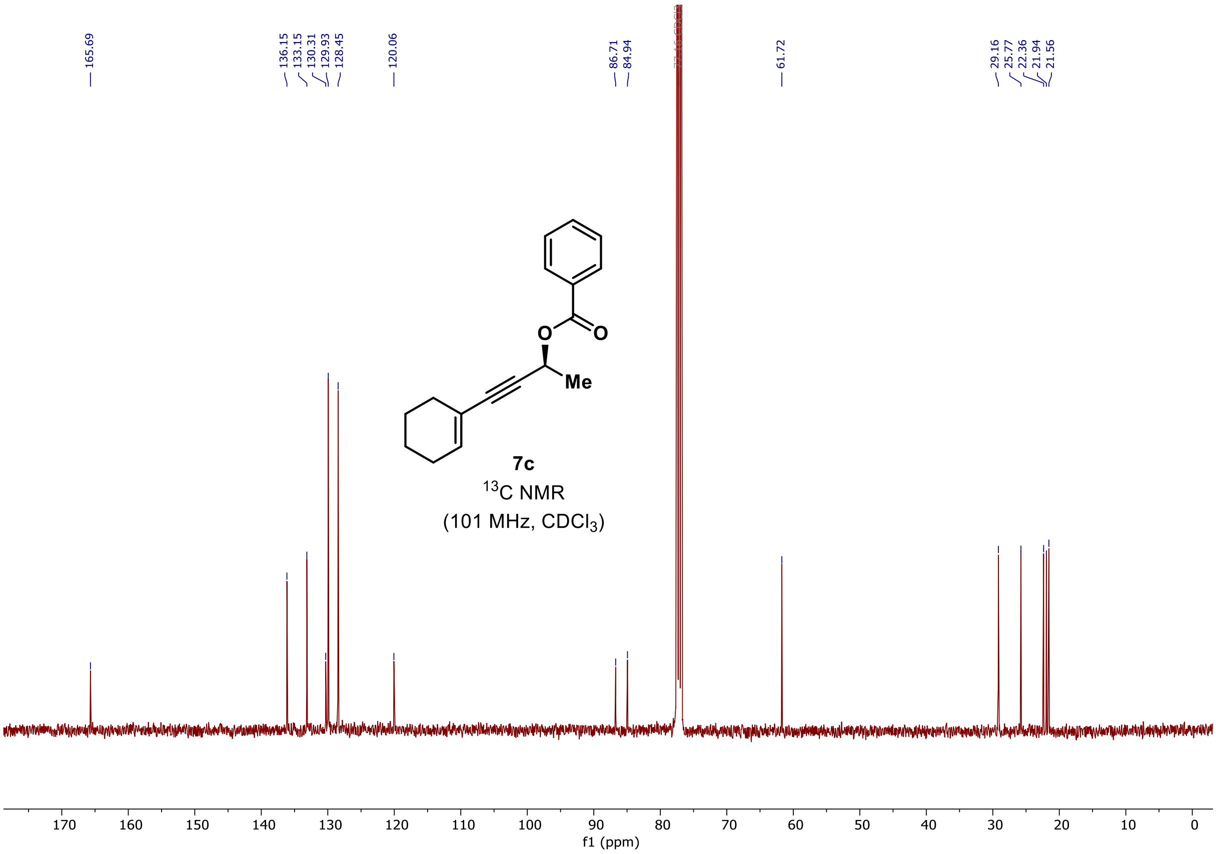
7c
^{13}C NMR
(101 MHz, CDCl$_3$)
f1 (ppm)

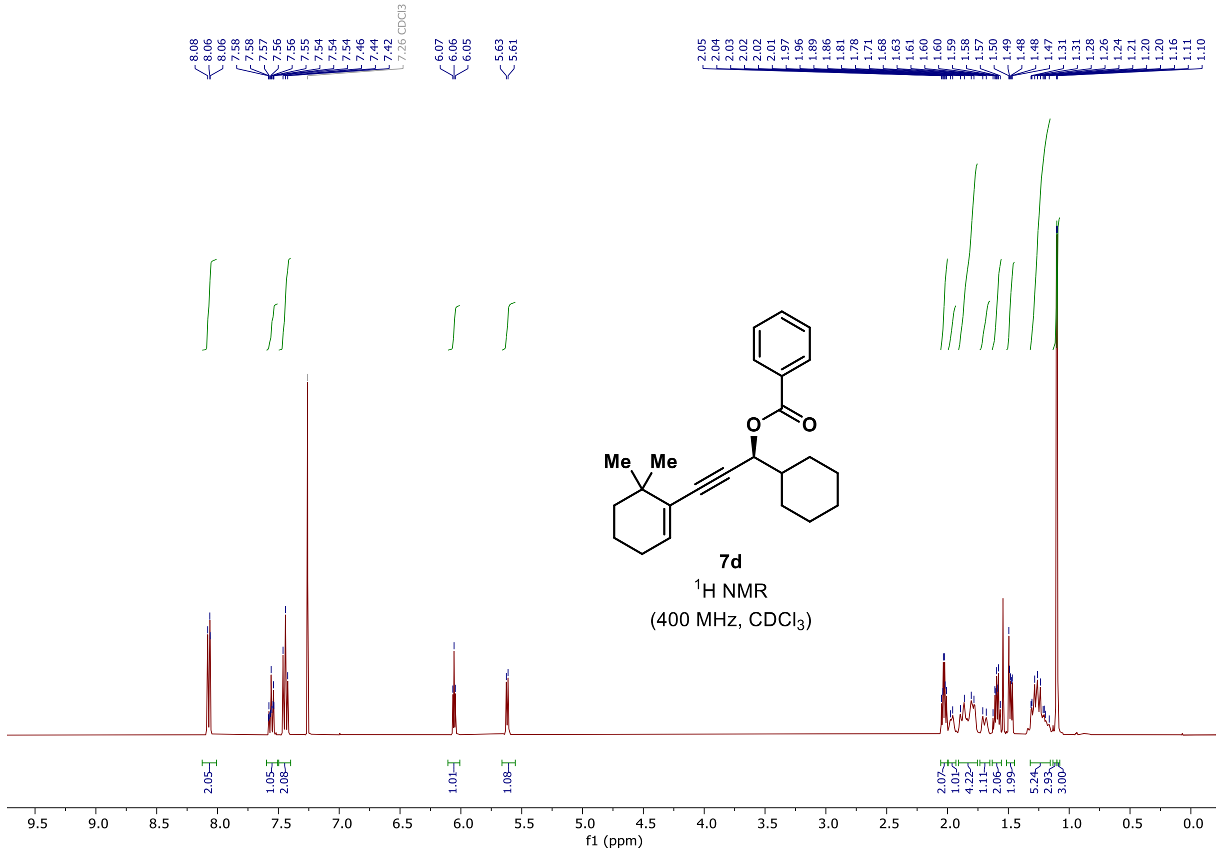
8.08 8.06 8.06 7.58 7.57 7.56 7.56 7.55 7.54 7.54 7.54 7.46 7.44 7.42 7.26 CDCl3
6.07 6.06 6.05
5.63 5.61
2.05 2.04 2.03 2.02 2.02 2.01 1.97 1.96 1.89 1.86 1.81 1.78 1.71 1.68 1.63 1.61 1.61 1.60 1.60 1.59 1.58 1.57 1.50 1.49 1.48 1.47 1.31 1.28 1.26 1.24 1.21 1.20 1.20 1.16 1.11 1.10
Me
Me
O
O
7d
^{1}H NMR
(400 MHz, $CDCl_3$)
2.05
1.05
2.08
1.01
1.08
2.07
1.01
4.22
1.11
2.06
1.99
5.24
2.93
3.00
9.5 9.0 8.5 8.0 7.5 7.0 6.5 6.0 5.5 5.0 4.5 4.0 3.5 3.0 2.5 2.0 1.5 1.0 0.5 0.0
f1 (ppm)

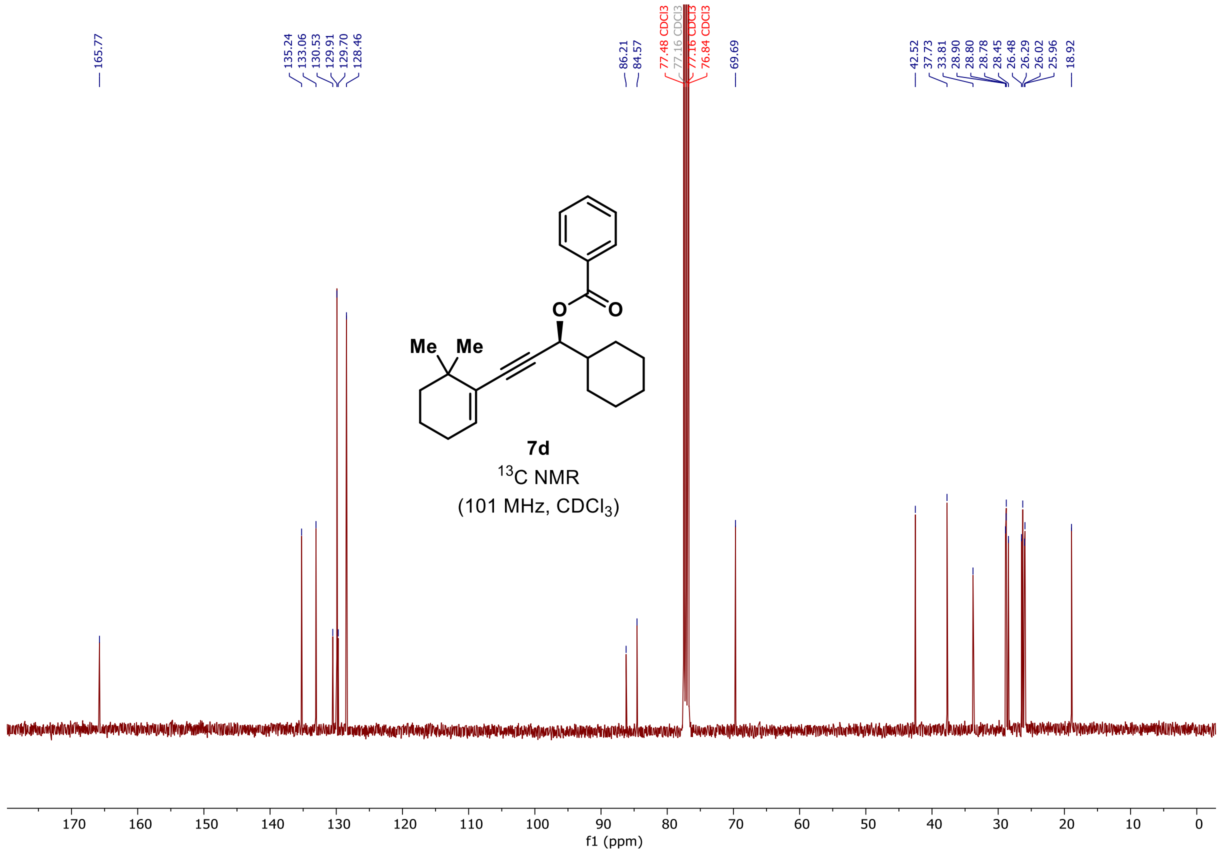
165.77
135.24 133.06 130.53 129.91 129.70 128.46
86.21 84.57
77.48 CDCl3 77.16 CDCl3 77.16 CDCl3 76.84 CDCl3
69.69
42.52 37.73 33.81 28.90 28.80 28.78 28.45 26.48 26.29 26.02 25.96 18.92
Me
Me
O
O
7d
^{13}C NMR
(101 MHz, $CDCl_3$)
170 160 150 140 130 120 110 100 90 80 70 60 50 40 30 20 10 0
f1 (ppm)

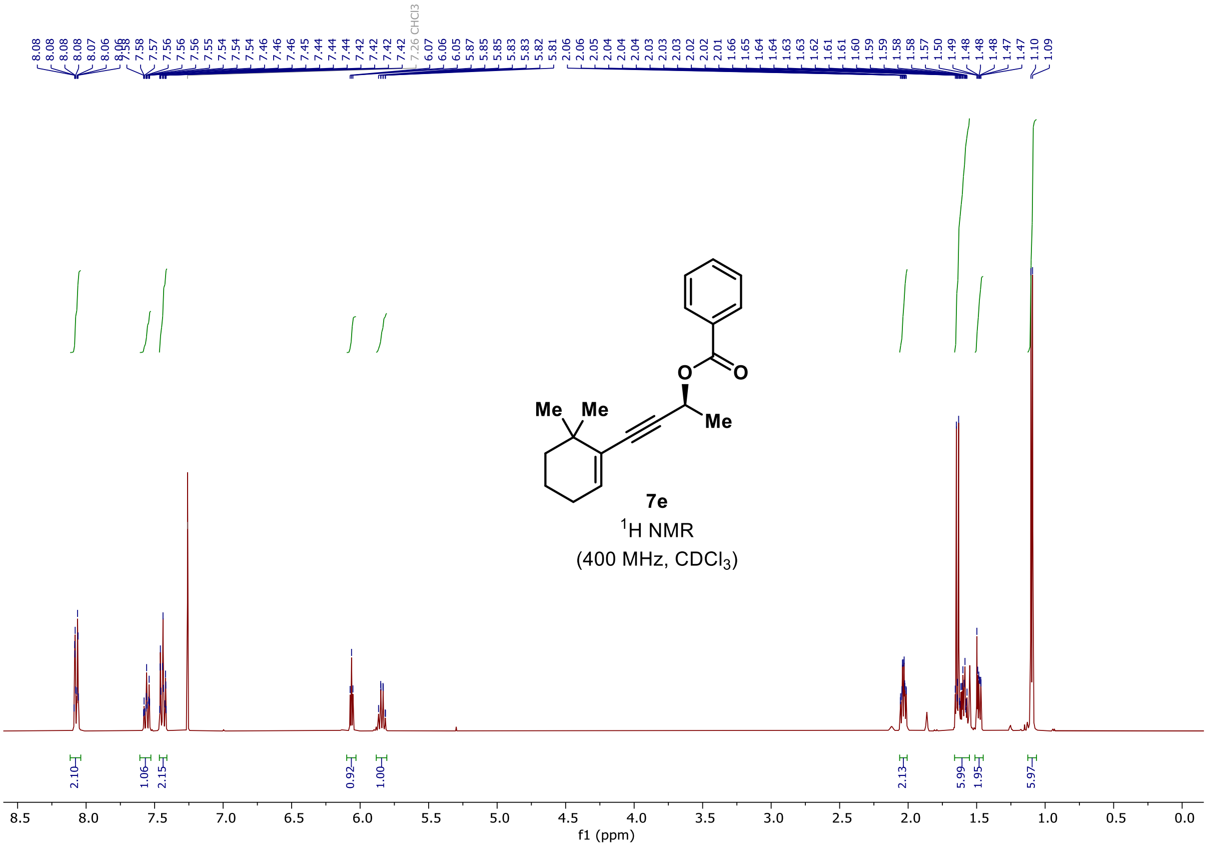
7e
^{1}H NMR
(400 MHz, CDCl$_3$)
f1 (ppm)

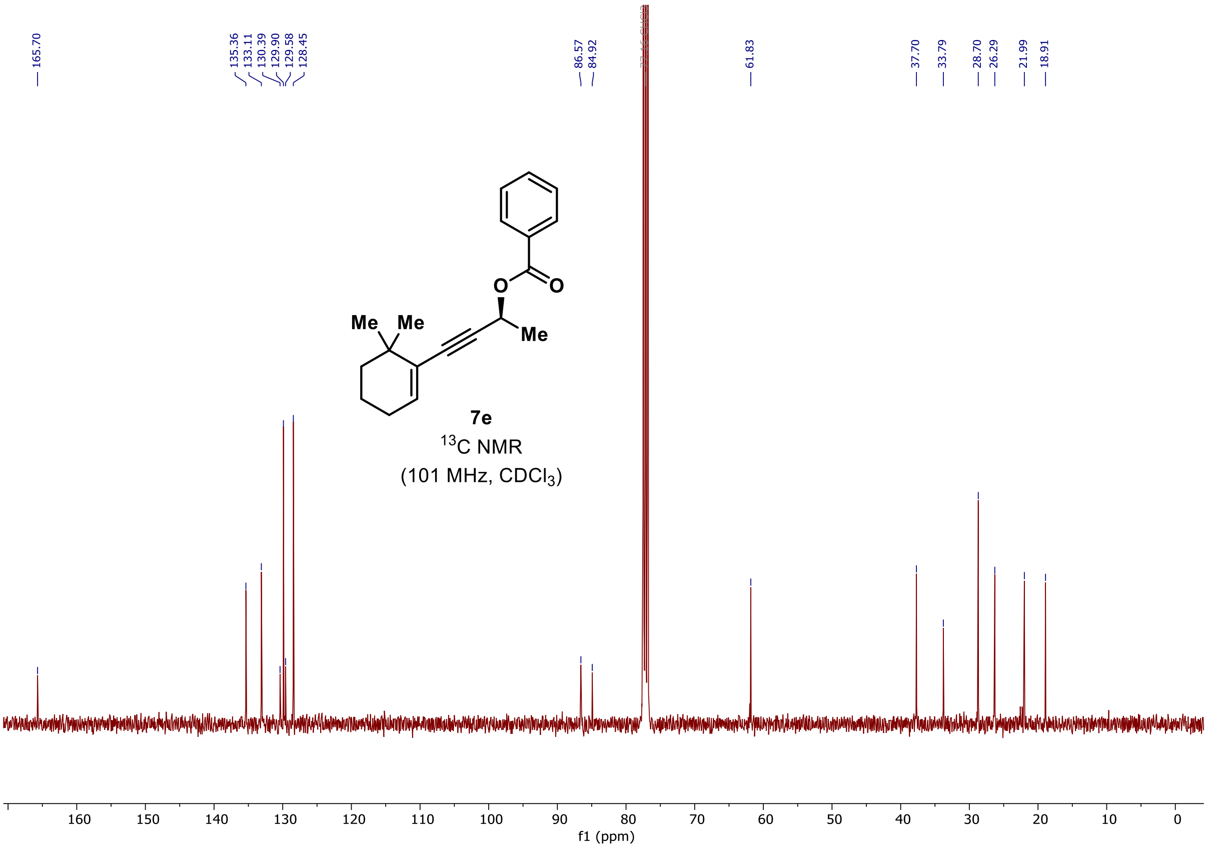
7e
^{13}C NMR
(101 MHz, CDCl$_3$)
f1 (ppm)

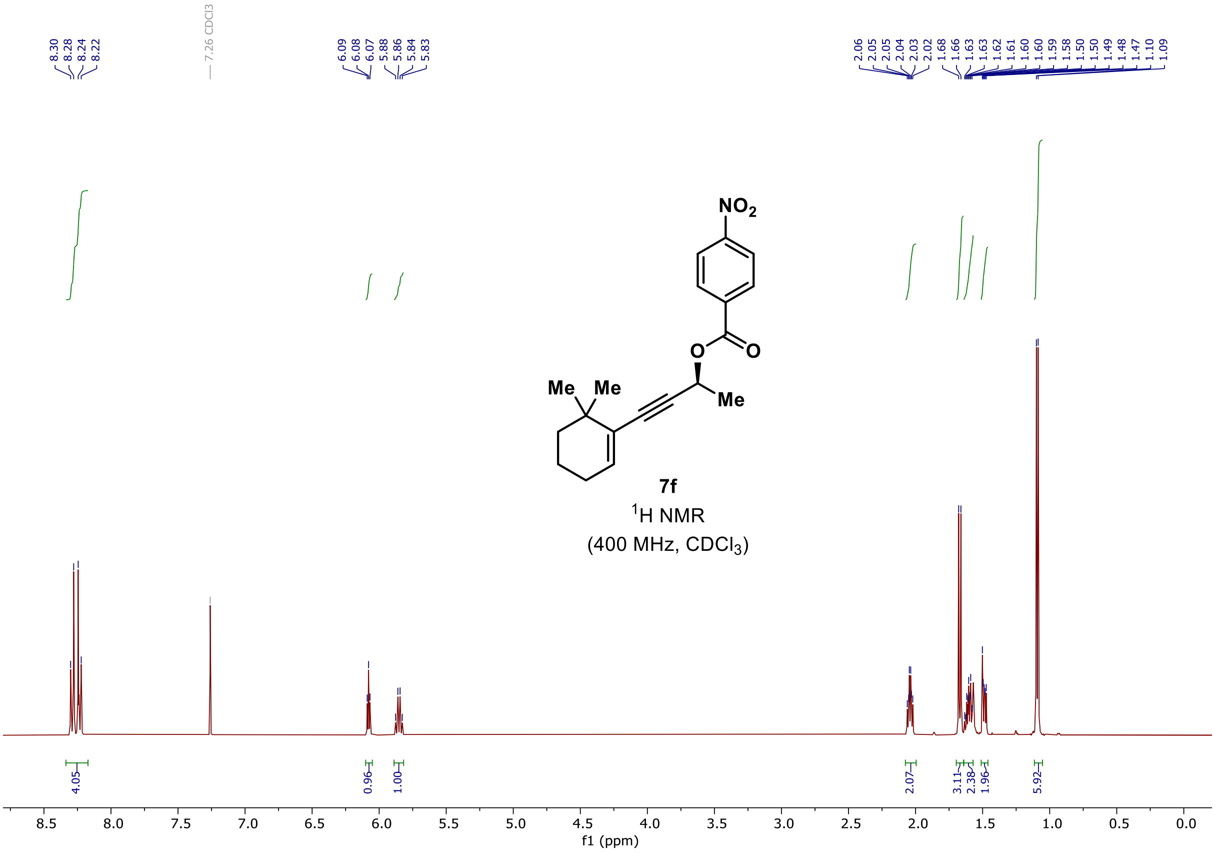
8.30 8.28 8.24 8.22
7.26 CDCl3
6.09 6.08 6.07 5.88 5.86 5.84 5.83
2.06 2.05 2.05 2.04 2.03 2.02 1.68 1.66 1.63 1.63 1.62 1.61 1.60 1.60 1.59 1.58 1.50 1.50 1.49 1.48 1.47 1.10 1.09
NO2
O
O
Me Me
Me
7f
1H NMR
(400 MHz, CDCl3)
4.05 0.96 1.00 2.07 3.11 2.38 1.96 5.92
8.5 8.0 7.5 7.0 6.5 6.0 5.5 5.0 4.5 4.0 3.5 3.0 2.5 2.0 1.5 1.0 0.5 0.0
f1 (ppm)

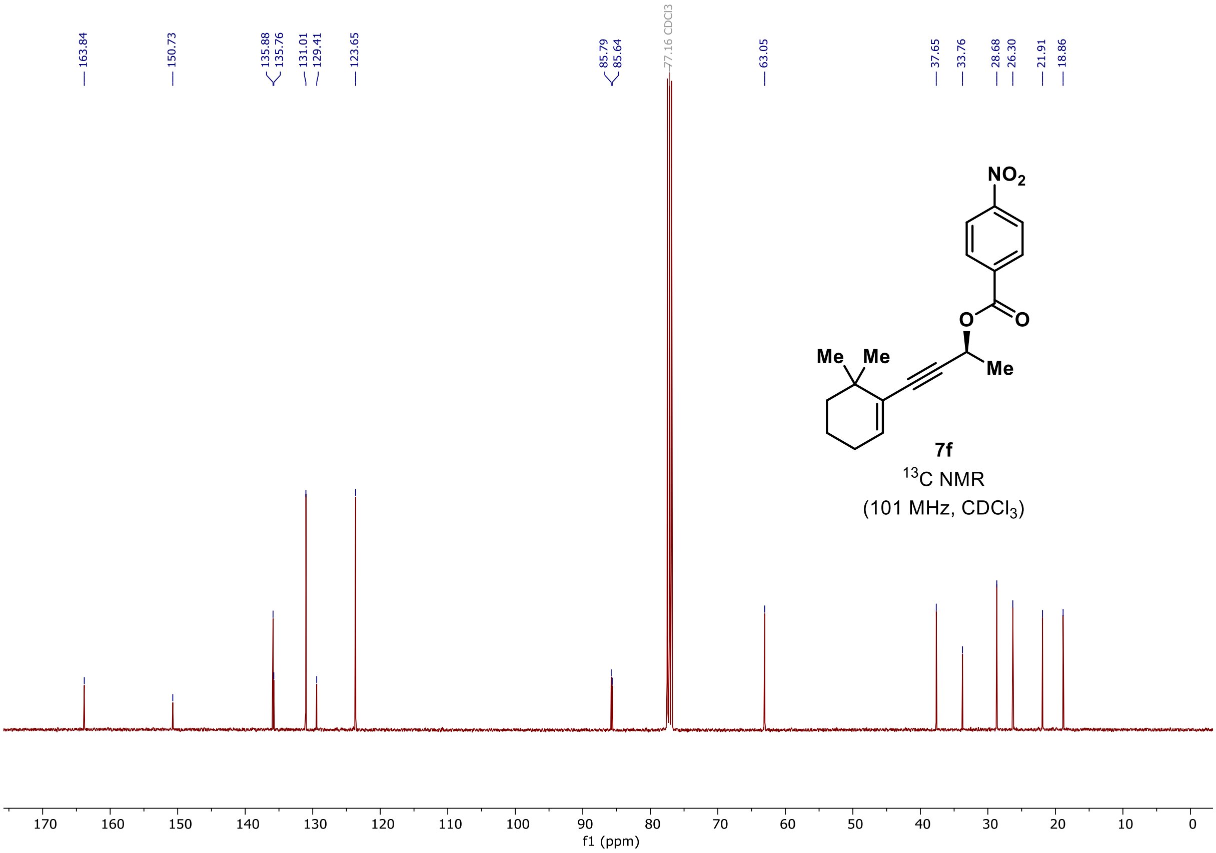
163.84
150.73
135.88 135.76 131.01 129.41
123.65
85.79 85.64
77.16 CDCl3
63.05
37.65 33.76 28.68 26.30 21.91 18.86
NO2
O
O
Me Me
Me
7f
13C NMR
(101 MHz, CDCl3)
170 160 150 140 130 120 110 100 90 80 70 60 50 40 30 20 10 0
f1 (ppm)

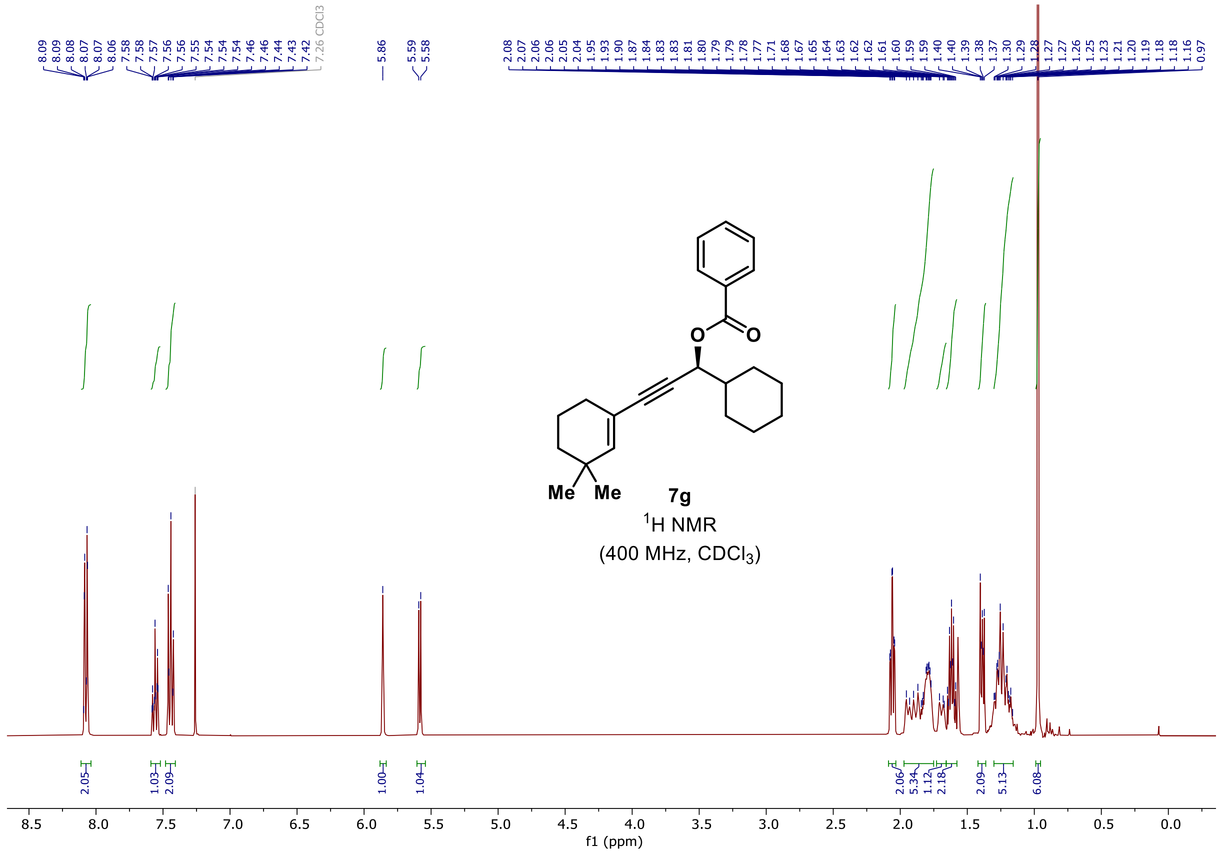
7g
^{1}H NMR
(400 MHz, $CDCl_3$)
f1 (ppm)

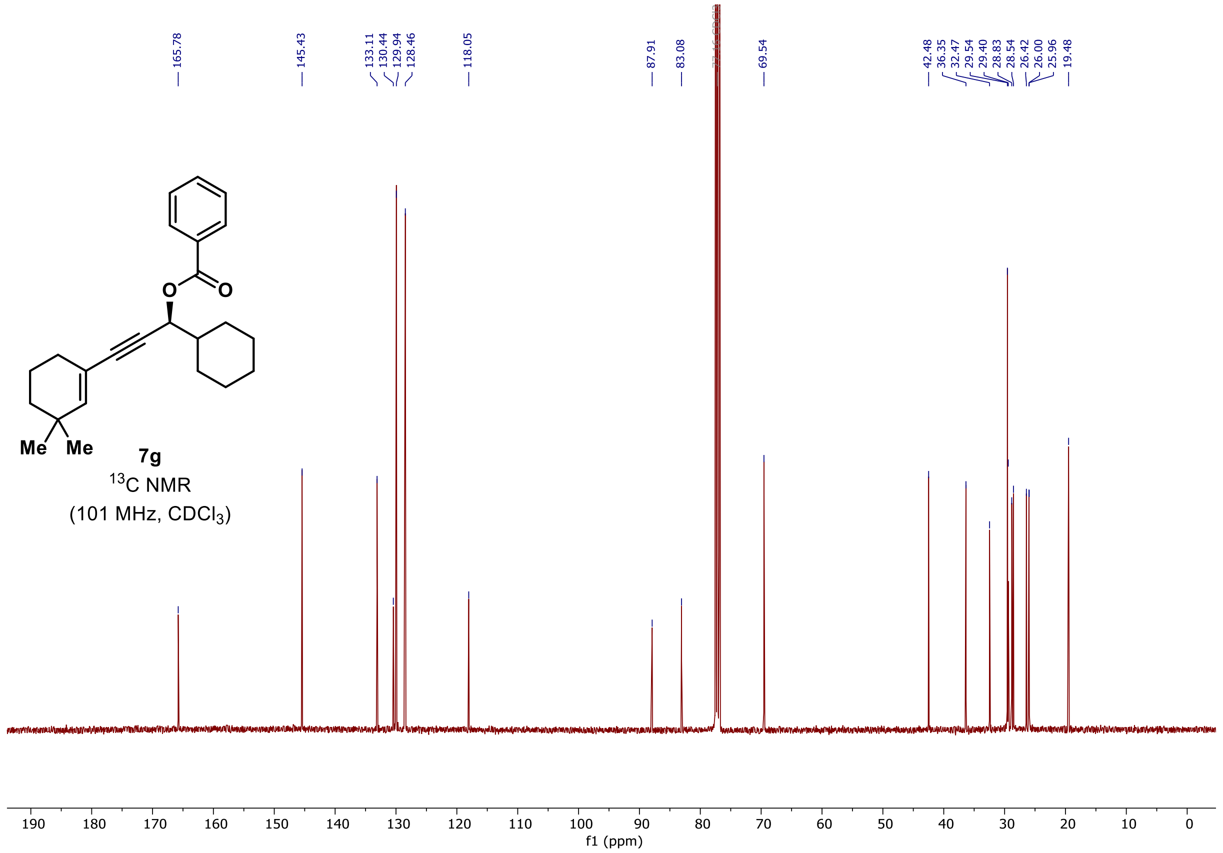
7g
^{13}C NMR
(101 MHz, $CDCl_3$)
f1 (ppm)

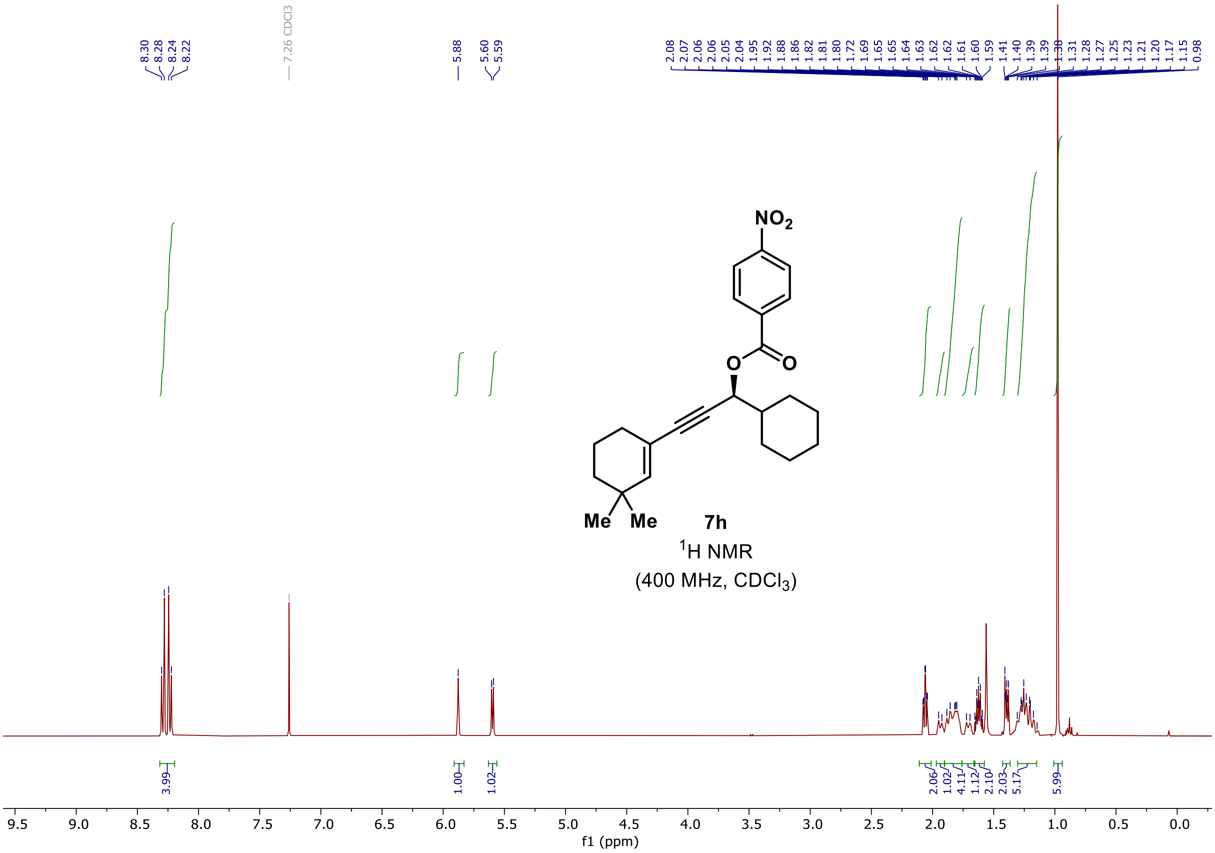
8.30
8.28
8.24
8.22
7.26 CDCl3
5.88
5.60
5.59
2.08 2.07 2.06 2.06 2.05 2.04 1.95 1.92 1.88 1.86 1.82 1.81 1.80 1.72 1.69 1.65 1.65 1.64 1.63 1.62 1.62 1.61 1.60 1.59 1.41 1.40 1.39 1.39 1.38 1.31 1.28 1.27 1.25 1.23 1.21 1.20 1.17 1.15 0.98
NO2
O
O
Me
Me
7h
1H NMR
(400 MHz, CDCl3)
3.99
1.00
1.02
2.06
1.02
4.11
1.12
2.10
2.03
5.17
5.99
9.5 9.0 8.5 8.0 7.5 7.0 6.5 6.0 5.5 5.0 4.5 4.0 3.5 3.0 2.5 2.0 1.5 1.0 0.5 0.0
f1 (ppm)

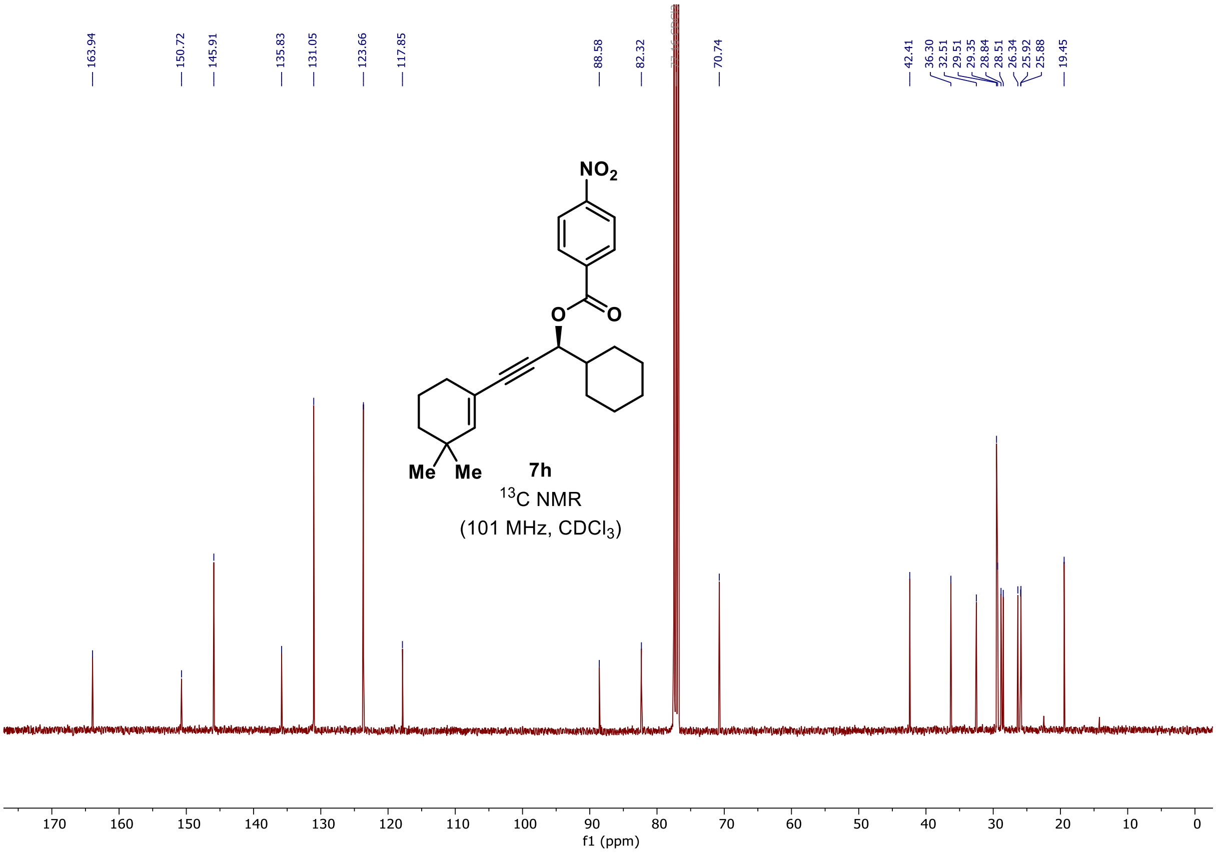
163.94
150.72
145.91
135.83
131.05
123.66
117.85
88.58
82.32
70.74
42.41 36.30 32.51 29.51 29.35 28.84 28.51 26.34 25.92 25.88 19.45
NO2
O
O
Me
Me
7h
13C NMR
(101 MHz, CDCl3)
170 160 150 140 130 120 110 100 90 80 70 60 50 40 30 20 10 0
f1 (ppm)

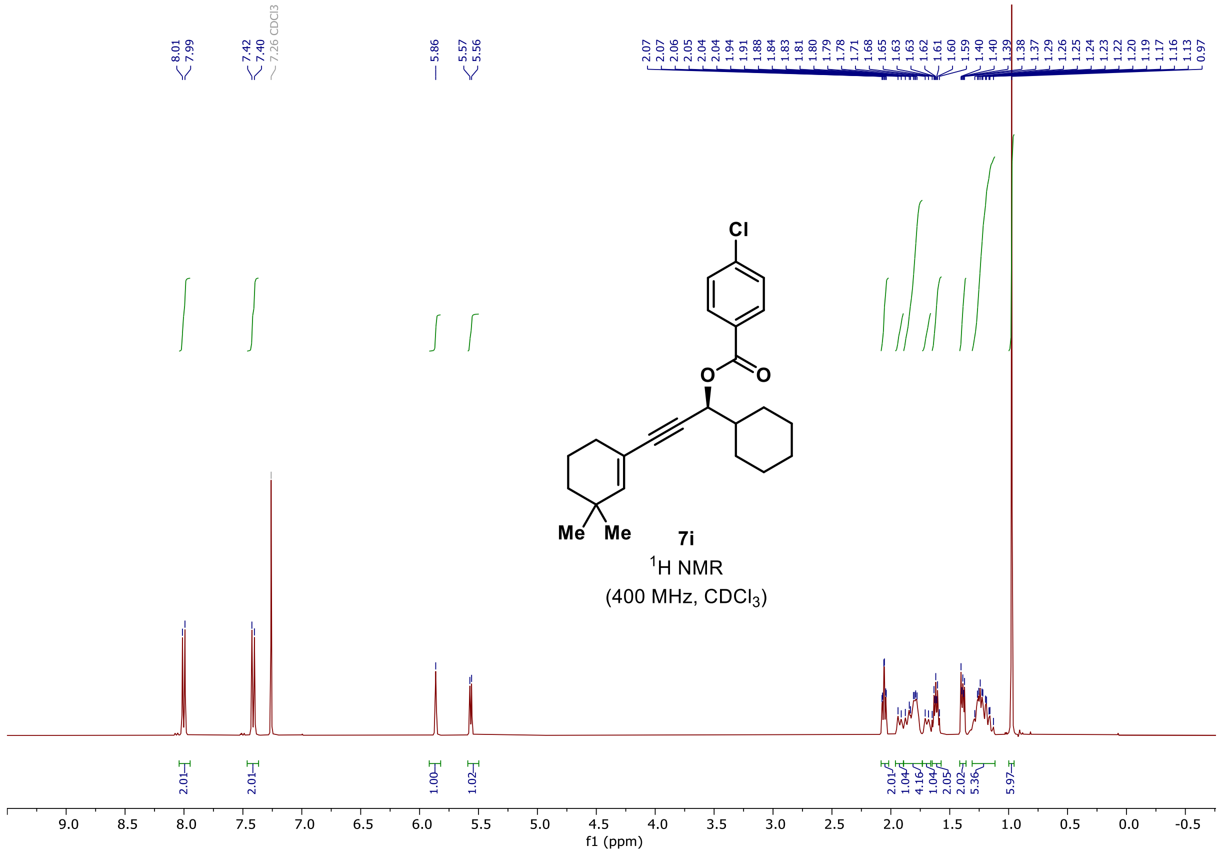
8.01
7.99
7.42
7.40
7.26 CDCl3
5.86
5.57
5.56
Cl
O
O
Me
Me
7i
^{1}H NMR
(400 MHz, CDCl$_3$)
2.01
2.01
1.00
1.02
2.01
1.04
4.16
1.04
2.05
2.02
5.36
5.97
f1 (ppm)

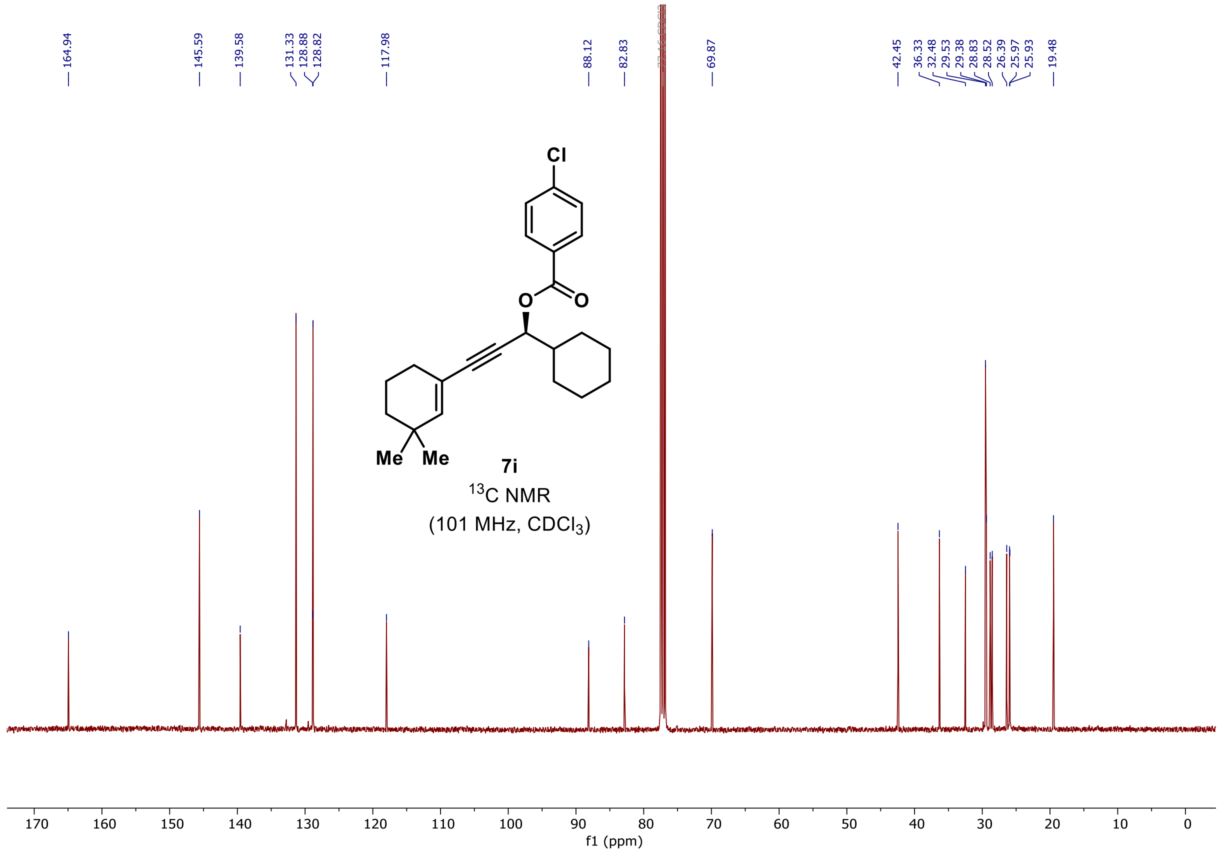
164.94
145.59
139.58
131.33
128.88
128.82
117.98
88.12
82.83
69.87
42.45
36.33
32.48
29.53
29.38
28.83
28.52
26.39
25.97
25.93
19.48
Cl
O
O
Me
Me
7i
^{13}C NMR
(101 MHz, CDCl$_3$)
f1 (ppm)

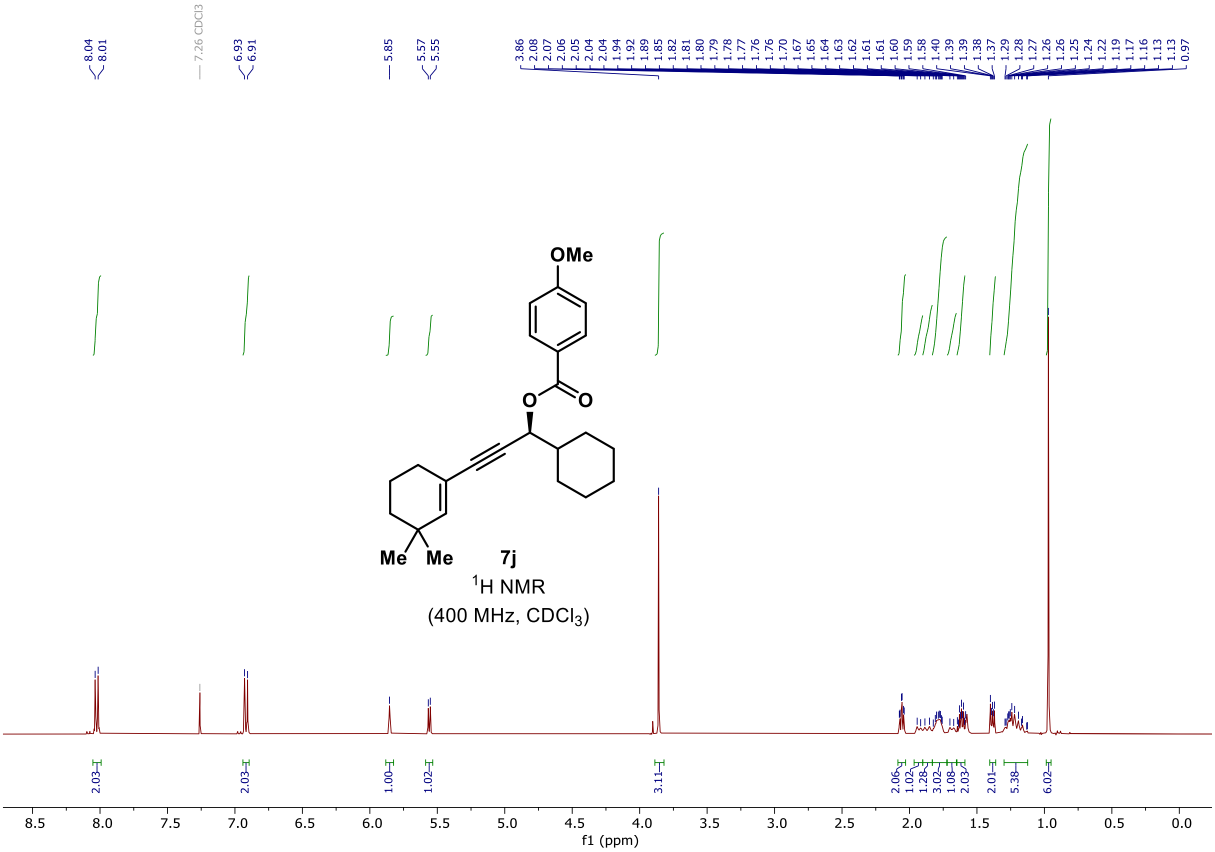
OMe
Me
Me
7j
^{1}H NMR
(400 MHz, CDCl$_3$)
f1 (ppm)

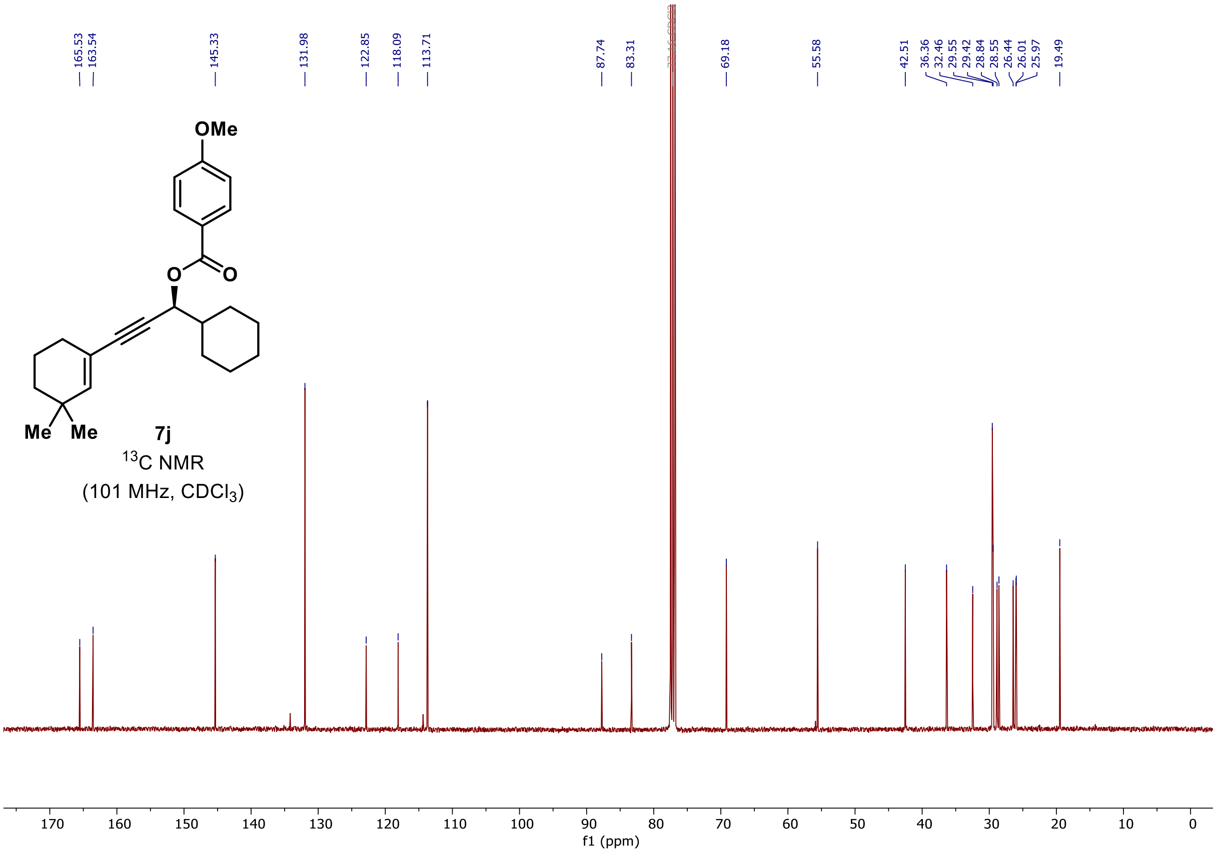
OMe
Me
Me
7j
^{13}C NMR
(101 MHz, CDCl$_3$)
f1 (ppm)

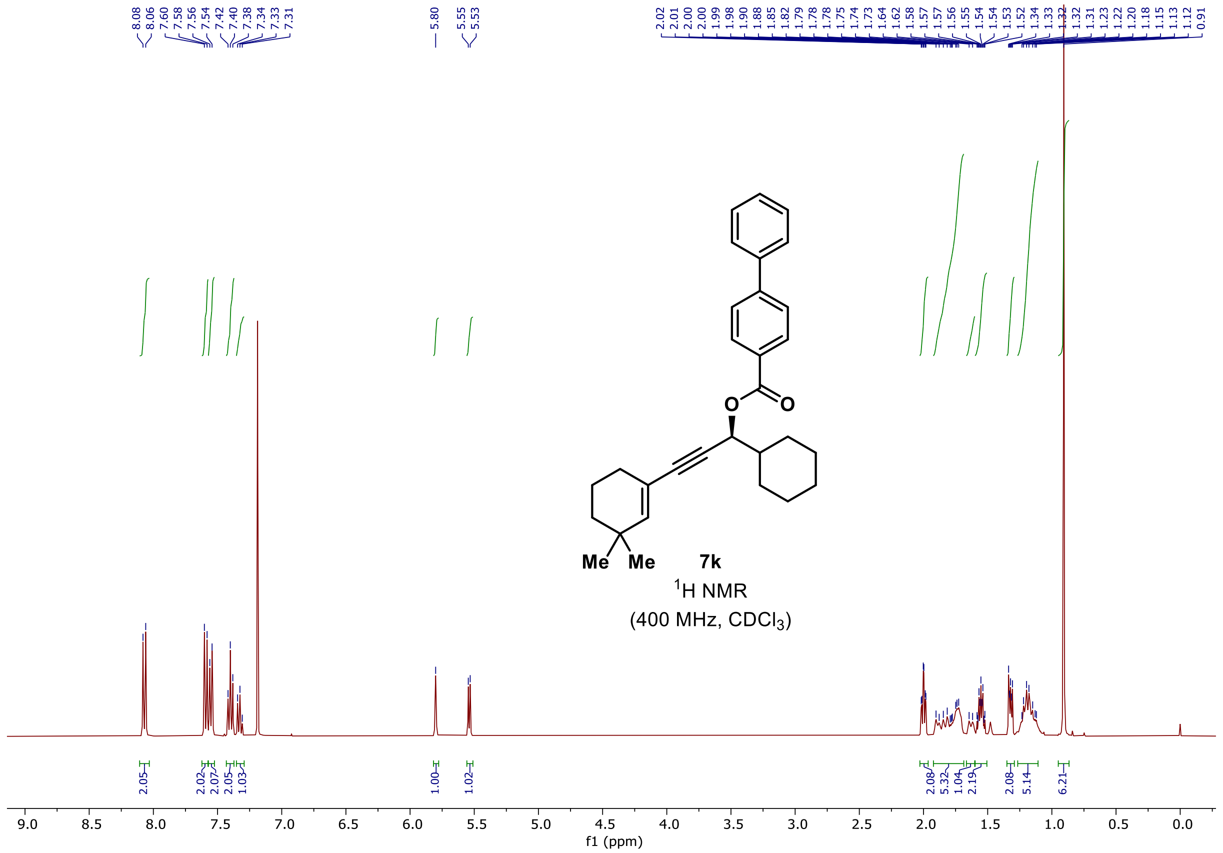

Me
Me
7k
^{1}H NMR
(400 MHz, CDCl$_3$)
f1 (ppm)

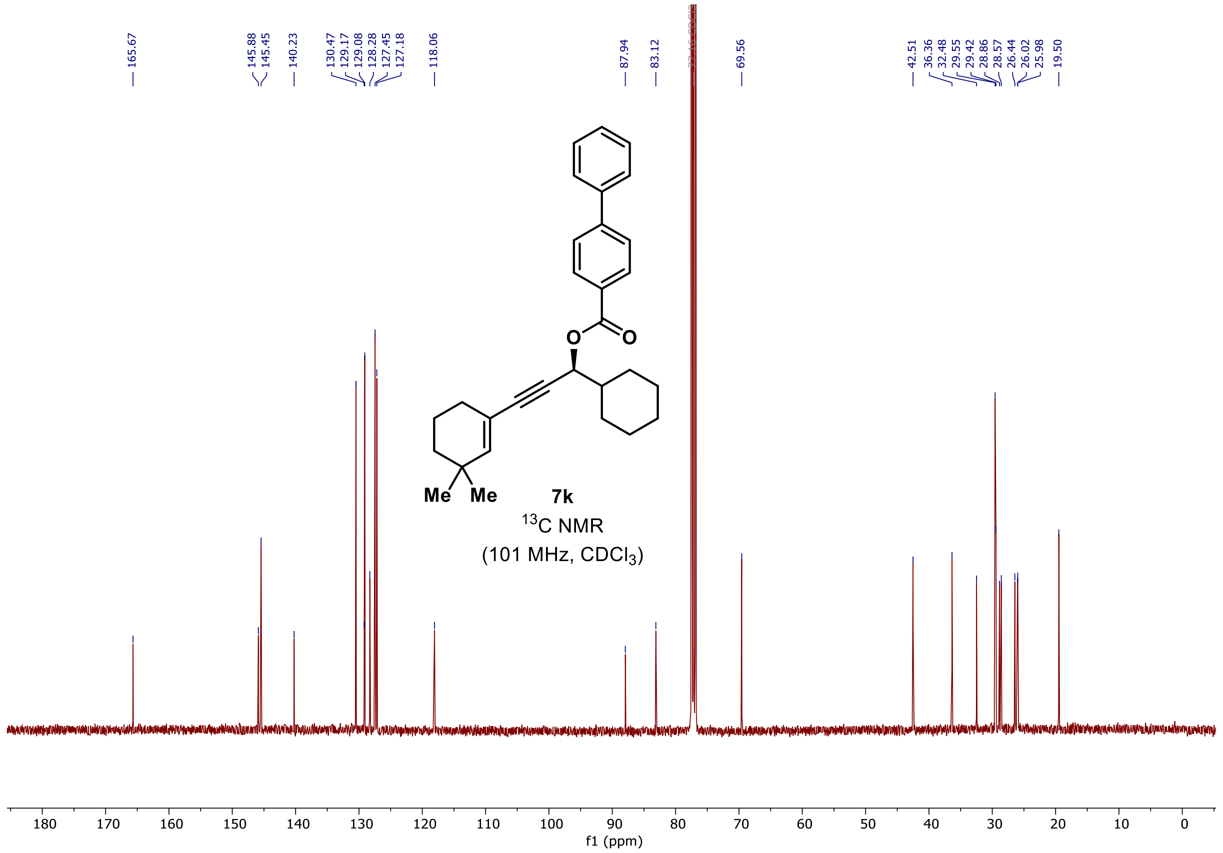

Me
Me
7k
^{13}C NMR
(101 MHz, CDCl$_3$)
f1 (ppm)

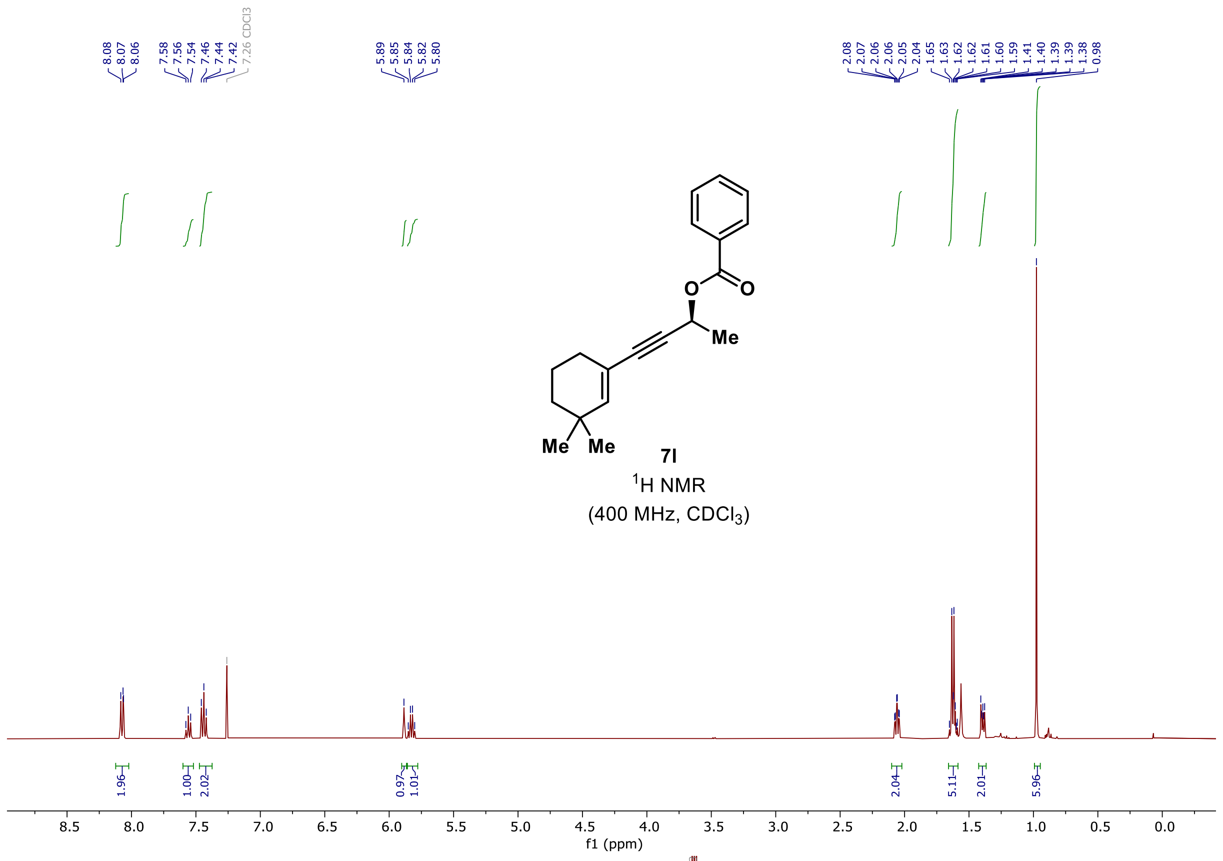

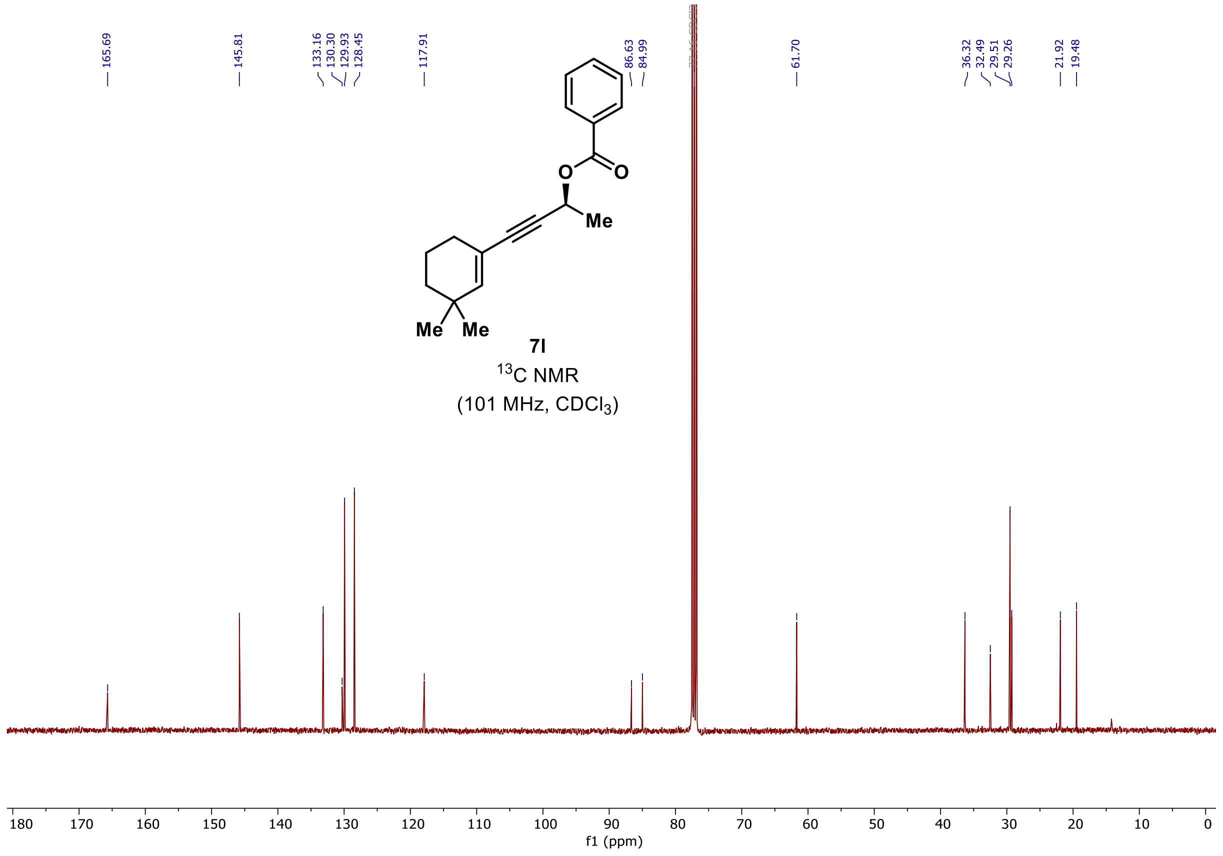

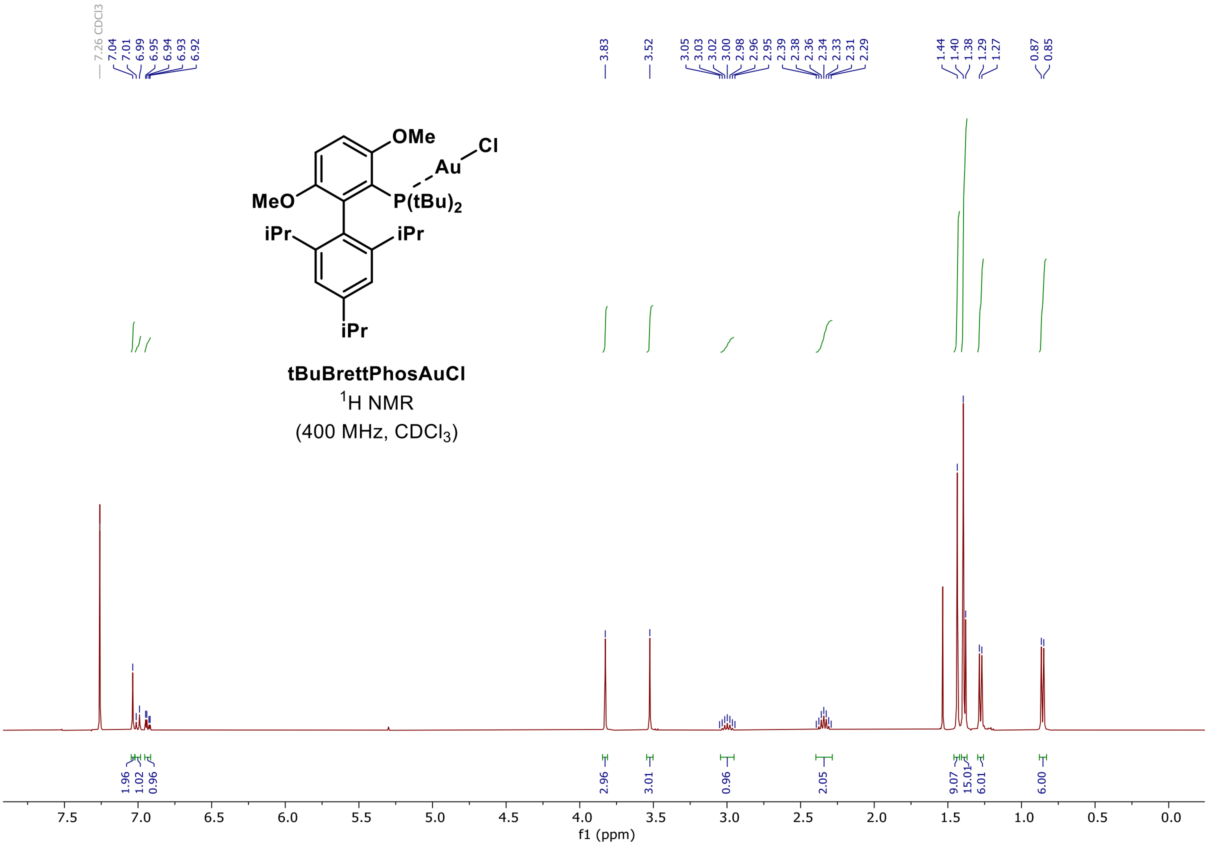
OMe
Au
Cl
MeO
P(tBu)2
iPr
iPr
iPr
tBuBrettPhosAuCl
1H NMR
(400 MHz, CDCl3)
f1 (ppm)

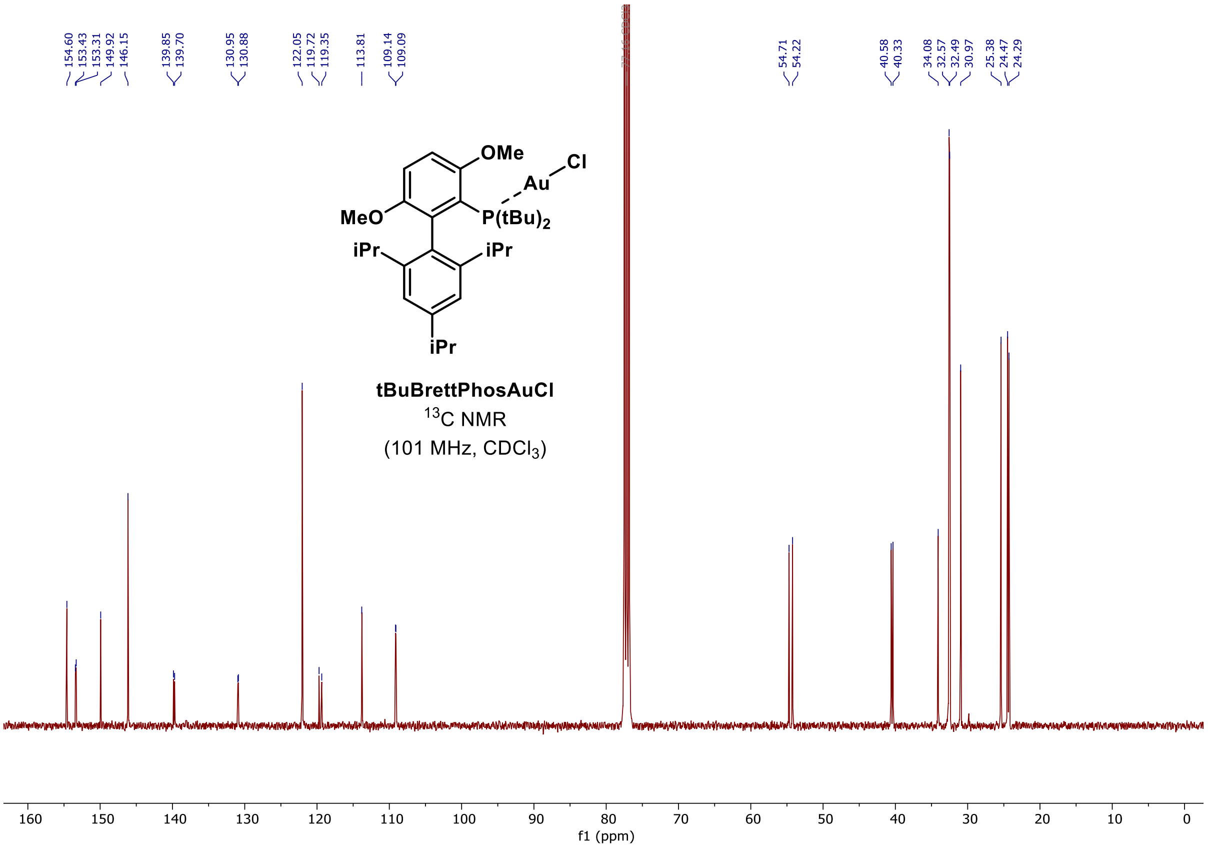
OMe
Au
Cl
MeO
P(tBu)2
iPr
iPr
iPr
tBuBrettPhosAuCl
13C NMR
(101 MHz, CDCl3)
f1 (ppm)

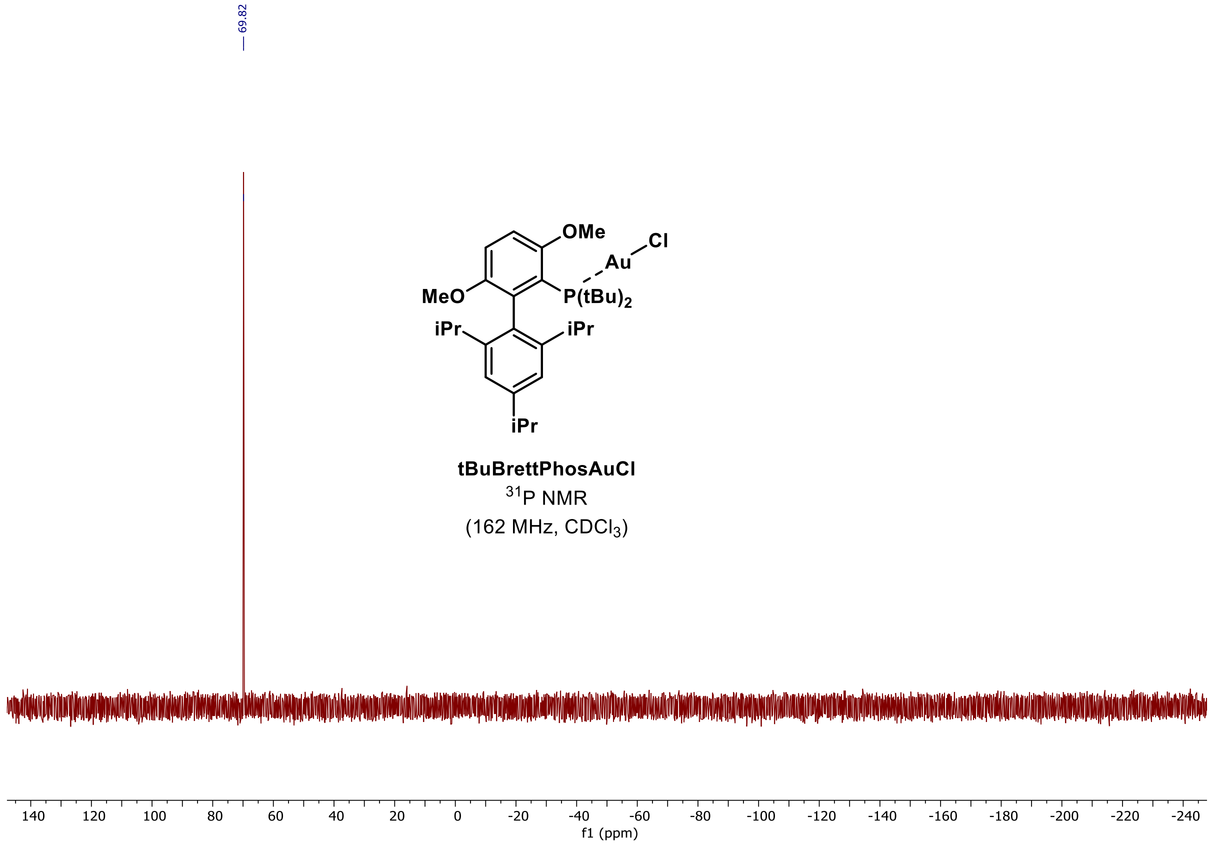
69.82
OMe
Cl
Au
MeO
P(tBu)2
iPr
iPr
iPr
tBuBrettPhosAuCl
31P NMR
(162 MHz, CDCl3)
140
120
100
80
60
40
20
0
-20
-40
-60
-80
-100
-120
-140
-160
-180
-200
-220
-240
f1 (ppm)

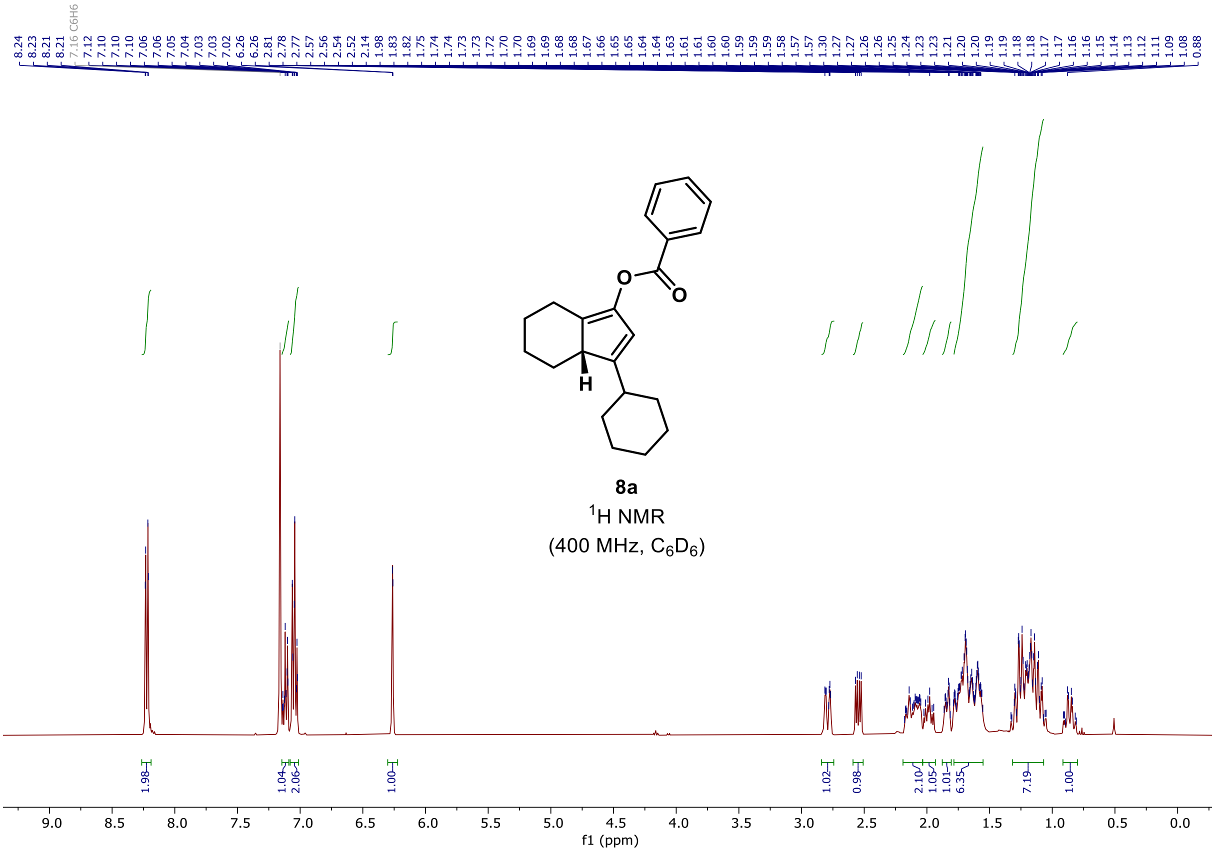
8.24
8.23
8.21
8.21
7.16 C6H6
7.12
7.10
7.10
7.10
7.06
7.06
7.05
7.04
7.03
7.03
7.02
6.26
6.26
2.81
2.78
2.77
2.57
2.56
2.54
2.52
2.14
1.98
1.83
1.82
1.75
1.74
1.74
1.73
1.73
1.72
1.70
1.70
1.69
1.69
1.68
1.67
1.66
1.65
1.65
1.64
1.64
1.63
1.61
1.61
1.60
1.59
1.59
1.59
1.58
1.57
1.57
1.30
1.27
1.27
1.26
1.26
1.25
1.24
1.23
1.23
1.21
1.20
1.20
1.19
1.19
1.18
1.18
1.17
1.17
1.16
1.16
1.15
1.14
1.13
1.12
1.11
1.09
1.08
0.88
O
O
H
8a
^{1}H NMR
(400 MHz, C_6D_6)
1.98
1.04
2.06
1.00
1.02
0.98
2.10
1.05
1.01
6.35
7.19
1.00
9.0
8.5
8.0
7.5
7.0
6.5
6.0
5.5
5.0
4.5
4.0
3.5
3.0
2.5
2.0
1.5
1.0
0.5
0.0
f1 (ppm)

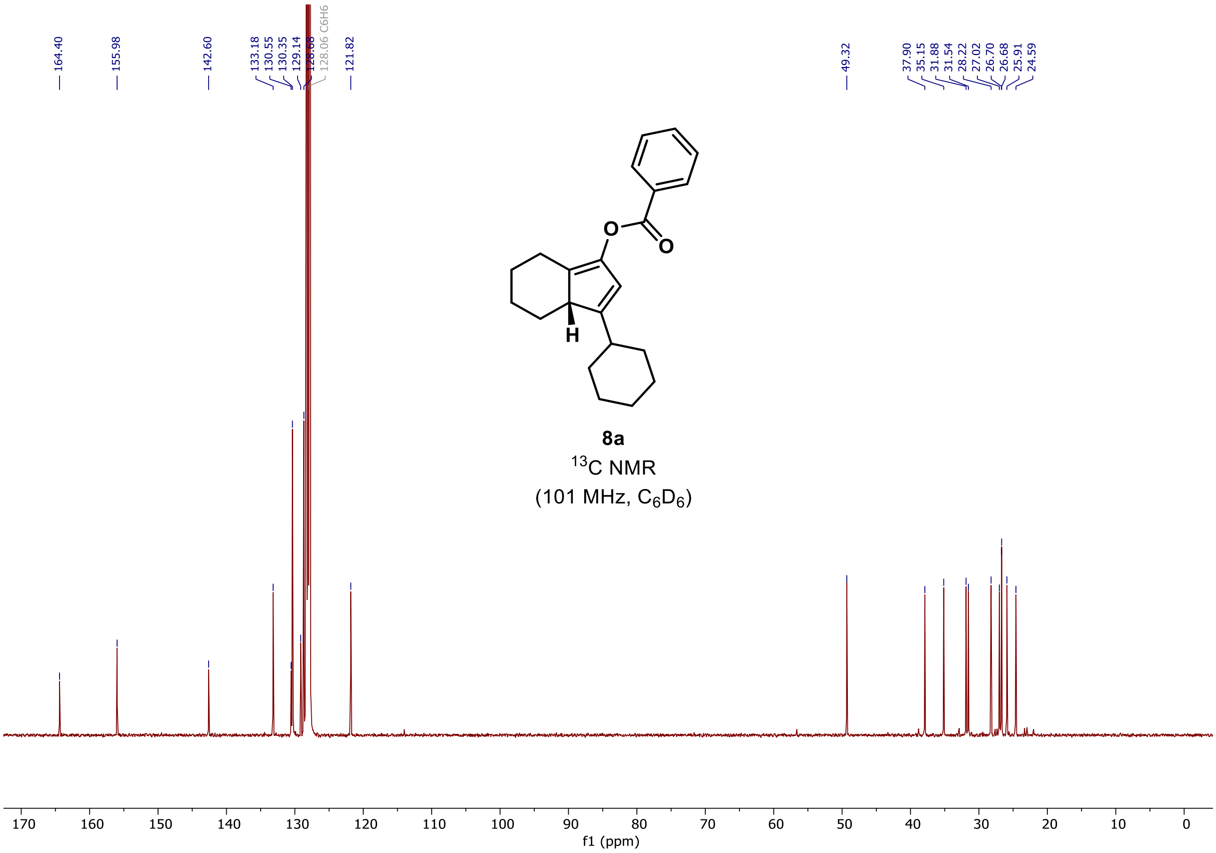
164.40
155.98
142.60
133.18
130.55
130.35
129.14
128.68
128.06 C6H6
121.82
49.32
37.90
35.15
31.88
31.54
28.22
27.02
26.70
26.68
25.91
24.59
O
O
H
8a
^{13}C NMR
(101 MHz, C_6D_6)
170
160
150
140
130
120
110
100
90
80
70
60
50
40
30
20
10
0
f1 (ppm)

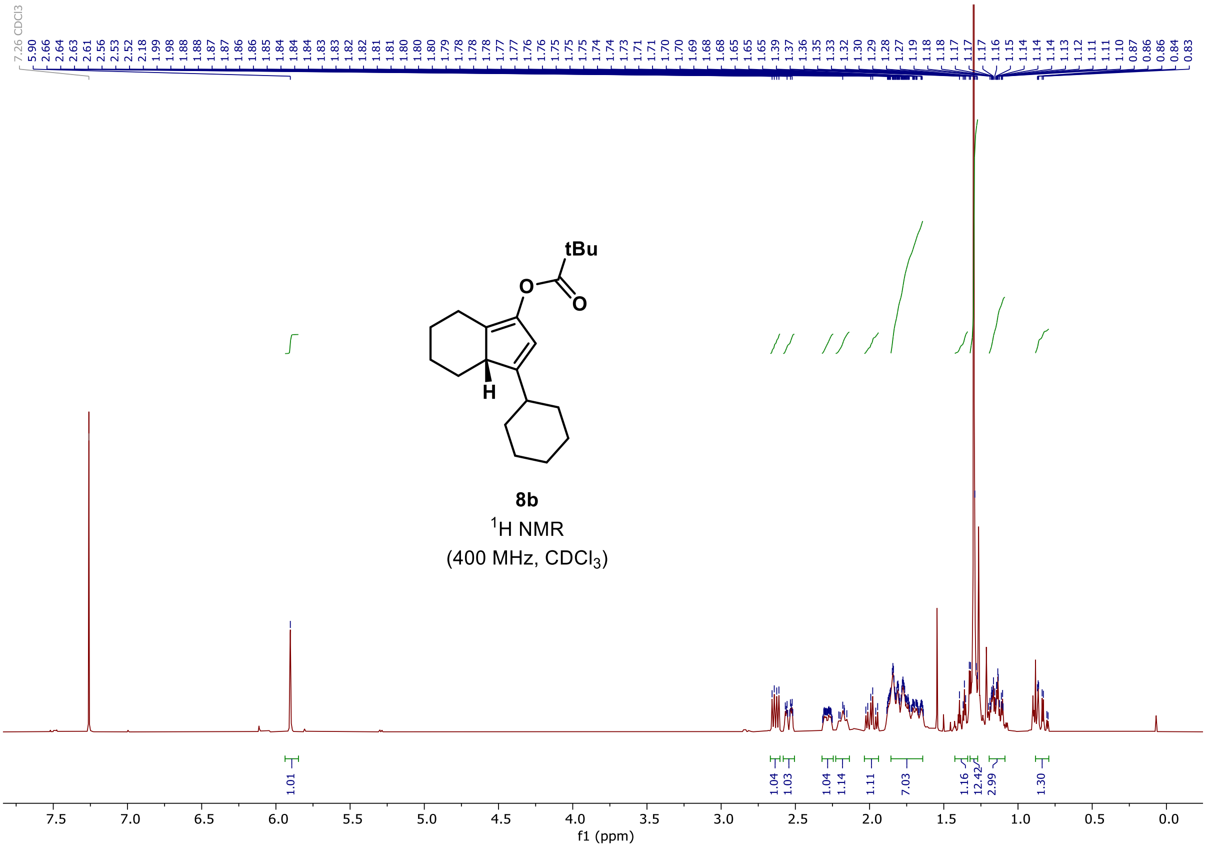
tBu
O
O
H
8b
1H NMR
(400 MHz, CDCl3)
f1 (ppm)

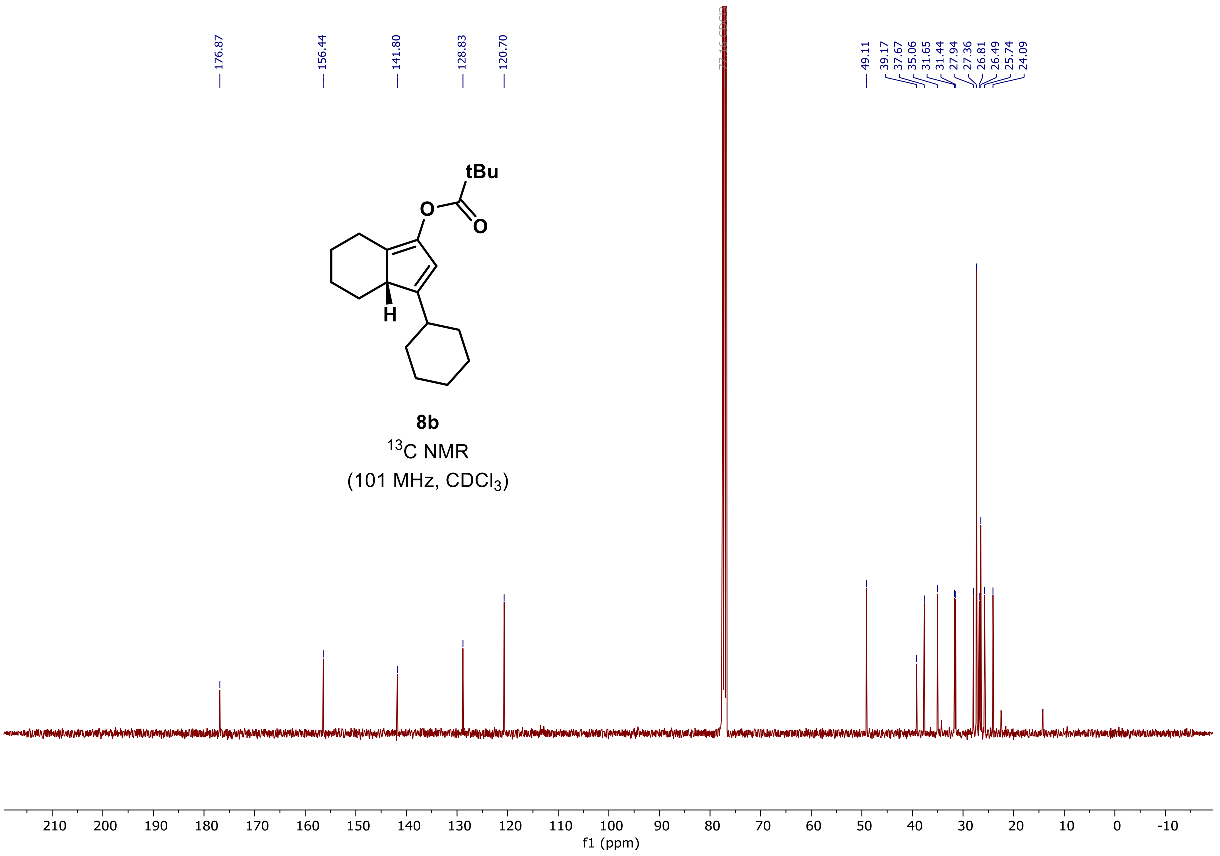
176.87
156.44
141.80
128.83
120.70
49.11
39.17
37.67
35.06
31.65
31.44
27.94
27.36
26.81
26.49
25.74
24.09
tBu
O
O
H
8b
13C NMR
(101 MHz, CDCl3)
f1 (ppm)

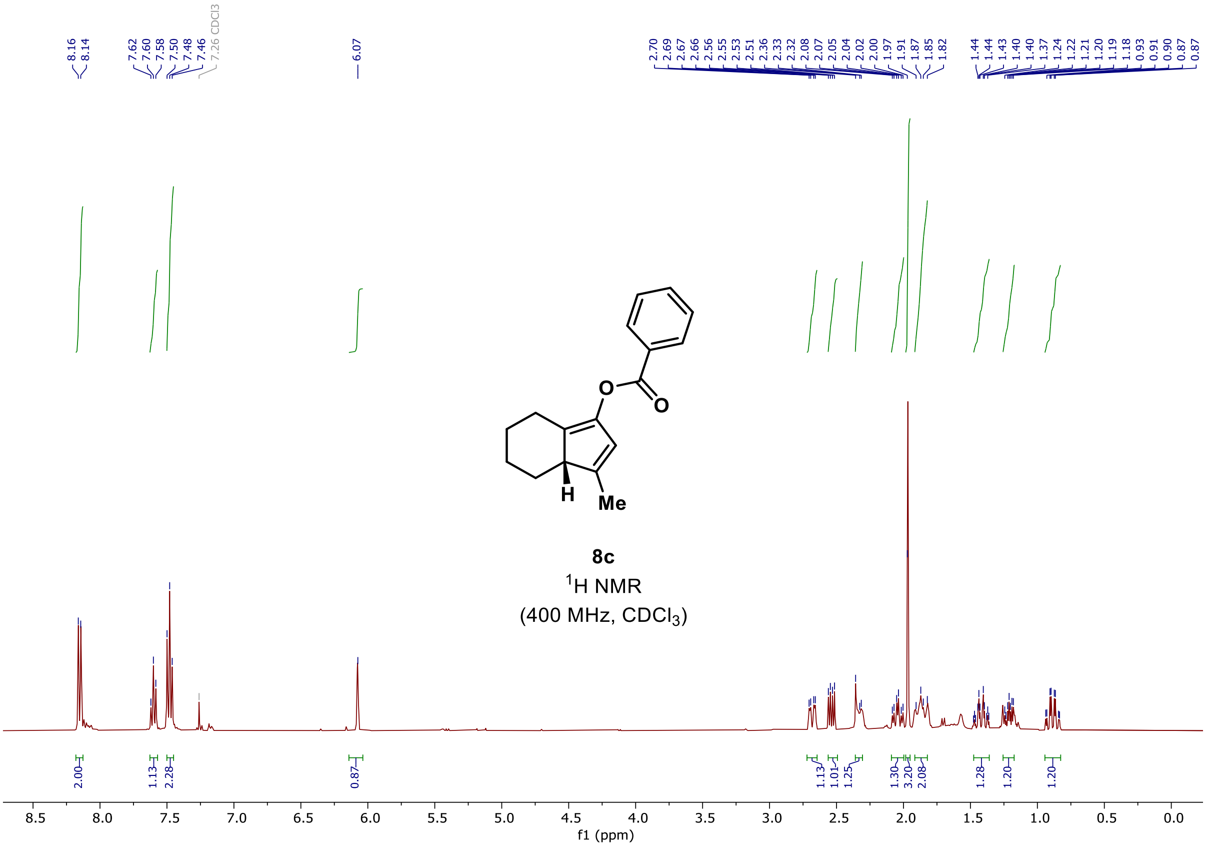
O
O
H
Me
8c
1H NMR
(400 MHz, CDCl3)
f1 (ppm)

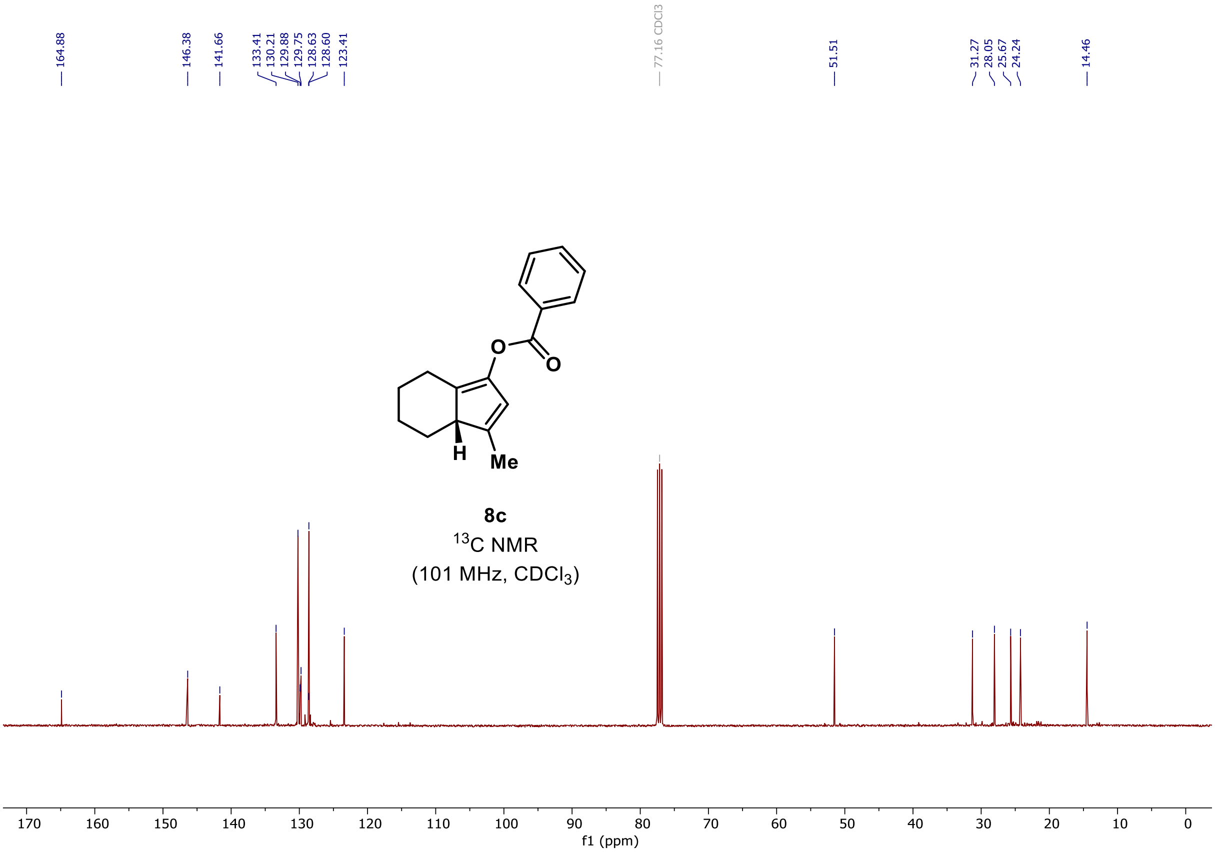
164.88
146.38
141.66
133.41
130.21
129.88
129.75
128.63
128.60
123.41
77.16 CDCl3
51.51
31.27
28.05
25.67
24.24
14.46
O
O
H
Me
8c
13C NMR
(101 MHz, CDCl3)
f1 (ppm)

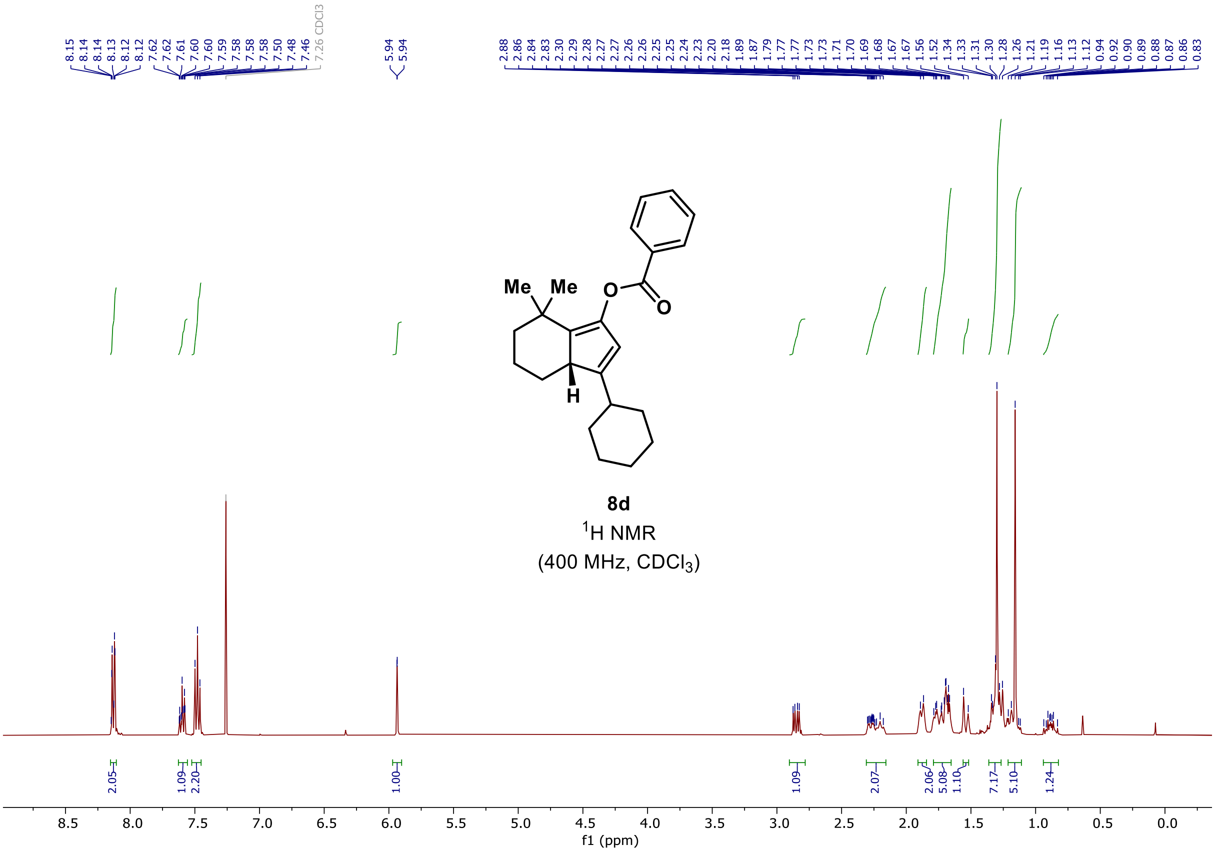
8.15 8.14 8.14 8.13 8.12 8.12 7.62 7.61 7.60 7.60 7.59 7.58 7.58 7.58 7.50 7.48 7.46 7.26 CDCl3
5.94 5.94
2.88 2.86 2.84 2.83 2.30 2.29 2.28 2.27 2.27 2.26 2.26 2.25 2.25 2.24 2.23 2.20 2.18 1.89 1.87 1.79 1.77 1.77 1.73 1.73 1.71 1.70 1.69 1.68 1.67 1.67 1.56 1.52 1.34 1.33 1.31 1.30 1.28 1.26 1.21 1.19 1.16 1.13 1.12 0.94 0.92 0.90 0.89 0.88 0.87 0.86 0.83
Me
Me
O
O
H
8d
^{1}H NMR
(400 MHz, $CDCl_3$)
2.05 1.09 2.20 1.00 1.09 2.07 2.06 5.08 1.10 7.17 5.10 1.24
8.5 8.0 7.5 7.0 6.5 6.0 5.5 5.0 4.5 4.0 3.5 3.0 2.5 2.0 1.5 1.0 0.5 0.0
f1 (ppm)

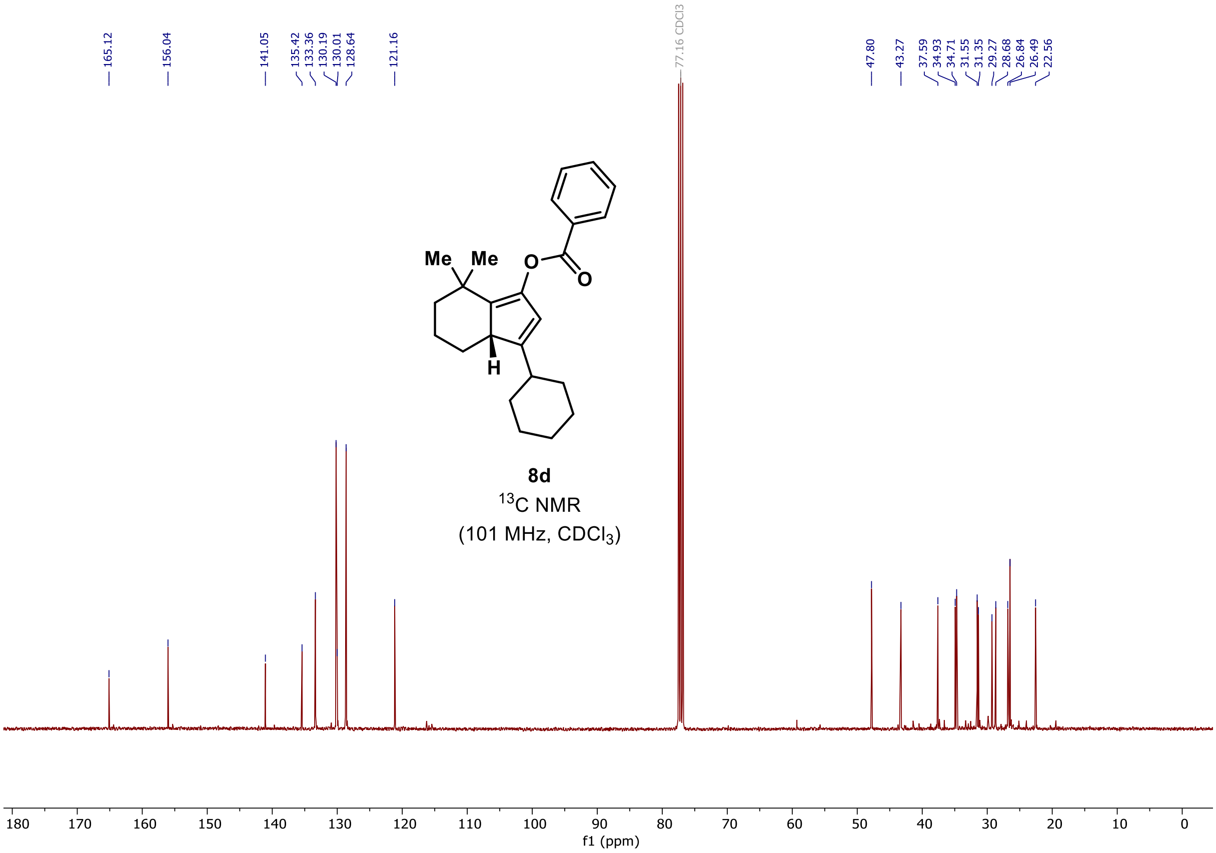
165.12
156.04
141.05 135.42 133.36 130.19 130.01 128.64
121.16
77.16 CDCl3
47.80 43.27 37.59 34.93 34.71 31.55 31.35 29.27 28.68 26.84 26.49 22.56
Me
Me
O
O
H
8d
^{13}C NMR
(101 MHz, $CDCl_3$)
180 170 160 150 140 130 120 110 100 90 80 70 60 50 40 30 20 10 0
f1 (ppm)

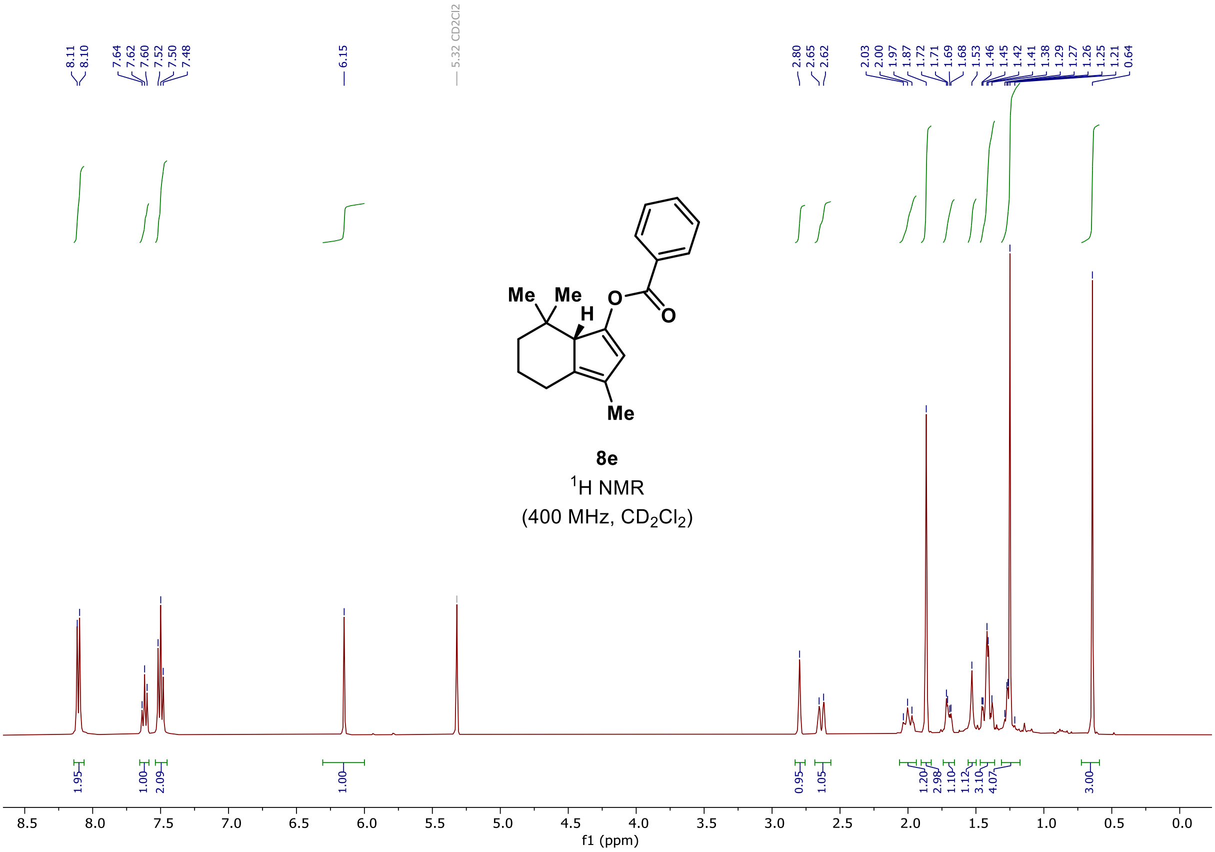
8e
^{1}H NMR
(400 MHz, CD_2Cl_2)
f1 (ppm)

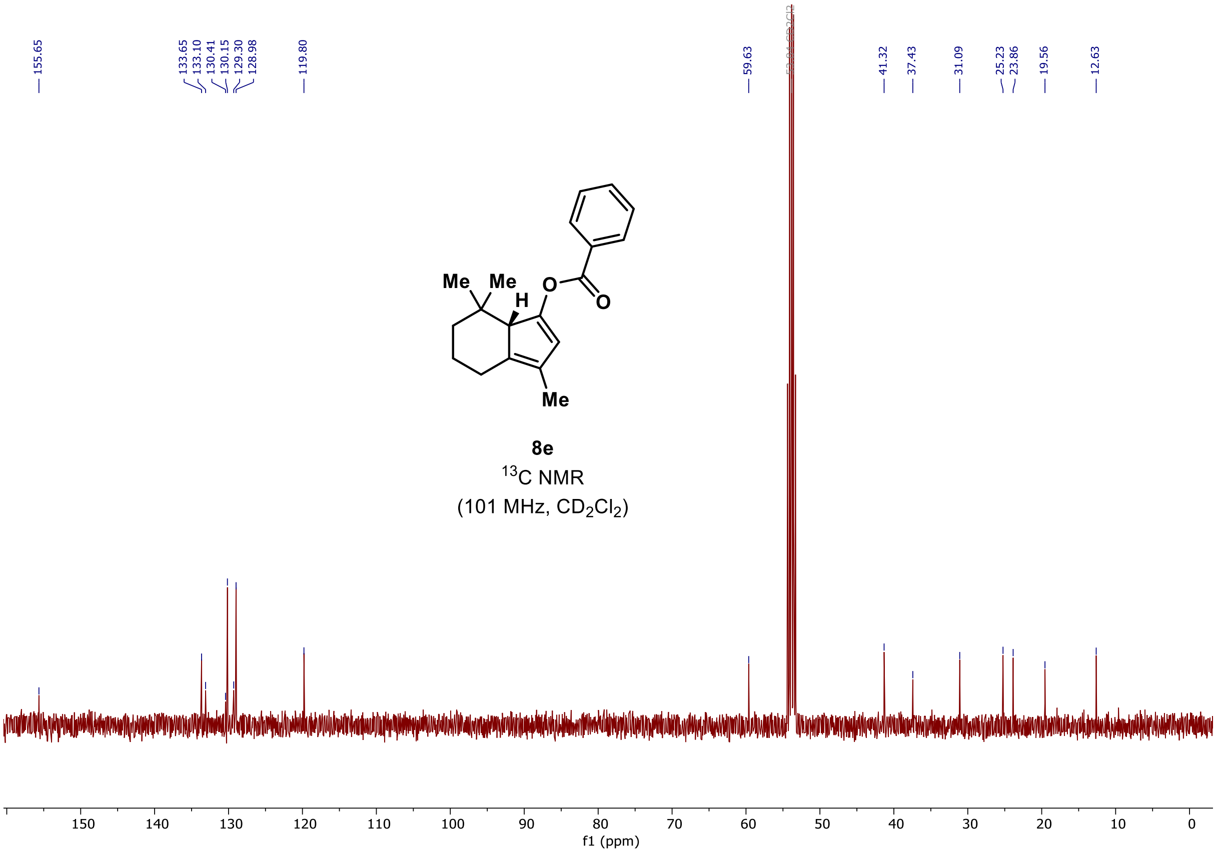
8e
^{13}C NMR
(101 MHz, CD_2Cl_2)
f1 (ppm)

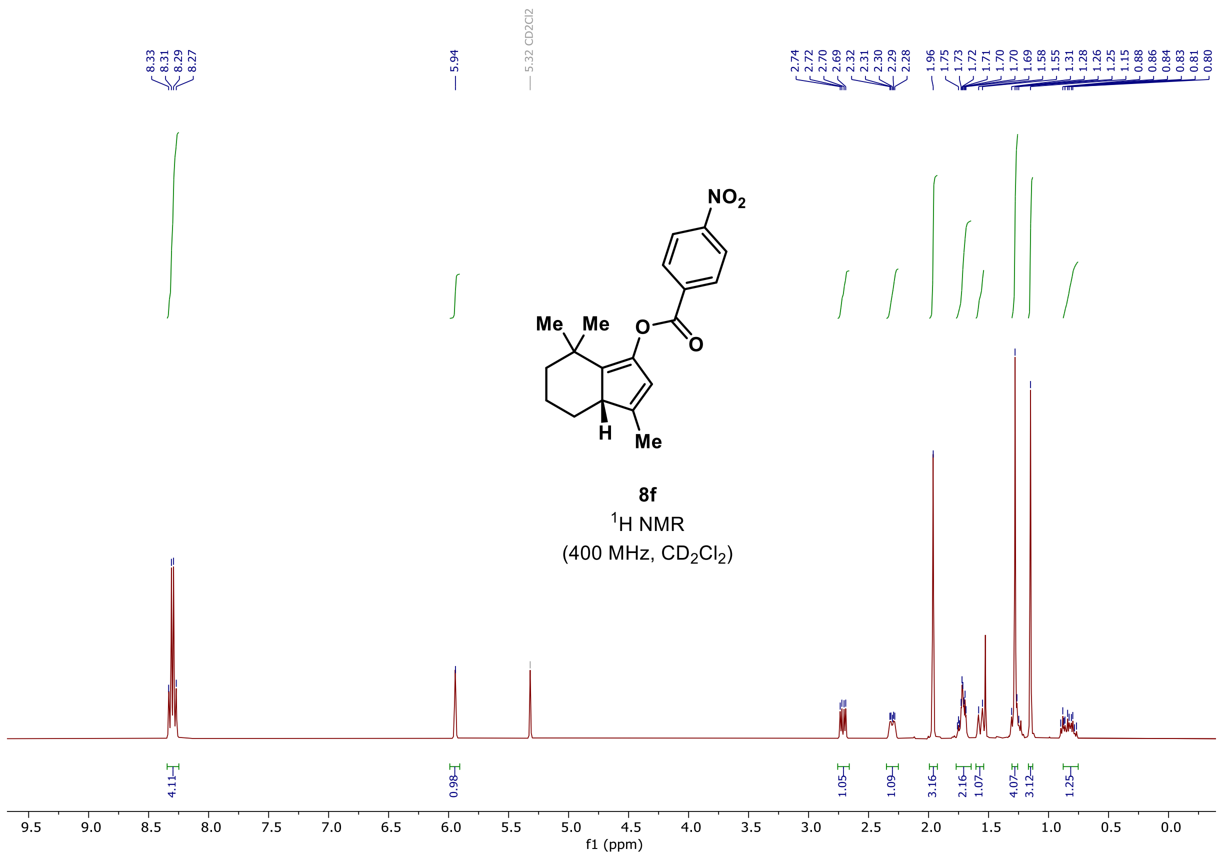
8.33
8.31
8.29
8.27
5.94
5.32 CD2Cl2
2.74
2.72
2.70
2.69
2.32
2.31
2.30
2.29
2.28
1.96
1.75
1.73
1.72
1.71
1.70
1.70
1.69
1.58
1.55
1.31
1.28
1.26
1.25
1.15
0.88
0.86
0.84
0.83
0.81
0.80
NO2
Me
Me
O
O
H
Me
8f
1H NMR
(400 MHz, CD2Cl2)
4.11
0.98
1.05
1.09
3.16
2.16
1.07
4.07
3.12
1.25
f1 (ppm)

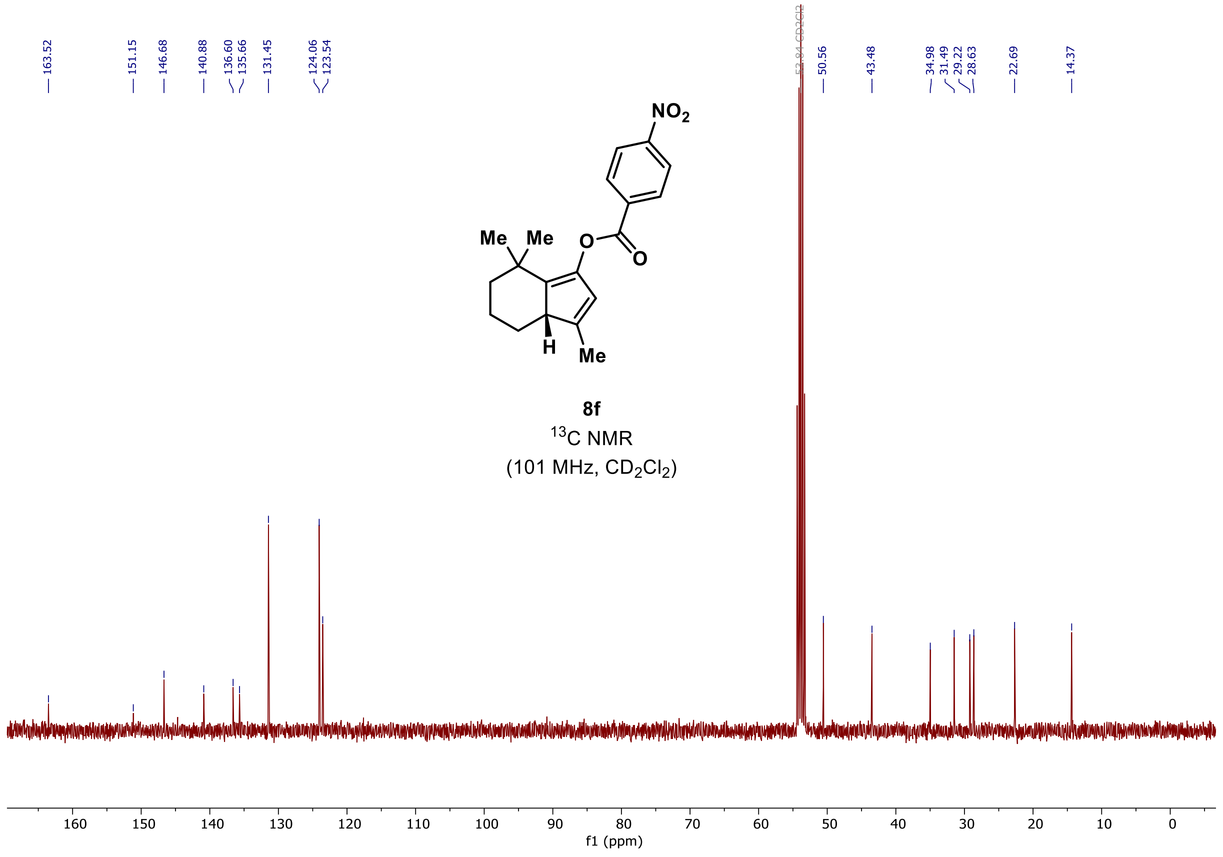
163.52
151.15
146.68
140.88
136.60
135.66
131.45
124.06
123.54
50.56
43.48
34.98
31.49
29.22
28.63
22.69
14.37
NO2
Me
Me
O
O
H
Me
8f
13C NMR
(101 MHz, CD2Cl2)
f1 (ppm)

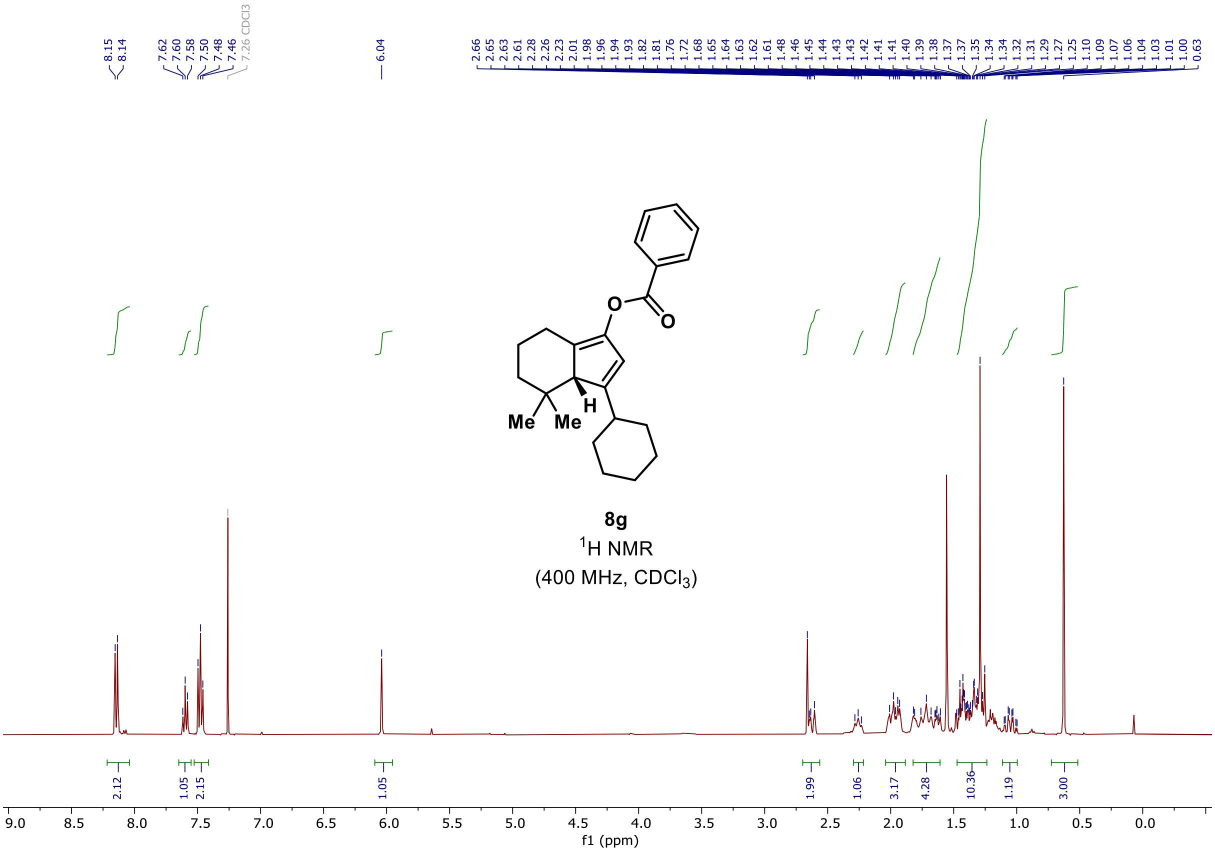
8g
1H NMR
(400 MHz, CDCl3)
Me
Me
H
O
O
f1 (ppm)

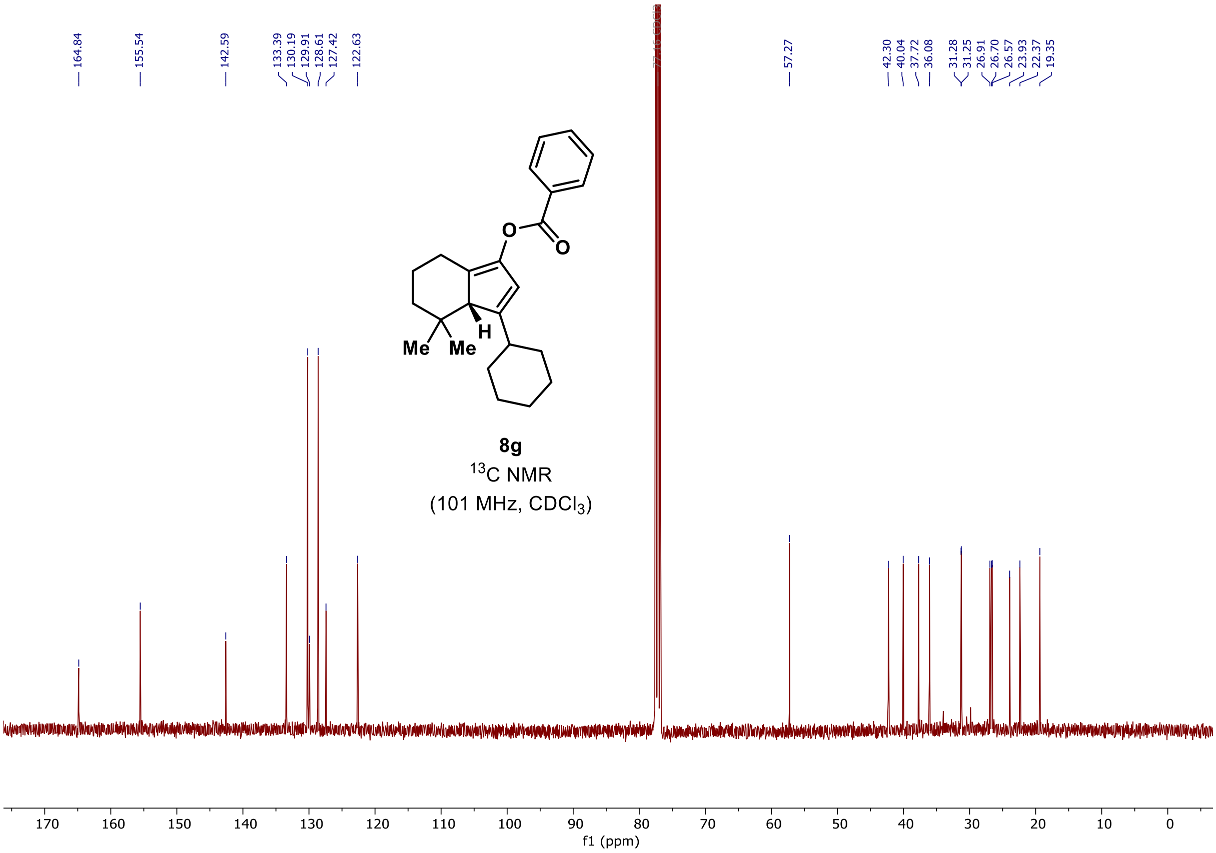
8g
13C NMR
(101 MHz, CDCl3)
Me
Me
H
O
O
f1 (ppm)

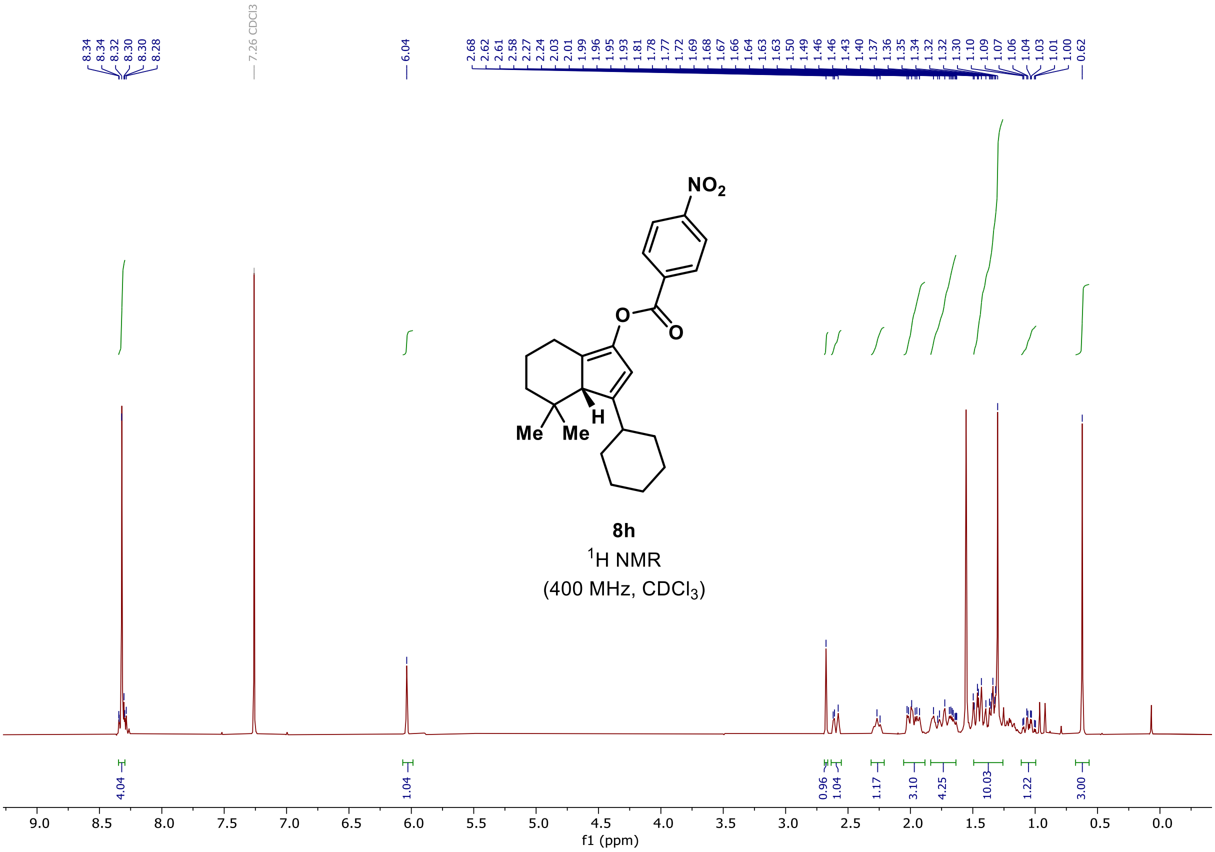
8h
^{1}H NMR
(400 MHz, CDCl$_3$)
f1 (ppm)

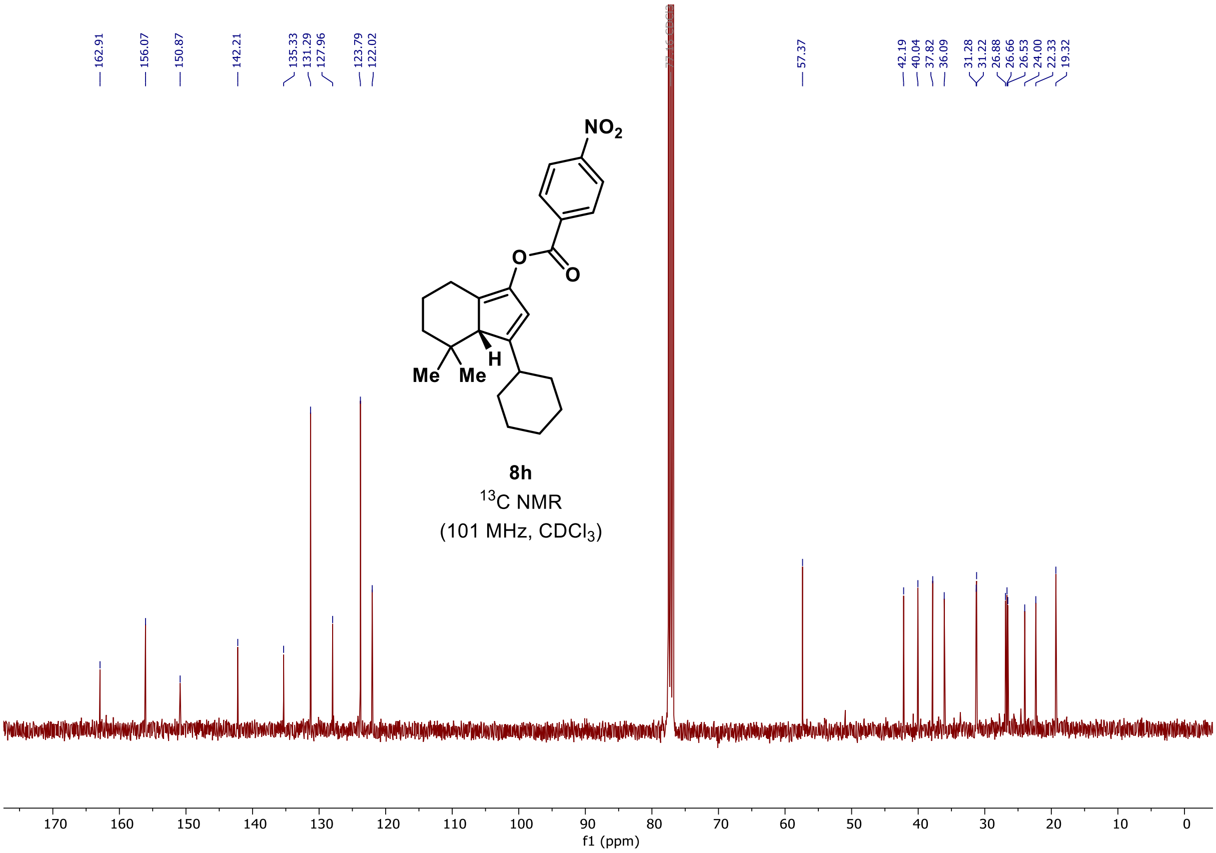
8h
^{13}C NMR
(101 MHz, CDCl$_3$)
f1 (ppm)

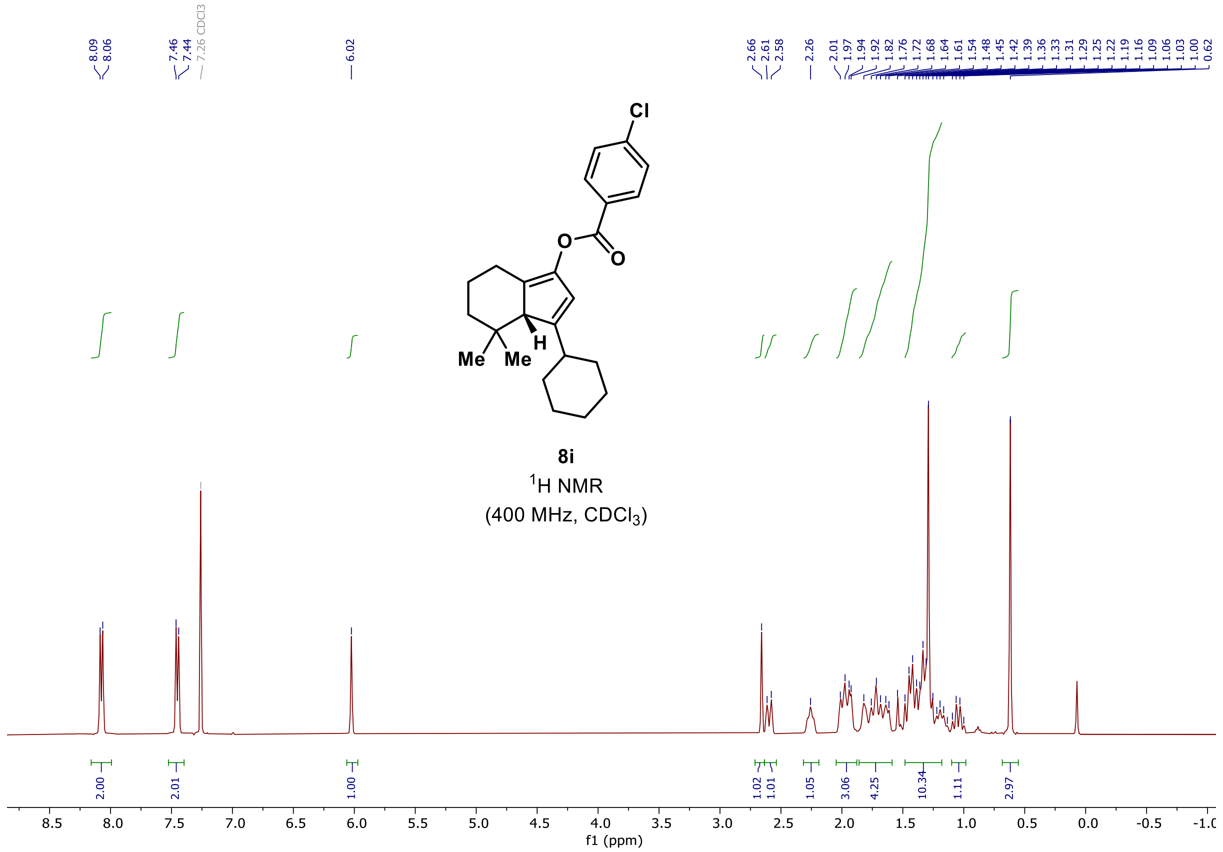
8i
1H NMR
(400 MHz, CDCl3)
f1 (ppm)

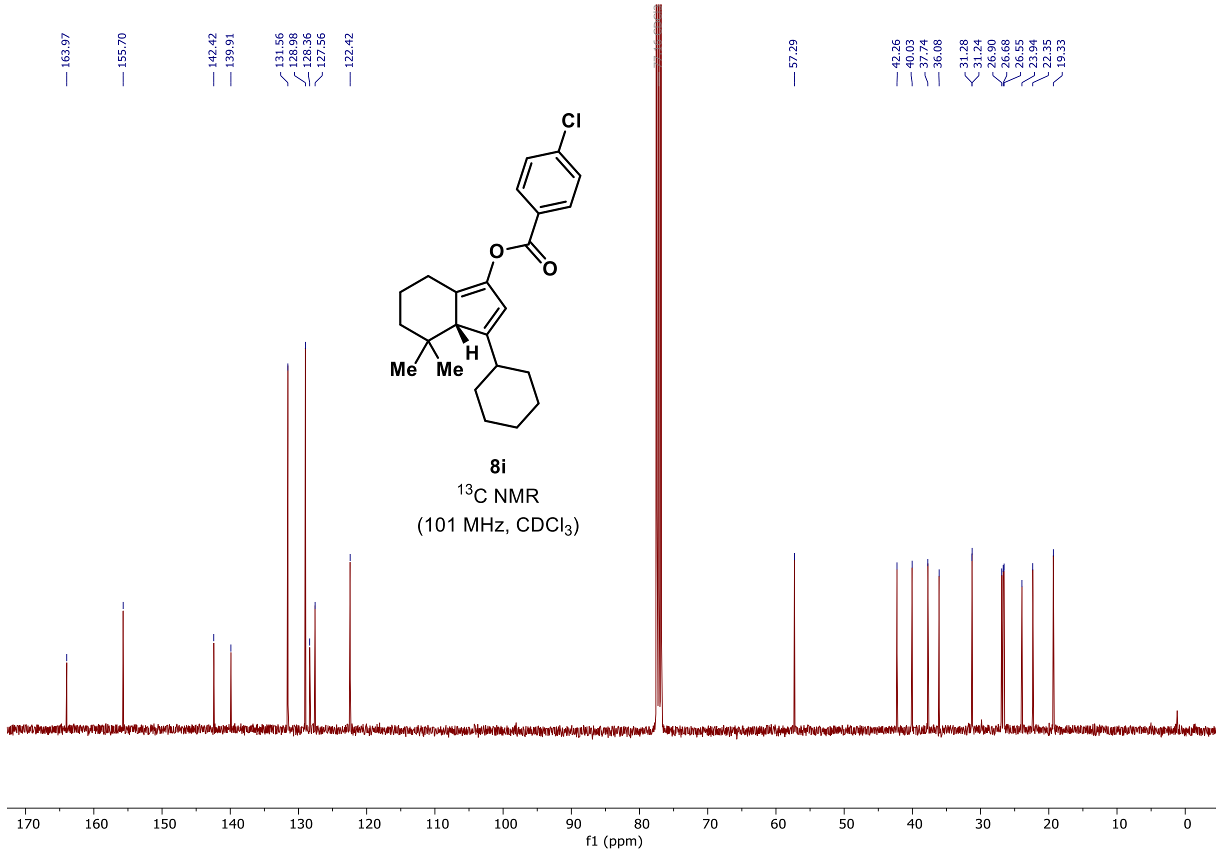
8i
13C NMR
(101 MHz, CDCl3)
f1 (ppm)

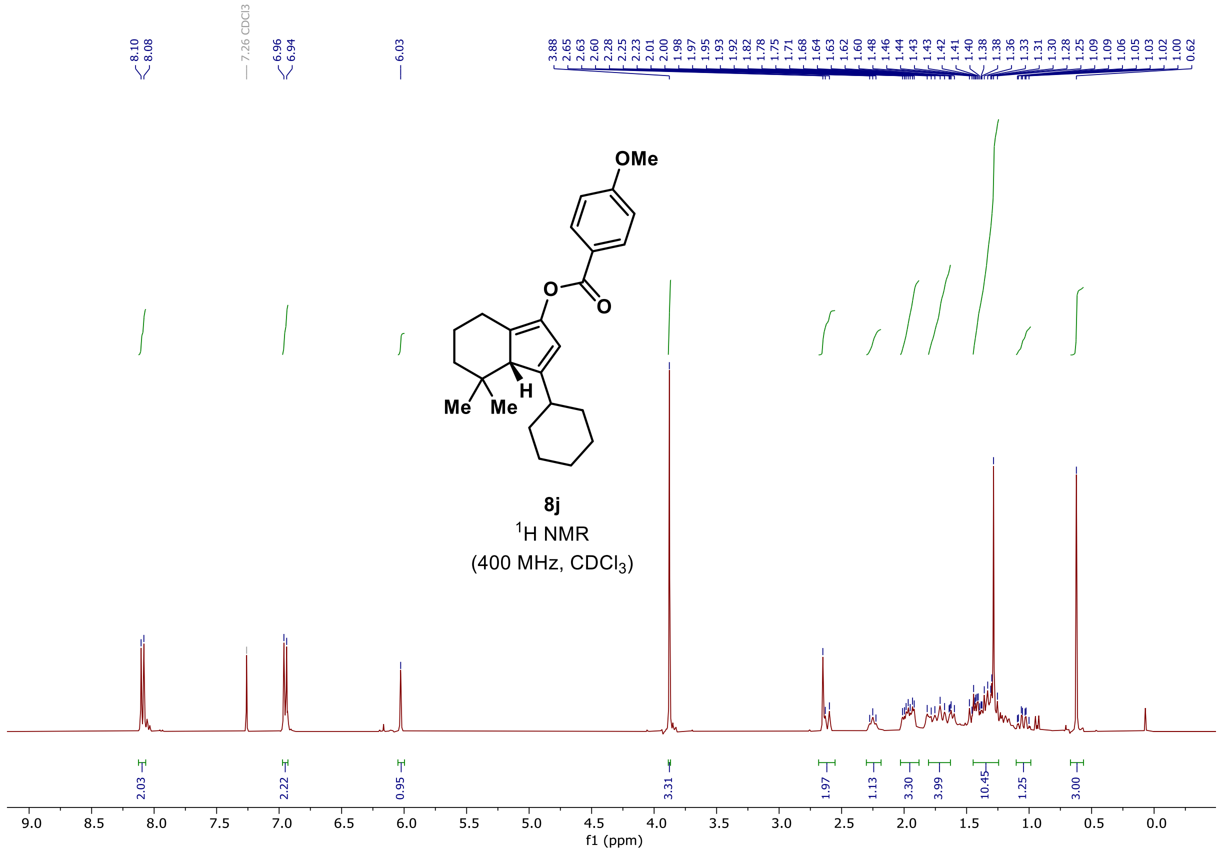
8j
^{1}H NMR
(400 MHz, $CDCl_3$)
f1 (ppm)

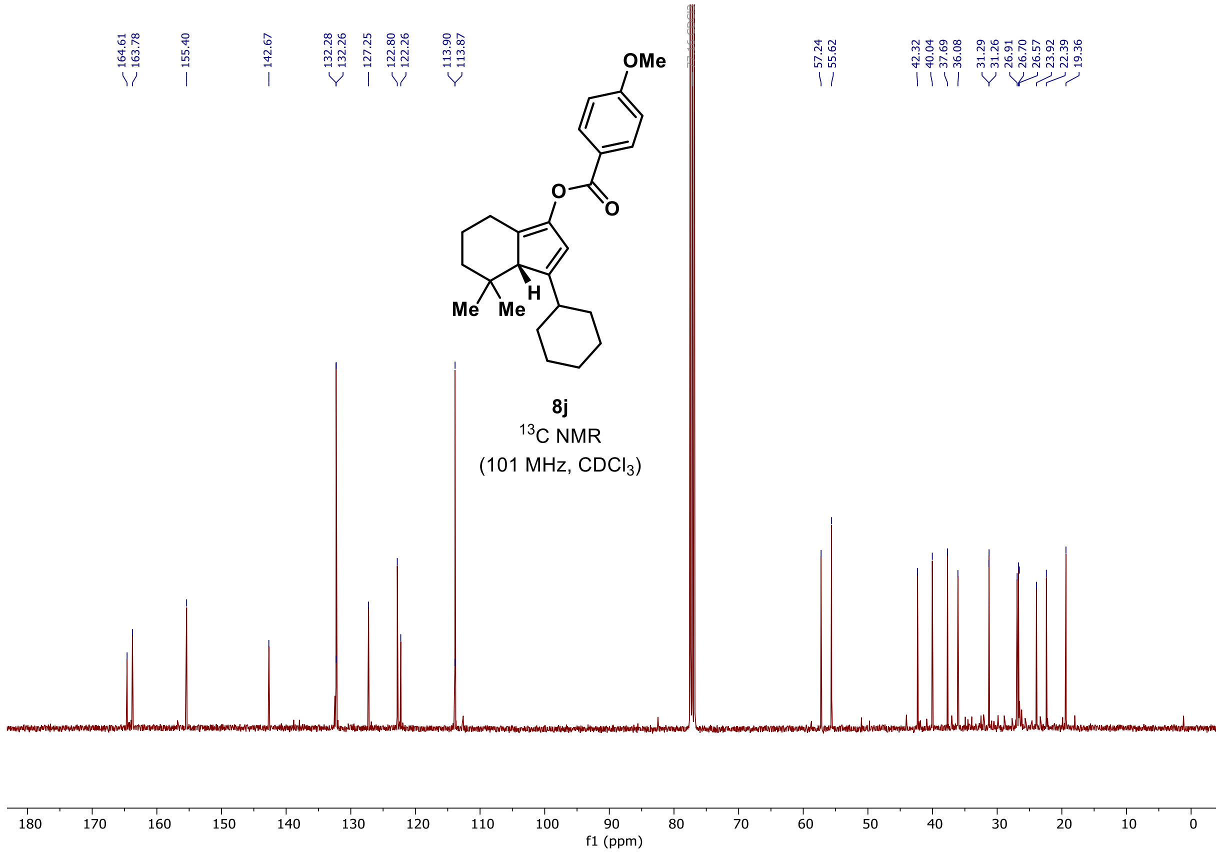
8j
^{13}C NMR
(101 MHz, $CDCl_3$)
f1 (ppm)

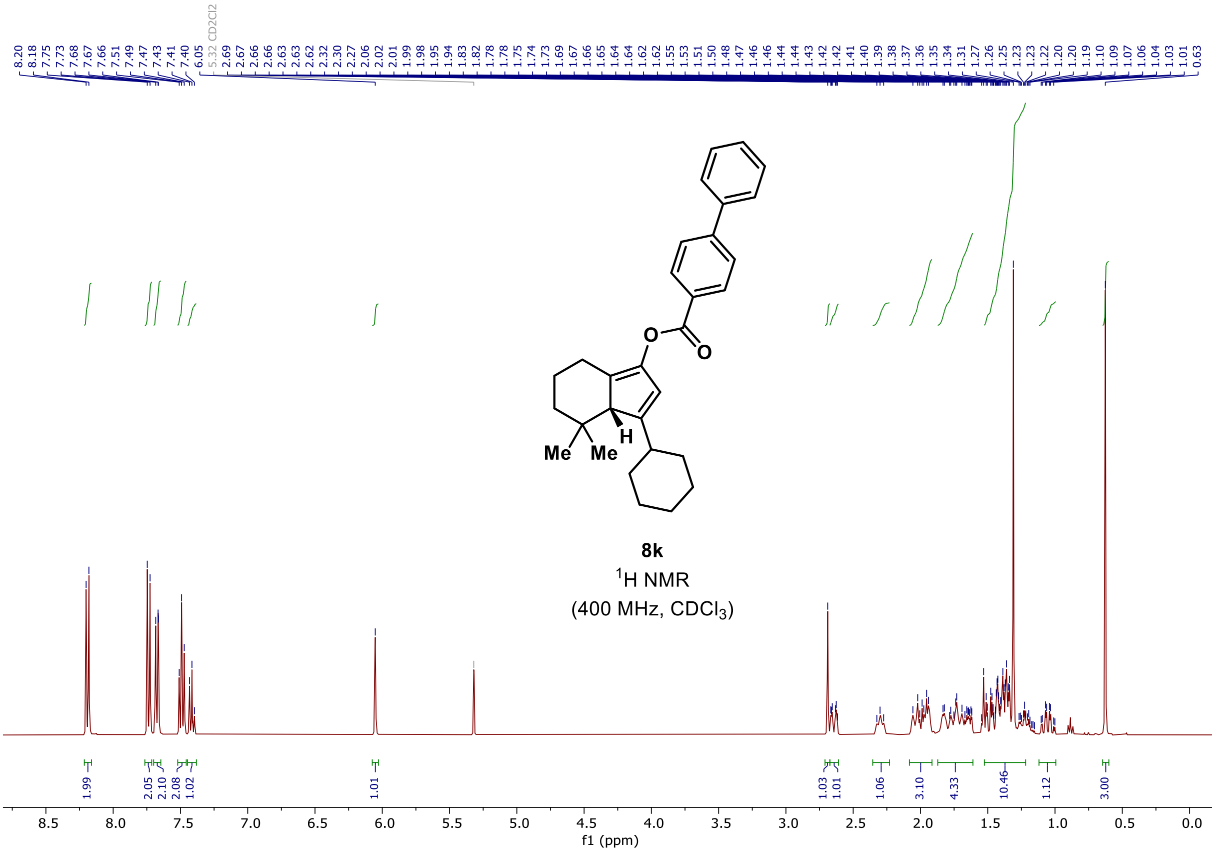

8k

^{1}H NMR

(400 MHz, $CDCl_3$)

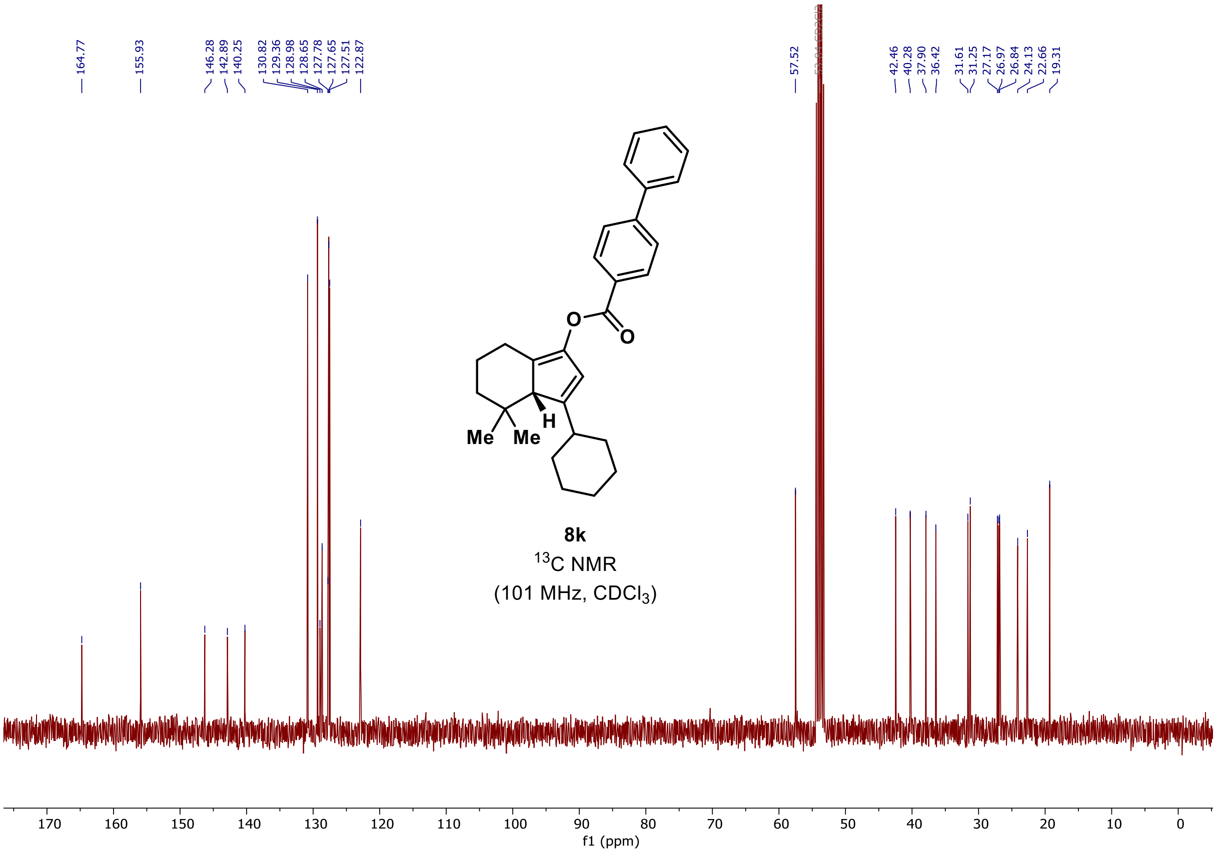

8k

^{13}C NMR

(101 MHz, $CDCl_3$)

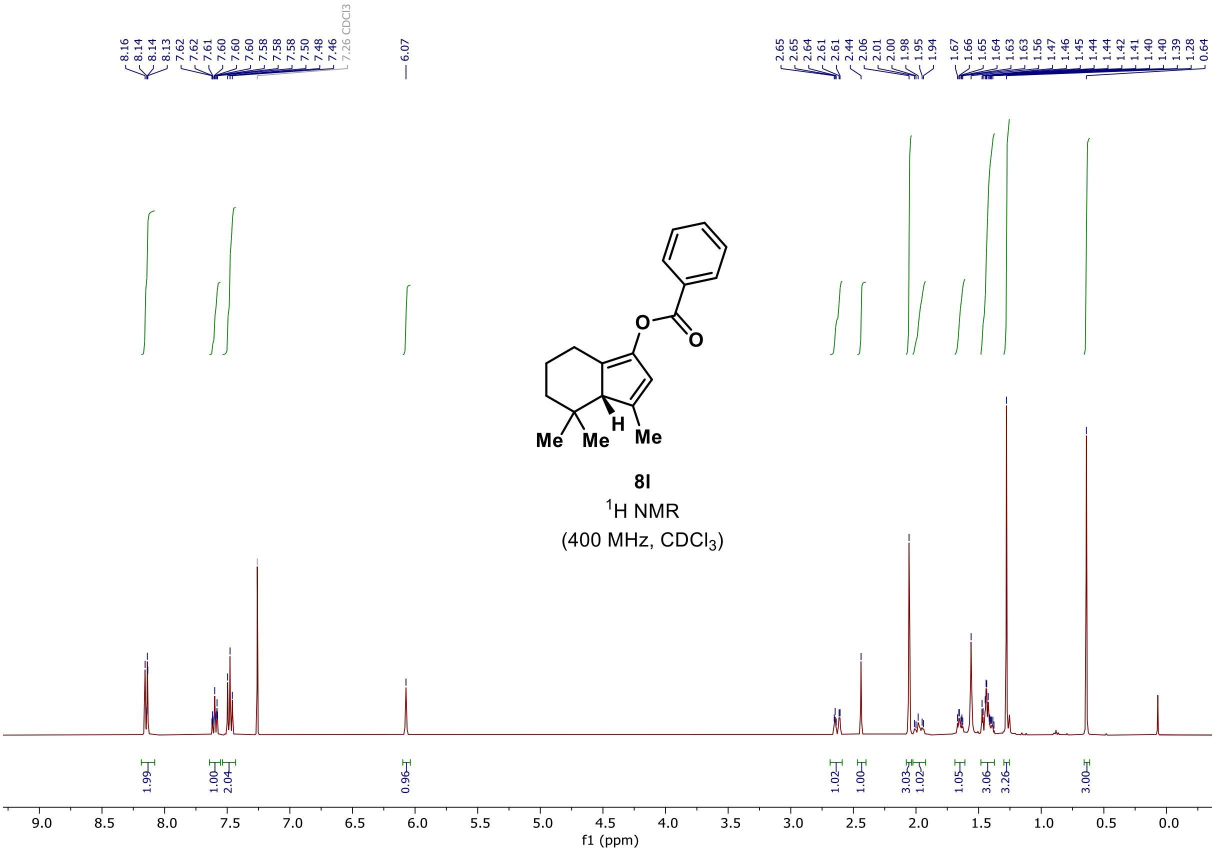
8.16
8.14
8.14
8.13
7.62
7.62
7.61
7.60
7.60
7.60
7.58
7.58
7.58
7.50
7.48
7.46
7.26 CDCl3
6.07
2.65
2.65
2.64
2.61
2.61
2.44
2.06
2.01
2.00
1.98
1.95
1.94
1.67
1.66
1.65
1.64
1.63
1.63
1.56
1.47
1.46
1.45
1.44
1.44
1.42
1.41
1.40
1.39
1.28
0.64
Me
Me
H
Me
O
O
8l
1H NMR
(400 MHz, CDCl3)
1.99
1.00
2.04
0.96
1.02
1.00
3.03
1.02
1.05
3.06
3.26
3.00
9.0
8.5
8.0
7.5
7.0
6.5
6.0
5.5
5.0
4.5
4.0
3.5
3.0
2.5
2.0
1.5
1.0
0.5
0.0
f1 (ppm)

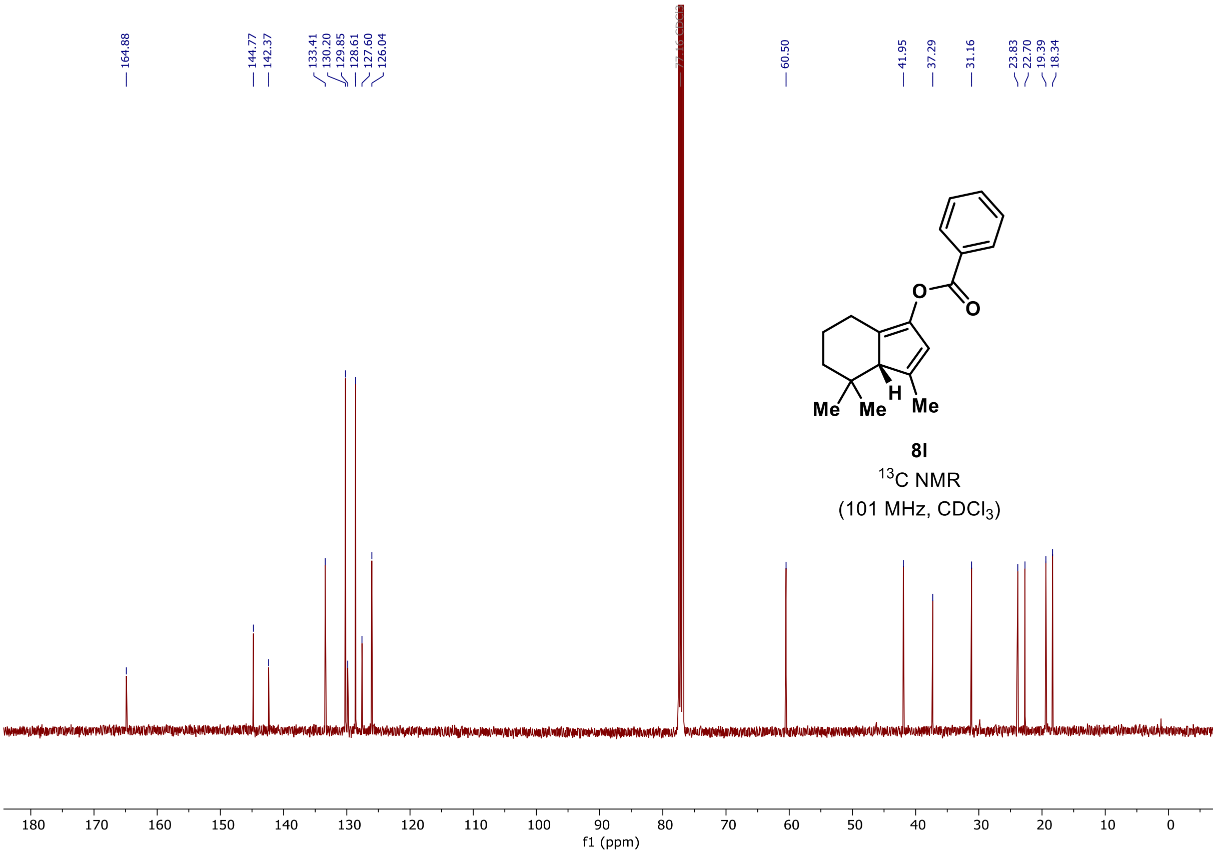
164.88
144.77
142.37
133.41
130.20
129.85
128.61
127.60
126.04
60.50
41.95
37.29
31.16
23.83
22.70
19.39
18.34
Me
Me
H
Me
O
O
8l
13C NMR
(101 MHz, CDCl3)
180
170
160
150
140
130
120
110
100
90
80
70
60
50
40
30
20
10
0
f1 (ppm)

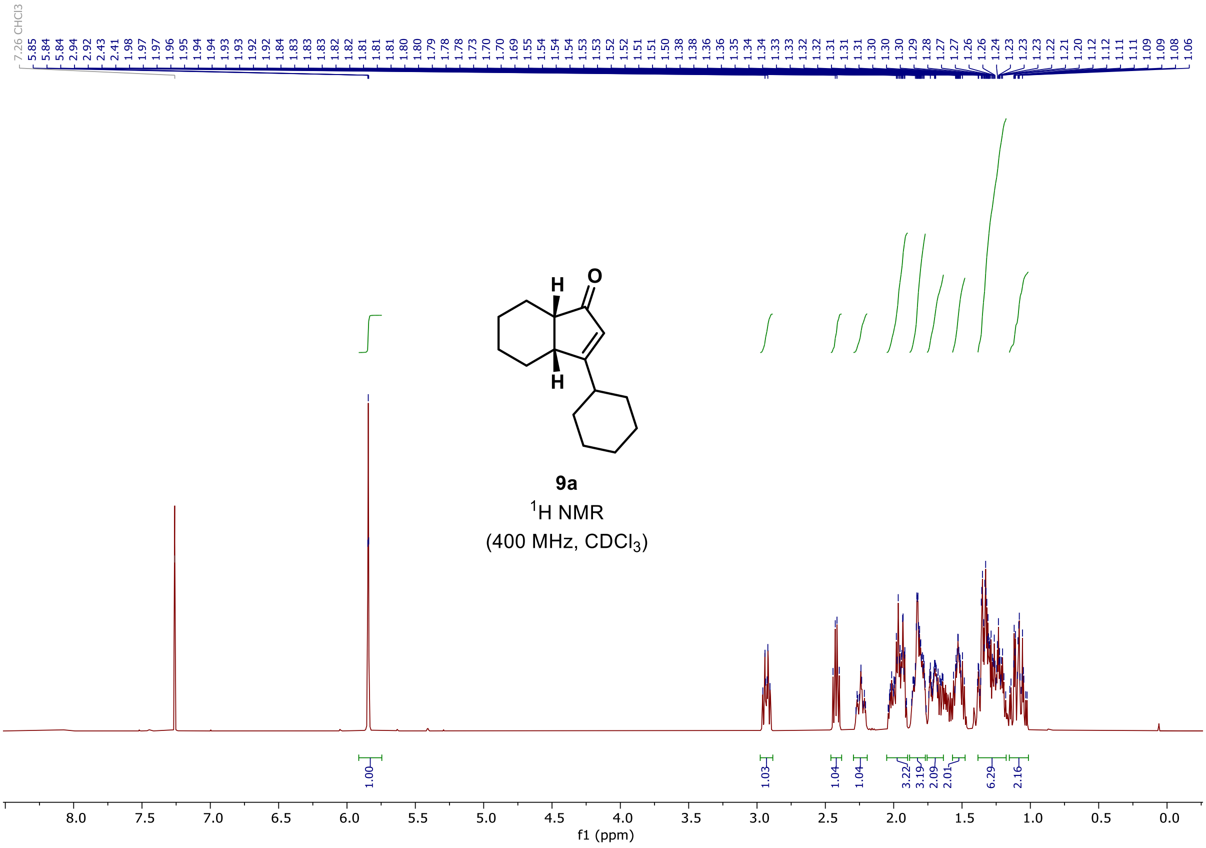
7.26 CHCl3
5.85 5.84 5.84 2.94 2.92 2.43 2.41 1.98 1.97 1.97 1.96 1.95 1.94 1.94 1.93 1.93 1.92 1.92 1.84 1.83 1.83 1.83 1.82 1.82 1.81 1.81 1.81 1.80 1.80 1.79 1.78 1.78 1.73 1.70 1.70 1.69 1.55 1.54 1.54 1.54 1.53 1.53 1.52 1.52 1.51 1.51 1.50 1.38 1.38 1.36 1.36 1.35 1.34 1.34 1.33 1.33 1.32 1.32 1.31 1.31 1.31 1.30 1.30 1.29 1.28 1.27 1.27 1.26 1.26 1.24 1.23 1.23 1.23 1.22 1.21 1.20 1.12 1.12 1.11 1.09 1.08 1.06
H
O
H
9a
^{1}H NMR
(400 MHz, $CDCl_3$)
1.00 1.03 1.04 1.04 3.22 3.19 2.09 2.01 6.29 2.16
8.0 7.5 7.0 6.5 6.0 5.5 5.0 4.5 4.0 3.5 3.0 2.5 2.0 1.5 1.0 0.5 0.0
f1 (ppm)

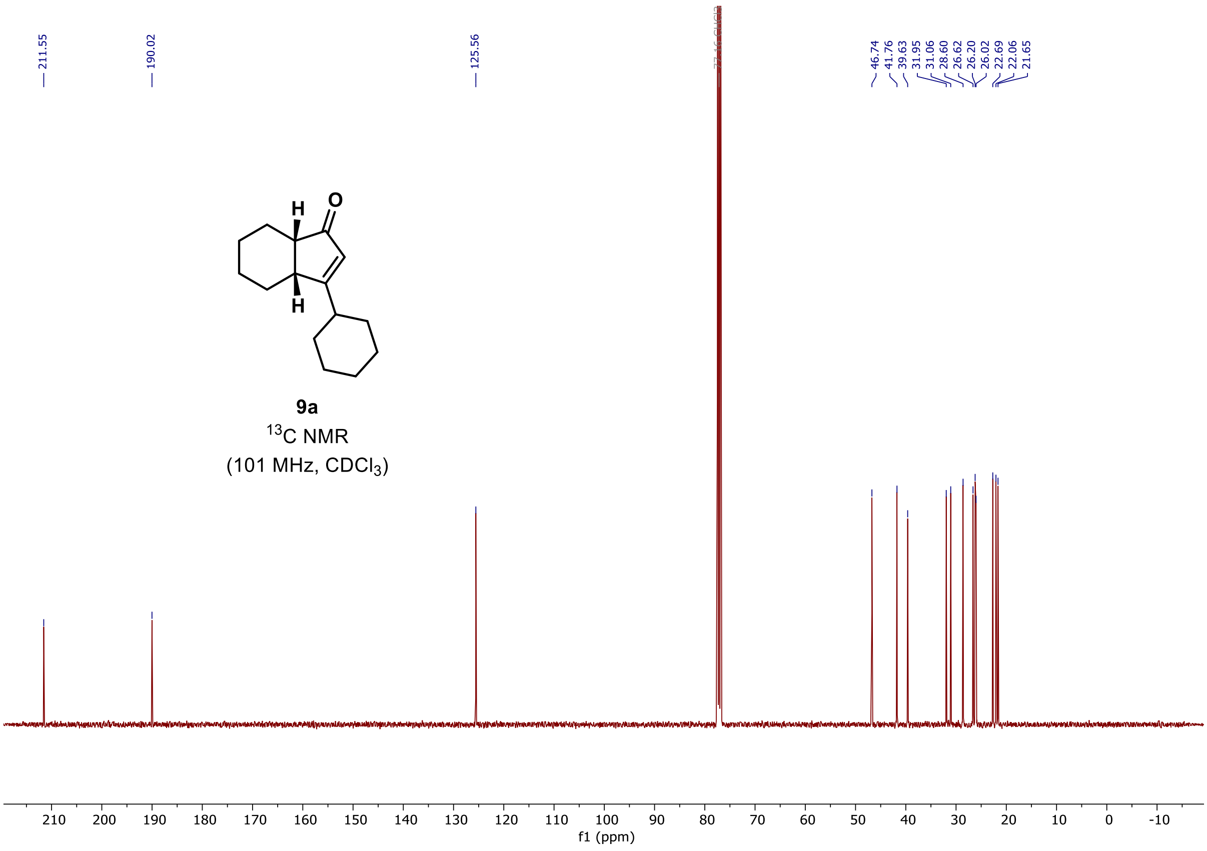
211.55
190.02
125.56
46.74 41.76 39.63 31.95 31.06 28.60 26.62 26.20 26.02 22.69 22.06 21.65
H
O
H
9a
^{13}C NMR
(101 MHz, $CDCl_3$)
210 200 190 180 170 160 150 140 130 120 110 100 90 80 70 60 50 40 30 20 10 0 -10
f1 (ppm)

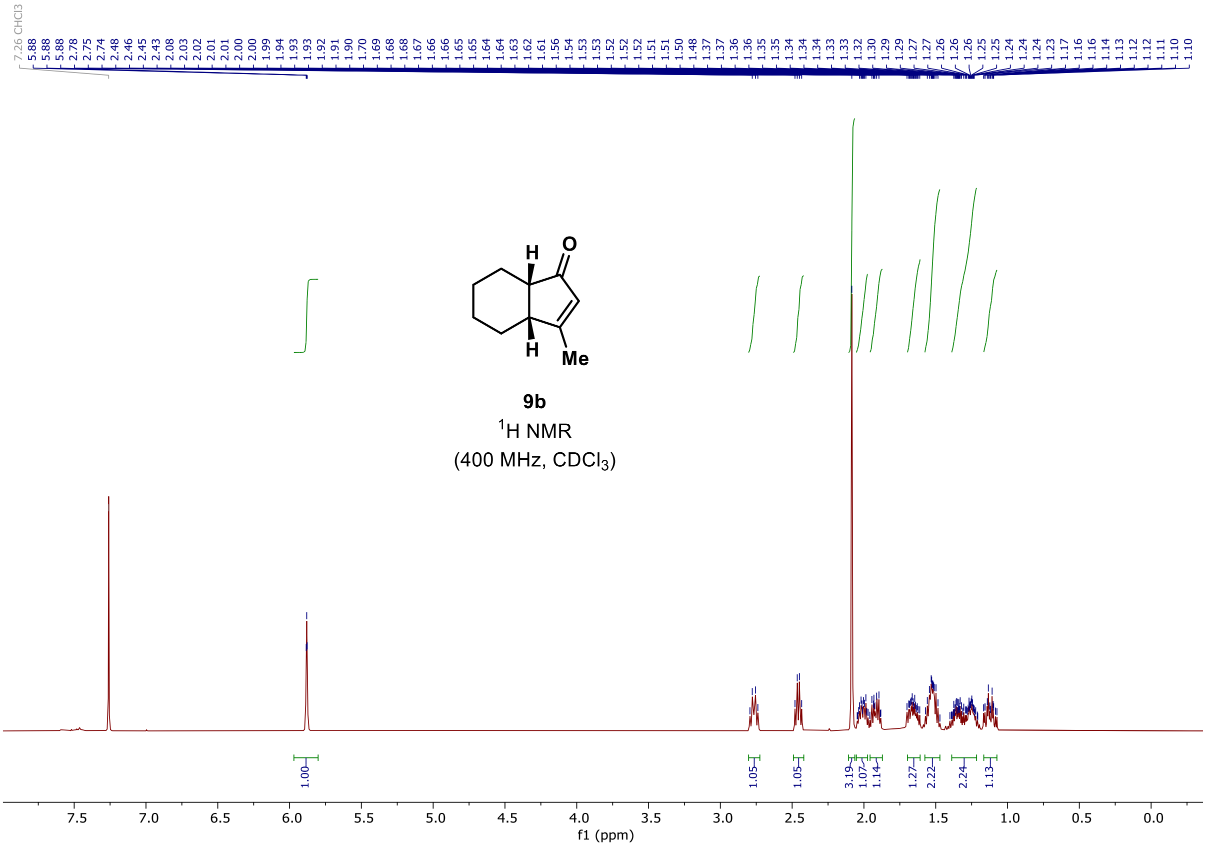
7.26 CHCl3
5.88 5.88 5.88 2.78 2.75 2.74 2.48 2.46 2.45 2.43 2.08 2.03 2.02 2.01 2.01 2.00 2.00 1.99 1.94 1.93 1.93 1.92 1.91 1.90 1.70 1.69 1.68 1.67 1.66 1.66 1.65 1.65 1.64 1.64 1.63 1.62 1.61 1.56 1.54 1.53 1.53 1.52 1.52 1.52 1.51 1.51 1.50 1.48 1.37 1.37 1.36 1.36 1.35 1.35 1.34 1.34 1.34 1.33 1.33 1.32 1.30 1.29 1.29 1.27 1.27 1.26 1.26 1.26 1.25 1.25 1.24 1.24 1.23 1.17 1.16 1.14 1.13 1.12 1.12 1.11 1.10 1.10
H
O
H
Me
9b
¹H NMR
(400 MHz, CDCl3)
1.00
1.05
1.05
3.19
1.07
1.14
1.27
2.22
2.24
1.13
7.5 7.0 6.5 6.0 5.5 5.0 4.5 4.0 3.5 3.0 2.5 2.0 1.5 1.0 0.5 0.0
f1 (ppm)

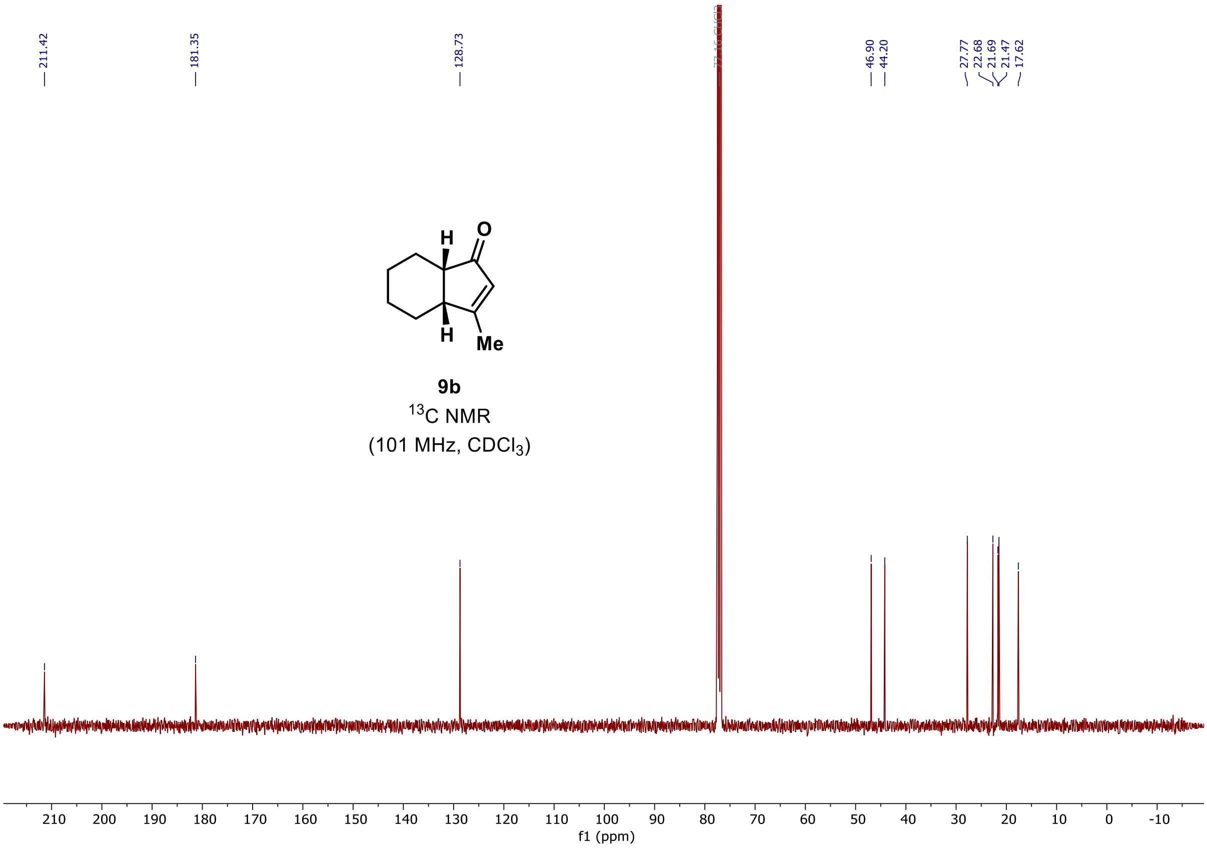
211.42
181.35
128.73
46.90
44.20
27.77
22.68
21.69
21.47
17.62
H
O
H
Me
9b
¹³C NMR
(101 MHz, CDCl3)
210 200 190 180 170 160 150 140 130 120 110 100 90 80 70 60 50 40 30 20 10 0 -10
f1 (ppm)

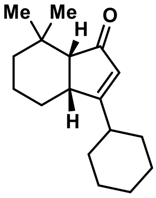

9c
^{1}H NMR
(400 MHz, $CDCl_3$)

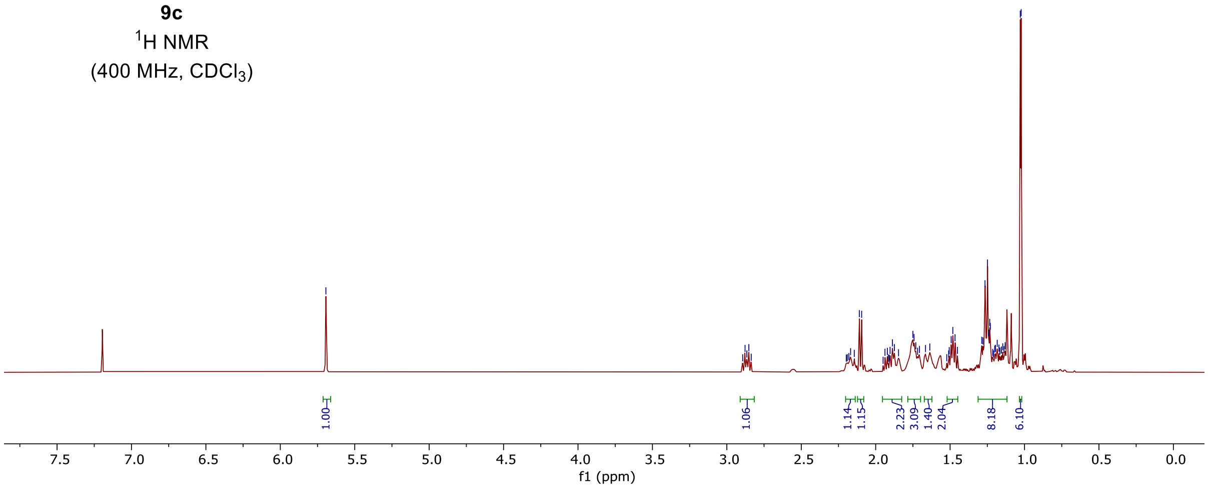

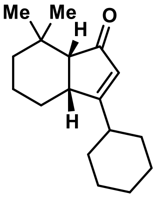

9c
^{13}C NMR
(101 MHz, $CDCl_3$)

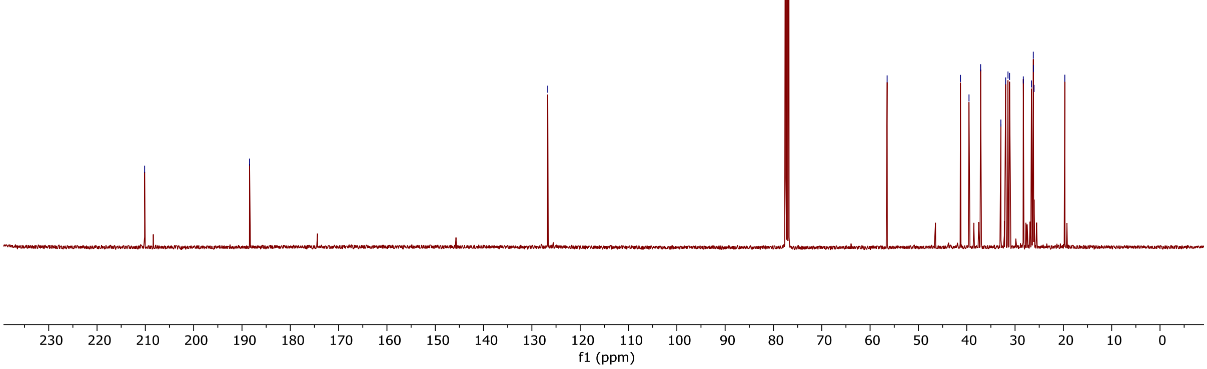

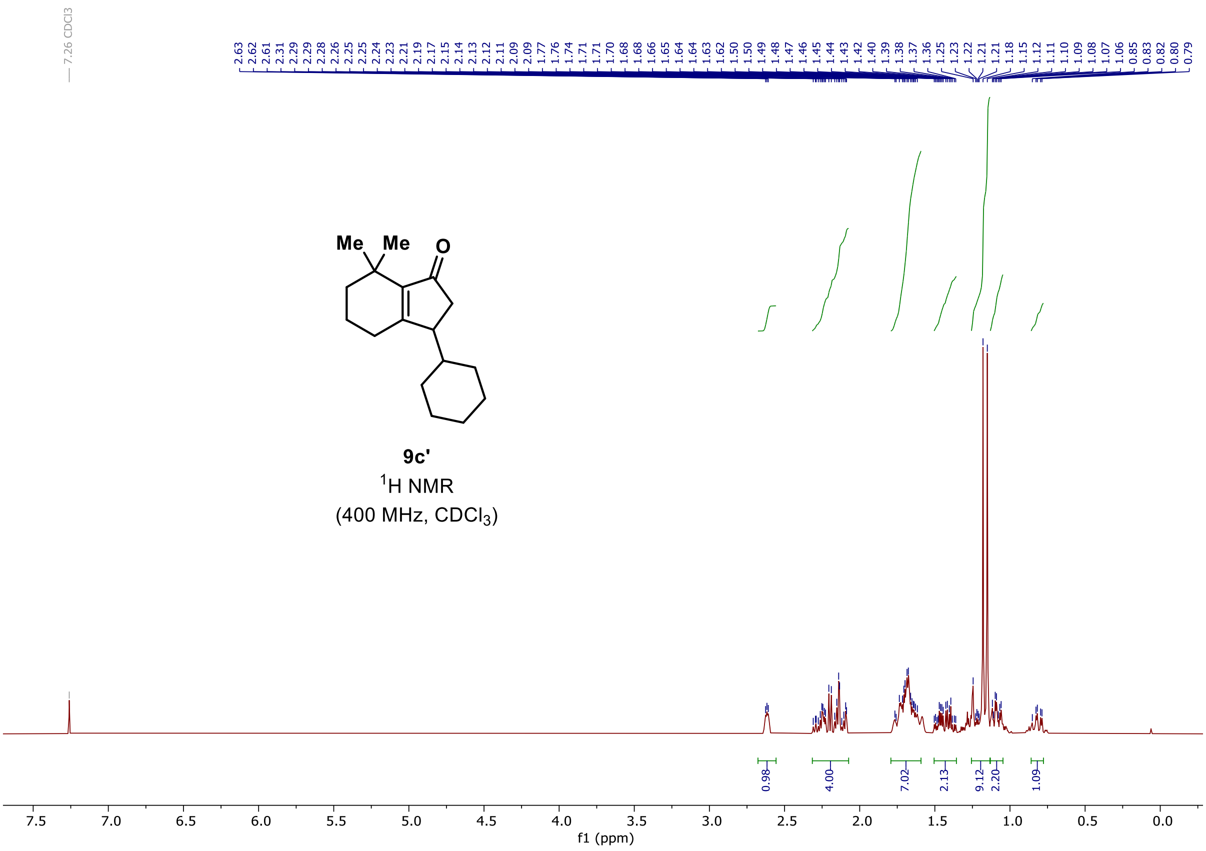
9c'
1H NMR
(400 MHz, CDCl3)
f1 (ppm)

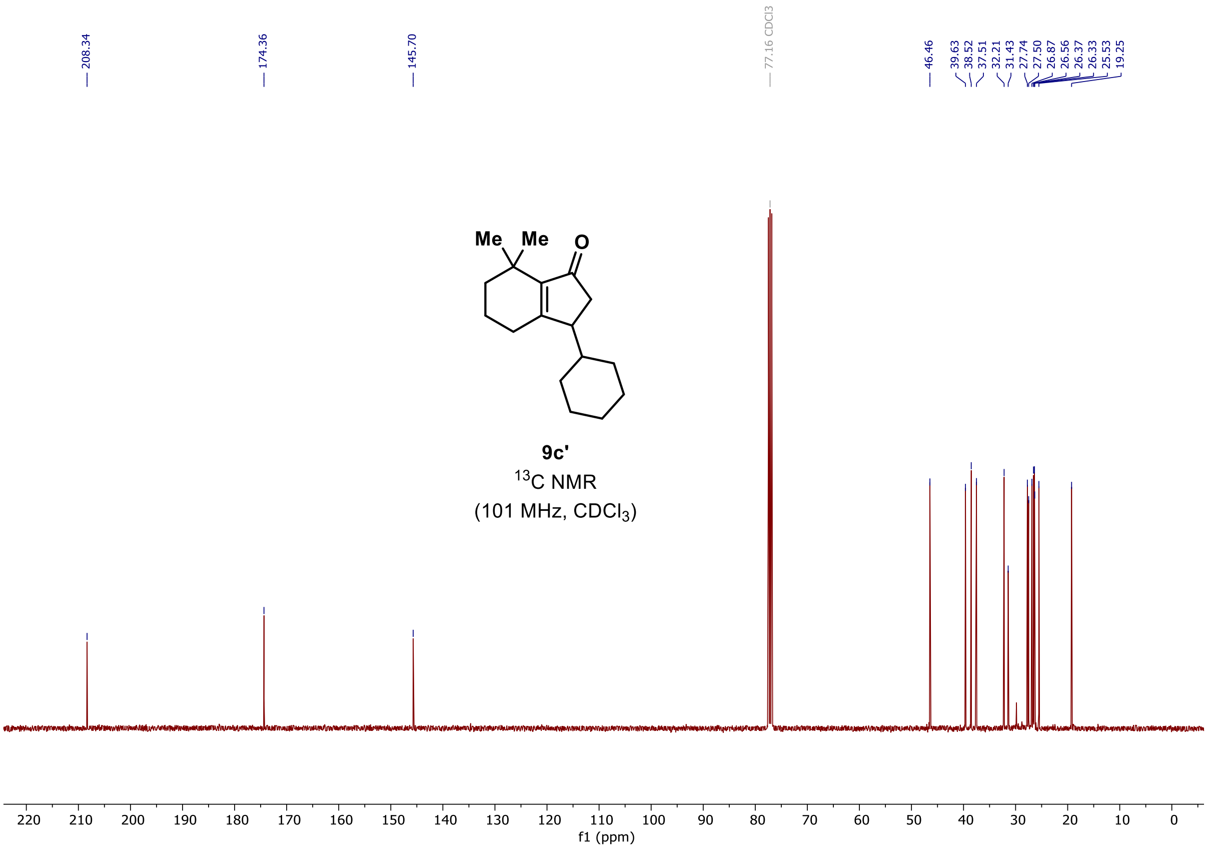
9c'
13C NMR
(101 MHz, CDCl3)
f1 (ppm)

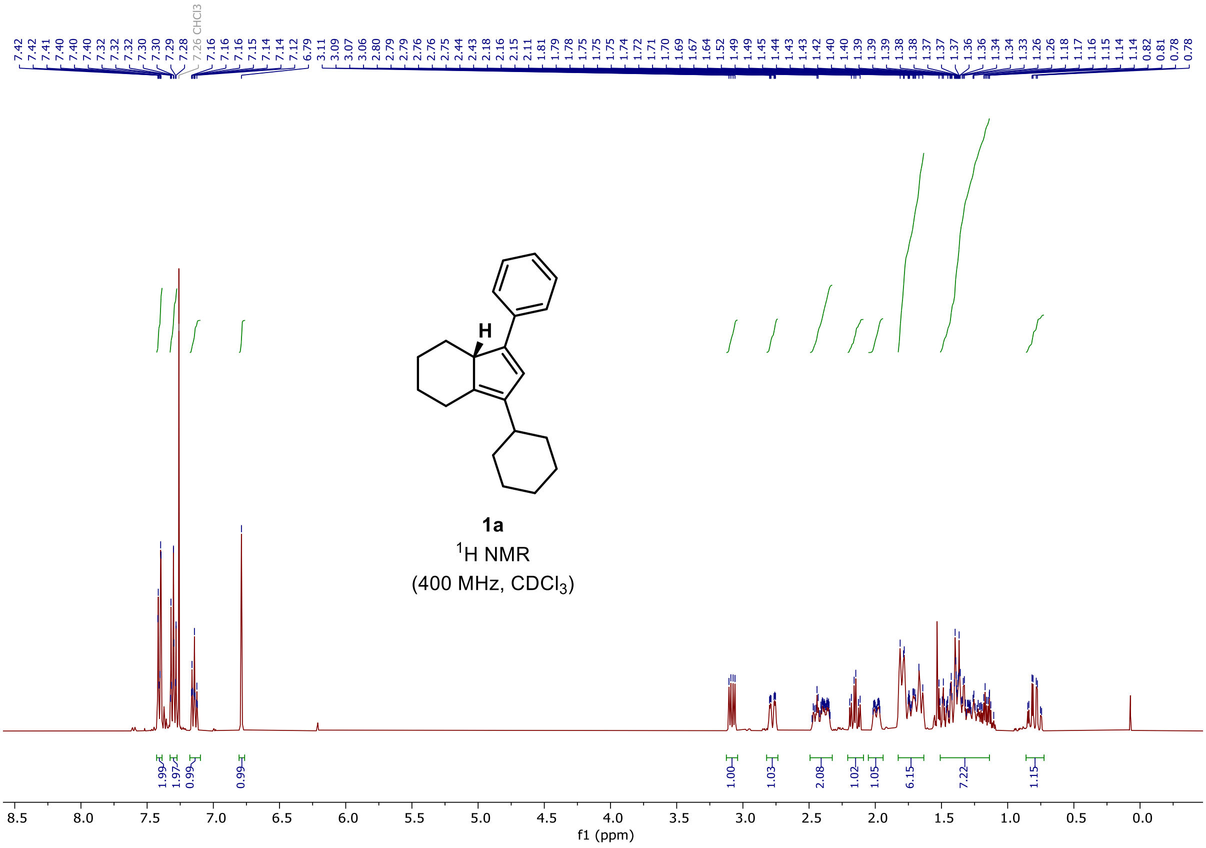

1a
^{1}H NMR
(400 MHz, $CDCl_3$)
f1 (ppm)

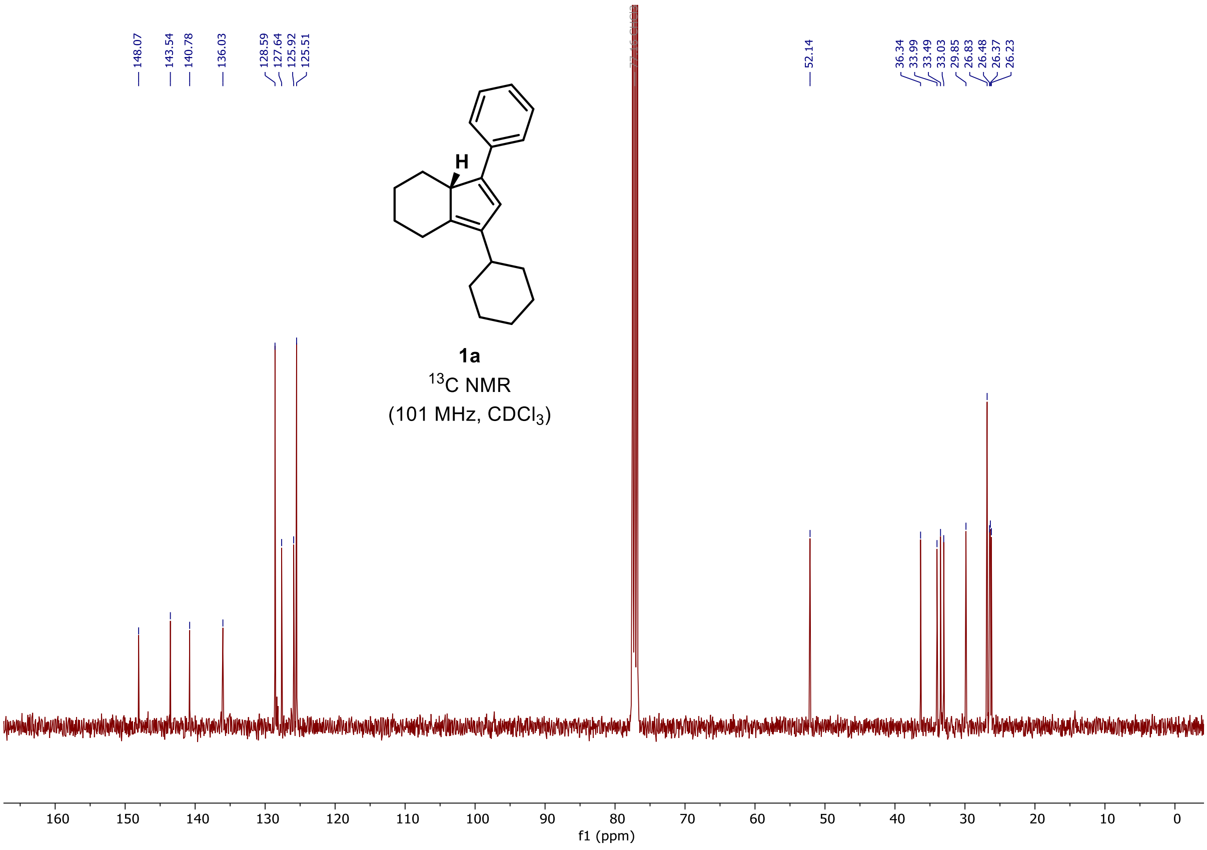

1a
^{13}C NMR
(101 MHz, $CDCl_3$)
f1 (ppm)

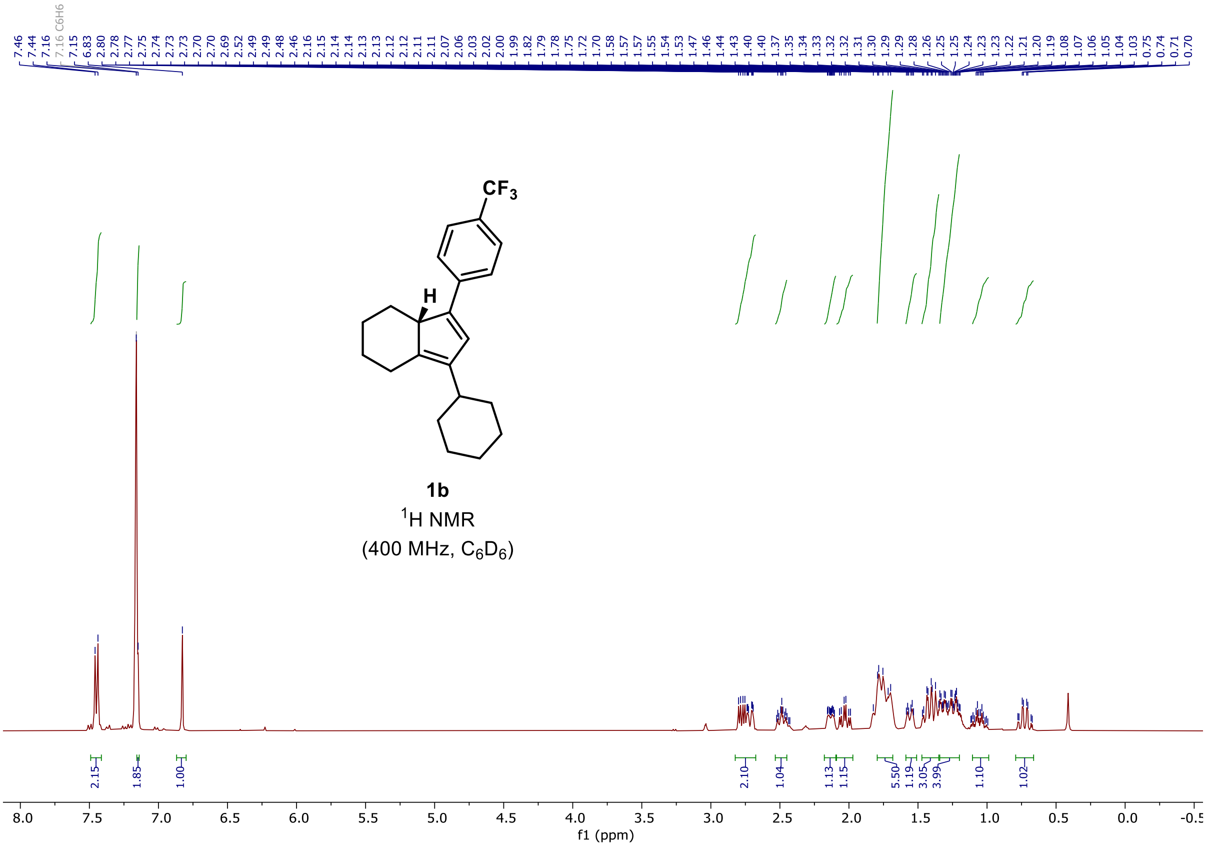
7.46
7.44
7.16 C6H6
7.15
6.83
2.80
2.78
2.77
2.75
2.74
2.73
2.73
2.70
2.70
2.69
2.52
2.49
2.49
2.48
2.46
2.16
2.15
2.14
2.14
2.13
2.13
2.12
2.12
2.11
2.11
2.07
2.06
2.03
2.02
2.00
1.99
1.82
1.79
1.78
1.75
1.72
1.70
1.58
1.57
1.57
1.55
1.54
1.53
1.47
1.46
1.44
1.43
1.40
1.40
1.37
1.35
1.34
1.33
1.32
1.32
1.31
1.30
1.29
1.29
1.28
1.26
1.25
1.25
1.24
1.23
1.23
1.22
1.21
1.20
1.19
1.08
1.07
1.06
1.05
1.04
1.03
0.75
0.74
0.71
0.70
CF3
H
1b
1H NMR
(400 MHz, C6D6)
2.15
1.85
1.00
2.10
1.04
1.13
1.15
5.50
1.19
3.05
3.99
1.10
1.02
8.0 7.5 7.0 6.5 6.0 5.5 5.0 4.5 4.0 3.5 3.0 2.5 2.0 1.5 1.0 0.5 0.0
f1 (ppm)

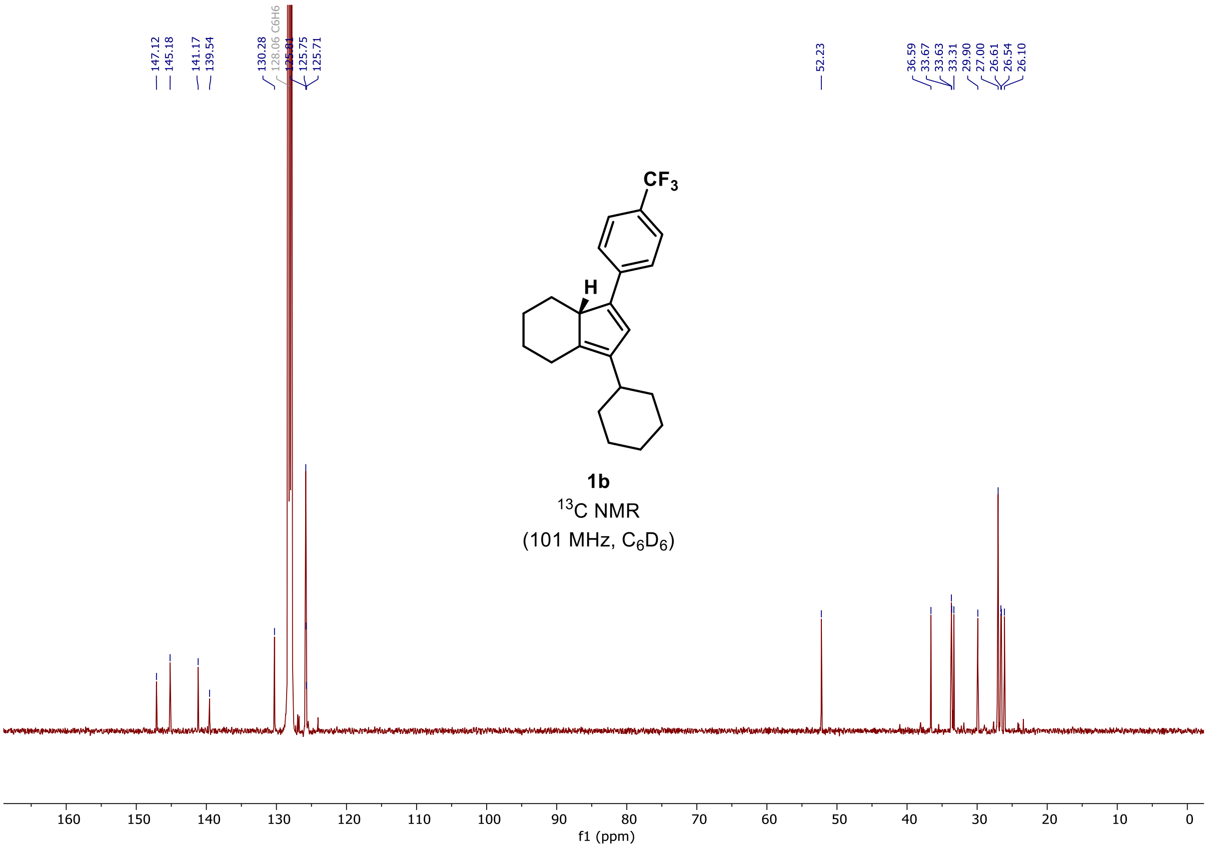
147.12
145.18
141.17
139.54
130.28
128.06 C6H6
125.81
125.75
125.71
52.23
36.59
33.67
33.63
33.31
29.90
27.00
26.61
26.54
26.10
CF3
H
1b
13C NMR
(101 MHz, C6D6)
160 150 140 130 120 110 100 90 80 70 60 50 40 30 20 10 0
f1 (ppm)

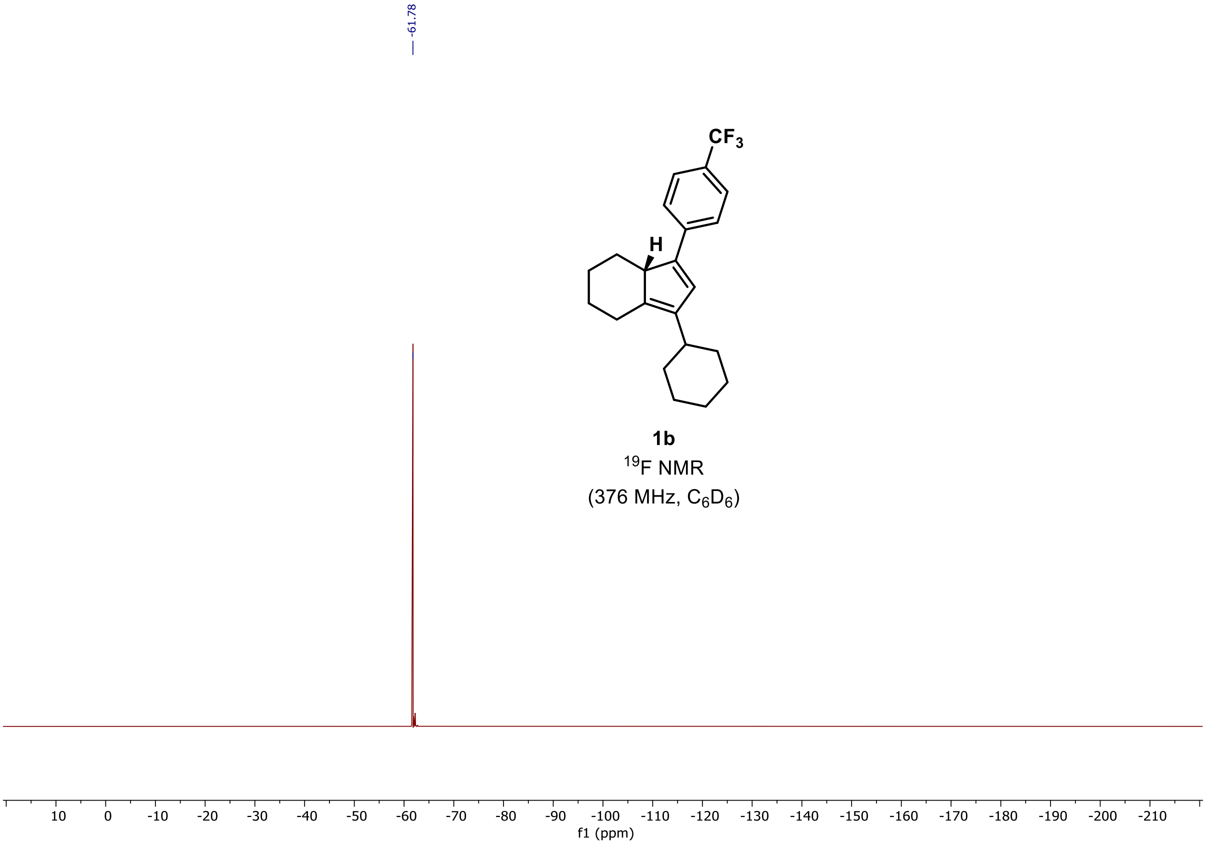

-61.78
CF3
H
1b
19F NMR
(376 MHz, C6D6)
10 0 -10 -20 -30 -40 -50 -60 -70 -80 -90 -100 -110 -120 -130 -140 -150 -160 -170 -180 -190 -200 -210
f1 (ppm)

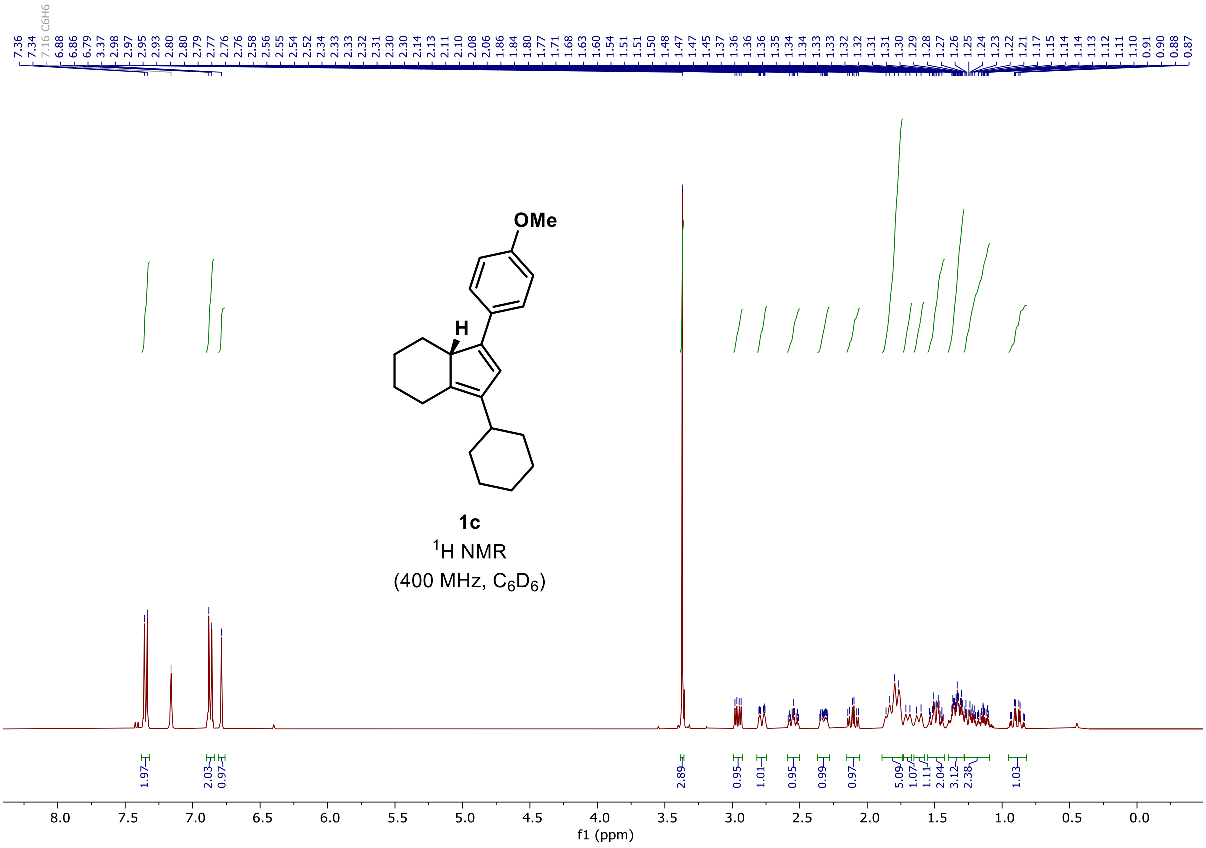
OMe
H
1c
^{1}H NMR
(400 MHz, C_6D_6)
f1 (ppm)

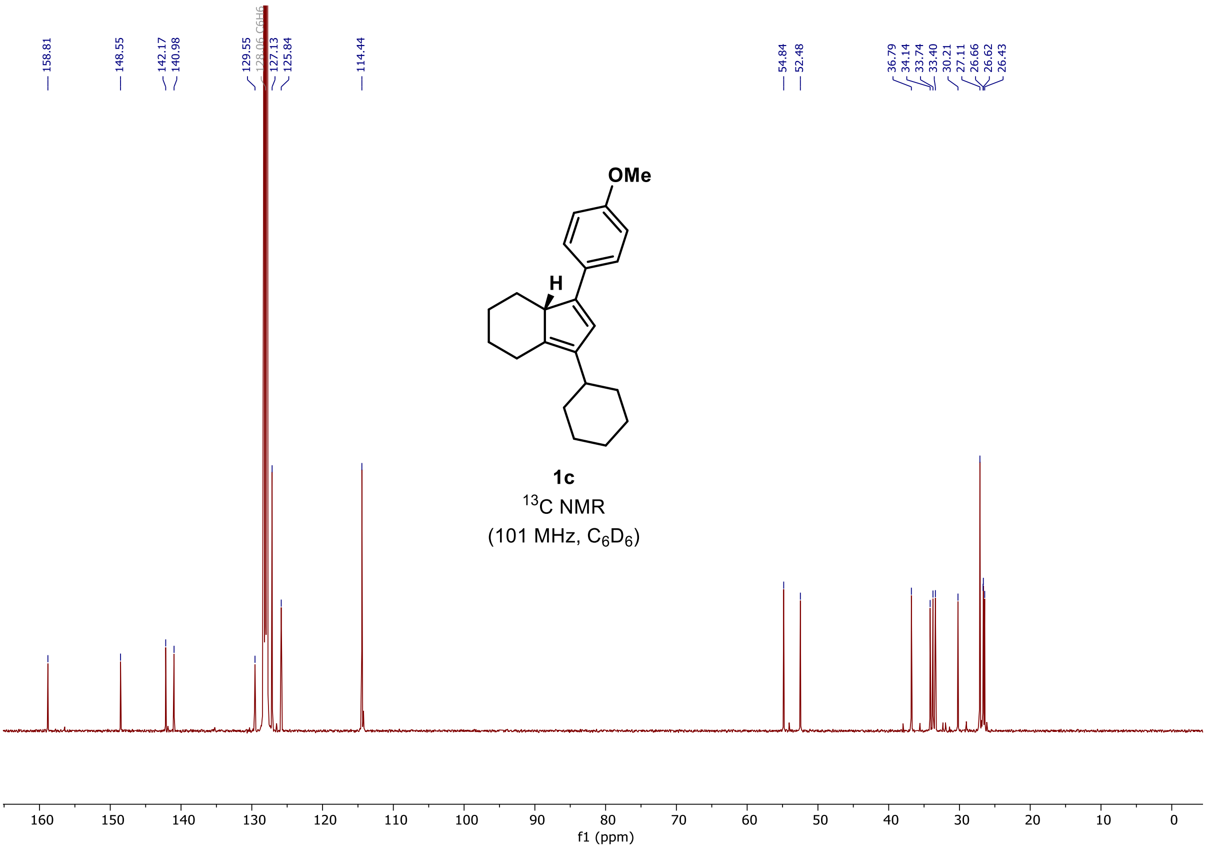
158.81
148.55
142.17
140.98
129.55
127.13
125.84
114.44
54.84
52.48
36.79
34.14
33.74
33.40
30.21
27.11
26.66
26.62
26.43
OMe
H
1c
^{13}C NMR
(101 MHz, C_6D_6)
f1 (ppm)

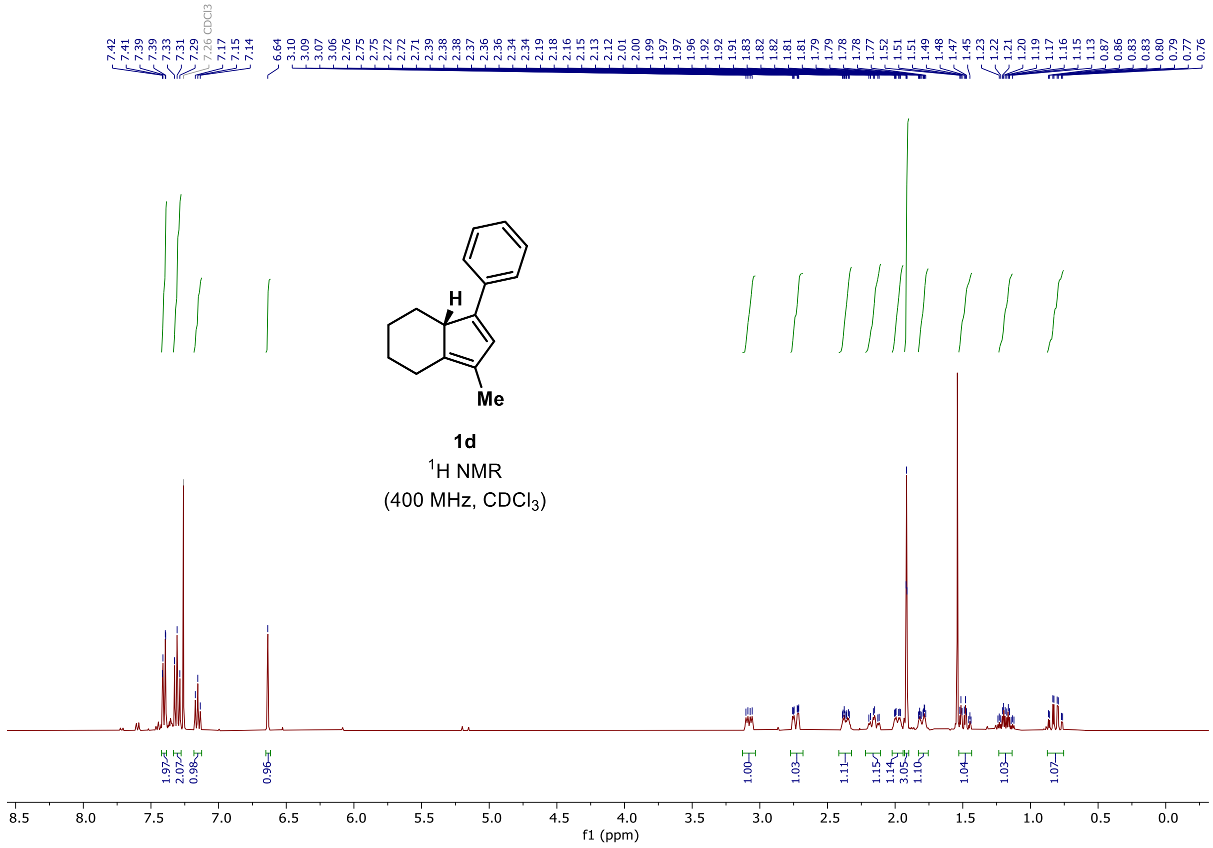
1d
^{1}H NMR
(400 MHz, CDCl$_3$)
Me
H
f1 (ppm)

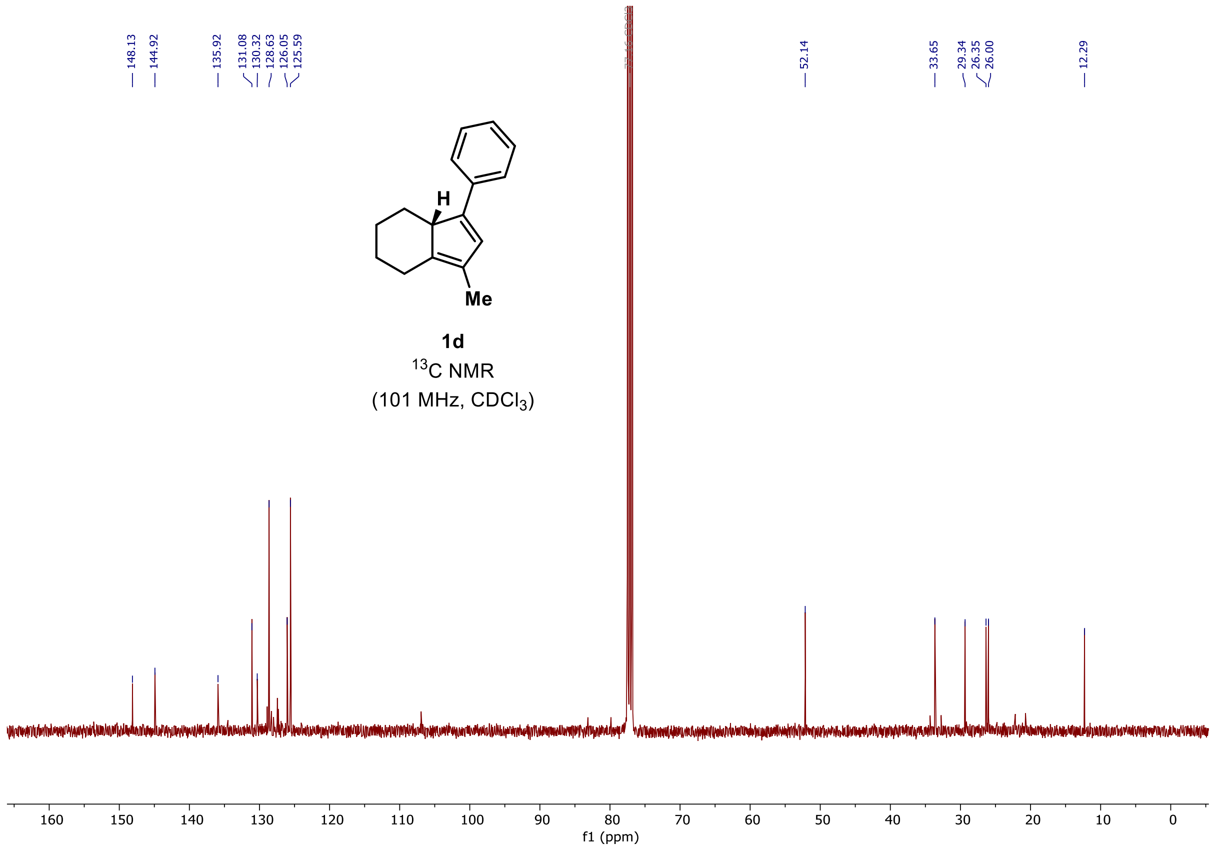
1d
^{13}C NMR
(101 MHz, CDCl$_3$)
Me
H
f1 (ppm)

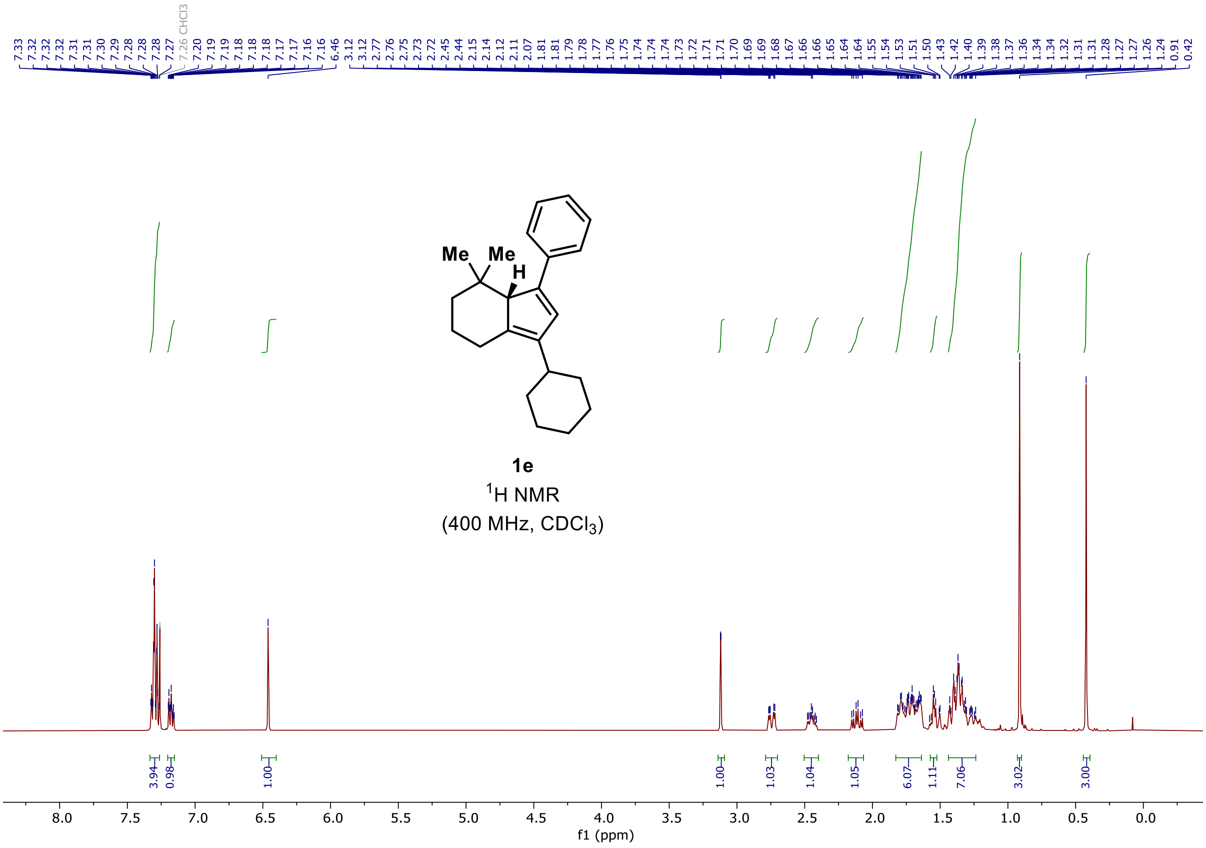
7.33 7.32 7.32 7.32 7.31 7.31 7.30 7.29 7.28 7.28 7.28 7.27 7.26 CHCl3 7.20 7.19 7.19 7.18 7.18 7.18 7.17 7.17 7.16 6.46 3.12 3.12 2.77 2.76 2.75 2.73 2.72 2.45 2.44 2.15 2.14 2.12 2.11 2.07 1.81 1.81 1.79 1.78 1.77 1.76 1.75 1.74 1.74 1.74 1.73 1.72 1.71 1.71 1.70 1.69 1.69 1.68 1.67 1.66 1.66 1.65 1.64 1.64 1.55 1.54 1.53 1.51 1.50 1.43 1.42 1.40 1.39 1.38 1.37 1.36 1.34 1.32 1.31 1.31 1.28 1.27 1.26 1.24 0.91 0.42
Me
Me
H
1e
1H NMR
(400 MHz, CDCl3)
3.94 0.98 1.00 1.00 1.03 1.04 1.05 6.07 1.11 7.06 3.02 3.00
8.0 7.5 7.0 6.5 6.0 5.5 5.0 4.5 4.0 3.5 3.0 2.5 2.0 1.5 1.0 0.5 0.0
f1 (ppm)

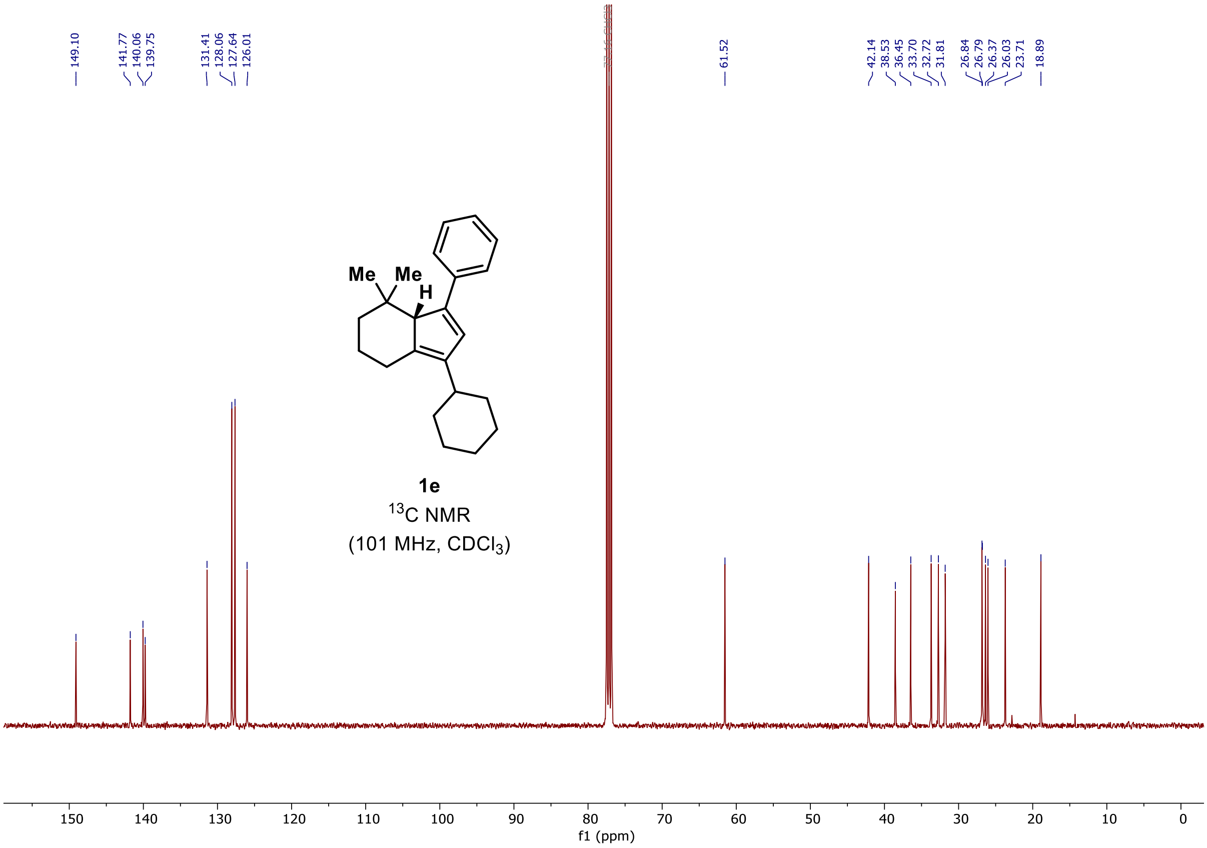
149.10 141.77 140.06 139.75 131.41 128.06 127.64 126.01 61.52 42.14 38.53 36.45 33.70 32.72 31.81 26.84 26.79 26.37 26.03 23.71 18.89
Me
Me
H
1e
13C NMR
(101 MHz, CDCl3)
150 140 130 120 110 100 90 80 70 60 50 40 30 20 10 0
f1 (ppm)

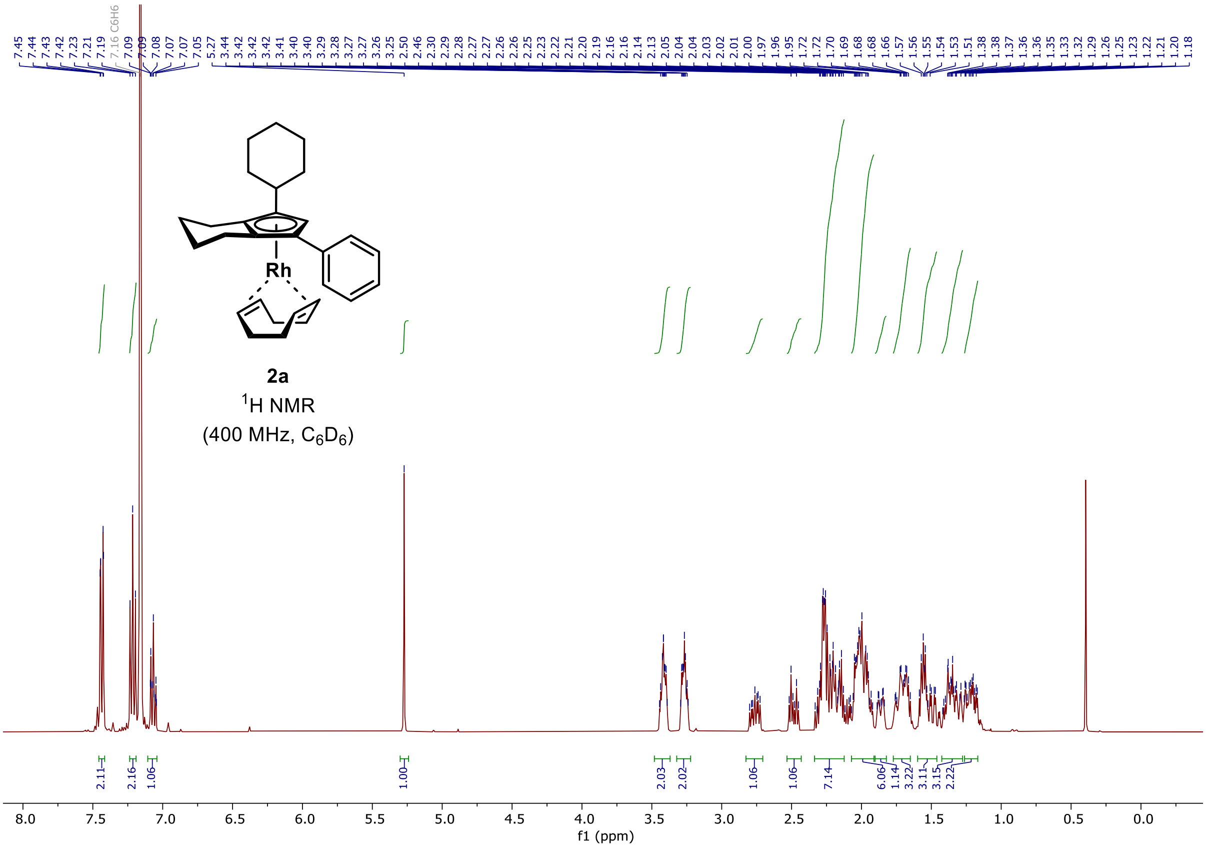

2a

^{1}H NMR

(400 MHz, C_6D_6)

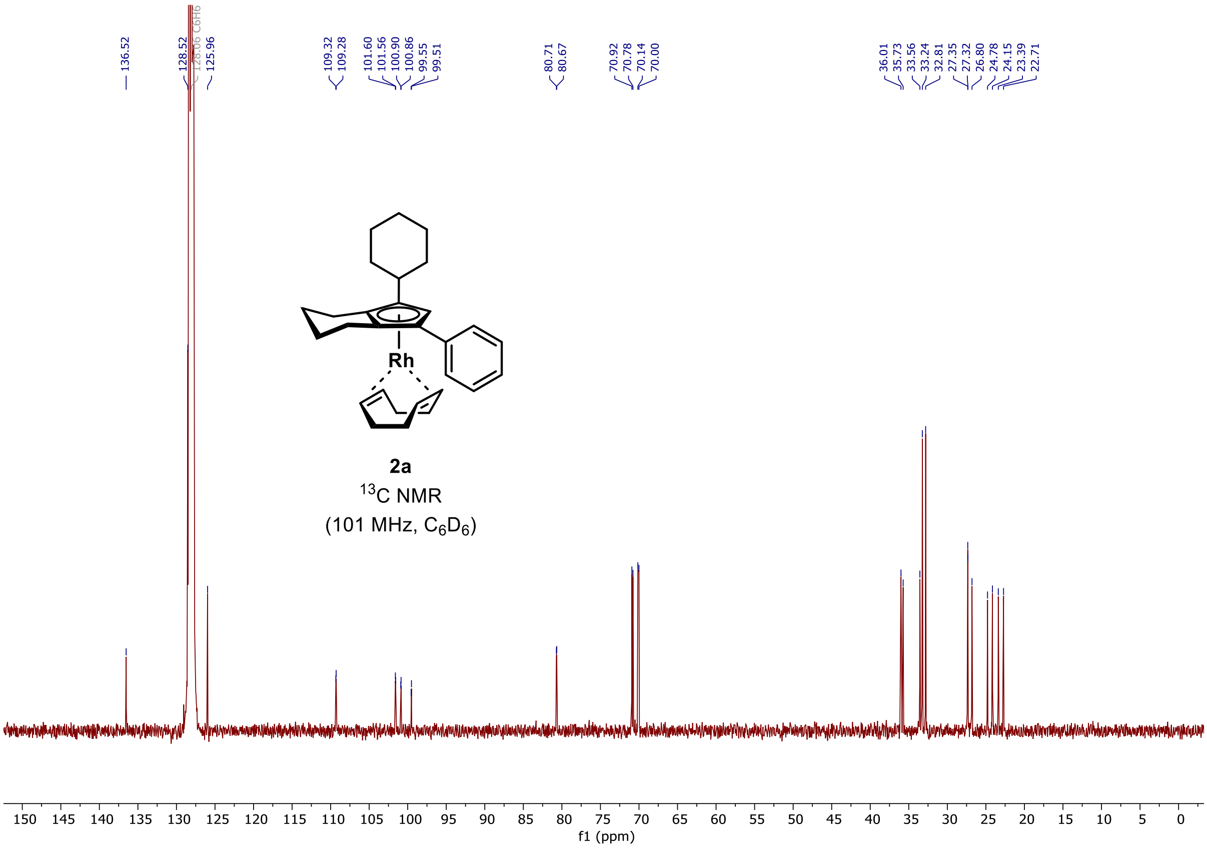

2a

^{13}C NMR

(101 MHz, C_6D_6)

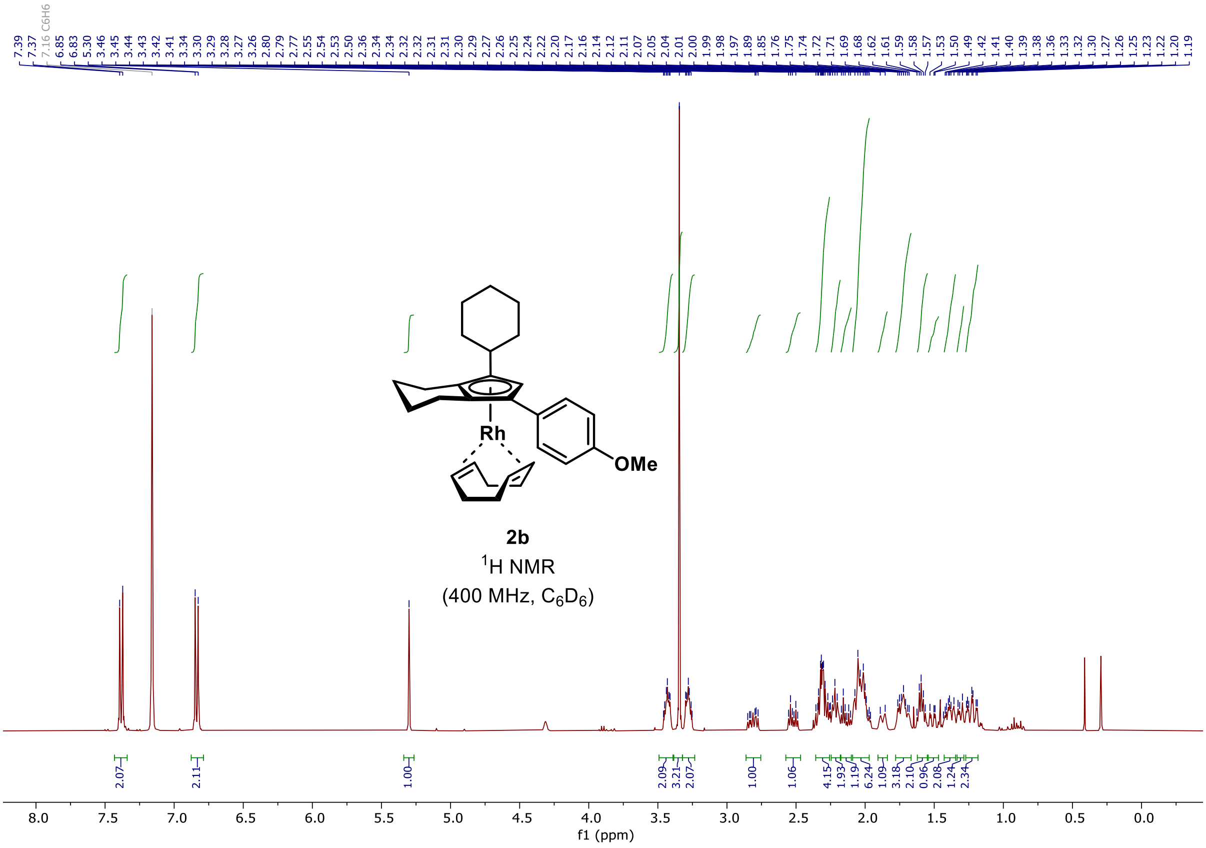
Rh
OMe
2b
^{1}H NMR
(400 MHz, C_6D_6)
f1 (ppm)

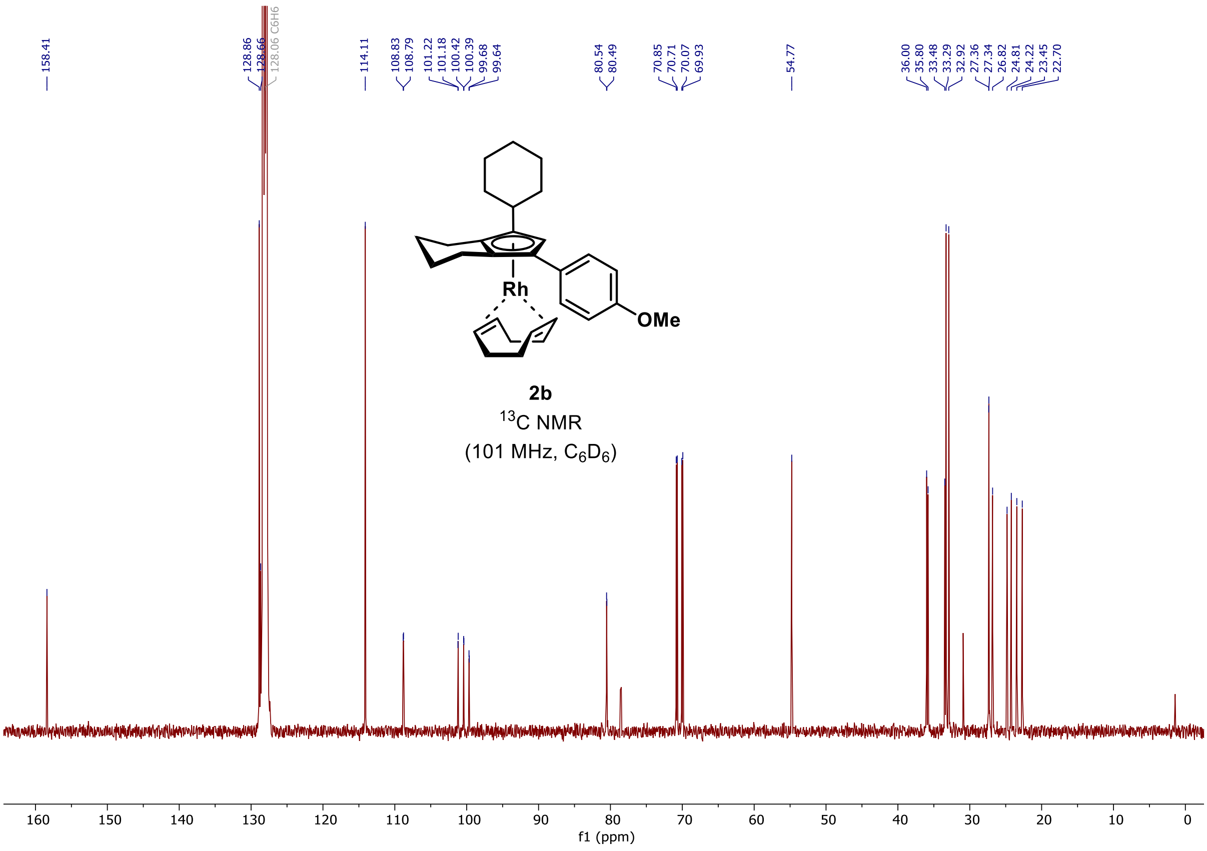
Rh
OMe
2b
^{13}C NMR
(101 MHz, C_6D_6)
f1 (ppm)

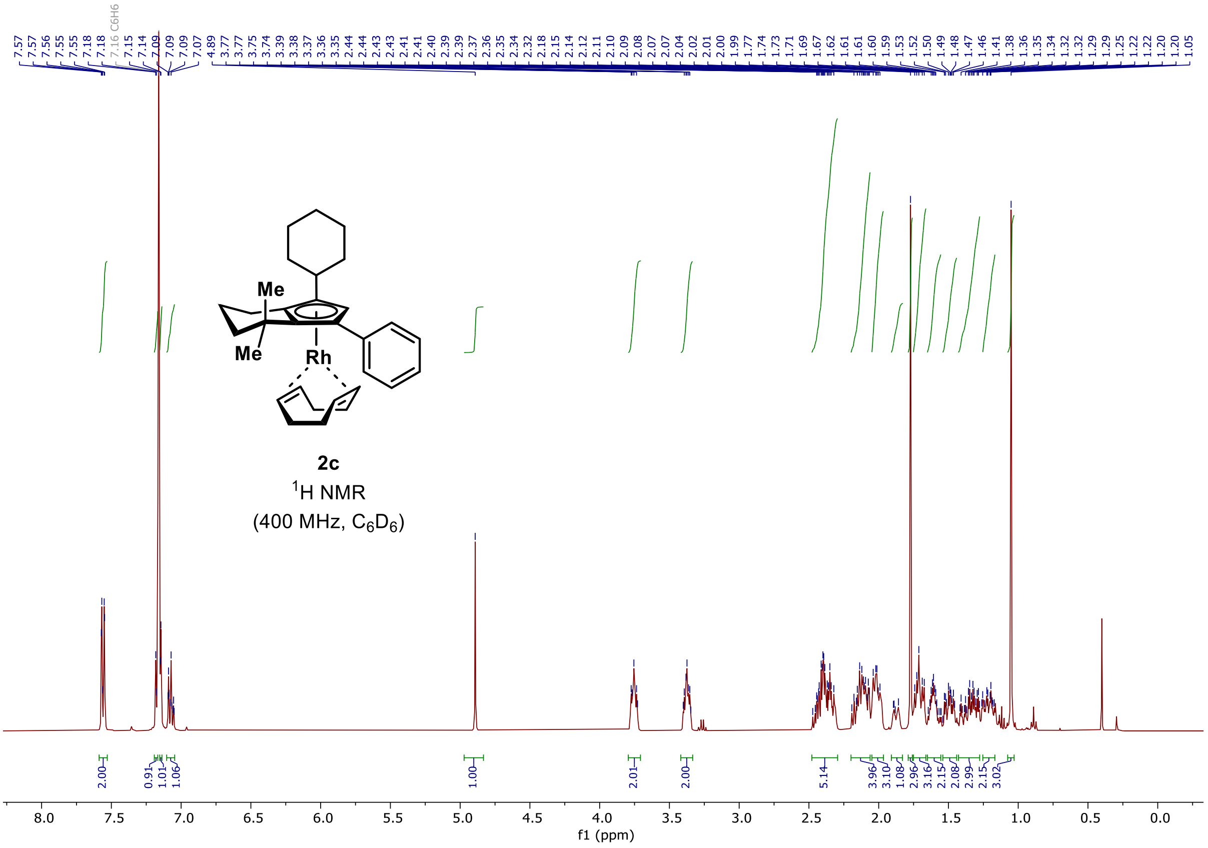

Me
Me
Rh
2c
^{1}H NMR
(400 MHz, C_6D_6)
f1 (ppm)

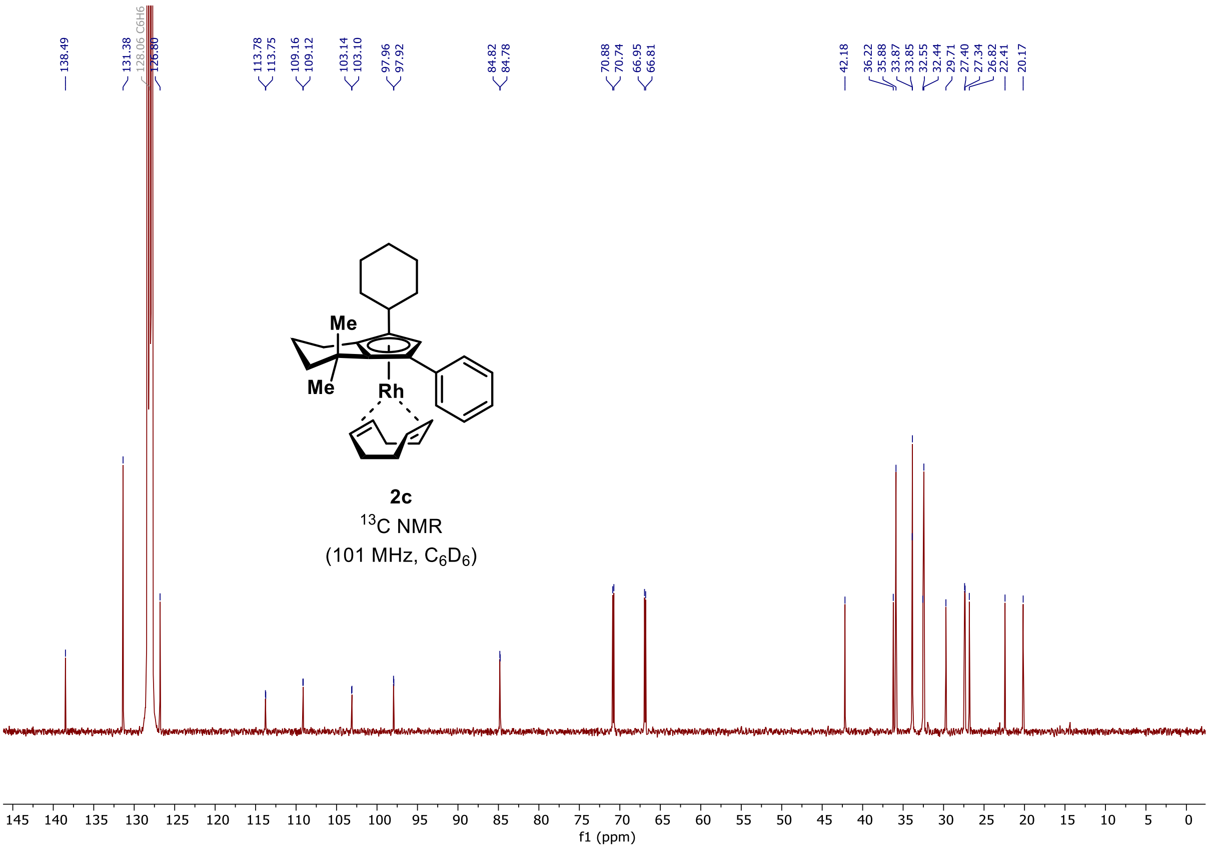

Me
Me
Rh
2c
^{13}C NMR
(101 MHz, C_6D_6)
f1 (ppm)

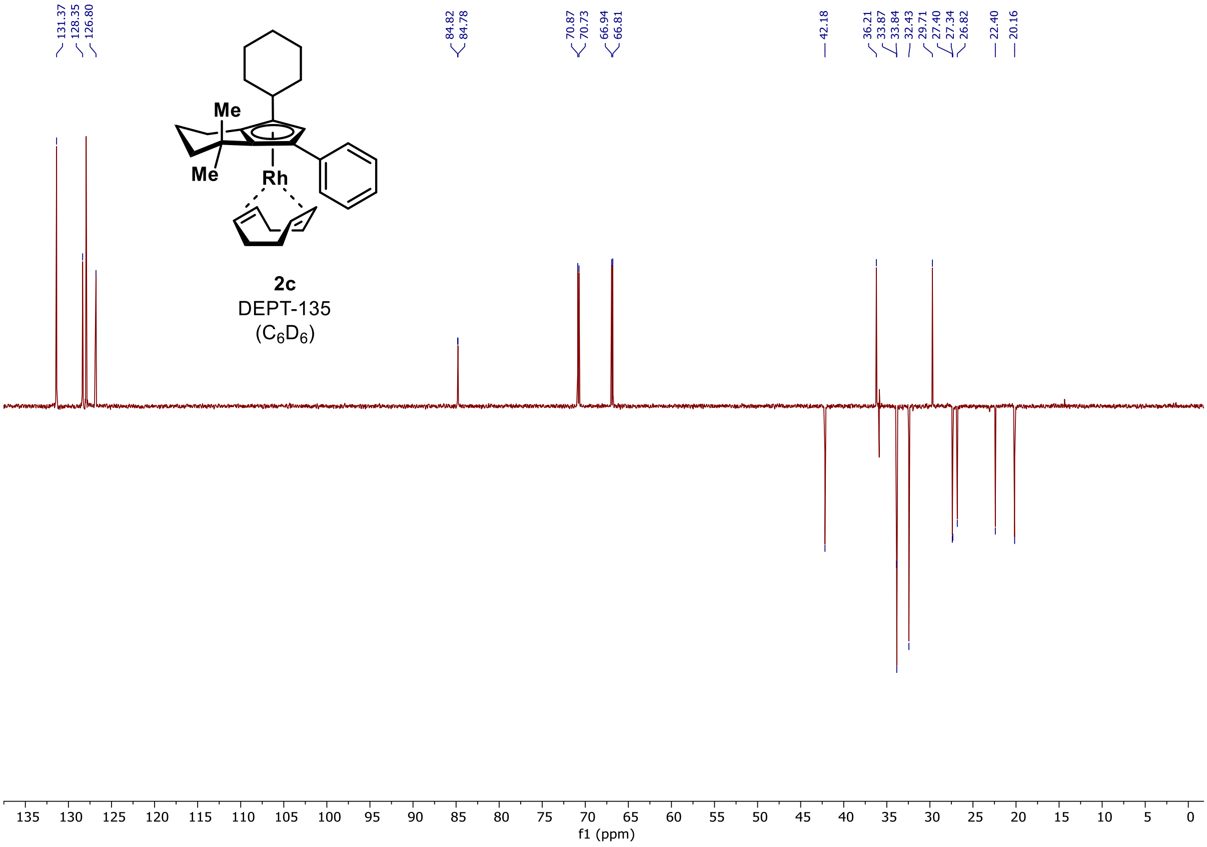

131.37
128.35
126.80
84.82
84.78
70.87
70.73
66.94
66.81
42.18
36.21
33.87
33.84
32.43
29.71
27.40
27.34
26.82
22.40
20.16
Me
Me
Rh
2c
DEPT-135
(C_6D_6)
135 130 125 120 115 110 105 100 95 90 85 80 75 70 65 60 55 50 45 40 35 30 25 20 15 10 5 0
f1 (ppm)

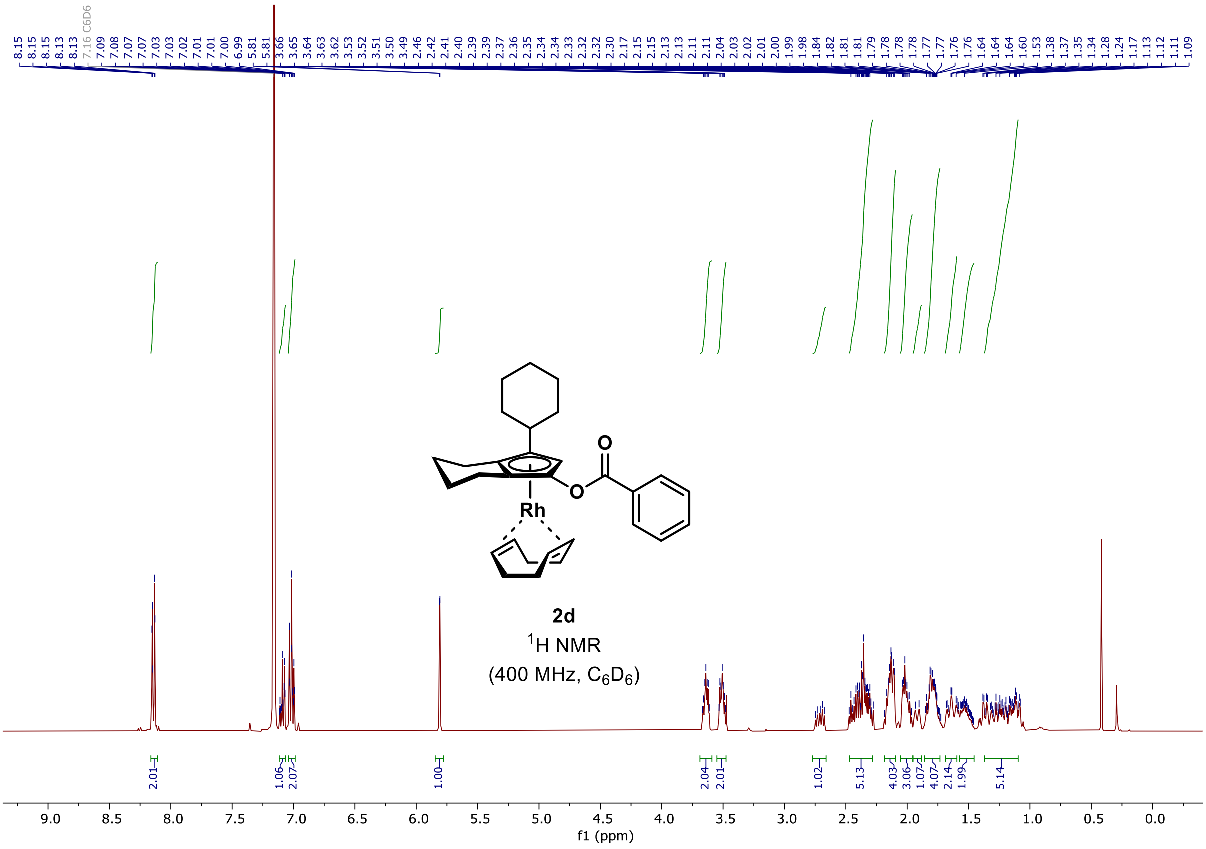
8.15
8.15
8.15
8.13
7.16 C6D6
7.09
7.08
7.07
7.07
7.03
7.03
7.02
7.01
7.01
7.00
6.99
5.81
5.81
3.66
3.65
3.64
3.63
3.62
3.53
3.52
3.51
3.50
3.49
2.46
2.42
2.41
2.40
2.39
2.39
2.37
2.36
2.35
2.34
2.34
2.33
2.32
2.32
2.30
2.17
2.15
2.15
2.13
2.13
2.11
2.11
2.04
2.03
2.02
2.01
2.00
1.99
1.98
1.84
1.82
1.81
1.81
1.79
1.78
1.78
1.78
1.77
1.77
1.76
1.76
1.64
1.64
1.60
1.53
1.38
1.37
1.35
1.34
1.28
1.24
1.17
1.13
1.12
1.11
1.09
O
Rh
O
2d
1H NMR
(400 MHz, C6D6)
2.01
1.06
2.07
1.00
2.04
2.01
1.02
5.13
4.03
3.06
1.07
4.07
2.14
1.99
5.14
9.0
8.5
8.0
7.5
7.0
6.5
6.0
5.5
5.0
4.5
4.0
3.5
3.0
2.5
2.0
1.5
1.0
0.5
0.0
f1 (ppm)

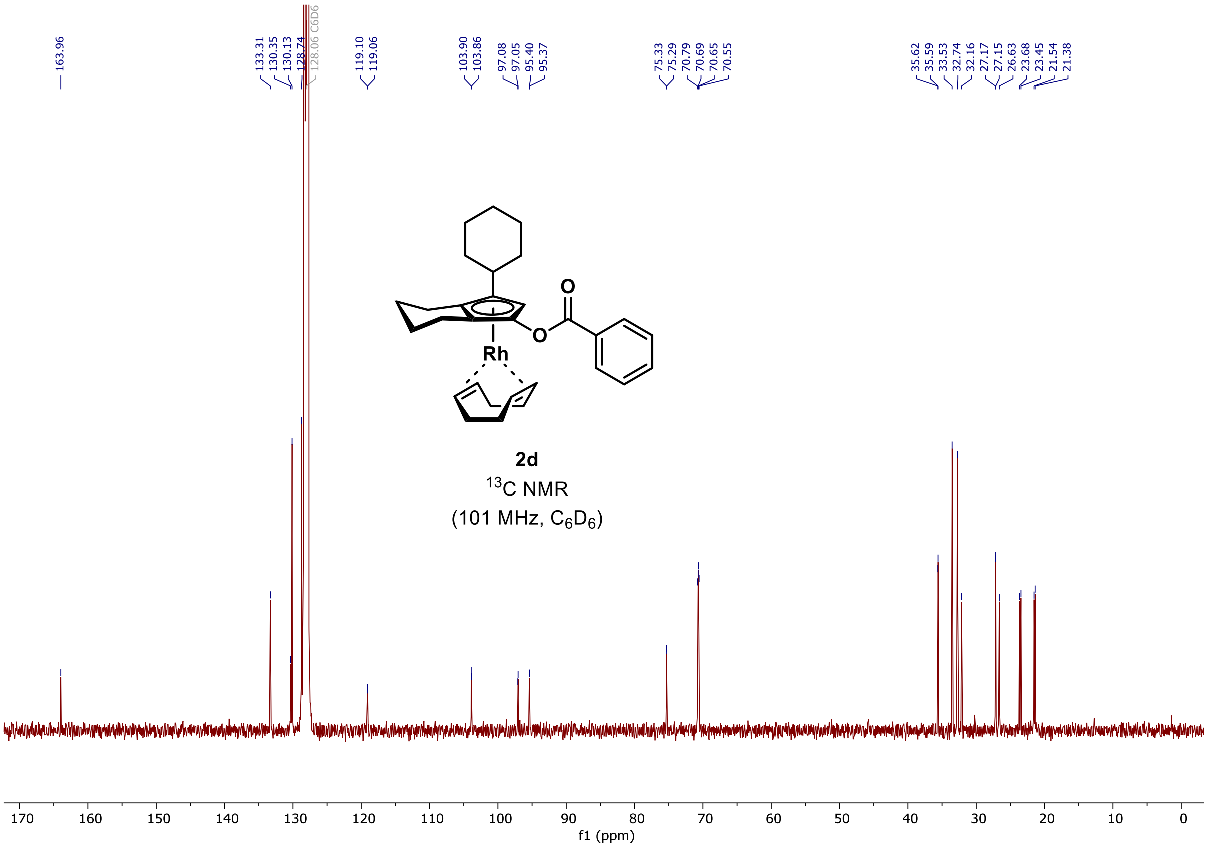
163.96
133.31
130.35
130.13
128.74
128.06 C6D6
119.10
119.06
103.90
103.86
97.08
97.05
95.40
95.37
75.33
75.29
70.79
70.69
70.65
70.55
35.62
35.59
33.53
32.74
32.16
27.17
27.15
26.63
23.68
23.45
21.54
21.38
O
Rh
O
2d
13C NMR
(101 MHz, C6D6)
170
160
150
140
130
120
110
100
90
80
70
60
50
40
30
20
10
0
f1 (ppm)

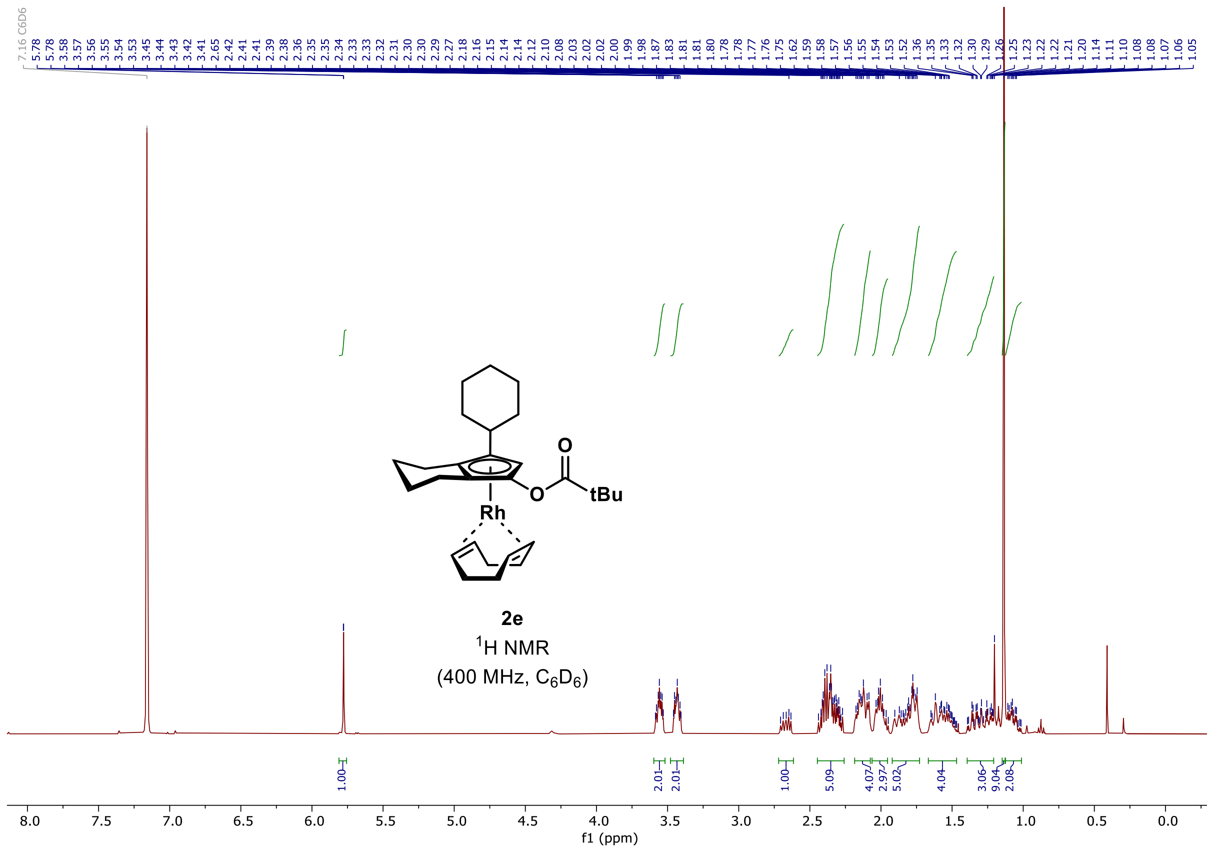
O
tBu
Rh
2e
^{1}H NMR
(400 MHz, C_6D_6)
f1 (ppm)

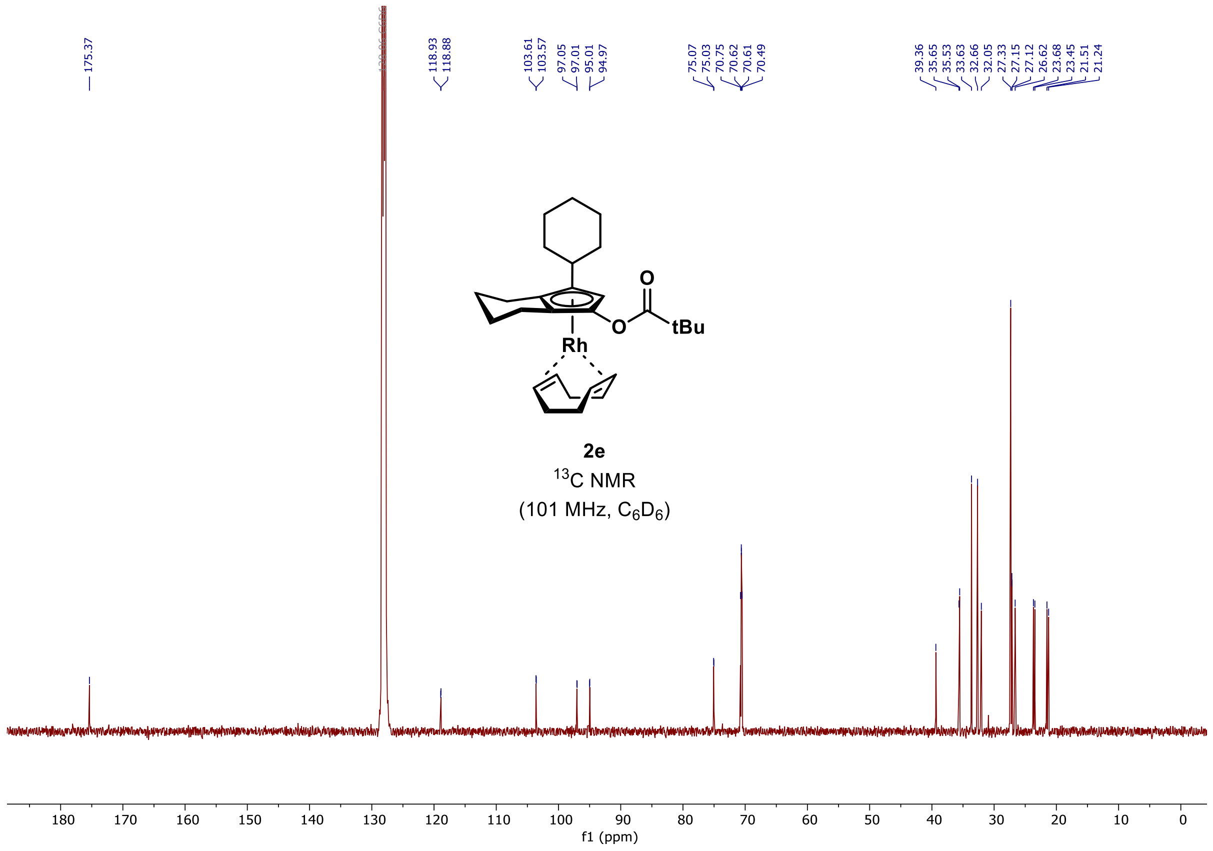
175.37
O
tBu
Rh
2e
^{13}C NMR
(101 MHz, C_6D_6)
f1 (ppm)

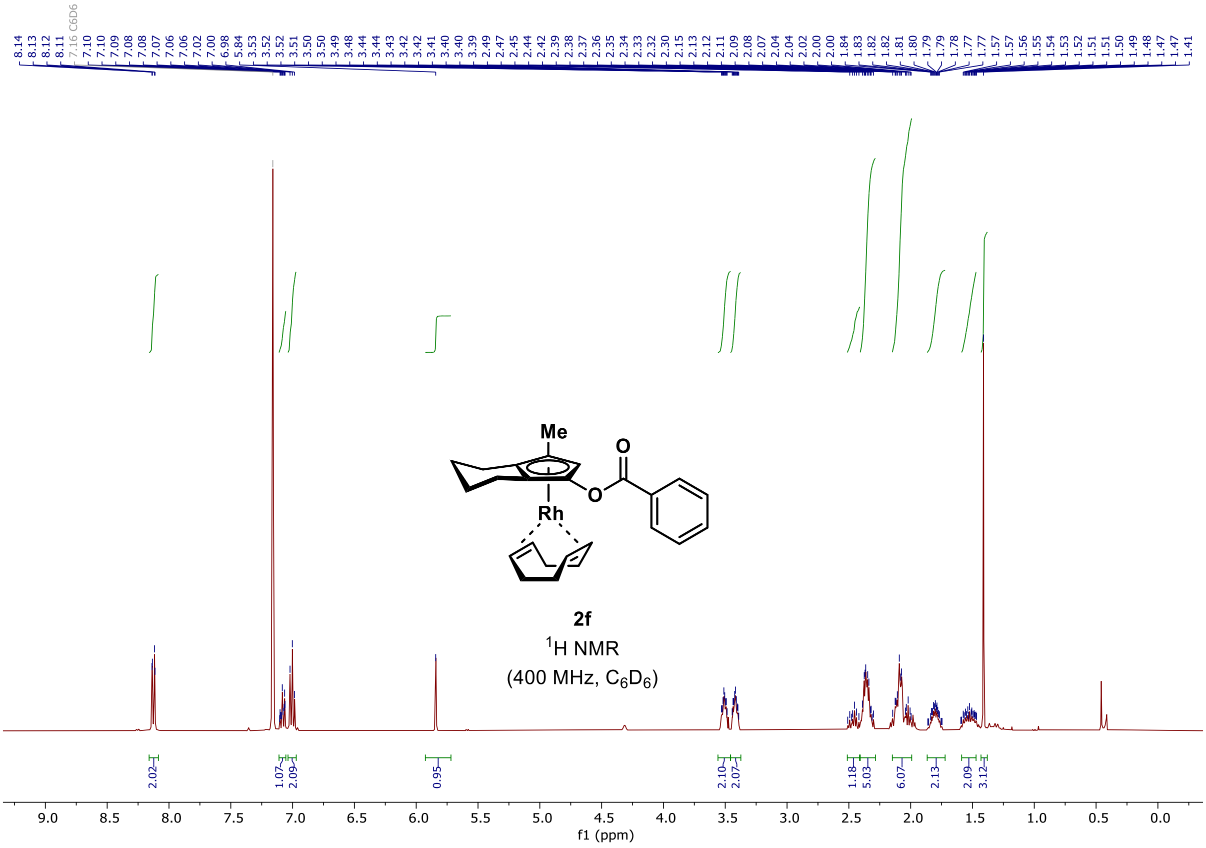
8.14 8.13 8.12 8.11 7.16 C6D6 7.10 7.10 7.09 7.08 7.08 7.07 7.06 7.06 7.02 7.00 6.98 5.84 3.53 3.52 3.52 3.51 3.50 3.50 3.49 3.48 3.44 3.44 3.43 3.42 3.42 3.41 3.40 3.40 3.39 2.49 2.47 2.45 2.44 2.42 2.39 2.38 2.37 2.36 2.35 2.34 2.33 2.32 2.30 2.15 2.13 2.12 2.11 2.09 2.08 2.07 2.04 2.04 2.02 2.00 2.00 1.84 1.83 1.82 1.82 1.81 1.80 1.79 1.79 1.78 1.77 1.77 1.57 1.57 1.56 1.55 1.54 1.53 1.52 1.51 1.51 1.50 1.49 1.48 1.47 1.41
Me
O
O
Rh
2f
^{1}H NMR
(400 MHz, C_6D_6)
2.02
1.07
2.09
0.95
2.10
2.07
1.18
5.03
6.07
2.13
2.09
3.12
9.0 8.5 8.0 7.5 7.0 6.5 6.0 5.5 5.0 4.5 4.0 3.5 3.0 2.5 2.0 1.5 1.0 0.5 0.0
f1 (ppm)

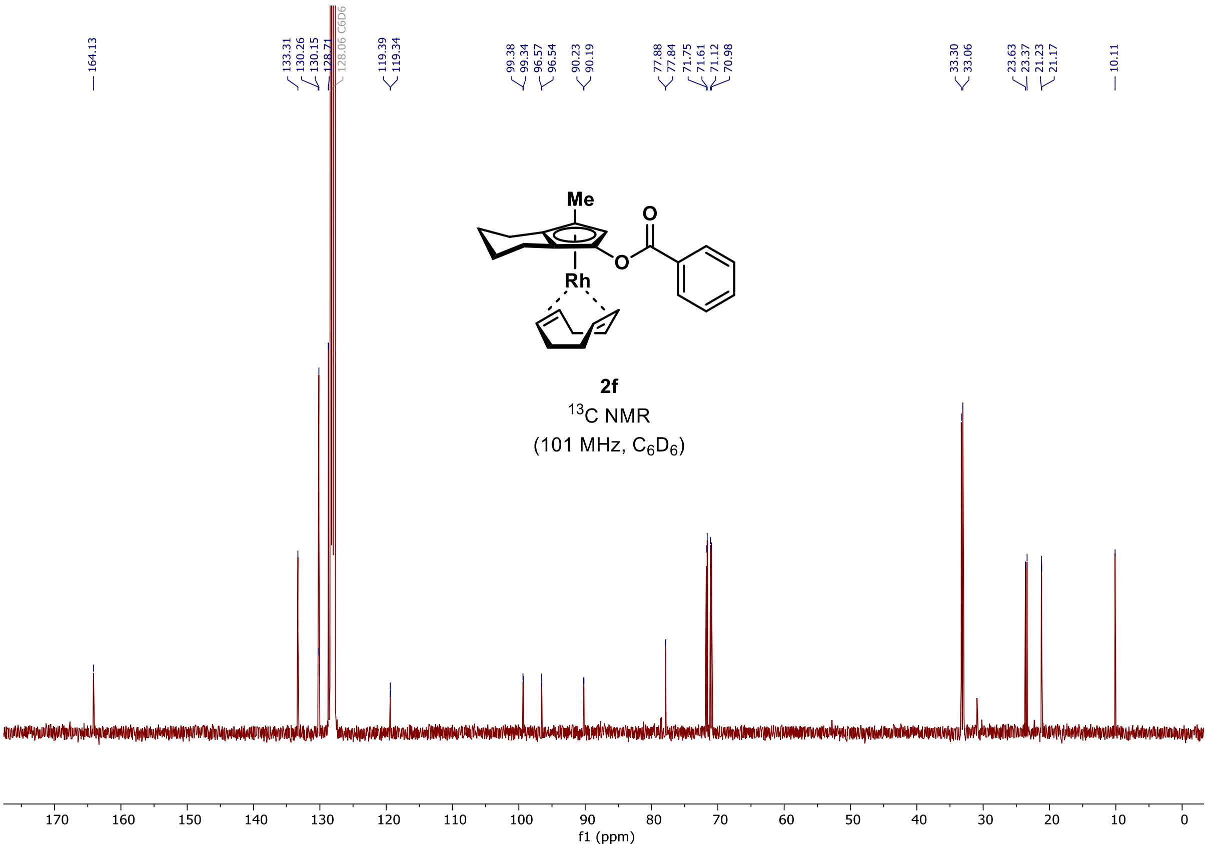
164.13 133.31 130.26 130.15 128.71 128.06 C6D6 119.39 119.34 99.38 99.34 96.57 96.54 90.23 90.19 77.88 77.84 71.75 71.61 71.12 70.98 33.30 33.06 23.63 23.37 21.23 21.17 10.11
Me
O
O
Rh
2f
^{13}C NMR
(101 MHz, C_6D_6)
170 160 150 140 130 120 110 100 90 80 70 60 50 40 30 20 10 0
f1 (ppm)

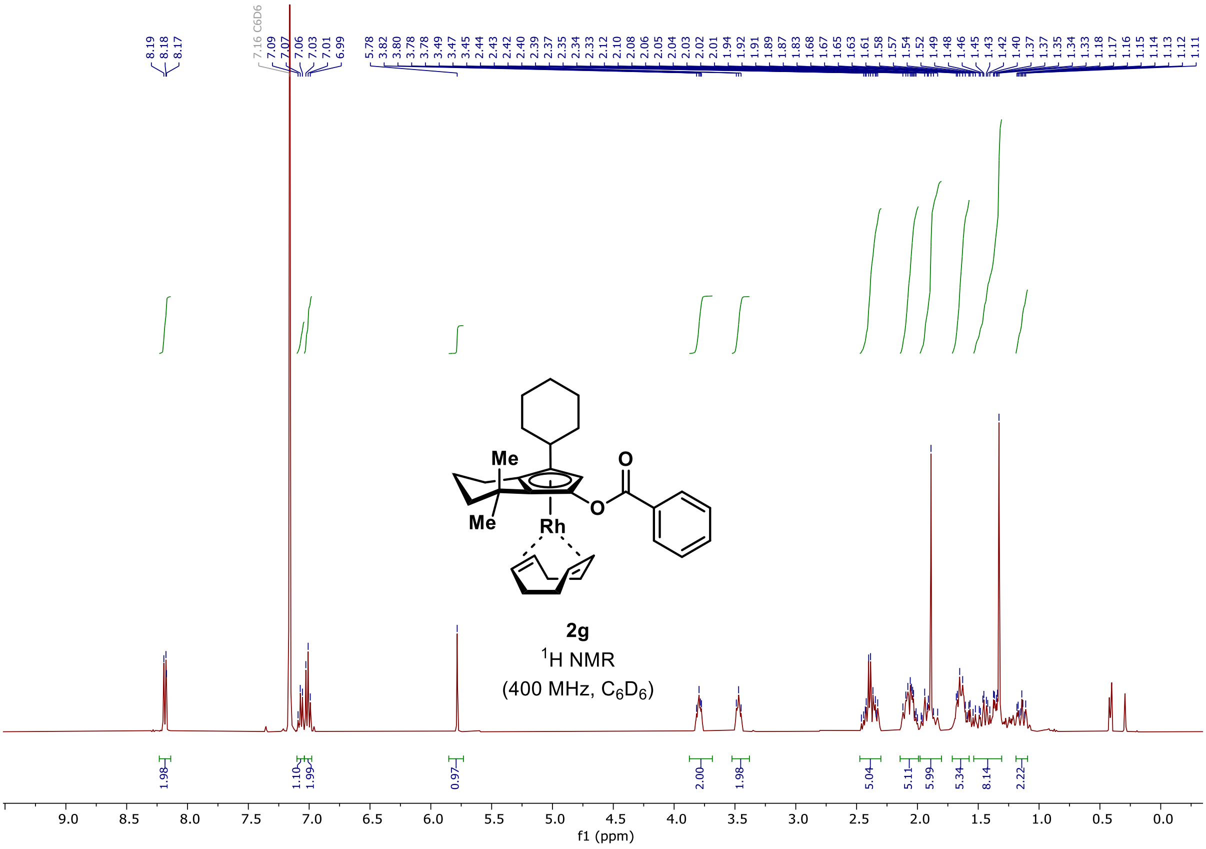

Me
Me
O
O
Rh
2g
^{1}H NMR
(400 MHz, C_6D_6)
f1 (ppm)

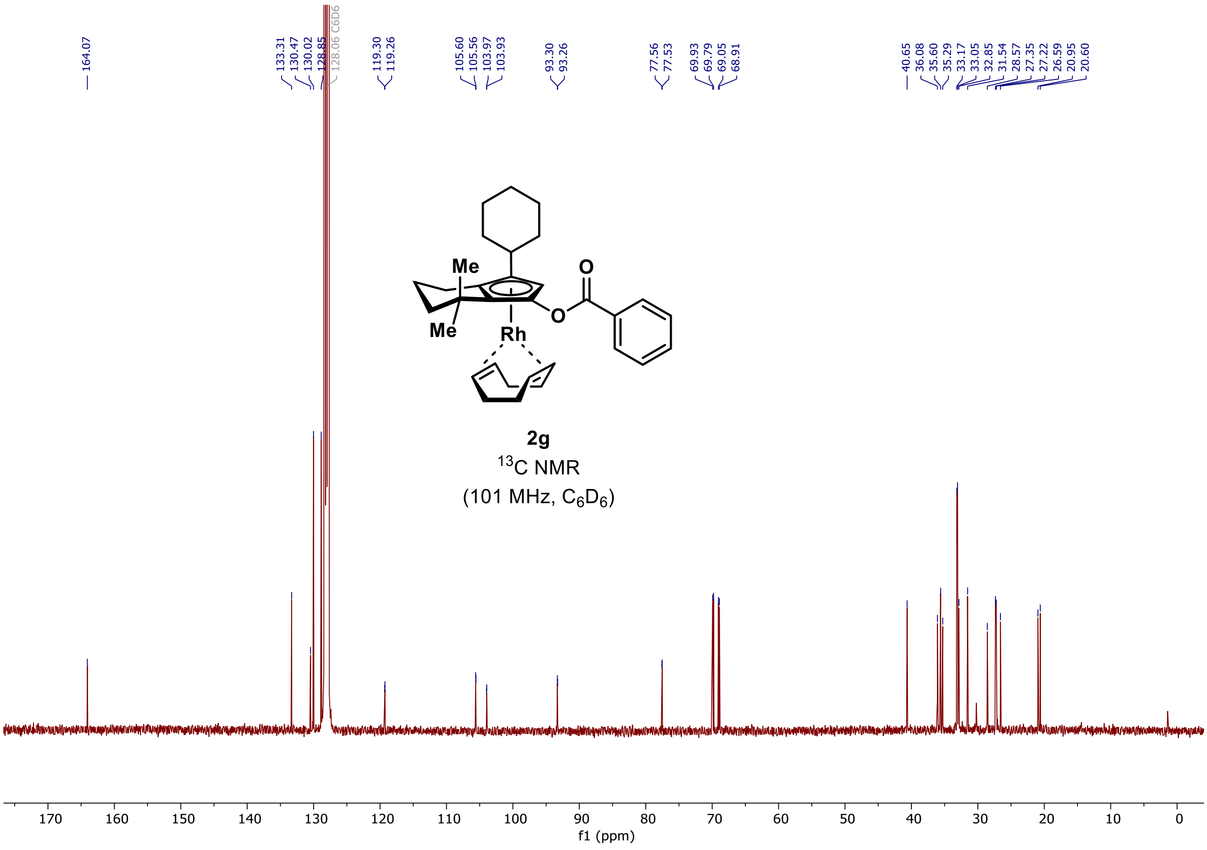

Me
Me
O
O
Rh
2g
^{13}C NMR
(101 MHz, C_6D_6)
f1 (ppm)

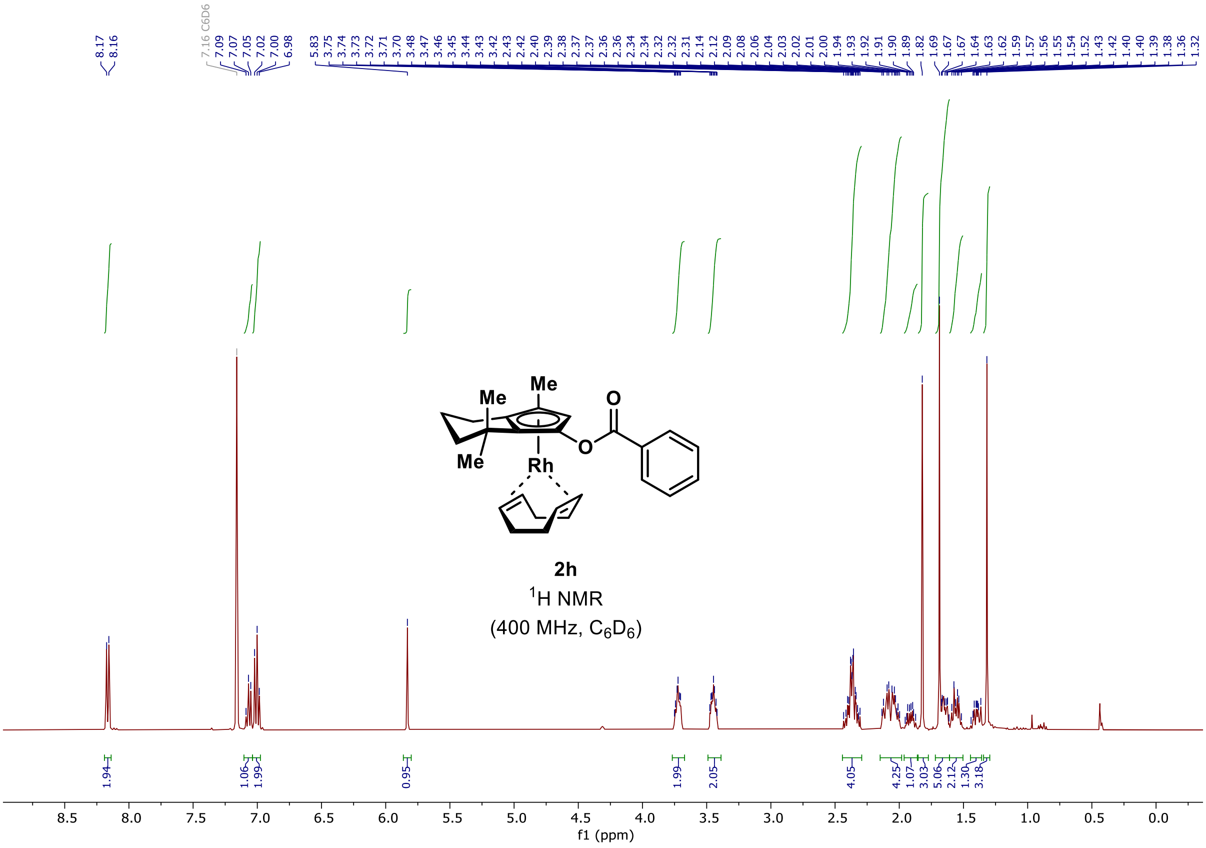
2h
^{1}H NMR
(400 MHz, C_6D_6)
f1 (ppm)

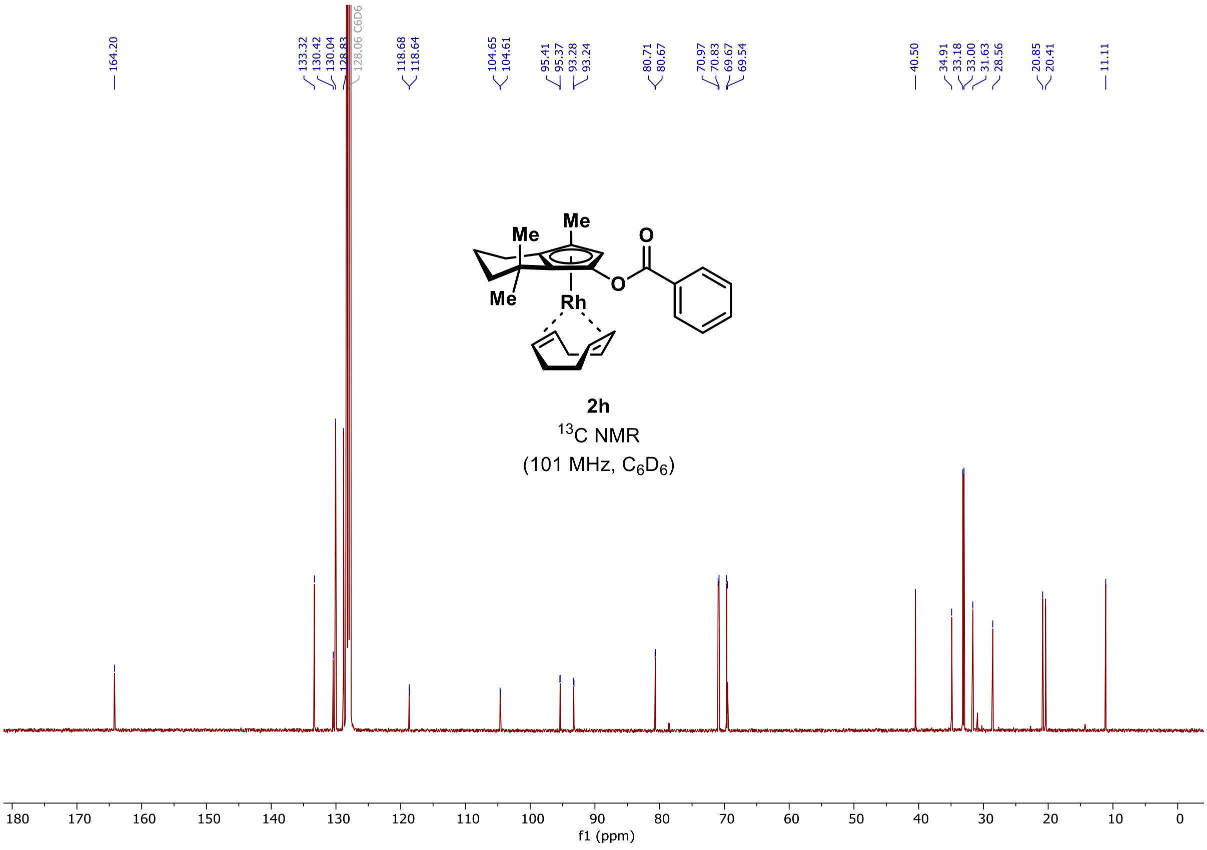
2h
^{13}C NMR
(101 MHz, C_6D_6)
f1 (ppm)

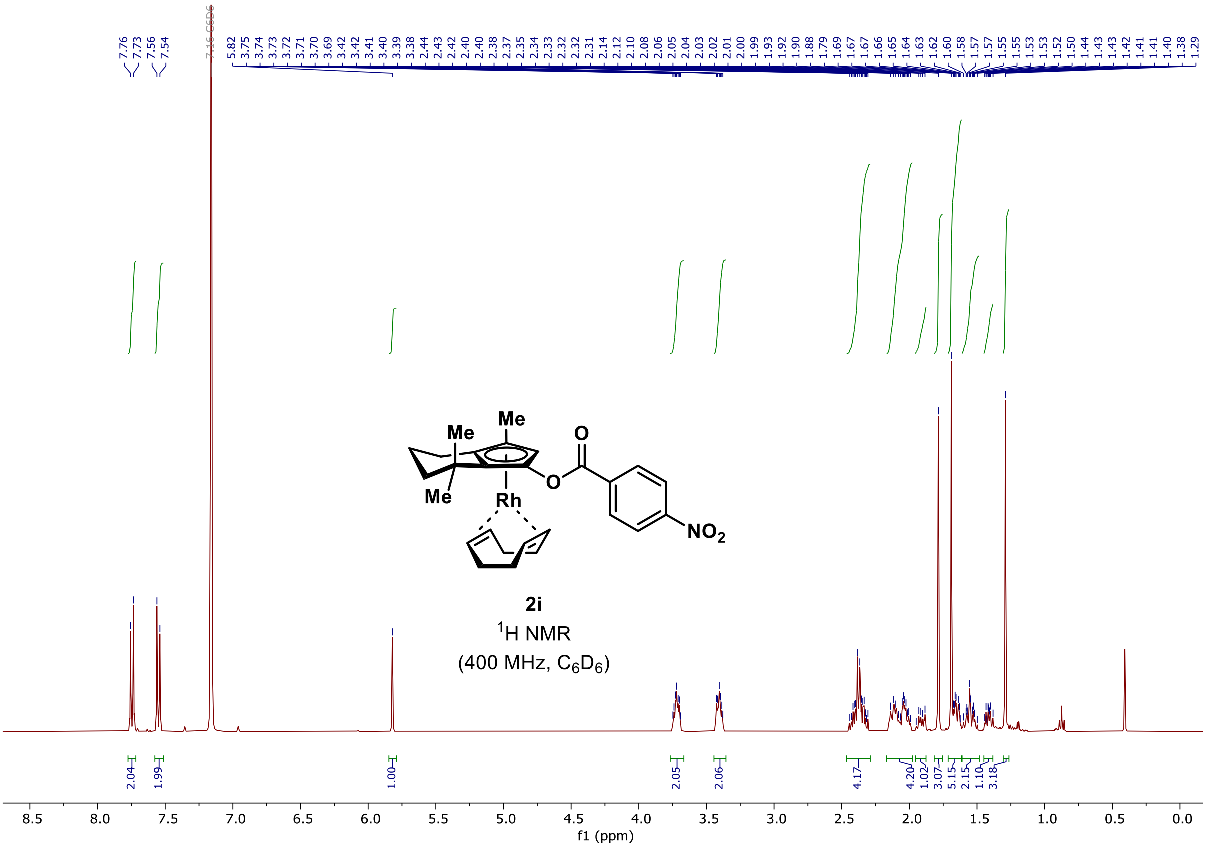
Me
Me
Me
O
Rh
NO2
2i
1H NMR
(400 MHz, C6D6)
f1 (ppm)

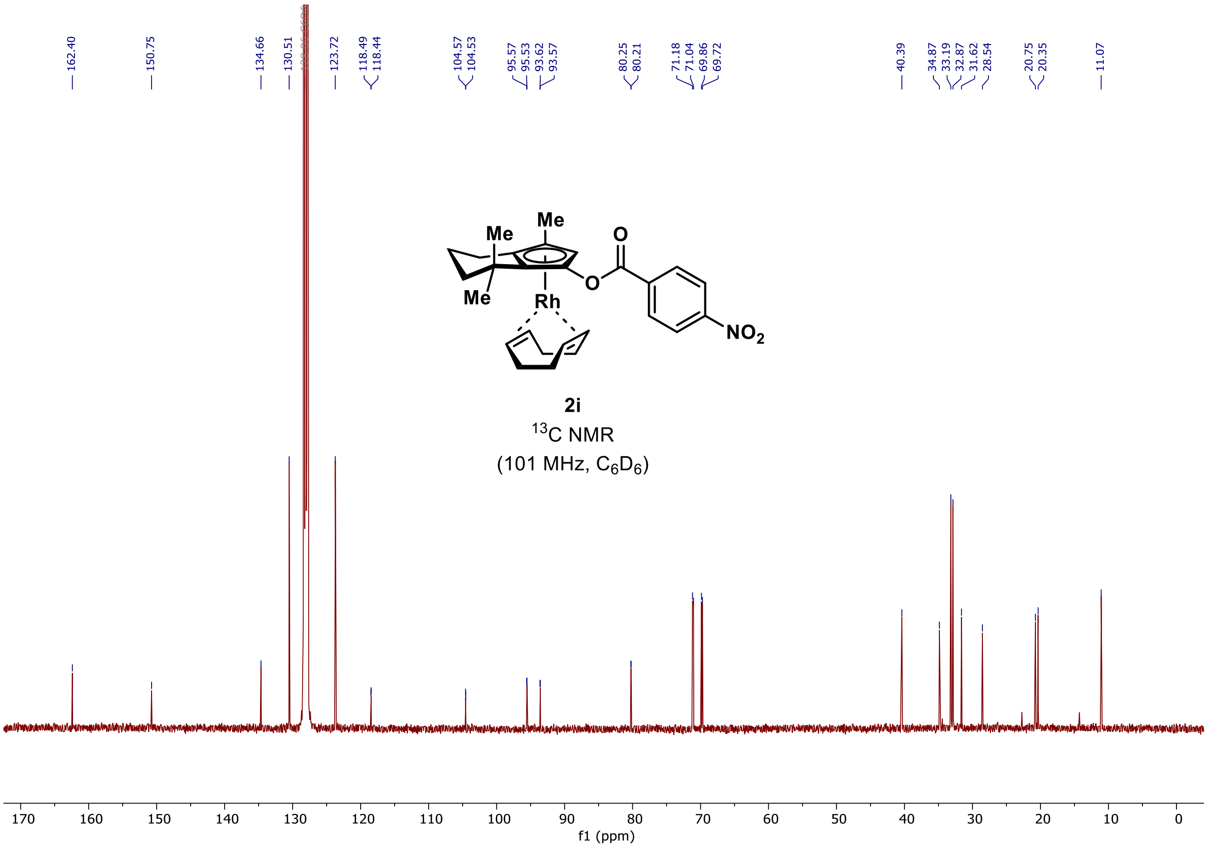
Me
Me
Me
O
Rh
NO2
2i
13C NMR
(101 MHz, C6D6)
f1 (ppm)

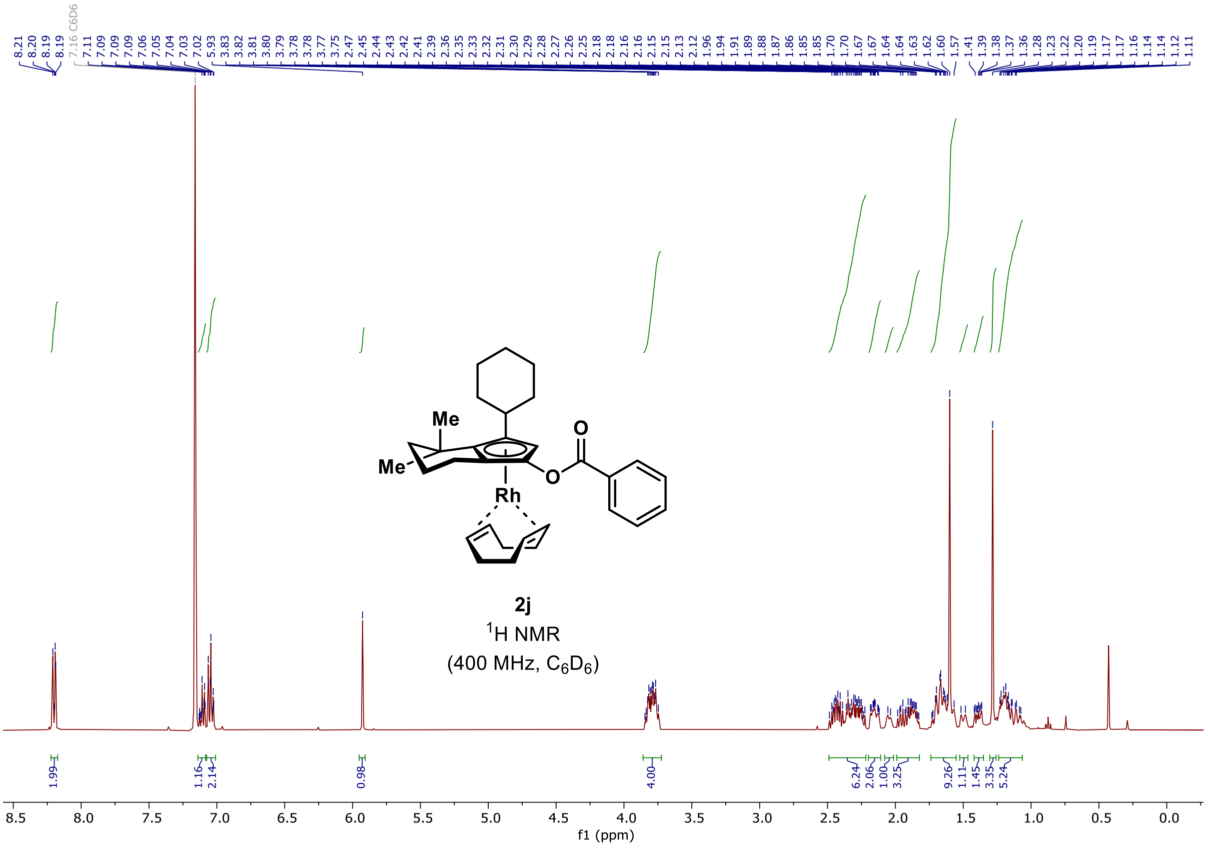

2j
^{1}H NMR
(400 MHz, C_6D_6)
f1 (ppm)

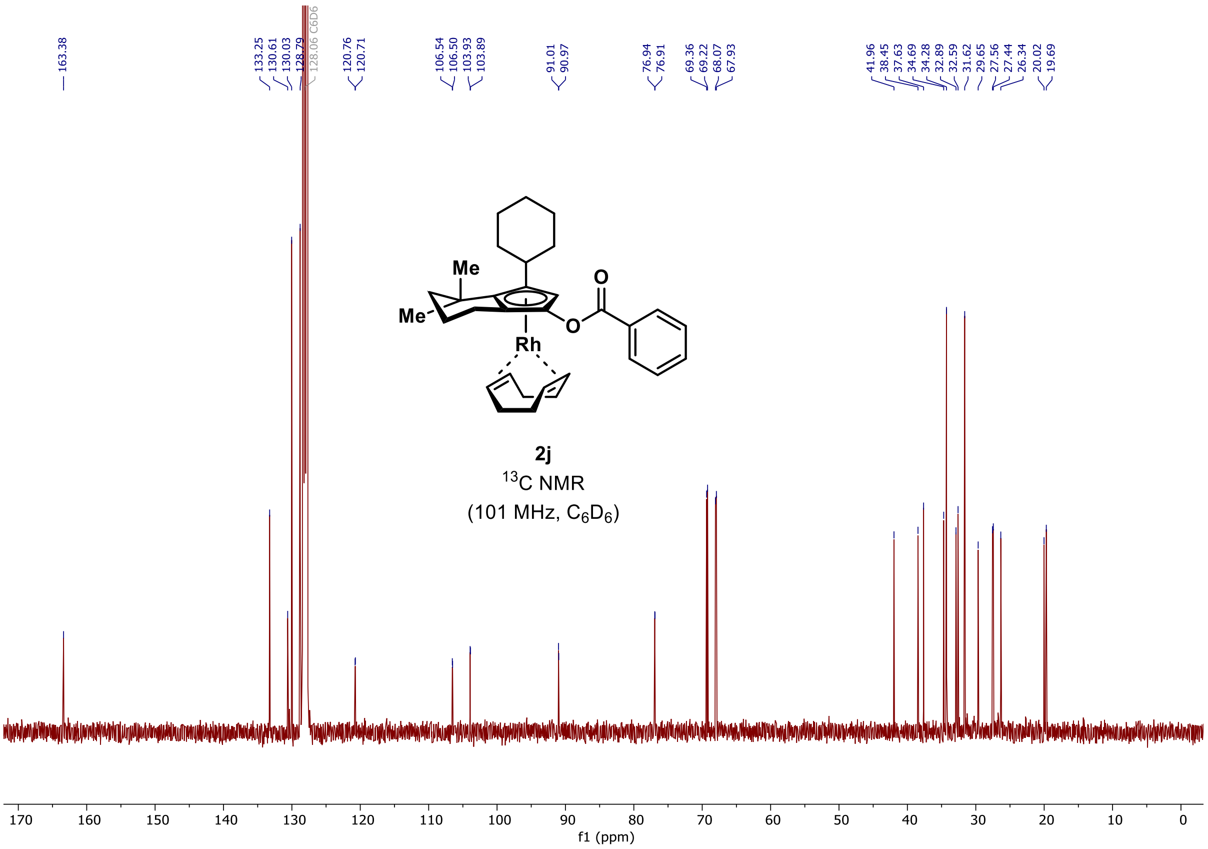

2j
^{13}C NMR
(101 MHz, C_6D_6)
f1 (ppm)

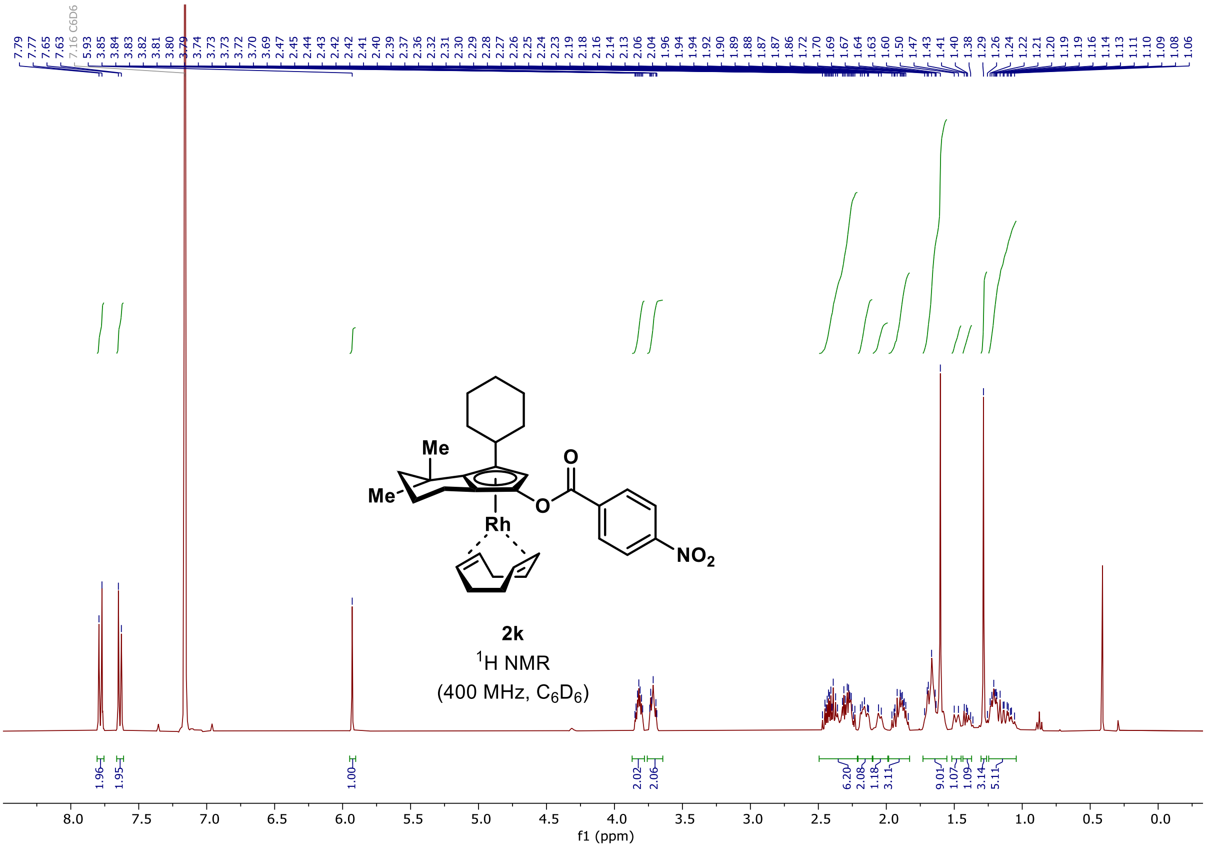
2k
1H NMR
(400 MHz, C6D6)
f1 (ppm)

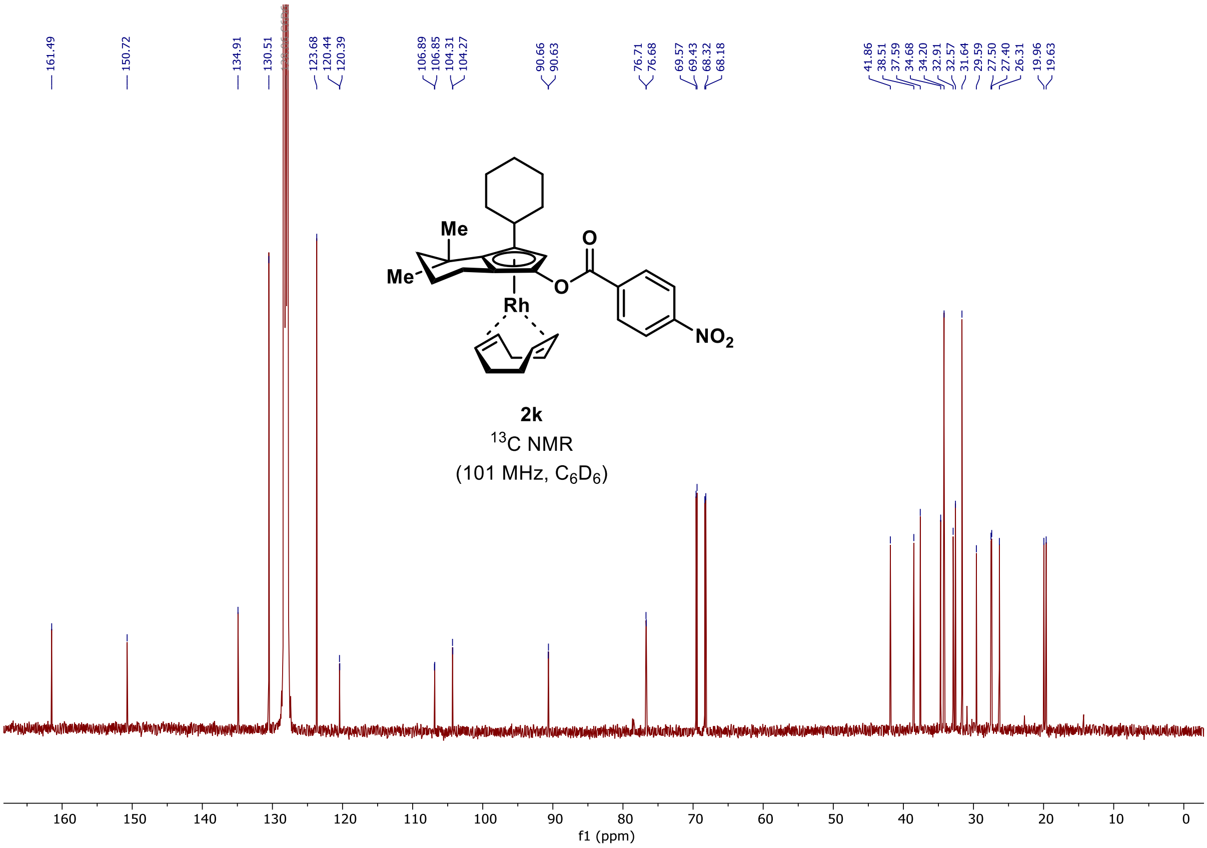
2k
13C NMR
(101 MHz, C6D6)
f1 (ppm)

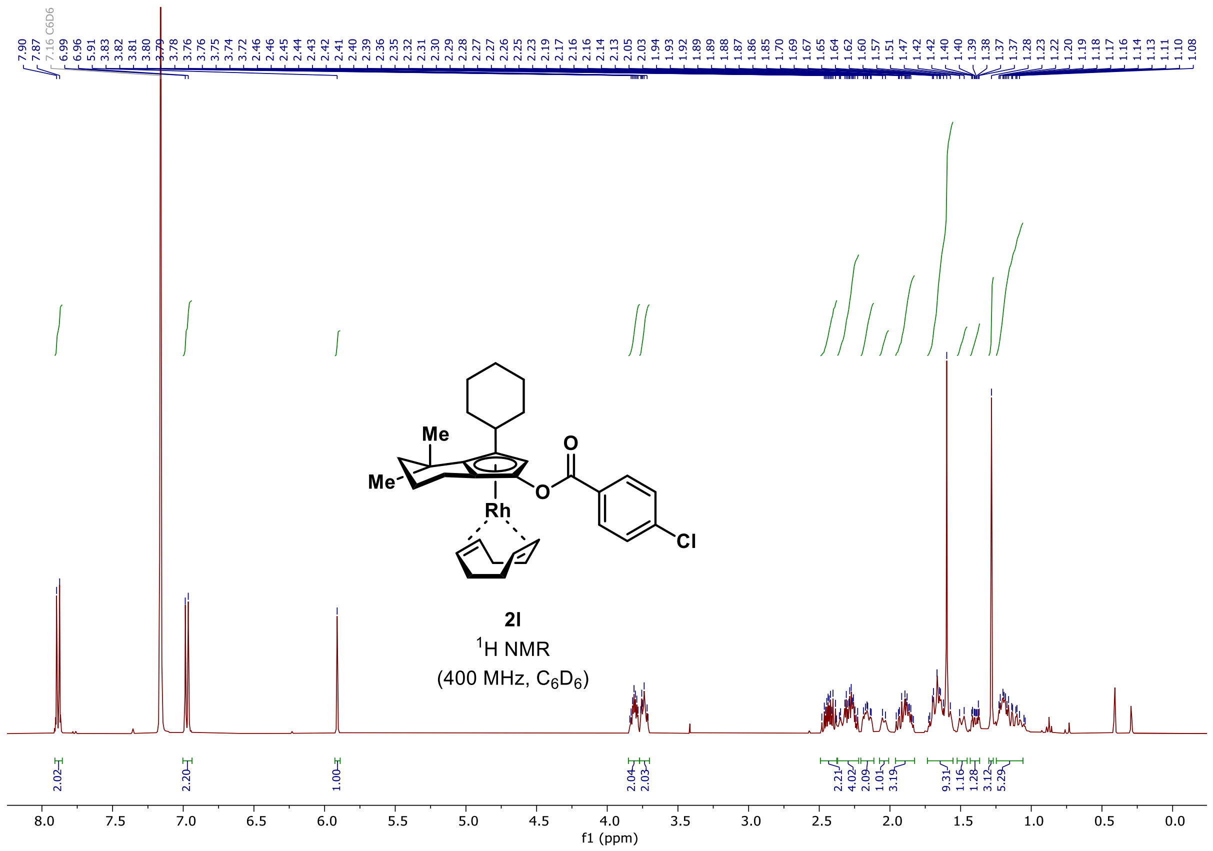
Me
Me
O
O
Rh
Cl
2l
^{1}H NMR
(400 MHz, C_6D_6)
f1 (ppm)

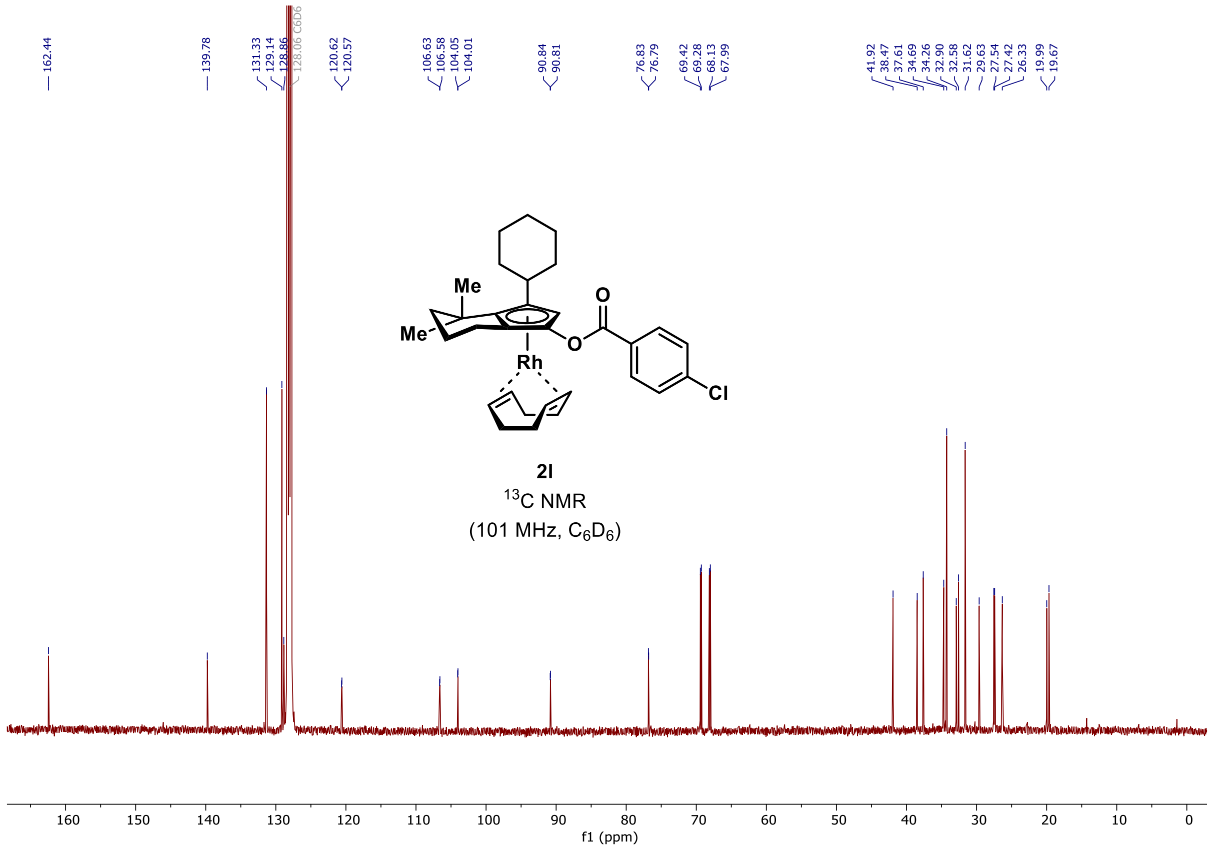
Me
Me
O
O
Rh
Cl
2l
^{13}C NMR
(101 MHz, C_6D_6)
f1 (ppm)

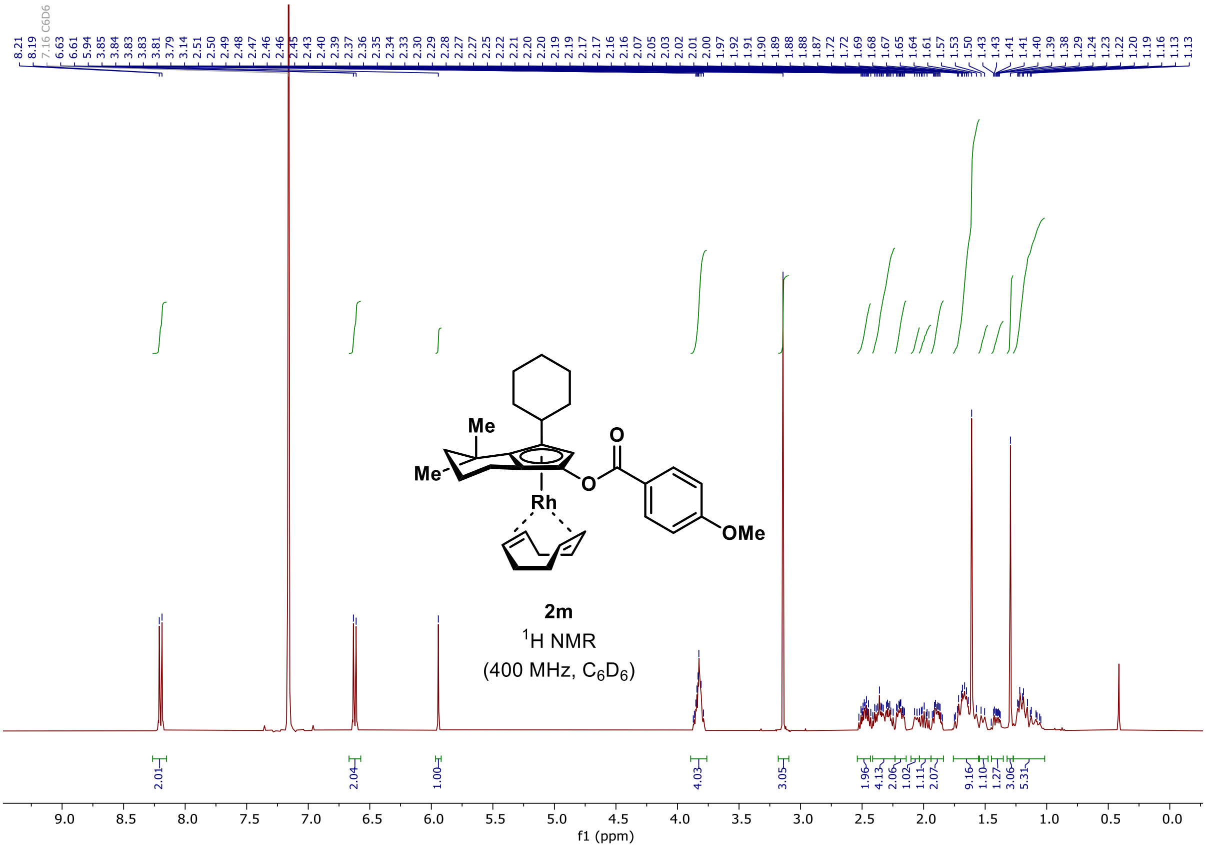
8.21 8.19 7.16 C6D6 6.63 6.61 5.94 3.85 3.84 3.83 3.83 3.81 3.79 3.14 2.51 2.50 2.49 2.48 2.47 2.46 2.46 2.45 2.43 2.40 2.39 2.37 2.36 2.35 2.34 2.33 2.30 2.29 2.28 2.27 2.27 2.25 2.22 2.21 2.20 2.20 2.19 2.19 2.17 2.16 2.16 2.07 2.05 2.03 2.02 2.01 2.00 1.97 1.92 1.91 1.90 1.89 1.88 1.88 1.87 1.72 1.72 1.69 1.68 1.67 1.65 1.64 1.61 1.57 1.53 1.50 1.43 1.43 1.41 1.41 1.40 1.39 1.38 1.29 1.24 1.23 1.22 1.20 1.19 1.16 1.13
Me
Me
O
Rh
OMe
2m
1H NMR
(400 MHz, C6D6)
2.01 2.04 1.00 4.03 3.05 1.96 4.13 2.06 1.02 1.11 2.07 9.16 1.10 1.27 3.06 5.31
9.0 8.5 8.0 7.5 7.0 6.5 6.0 5.5 5.0 4.5 4.0 3.5 3.0 2.5 2.0 1.5 1.0 0.5 0.0
f1 (ppm)

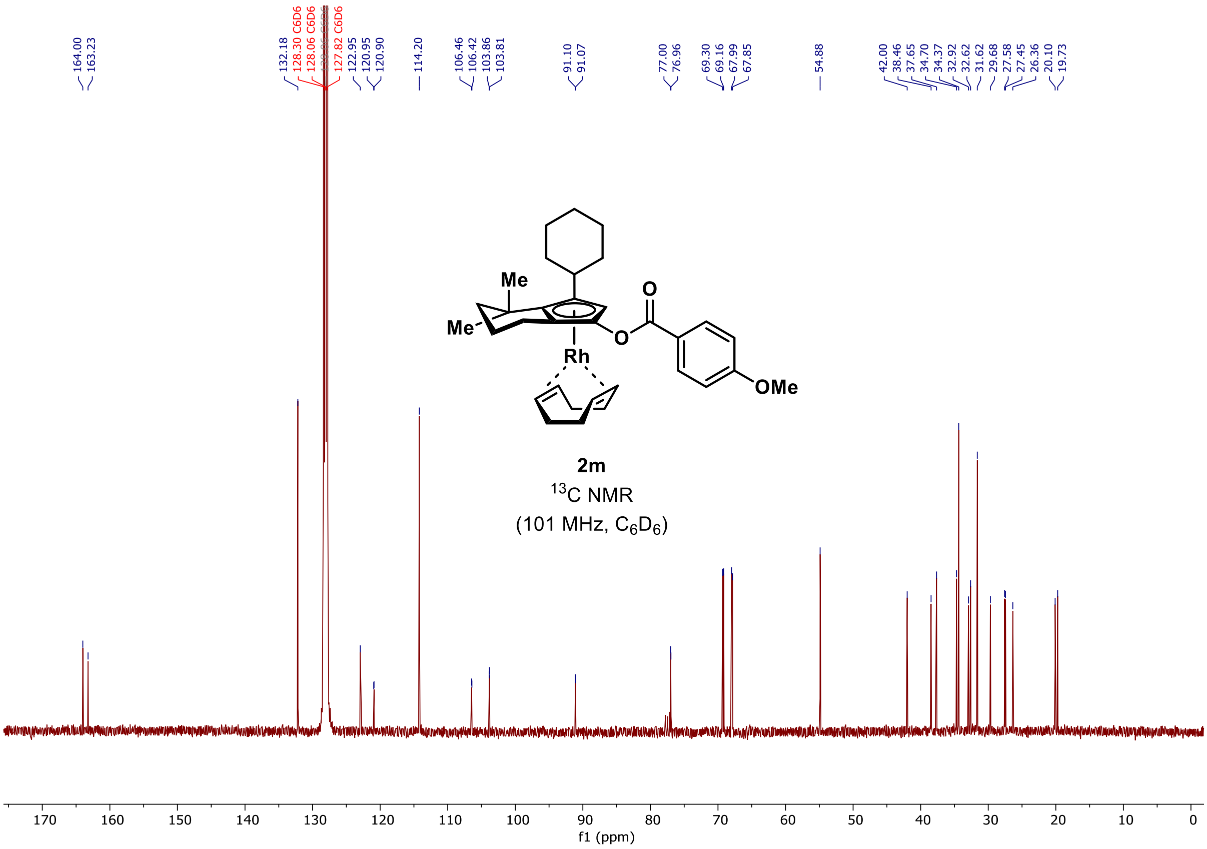
164.00 163.23 132.18 128.30 C6D6 128.06 C6D6 127.82 C6D6 122.95 120.95 120.90 114.20 106.46 106.42 103.86 103.81 91.10 91.07 77.00 76.96 69.30 69.16 67.99 67.85 54.88 42.00 38.46 37.65 34.70 34.37 32.92 32.62 31.62 29.68 27.58 27.45 26.36 20.10 19.73
Me
Me
O
Rh
OMe
2m
13C NMR
(101 MHz, C6D6)
170 160 150 140 130 120 110 100 90 80 70 60 50 40 30 20 10 0
f1 (ppm)

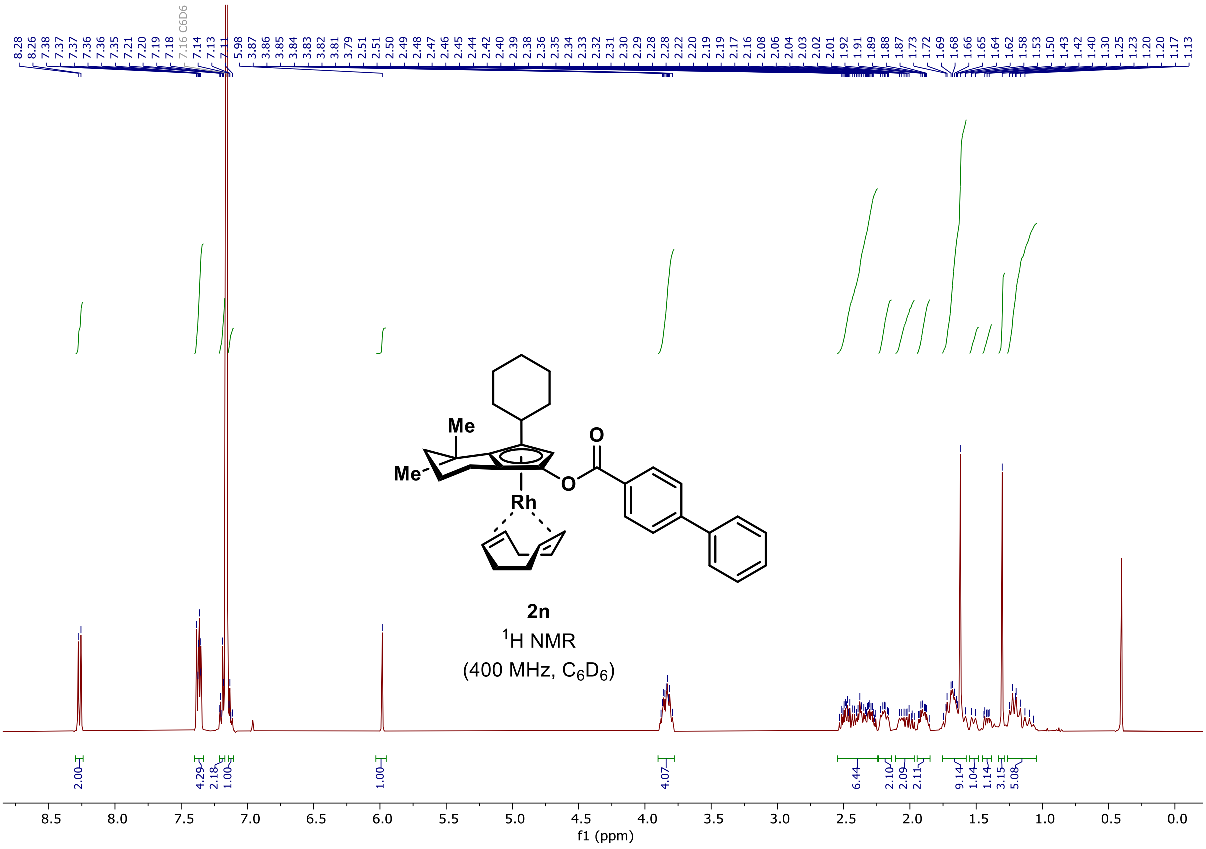
8.28 8.26 7.38 7.37 7.37 7.36 7.36 7.35 7.21 7.20 7.19 7.18 7.16 C6D6 7.14 7.13 7.11 5.98 3.87 3.86 3.85 3.84 3.83 3.82 3.81 3.79 2.51 2.51 2.50 2.49 2.48 2.47 2.46 2.45 2.44 2.42 2.40 2.39 2.38 2.36 2.35 2.34 2.33 2.32 2.31 2.30 2.29 2.28 2.28 2.22 2.20 2.19 2.19 2.17 2.16 2.08 2.06 2.04 2.03 2.02 2.01 1.92 1.91 1.89 1.88 1.87 1.73 1.72 1.69 1.68 1.66 1.65 1.64 1.62 1.58 1.53 1.50 1.43 1.42 1.40 1.30 1.25 1.23 1.20 1.17 1.13
Me
Me
O
O
Rh
2n
^{1}H NMR
(400 MHz, C_6D_6)
2.00 4.29 2.18 1.00 1.00 4.07 6.44 2.10 2.09 2.11 9.14 1.04 1.14 3.15 5.08
8.5 8.0 7.5 7.0 6.5 6.0 5.5 5.0 4.5 4.0 3.5 3.0 2.5 2.0 1.5 1.0 0.5 0.0
f1 (ppm)

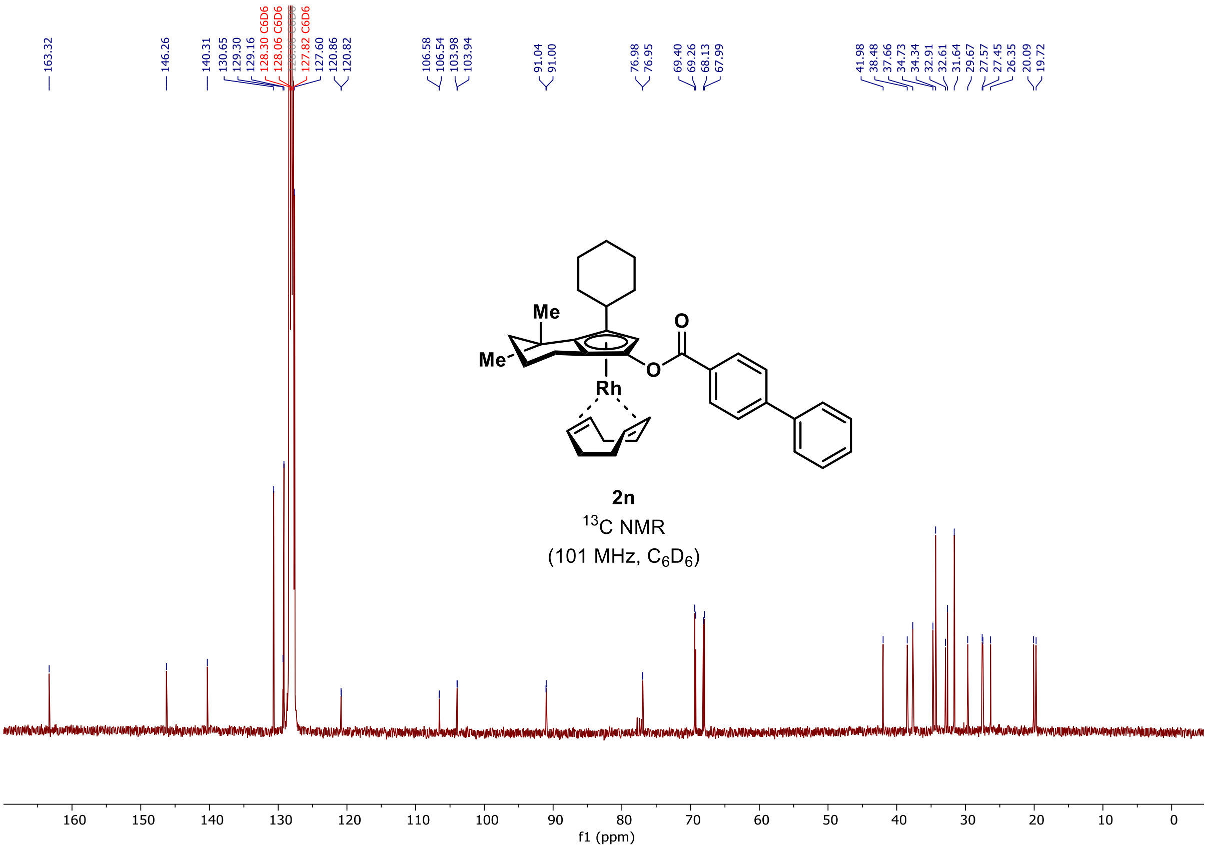
163.32 146.26 140.31 130.65 129.30 129.16 128.30 C6D6 128.06 C6D6 127.82 C6D6 127.60 120.86 120.82 106.58 106.54 103.98 103.94 91.04 91.00 76.98 76.95 69.40 69.26 68.13 67.99 41.98 38.48 37.66 34.73 34.34 32.91 32.61 31.64 29.67 27.57 27.45 26.35 20.09 19.72
Me
Me
O
O
Rh
2n
^{13}C NMR
(101 MHz, C_6D_6)
160 150 140 130 120 110 100 90 80 70 60 50 40 30 20 10 0
f1 (ppm)

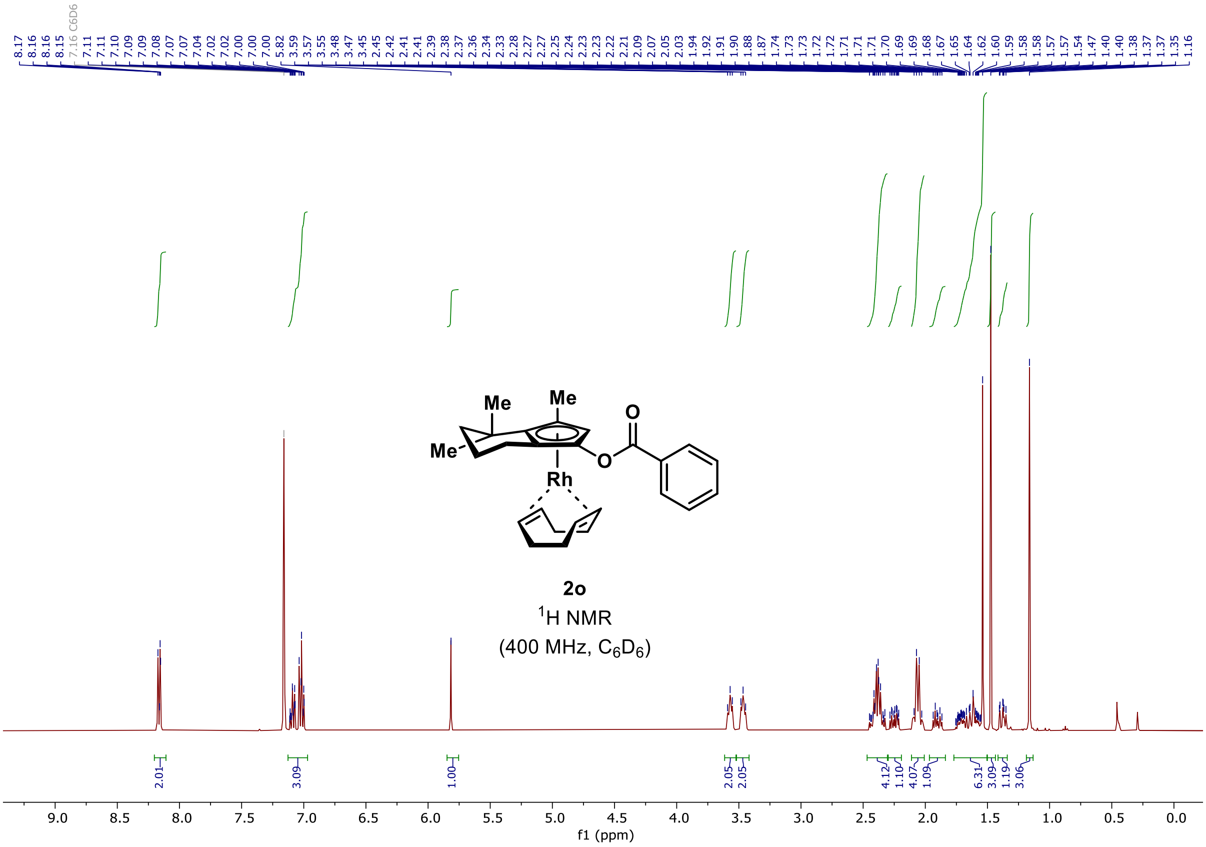
Me
Me
Me
O
O
Rh
2o
^{1}H NMR
(400 MHz, C_6D_6)
f1 (ppm)

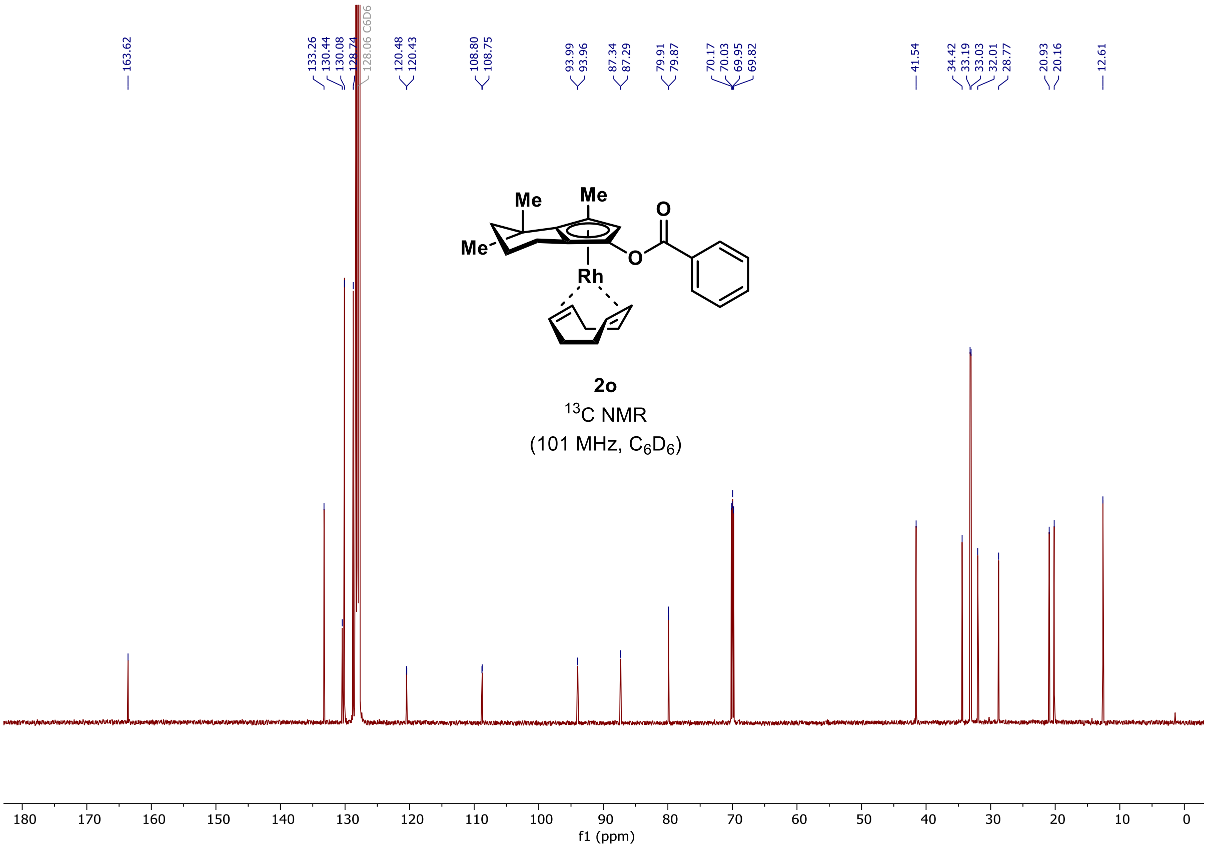
Me
Me
Me
O
O
Rh
2o
^{13}C NMR
(101 MHz, C_6D_6)
f1 (ppm)

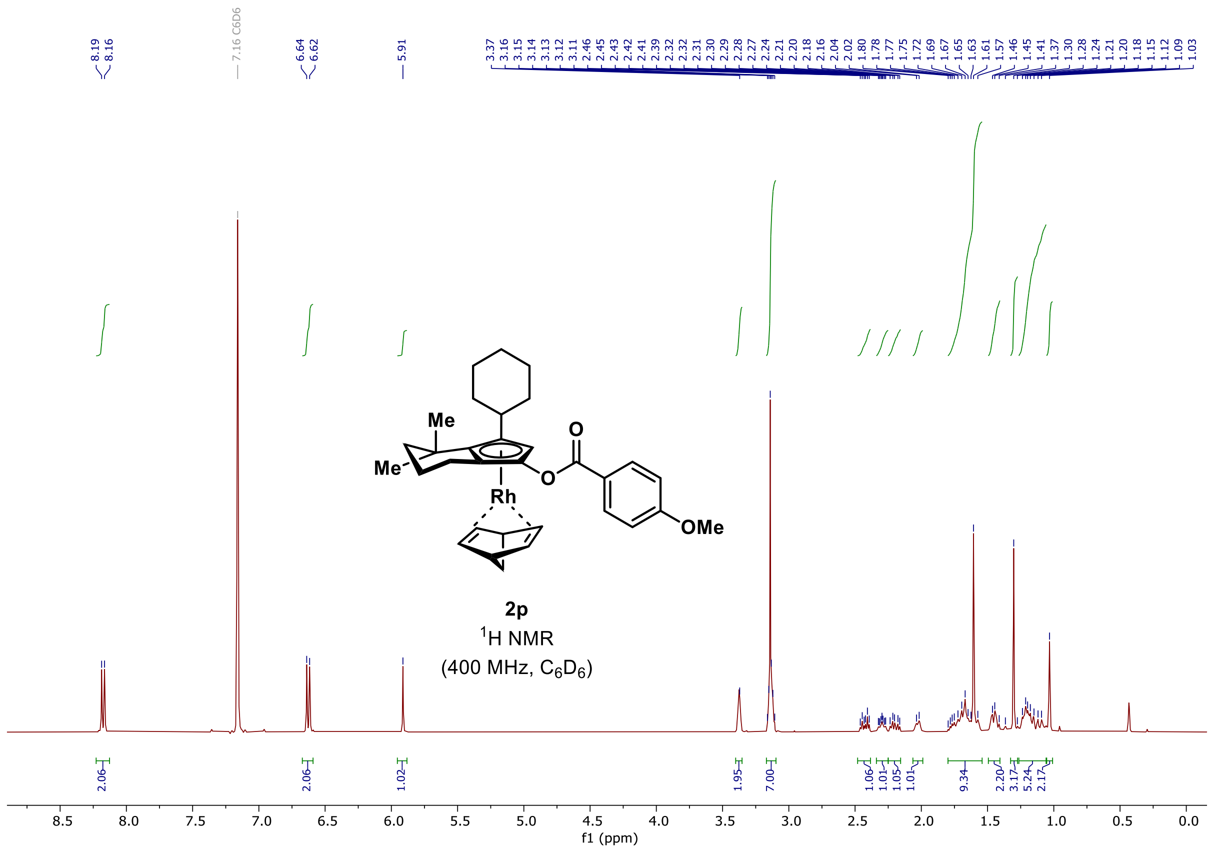

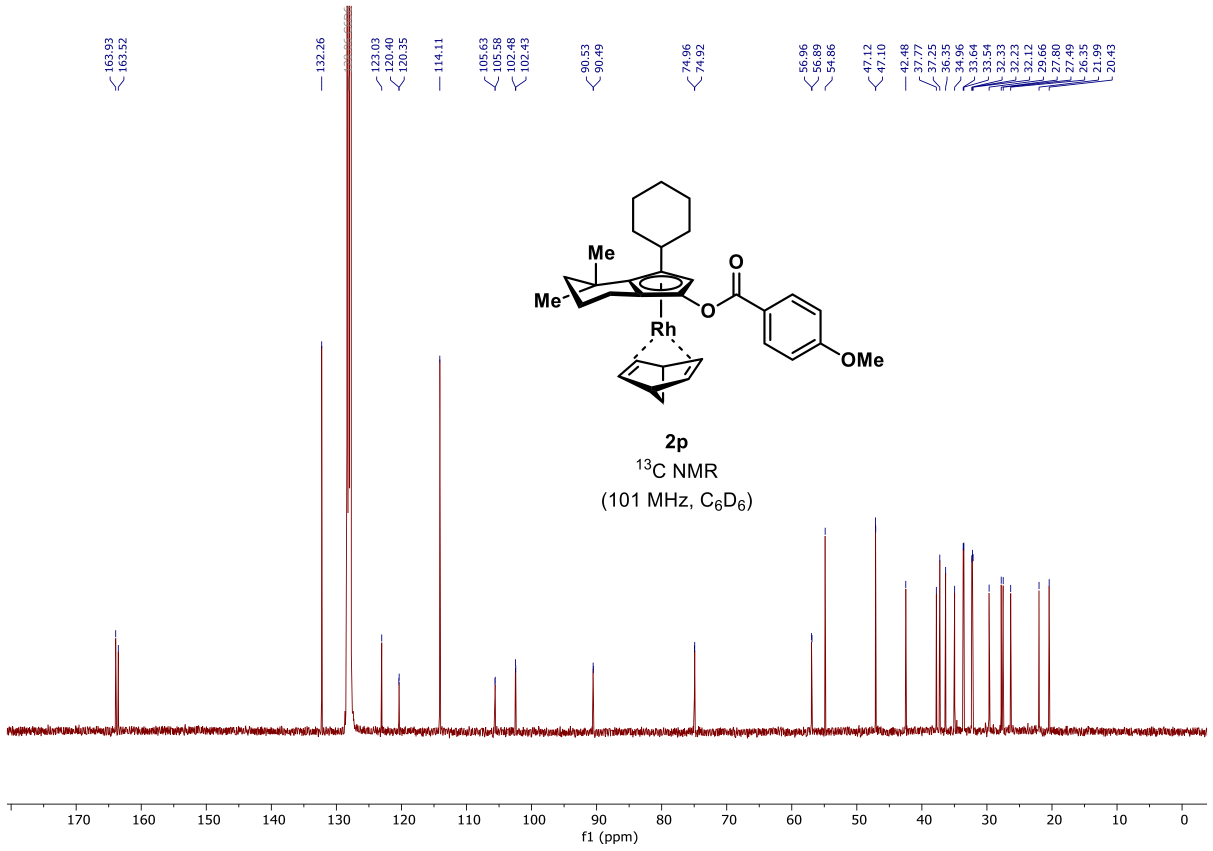

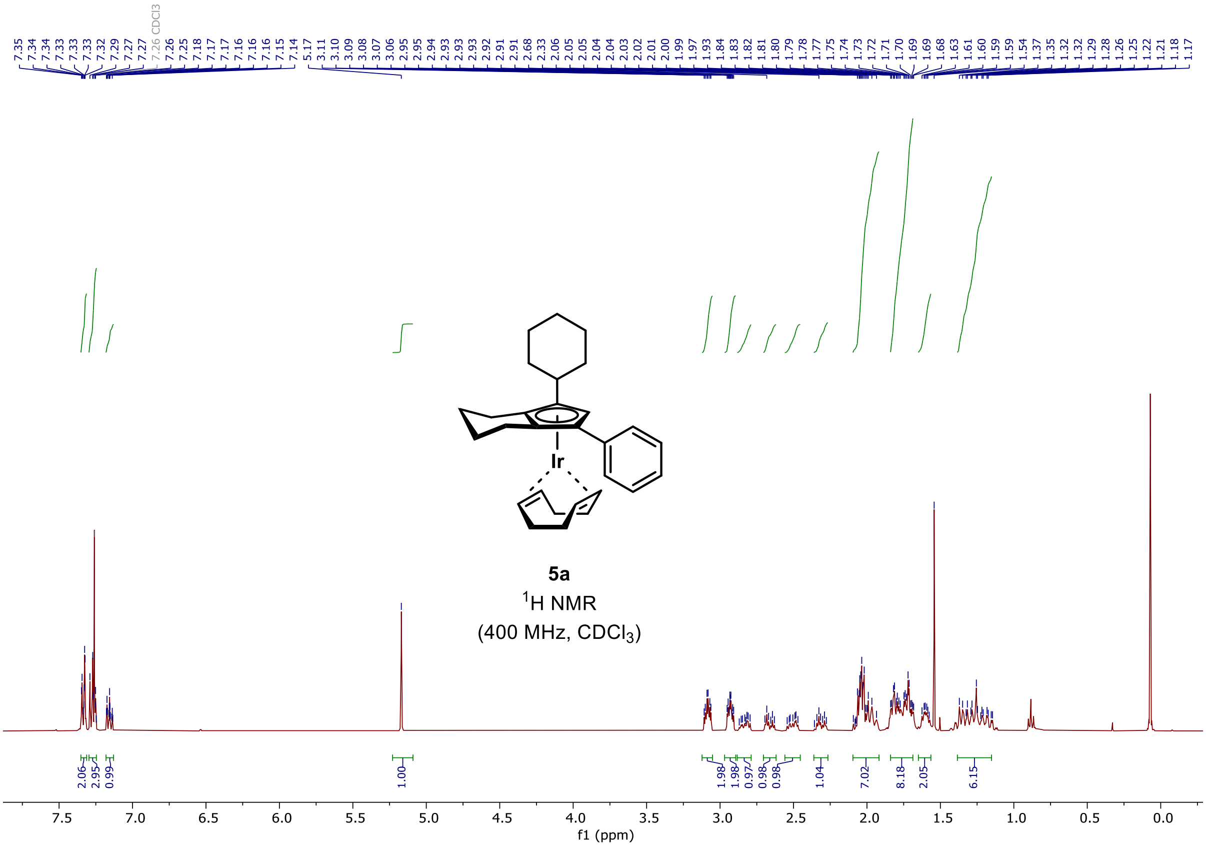
Ir
5a
1H NMR
(400 MHz, CDCl3)
f1 (ppm)

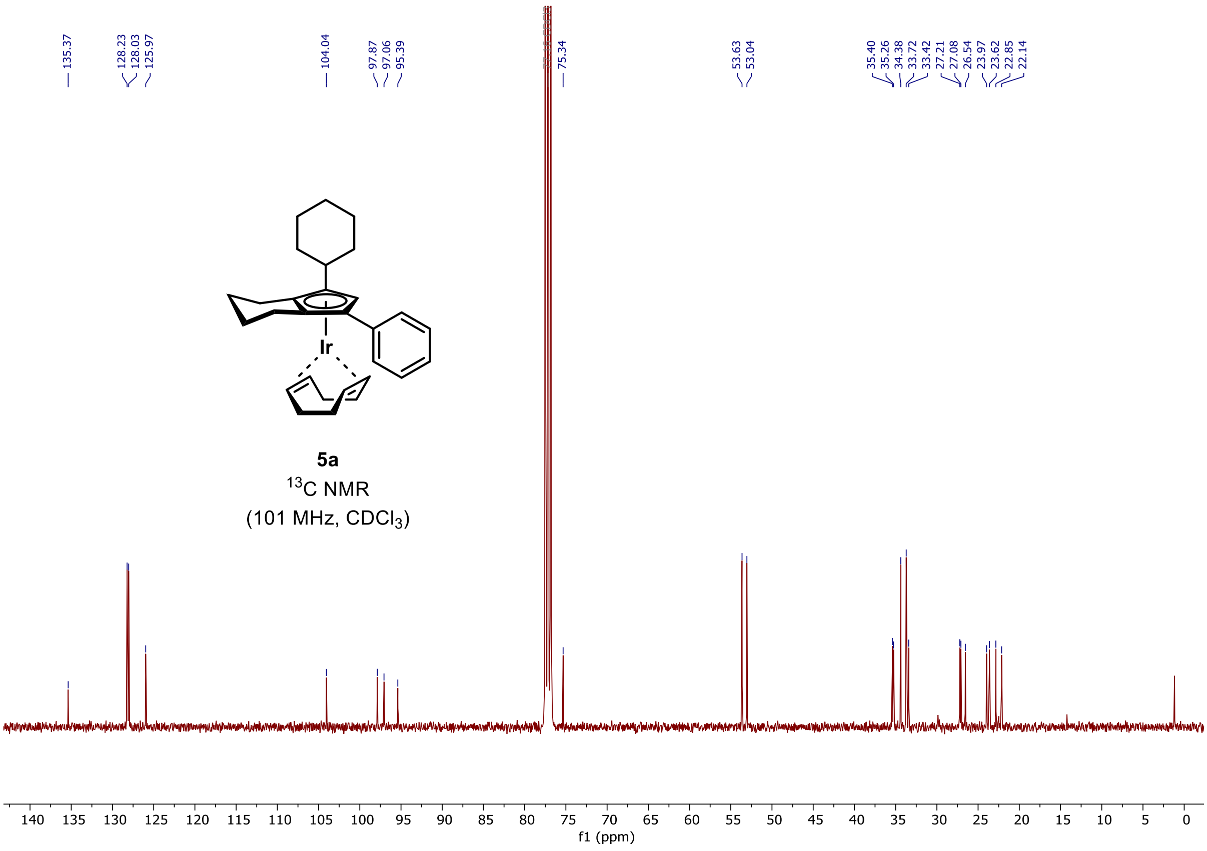
Ir
5a
13C NMR
(101 MHz, CDCl3)
f1 (ppm)

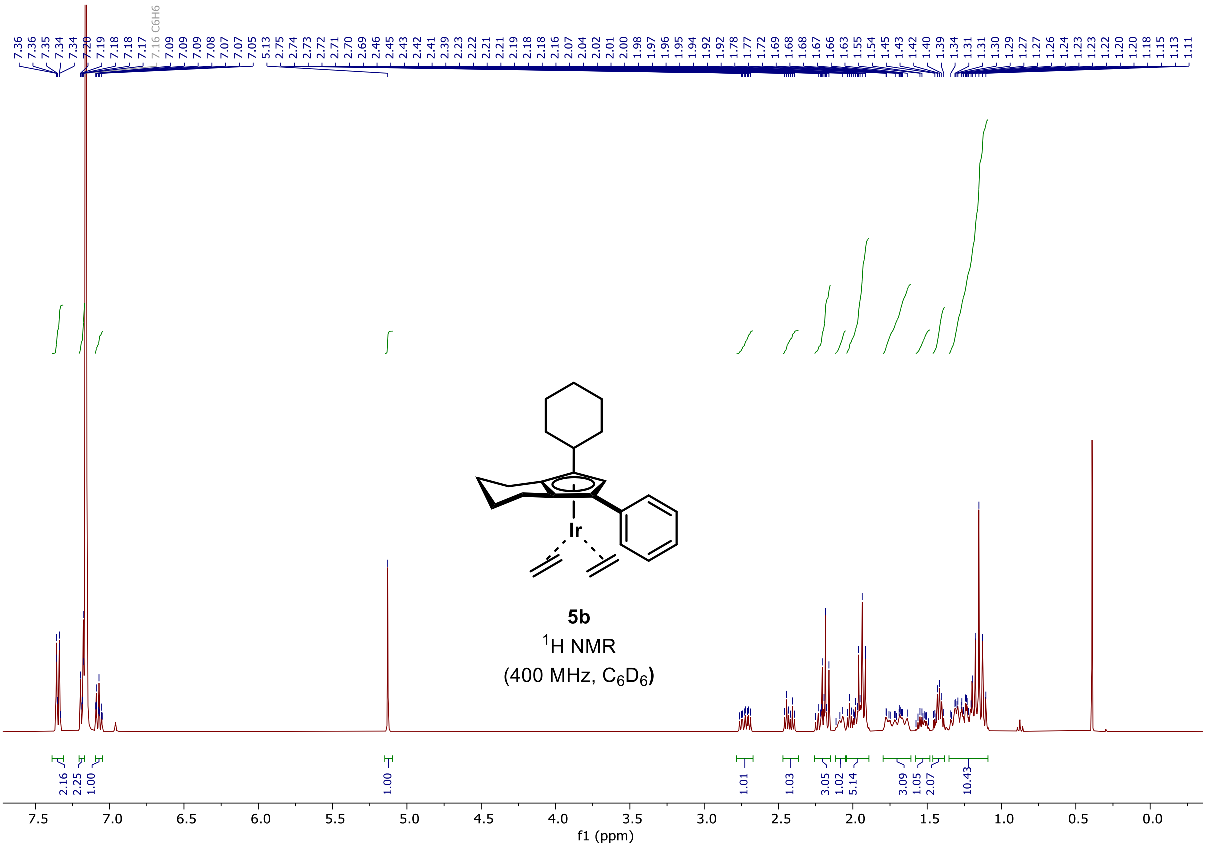
5b
^{1}H NMR
(400 MHz, C_6D_6)
f1 (ppm)

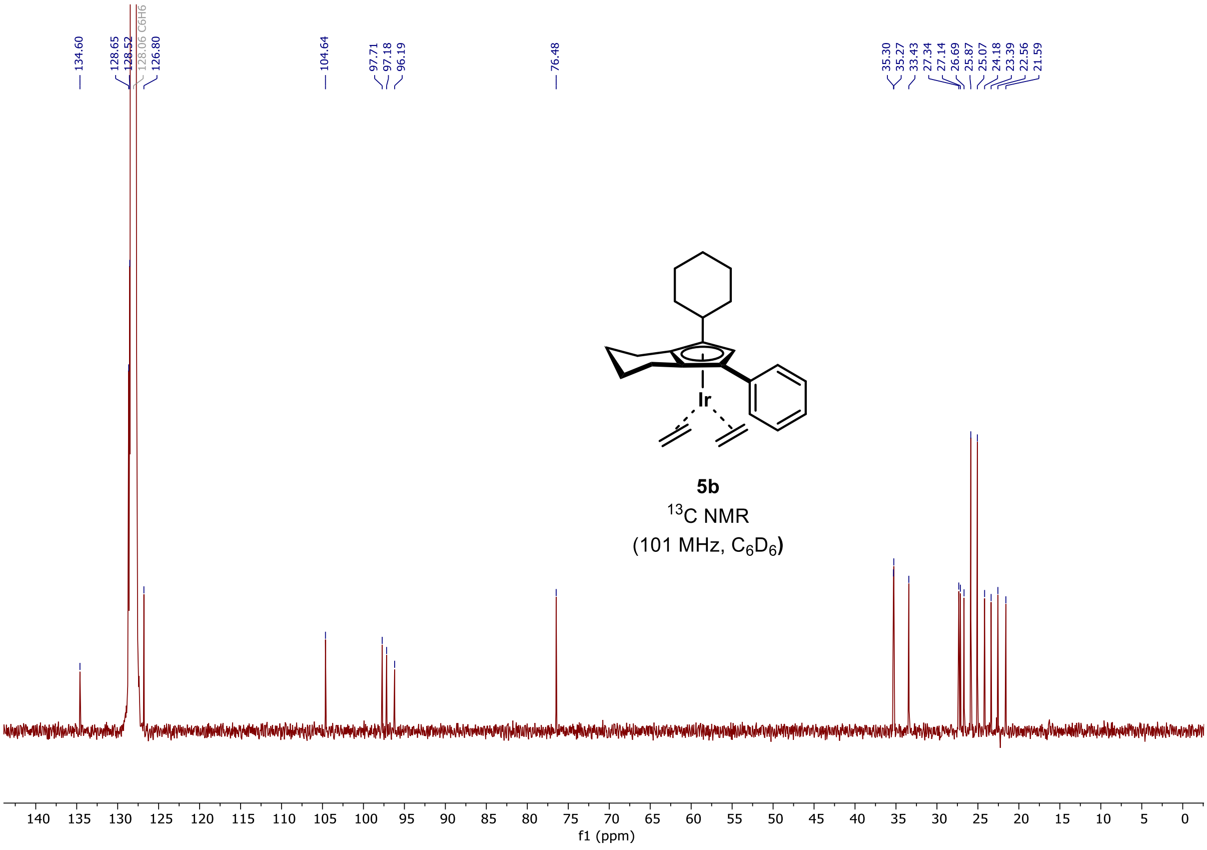
5b
^{13}C NMR
(101 MHz, C_6D_6)
f1 (ppm)

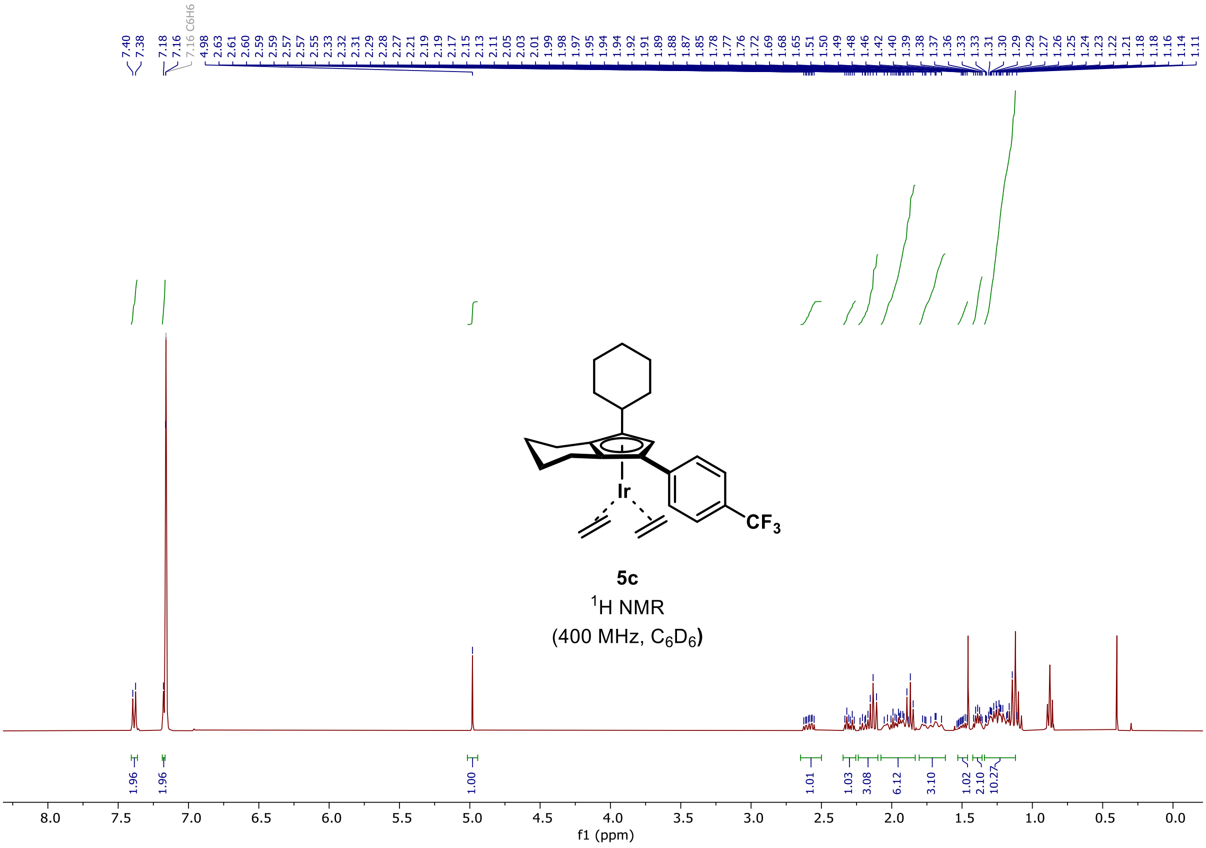
7.40
7.38
7.18
7.16
7.16 C6H6
4.98
2.63
2.61
2.60
2.59
2.59
2.57
2.57
2.55
2.33
2.32
2.31
2.29
2.28
2.27
2.21
2.19
2.19
2.17
2.15
2.13
2.11
2.05
2.03
2.01
1.99
1.98
1.97
1.95
1.94
1.92
1.91
1.89
1.88
1.87
1.85
1.78
1.77
1.76
1.72
1.69
1.68
1.65
1.51
1.50
1.49
1.48
1.46
1.42
1.40
1.39
1.38
1.37
1.36
1.33
1.33
1.31
1.30
1.29
1.29
1.27
1.26
1.25
1.24
1.23
1.22
1.21
1.18
1.18
1.16
1.14
1.11
Ir
CF_3
5c
^{1}H NMR
(400 MHz, C_6D_6)
1.96
1.96
1.00
1.01
1.03
3.08
6.12
3.10
1.02
2.10
10.27
8.0 7.5 7.0 6.5 6.0 5.5 5.0 4.5 4.0 3.5 3.0 2.5 2.0 1.5 1.0 0.5 0.0
f1 (ppm)

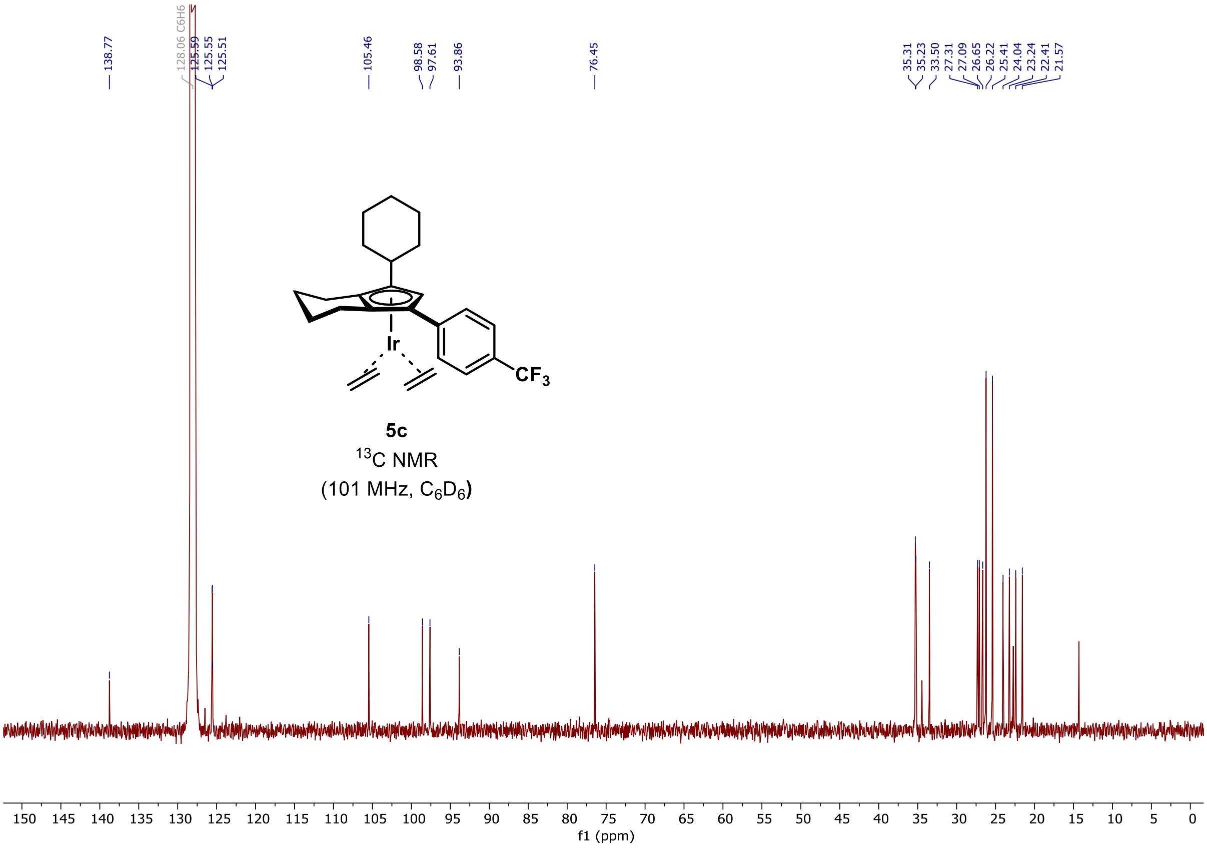
138.77
128.06 C6H6
125.59
125.55
125.51
105.46
98.58
97.61
93.86
76.45
35.31
35.23
33.50
27.31
27.09
26.65
26.22
25.41
24.04
23.24
22.41
21.57
Ir
CF_3
5c
^{13}C NMR
(101 MHz, C_6D_6)
150 145 140 135 130 125 120 115 110 105 100 95 90 85 80 75 70 65 60 55 50 45 40 35 30 25 20 15 10 5 0
f1 (ppm)

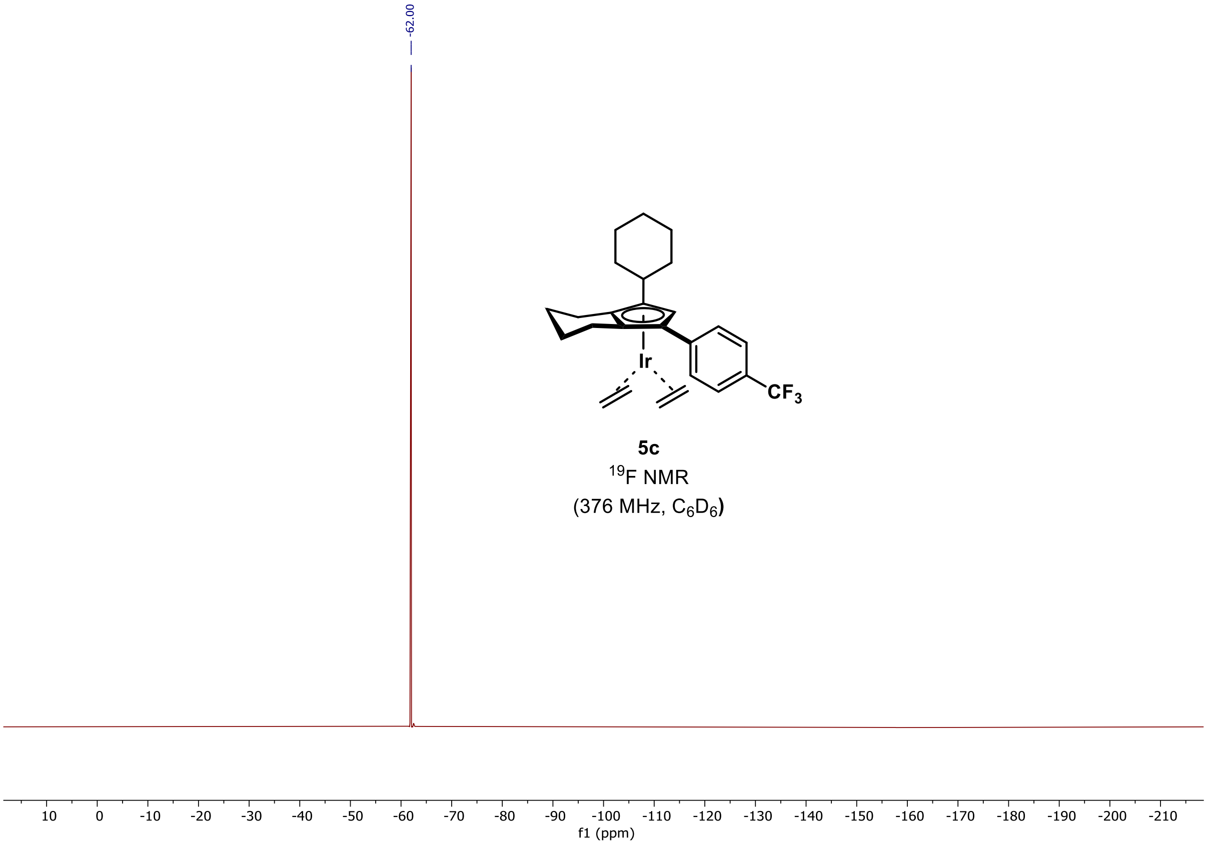
-62.00
Ir
CF3
5c
19F NMR
(376 MHz, C6D6)
10 0 -10 -20 -30 -40 -50 -60 -70 -80 -90 -100 -110 -120 -130 -140 -150 -160 -170 -180 -190 -200 -210
f1 (ppm)

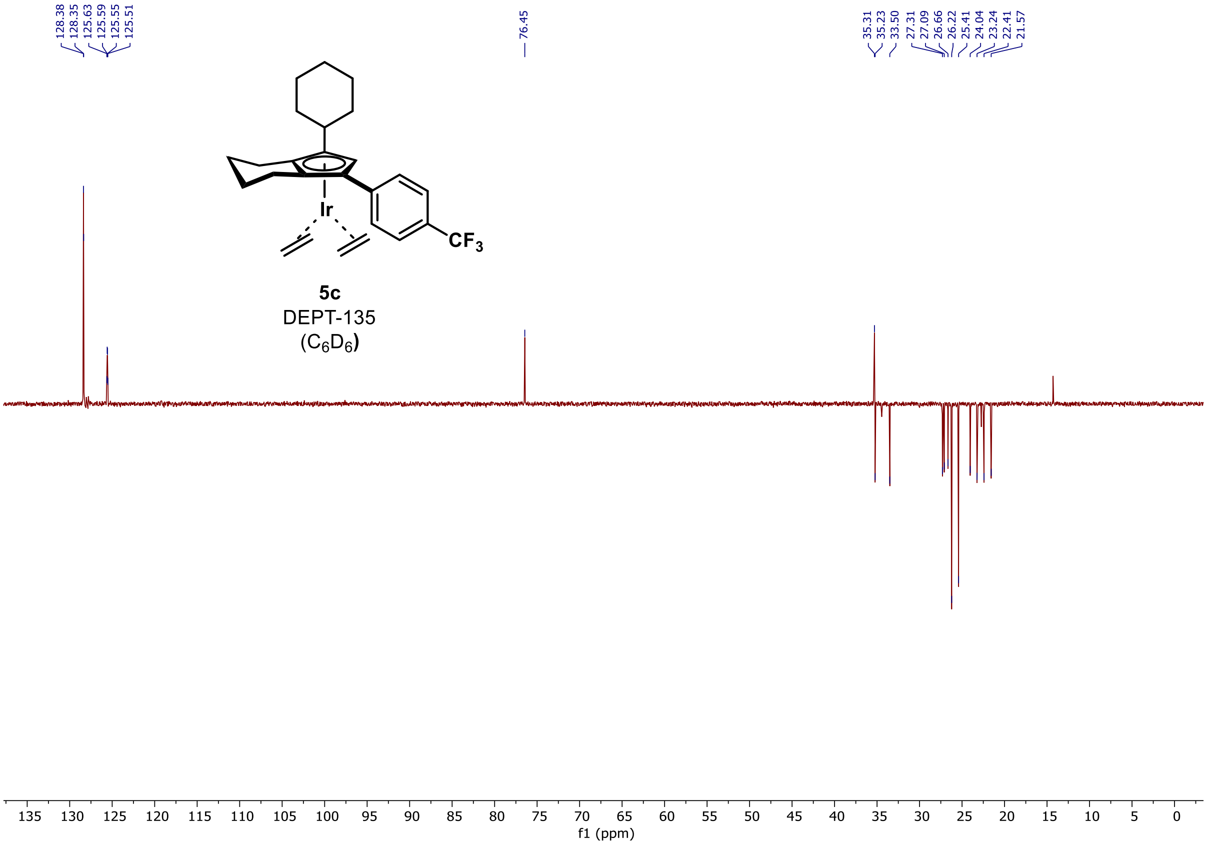
128.38
128.35
125.63
125.59
125.55
125.51
76.45
35.31
35.23
33.50
27.31
27.09
26.66
26.22
25.41
24.04
23.24
22.41
21.57
Ir
CF3
5c
DEPT-135
(C6D6)
135 130 125 120 115 110 105 100 95 90 85 80 75 70 65 60 55 50 45 40 35 30 25 20 15 10 5 0
f1 (ppm)

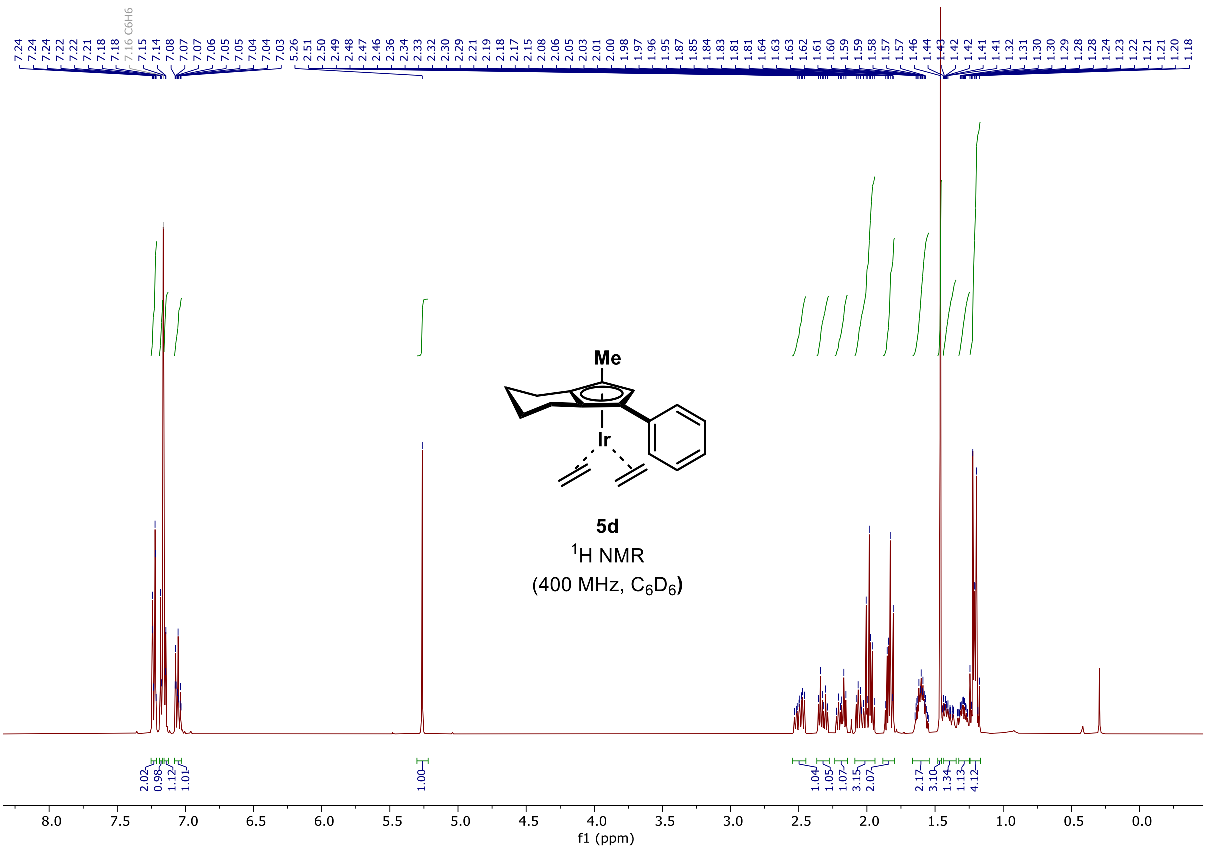
Me
Ir
5d
^{1}H NMR
(400 MHz, C_6D_6)
f1 (ppm)

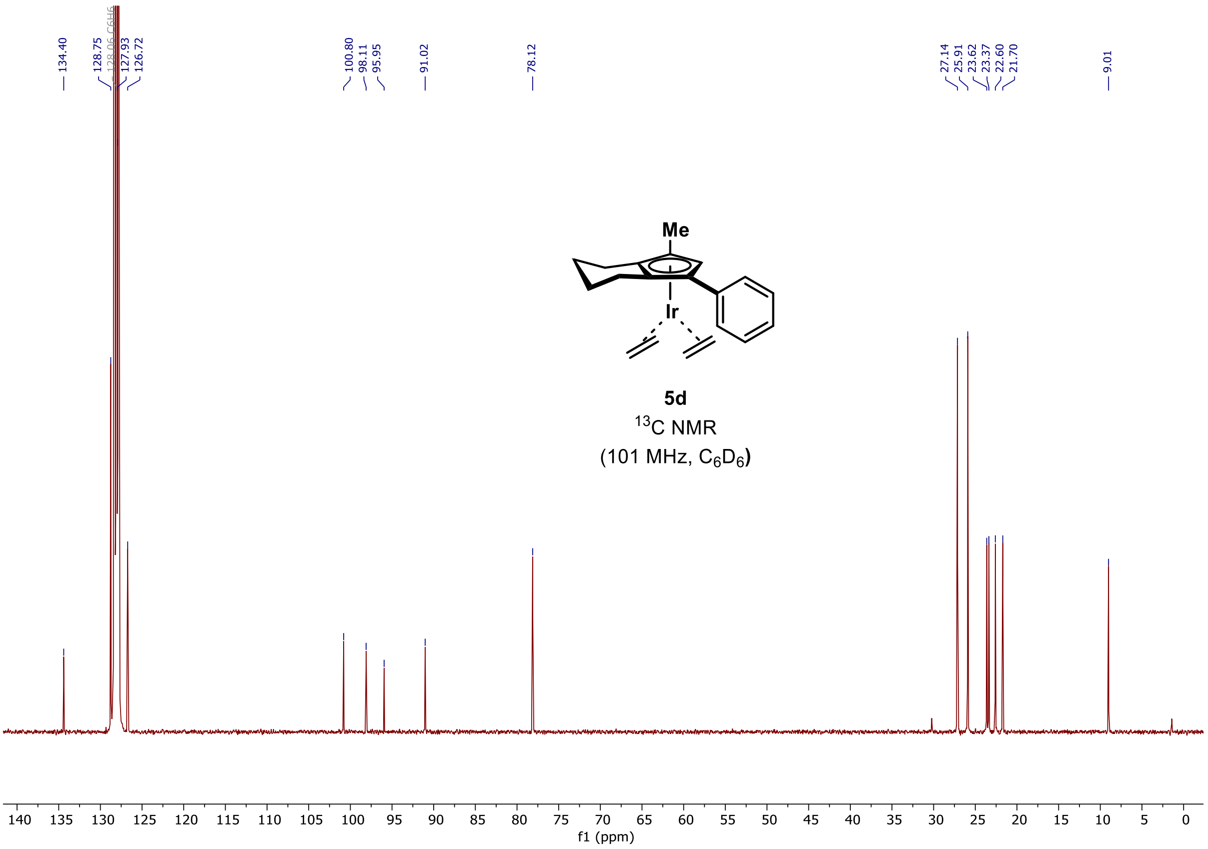
Me
Ir
5d
^{13}C NMR
(101 MHz, C_6D_6)
f1 (ppm)

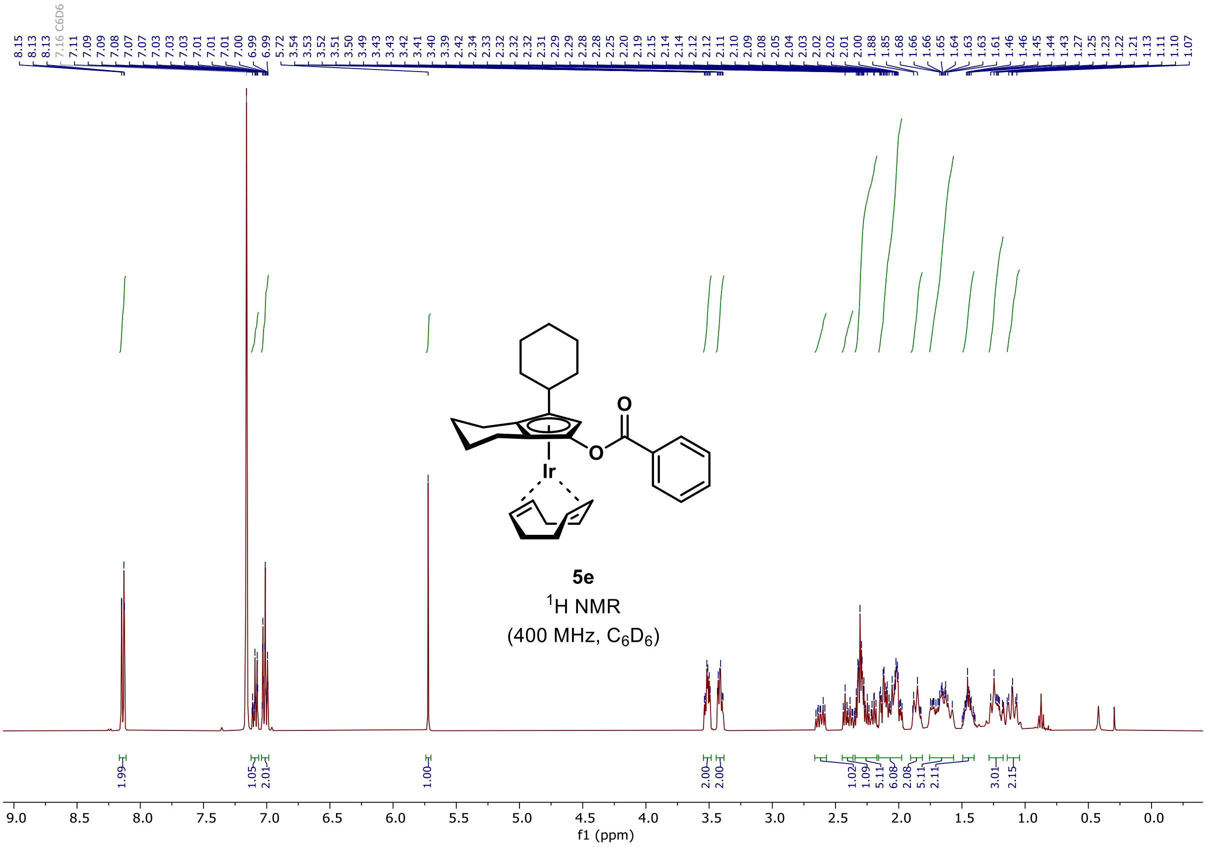
Ir
O
O
5e
^{1}H NMR
(400 MHz, C_6D_6)
f1 (ppm)

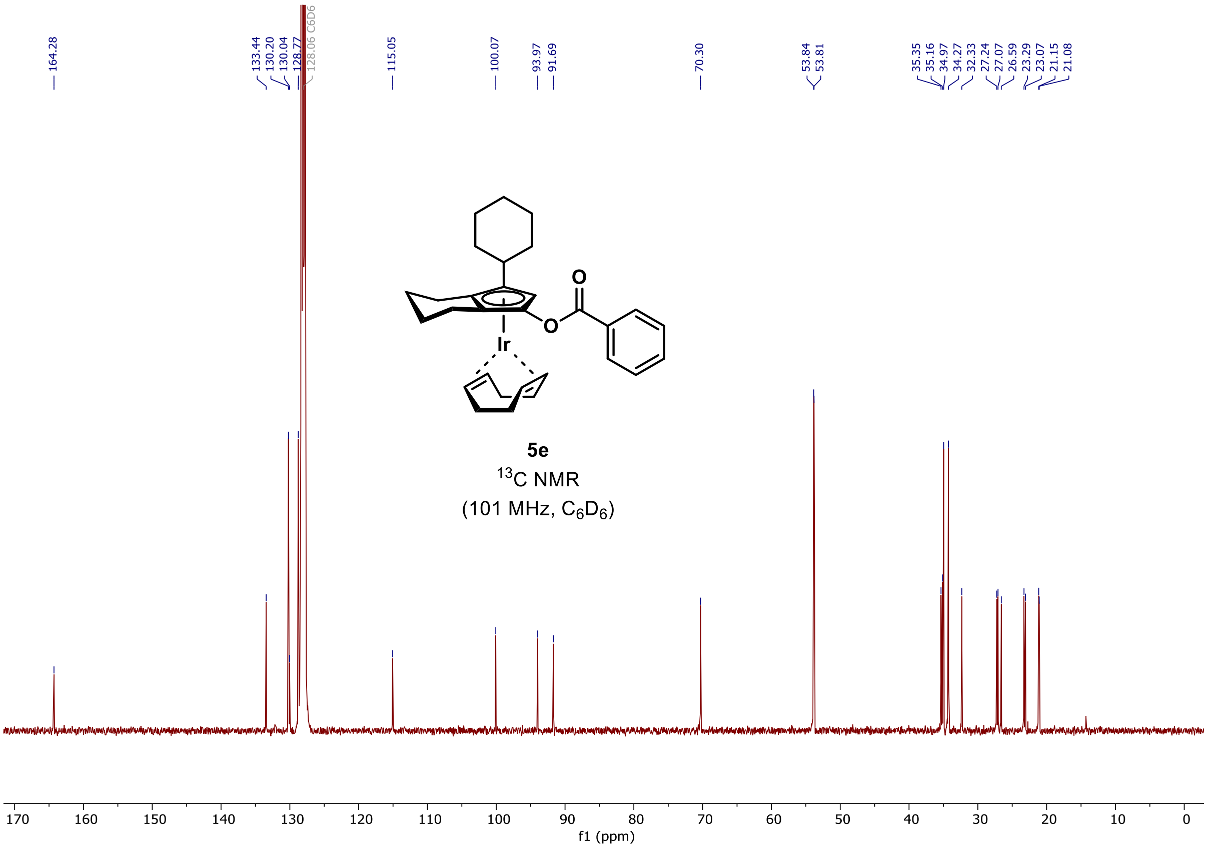
Ir
O
O
5e
^{13}C NMR
(101 MHz, C_6D_6)
f1 (ppm)

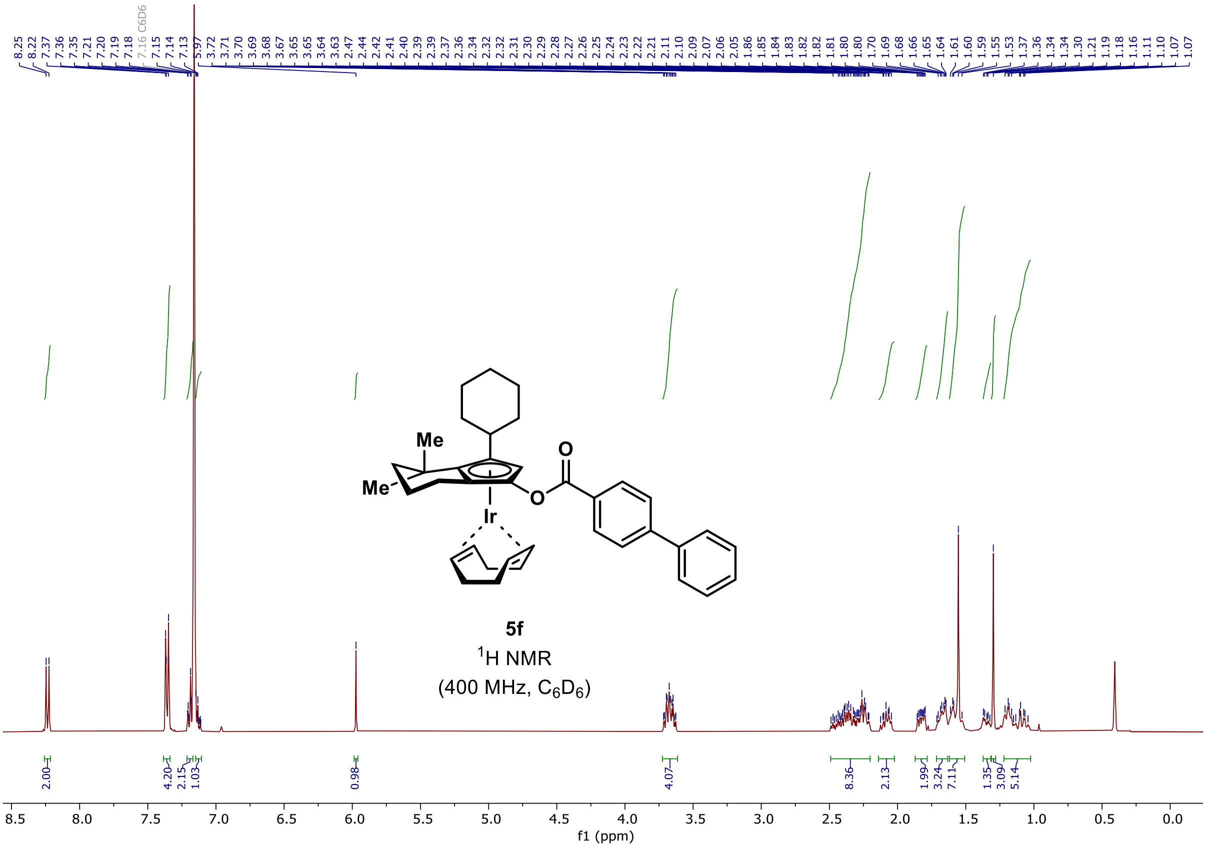
8.25 8.22 7.37 7.36 7.35 7.21 7.20 7.19 7.18 7.16 C6D6 7.15 7.14 7.13 5.97 3.72 3.71 3.70 3.69 3.68 3.67 3.65 3.65 3.64 3.63 2.47 2.44 2.42 2.41 2.40 2.39 2.39 2.37 2.36 2.34 2.32 2.32 2.31 2.30 2.29 2.28 2.27 2.26 2.25 2.24 2.23 2.22 2.21 2.11 2.10 2.09 2.07 2.06 2.05 1.86 1.85 1.84 1.83 1.82 1.81 1.80 1.80 1.70 1.69 1.68 1.66 1.65 1.64 1.61 1.60 1.59 1.55 1.53 1.37 1.36 1.34 1.30 1.21 1.19 1.18 1.16 1.11 1.10 1.07 1.07
Me
Me
O
O
Ir
5f
^{1}H NMR
(400 MHz, C_6D_6)
2.00 4.20 2.15 1.03 0.98 4.07 8.36 2.13 1.99 3.24 7.11 1.35 3.09 5.14
8.5 8.0 7.5 7.0 6.5 6.0 5.5 5.0 4.5 4.0 3.5 3.0 2.5 2.0 1.5 1.0 0.5 0.0
f1 (ppm)

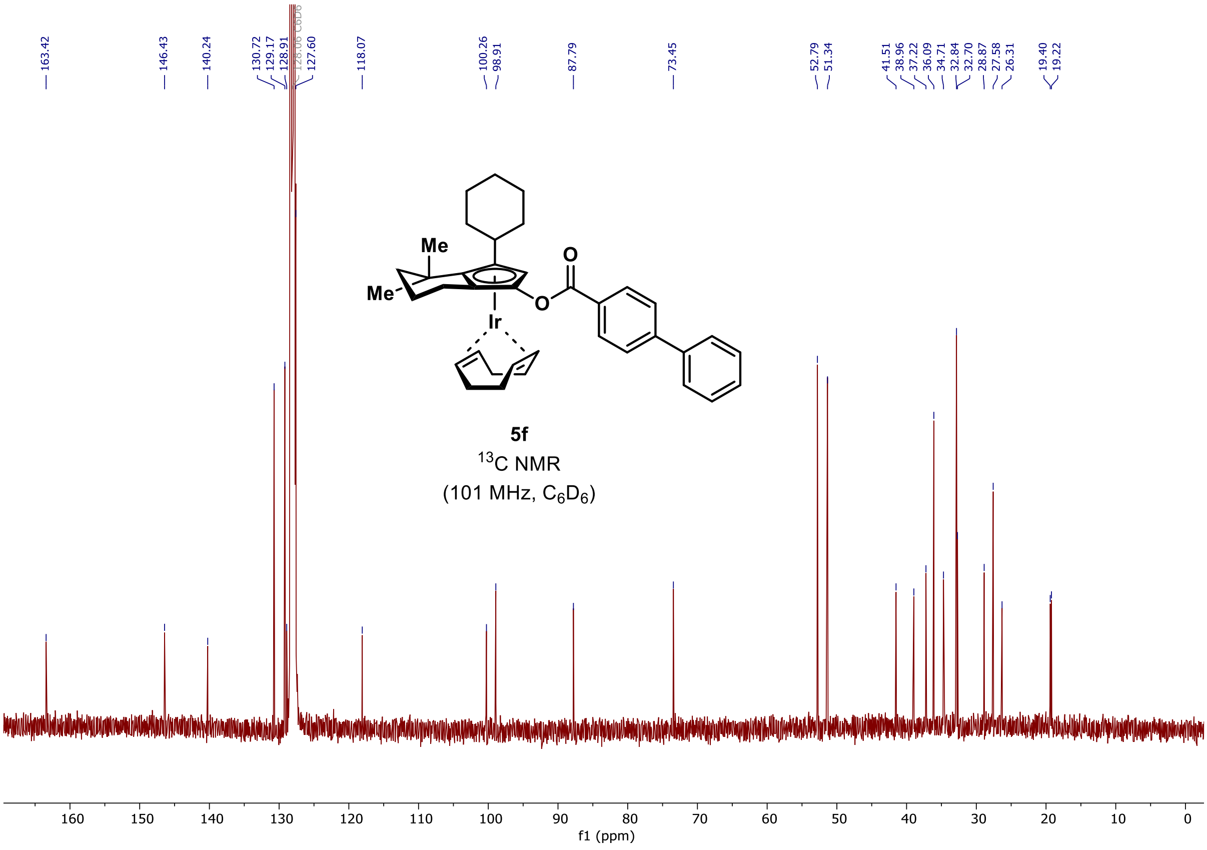
163.42 146.43 140.24 130.72 129.17 128.91 128.06 C6D6 127.60 118.07 100.26 98.91 87.79 73.45 52.79 51.34 41.51 38.96 37.22 36.09 34.71 32.84 32.70 28.87 27.58 26.31 19.40 19.22
Me
Me
O
O
Ir
5f
^{13}C NMR
(101 MHz, C_6D_6)
160 150 140 130 120 110 100 90 80 70 60 50 40 30 20 10 0
f1 (ppm)

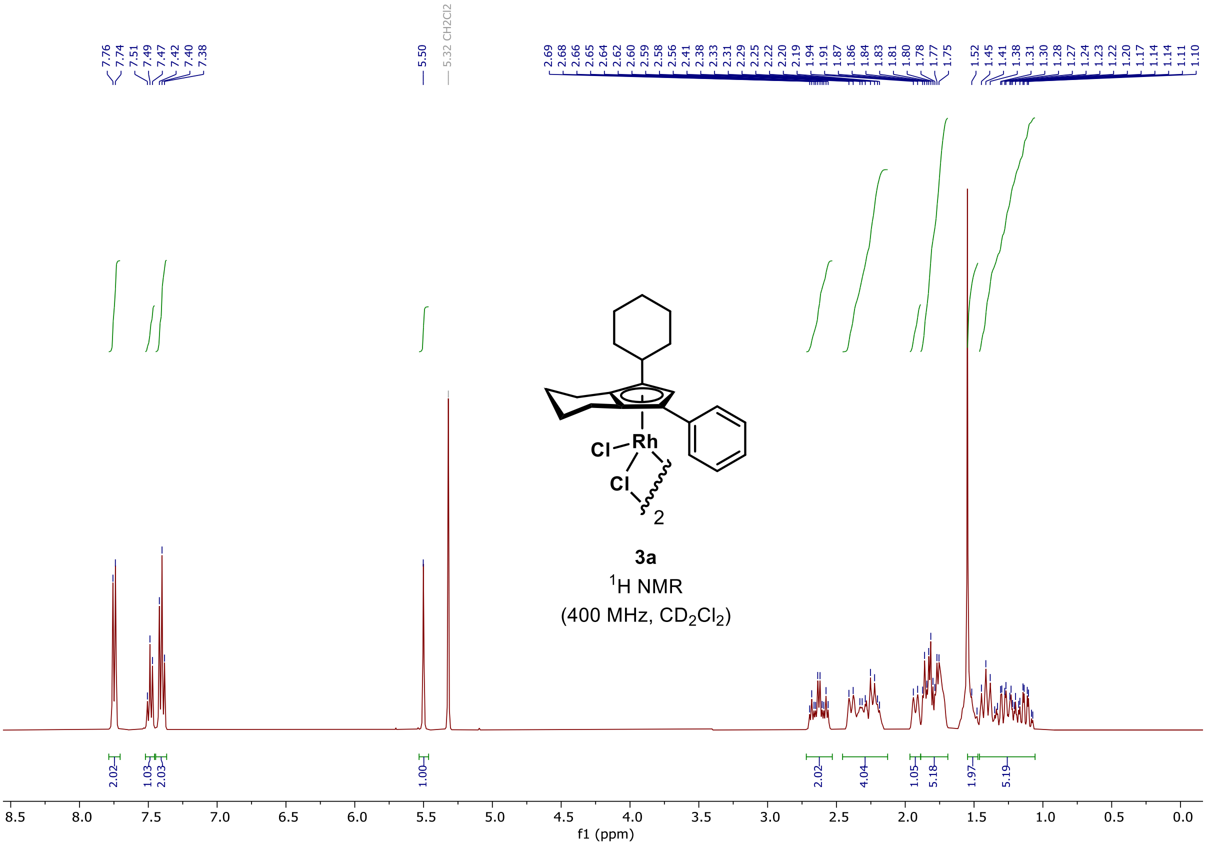
7.76
7.74
7.51
7.49
7.47
7.42
7.40
7.38
5.50
5.32 CH2Cl2
2.69
2.68
2.66
2.65
2.64
2.62
2.60
2.59
2.58
2.56
2.41
2.38
2.33
2.31
2.29
2.25
2.22
2.20
2.19
1.94
1.91
1.87
1.86
1.84
1.83
1.81
1.80
1.78
1.77
1.75
1.52
1.45
1.41
1.38
1.31
1.30
1.28
1.27
1.24
1.23
1.22
1.20
1.17
1.14
1.11
1.10
Cl
Rh
Cl
2
3a
^{1}H NMR
(400 MHz, CD_2Cl_2)
2.02
1.03
2.03
1.00
2.02
4.04
1.05
5.18
1.97
5.19
f1 (ppm)

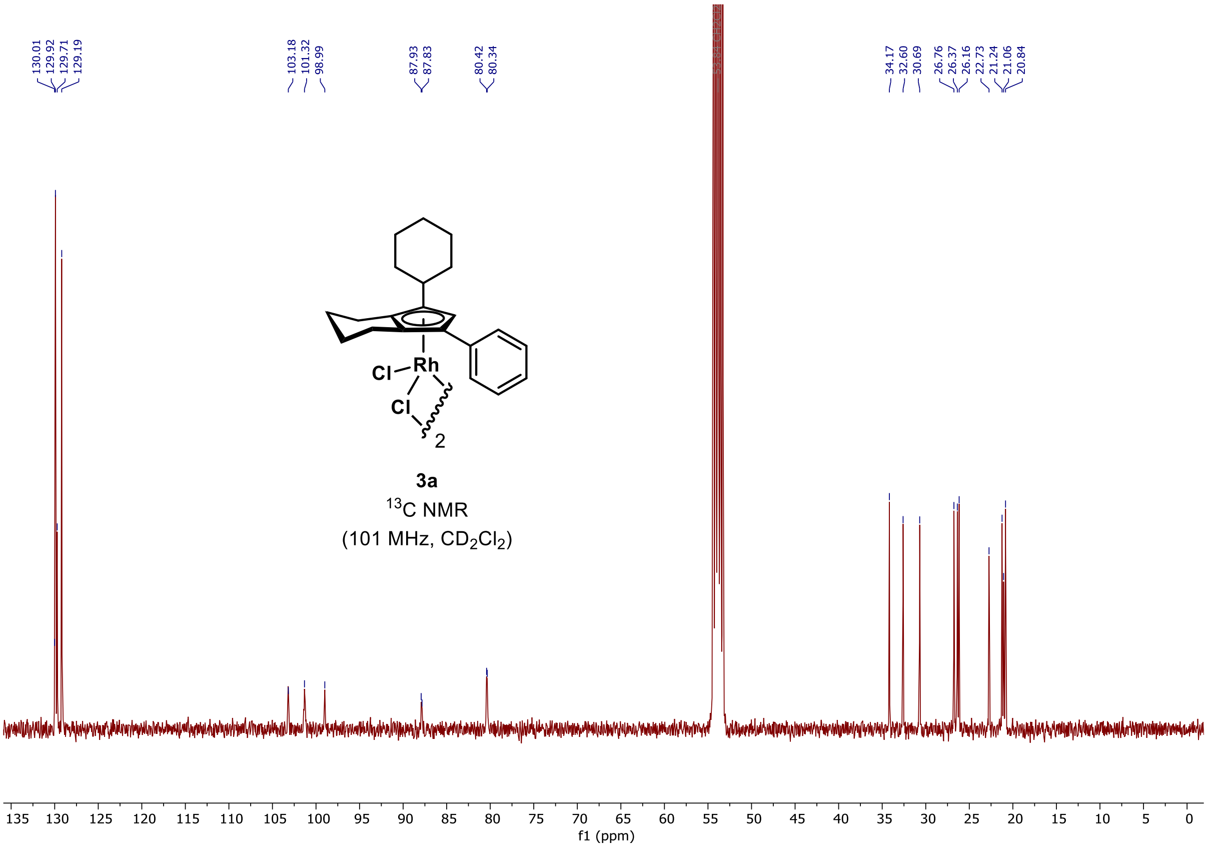
130.01
129.92
129.71
129.19
103.18
101.32
98.99
87.93
87.83
80.42
80.34
34.17
32.60
30.69
26.76
26.37
26.16
22.73
21.24
21.06
20.84
Cl
Rh
Cl
2
3a
^{13}C NMR
(101 MHz, CD_2Cl_2)
f1 (ppm)

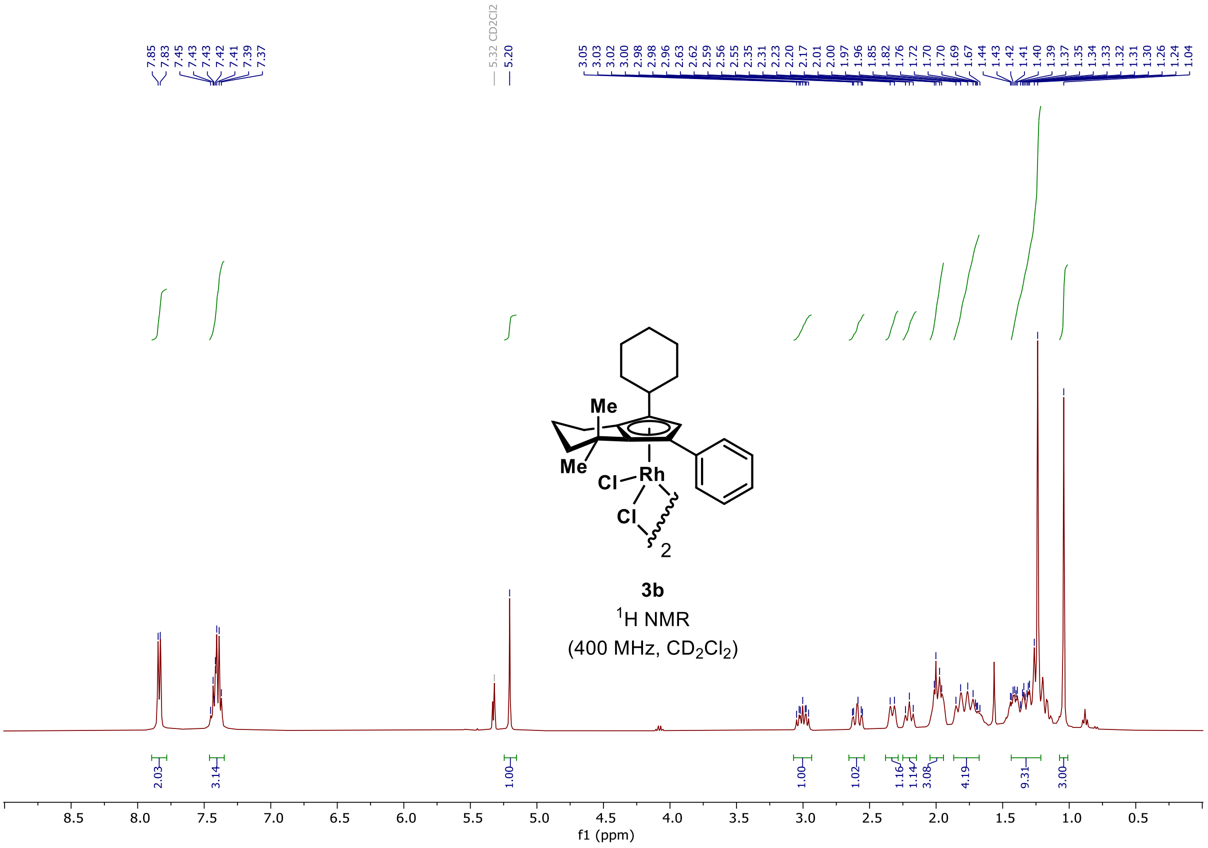

7.85
7.83
7.45
7.43
7.43
7.42
7.41
7.39
7.37
5.32 CD2Cl2
5.20
3.05
3.03
3.02
3.00
2.98
2.98
2.96
2.63
2.62
2.59
2.56
2.55
2.35
2.31
2.23
2.20
2.17
2.01
2.00
1.97
1.96
1.85
1.82
1.76
1.72
1.70
1.70
1.69
1.67
1.44
1.43
1.42
1.41
1.40
1.39
1.37
1.35
1.34
1.33
1.32
1.31
1.30
1.26
1.24
1.04
Me
Me
Cl
Rh
Cl
2
3b
¹H NMR
(400 MHz, CD₂Cl₂)
2.03
3.14
1.00
1.00
1.02
1.16
1.14
3.08
4.19
9.31
3.00
8.5
8.0
7.5
7.0
6.5
6.0
5.5
5.0
4.5
4.0
3.5
3.0
2.5
2.0
1.5
1.0
0.5
f1 (ppm)

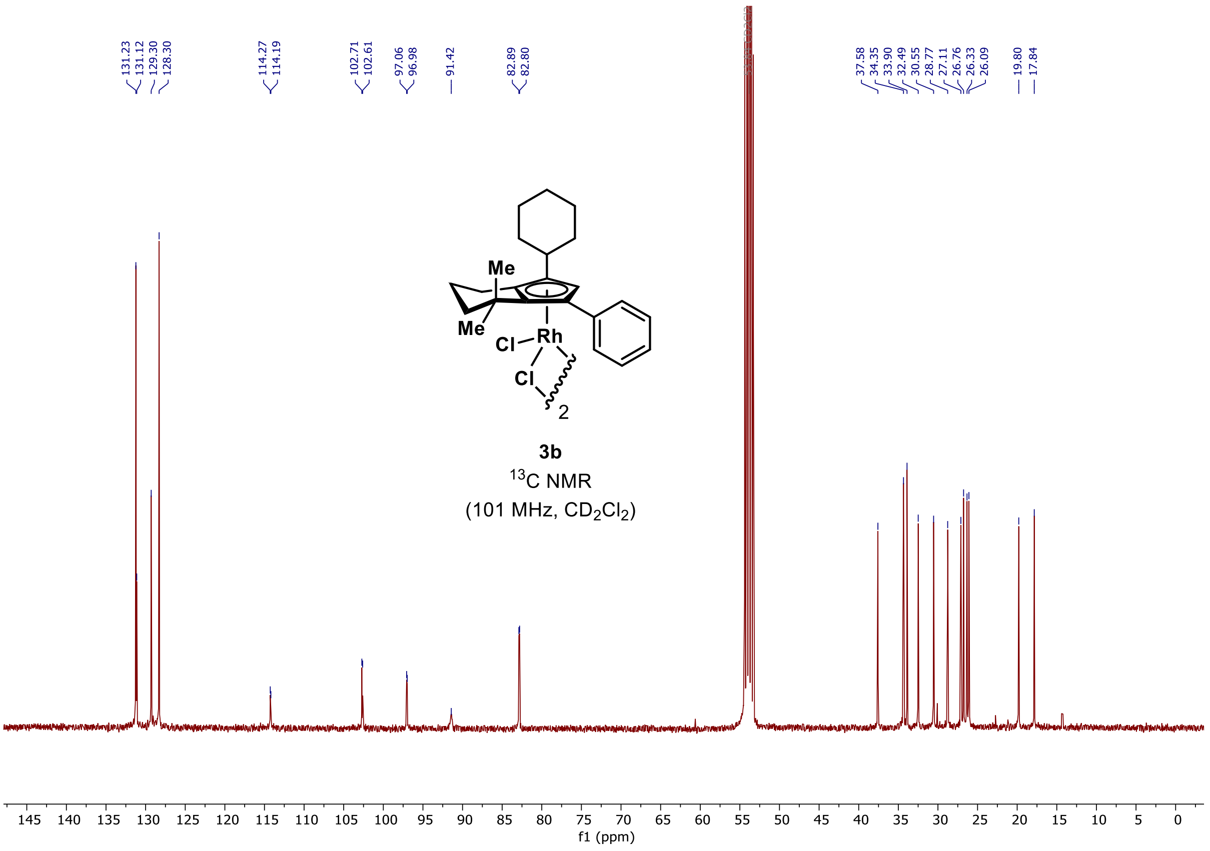

131.23
131.12
129.30
128.30
114.27
114.19
102.71
102.61
97.06
96.98
91.42
82.89
82.80
37.58
34.35
33.90
32.49
30.55
28.77
27.11
26.76
26.33
26.09
19.80
17.84
Me
Me
Cl
Rh
Cl
2
3b
¹³C NMR
(101 MHz, CD₂Cl₂)
145
140
135
130
125
120
115
110
105
100
95
90
85
80
75
70
65
60
55
50
45
40
35
30
25
20
15
10
5
0
f1 (ppm)

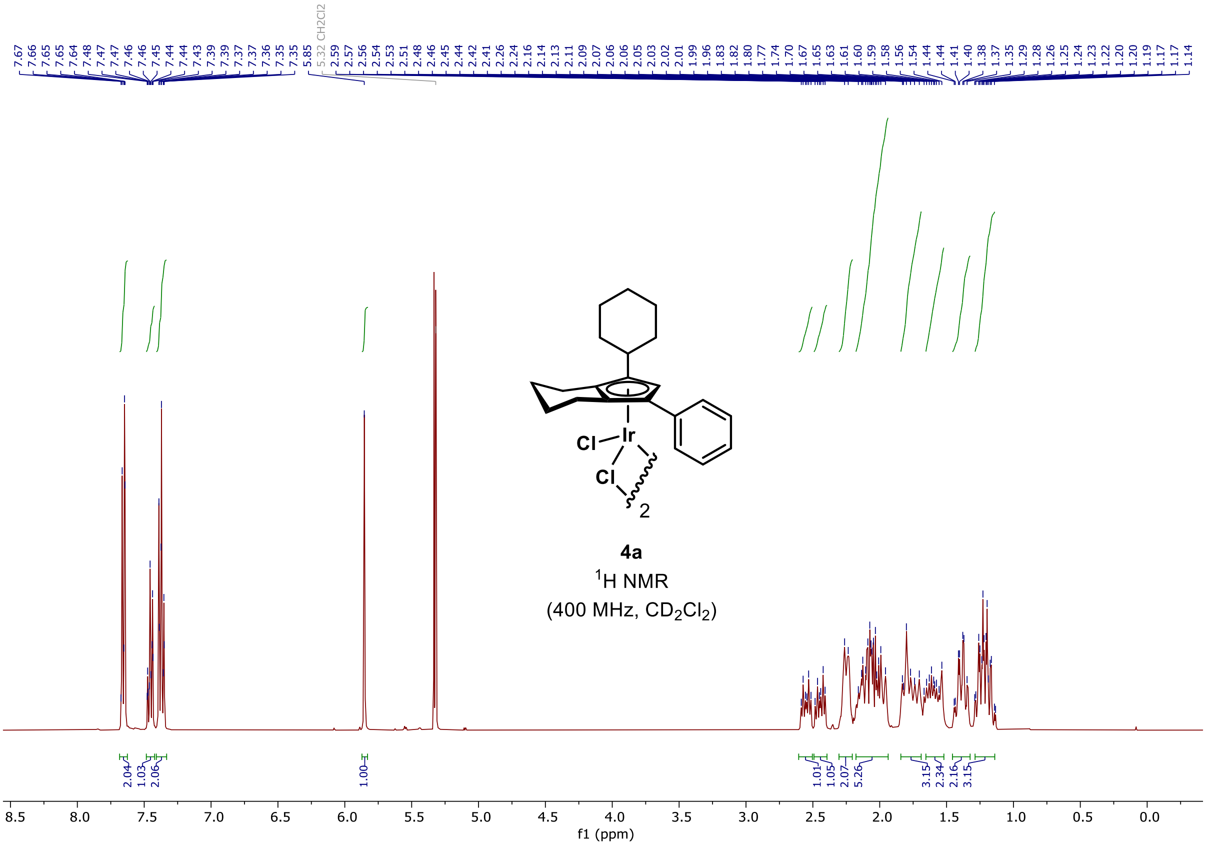
7.67 7.66 7.65 7.65 7.64 7.48 7.47 7.47 7.46 7.45 7.44 7.44 7.43 7.39 7.39 7.37 7.37 7.36 7.35 7.35 5.85 5.32 CH2Cl2 2.59 2.57 2.56 2.54 2.53 2.51 2.48 2.46 2.45 2.44 2.42 2.41 2.26 2.24 2.16 2.14 2.13 2.11 2.09 2.07 2.06 2.05 2.03 2.02 2.01 1.99 1.96 1.83 1.82 1.80 1.77 1.74 1.70 1.67 1.65 1.63 1.61 1.60 1.59 1.58 1.56 1.54 1.44 1.44 1.41 1.40 1.38 1.37 1.35 1.29 1.28 1.26 1.25 1.24 1.23 1.22 1.20 1.19 1.17 1.14
Cl
Ir
Cl
2
4a
1H NMR
(400 MHz, CD2Cl2)
2.04 1.03 2.06 1.00 1.01 1.05 2.07 5.26 3.15 2.34 2.16 3.15
8.5 8.0 7.5 7.0 6.5 6.0 5.5 5.0 4.5 4.0 3.5 3.0 2.5 2.0 1.5 1.0 0.5 0.0
f1 (ppm)

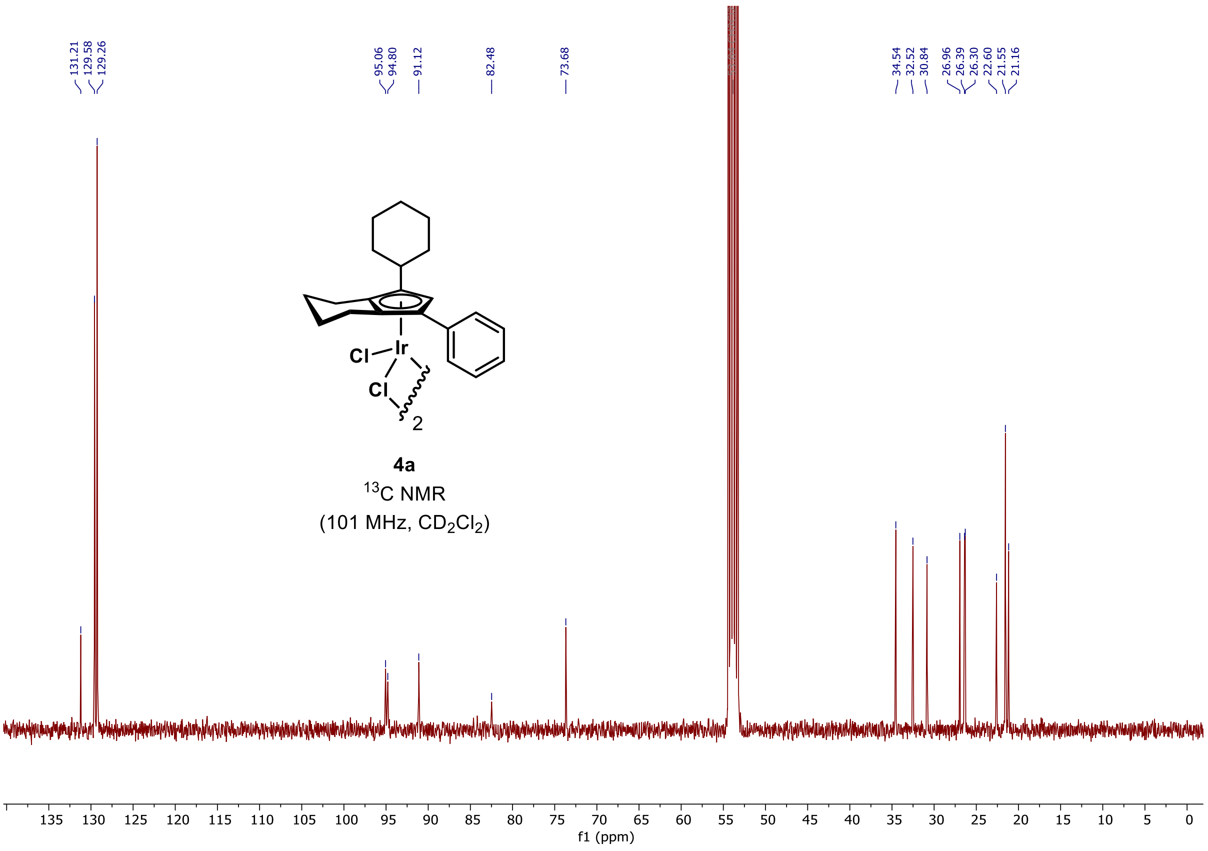
131.21 129.58 129.26 95.06 94.80 91.12 82.48 73.68 34.54 32.52 30.84 26.96 26.39 26.30 22.60 21.55 21.16
Cl
Ir
Cl
2
4a
13C NMR
(101 MHz, CD2Cl2)
135 130 125 120 115 110 105 100 95 90 85 80 75 70 65 60 55 50 45 40 35 30 25 20 15 10 5 0
f1 (ppm)

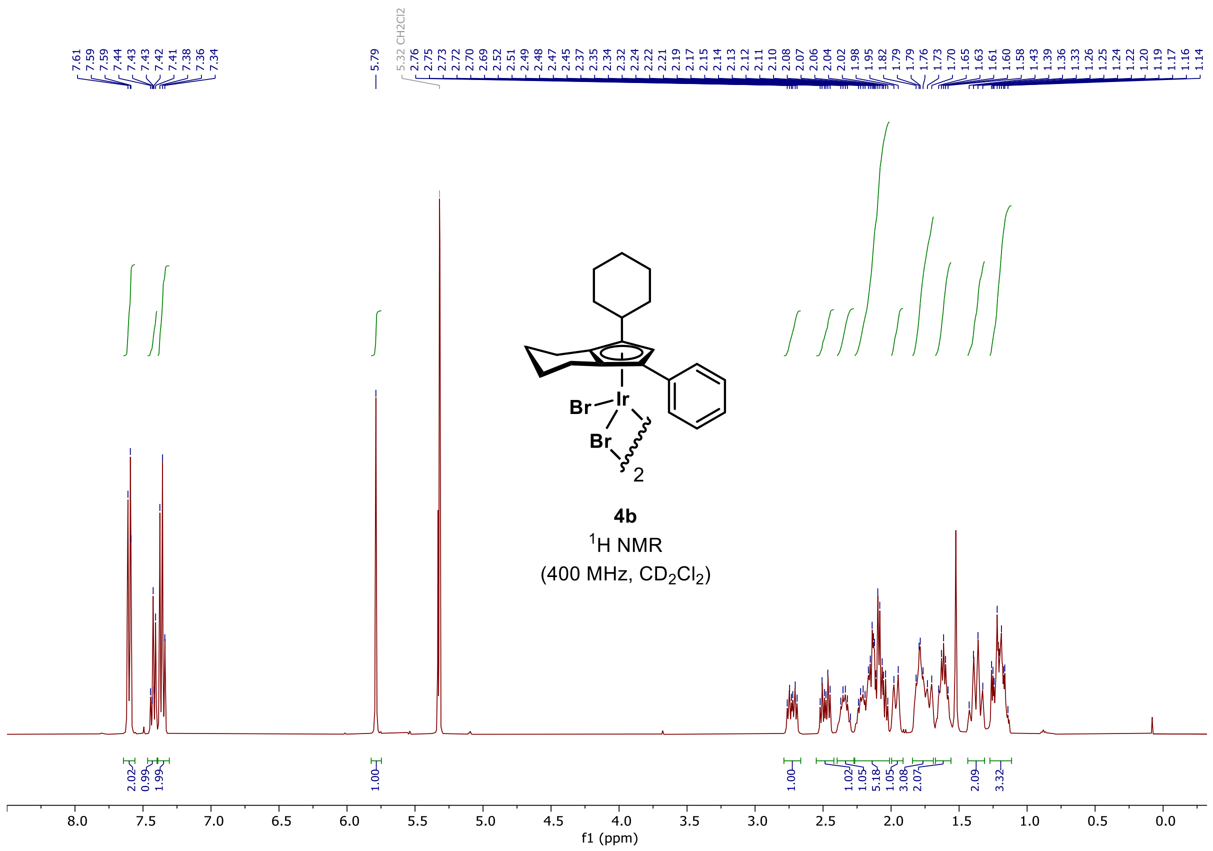
4b
^{1}H NMR
(400 MHz, CD_2Cl_2)
f1 (ppm)

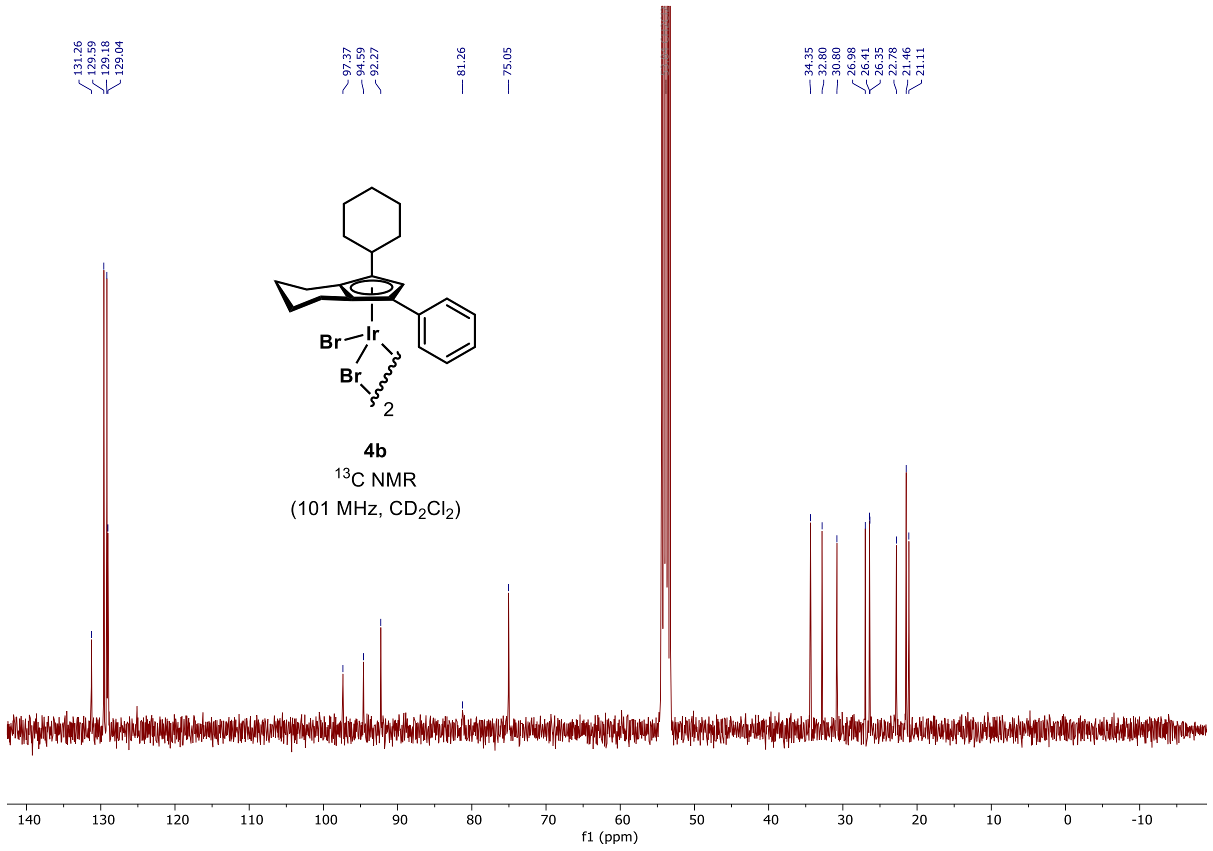
4b
^{13}C NMR
(101 MHz, CD_2Cl_2)
f1 (ppm)

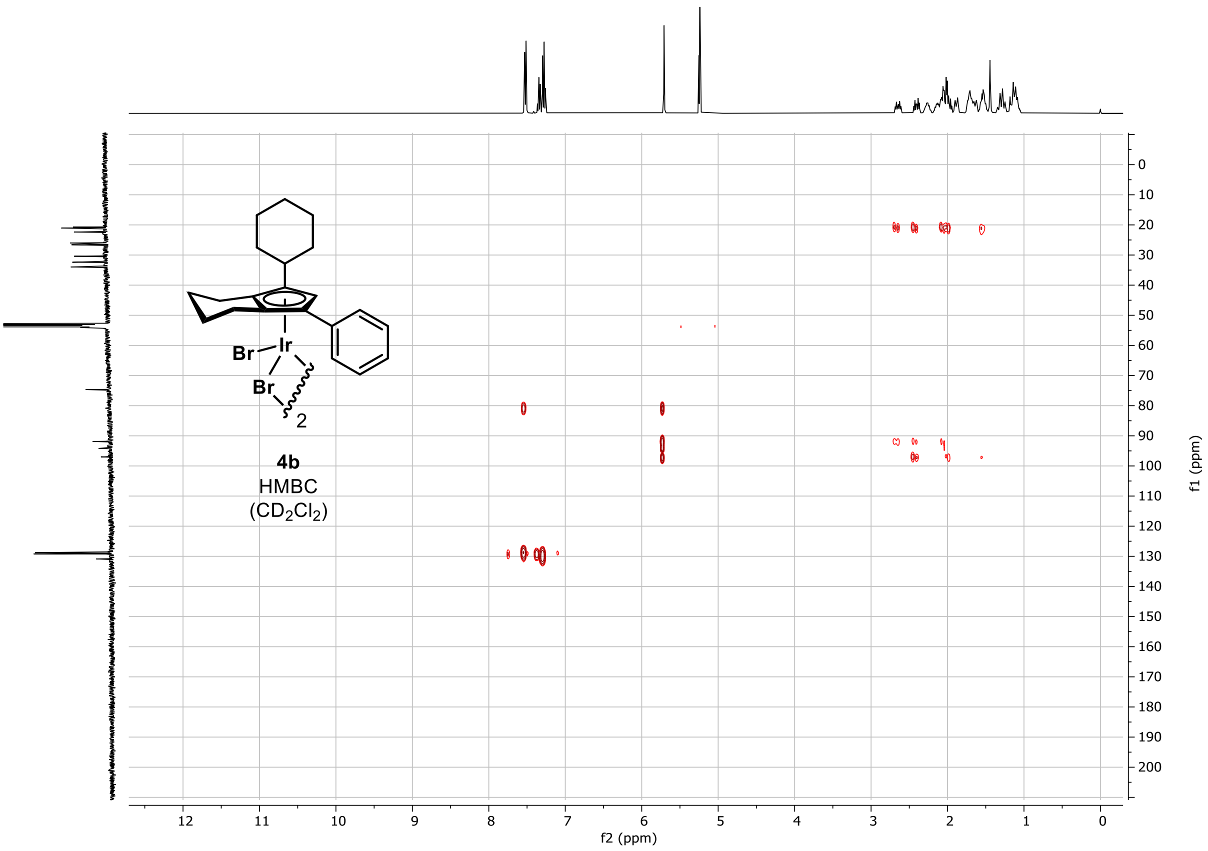
Br
Ir
Br
2
4b
HMBC
(CD2Cl2)
f2 (ppm)
f1 (ppm)

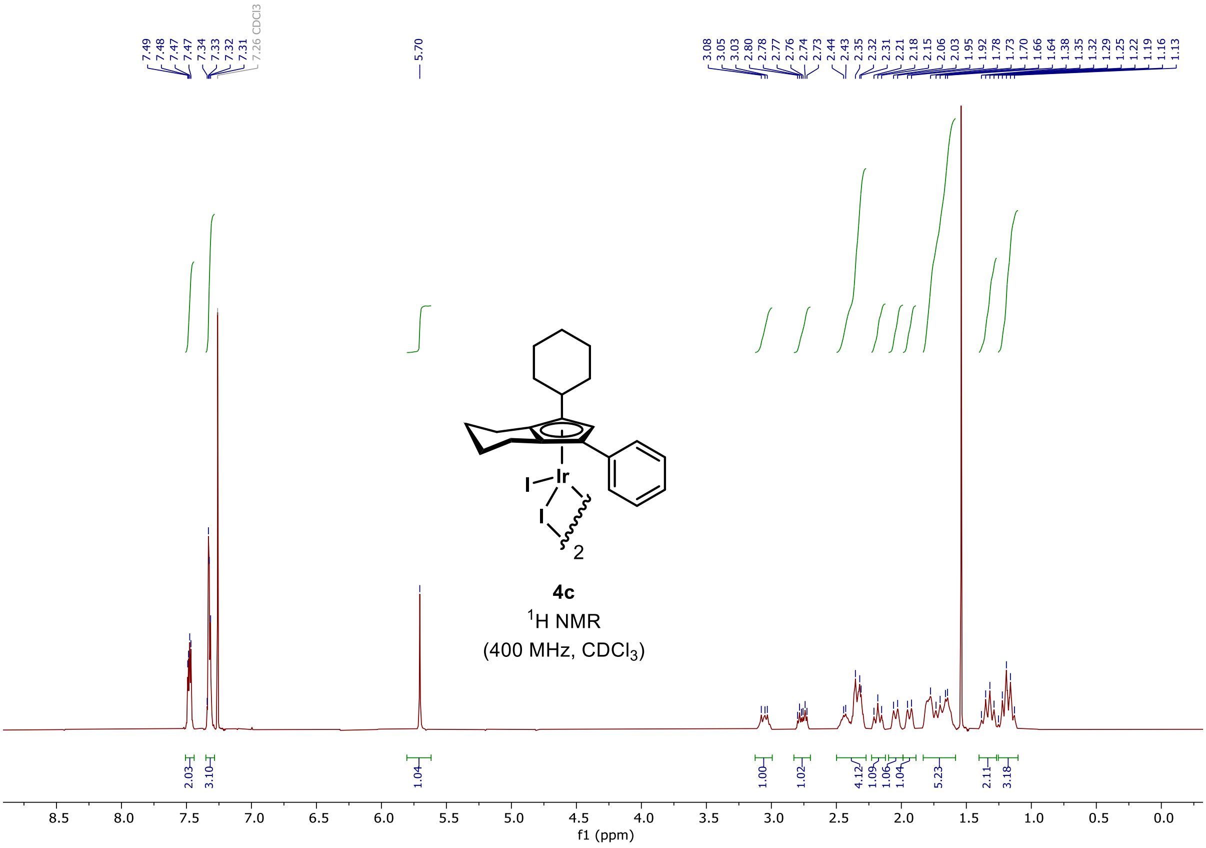

4c

^{1}H NMR

(400 MHz, $CDCl_3$)

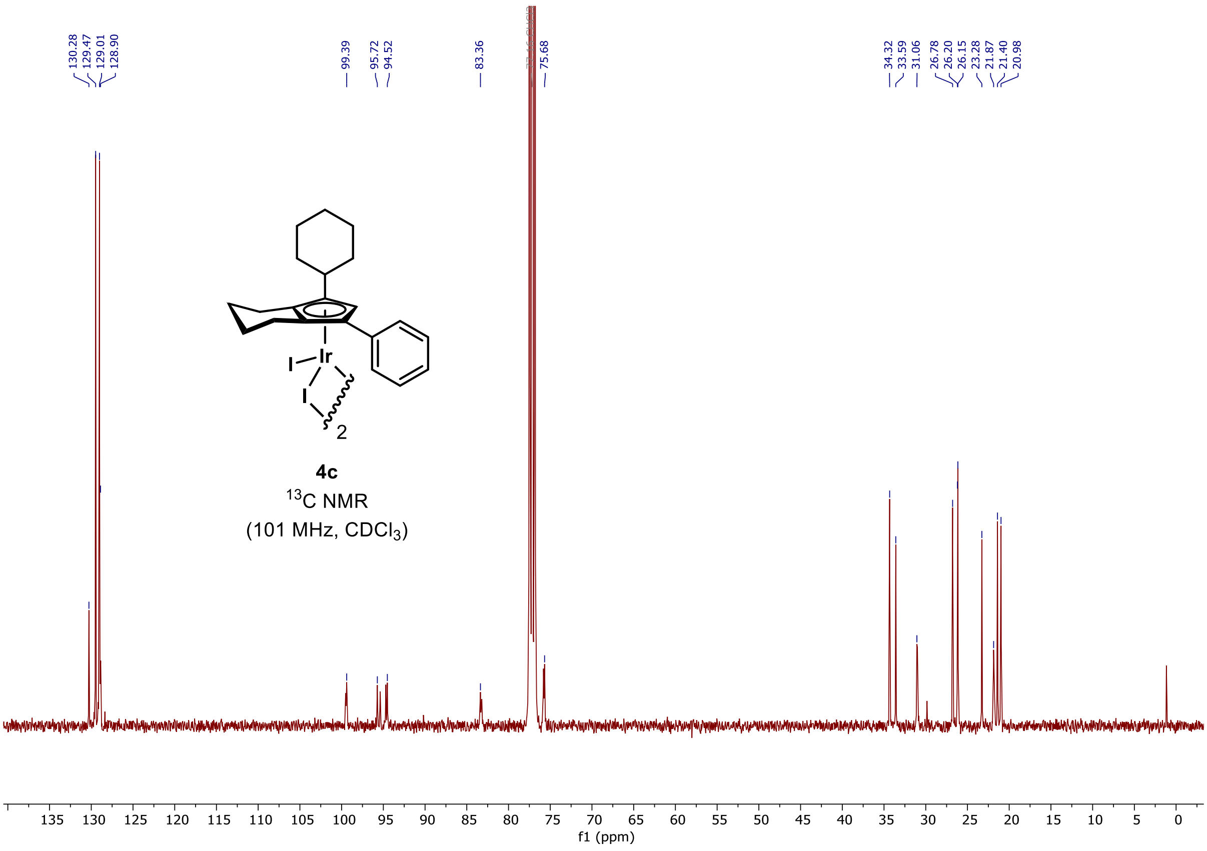

4c

^{13}C NMR

(101 MHz, $CDCl_3$)

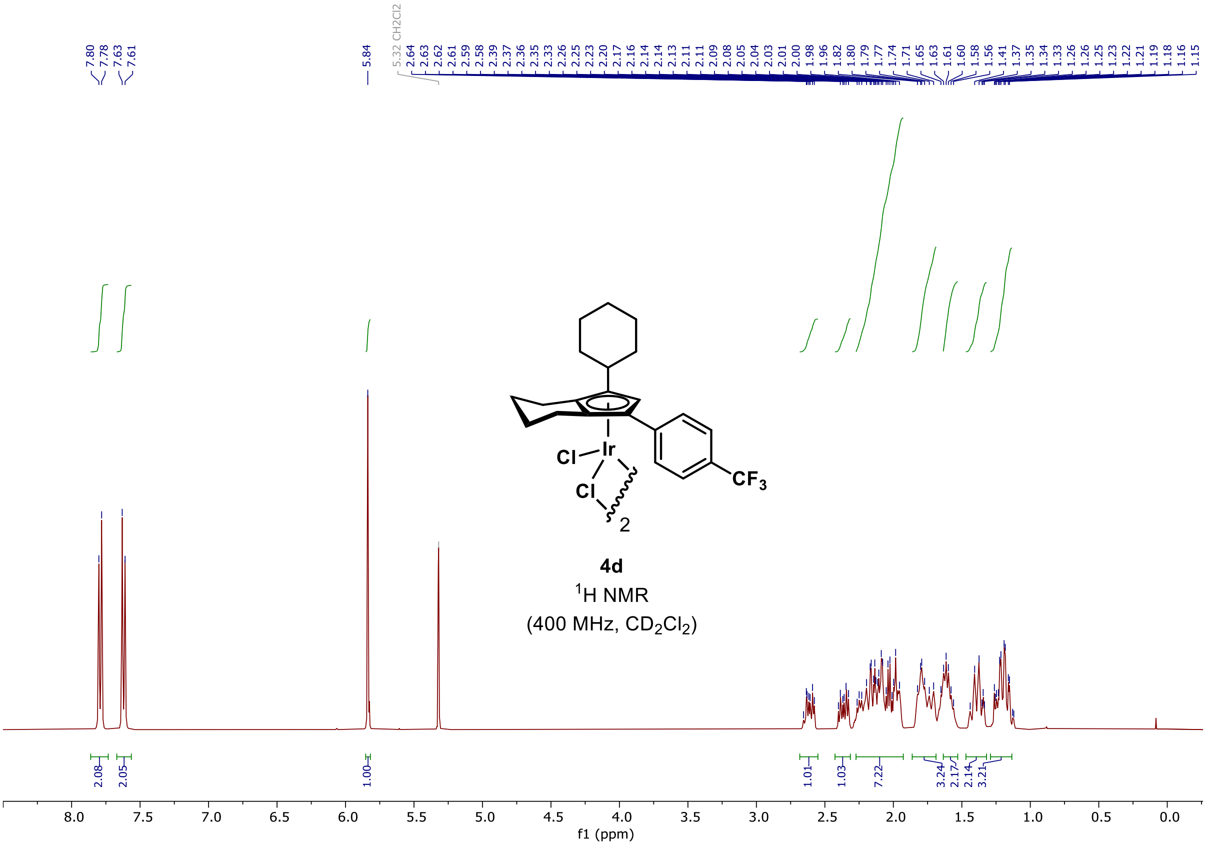

4d
^{1}H NMR
(400 MHz, CD_2Cl_2)

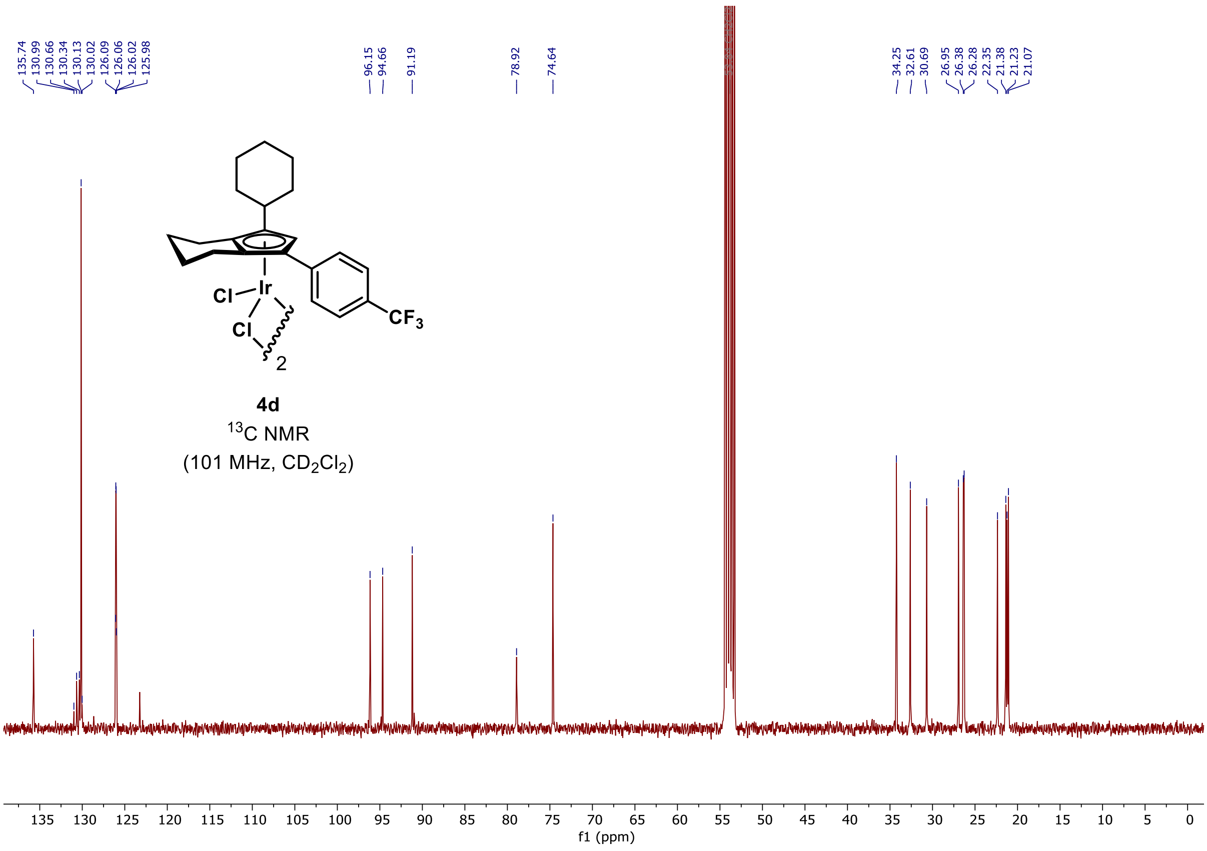

4d
^{13}C NMR
(101 MHz, CD_2Cl_2)

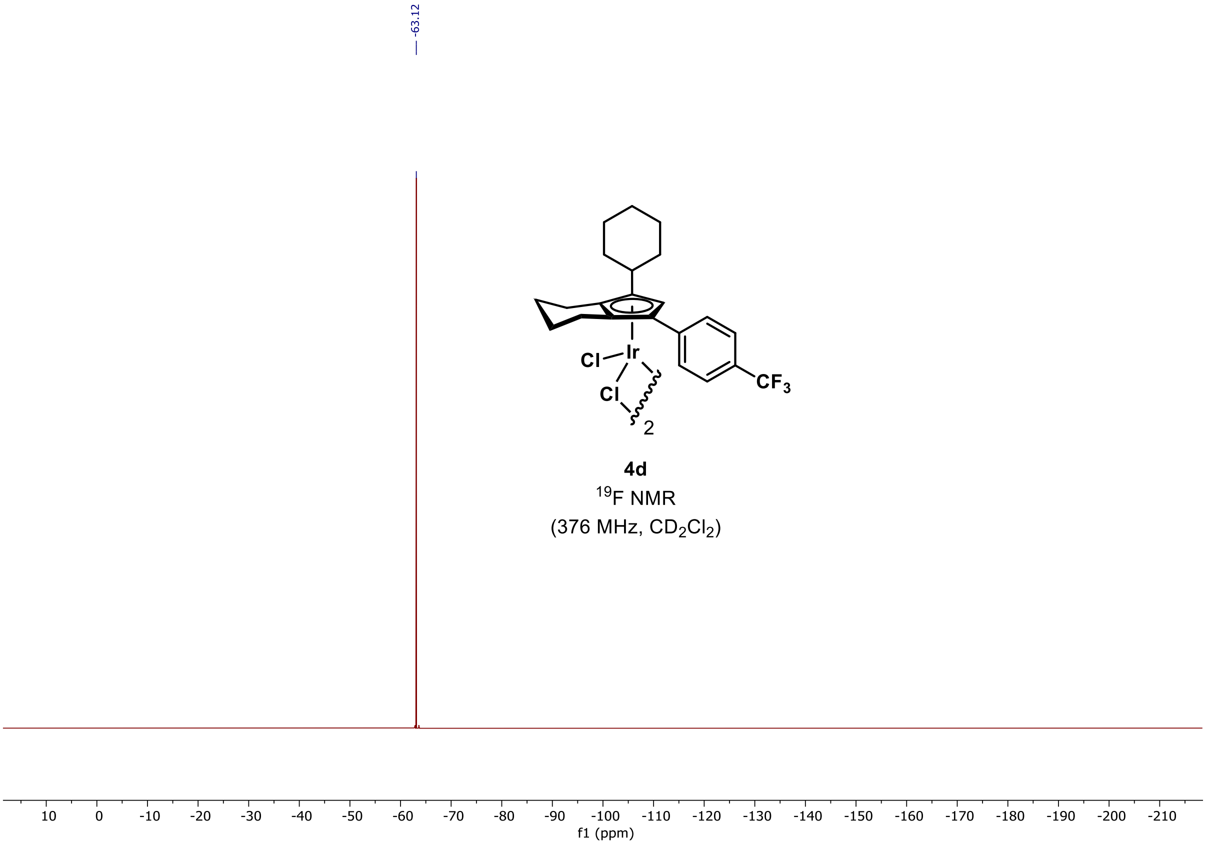
-63.12
Cl
Ir
Cl
2
CF_3
4d
^{19}F NMR
(376 MHz, CD_2Cl_2)
10 0 -10 -20 -30 -40 -50 -60 -70 -80 -90 -100 -110 -120 -130 -140 -150 -160 -170 -180 -190 -200 -210
f1 (ppm)

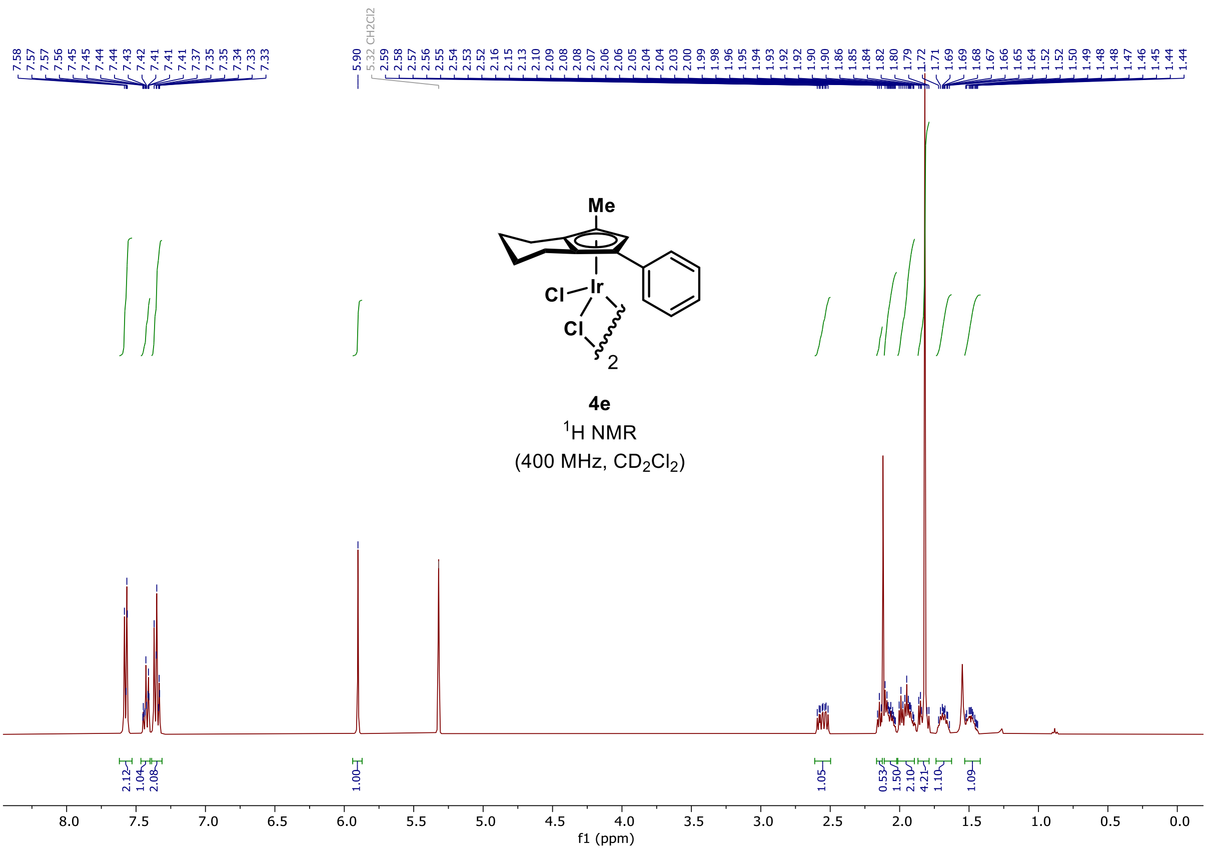
Me
Cl
Ir
Cl
2
4e
^{1}H NMR
(400 MHz, CD_2Cl_2)
f1 (ppm)

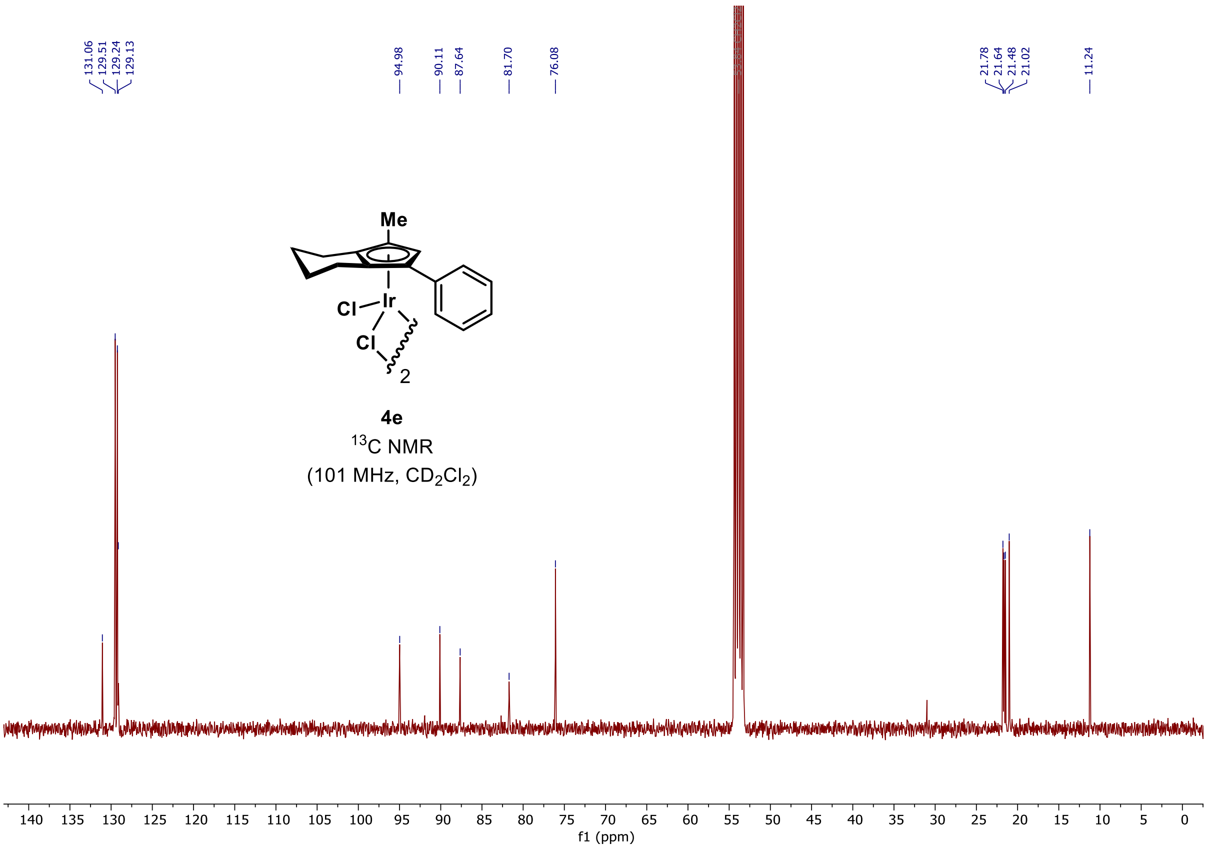
Me
Cl
Ir
Cl
2
4e
^{13}C NMR
(101 MHz, CD_2Cl_2)
f1 (ppm)

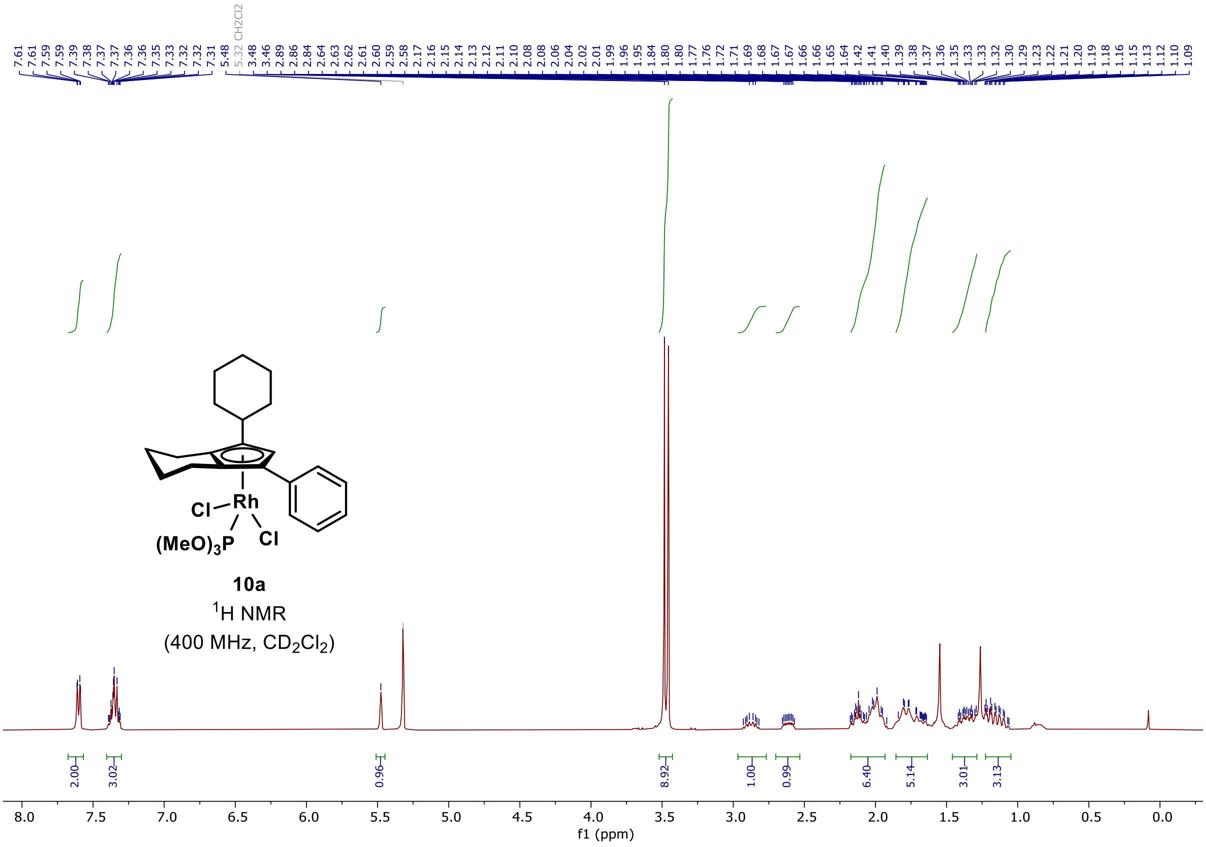
7.61 7.61 7.59 7.59 7.39 7.38 7.37 7.37 7.36 7.36 7.35 7.33 7.32 7.32 7.31 5.48 5.32 CH2Cl2 3.48 3.46 2.89 2.86 2.84 2.64 2.63 2.62 2.61 2.60 2.59 2.58 2.17 2.16 2.15 2.14 2.13 2.12 2.11 2.10 2.08 2.08 2.06 2.04 2.02 2.01 1.99 1.96 1.95 1.84 1.80 1.80 1.77 1.76 1.72 1.71 1.69 1.68 1.67 1.67 1.66 1.65 1.64 1.42 1.41 1.40 1.39 1.38 1.37 1.36 1.35 1.33 1.33 1.32 1.30 1.29 1.23 1.22 1.21 1.20 1.19 1.18 1.16 1.15 1.13 1.12 1.10 1.09
Cl
Rh
Cl
(MeO)3P
10a
1H NMR
(400 MHz, CD2Cl2)
2.00 3.02 0.96 8.92 1.00 0.99 6.40 5.14 3.01 3.13
8.0 7.5 7.0 6.5 6.0 5.5 5.0 4.5 4.0 3.5 3.0 2.5 2.0 1.5 1.0 0.5 0.0
f1 (ppm)

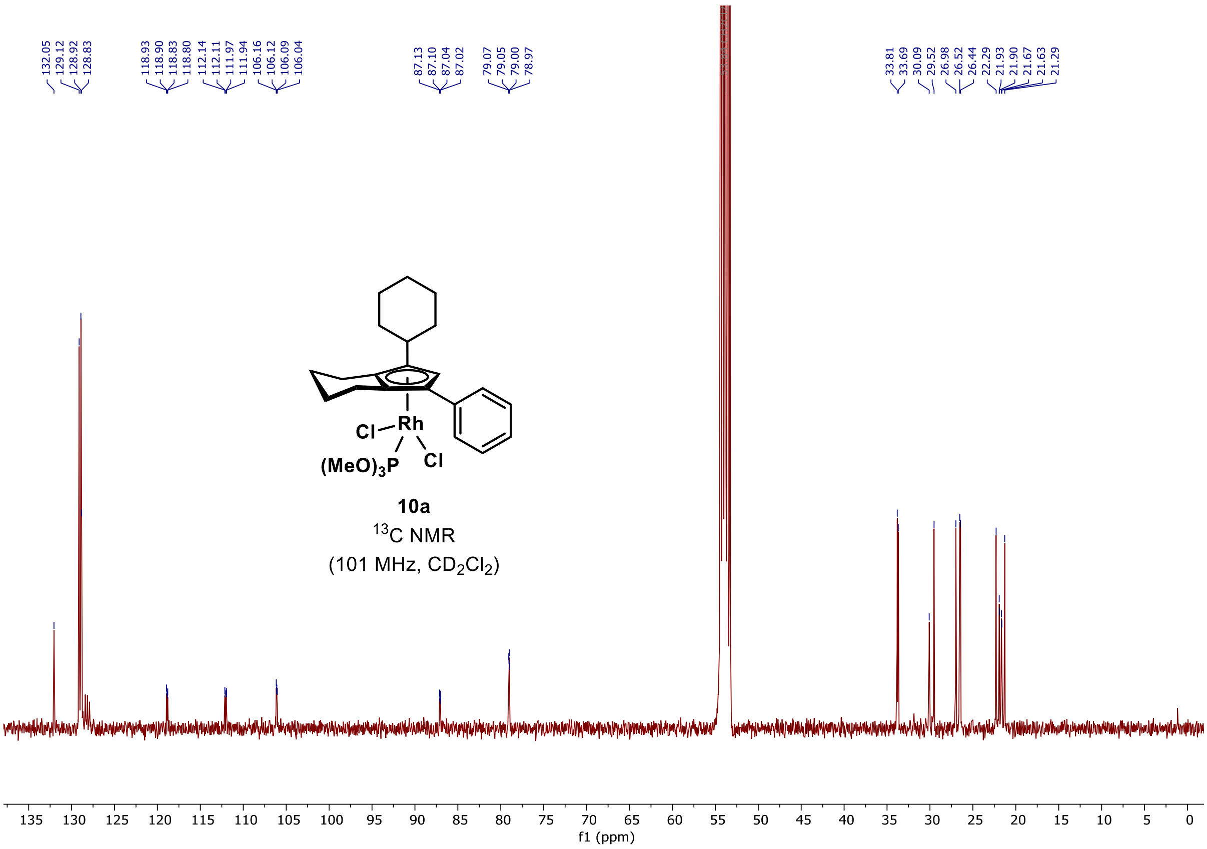
132.05 129.12 128.92 128.83 118.93 118.90 118.83 118.80 112.14 112.11 111.97 111.94 106.16 106.12 106.09 106.04 87.13 87.10 87.04 87.02 79.07 79.05 79.00 78.97 33.81 33.69 30.09 29.52 26.98 26.52 26.44 22.29 21.93 21.90 21.67 21.63 21.29
Cl
Rh
Cl
(MeO)3P
10a
13C NMR
(101 MHz, CD2Cl2)
135 130 125 120 115 110 105 100 95 90 85 80 75 70 65 60 55 50 45 40 35 30 25 20 15 10 5 0
f1 (ppm)

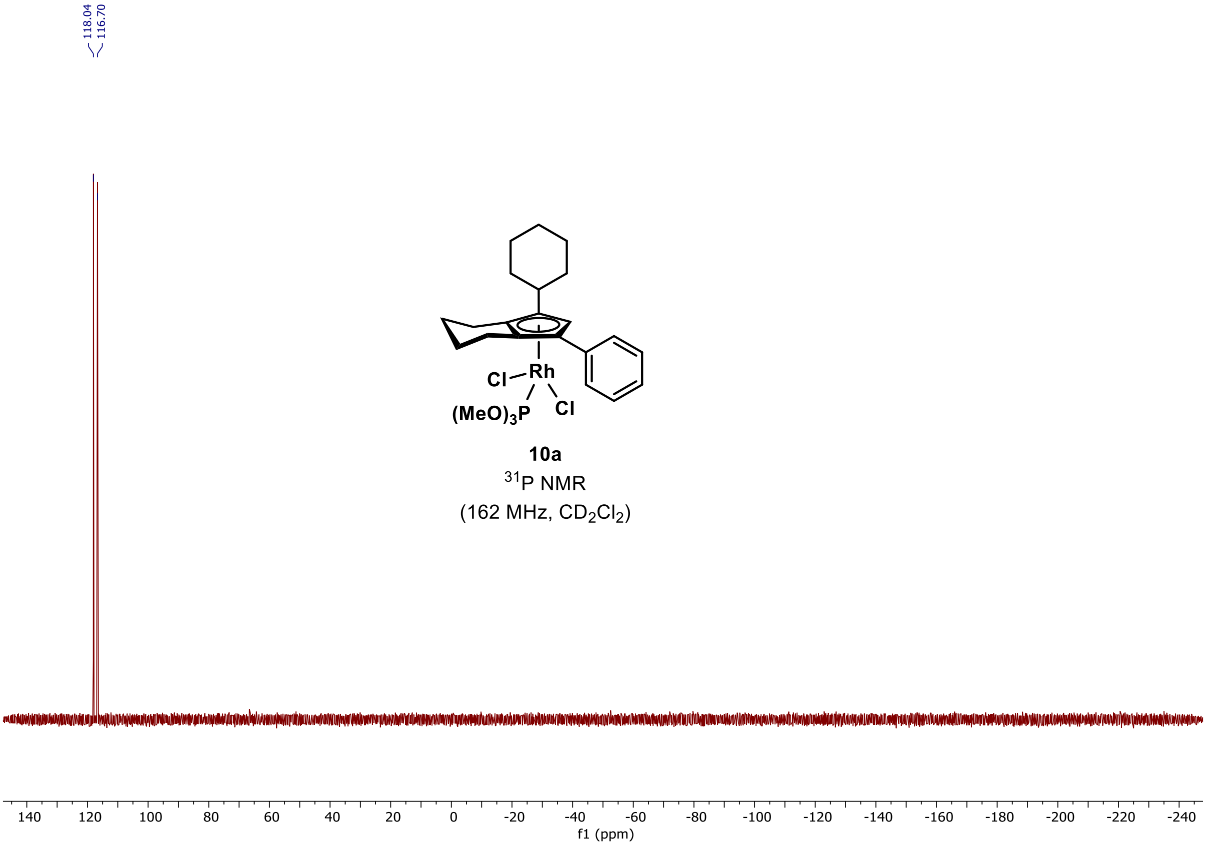

10a

^{31}P NMR

(162 MHz, CD_2Cl_2)

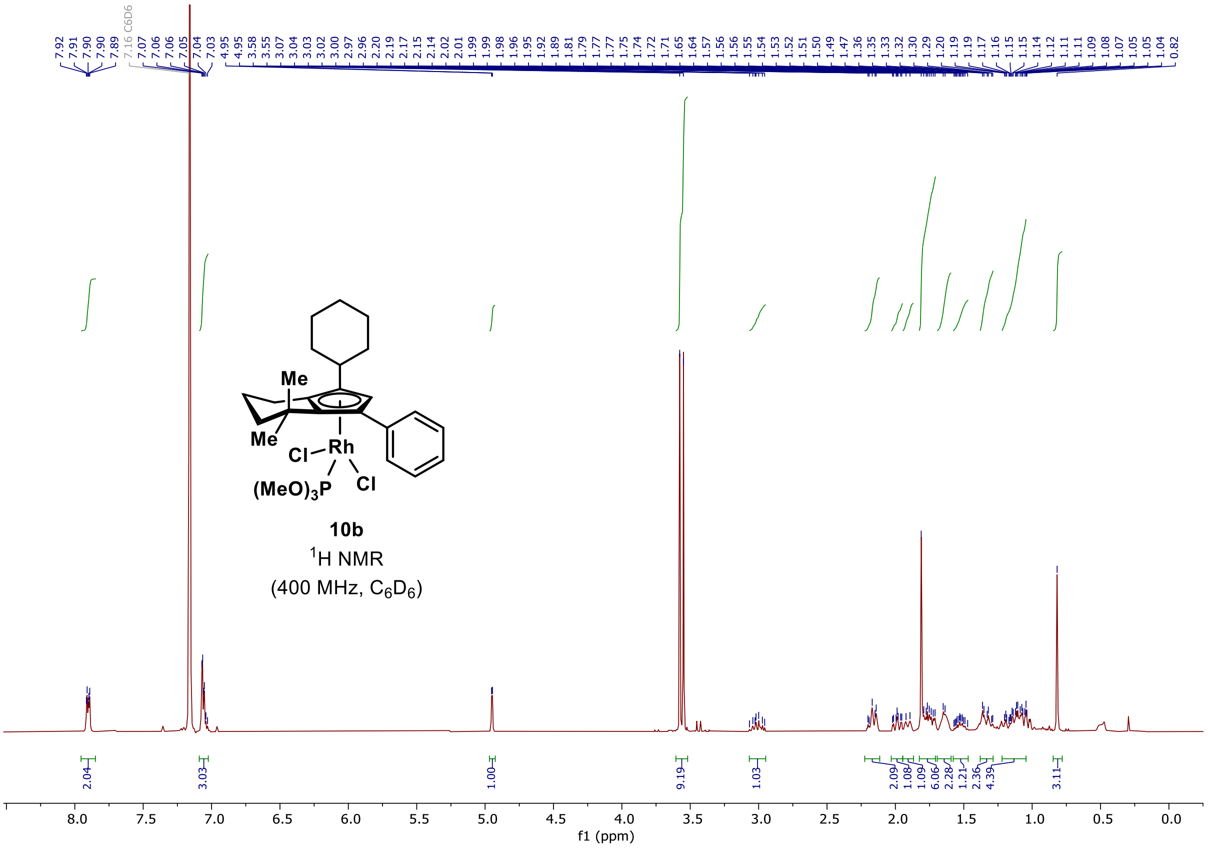
7.92
7.91
7.90
7.90
7.89
7.16 C6D6
7.07
7.06
7.06
7.05
7.04
7.03
4.95
4.95
3.58
3.55
3.07
3.04
3.03
3.02
3.00
2.97
2.96
2.20
2.19
2.17
2.15
2.14
2.02
2.01
1.99
1.99
1.98
1.96
1.95
1.92
1.89
1.81
1.79
1.77
1.77
1.75
1.74
1.72
1.71
1.65
1.64
1.57
1.56
1.56
1.55
1.54
1.53
1.52
1.51
1.50
1.49
1.47
1.36
1.35
1.33
1.32
1.30
1.29
1.20
1.19
1.19
1.17
1.16
1.15
1.15
1.14
1.12
1.11
1.11
1.09
1.08
1.07
1.05
1.05
1.04
0.82
Me
Me
Cl
Rh
(MeO)3P
Cl
10b
1H NMR
(400 MHz, C6D6)
2.04
3.03
1.00
9.19
1.03
2.09
1.08
1.09
6.06
2.28
1.21
2.36
4.39
3.11
8.0
7.5
7.0
6.5
6.0
5.5
5.0
4.5
4.0
3.5
3.0
2.5
2.0
1.5
1.0
0.5
0.0
f1 (ppm)

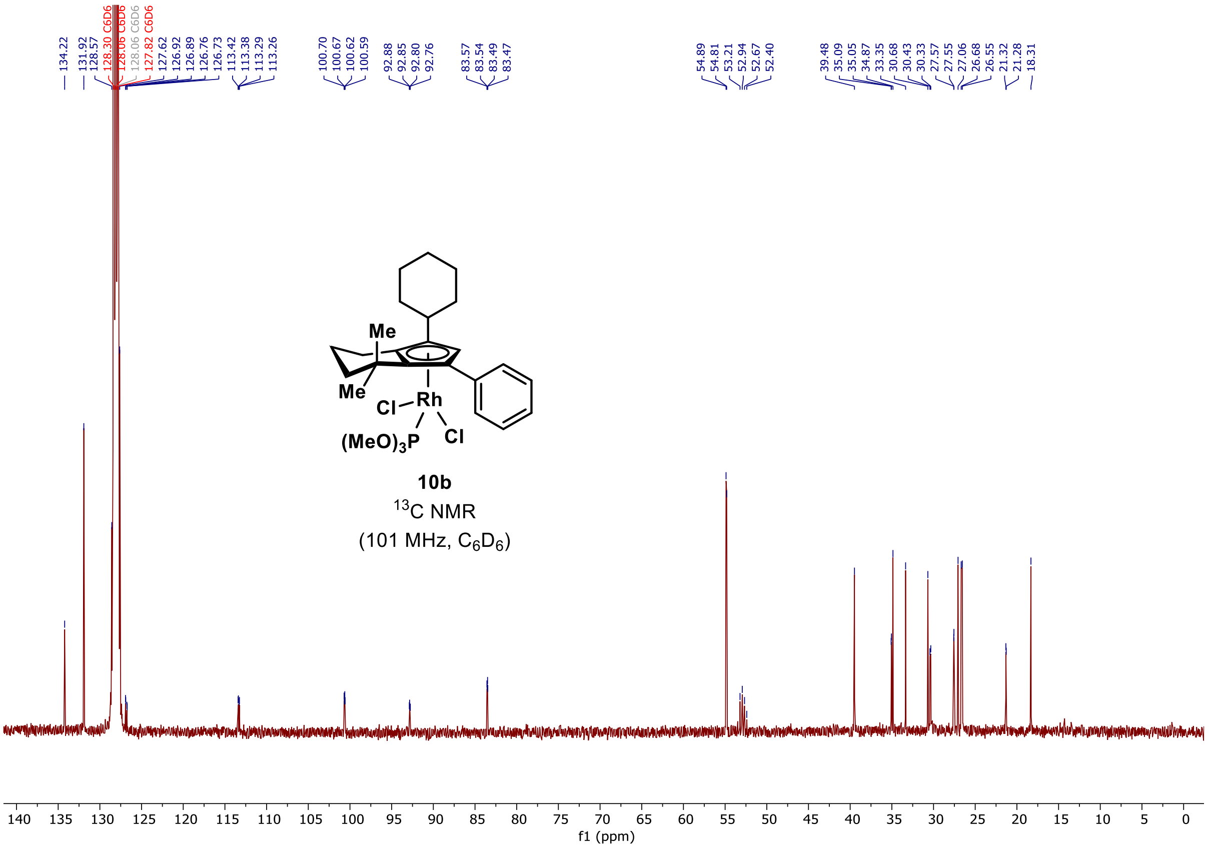
134.22
131.92
128.57
128.30 C6D6
128.06 C6D6
128.06 C6D6
127.82 C6D6
127.62
126.92
126.89
126.76
126.73
113.42
113.38
113.29
113.26
100.70
100.67
100.62
100.59
92.88
92.85
92.80
92.76
83.57
83.54
83.49
83.47
54.89
54.81
53.21
52.94
52.67
52.40
39.48
35.09
35.05
34.87
33.35
30.68
30.43
30.33
27.57
27.55
27.06
26.68
26.55
21.32
21.28
18.31
Me
Me
Cl
Rh
(MeO)3P
Cl
10b
13C NMR
(101 MHz, C6D6)
140
135
130
125
120
115
110
105
100
95
90
85
80
75
70
65
60
55
50
45
40
35
30
25
20
15
10
5
0
f1 (ppm)

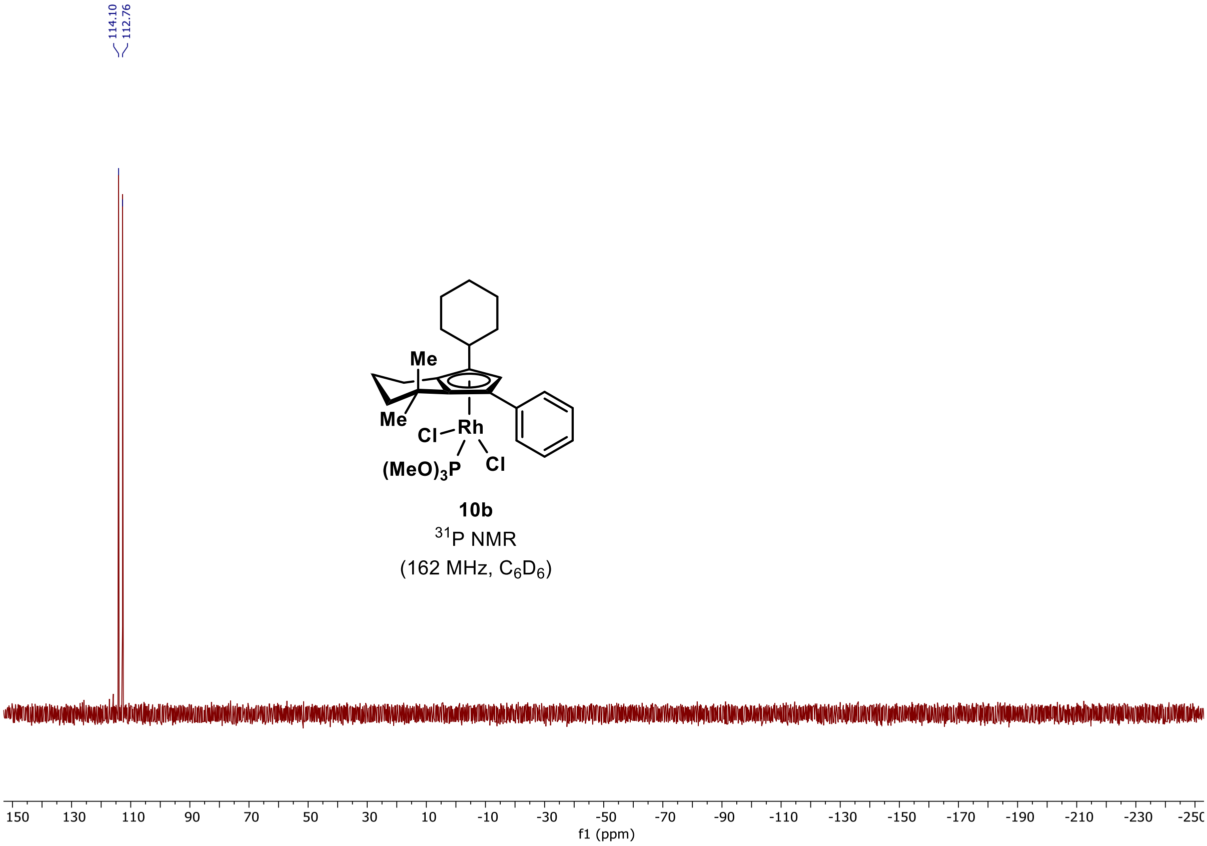
114.10
112.76
Me
Me
Cl
Rh
Cl
(MeO)3P
10b
31P NMR
(162 MHz, C6D6)
150
130
110
90
70
50
30
10
-10
-30
-50
-70
-90
-110
-130
-150
-170
-190
-210
-230
-250
f1 (ppm)

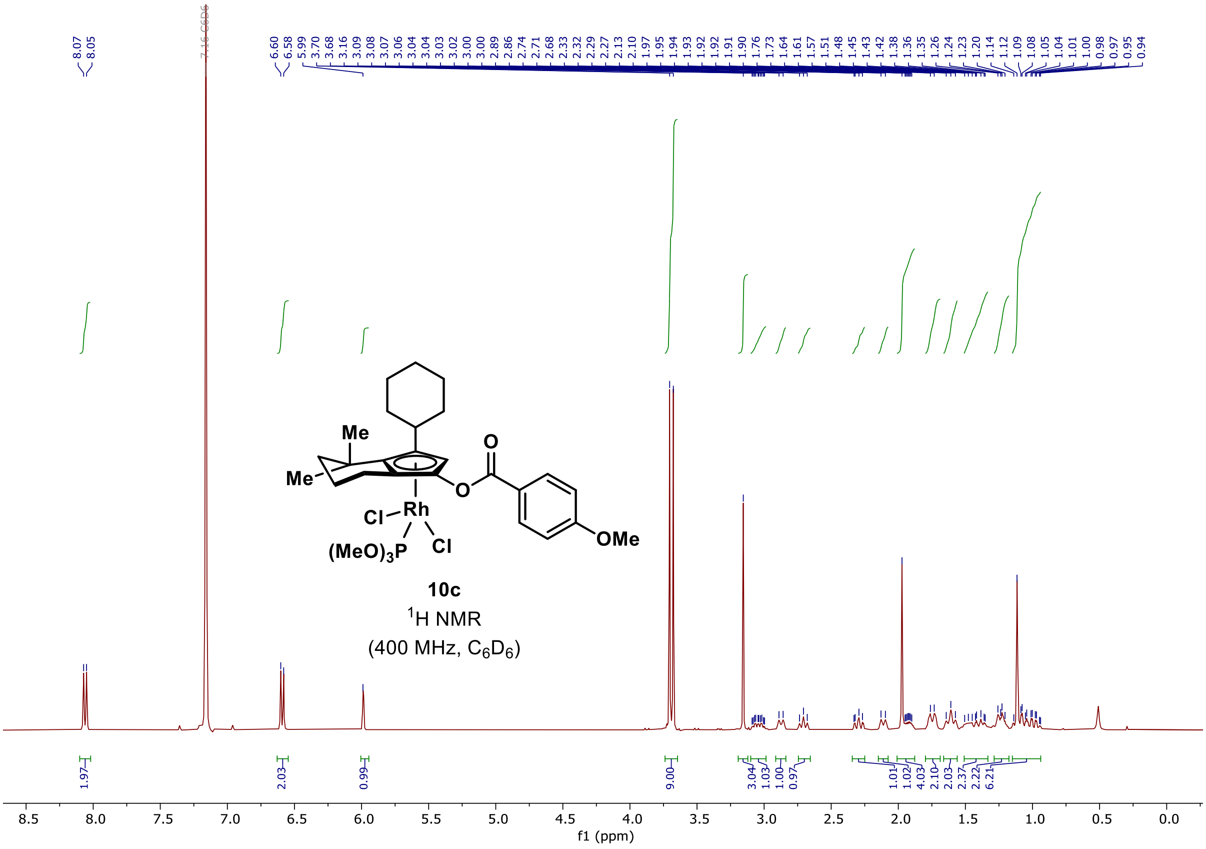
8.07
8.05
7.16 C6D6
6.60
6.58
5.99
3.70
3.68
3.16
3.09
3.08
3.07
3.06
3.04
3.04
3.03
3.02
3.00
3.00
2.89
2.86
2.74
2.71
2.68
2.33
2.32
2.29
2.27
2.13
2.10
1.97
1.95
1.94
1.93
1.93
1.92
1.92
1.91
1.90
1.76
1.73
1.64
1.61
1.57
1.51
1.48
1.45
1.43
1.42
1.38
1.36
1.35
1.26
1.24
1.23
1.20
1.14
1.12
1.09
1.08
1.05
1.04
1.01
1.00
0.98
0.97
0.95
0.94
Me
Me
O
O
Cl
Rh
Cl
(MeO)3P
OMe
10c
1H NMR
(400 MHz, C6D6)
1.97
2.03
0.99
9.00
3.04
1.03
1.00
0.97
1.01
1.02
4.03
2.10
2.03
2.37
2.22
6.21
8.5
8.0
7.5
7.0
6.5
6.0
5.5
5.0
4.5
4.0
3.5
3.0
2.5
2.0
1.5
1.0
0.5
0.0
f1 (ppm)

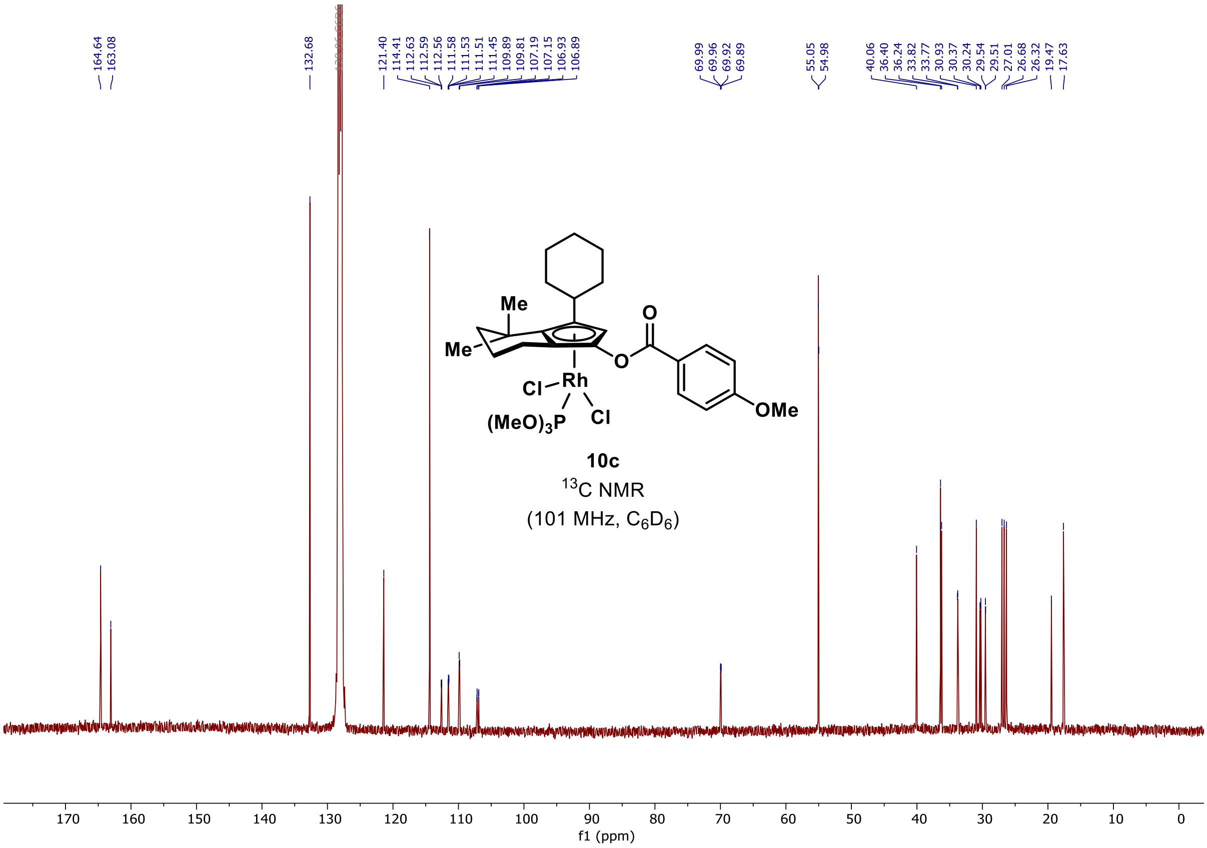
164.64
163.08
132.68
121.40
114.41
112.63
112.59
112.56
111.58
111.53
111.51
111.45
109.89
109.81
107.19
107.15
106.93
106.89
69.99
69.96
69.92
69.89
55.05
54.98
40.06
36.40
36.24
33.82
33.77
30.93
30.37
30.24
29.54
29.51
27.01
26.68
26.32
19.47
17.63
Me
Me
O
O
Cl
Rh
Cl
(MeO)3P
OMe
10c
13C NMR
(101 MHz, C6D6)
170
160
150
140
130
120
110
100
90
80
70
60
50
40
30
20
10
0
f1 (ppm)

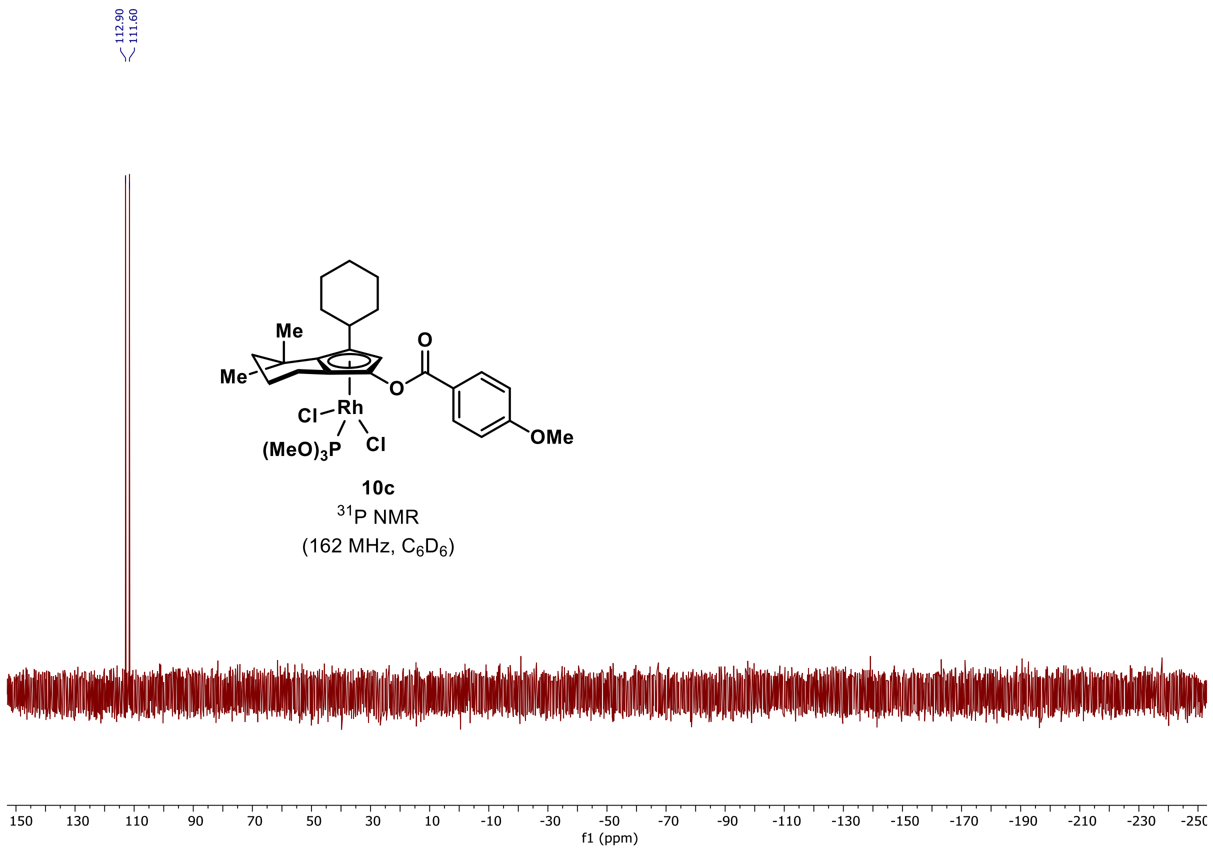
112.90
111.60
Me
Me
O
O
Cl
Rh
Cl
(MeO)3P
OMe
10c
31P NMR
(162 MHz, C6D6)
150
130
110
90
70
50
30
10
-10
-30
-50
-70
-90
-110
-130
-150
-170
-190
-210
-230
-250
f1 (ppm)

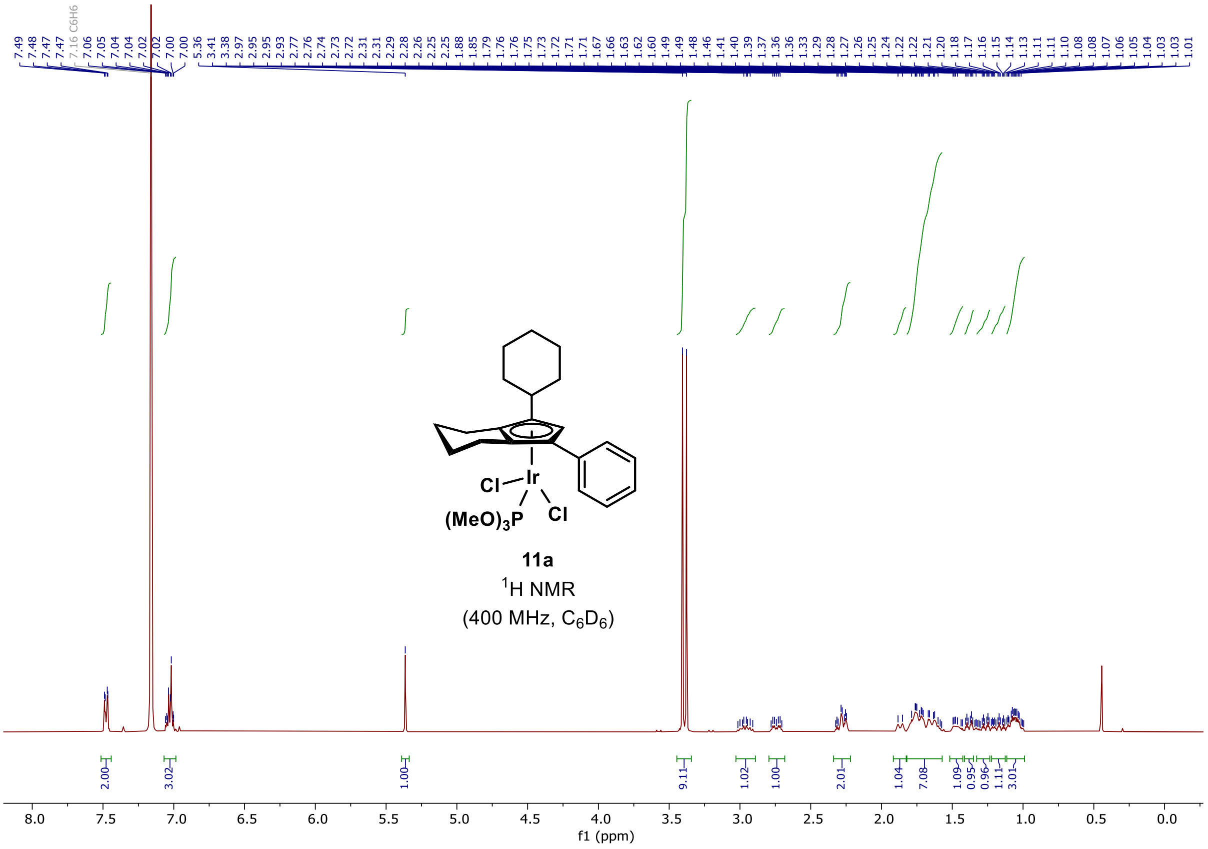
Cl
Ir
(MeO)3P
Cl
11a
1H NMR
(400 MHz, C6D6)
f1 (ppm)

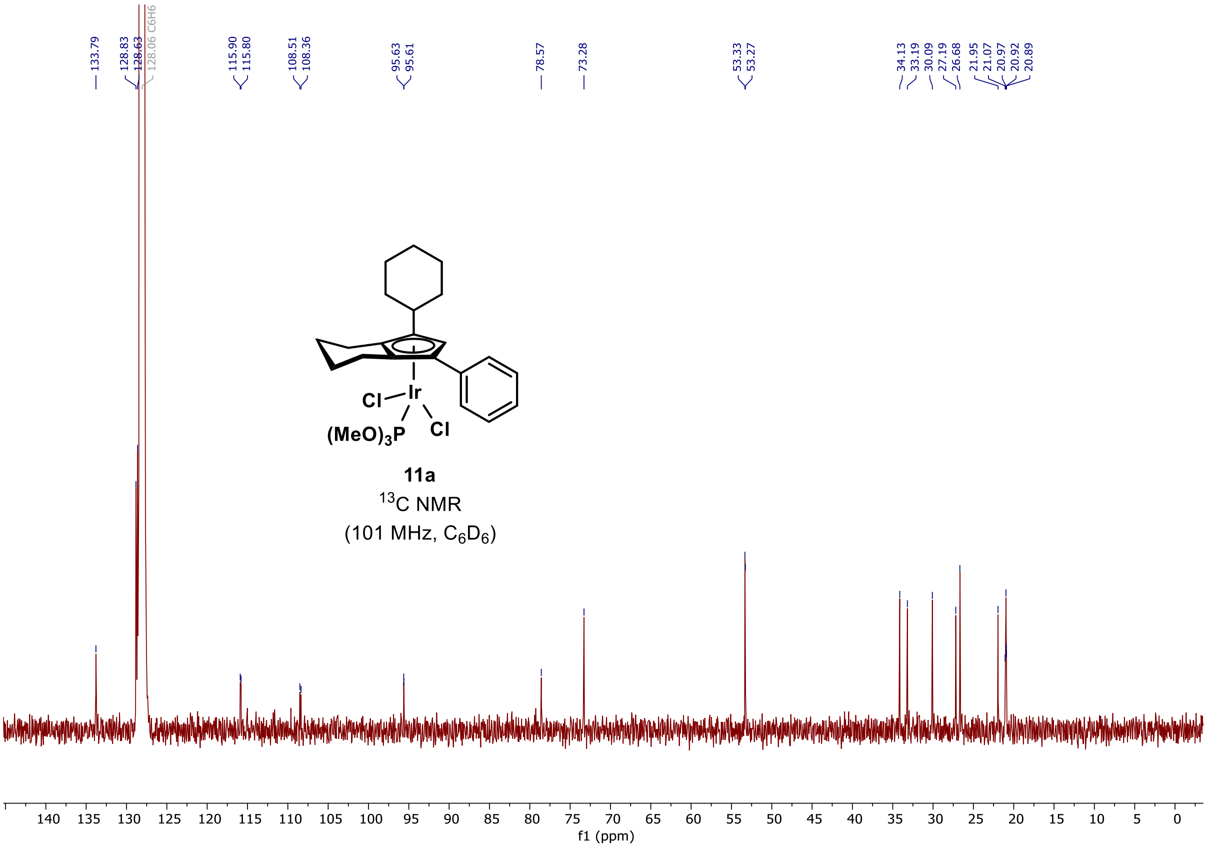
Cl
Ir
(MeO)3P
Cl
11a
13C NMR
(101 MHz, C6D6)
f1 (ppm)

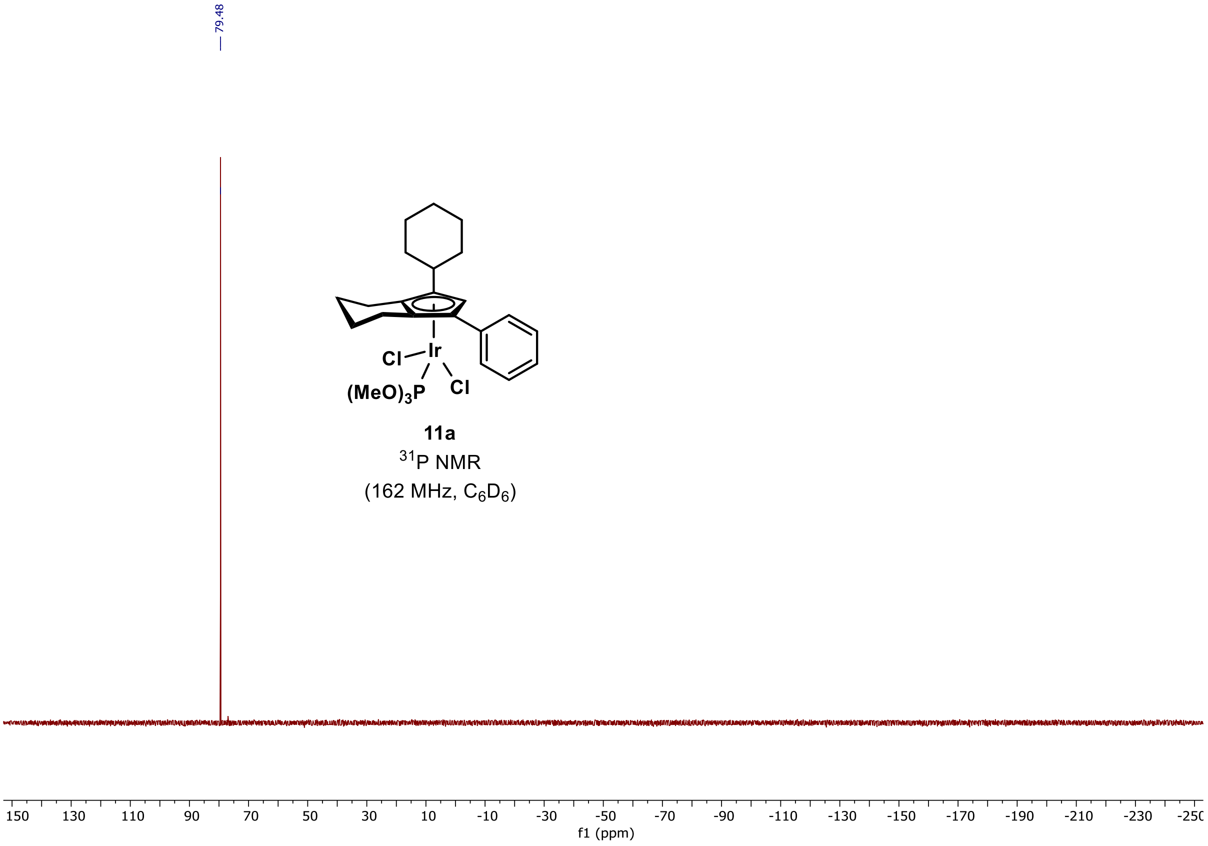
79.48
Cl
Ir
(MeO)3P
Cl
11a
31P NMR
(162 MHz, C6D6)
150 130 110 90 70 50 30 10 -10 -30 -50 -70 -90 -110 -130 -150 -170 -190 -210 -230 -250
f1 (ppm)

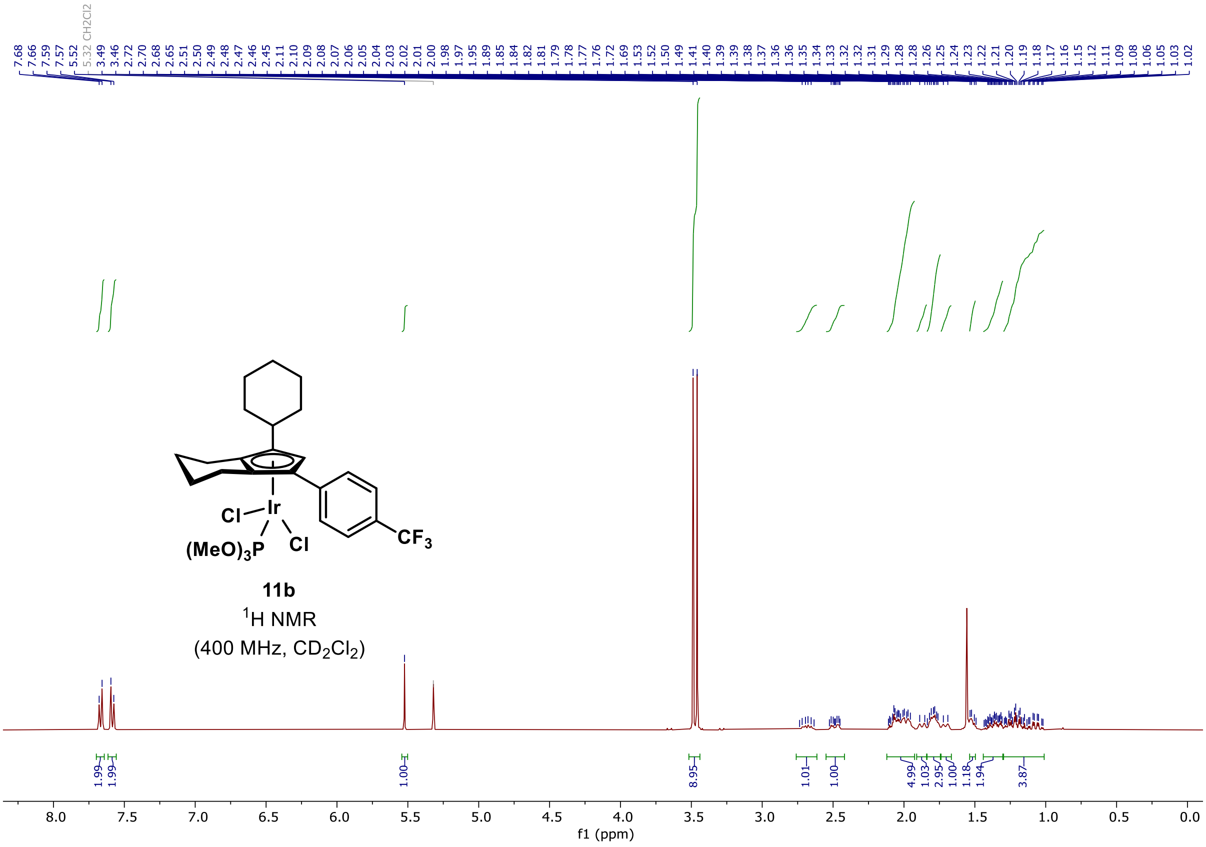

11b

^{1}H NMR

(400 MHz, CD_2Cl_2)

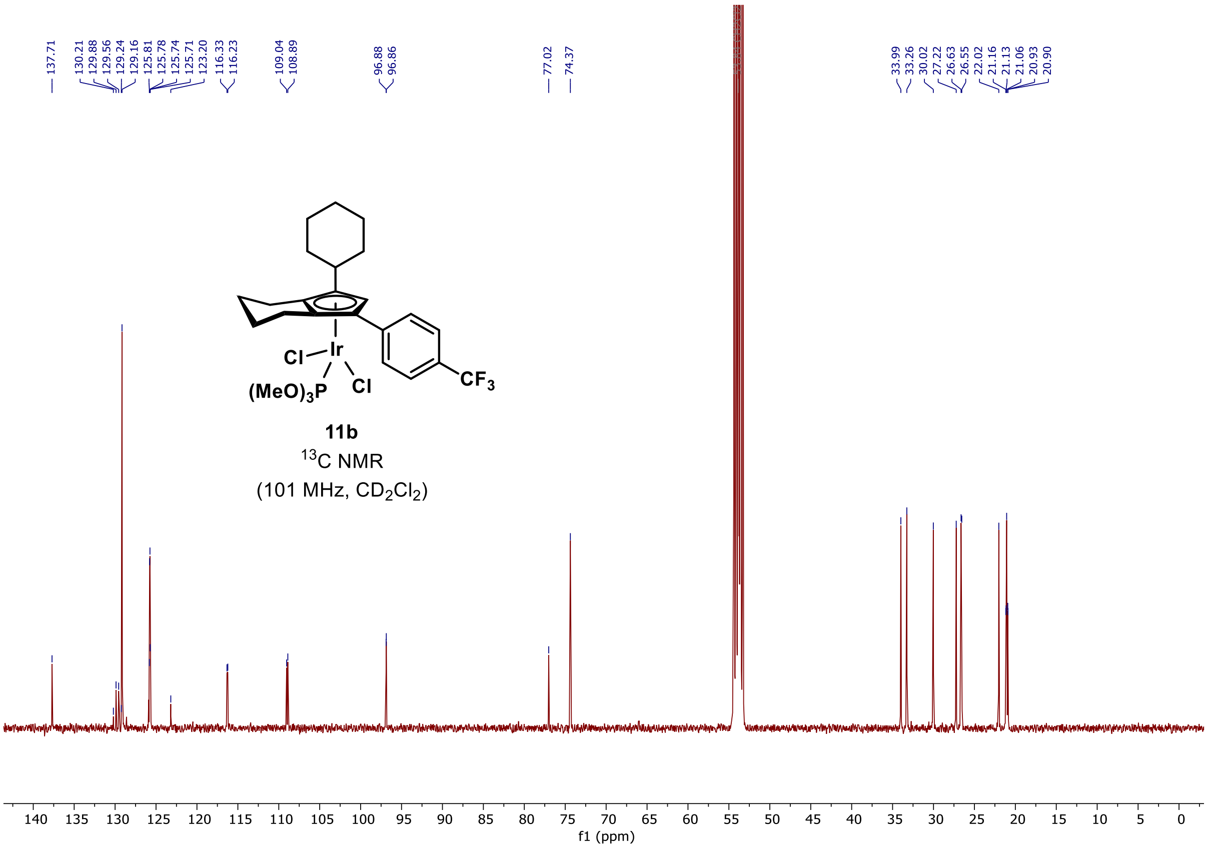

11b

^{13}C NMR

(101 MHz, CD_2Cl_2)

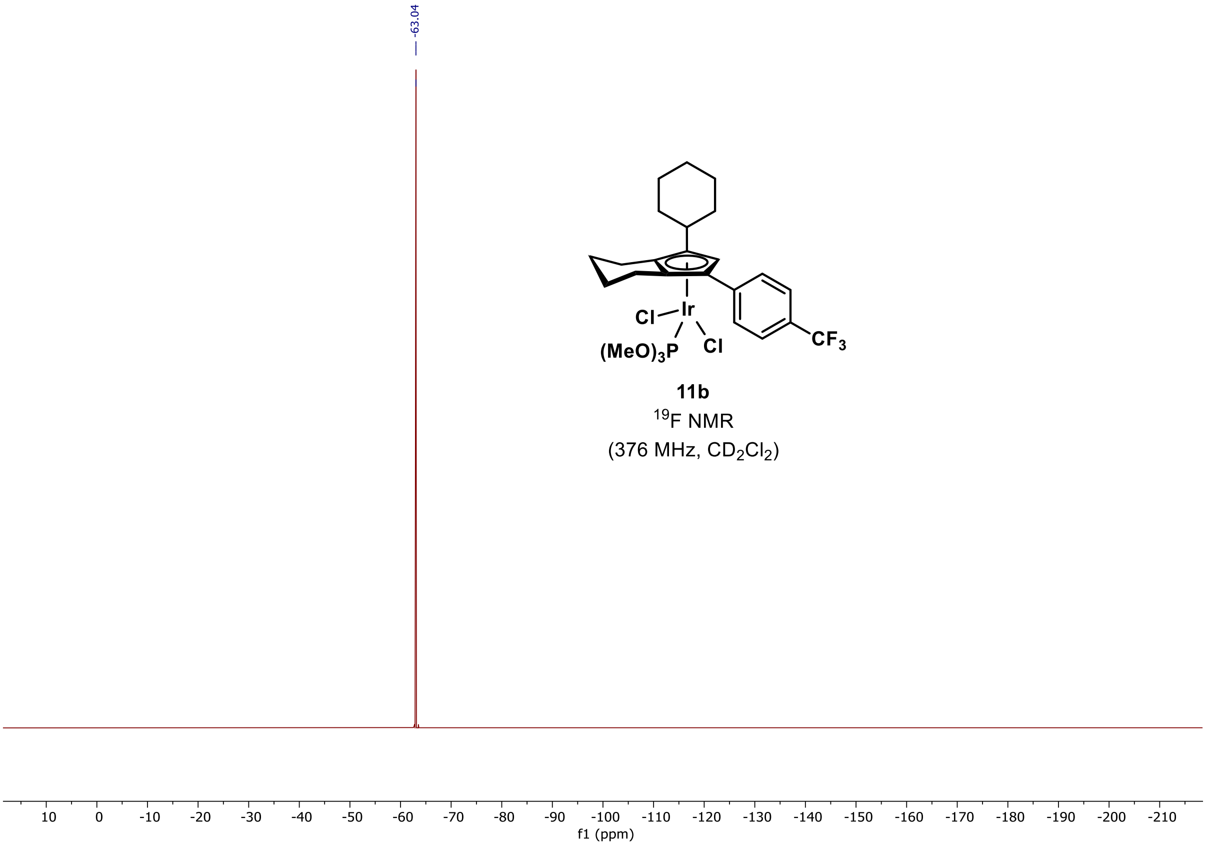
-63.04
Cl
Ir
(MeO)3P
Cl
CF3
11b
19F NMR
(376 MHz, CD2Cl2)
10
0
-10
-20
-30
-40
-50
-60
-70
-80
-90
-100
-110
-120
-130
-140
-150
-160
-170
-180
-190
-200
-210
f1 (ppm)

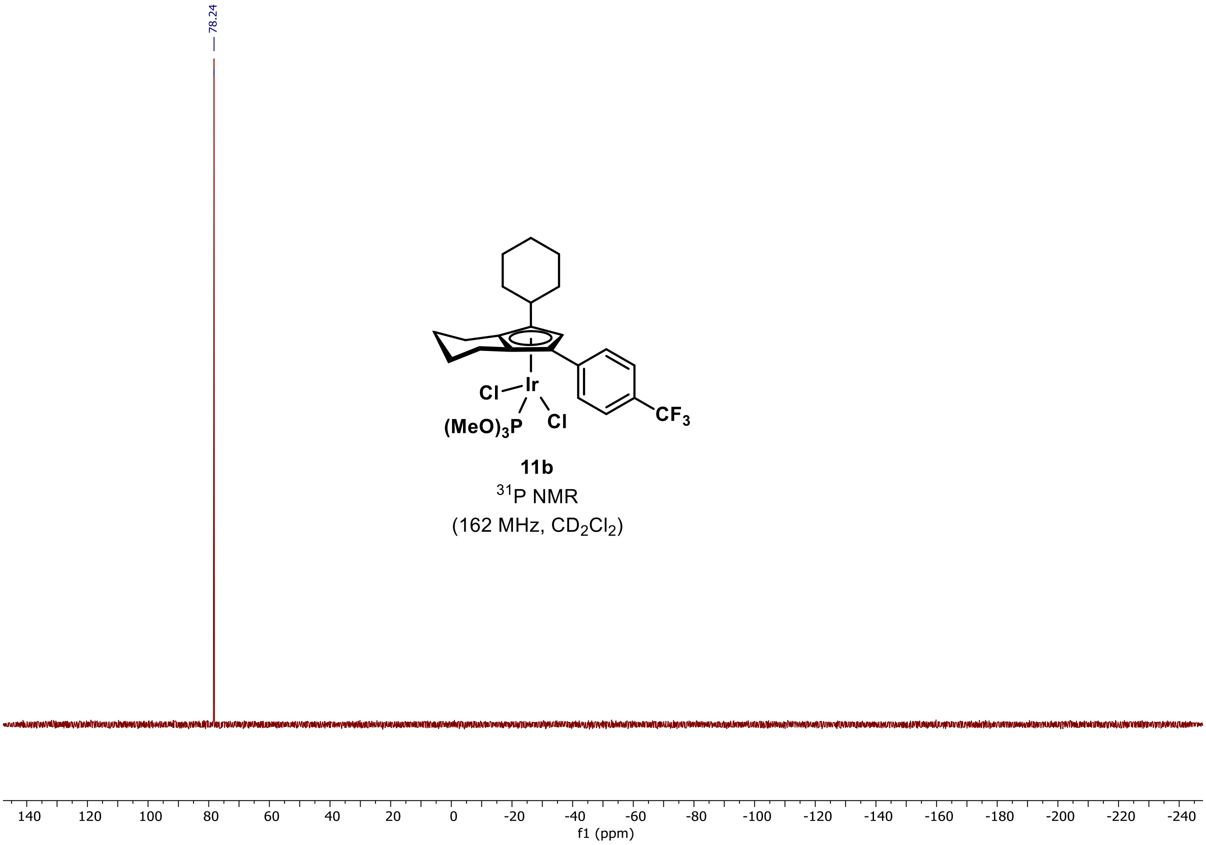
78.24
Cl
Ir
(MeO)3P
Cl
CF3
11b
31P NMR
(162 MHz, CD2Cl2)
140
120
100
80
60
40
20
0
-20
-40
-60
-80
-100
-120
-140
-160
-180
-200
-220
-240
f1 (ppm)

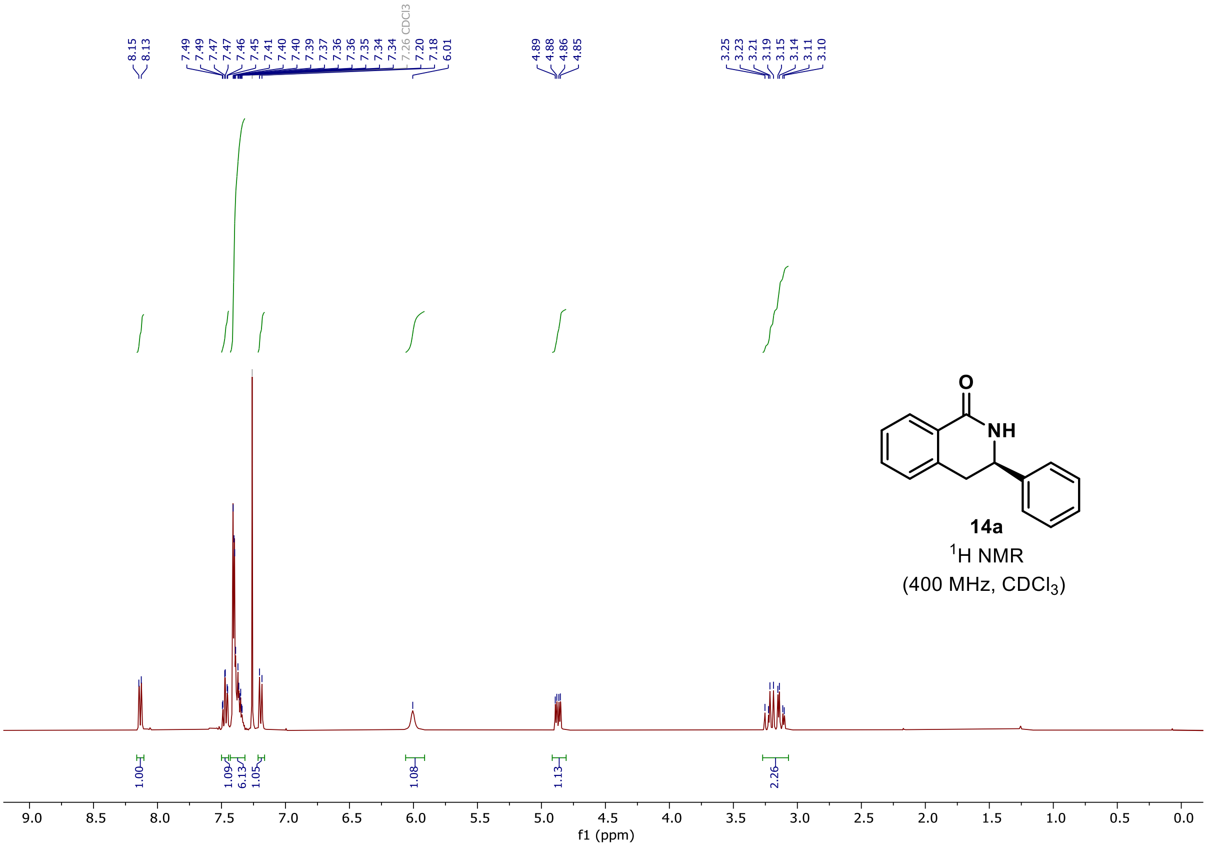
14a
1H NMR
(400 MHz, CDCl3)
f1 (ppm)

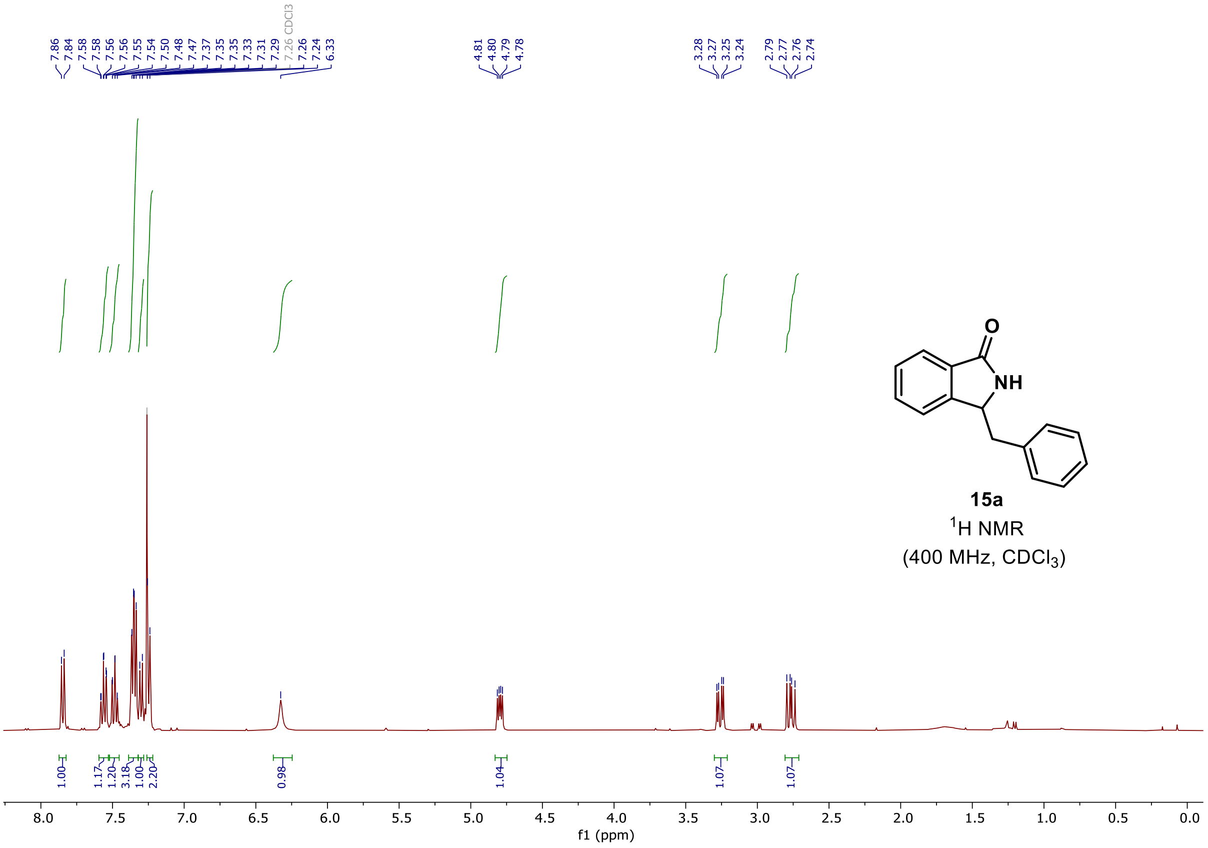

15a

^{1}H NMR

(400 MHz, $CDCl_3$)

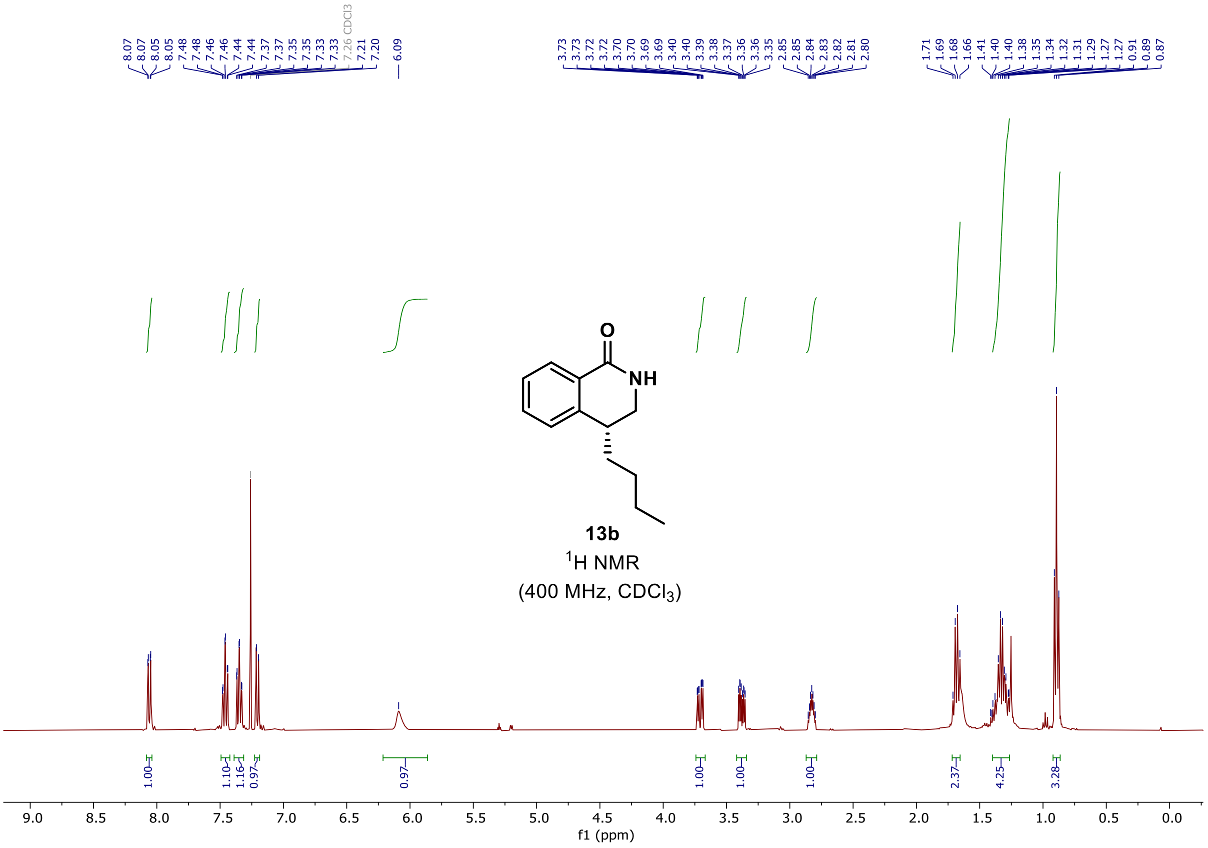
O
NH
13b
¹H NMR
(400 MHz, CDCl3)
f1 (ppm)

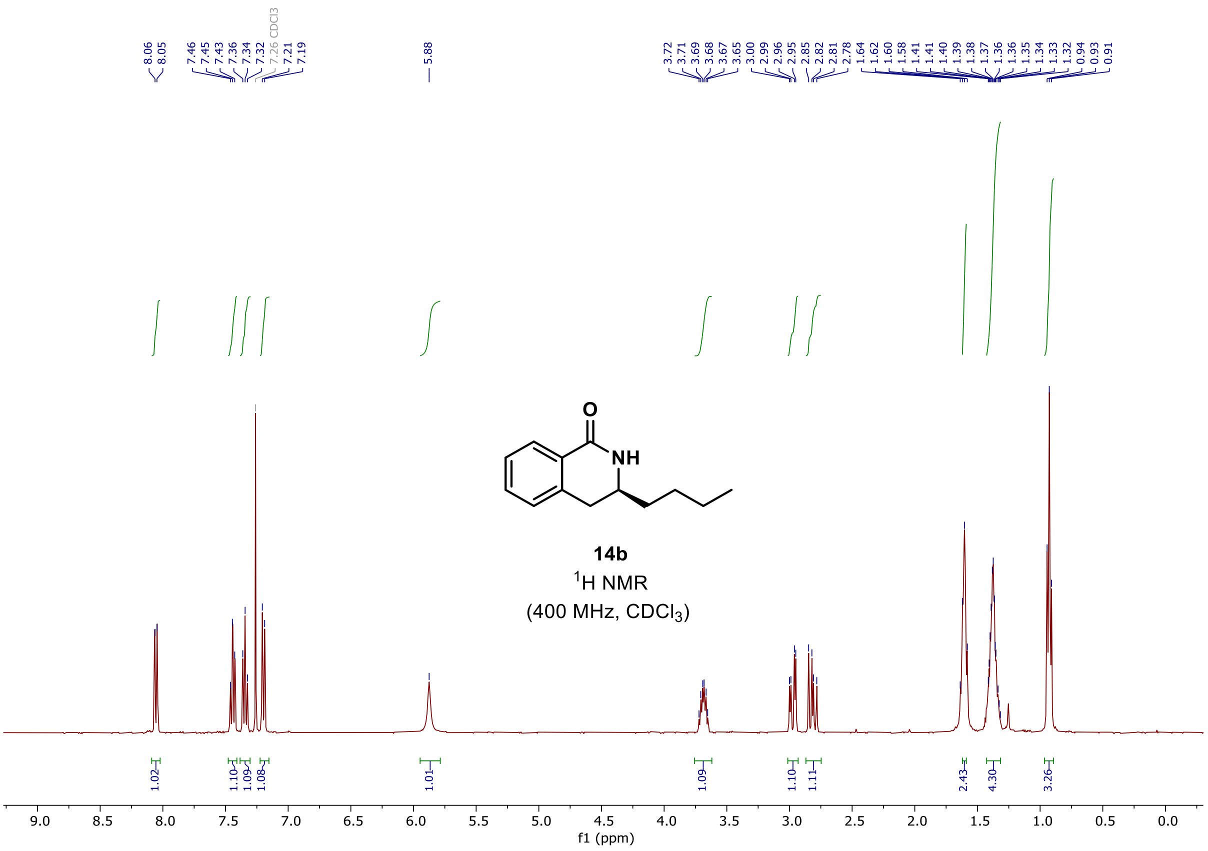

14b
^{1}H NMR
(400 MHz, $CDCl_3$)
f1 (ppm)

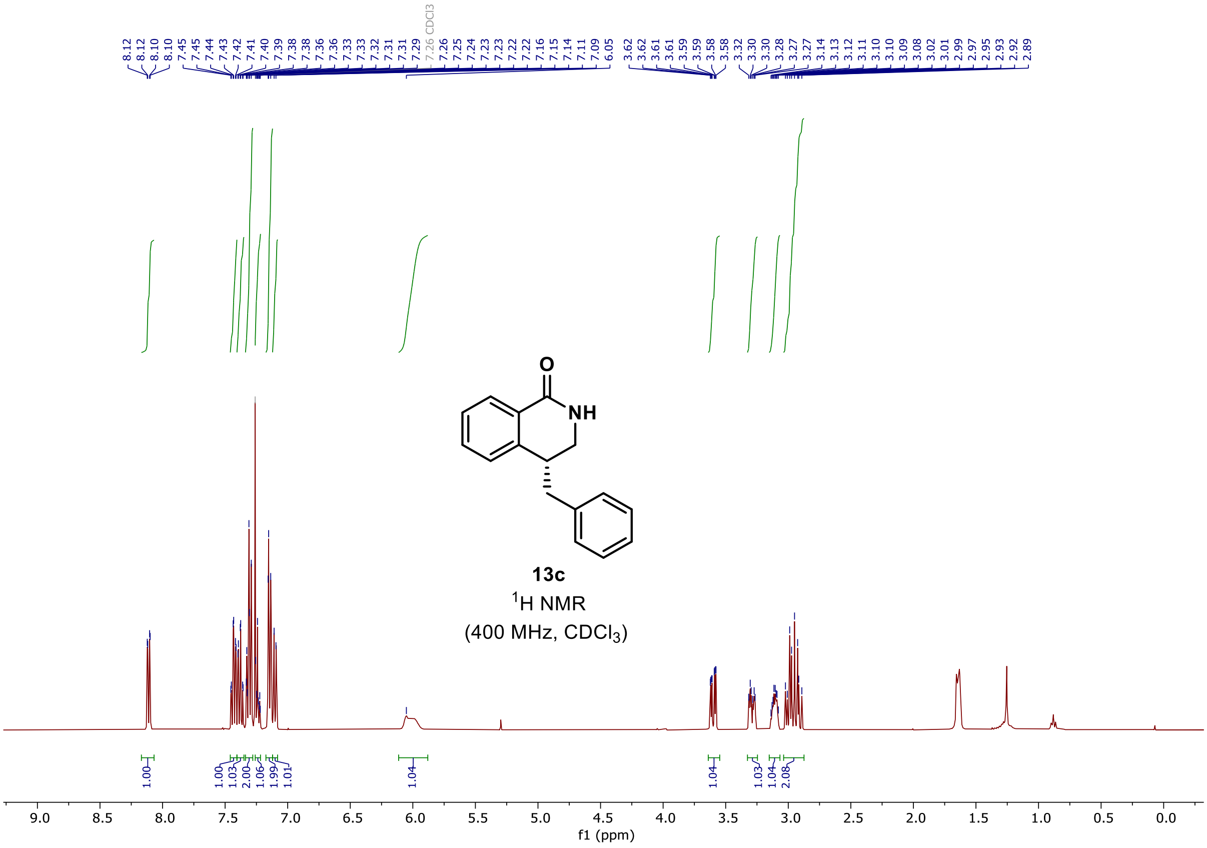

13c
^{1}H NMR
(400 MHz, CDCl$_3$)
f1 (ppm)

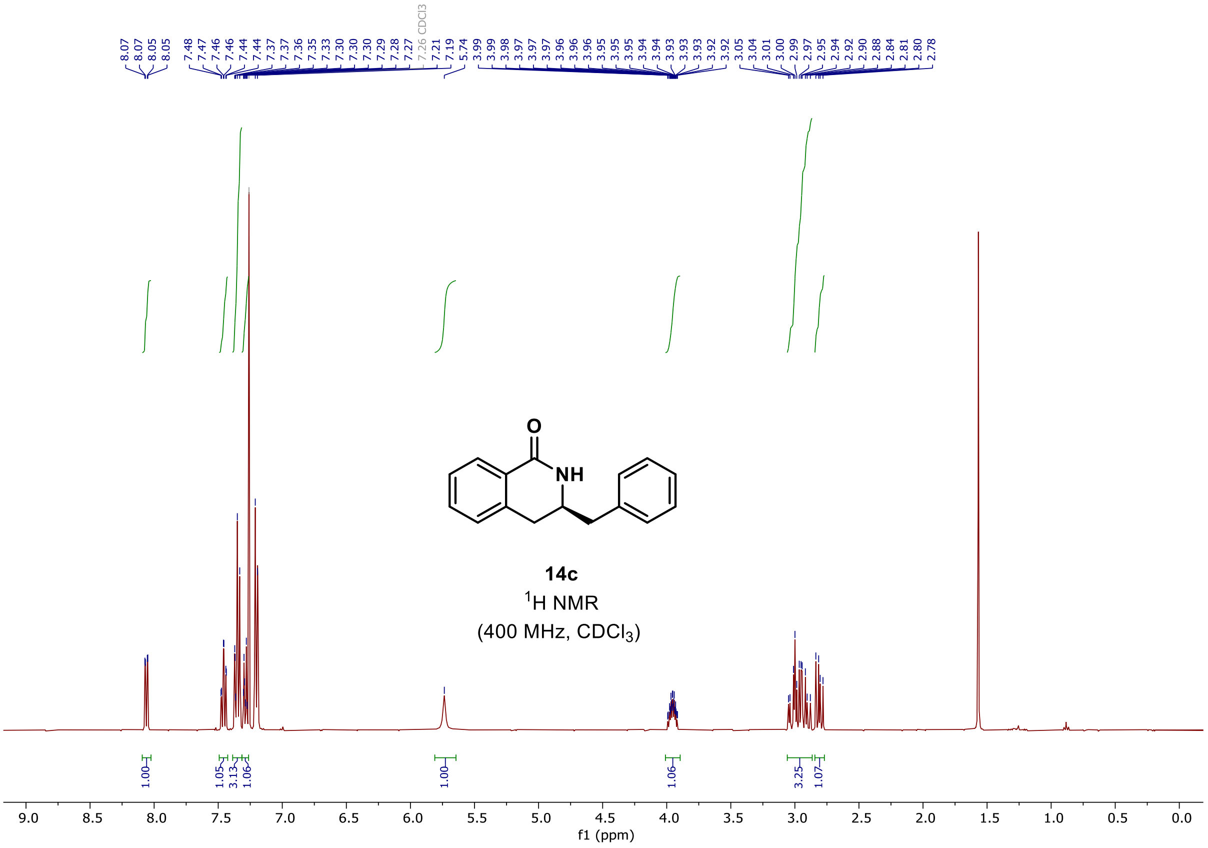
14c
1H NMR
(400 MHz, CDCl3)
f1 (ppm)

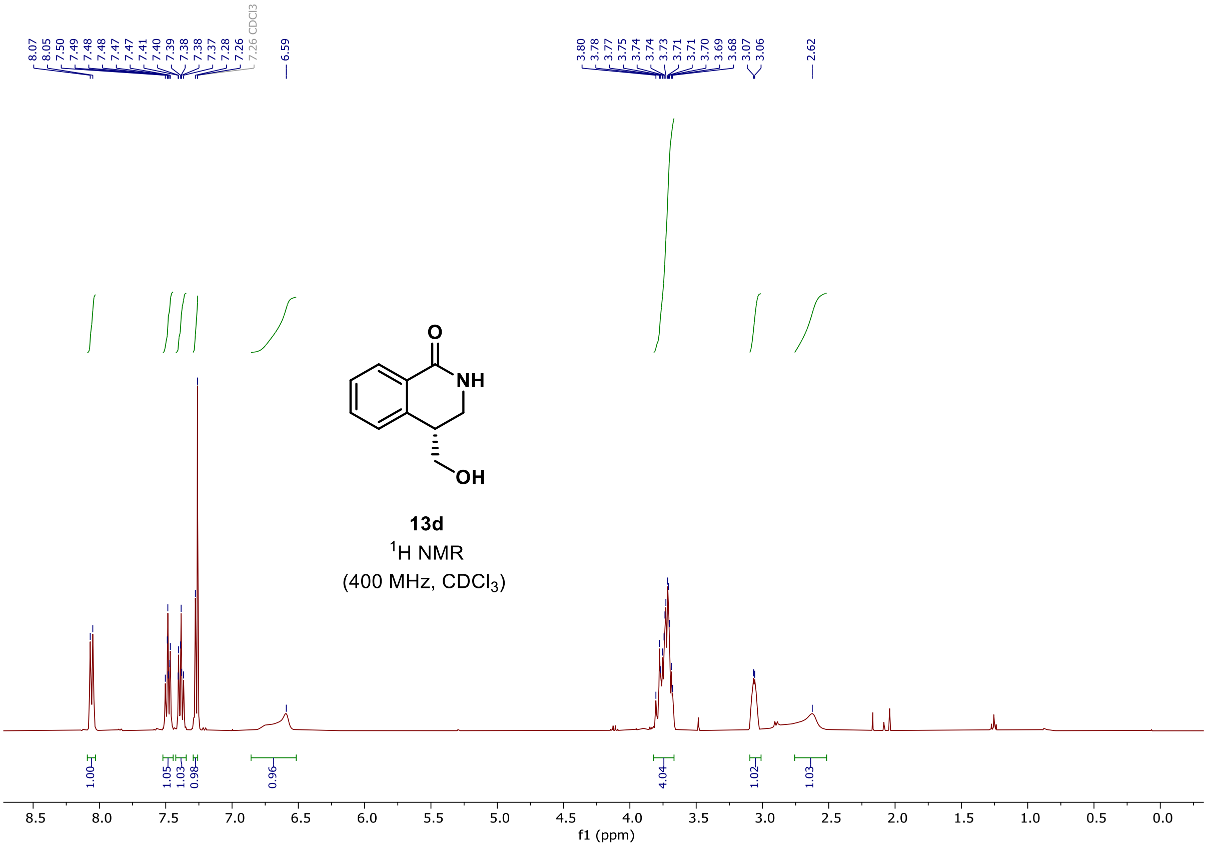

13d
^{1}H NMR
(400 MHz, $CDCl_3$)

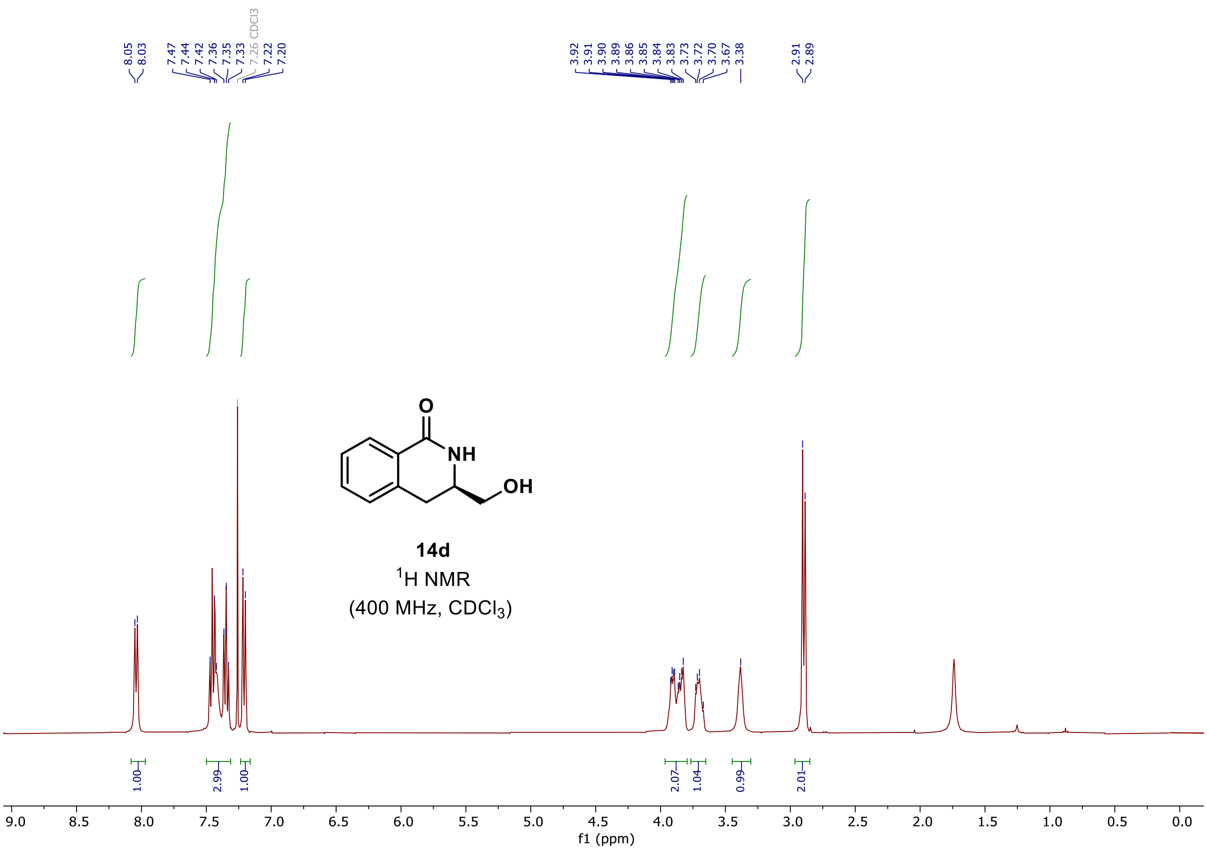

14d
^{1}H NMR
(400 MHz, $CDCl_3$)

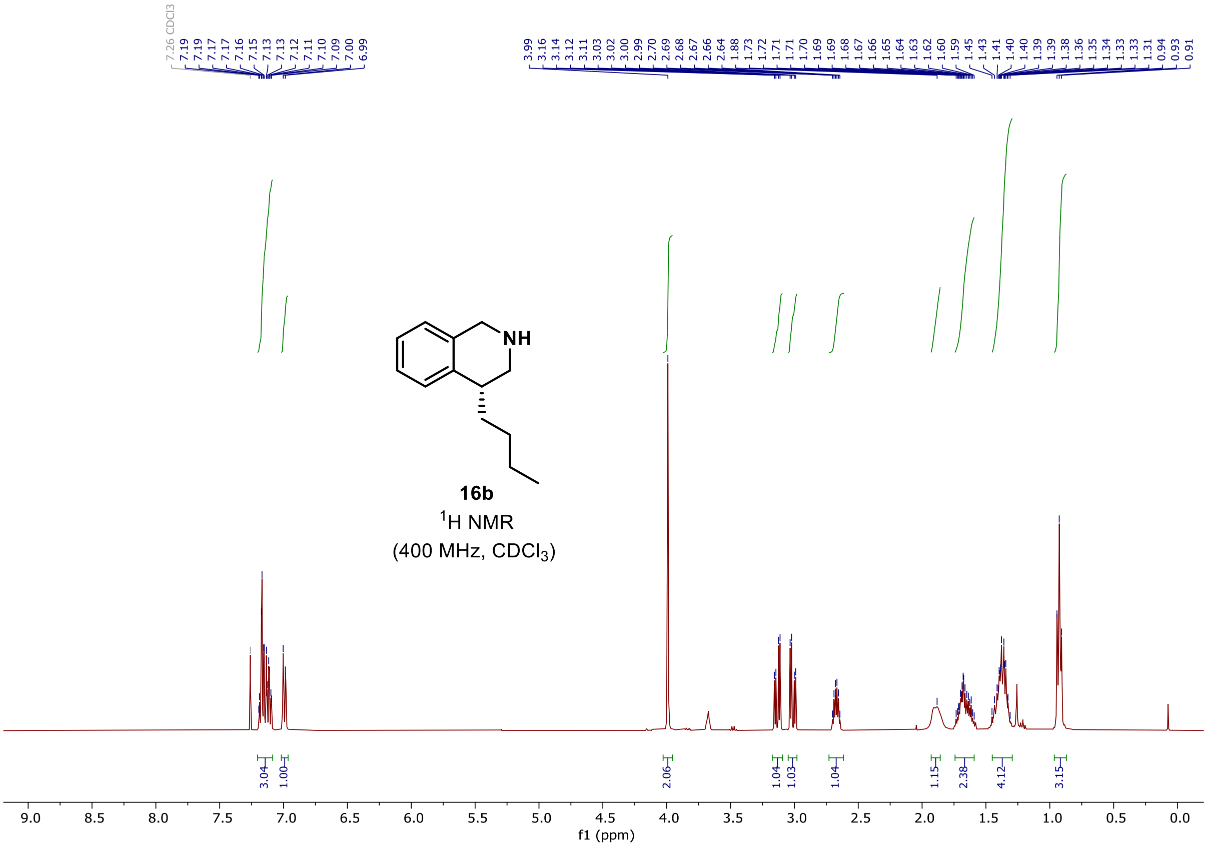
16b
^{1}H NMR
(400 MHz, $CDCl_3$)
f1 (ppm)

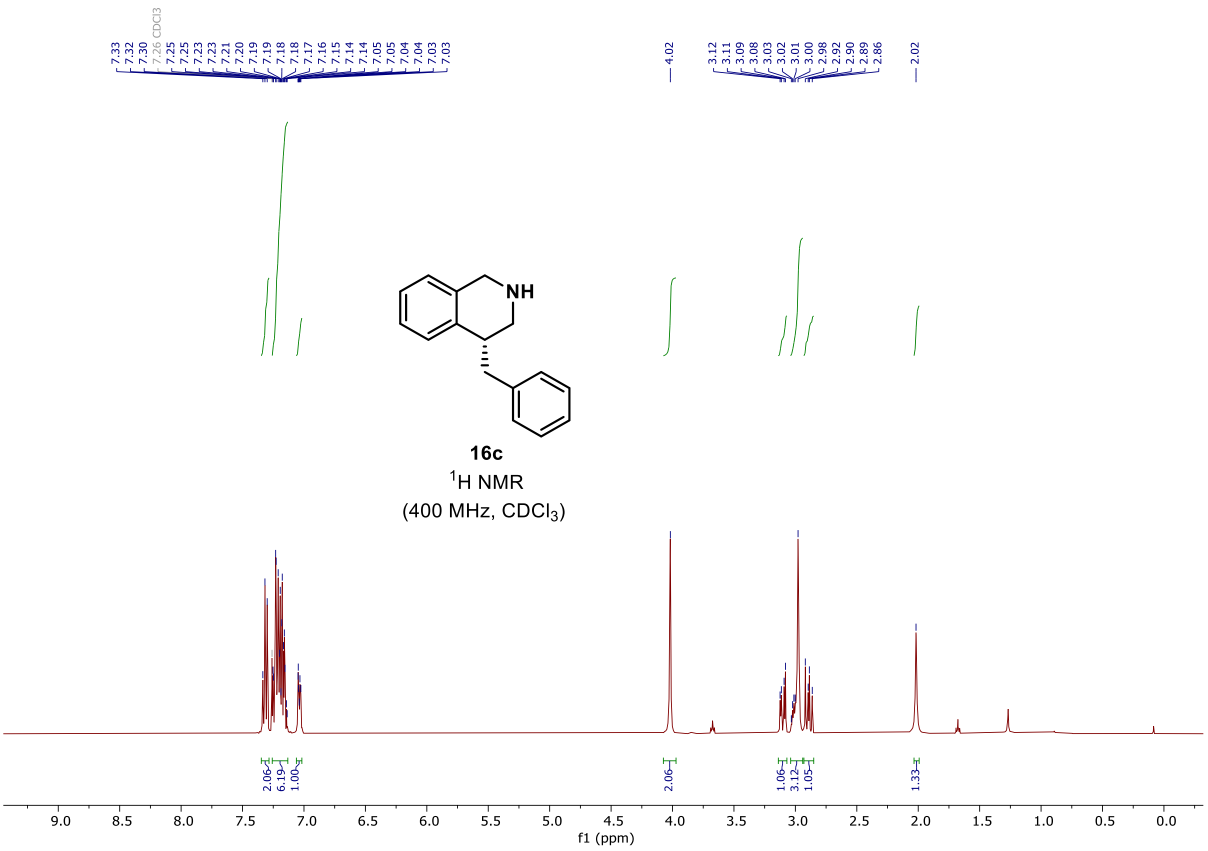
16c
^{1}H NMR
(400 MHz, CDCl$_3$)
f1 (ppm)

8. X-ray Crystallography Data

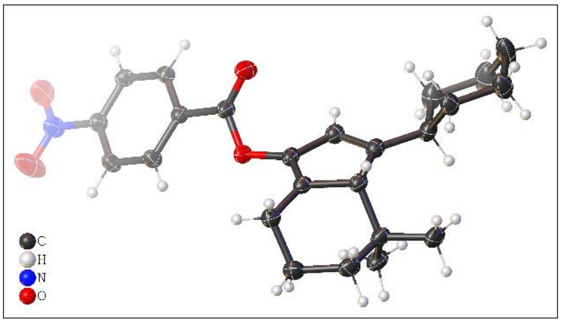

Experimental. Single clear intense yellow prism-shaped crystals of **8h** were used as supplied. A suitable crystal with dimensions 0.87 × 0.55 × 0.32 mm^3 was selected and mounted on a XtaLAB Synergy R, DW system, HyPix-Arc 150 diffractometer. The crystal was kept at a steady T = 140.00(10) K during data collection. The structure was solved with the ShelXT 2018/2 (Sheldrick, 2015) solution program using dual methods and by using Olex2 1.5 (Dolomanov et al., 2009) as the graphical interface. The model was refined with ShelXL 2019/3 (Sheldrick, 2015) using full matrix least squares minimisation on $\boldsymbol{F}^2$.

Crystal Data. $C_{24}H_{29}NO_4$, M_r = 395.48, monoclinic, $P2_1$ (No. 4), a = 9.67976(16) Å, b = 11.92698(13) Å, c = 10.11016(16) Å, β = 114.4033(19)°, $\alpha = \gamma$ = 90°, V = 1062.94(3) Å^3, T = 140.00(10) K, Z = 2, Z' = 1, μ(Cu K$_\alpha$) = 0.671, 20487 reflections measured, 4282 unique (R_{int} = 0.0153) which were used in all calculations. The final wR_2 was 0.0773 (all data) and R_1 was 0.0291 (I≥2 σ(I)).

**Compound**	**8h**
Formula	$C_{24}H_{29}NO_4$
$D_{calc.}$/ g cm^{-3}	1.236
μ/mm^{-1}	0.671
Formula Weight	395.48
Colour	clear intense yellow
Shape	prism-shaped
Size/mm^3	0.87×0.55×0.32
T/K	140.00(10)
Crystal System	monoclinic
Flack Parameter	-0.01(4)
Space Group	$P2_1$
a/Å	9.67976(16)
b/Å	11.92698(13)
c/Å	10.11016(16)
α/°	90
β/°	114.4033(19)
γ/°	90
V/Å^3	1062.94(3)
Z	2
Z'	1
Wavelength/Å	1.54184
Radiation type	Cu K$_\alpha$
Θ_{min}/°	4.803
Θ_{max}/°	75.146
Measured Refl's.	20487
Indep't Refl's	4282
Refl's I≥2 σ(I)	4261
R_{int}	0.0153
Parameters	265
Restraints	1
Largest Peak	0.301
Deepest Hole	-0.228
GooF	1.037
wR_2 (all data)	0.0773
wR_2	0.0772
R_1 (all data)	0.0292
R_1	0.0291
CCDC number	2359464

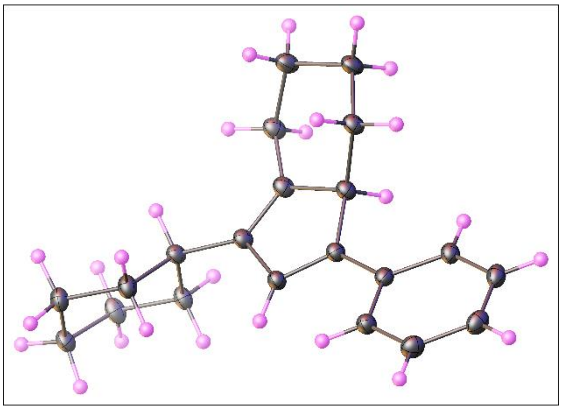

Experimental. Single colourless plate crystals of *rac*-**1a** were used as supplied. A suitable crystal with dimensions 0.14 × 0.06 × 0.02 mm^3 was selected and mounted on a XtaLAB Synergy R, DW system, HyPix-Arc 150 diffractometer. The crystal was kept at a steady T = 139.99(10) K during data collection. The structure was solved with the ShelXT 2018/2 (Sheldrick, 2015) solution program using dual methods and by using Olex2 (Dolomanov et al., 2009) as the graphical interface. The model was refined with ShelXL 2018/3 (Sheldrick, 2015) using full matrix least squares minimisation on $\boldsymbol{F}^2$.

Crystal Data. $C_{21}H_{26}$, M_r = 278.42, monoclinic, $P2_1/c$ (No. 14), a = 12.8949(3) Å, b = 11.2985(2) Å, c = 10.99813(19) Å, β = 96.4097(19)°, $\alpha = \gamma$ = 90°, V = 1592.33(6) Å^3, T = 139.99(10) K, Z = 4, Z' = 1, μ(Cu K_α) = 0.479, 28052 reflections measured, 3267 unique (R_{int} = 0.0268) which were used in all calculations. The final wR_2 was 0.1881 (all data) and R_1 was 0.0655 (I≥2 σ(I)).

**Compound**	*rac*-**1a**
Formula	$C_{21}H_{26}$
$D_{calc.}$/ g cm^{-3}	1.161
μ/mm^{-1}	0.479
Formula Weight	278.42
Colour	colourless
Shape	plate
Size/mm^3	0.14×0.06×0.02
T/K	139.99(10)
Crystal System	monoclinic
Space Group	$P2_1/c$
a/Å	12.8949(3)
b/Å	11.2985(2)
c/Å	10.99813(19)
α/°	90
β/°	96.4097(19)
γ/°	90
V/Å^3	1592.33(6)
Z	4
Z'	1
Wavelength/Å	1.54184
Radiation type	Cu$K\alpha$
Θ_{min}/°	3.449
Θ_{max}/°	75.817
Measured Refl's.	28052
Indep't Refl's	3267
Refl's I≥2σ(I)	2872
R_{int}	0.0268
Parameters	190
Restraints	0
Largest Peak/e Å^{-3}	0.387
Deepest Hole/e Å^{-3}	-0.238
GooF	1.142
wR_2 (all data)	0.1881
wR_2	0.1839
R_1 (all data)	0.0720
R_1	0.0655
CCDC number	2359458

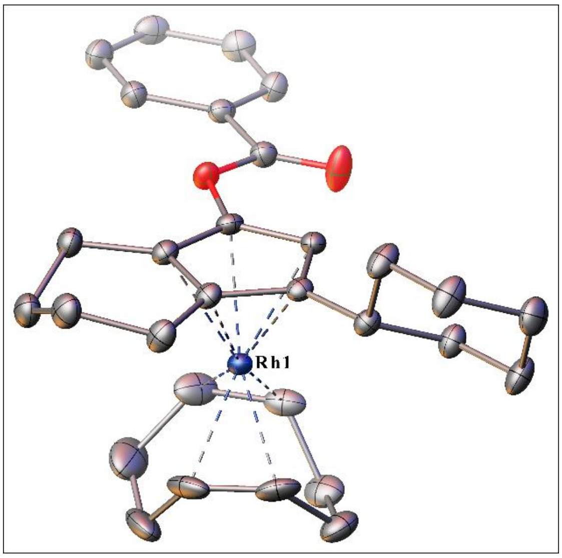

Experimental. Single clear intense yellow prism crystals of *rac*-**2d** were used as supplied. A suitable crystal with dimensions 0.14 × 0.10 × 0.07 mm³ was selected and mounted on a SuperNova, Dual, Cu at home/near, AtlasS2 diffractometer. The crystal was kept at a steady T = 140.01(10) K during data collection. The structure was solved with the ShelXT 2018/2 (Sheldrick, 2015) solution program using dual methods and by using Olex2 (Dolomanov et al., 2009) as the graphical interface. The model was refined with ShelXL 2018/3 (Sheldrick, 2015) using full matrix least squares minimisation on F^2.

Crystal Data. $C_{30}H_{37}O_2Rh$, M_r = 532.50, monoclinic, $C2/c$ (No. 15), a = 33.0434(3) Å, b = 13.06155(11) Å, c = 11.56396(11) Å, β = 99.3953(9)°, $\alpha = \gamma$ = 90°, V = 4924.04(8) Å³, T = 140.01(10) K, Z = 8, Z' = 1, μ(Cu K$_\alpha$) = 5.789, 20471 reflections measured, 4843 unique (R_{int} = 0.0258) which were used in all calculations. The final wR_2 was 0.0484 (all data) and R_1 was 0.0196 (I≥2 σ(I)).

**Compound**	*rac*-**2d**
Formula	$C_{30}H_{37}O_2Rh$
$D_{calc.}$/ g cm^{-3}	1.437
μ/mm^{-1}	5.789
Formula Weight	532.50
Colour	clear intense yellow
Shape	prism
Size/mm³	0.14×0.10×0.07
T/K	140.01(10)
Crystal System	monoclinic
Space Group	$C2/c$
a/Å	33.0434(3)
b/Å	13.06155(11)
c/Å	11.56396(11)
α/°	90
β/°	99.3953(9)
γ/°	90
V/Å³	4924.04(8)
Z	8
Z'	1
Wavelength/Å	1.54184
Radiation type	Cu$K\alpha$
Θ_{min}/°	3.646
Θ_{max}/°	72.640
Measured Refl's.	20471
Indep't Refl's	4843
Refl's I≥2σ(I)	4552
R_{int}	0.0258
Parameters	299
Restraints	0
Largest Peak/e Å^{-3}	0.360
Deepest Hole/e Å^{-3}	-0.316
GooF	1.038
wR_2 (all data)	0.0484
wR_2	0.0473
R_1 (all data)	0.0215
R_1	0.0196
CCDC number	2359457

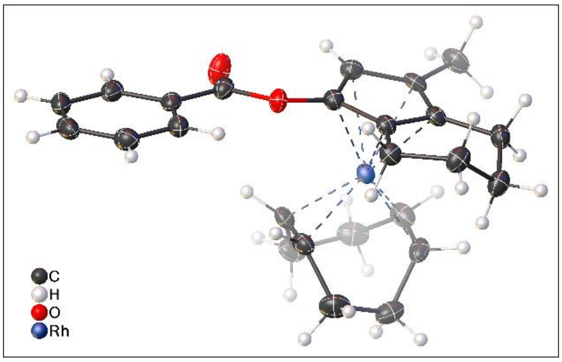

Experimental. Single clear intense yellow prism-shaped crystals of *rac*-**2f** were used as supplied. A suitable crystal with dimensions 0.10 × 0.07 × 0.05 mm³ was selected and mounted on a XtaLAB Synergy R, DW system, HyPix-Arc 150 diffractometer. The crystal was kept at a steady T = 140.01(10) K during data collection. The structure was solved with the ShelXT 2018/2 (Sheldrick, 2015) solution program using dual methods and by using Olex2 1.5 (Dolomanov et al., 2009) as the graphical interface. The model was refined with ShelXL 2019/3 (Sheldrick, 2015) using full matrix least squares minimisation on $\boldsymbol{F}^2$.

Crystal Data. $C_{25}H_{29}O_2Rh$, M_r = 464.39, monoclinic, $P2_1/n$ (No. 14), a = 10.84647(17) Å, b = 15.4224(3) Å, c = 12.23753(18) Å, β = 100.6438(15)°, $\alpha = \gamma$ = 90°, V = 2011.85(6) Å³, T = 140.01(10) K, Z = 4, Z' = 1, μ(Cu K_α) = 6.993, 16100 reflections measured, 3952 unique (R_{int} = 0.0265) which were used in all calculations. The final wR_2 was 0.0671 (all data) and R_1 was 0.0266 (I≥2 σ(I)).

**Compound**	*rac*-**2f**
Formula	$C_{25}H_{29}O_2Rh$
$D_{calc.}$/ g cm⁻³	1.533
μ/mm⁻¹	6.993
Formula Weight	464.39
Colour	clear intense yellow
Shape	prism-shaped
Size/mm³	0.10×0.07×0.05
T/K	140.01(10)
Crystal System	monoclinic
Space Group	$P2_1/n$
a/Å	10.84647(17)
b/Å	15.4224(3)
c/Å	12.23753(18)
α/°	90
β/°	100.6438(15)
γ/°	90
V/Å³	2011.85(6)
Z	4
Z'	1
Wavelength/Å	1.54184
Radiation type	Cu K_α
Θ_{min}/°	4.662
Θ_{max}/°	74.610
Measured Refl's.	16100
Indep't Refl's	3952
Refl's I≥2 σ(I)	3603
R_{int}	0.0265
Parameters	254
Restraints	0
Largest Peak	1.582
Deepest Hole	-0.627
GooF	1.030
wR_2 (all data)	0.0671
wR_2	0.0657
R_1 (all data)	0.0299
R_1	0.0266
CCDC number	2359461

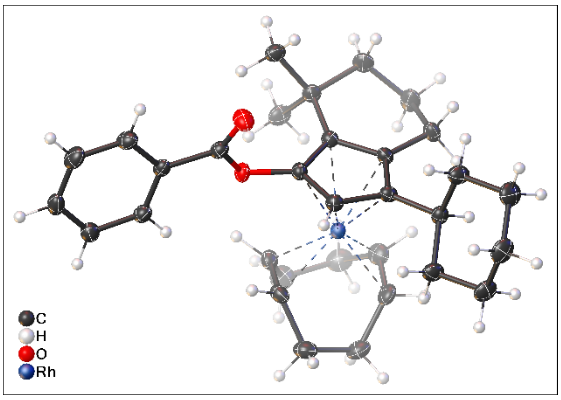

Experimental. Single clear intense yellow prism-shaped crystals of **2g** were used as supplied. A suitable crystal with dimensions 0.24 × 0.15 × 0.14 mm^3 was selected and mounted on a XtaLAB Synergy R, DW system, HyPix-Arc 150 diffractometer. The crystal was kept at a steady T = 140.00(10) K during data collection. The structure was solved with the ShelXT 2018/2 (Sheldrick, 2015) solution program using dual methods and by using Olex2 1.5 (Dolomanov et al., 2009) as the graphical interface. The model was refined with ShelXL 2019/3 (Sheldrick, 2015) using full matrix least squares minimisation on F^2.

Crystal Data. $C_{32}H_{41}O_2Rh$, M_r = 560.56, tetragonal, $P4_3$ (No. 78), a = 8.61383(4) Å, b = 8.61383(4) Å, c = 36.3572(3) Å, $\alpha = \beta = \gamma = 90°$, V = 2697.64(3) Å^3, T = 140.00(10) K, Z = 4, Z' = 1, μ(Cu K$_\alpha$) = 5.310, 23024 reflections measured, 5245 unique (R_{int} = 0.0196) which were used in all calculations. The final wR_2 was 0.0367 (all data) and R_1 was 0.0144 (I≥2 σ(I)).

**Compound**	**2g**
Formula	$C_{32}H_{41}O_2Rh$
$D_{calc.}$/ g cm^{-3}	1.380
μ/mm^{-1}	5.310
Formula Weight	560.56
Colour	clear intense yellow
Shape	prism-shaped
Size/mm^3	0.24×0.15×0.14
T/K	140.00(10)
Crystal System	tetragonal
Flack Parameter	-0.035(2)
Space Group	$P4_3$
a/Å	8.61383(4)
b/Å	8.61383(4)
c/Å	36.3572(3)
α/°	90
β/°	90
γ/°	90
V/Å^3	2697.64(3)
Z	4
Z'	1
Wavelength/Å	1.54184
Radiation type	Cu K$_\alpha$
Θ_{min}/°	4.865
Θ_{max}/°	73.364
Measured Refl's.	23024
Indep't Refl's	5245
Refl's I≥2 σ(I)	5213
R_{int}	0.0196
Parameters	319
Restraints	1
Largest Peak	0.180
Deepest Hole	-0.201
GooF	1.049
wR_2 (all data)	0.0367
wR_2	0.0366
R_1 (all data)	0.0145
R_1	0.0144
CCDC number	2359462

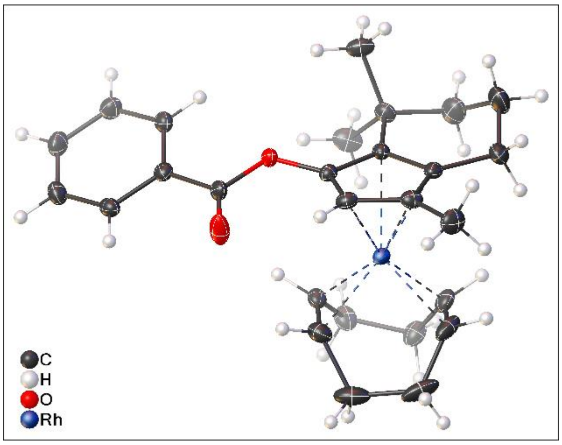

Experimental. Single clear intense yellow plate-shaped crystals of **2h** were used as supplied. A suitable crystal with dimensions 0.05 × 0.04 × 0.01 mm³ was selected and mounted on a XtaLAB Synergy R, DW system, HyPix-Arc 150 diffractometer. The crystal was kept at a steady *T* = 140.00(10) K during data collection. The structure was solved with the ShelXT 2018/2 (Sheldrick, 2015) solution program using dual methods and by using Olex2 1.5 (Dolomanov et al., 2009) as the graphical interface. The model was refined with ShelXL 2019/3 (Sheldrick, 2015) using full matrix least squares minimisation on F^2.

Crystal Data. $C_{27}H_{33}O_2Rh$, M_r = 492.44, orthorhombic, $P2_12_12_1$ (No. 19), a = 8.84526(12) Å, b = 15.37197(18) Å, c = 16.3872(2) Å, $\alpha = \beta = \gamma = 90°$, V = 2228.16(5) Å³, T = 140.00(10) K, Z = 4, Z' = 1, μ(Cu K$_\alpha$) = 6.347, 16071 reflections measured, 4314 unique (R_{int} = 0.0278) which were used in all calculations. The final wR_2 was 0.0474 (all data) and R_1 was 0.0211 (I≥2 σ(I)).

**Compound**	**2h**
Formula	$C_{27}H_{33}O_2Rh$
$D_{calc.}$/ g cm⁻³	1.468
μ/mm⁻¹	6.347
Formula Weight	492.44
Colour	clear intense yellow
Shape	plate-shaped
Size/mm³	0.05×0.04×0.01
T/K	140.00(10)
Crystal System	orthorhombic
Flack Parameter	-0.028(4)
Space Group	$P2_12_12_1$
a/Å	8.84526(12)
b/Å	15.37197(18)
c/Å	16.3872(2)
α/°	90
β/°	90
γ/°	90
V/Å³	2228.16(5)
Z	4
Z'	1
Wavelength/Å	1.54184
Radiation type	Cu K$_\alpha$
Θ_{min}/°	3.943
Θ_{max}/°	73.986
Measured Refl's.	16071
Indep't Refl's	4314
Refl's I≥2 σ(I)	4107
R_{int}	0.0278
Parameters	274
Restraints	0
Largest Peak	0.275
Deepest Hole	-0.401
GooF	1.052
wR_2 (all data)	0.0474
wR_2	0.0468
R_1 (all data)	0.0233
R_1	0.0211
CCDC number	2359460

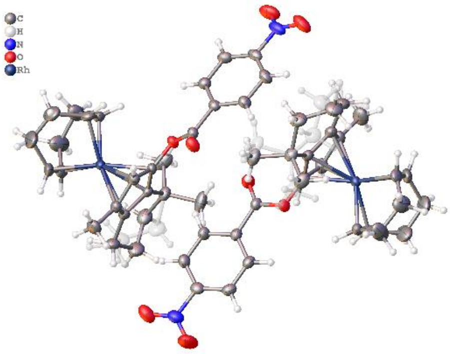

Experimental. Single clear intense red irregular-shaped crystals of *rac*-**2i** were used as supplied. A suitable crystal with dimensions 0.31 × 0.15 × 0.07 mm^3 was selected and mounted on a SuperNova, Dual, Cu at home/near, AtlasS2 diffractometer. The crystal was kept at a steady T = 140.00(10) K during data collection. The structure was solved with the ShelXT (Sheldrick, 2015) solution program using dual methods and by using Olex2 1.5 (Dolomanov et al., 2009) as the graphical interface. The model was refined with ShelXL 2019/3 (Sheldrick, 2015) using full matrix least squares minimisation on F^2.

Crystal Data. $C_{27}H_{32}NO_4Rh$, M_r = 537.44, triclinic, P-1 (No. 2), a = 11.8888(3) Å, b = 12.5067(2) Å, c = 16.1750(4) Å, α = 81.9942(18)°, β = 88.834(2)°, γ = 83.3164(18)°, V = 2365.41(9) Å^3, T = 140.00(10) K, Z = 4, Z' = 2, μ(Cu K$_\alpha$) = 6.111, 9477 reflections measured, 9477 unique (R_{int} = .) which were used in all calculations. The final wR_2 was 0.0883 (all data) and R_1 was 0.0335 (I≥2 σ(I)).

**Compound**	*rac*-**2i**
Formula	$C_{27}H_{32}NO_4Rh$
$D_{calc.}$/ g cm^{-3}	1.509
μ/mm^{-1}	6.111
Formula Weight	537.44
Colour	clear intense red
Shape	irregular
Size/mm^3	0.31×0.15×0.07
T/K	140.00(10)
Crystal System	triclinic
Space Group	P-1
a/Å	11.8888(3)
b/Å	12.5067(2)
c/Å	16.1750(4)
α/°	81.9942(18)
β/°	88.834(2)
γ/°	83.3164(18)
V/Å^3	2365.41(9)
Z	4
Z'	2
Wavelength/Å	1.54184
Radiation type	Cu K$_\alpha$
Θ_{min}/°	3.593
Θ_{max}/°	72.826
Measured Refl's.	9477
Indep't Refl's	9477
Refl's I≥2 σ(I)	8089
R_{int}	.
Parameters	730
Restraints	640
Largest Peak	0.579
Deepest Hole	-0.653
GooF	0.967
wR_2 (all data)	0.0883
wR_2	0.0862
R_1 (all data)	0.0381
R_1	0.0335
CCDC number	2371482

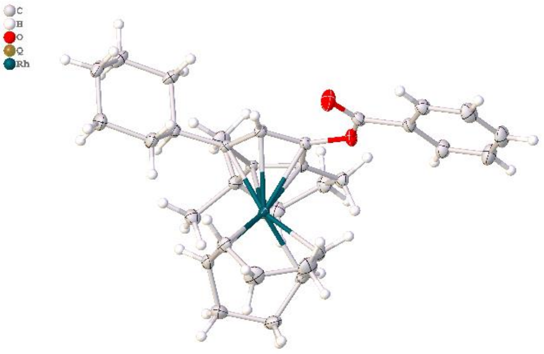

Experimental. Single clear intense yellow prism-shaped crystals of *rac*-**2j** were used as supplied. A suitable crystal with dimensions 0.21 × 0.17 × 0.09 mm^3 was selected and mounted on a SuperNova, Dual, Cu at home/near, AtlasS2 diffractometer. The crystal was kept at a steady T = 140.00(10) K during data collection. The structure was solved with the ShelXT (Sheldrick, 2015) solution program using dual methods and by using Olex2 1.5 (Dolomanov et al., 2009) as the graphical interface. The model was refined with ShelXL 2019/3 (Sheldrick, 2015) using full matrix least squares minimisation on $\boldsymbol{F}^2$.

Crystal Data. $C_{32}H_{41}O_2Rh$, M_r = 560.56, monoclinic, $P2_1/c$ (No. 14), a = 20.7031(4) Å, b = 10.3731(2) Å, c = 12.7123(2) Å, β = 106.307(2)°, $\alpha = \gamma$ = 90°, V = 2620.21(9) Å^3, T = 140.00(10) K, Z = 4, Z' = 1, μ(Mo K$_\alpha$) = 0.679, 34430 reflections measured, 8935 unique (R_{int} = 0.0366) which were used in all calculations. The final wR_2 was 0.0651 (all data) and R_1 was 0.0300 (I≥2 σ(I)).

**Compound**	*rac*-**2j**
Formula	$C_{32}H_{41}O_2Rh$
$D_{calc.}$/ g cm^{-3}	1.421
μ/mm^{-1}	0.679
Formula Weight	560.56
Colour	clear intense yellow
Shape	prism
Size/mm^3	0.21×0.17×0.09
T/K	140.00(10)
Crystal System	monoclinic
Space Group	$P2_1/c$
a/Å	20.7031(4)
b/Å	10.3731(2)
c/Å	12.7123(2)
α/°	90
β/°	106.307(2)
γ/°	90
V/Å^3	2620.21(9)
Z	4
Z'	1
Wavelength/Å	0.71073
Radiation type	Mo K$_\alpha$
Θ_{min}/°	2.839
Θ_{max}/°	32.805
Measured Refl's.	34430
Indep't Refl's	8935
Refl's I≥2 σ(I)	7369
R_{int}	0.0366
Parameters	480
Restraints	0
Largest Peak	0.492
Deepest Hole	-0.394
GooF	1.072
wR_2 (all data)	0.0651
wR_2	0.0596
R_1 (all data)	0.0438
R_1	0.0300
CCDC number	2371491

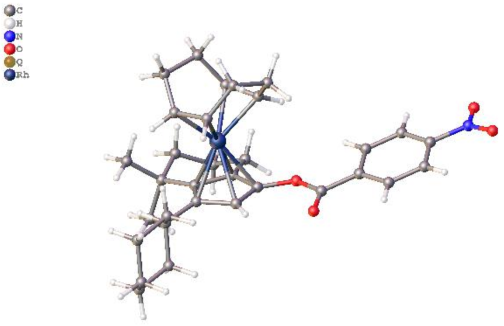

Experimental. Single clear dark purple prism-shaped crystals of *rac*-**2k** were used as supplied. A suitable crystal with dimensions 0.21 × 0.14 × 0.08 mm³ was selected and mounted on a SuperNova, Dual, Cu at home/near, AtlasS2 diffractometer. The crystal was kept at a steady T = 140.00(10) K during data collection. The structure was solved with the ShelXT (Sheldrick, 2015) solution program using dual methods and by using Olex2 1.5 (Dolomanov et al., 2009) as the graphical interface. The model was refined with ShelXL 2019/3 (Sheldrick, 2015) using full matrix least squares minimisation on F^2.

Crystal Data. $C_{32}H_{40}NO_4Rh$, M_r = 605.56, orthorhombic, *Pbca* (No. 61), a = 10.64524(16) Å, b = 12.16432(19) Å, c = 42.3493(6) Å, $\alpha = \beta = \gamma$ = 90°, V = 5483.90(14) Å³, T = 140.00(10) K, Z = 8, Z' = 1, μ(Cu K_α) = 5.338, 43733 reflections measured, 5435 unique (R_{int} = 0.0471) which were used in all calculations. The final wR_2 was 0.0715 (all data) and R_1 was 0.0281 (I≥2 σ(I)).

**Compound**	*rac*-**2k**
Formula	$C_{32}H_{40}NO_4Rh$
$D_{calc.}$/ g cm^{-3}	1.467
μ/mm^{-1}	5.338
Formula Weight	605.56
Colour	clear dark purple
Shape	prism
Size/mm³	0.21×0.14×0.08
T/K	140.00(10)
Crystal System	orthorhombic
Space Group	*Pbca*
a/Å	10.64524(16)
b/Å	12.16432(19)
c/Å	42.3493(6)
α/°	90
β/°	90
γ/°	90
V/Å³	5483.90(14)
Z	8
Z'	1
Wavelength/Å	1.54184
Radiation type	Cu K_α
Θ_{min}/°	4.176
Θ_{max}/°	72.915
Measured Refl's.	43733
Indep't Refl's	5435
Refl's I≥2 σ(I)	4432
R_{int}	0.0471
Parameters	346
Restraints	0
Largest Peak	0.302
Deepest Hole	-0.513
GooF	1.044
wR_2 (all data)	0.0715
wR_2	0.0668
R_1 (all data)	0.0373
R_1	0.0281
CCDC number	2371486

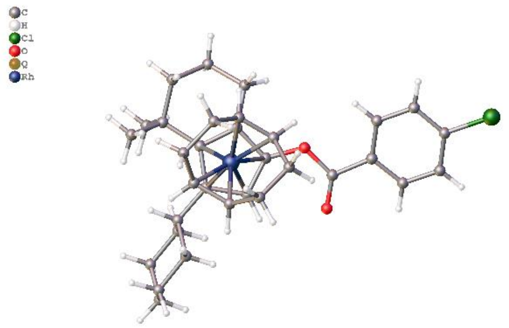

Experimental. Single clear intense yellow irregular-shaped crystals of *rac*-**2l** were used as supplied. A suitable crystal with dimensions 0.25 × 0.10 × 0.05 mm³ was selected and mounted on a SuperNova, Dual, Cu at home/near, Atlas diffractometer. The crystal was kept at a steady T = 200.00(10) K during data collection. The structure was solved with the ShelXT (Sheldrick, 2015) solution program using dual methods and by using Olex2 1.5 (Dolomanov et al., 2009) as the graphical interface. The model was refined with ShelXL 2019/3 (Sheldrick, 2015) using full matrix least squares minimisation on F^2.

Crystal Data. $C_{32}H_{40}ClO_2Rh$, M_r = 595.00, orthorhombic, *Pbca* (No. 61), a = 10.9067(4) Å, b = 12.0454(5) Å, c = 42.0425(11) Å, $\alpha = \beta = \gamma$ = 90°, V = 5523.4(3) Å³, T = 200.00(10) K, Z = 8, Z' = 1, μ(Cu K_α) = 6.091, 15080 reflections measured, 5283 unique (R_{int} = 0.0664) which were used in all calculations. The final wR_2 was 0.2564 (all data) and R_1 was 0.0580 (I≥2 σ(I)).

**Compound**	*rac*-**2l**
Formula	$C_{32}H_{40}ClO_2Rh$
$D_{calc.}$/ g cm^{-3}	1.431
μ/mm^{-1}	6.091
Formula Weight	595.00
Colour	clear intense yellow
Shape	irregular
Size/mm³	0.25×0.10×0.05
T/K	200.00(10)
Crystal System	orthorhombic
Space Group	*Pbca*
a/Å	10.9067(4)
b/Å	12.0454(5)
c/Å	42.0425(11)
α/°	90
β/°	90
γ/°	90
V/Å³	5523.4(3)
Z	8
Z'	1
Wavelength/Å	1.54184
Radiation type	Cu K_α
Θ_{min}/°	4.206
Θ_{max}/°	73.277
Measured Refl's.	15080
Indep't Refl's	5283
Refl's I≥2 σ(I)	3920
R_{int}	0.0664
Parameters	327
Restraints	0
Largest Peak	0.952
Deepest Hole	-1.153
GooF	1.065
wR_2 (all data)	0.2564
wR_2	0.2020
R_1 (all data)	0.0831
R_1	0.0580
CCDC number	2371487

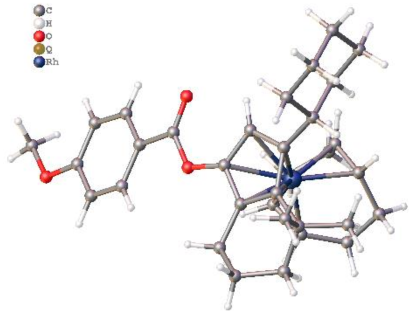

Experimental. Single clear intense yellow prism-shaped crystals of *rac*-**2m** were used as supplied. A suitable crystal with dimensions 0.28 × 0.18 × 0.11 mm^3 was selected and mounted on a SuperNova, Dual, Cu at home/near, AtlasS2 diffractometer. The crystal was kept at a steady T = 140.00(10) K during data collection. The structure was solved with the ShelXT (Sheldrick, 2015) solution program using dual methods and by using Olex2 1.5 (Dolomanov et al., 2009) as the graphical interface. The model was refined with ShelXL 2019/3 (Sheldrick, 2015) using full matrix least squares minimisation on $\boldsymbol{F}^2$.

Crystal Data. $C_{33}H_{43}O_3Rh$, M_r = 590.58, orthorhombic, *Pccn* (No. 56), a = 10.44218(11) Å, b = 41.9945(4) Å, c = 12.68539(14) Å, $\alpha = \beta = \gamma =$ 90°, V = 5562.72(10) Å^3, T = 140.00(10) K, Z = 8, Z' = 1, μ(Cu K$_\alpha$) = 5.207, 19581 reflections measured, 5730 unique (R_{int} = 0.0358) which were used in all calculations. The final wR_2 was 0.0871 (all data) and R_1 was 0.0325 (I≥2 σ(I)).

**Compound**	*rac*-**2m**
Formula	$C_{33}H_{43}O_3Rh$
$D_{calc.}$/ g cm^{-3}	1.410
μ/mm^{-1}	5.207
Formula Weight	590.58
Colour	clear intense yellow
Shape	prism
Size/mm^3	0.28×0.18×0.11
T/K	140.00(10)
Crystal System	orthorhombic
Space Group	*Pccn*
a/Å	10.44218(11)
b/Å	41.9945(4)
c/Å	12.68539(14)
α/°	90
β/°	90
γ/°	90
V/Å^3	5562.72(10)
Z	8
Z'	1
Wavelength/Å	1.54184
Radiation type	Cu K$_\alpha$
Θ_{min}/°	4.211
Θ_{max}/°	76.024
Measured Refl's.	19581
Indep't Refl's	5730
Refl's I≥2 σ(I)	5143
R_{int}	0.0358
Parameters	338
Restraints	0
Largest Peak	0.551
Deepest Hole	-0.856
GooF	1.049
wR_2 (all data)	0.0871
wR_2	0.0835
R_1 (all data)	0.0368
R_1	0.0325
CCDC number	2371488

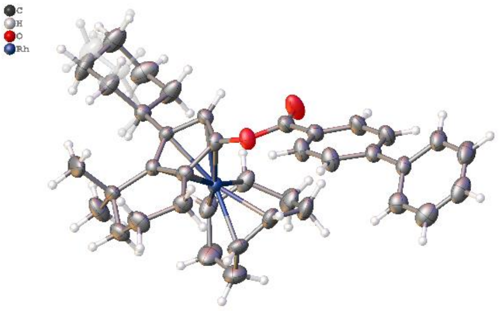

Experimental. Single clear intense yellow irregular-shaped crystals of **2n** were used as supplied. A suitable crystal with dimensions 0.22 × 0.18 × 0.11 mm^3 was selected and mounted on a SuperNova, Dual, Cu at home/near, Atlas diffractometer. The crystal was kept at a steady T = 200.00(10) K during data collection. The structure was solved with the ShelXS (Sheldrick, 2008) solution program using heavy methods and by using Olex2 1.5 (Dolomanov et al., 2009) as the graphical interface. The model was refined with ShelXL 2019/3 (Sheldrick, 2015) using full matrix least squares minimisation on $\boldsymbol{F}^2$.

Crystal Data. $C_{38}H_{45}O_2Rh$, M_r = 636.65, monoclinic, $P2_1$ (No. 4), a = 10.48340(17) Å, b = 11.6375(2) Å, c = 12.7606(2) Å, β = 98.1048(15)°, α = γ = 90°, V = 1541.25(4) $Å^3$, T = 200.00(10) K, Z = 2, Z' = 1, μ(Cu K_α) = 4.717, 16647 reflections measured, 6171 unique (R_{int} = 0.0239) which were used in all calculations. The final wR_2 was 0.0552 (all data) and R_1 was 0.0224 (I≥2 σ(I)).

**Compound**	**2n**
Formula	$C_{38}H_{45}O_2Rh$
$D_{calc.}$/ g cm^{-3}	1.372
μ/mm^{-1}	4.717
Formula Weight	636.65
Colour	clear intense yellow
Shape	irregular
Size/mm^3	0.22×0.18×0.11
T/K	200.00(10)
Crystal System	monoclinic
Flack Parameter	-0.022(5)
Hooft Parameter	-0.022(5)
Space Group	$P2_1$
a/Å	10.48340(17)
b/Å	11.6375(2)
c/Å	12.7606(2)
α/°	90
β/°	98.1048(15)
γ/°	90
V/$Å^3$	1541.25(4)
Z	2
Z'	1
Wavelength/Å	1.54184
Radiation type	Cu K_α
Θ_{min}/°	3.499
Θ_{max}/°	76.856
Measured Refl's.	16647
Indep't Refl's	6171
Refl's I≥2 σ(I)	5956
R_{int}	0.0239
Parameters	391
Restraints	1
Largest Peak	0.430
Deepest Hole	-0.332
GooF	1.024
wR_2 (all data)	0.0552
wR_2	0.0544
R_1 (all data)	0.0239
R_1	0.0224
CCDC number	2371483

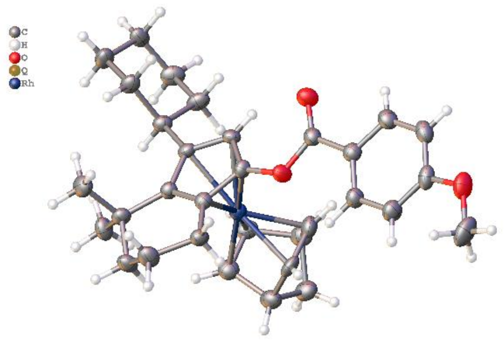

Experimental. Single clear pale yellow irregular-shaped crystals of *rac*-**2p** were used as supplied. A suitable crystal with dimensions 0.21 × 0.10 × 0.06 mm^3 was selected and mounted on a XtaLAB Synergy R, DW system, HyPix-Arc 150 diffractometer. The crystal was kept at a steady T = 140.00(10) K during data collection. The structure was solved with the ShelXT (Sheldrick, 2015) solution program using dual methods and by using Olex2 1.5 (Dolomanov et al., 2009) as the graphical interface. The model was refined with ShelXL 2019/3 (Sheldrick, 2015) using full matrix least squares minimisation on $\boldsymbol{F}^2$.

Crystal Data. $C_{32}H_{39}O_3Rh$, M_r = 574.54, monoclinic, $P2_1/c$ (No. 14), a = 21.8538(5) Å, b = 10.41589(19) Å, c = 11.53436(19) Å, β = 97.9707(19)°, $\alpha = \gamma$ = 90°, V = 2600.16(9) Å^3, T = 140.00(10) K, Z = 4, Z' = 1, μ(Cu K$_\alpha$) = 5.556, 30158 reflections measured, 5333 unique (R_{int} = 0.0623) which were used in all calculations. The final wR_2 was 0.1195 (all data) and R_1 was 0.0459 (I≥2 σ(I)).

**Compound**	*rac*-**2p**
Formula	$C_{32}H_{39}O_3Rh$
$D_{calc.}$/ g cm^{-3}	1.468
μ/mm^{-1}	5.556
Formula Weight	574.54
Colour	clear pale yellow
Shape	irregular-shaped
Size/mm^3	0.21×0.10×0.06
T/K	140.00(10)
Crystal System	monoclinic
Space Group	$P2_1/c$
a/Å	21.8538(5)
b/Å	10.41589(19)
c/Å	11.53436(19)
α/°	90
β/°	97.9707(19)
γ/°	90
V/Å^3	2600.16(9)
Z	4
Z'	1
Wavelength/Å	1.54184
Radiation type	Cu K$_\alpha$
Θ_{min}/°	2.041
Θ_{max}/°	75.776
Measured Refl's.	30158
Indep't Refl's	5333
Refl's I≥2 σ(I)	4070
R_{int}	0.0623
Parameters	329
Restraints	0
Largest Peak	0.958
Deepest Hole	-1.388
GooF	1.072
wR_2 (all data)	0.1195
wR_2	0.1113
R_1 (all data)	0.0667
R_1	0.0459
CCDC number	2371485

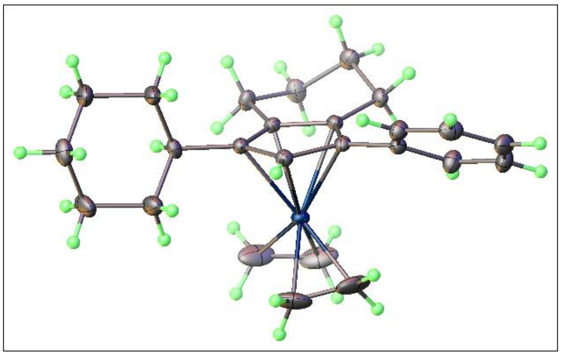

Experimental. Single colourless prism-shaped crystals of *rac*-**5b** were used as supplied. A suitable crystal with dimensions 0.19 × 0.17 × 0.08 mm³ was selected and mounted on a SuperNova, Dual, Cu at home/near, Atlas diffractometer. The crystal was kept at a steady T = 139.99(10) K during data collection. The structure was solved with the **ShelXT** 2018/2 (Sheldrick, 2015) solution program using dual methods and by using **Olex2** 1.5 (Dolomanov et al., 2009) as the graphical interface. The model was refined with **ShelXL** 2018/3 (Sheldrick, 2015) using full-matrix least-squares minimisation on $\boldsymbol{F}^2$.

Crystal Data. $C_{25}H_{33}Ir$, M_r = 525.71, orthorhombic, *Pbca* (No. 61), a = 11.2766(2) Å, b = 18.1893(4) Å, c = 20.1751(4) Å, $\alpha = \beta = \gamma$ = 90°, V = 4138.18(15) Å³, T = 139.99(10) K, Z = 8, Z' = 1, μ(Mo K_α) = 6.459, 66025 reflections measured, 7250 unique (R_{int} = 0.0400) which were used in all calculations. The final wR_2 was 0.0440 (all data) and R_1 was 0.0221 ($I \geq 2\sigma(I)$).

**Compound**	*rac*-**5b**
Formula	$C_{25}H_{33}Ir$
$D_{calc.}$/ g cm^{-3}	1.688
μ/mm^{-1}	6.459
Formula Weight	525.71
Colour	colourless
Shape	prism-shaped
Size/mm³	0.19×0.17×0.08
T/K	139.99(10)
Crystal System	orthorhombic
Space Group	*Pbca*
a/Å	11.2766(2)
b/Å	18.1893(4)
c/Å	20.1751(4)
α/°	90
β/°	90
γ/°	90
V/Å³	4138.18(15)
Z	8
Z'	1
Wavelength/Å	0.71073
Radiation type	MoK_α
Θ_{min}/°	2.709
Θ_{max}/°	32.758
Measured Refl's.	66025
Indep't Refl's	7250
Refl's $I \geq 2\sigma(I)$	5509
R_{int}	0.0400
Parameters	236
Restraints	0
Largest Peak/e Å^{-3}	1.073
Deepest Hole/e Å^{-3}	-0.780
GooF	1.053
wR_2 (all data)	0.0440
wR_2	0.0387
R_1 (all data)	0.0402
R_1	0.0221
CCDC number	2154256

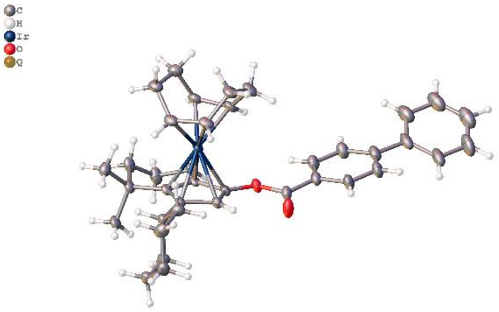

Experimental. Single clear intense yellow irregular-shaped crystals of *rac*-**5f** were used as supplied. A suitable crystal with dimensions 0.09 × 0.07 × 0.06 mm^3 was selected and mounted on a SuperNova, Dual, Cu at home/near, AtlasS2 diffractometer. The crystal was kept at a steady T = 140.00(10) K during data collection. The structure was solved with the ShelXT (Sheldrick, 2015) solution program using dual methods and by using Olex2 1.5 (Dolomanov et al., 2009) as the graphical interface. The model was refined with ShelXL 2019/3 (Sheldrick, 2015) using full matrix least squares minimisation on $\boldsymbol{F}^2$.

Crystal Data. $C_{38}H_{45}IrO_2$, M_r = 725.94, monoclinic, $I2/a$ (No. 15), a = 12.0743(5) Å, b = 10.7400(4) Å, c = 47.3644(11) Å, β = 97.224(3)°, $\alpha = \gamma$ = 90°, V = 6093.4(4) Å^3, T = 140.00(10) K, Z = 8, Z' = 1, μ(Cu K_α) = 8.725, 22593 reflections measured, 5983 unique (R_{int} = 0.0963) which were used in all calculations. The final wR_2 was 0.1110 (all data) and R_1 was 0.0465 (I≥2 σ(I)).

**Compound**	*rac*-**5f**
Formula	$C_{38}H_{45}IrO_2$
$D_{calc.}$/ g cm^{-3}	1.583
μ/mm^{-1}	8.725
Formula Weight	725.94
Colour	clear intense yellow
Shape	irregular
Size/mm^3	0.09×0.07×0.06
T/K	140.00(10)
Crystal System	monoclinic
Space Group	$I2/a$
a/Å	12.0743(5)
b/Å	10.7400(4)
c/Å	47.3644(11)
α/°	90
β/°	97.224(3)
γ/°	90
V/Å^3	6093.4(4)
Z	8
Z'	1
Wavelength/Å	1.54184
Radiation type	Cu K_α
Θ_{min}/°	3.763
Θ_{max}/°	72.937
Measured Refl's.	22593
Indep't Refl's	5983
Refl's I≥2 σ(I)	4365
R_{int}	0.0963
Parameters	372
Restraints	418
Largest Peak	1.365
Deepest Hole	-2.470
GooF	1.022
wR_2 (all data)	0.1110
wR_2	0.0996
R_1 (all data)	0.0743
R_1	0.0465
CCDC number	2371490

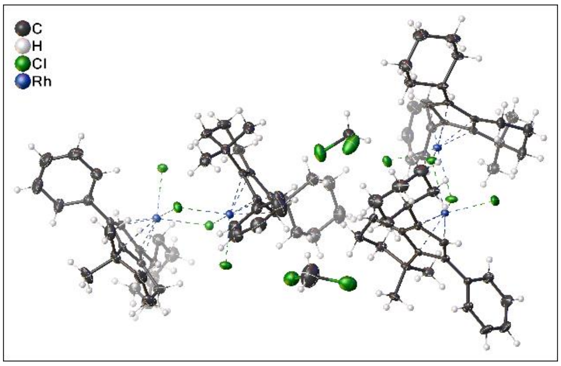

Experimental. Single clear intense orange plate-shaped crystals of **3b** were used as supplied. A suitable crystal with dimensions 0.08 × 0.06 × 0.01 mm^3 was selected and mounted on a XtaLAB Synergy R, DW system, HyPix-Arc 150 diffractometer. The crystal was kept at a steady T = 140.00(10) K during data collection. The structure was solved with the ShelXT 2018/2 (Sheldrick, 2015) solution program using dual methods and by using Olex2 1.5 (Dolomanov et al., 2009) as the graphical interface. The model was refined with ShelXL 2019/3 (Sheldrick, 2015) using full matrix least squares minimisation on $\boldsymbol{F}^2$.

Crystal Data. $C_{47}H_{60}Cl_6Rh_2$, M_r = 1043.47, orthorhombic, $P2_12_12$ (No. 18), a = 38.0154(3) Å, b = 15.33747(13) Å, c = 11.71410(11) Å, $\alpha = \beta = \gamma = 90°$, V = 6830.02(10) Å^3, T = 140.00(10) K, Z = 6, Z' = 1.5, μ(Cu K$_\alpha$) = 9.339, 50709 reflections measured, 13275 unique (R_{int} = 0.0391) which were used in all calculations. The final wR_2 was 0.0672 (all data) and R_1 was 0.0298 (I≥2 σ(I)).

Compound	3b
Formula	$C_{47}H_{60}Cl_6Rh_2$
$D_{calc.}$/ g cm^{-3}	1.522
μ/mm^{-1}	9.339
Formula Weight	1043.47
Colour	clear intense orange
Shape	plate-shaped
Size/mm^3	0.08×0.06×0.01
T/K	140.00(10)
Crystal System	orthorhombic
Flack Parameter	-0.029(4)
Space Group	$P2_12_12$
a/Å	38.0154(3)
b/Å	15.33747(13)
c/Å	11.71410(11)
α/°	90
β/°	90
γ/°	90
V/Å^3	6830.02(10)
Z	6
Z'	1.5
Wavelength/Å	1.54184
Radiation type	Cu K$_\alpha$
Θ_{min}/°	3.107
Θ_{max}/°	73.242
Measured Refl's.	50709
Indep't Refl's	13275
Refl's I≥2 σ(I)	12263
R_{int}	0.0391
Parameters	751
Restraints	6
Largest Peak	0.581
Deepest Hole	-0.830
GooF	1.019
wR_2 (all data)	0.0672
wR_2	0.0657
R_1 (all data)	0.0345
R_1	0.0298
CCDC number	2359463

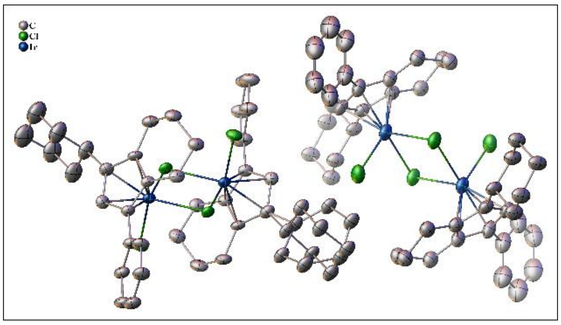

Experimental. Single clear light yellow prism-shaped crystals of *rac*-**4a** were used as supplied. A suitable crystal with dimensions 0.06 × 0.01 × 0.01 mm^3 was selected and mounted on a XtaLAB Synergy R, DW system, HyPix-Arc 150 diffractometer. The crystal was kept at a steady T = 140.00(10) K during data collection. The structure was solved with the **ShelXT** 2018/2 (Sheldrick, 2015) solution program using dual methods and by using **Olex2** 1.5 (Dolomanov et al., 2009) as the graphical interface. The model was refined with **ShelXL** 2018/3 (Sheldrick, 2015) using full matrix least squares minimisation on $\boldsymbol{F}^2$.

Crystal Data. $C_{42}H_{50}Cl_4Ir_2$, M_r = 1081.02, monoclinic, $P2_1/c$ (No. 14), a = 11.9048(3) Å, b = 13.2894(3) Å, c = 40.4105(11) Å, β = 91.560(3)°, $\alpha = \gamma$ = 90°, V = 6390.9(3) Å^3, T = 140.00(10) K, Z = 6, Z' = 1.5, μ(Cu K_α) = 14.418, 39369 reflections measured, 11275 unique (R_{int} = 0.0685) which were used in all calculations. The final wR_2 was 0.1432 (all data) and R_1 was 0.0584 (I≥2σ(I)).

**Compound**	*rac*-**4a**
Formula	$C_{42}H_{50}Cl_4Ir_2$
$D_{calc.}$/ g cm^{-3}	1.685
μ/mm^{-1}	14.418
Formula Weight	1081.02
Colour	clear light yellow
Shape	prism-shaped
Size/mm^3	0.06×0.01×0.01
T/K	140.00(10)
Crystal System	monoclinic
Space Group	$P2_1/c$
a/Å	11.9048(3)
b/Å	13.2894(3)
c/Å	40.4105(11)
α/°	90
β/°	91.560(3)
γ/°	90
V/Å^3	6390.9(3)
Z	6
Z'	1.5
Wavelength/Å	1.54184
Radiation type	Cu$K\alpha$
Θ_{min}/°	2.187
Θ_{max}/°	66.593
Measured Refl's.	39369
Indep't Refl's	11275
Refl's I≥2σ(I)	7238
R_{int}	0.0685
Parameters	668
Restraints	594
Largest Peak/e Å^{-3}	2.427
Deepest Hole/e Å^{-3}	-1.929
GooF	1.030
wR_2 (all data)	0.1432
wR_2	0.1294
R_1 (all data)	0.0991
R_1	0.0584
CCDC number	2144637

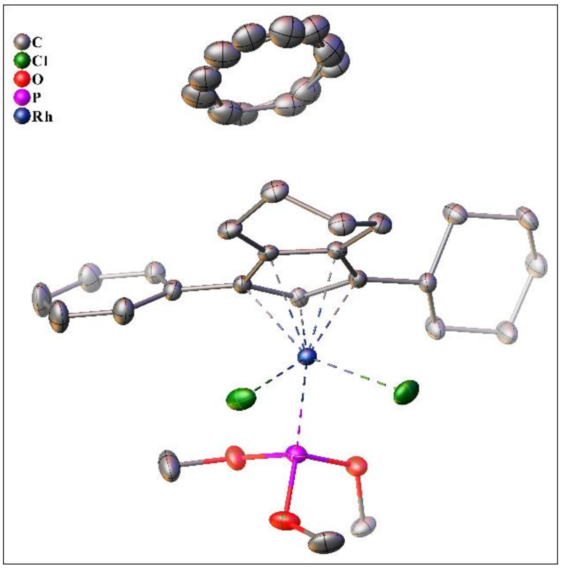

Experimental. Single clear intense orange needle-shaped crystals of *rac*-**10a** were used as supplied. A suitable crystal with dimensions 0.52 × 0.06 × 0.05 mm^3 was selected and mounted on a SuperNova, Dual, Cu at home/near, AtlasS2 diffractometer. The crystal was kept at a steady T = 140.01(10) K during data collection. The structure was solved with the **ShelXT** 2018/2 (Sheldrick, 2015) solution program using dual methods and by using **Olex2** 1.5 (Dolomanov et al., 2009) as the graphical interface. The model was refined with **ShelXL** 2018/3 (Sheldrick, 2015) using full-matrix least-squares minimisation on $\boldsymbol{F^2}$.

Crystal Data. $C_{30}H_{40}Cl_2O_3PRh$, M_r = 653.40, monoclinic, $P2_1/n$ (No. 14), a = 13.0172(3) Å, b = 14.6136(3) Å, c = 15.6821(4) Å, β = 92.806(2)°, $\alpha = \gamma$ = 90°, V = 2979.58(12) Å^3, T = 140.01(10) K, Z = 4, Z' = 1, μ(Mo K_α) = 0.835, 32399 reflections measured, 9966 unique (R_{int} = 0.0436) which were used in all calculations. The final wR_2 was 0.0725 (all data) and R_1 was 0.0389 (I≥2σ(I)).

**Compound**	*rac*-**10a**
Formula	$C_{30}H_{40}Cl_2O_3PRh$
$D_{calc.}$/ g cm^{-3}	1.457
μ/mm^{-1}	0.835
Formula Weight	653.40
Colour	clear intense orange
Shape	needle-shaped
Size/mm^3	0.52×0.06×0.05
T/K	140.01(10)
Crystal System	monoclinic
Space Group	$P2_1/n$
a/Å	13.0172(3)
b/Å	14.6136(3)
c/Å	15.6821(4)
α/°	90
β/°	92.806(2)
γ/°	90
V/Å^3	2979.58(12)
Z	4
Z'	1
Wavelength/Å	0.71073
Radiation type	Mo$K\alpha$
Θ_{min}/°	2.788
Θ_{max}/°	32.837
Measured Refl's.	32399
Indep't Refl's	9966
Refl's I≥2σ(I)	7635
R_{int}	0.0436
Parameters	368
Restraints	186
Largest Peak/e Å^{-3}	0.660
Deepest Hole/e Å^{-3}	-0.601
GooF	1.031
wR_2 (all data)	0.0725
wR_2	0.0657
R_1 (all data)	0.0607
R_1	0.0389
CCDC number	2204618

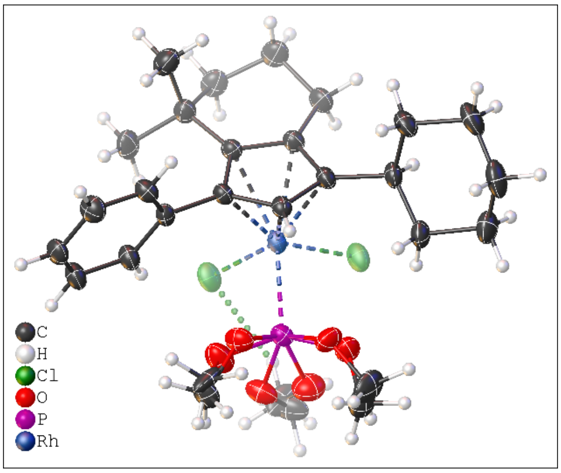

Experimental. Single clear intense colourless plate-shaped crystals of **10b** were used as supplied. A suitable crystal with dimensions 0.14 × 0.04 × 0.01 mm^3 was selected and mounted on a XtaLAB Synergy R, DW system, HyPix-Arc 150 diffractometer. The crystal was kept at a steady T = 139.99(10) K during data collection. The structure was solved with the ShelXT 2018/2 (Sheldrick, 2015) solution program using dual methods and by using Olex2 1.5 (Dolomanov et al., 2009) as the graphical interface. The model was refined with ShelXL 2019/3 (Sheldrick, 2015) using full matrix least squares minimisation on $\boldsymbol{F}^2$.

Crystal Data. $C_{26}H_{38}Cl_2O_3PRh$, M_r = 603.34, monoclinic, $C2$ (No. 5), a = 19.2099(3) Å, b = 8.26778(11) Å, c = 18.4733(3) Å, β = 113.8079(19)°, $\alpha = \gamma$ = 90°, V = 2684.32(8) Å^3, T = 139.99(10) K, Z = 4, Z' = 1, μ(Cu K$_\alpha$) = 7.739, 19969 reflections measured, 5242 unique (R_{int} = 0.0278) which were used in all calculations. The final wR_2 was 0.0595 (all data) and R_1 was 0.0235 (I≥2 σ(I)).

**Compound**	**10b**
Formula	$C_{26}H_{38}Cl_2O_3PRh$
$D_{calc.}$/ g cm^{-3}	1.493
μ/mm^{-1}	7.739
Formula Weight	603.34
Colour	clear intense colourless
Shape	plate-shaped
Size/mm^3	0.14×0.04×0.01
T/K	139.99(10)
Crystal System	monoclinic
Flack Parameter	-0.028(6)
Space Group	$C2$
a/Å	19.2099(3)
b/Å	8.26778(11)
c/Å	18.4733(3)
α/°	90
β/°	113.8079(19)
γ/°	90
V/Å^3	2684.32(8)
Z	4
Z'	1
Wavelength/Å	1.54184
Radiation type	Cu K$_\alpha$
Θ_{min}/°	2.614
Θ_{max}/°	74.743
Measured Refl's.	19969
Indep't Refl's	5242
Refl's I≥2 σ(I)	5018
R_{int}	0.0278
Parameters	361
Restraints	340
Largest Peak	0.487
Deepest Hole	-0.604
GooF	1.009
wR_2 (all data)	0.0595
wR_2	0.0588
R_1 (all data)	0.0251
R_1	0.0235
CCDC number	2359465

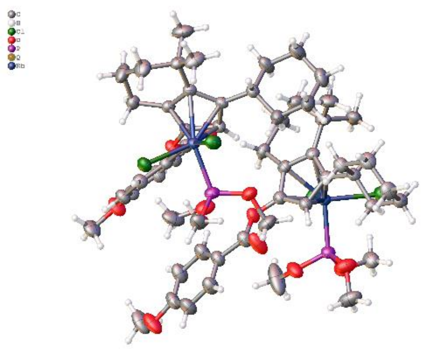

Experimental. Single clear dark red irregular-shaped crystals of *rac*-**10c** were used as supplied. A suitable crystal with dimensions 0.29 × 0.21 × 0.13 mm^3 was selected and mounted on a SuperNova, Dual, Cu at home/near, Atlas diffractometer. The crystal was kept at a steady T = 200.00(10) K during data collection. The structure was solved with the ShelXT (Sheldrick, 2015) solution program using dual methods and by using Olex2 1.5 (Dolomanov et al., 2009) as the graphical interface. The model was refined with ShelXL 2019/3 (Sheldrick, 2015) using full matrix least squares minimisation on $\boldsymbol{F}^2$.

Crystal Data. $C_{28}H_{40}Cl_2O_6PRh$, M_r = 677.38, triclinic, P-1 (No. 2), a = 12.6218(4) Å, b = 14.2384(4) Å, c = 18.3631(5) Å, α = 107.607(2)°, β = 96.748(2)°, γ = 100.605(2)°, V = 3038.51(16) Å^3, T = 200.00(10) K, Z = 4, Z' = 2, μ(Cu K$_\alpha$) = 6.981, 25460 reflections measured, 11709 unique (R_{int} = 0.0331) which were used in all calculations. The final wR_2 was 0.0817 (all data) and R_1 was 0.0326 (I≥2 σ(I)).

**Compound**	*rac*-**10c**
Formula	$C_{28}H_{40}Cl_2O_6PRh$
$D_{calc.}$/ g cm^{-3}	1.481
μ/mm^{-1}	6.981
Formula Weight	677.38
Colour	clear dark red
Shape	irregular
Size/mm^3	0.29×0.21×0.13
T/K	200.00(10)
Crystal System	triclinic
Space Group	P-1
a/Å	12.6218(4)
b/Å	14.2384(4)
c/Å	18.3631(5)
α/°	107.607(2)
β/°	96.748(2)
γ/°	100.605(2)
V/Å^3	3038.51(16)
Z	4
Z'	2
Wavelength/Å	1.54184
Radiation type	Cu K$_\alpha$
Θ_{min}/°	3.348
Θ_{max}/°	72.422
Measured Refl's.	25460
Indep't Refl's	11709
Refl's I≥2 σ(I)	10030
R_{int}	0.0331
Parameters	708
Restraints	19
Largest Peak	0.597
Deepest Hole	-0.531
GooF	1.033
wR_2 (all data)	0.0817
wR_2	0.0768
R_1 (all data)	0.0409
R_1	0.0326
CCDC number	2371484

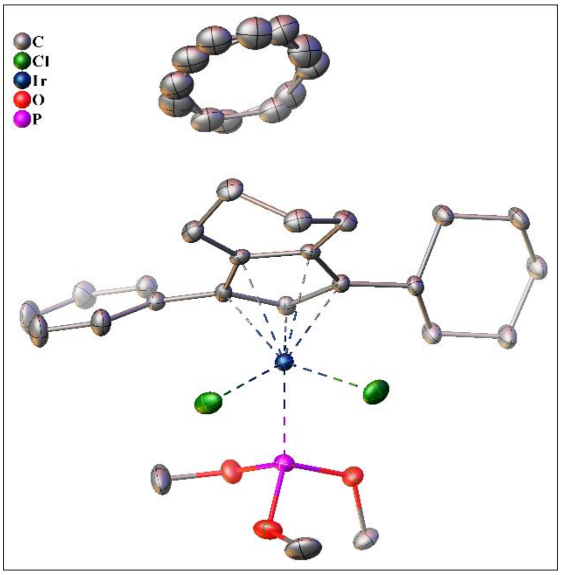

Experimental. Single clear intense yellow needle-shaped crystals of *rac*-**11a** were used as supplied. A suitable crystal with dimensions 0.50 × 0.10 × 0.06 mm^3 was selected and mounted on a SuperNova, Dual, Cu at home/near, Atlas diffractometer. The crystal was kept at a steady T = 140.0(3) K during data collection. The structure was solved with the **ShelXT** 2018/2 (Sheldrick, 2015) solution program using dual methods and by using **Olex2** 1.5 (Dolomanov et al., 2009) as the graphical interface. The model was refined with **ShelXL** 2018/3 (Sheldrick, 2015) using full-matrix least-squares minimisation on $\boldsymbol{F^2}$.

Crystal Data. $C_{30}H_{40}Cl_2IrO_3P$, M_r = 742.69, monoclinic, $P2_1/n$ (No. 14), a = 12.9958(3) Å, b = 14.6810(3) Å, c = 15.6585(3) Å, β = 92.8186(19)°, $\alpha = \gamma$ = 90°, V = 2983.87(11) Å^3, T = 140.0(3) K, Z = 4, Z' = 1, μ(Mo K_α) = 4.737, 27786 reflections measured, 9861 unique (R_{int} = 0.0296) which were used in all calculations. The final wR_2 was 0.0498 (all data) and R_1 was 0.0270 (I≥2σ(I)).

**Compound**	*rac*-**11a**
Formula	$C_{30}H_{40}Cl_2IrO_3P$
$D_{calc.}$/ g cm^{-3}	1.653
μ/mm^{-1}	4.737
Formula Weight	742.69
Colour	clear intense yellow
Shape	needle-shaped
Size/mm^3	0.50×0.10×0.06
T/K	140.0(3)
Crystal System	monoclinic
Space Group	$P2_1/n$
a/Å	12.9958(3)
b/Å	14.6810(3)
c/Å	15.6585(3)
α/°	90
β/°	92.8186(19)
γ/°	90
V/Å^3	2983.87(11)
Z	4
Z'	1
Wavelength/Å	0.71073
Radiation type	Mo$K\alpha$
Θ_{min}/°	2.605
Θ_{max}/°	32.734
Measured Refl's.	27786
Indep't Refl's	9861
Refl's I≥2σ(I)	8119
R_{int}	0.0296
Parameters	368
Restraints	186
Largest Peak/e Å^{-3}	1.728
Deepest Hole/e Å^{-3}	-1.058
GooF	1.039
wR_2 (all data)	0.0498
wR_2	0.0453
R_1 (all data)	0.0409
R_1	0.0270
CCDC number	2204617

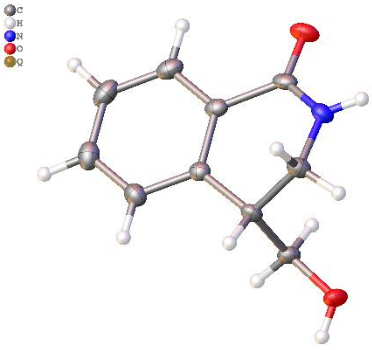

Experimental. Single clear pale colourless prism-shaped crystals of **13d** were used as supplied. A suitable crystal with dimensions 0.45 × 0.17 × 0.16 mm^3 was selected and mounted on a SuperNova, Dual, Cu at home/near, AtlasS2 diffractometer. The crystal was kept at a steady T = 140.00(10) K during data collection. The structure was solved with the ShelXT (Sheldrick, 2015) solution program using dual methods and by using Olex2 1.5 (Dolomanov et al., 2009) as the graphical interface. The model was refined with ShelXL 2019/3 (Sheldrick, 2015) using full matrix least squares minimisation on $\boldsymbol{F}^2$.

Crystal Data. $C_{10}H_{11}NO_2$, M_r = 177.20, orthorhombic, $P2_12_12_1$ (No. 19), a = 7.79506(9) Å, b = 8.74404(11) Å, c = 13.26108(17) Å, $\alpha = \beta = \gamma =$ 90°, V = 903.879(19) Å^3, T = 140.00(10) K, Z = 4, Z' = 1, μ(Cu K$_\alpha$) = 0.747, 8239 reflections measured, 1868 unique (R_{int} = 0.0326) which were used in all calculations. The final wR_2 was 0.0718 (all data) and R_1 was 0.0265 (I≥2 σ(I)).

**Compound**	**13d**
Formula	$C_{10}H_{11}NO_2$
$D_{calc.}$/ g cm^{-3}	1.302
μ/mm^{-1}	0.747
Formula Weight	177.20
Colour	clear pale colourless
Shape	prism
Size/mm^3	0.45×0.17×0.16
T/K	140.00(10)
Crystal System	orthorhombic
Flack Parameter	0.1(2)
Hooft Parameter	-0.04(8)
Space Group	$P2_12_12_1$
a/Å	7.79506(9)
b/Å	8.74404(11)
c/Å	13.26108(17)
α/°	90
β/°	90
γ/°	90
V/Å^3	903.879(19)
Z	4
Z'	1
Wavelength/Å	1.54184
Radiation type	Cu K$_\alpha$
Θ_{min}/°	6.062
Θ_{max}/°	75.826
Measured Refl's.	8239
Indep't Refl's	1868
Refl's I≥2 σ(I)	1831
R_{int}	0.0326
Parameters	128
Restraints	0
Largest Peak	0.198
Deepest Hole	-0.139
GooF	1.048
wR_2 (all data)	0.0718
wR_2	0.0713
R_1 (all data)	0.0272
R_1	0.0265
CCDC number	2371489

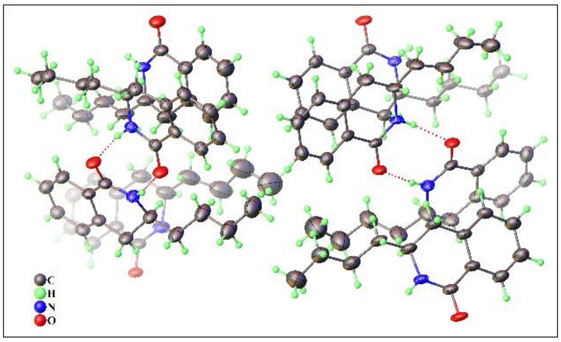

Experimental. Single colourless plate-shaped crystals of **14b** were used as supplied. A suitable crystal with dimensions 0.14 × 0.06 × 0.02 mm^3 was selected and mounted on a XtaLAB Synergy R, DW system, HyPix-Arc 150 diffractometer. The crystal was kept at a steady T = 140.01(10) K during data collection. The structure was solved with the ShelXT 2018/2 (Sheldrick, 2015) solution program using dual methods and by using Olex2 1.5 (Dolomanov et al., 2009) as the graphical interface. The model was refined with ShelXL 2019/3 (Sheldrick, 2015) using full matrix least squares minimisation on $\boldsymbol{F^2}$.

Crystal Data. $C_{13}H_{17}NO$, M_r = 203.27, monoclinic, $P2_1$ (No. 4), a = 9.7261(2) Å, b = 43.2950(9) Å, c = 10.88753(19) Å, β = 90.1176(18)°, $\alpha = \gamma$ = 90°, V = 4584.65(16) Å^3, T = 140.01(10) K, Z = 16, Z' = 8, μ(Cu K$_\alpha$) = 0.578, 59121 reflections measured, 15579 unique (R_{int} = 0.0363) which were used in all calculations. The final wR_2 was 0.3203 (all data) and R_1 was 0.0977 (I≥2 σ(I)).

**Compound**	**14b**
Formula	$C_{13}H_{17}NO$
$D_{calc.}$/ g cm^{-3}	1.178
μ/mm^{-1}	0.578
Formula Weight	203.27
Colour	colourless
Shape	plate-shaped
Size/mm^3	0.14×0.06×0.02
T/K	140.01(10)
Crystal System	monoclinic
Flack Parameter	-0.14(15)
Space Group	$P2_1$
a/Å	9.7261(2)
b/Å	43.2950(9)
c/Å	10.88753(19)
α/°	90
β/°	90.1176(18)
γ/°	90
V/Å^3	4584.65(16)
Z	16
Z'	8
Wavelength/Å	1.54184
Radiation type	Cu K$_\alpha$
Θ_{min}/°	4.060
Θ_{max}/°	75.566
Measured Refl's.	59121
Indep't Refl's	15579
Refl's I≥2 σ(I)	10255
R_{int}	0.0363
Parameters	1089
Restraints	1691
Largest Peak	0.603
Deepest Hole	-0.484
GooF	1.188
wR_2 (all data)	0.3203
wR_2	0.2894
R_1 (all data)	0.1322
R_1	0.0977
CCDC number	2359459

9. Steric Maps

Rh(I) Complex 2d

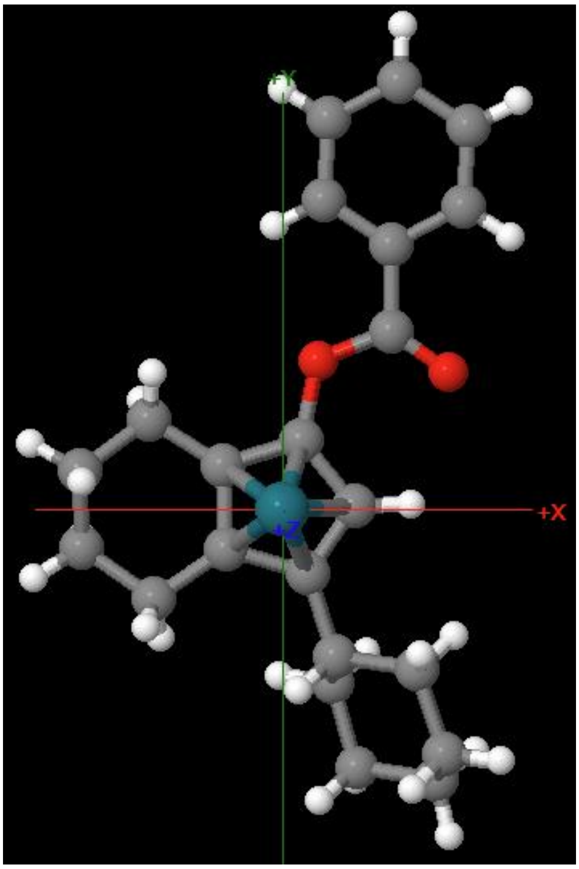

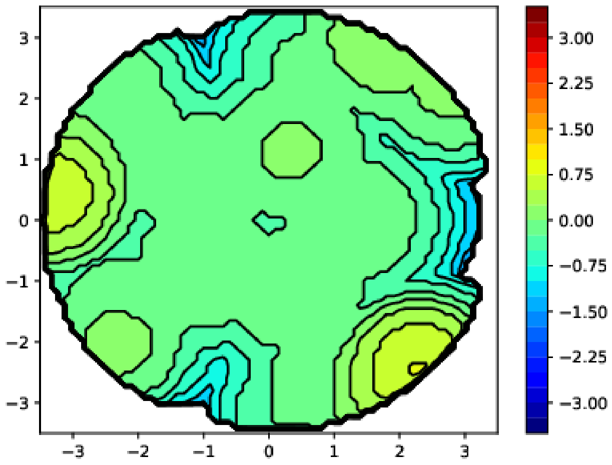

%V Free	%V Buried	% V Tot/V Ex
51.9	48.1	99.9

Quadrant	V f	V b	V t	%V f	%V b
SW	23.7	21.2	44.9	52.8	47.2
NW	23.2	21.7	44.9	51.7	48.3
NE	23.5	21.3	44.9	52.5	47.5
SE	22.8	22.1	44.9	50.7	49.3

Rh(I) Complex 2f

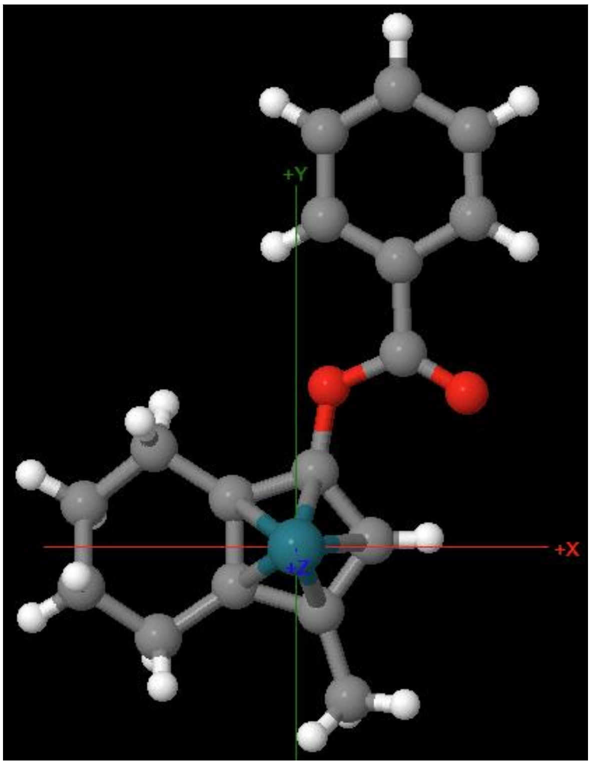

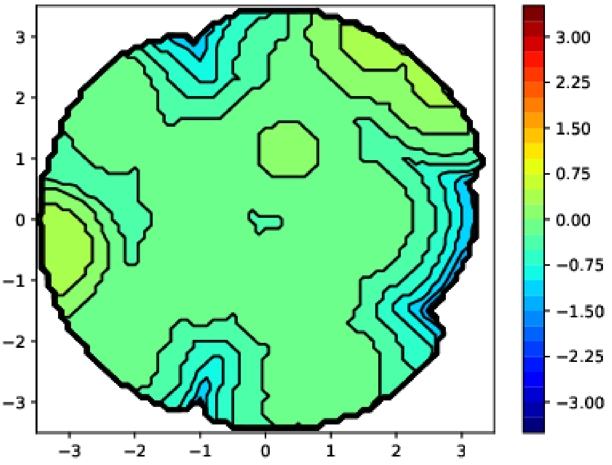

%V Free	%V Buried	% V Tot/V Ex
53.8	46.2	99.9

Quadrant	V f	V b	V t	%V f	%V b
SW	23.6	21.2	44.9	52.6	47.4
NW	24.4	20.4	44.9	54.5	45.5
NE	23.0	21.8	44.9	51.3	48.7
SE	25.4	19.5	44.9	56.6	43.4

Rh(I) Complex 2g

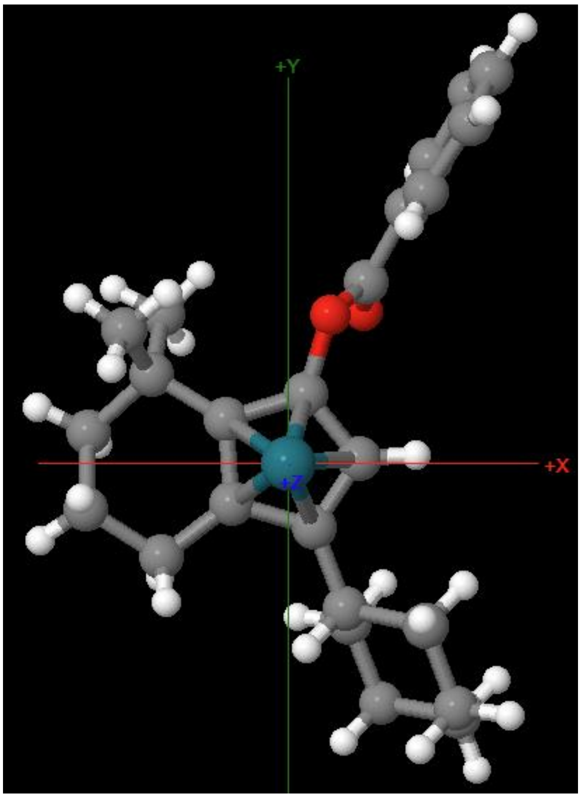

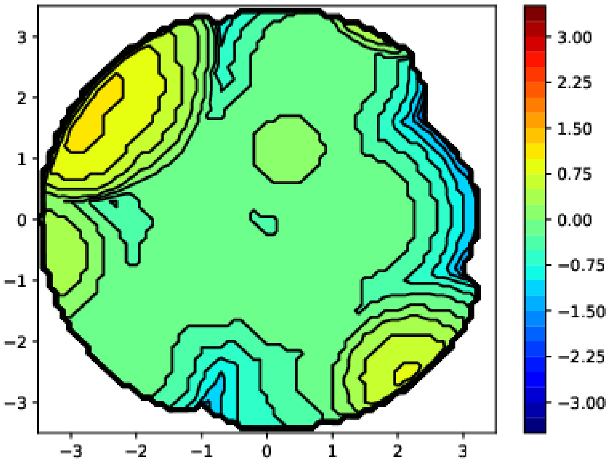

%V Free	%V Buried	% V Tot/V Ex
51.1	48.9	99.9

Quadrant	V f	V b	V t	%V f	%V b
SW	23.7	21.1	44.9	52.9	47.1
NW	19.7	25.2	44.9	43.9	56.1
NE	25.2	19.7	44.9	56.1	43.9
SE	23.1	21.8	44.9	51.4	48.6

Rh(I) Complex 2h

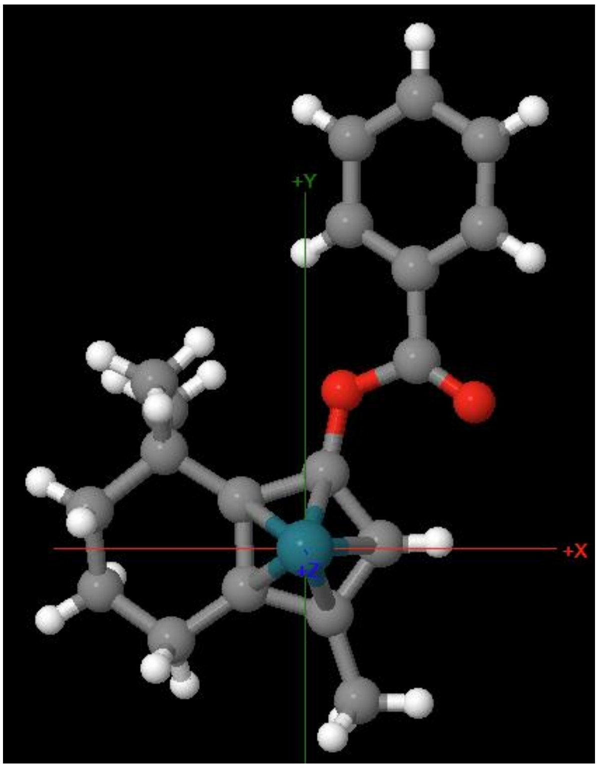

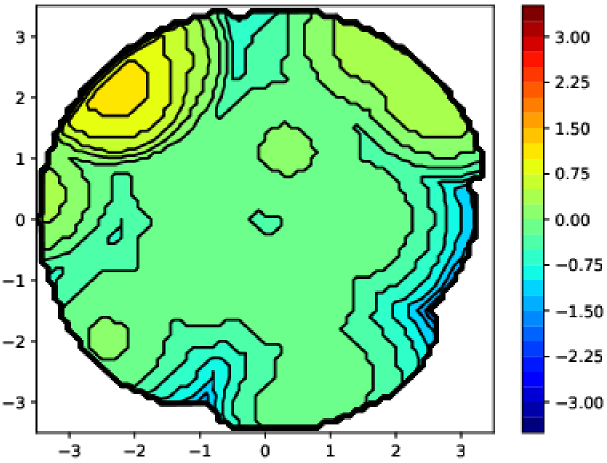

%V Free	%V Buried	% V Tot/V Ex
51.6	48.4	99.9

Quadrant	V f	V b	V t	%V f	%V b
SW	24.4	20.5	44.9	54.3	45.7
NW	20.2	24.7	44.9	45.0	55.0
NE	22.4	22.5	44.9	49.9	50.1
SE	25.7	19.2	44.9	57.3	42.7

Rh(I) Complex 2i

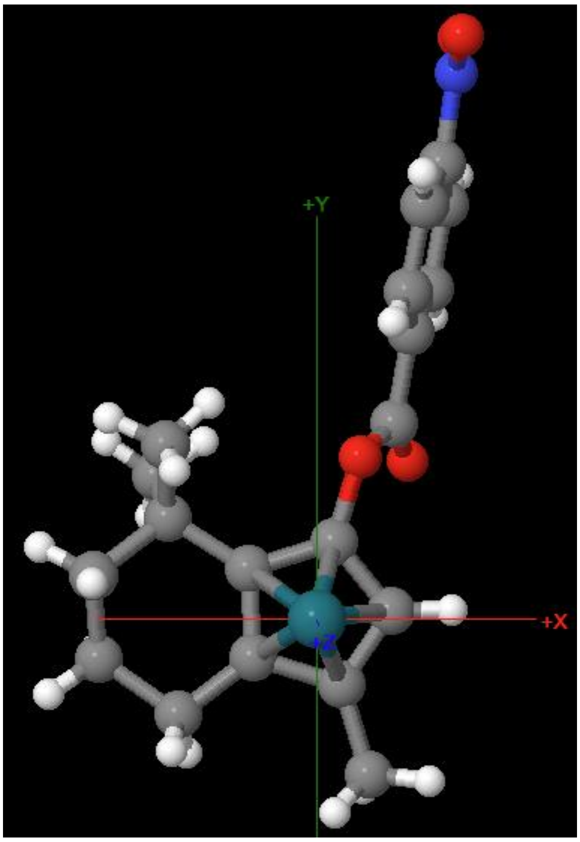

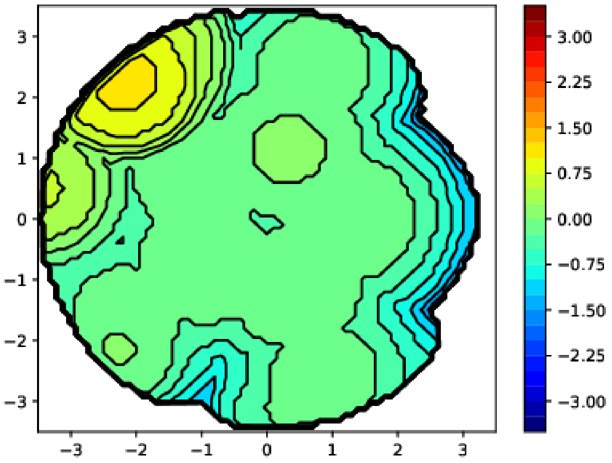

%V Free	%V Buried	% V Tot/V Ex
52.9	47.1	99.9

Quadrant	V f	V b	V t	%V f	%V b
SW	24.1	20.7	44.9	53.8	46.2
NW	19.7	25.2	44.9	43.9	56.1
NE	25.6	19.3	44.9	57.0	43.0
SE	25.6	19.3	44.9	57.0	43.0

Rh(I) Complex 2j

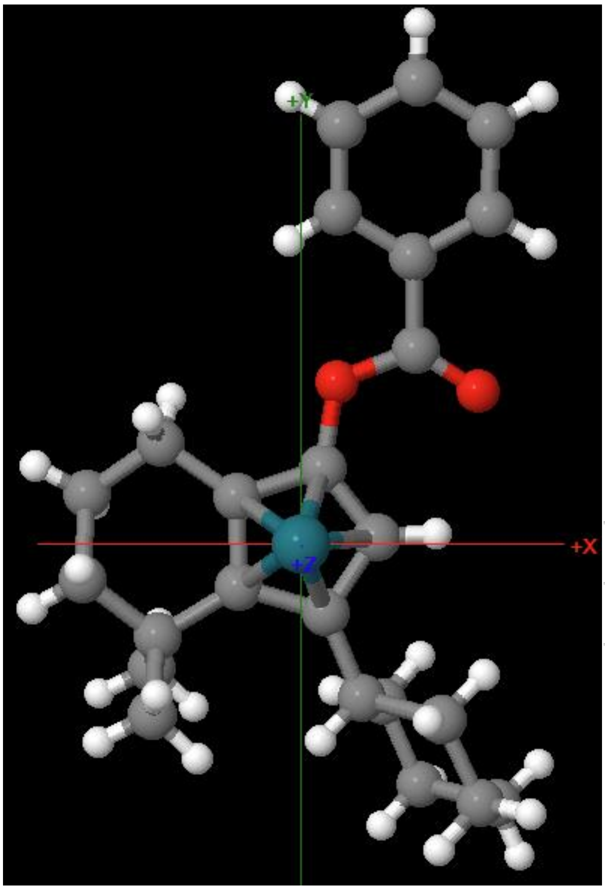

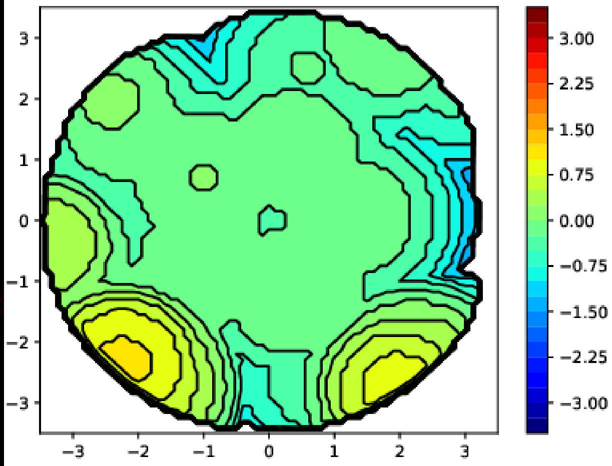

%V Free	%V Buried	% V Tot/V Ex
51.3	48.7	99.9

Quadrant	V f	V b	V t	%V f	%V b
SW	20.2	24.6	44.9	45.1	54.9
NW	24.1	20.8	44.9	53.7	46.3
NE	25.1	19.8	44.9	55.9	44.1
SE	22.6	22.2	44.9	50.5	49.5

Rh(I) Complex 2k

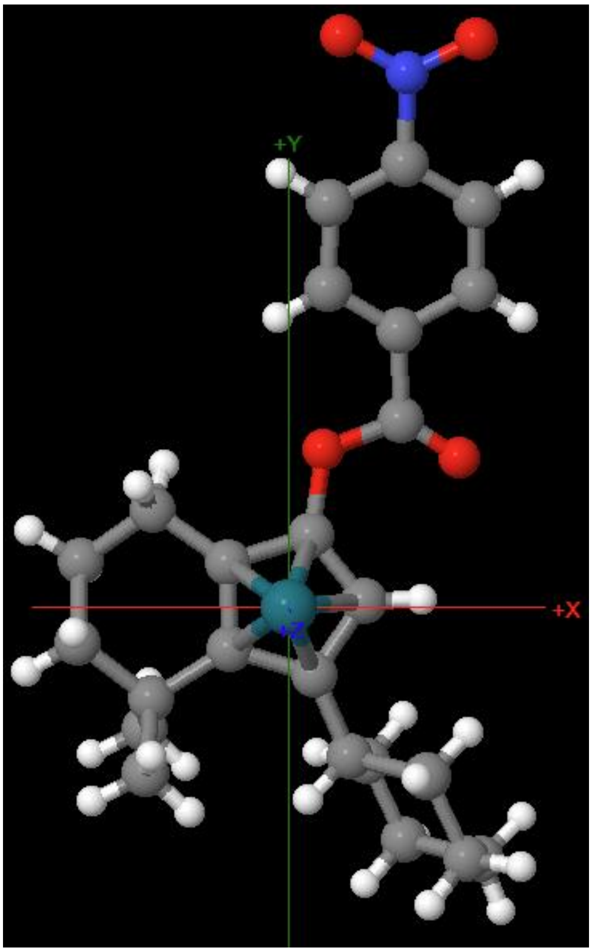

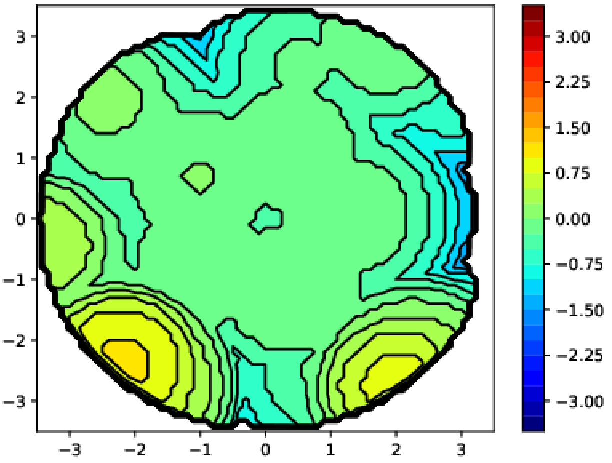

%V Free	%V Buried	% V Tot/V Ex
51.4	48.6	99.9

Quadrant	V f	V b	V t	%V f	%V b
SW	20.3	24.5	44.9	45.3	54.7
NW	24.0	20.9	44.9	53.5	46.5
NE	25.1	19.8	44.9	56.0	44.0
SE	22.8	22.1	44.9	50.7	49.3

Rh(I) Complex 2l

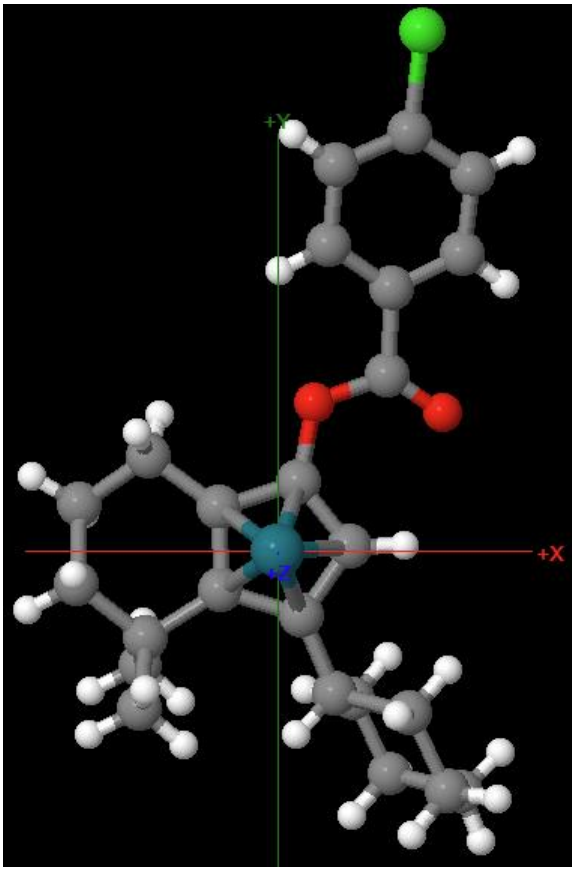

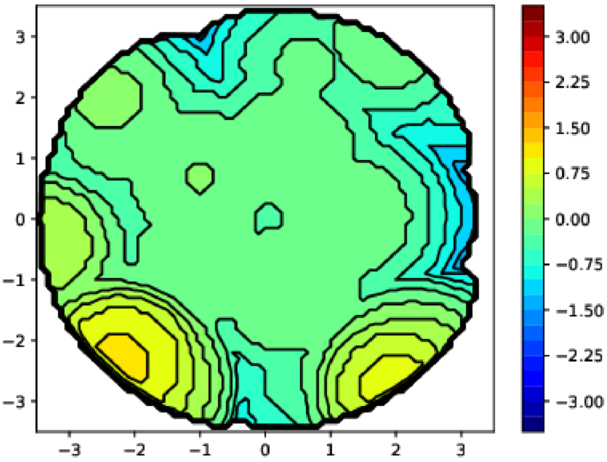

%V Free	%V Buried	% V Tot/V Ex
51.4	48.6	99.9

Quadrant	V f	V b	V t	%V f	%V b
SW	20.2	24.7	44.9	45.0	55.0
NW	24.0	20.8	44.9	53.5	46.5
NE	25.3	19.6	44.9	56.3	43.7
SE	22.7	22.2	44.9	50.6	49.4

Rh(I) Complex 2m

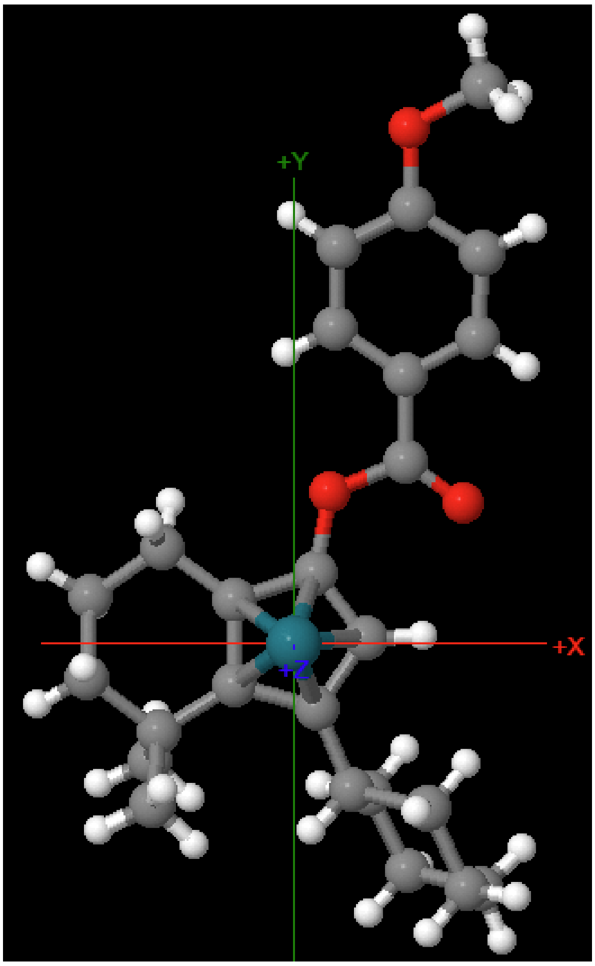

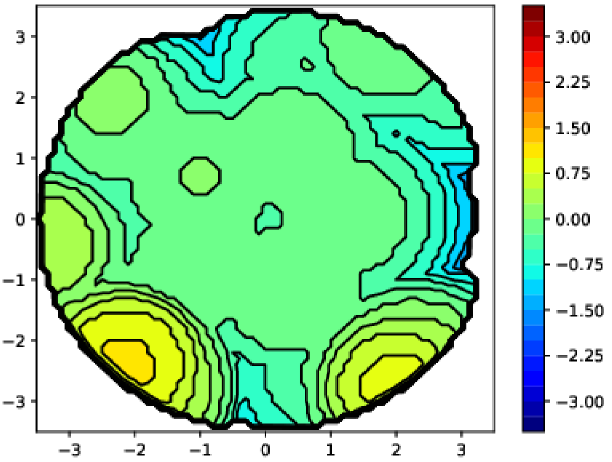

%V Free	%V Buried	% V Tot/V Ex
51.2	48.8	99.9

Quadrant	V f	V b	V t	%V f	%V b
SW	20.1	24.7	44.9	44.9	55.1
NW	23.9	20.9	44.9	53.3	46.7
NE	25.0	19.8	44.9	55.8	44.2
SE	22.7	22.1	44.9	50.7	49.3

Rh(I) Complex 2n

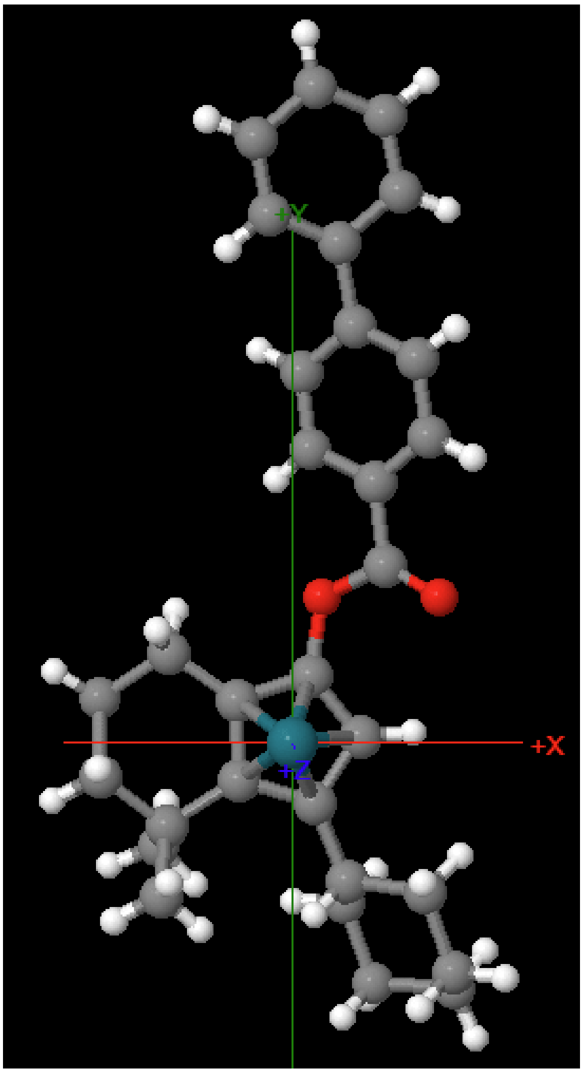

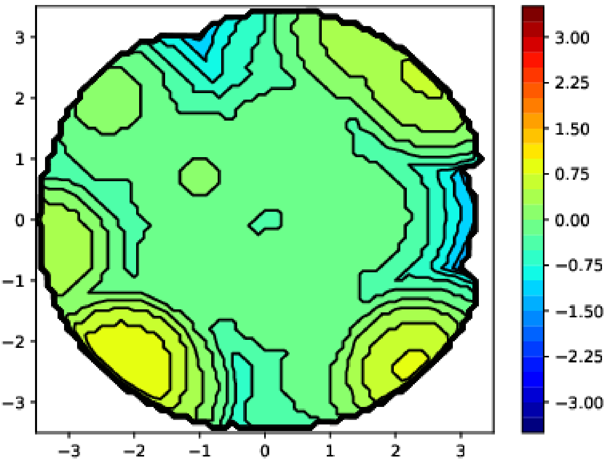

%V Free	%V Buried	% V Tot/V Ex
50.2	49.8	99.9

Quadrant	V f	V b	V t	%V f	%V b
SW	20.7	24.2	44.9	46.1	53.9
NW	24.0	20.8	44.9	53.6	46.4
NE	22.6	22.2	44.9	50.4	49.6
SE	22.8	22.1	44.9	50.8	49.2

10. References

(1) Falivene, L.; Cao, Z.; Petta, A.; Serra, L.; Poater, A.; Oliva, R.; Scarano, V.; Cavallo, L. Towards the Online Computer-Aided Design of Catalytic Pockets. *Nat. Chem.* **2019**, *11*, 872–879.

(2) Cernijenko, A.; Risgaard, R.; Baran, P. S. 11-Step Total Synthesis of (−)-Maoecrystal V. *J. Am. Chem. Soc.* **2016**, *138*, 9425–9428.

(3) Edwards, O. E.; Dixon, J.; Elder, J. W.; Kolt, R. J.; Lesage, M. Protonated Cyclopropanes in α-Aminoketone Deamination. *Can. J. Chem.* **1981**, *59*, 2096–2115.

(4) Adah, S. A.; Nair, V. Synthesis of Complex Ethynyladenosines Using Organic Triflic Enolates in Palladium-Catalyzed Reactions: Potential Agonists for the Adenosine A2 Receptor. *Tetrahedron*, **1997**, *53*, 6747–6754.

(5) Tucker, J. K.; Shair, M. D. Catalytic Allylic Oxidation to Generate Vinylogous Acyl Sulfonates from Vinyl Sulfonates. *Org. Lett.* **2019**, *21*, 2473–2476.

(6) Ishihara, K.; Nakano, K. Enantioselective [2 + 2] Cycloaddition of Unactivated Alkenes with α-Acyloxyacroleins Catalyzed by Chiral Organoammonium Salts. *J. Am. Chem. Soc.* **2007**, *129*, 8930–8931.

(7) Anand, N. K.; Carreira, E. M. A Simple, Mild, Catalytic, Enantioselective Addition of Terminal Acetylenes to Aldehydes. *J. Am. Chem. Soc.* **2001**, *123*, 9687–9688.

(8) Skepper, C. K.; MacMillan, J. B.; Zhou, G.-X.; Masuno, M. N.; Molinski, T. F. Chlorocyclopropane Macrolides from the Marine Sponge Phorbas Sp. Assignment of the Configurations of Phorbasides A and B by Quantitative CD. *J. Am. Chem. Soc.* **2007**, *129*, 4150–4151.

(9) a) Sasaki, H.; Boyall, D.; Carreira, E. M. Facile, Asymmetric Addition of Acetylene to Aldehydes: In Situ Generation of Reactive Zinc Acetylide. *Helv. Chim. Acta*, **2001**, *84*, 964–971. b) Kim, C.; Lee, J.; Cho, J.; Oh, Y.; Choi, Y. K.; Choi, E.; Park, J.; Kim, M.-J. Kinetic and Dynamic Kinetic Resolution of Secondary Alcohols with Ionic-Surfactant-Coated Burkholderia Cepacia Lipase: Substrate Scope and Enantioselectivity. *J. Org. Chem.* **2013**, *78*, 2571–2578.

(10) Frantz, D. E.; Fässler, R.; Carreira, E. M. Facile Enantioselective Synthesis of Propargylic Alcohols by Direct Addition of Terminal Alkynes to Aldehydes. *J. Am. Chem. Soc*, **2000**, *122*, 1806–1807.

(11) Huang, W.-C.; Liu, W.; Wu, X.-D.; Ying, J.; Pu, L. Enantioselective Alkyne Addition to Aliphatic, Aromatic, and Vinyl Aldehydes Using Zn, ^{i}PrI, H_8BINOL, and Ti(O^{i}Pr)$_4$. *J. Org. Chem.* **2015**, *80*, 11480–11484.

(12) Birman, V. B.; Guo, L. Kinetic Resolution of Propargylic Alcohols Catalyzed by Benzotetramisole. *Org. Lett.* **2006**, *8*, 4859–4861.

(13) Arai, N.; Satoh, H.; Utsumi, N.; Murata, K.; Tsutsumi, K.; Ohkuma, T. Asymmetric Hydrogenation of Alkynyl Ketones with the η6-Arene/TsDPEN–Ruthenium(II) Catalyst. *Org. Lett.* **2013**, *15*, 3030–3033.

(14) Zhao, K.; Hsu, Y.-C.; Yang, Z.; Liu, R.-S.; Zhang, L. Gold-Catalyzed Synthesis of Chiral Cyclopentadienyl Esters via Chirality Transfer. *Org. Lett.*, **2020**, *22*, 6500–6504.

(15) Chan, K. T.; Tong, G. S. M.; To, W.-P.; Yang, C.; Du, L.; Phillips, D. L.; Che, C.-M. The Interplay between Fluorescence and Phosphorescence with Luminescent Gold(I) and Gold(III) Complexes Bearing Heterocyclic Arylacetylide Ligands. *Chem. Sci.*, **2017**, *8*, 2352–2364.

(16) Obradors, C.; Leboeuf, D.; Aydin, J.; Echavarren, A. M. Gold(I)-Catalyzed Macrocyclization of 1,n-Enynes. *Org. Lett.*, **2013**, *15*, 1576–1579.
(17) Denmark, S. E.; Habermas, K. L.; Hite, G. A. Silicon-Directed Nazarov Cyclizations. Part V. Substituent and Heteroatom Effects on the Reaction. *Helv. Chim. Acta*, **1988**, *71*, 168–194.
(18) Fast, C. D.; Schley, N. D. Light-Promoted Transfer of an Iridium Hydride in Alkyl Ether Cleavage. *Organometallics*, **2021**, *40*, 3291–3297.
(19) Trifonova, E. A.; Ankudinov, N. M.; Kozlov, M. V.; Sharipov, M. Y.; Nelyubina, Y. V.; Perekalin, D. S. Rhodium(III) Complex with a Bulky Cyclopentadienyl Ligand as a Catalyst for Regioselective Synthesis of Dihydroisoquinolones through C−H Activation of Arylhydroxamic Acids. *Chem. Eur. J.* **2018**, *24*, 16570–16575.
(20) Lin, W.; Li, W.; Lu, D.; Su, F.; Wen, T.-B.; Zhang, H.-J. Dual Effects of Cyclopentadienyl Ligands on Rh(III)-Catalyzed Dehydrogenative Arylation of Electron-Rich Alkenes. *ACS Catal.* **2018**, *8*, 8070–8076.
(21) Tellers, D. M.; Yung, C. M.; Arndtsen, B. A.; Adamson, D. R.; Bergman, R. G. Electronic and Medium Effects on the Rate of Arene C−H Bond Activation by Cationic Ir(III) Complexes. *J. Am. Chem. Soc.* **2002**, *124*, 1400–1410.
(22) Ye, B.; Cramer, N. Chiral Cyclopentadienyl Ligands as Stereocontrolling Element in Asymmetric C–H Functionalization. *Science*, **2012**, *338*, 504–506.
(23) Pan, C.; Yin, S.; Wang, S.; Gu, Q.; You, S. Oxygen-Linked Cyclopentadienyl Rhodium(III) Complexes-Catalyzed Asymmetric C−H Arylation of Benzo[*h*]Quinolines with 1-Diazonaphthoquinones. *Angew. Chem. Int. Ed.* **2021**, *60*, 15510–15516.
(24) Brauns, M.; Cramer, N. Efficient kinetic resolution of sulfur-stereogenic sulfoximes exploiting Cp^XRh^{III}-catalyzed C-H functionalization. *Angew. Chem. Int. Ed.* **2019**, *58*, 8902-8906.
(25) Amouri, H.; Gruselle, M.; Jaouen, G. bis[Dichloro(η-pentamethylcyclopentadienyl)rhodium(III) and –iridium(III)] from bis[Chloro(1, 5-cyclooctadiene)rhodium(I) and –iridium(I)] Oxidation, and Formation of 1, 5-Cyclooctadiene(η-pentamethylcyclopentadienyl)rhodium(I). *Synth. React. Inorg. Met.-Org. Chem*, **1994**, *24*, 395-400.
(26) Ozols, K.; Jang, Y.-S.; Cramer, N. Chiral Cyclopentadienyl Cobalt(III) Complexes Enable Highly Enantioselective 3d-Metal-Catalyzed C–H Functionalizations. *J. Am. Chem. Soc.* **2019**, *141*, 5675–5680.
(27) Xia, C.; White, A. J. P.; Hii, K. K. M. Synthesis of Isoindolinones by Pd-Catalyzed Coupling between N-Methoxybenzamide and Styrene Derivatives. *J. Org. Chem.* **2016**, *81*, 7931–7938.
(28) Kolos, A. V.; Nelyubina, Y. V.; Sundararaju, B.; Perekalin, D. S. Synthesis of Overloaded Cyclopentadienyl Rhodium(III) Complexes via Cyclotetramerization of Tert-Butylacetylene. *Organometallics*, **2021**, *40*, 3712–3719.
(29) Guimond, N.; Gorelsky, S. I.; Fagnou, K. Rhodium(III)-Catalyzed Heterocycle Synthesis Using an Internal Oxidant: Improved Reactivity and Mechanistic Studies. *J. Am. Chem. Soc.* **2011**, *133*, 6449–6457.
(30) Han, H.; Zhang, T.; Yang, S.-D.; Lan, Y.; Xia, J.-B. Palladium-Catalyzed Enantioselective C–H Aminocarbonylation: Synthesis of Chiral Isoquinolinones. *Org. Lett.* **2019**, *21*, 1749–1754.
(31) Grunewald, G. L.; Caldwell, T. M.; Li, Q.; Dahanukar, V. H.; McNeil, B.; Criscione, K. R. Enantiospecific Synthesis of 3-Fluoromethyl-, 3-Hydroxymethyl-, and 3-Chloromethyl-

1,2,3,4-Tetrahydroisoquinolines as Selective Inhibitors of Phenylethanolamine N-Methyltransferase versus the α2-Adrenoceptor. *J. Med. Chem.* **1999**, *42*, 4351–4361.
(32) Yamashita, M.; Yamada, K.; Tomioka, K. Construction of Arene-Fused-Piperidine Motifs by Asymmetric Addition of 2-Trityloxymethylaryllithiums to Nitroalkenes: The Asymmetric Synthesis of a Dopamine D1 Full Agonist, A-86929. *J. Am. Chem. Soc.* **2004**, *126*, 1954–1955.
(33) Pedrosa, R.; Andrés, C.; Iglesias, J. M.; Pérez-Encabo, A. Diastereoselective Tandem 6-*exo* Carbolithiation Intramolecular Ring Opening in (−)-8-Aminomenthol-Derived Perhydrobenzoxazines. A New Synthesis of Enantiopure 4-Substituted Tetrahydro Isoquinolines and 2-Azabenzonorbornanes. *J. Am. Chem. Soc.* **2001**, *123*, 1817–1821.
(34) Laplaza, R.; Sobez, J.-G.; Wodrich, M. D.; Reiher, M.; Corminboeuf, C. The (Not So) Simple Prediction of Enantioselectivity – A Pipeline for High-Fidelity Computations. *Chem. Sci.* **2022**, *13*, 6858-6864.
(35) Wodrich, M. D.; Laplaza, R.; Cramer, N.; Reiher, M.; Corminboeuf, C. Toward in silico Catalyst Optimization. *Chimia* **2023**, *77*, 139-143.
(36) Sobez, J.-G.; Reiher, M. MOLASSEMBLER: Molecular Graph Construction, Modification, and Conformer Generation for Inorganic and Organic Molecules. *J. Chem. Inf. Model.* **2020**, *60*, 3884-3900.
(37) Bensberg, M.; Grimmel, S.; Sobez, J.-G.; Steiner, M.; Unsleber, J. P.; Rieher, M. qcscine/Molassembler: Release 3.0.0, 2024, https://zenodo.org/records/13372940.
(38) Stewart, J. J. P. Optimization of Parameters for Semiempirical Methods VI: More Modifications to the NDDO Approximations and Re-optimization of Parameters. *J. Mol. Model.* **2013**, *19*, 1-32.
(39) Perdew, J. P.; Burke, K.; Ernzerhof, M. Generalized Gradient Approximation Made Simple. *Phys. Rev. Lett.* **1996**, *77*, 3865-3868.
(40) Adamo, C.; Barone, V. Toward Reliable Density Functional Methods without Adjustable Parameters: The PBE0 Model. *J. Chem. Phys.* **1998**, *110*, 6158-6170.
(41) Grimme, S.; Antony, J.; Ehrlich, S.; Krieg, H. A Consistent and Accurate ab initio Parameterization of Density Functional Dispersion Correction (DFT-D) for the 94 Elements H-Pu. *J. Chem. Phys.* **2010**, *132*, 154104.
(42) Grimme, S.; Ehrlich, S.; Goerigk, L. Effect of the Damping Function in Dispersion Corrected Density Functional Theory. *J. Comput. Chem.* **2011**, *32*, 1456-1465.
(43) Weigend, F.; Ahlrichs, R. Balanced Basis Sets of Split Valence, Triple Zeta Valence and Quadruple Zeta Valence Quality for H to Rn: Design and Assessment of Accuracy. *Phys. Chem. Chem. Phys.* **2005**, *7*, 3297-3305.
(44) Marenich, A. V.; Cramer, C. J.; Truhlar, D. G. Universal Solvation Model Based on Solute Electron Density and on a Continuum Model of the Solvent Defined by the Bulk Dielectric Constant and Atomic Surface Tensions. *J. Phys. Chem. B* **2009**, *113*, 6378-6396.
(45) Laplaza, R.; Wodrich, M. D.; Corminboeuf, C. Overcoming the Pitfalls of Computing Reaction Selectivity from Ensembles of Transition States. *J. Phys. Chem. Lett.* **2024**, *15*, 7363-7370.
